# Effect of Ionic and Non-Ionic Surfactant on Bovine Serum Albumin Encapsulation and Biological Properties of Emulsion-Electrospun Fibers

**DOI:** 10.3390/molecules27103232

**Published:** 2022-05-18

**Authors:** Roksana Kurpanik, Agnieszka Lechowska-Liszka, Joanna Mastalska-Popławska, Marek Nocuń, Alicja Rapacz-Kmita, Anna Ścisłowska-Czarnecka, Ewa Stodolak-Zych

**Affiliations:** 1 Department of Biomaterials and Composites, Faculty of Materials Science and Ceramics, AGH University of Science and Technology, 30-059 Krakow, Poland; 2 Department at Cosmetology, Academy of Physical Education, 31-571 Krakow, Poland; agnieszka.liszka@awf.krakow.pl (A.L.-L.); anna.scislowska@awf.krakow.pl (A.Ś.-C.); 3 Department of Ceramics and Refractories, Faculty of Materials Science and Ceramics, AGH University of Science and Technology, 30-059 Krakow, Poland; jmast@agh.edu.pl (J.M.-P.); kmita@agh.edu.pl (A.R.-K.); 4 Department of Glass Technology and Amorphous Coatings, Faculty of Materials Science and Ceramics, AGH University of Science and Technology, 30-059 Krakow, Poland; nocun@agh.edu.pl

**Keywords:** electrospinning, emulsion, surfactants, protein, drug delivery system

## Abstract

Emulsion electrospinning is a method of modifying a fibers’ surface and functional properties by encapsulation of the bioactive molecules. In our studies, bovine serum albumin (BSA) played the role of the modifier, and to protect the protein during the electrospinning process, the W/O (water-in-oil) emulsions were prepared, consisting of polymer and micelles formed from BSA and anionic (sodium dodecyl sulfate–S) or nonionic (Tween 80–T) surfactant. It was found that the micelle size distribution was strongly dependent on the nature and the amount of the surfactant, indicating that a higher concentration of the surfactant results in a higher tendency to form smaller micelles (4–9 µm for S and 8–13 µm for T). The appearance of anionic surfactant micelles reduced the diameter of the fiber (100–700 nm) and the wettability of the nonwoven surface (up to 77°) compared to un-modified PCL polymer fibers (100–900 nm and 130°). The use of a non-ionic surfactant resulted in better loading efficiency of micelles with albumin (about 90%), lower wettability of the nonwoven fabric (about 25°) and the formation of larger fibers (100–1100 nm). X-ray photoelectron spectroscopy (XPS) was used to detect the presence of the protein, and UV-Vis spectrophotometry was used to determine the loading efficiency and the nature of the release. The results showed that the location of the micelles influenced the release profiles of the protein, and the materials modified with micelles with the nonionic surfactant showed no burst release. The release kinetics was characteristic of the zero-order release model compared to anionic surfactants. The selected surfactant concentrations did not adversely affect the biological properties of fibrous substrates, such as high viability and low cytotoxicity of RAW macrophages 264.7.

## 1. Introduction

Providing a microenvironment conducive to tissue regeneration is a major goal of the scaffold-based approach. Due to the microstructure of fibrous substrates resembling the extracellular matrix, electrospinning is one of the most extensively researched and developed methods. Moreover, apart from providing physical and/or chemical stimuli for cell adhesion, electrospun scaffolds are good carriers for targeted drug delivery [1]. Due to the high surface-to-volume ratio, the fibrous carrier can be used not only for the delivery of drugs but also of biomolecules such as proteins, peptides, growth factors, genes, hormones, etc. [2,3,4]. Proteins and peptides are of significant interest among them, not only due to their impact on tissue regeneration (as they are a basic component of the cells) but also for their role in enzymatic catalysis, metabolic processes, signal transduction, and immune response. Human serum albumin (HSA) is one of the most abundant proteins in human plasma that performs dozens of important functions, including the transport of amino acids, fatty acids, steroids, and drugs; regulation of pH and oncotic blood pressure [5,6,7]. It also removes harmful factors for the cell’s survival, such as reactive oxygen species, and protects them against lysis (in low concentrations such as 1 g/L), making it an excellent molecule for both in vitro (maintaining the eucaryotic cell cultures) and in vivo conditions. Moreover, albumin itself is used as a drug delivery agent due to its binding and transport properties. It is also influenced by high serum concentration, abundant accumulation in both benign and malignant tissues, a long half-life, frequent recirculation, non-immunogenicity, and non-toxicity. Human albumin improves colloidal stability and has ligand-binding properties. Moreover, the ability to quickly diffuse across the tumor vessels makes it a good carrier for anticancer drugs [7,8,9]. Since it is one of the first proteins to appear on the implant surface, it also affects the initial cell adhesion. Due to the resemblance of the structure to HSA, bovine serum albumin (BSA) is one of the most used model proteins for studying the reaction of the human organism to biomaterials. It can be used both as the ion or drug carrier as well as the modifier itself (e.g., in a sustained delivery system for promoting early adhesion of the human gingival fibroblasts) [5,6].

However, due to the tendency to denaturation, problems with stability and the complexity of the structure, proteins are difficult to encapsulate into the delivery system. A small conformation change caused by inappropriate electrospinning conditions (shear stress, organic solvent exposure) could lead to the inactivation of protein or even make it toxic [10]. Moreover, due to the dependence on the environmental conditions, the group of materials with which proteins can be combined is narrowed down to those that will not adversely affect their biological activity [11]. Therefore, the incorporation of proteins via blend electrospinning creates a risk of losing their biological activity as a result of contact with organic solvents. In addition, the inhomogeneous dispersion within the fibers may contribute to the burst release effect [1]. Another important point is that PCL is a highly hydrophobic polymer for which water acts as a non-solvent. Additionally, the solvent system used for its dissolution consists predominantly of water-immiscible solvents. Therefore, combining aqueous soluble agents (such as proteins) with the polymer phase is quite difficult due to the risk of phase separation. This, in turn, makes it impossible to obtain fibers due to the precipitation of the polymer during the electrospinning process.

An alternative approach to solving such a problem may involve the use of emulsion electrospinning, in which proteins are introduced into the polymer solution in the form of micelles. The use of an emulsion as an electrospinning solution enables the encapsulation of proteins in the form of an aqueous solution (water phase) suspended in a polymer phase (oil phase) with the addition of a surfactant. This form of solution allows for the separation of proteins from the solvents that are hazardous to them and, consequently, to maintain their biological activity. Moreover, contrary to monolithic fibers obtained by blend electrospinning, emulsion electrospinning enables the production of core-shell fibers that are more advantageous in sustaining the molecule’s release. The advantages offered by emulsion electrospinning have contributed to their wide application as a method of encapsulating biomolecules such as laminin (renal protein) within PCL fibers [12] or BSA and Nerve Grow Factor inside PCL fibers [13]. An interesting approach was proposed by Qi et al., who prepared beads-in-string nanofibers via electrospinning from O/W (oil-in-water) and W/O (water-in-oil) emulsions. They used Ca-alginate microspheres, prepared by the reverse emulsion method, to encapsulate BSA and incorporate it into PLLA fibers. As a result, they obtained a more sustained release compared to the neat Ca-alginate microspheres [14]. Emulsion electrospun nanofibers are also commonly used as drug delivery systems. Sanchez et al. used this method to encapsulate lidocaine hydrochloride (LH) inside PLA-based fibers using blend and emulsion electrospinning, in which the core part was made of a drug solution with PVA or water (emulsion electrospun core-shell fibers). The results showed that the type of fibers influenced the character of LH release, which was more stable for core-shell fibers composed of PVA in the core part [15]. Another example of a drug incorporated into fibers by the emulsion electrospinning method is cephalexin. Moydeen et al. used PVA blended with various biopolymers (chitosan, carboxymethyl starch, carboxymethyl cellulose, hydroxyproyl cellulose) as the shell. As a result, they obtained a release character dominated by the diffusion mechanism, which promoted wound healing [16]. Other applications include improving the solubility of poorly water-soluble drugs (such as probutol) by encapsulating them in PVA-based fibers in the presence of a surfactant (Polysorbate 80) [17], the incorporation of antioxidants (sea buckthorn) in PLA/apocynum venetum cellulose fibers [18] and in dual-sensitive drugs for antitumor treatment consisting of Ag/Au and the therapeutic drug silibinin in the core part [19].

The surfactant plays a key role in the emulsification process, as it provides a kinetic barrier preventing phase separation [20]. Moreover, it acts as a separator between the polymer phase and the water phase preventing phase separation, thus enabling the obtaining of fibers by electrospinning. Due to the presence of both hydrophilic and lipophilic groups, the surfactant has a great impact on micelle formation as well as the microstructure of the fibers. In the formulation process, its type and concentration, as well as the water-to-oil phase ratio, are of particular importance, as they determine the initial morphology of the emulsion and then the rheological and electrostatic properties [1,21,22]. Depending on its HLB value (hydrophilic-lipophilic balance), it can promote either a water-in-oil (HLB 3–6) or oil-in-water emulsion (HLB 8–18) [20]. As a result, the water phase can be introduced into the fibers in the form of either droplet (forming core-shell fibers in the electrospinning process) or as a frame for the polymer matrix (forming pseudo-core-shell fibers). In their studies on the PLGA-based core-shell fibers, Wang et al. indicated that the type of surfactant had a great impact on the formulation of the core part. Span 80 (which is a lipophilic surfactant with HLB = 4.3) tended to form a water-in-oil emulsion which resulted in a continuous core. On the other hand, the hydrophilic nature of SDS has led to the formation of a discontinuous core characterized by the presence of parallel water phase frames around the polymer. Further continuous core formation depended on the shear forces caused by stretching [1]. Moreover, depending on the surfactant type, micelles can be stabilized by either electrostatic or steric forces, which also influence their size [20,23]. In their work, Hu et al. encapsulated BSA in PCL-based core-shell fibers and studied the effect of surfactant type on the morphology and mechanical properties of the scaffold. They indicated that due to the different effects of hydrogen and electrostatic bonding dependent on the type of surfactant, it is possible to obtain fibers with different morphology and tensile strength [21]. One of the most important physical parameters of a surfactant is the critical micelle concentration (CMC), which is defined as the concentration of the surfactant at which it begins to aggregate and form micelles. Properties (such as conductivity, viscosity, polarity, density, osmotic pressure, solubilization power, etc.) can be quite different for solutions with surfactant concentrations below and above CMC. Moreover, emulsification, dispersion, and solubilization are regulated by the formation of the micelles [24], while CMC is strongly dependent on the structure of the surfactant. Changing the length of the hydrophobic chain or the polar head group affects the self-assembly of the surfactant in the aqueous solution as well as the size of the micelles [24,25]. In the case of a polymer solution, the critical aggregation concentration (CAC) of the surfactant is another important factor influencing the surface tension and rheological properties of the emulsion. Above the CAC, micelles are not yet formed, however, the surfactant molecules begin to interact with the polymer chain and form aggregates until they reach the CMC value at which micelles are formed. The CAC value is significantly lower than the CMC after adding the ionic surfactant to the ionic polymer solution. When the solution consists of a non-ionic polymer and an ionic/non-ionic surfactant, the difference between CAC and CMC is not so significant [26,27]. Surfactant concentration also has a big impact on the core-shell fiber’s formation and properties. The excess of surface-active agents, due to the charge repulsion, may move outside the fibers and spread over their surface, contributing to problems with biocompatibility and defected morphology [28]. Yazgan et al. demonstrated that depending on the concentration of the surfactant, the fibers could be either porous or have a core-shell morphology. Due to the role of the surfactant as a plasticizer, further increasing its concentration contributed to a higher jet drawing ratio, which resulted in externally smooth core-shell fibers. Moreover, the results showed that humidity had a significant influence on both internal fiber morphology and surface chemistry [29]. The high concentration of the surfactant, correlated with the high relative humidity, contributed to its relocation within the fibers, as proven by Johnson et al. The increase in the humidity of the electrospinning environment created thermodynamically favorable conditions for the relocation of surfactant excess at the polymer-air interface [30]. Other factors influencing the performance of nonwovens include the viscosity of the emulsion [22,31], the type of its homogenization, and the interaction between the protein and the surfactant [32].

The promising application potential of emulsion electrospinning contributed to its wide use in tissue engineering as an encapsulation method. Various polymers from the group of both synthetic and natural polymers have been used as carriers for introducing the active agent into the aqueous phase with the aid of either an aqueous or polymer solution. The emulsion electrospinning method have been used to encapsulate the drug (theophylline) in PLA fibers [33], antimicrobial agent (*C. majus*) in PCL/PVA_PEC (polycaprolactone/polyvinyl alcohol pectin) fibers [34], or growth factors into PLACL (poly(l-lactide-co-caprolactone)) [35] and PDLLA/PLGA [36]. These materials also include nonwovens based on polymers, such as PCL consisting of tramadol + PEO [37], L-ascorbic acid 2-phosphate magnesium (ASP) for osteogenic differentiation [38], hyaluronic acid [39], or silk fibroin [31] in either the core or shell part.

All this indicates that the preparation at the pre-spinning stage is no less important than the conditions used during the electrospinning itself. Factors such as surfactant concentration, micelle size, viscosity, and conductivity of the solution have a great impact on the fibers’ diameter, uniformity, surface chemistry, release character, and overall biological performance of the nonwovens. However, to date, the influence of a surfactant of different polarity on the properties of the emulsion and the release kinetics of the protein additive has not been fully explored. Therefore, the correlation between the properties of the pre-spinning solution and the fibrous scaffold is still of great interest. Moreover, since the use of surfactants is of concern due to their potential adverse effect on protein activity and cell adhesion, the effect of emulsion electrospun fibers on cell viability needs further investigation.

In our research, core-shell fibers based on PCL enriched with BSA were produced using emulsion electrospinning. In order to limit the adverse effect of organic solvents on the encapsulated protein, two types of surfactants differing in the polarity of the hydrophilic head, molecular mass, and CMC were used: non-ionic, macromolecular (Tween 80) and anionic, micromolecular (SDS). Tween 80 is a hydrophilic surfactant with HLB = 15 and is considered an emulsifier, while SDS (empirical HLB = 40) is also a hydrophilic surfactant, however, its affinity to the water phase is characteristic of solubilizers. Tween 80 has a molecular mass of 1 310 g/mol (the highest among the “Tween family”) and a CMC value ~0.015 mM at room temperature (~25 °C) [24], while SDS has a molecular mass of 288 g/mol, which is significantly lower and a CMC ~0.008 M [24,40]. This paper focuses on the influence of both the type and concentration of surfactant on the physicochemical and rheological properties of emulsion and its correlation with the physicochemical and biological properties of nonwovens. The aim of the research was to obtain a substrate characterized by high encapsulation efficiency, extended-release time, and increased cell viability.

## 2. Results

### 2.1. Characterization of the Electrospinning Solution

The results of conductivity studies (Table 1) show significant differences in the conductance of the emulsion, depending on both the type of surfactant and its concentration. The use of Tween 80 as a stabilizer slightly increased the conductivity of the emulsion to 1.9 µS/cm for the T1 sample, which increased up to 5.4 µS/cm for the higher concentration (T4). On the other hand, the addition of anionic SDS contributed to a huge increase in conductivity, up to 140.5 µS/cm for the S1 sample and 183 µS/cm for the S4 sample.

**Figure 1:**
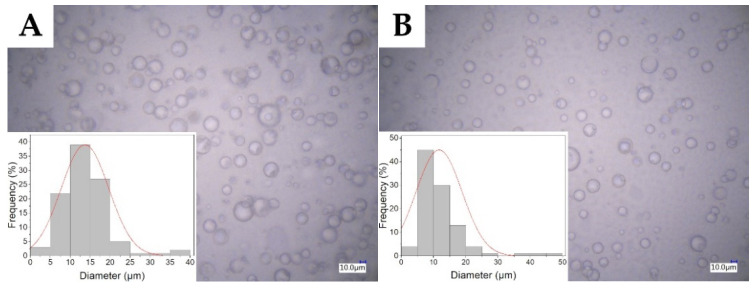

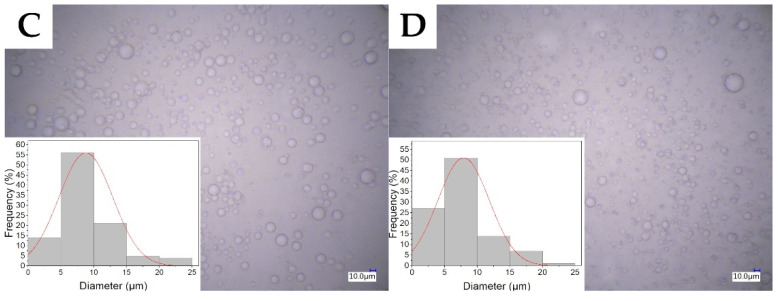
Bright field microscopy pictures and size distribution of surfactant stabilized dispersed phase for emulsions with addition of 0.1% Tween 80 (**A**), 0.4% Tween 80 (**B**), 0.1% SDS (**C**) and 0.4% SDS (**D**).

**Figure 2:**
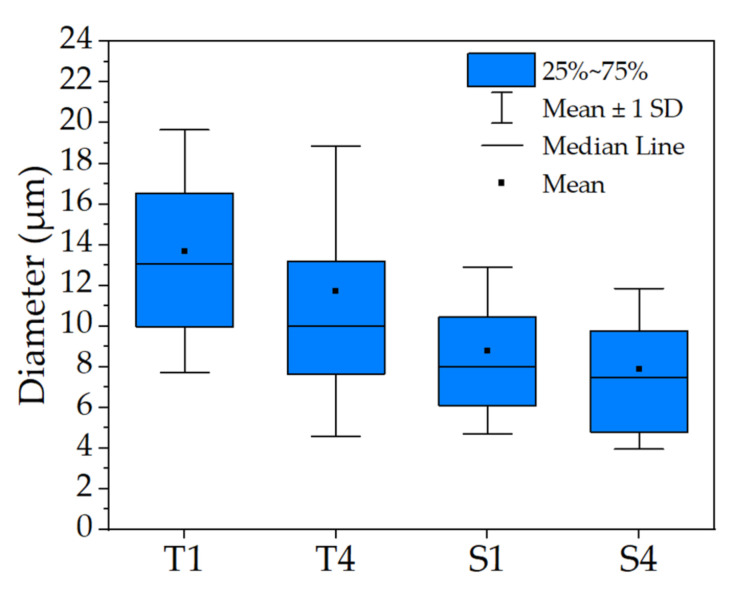
Micelle size distribution for all emulsions. Results are based on the measurement of N = 100 micelles from the microscopic picture for each sample. Standard deviation is shown as error bars.

Images of micelles from an optical microscope are presented in Figure 1. Spherical micelles were obtained for all samples, which differed in size depending on the type of surfactant used. Micelles in T1 and T4 emulsions were characterized by a wider range of size distribution with the presence of micelles with a significantly larger diameter deviating from the mean value (Figure 1a,b). In the case of S1 and S4 emulsions, a more symmetrical diameter size distribution was indicated on more uniform micelles obtained by SDS (Figure 1c,d). The micelle size distributions expressed as the interquartile range, mean and standard deviation (SD) are presented in Figure 2. Among the emulsions obtained, two groups of significantly different micelles sizes can be distinguished (ANOVA, α = 0.01, N = 100). The first one consisted of emulsions containing non-ionic surfactants that showed a larger micelle diameter with an interquartile range of ~6 µm and a mean of ~12–14 µm. The second group was emulsions containing anionic surfactant. The addition of SDS contributed to the formation of micelles with an interquartile range of ~5 µm and a mean diameter of ~8–9 µm (Table 1). Moreover, the micelle diameters did not differ significantly in the samples containing the same surfactant but different concentrations (ANOVA, α = 0.01, N = 100).

**Figure 3:**
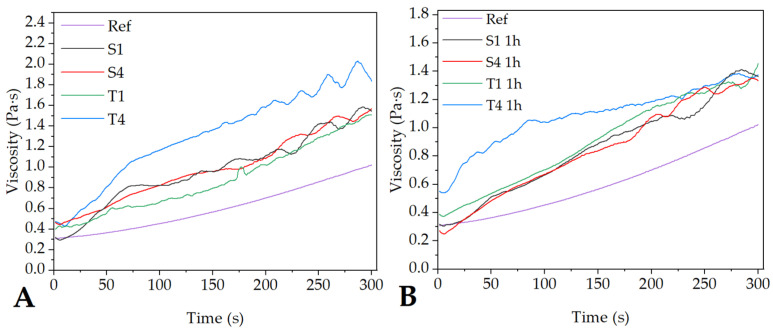
The viscosity of the emulsions immediately after preparation (**A**) and after 1 h (**B**).

The viscosity measurement results, presented in Figure 3, also depend on the type of surfactant. In the case of samples containing SDS, the concentration immediately after preparation did not have a significant effect on the viscosity, which is almost equal for both of them (S1 and S4). For samples containing Tween 80, the results differed depending on the concentration of the surfactant. Although the viscosity of the T1 sample was similar to the S1 and S4 samples, the results for the T4 sample were significantly higher than the rest of the emulsion (Figure 3A).

However, the results obtained one hour after the preparation of the emulsion (Figure 3B) showed a strong decrease in the viscosity of the T4 emulsion, while the remaining samples maintained their stability. This indicates a tendency of Tween 80 to form small micelles in the first moments after the formation of the emulsion, which, however, quickly destabilize (due to, for example, coalescence or flocculation) [37]. Nevertheless, all samples exhibit a higher viscosity than the reference sample regardless of the measuring time.

### 2.2. Fiber Morphology

**Figure 4:**
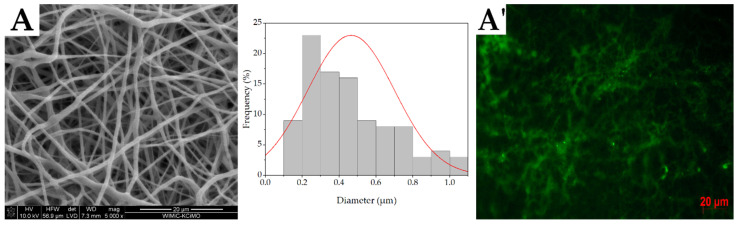

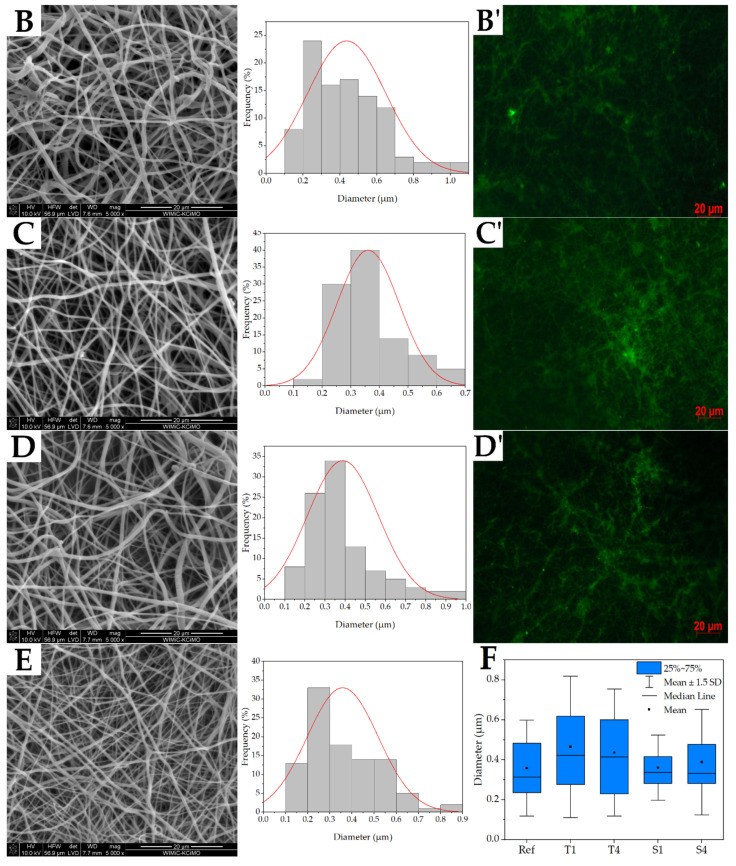
SEM micrographs, fibers size distribution (**A**–**E**), and fluorescence microscope images (**A’**–**D’**) of T1 (**A**,**A’**), T4 (**B**,**B’**), S1 (**C**,**C’**), S4 (**D**,**D’**), and PCL (**E**) fibrous scaffolds. Box chart of the fibers’ size distribution (**F**).

SEM micrographs of emulsion electrospun nonwovens are presented in Figure 4A–F. All samples exhibited a unimodal fiber diameter distribution, which was strongly correlated with the micelle size distribution (r = 0.92, r^2^ = 0.85). Samples containing Tween 80 had the largest fiber diameter and the widest range up to 1.1 µm regardless of the surfactant concentration. The mean diameter and interquartile range for the T1 and T4 samples (Figure 4F), which were ~0.5 µm and ~0.4 µm, respectively, were significantly different from neat PCL fibers as well as the S1 and S4 samples (ANOVA, α = 0.05, N = 100). On the other hand, the fibers with the addition of SDS were characterized by a more uniform size with a smaller diameter and a narrower range of diameter sizes. The interquartile range of the S1 and S4 samples was ~0.1 µm and ~0.2 µm, respectively, (mean diameter ~0.4 µm) (Figure 4F). Contrary to the samples containing Tween 80, no significant difference was observed with the neat PCL fibers (ANOVA, α = 0.05, N = 100).

Images obtained from a fluorescent microscope are presented in Figure 4A’–D’. The green areas visible in the pictures indicate the presence of a fluorescent dye (fluorescein). Since it was introduced only into the aqueous phase, it also indicates the localization of the core part of the fibers. In general, the lack of empty spaces indicates the non-domain nature of fluorescein localization and the uniform distribution of BSA within the emulsion electrospun fibers. However, there are several fluorescein aggregation spots that could indicate sites with an incompletely developed core.

### 2.3. Structure of Nonwovens

**Figure 5:**
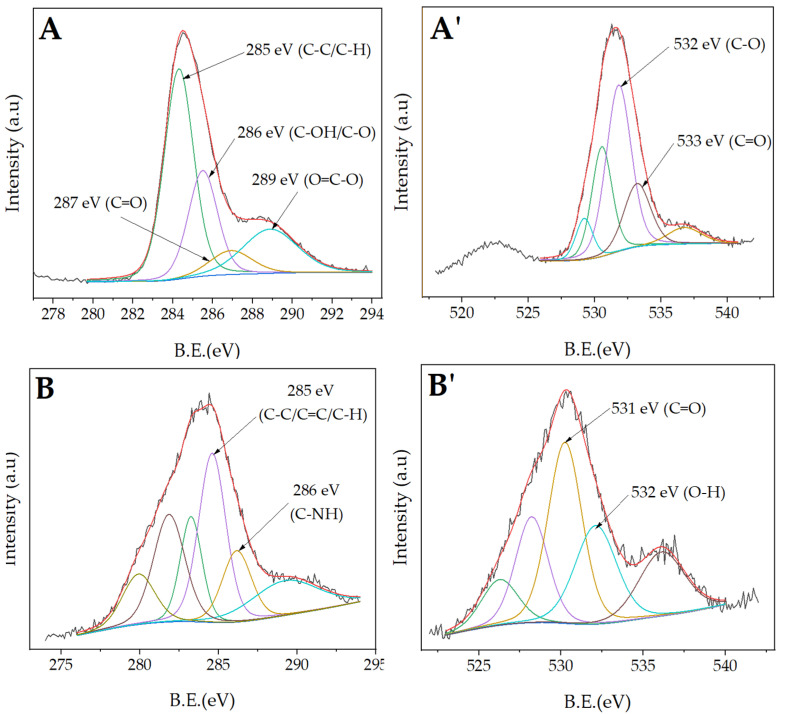

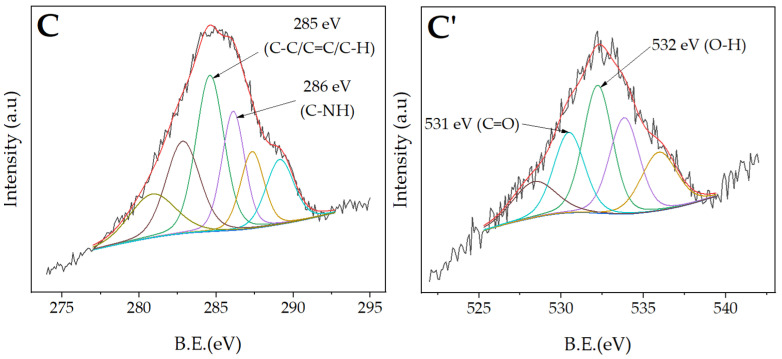
C1s (**A**–**C**) and O1s (**A’**–**C’**) deconvoluted spectra of neat PCL fibers (**A**,**A’**), T1 (**B**,**B’**), and S1 (**C**,**C’**).

To determine the presence of the protein inside the fibers, an X-ray photoelectron spectroscopic study was carried out. The XPS spectra of the emulsion electrospun nonwoven are presented in Figure 5. The Ref sample yield all the valence bands characteristic of neat PCL fibers. For high-resolution C1s spectra, there are binding energy peaks of 285 eV (C-C/C-H), 286 eV (C-OH/C-O), 287 eV (C=O) and 289 eV (O=C-O) [41]. For high-resolution O1s spectra, the 532 eV and 533 eV peaks correspond to C-O and C=O bonds, respectively [42]. As for the emulsion electrospun samples, they all consist of 285 eV (C-C, C=C, C-H) and 286 eV (C-NH) peaks characteristic of BSA on C1s spectra. The C=O and O-H bonds are located at around 531 eV and 532 eV, respectively [43].

### 2.4. Wettability

**Figure 6:**
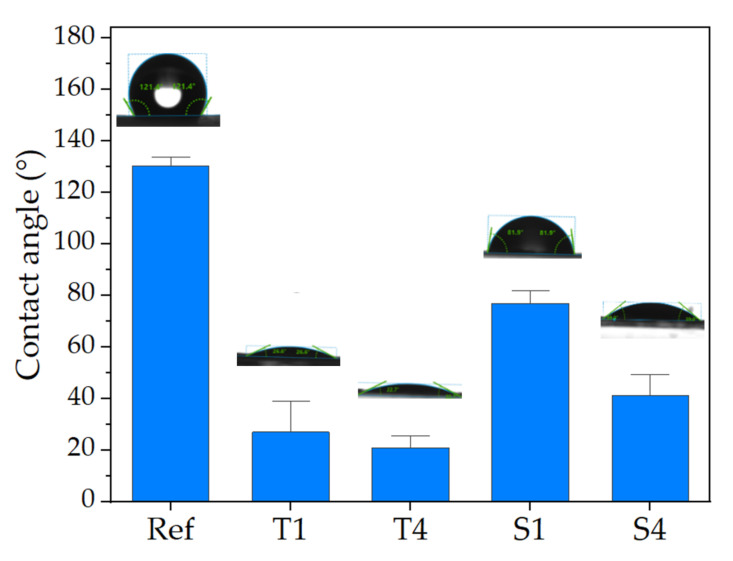
The contact angle of nonwovens obtained from emulsions containing various types and concentrations of surfactants. The graph shows the mean and standard deviation of the contact angle for deionized water (N = 10).

The contact angle of the samples is presented in Figure 6. The results show that the nature of the fibers' surface completely changed regardless of the type or concentration of the surfactant. Contrary to neat PCL fibers, which were highly hydrophobic (~130°), the contact angle of the emulsion electrospun nonwovens was within the range characteristic of hydrophilic surfaces. The strongest increase in wettability was shown by the samples modified with Tween 80. The mean contact angle for the T1 sample was ~27°, which was reduced up to ~20° for the T4 sample. In the case of samples containing SDS, the change in wettability was not as drastic as for the samples containing Tween 80. The contact angle for the S1 sample was ~77°, which is characteristic for surfaces considered to be moderately hydrophilic. A further increase in the surfactant concentration contributed to the reduction of the contact angle value up to ~41° for the S4 sample. In the case of anionic surfactants, the effect of its concentration on the wettability of the nonwoven surface was more noticeable than for non-ionic surfactants. For the T1 and T4 samples, no significant difference was observed (ANOVA, α = 0.05, N = 100).

### 2.5. Efficiency of Encapsulation and Release Studies

The loading efficiency of BSA into the core-shell fibers is presented in Table 2. The results show that the selection of the surfactant affects the loading and encapsulation efficiency. The most successful BSA encapsulation exhibited samples containing anionic surfactants (46.4% for the S1 sample and 52.4% for the S4 sample). In both cases, the LE and EE values increased with increasing surfactant concentration, however, for samples containing SDS the difference is more noticeable (the difference within one type of surfactant was 1.12% and 6% for the “T” and “S” groups).

**Figure 7:**
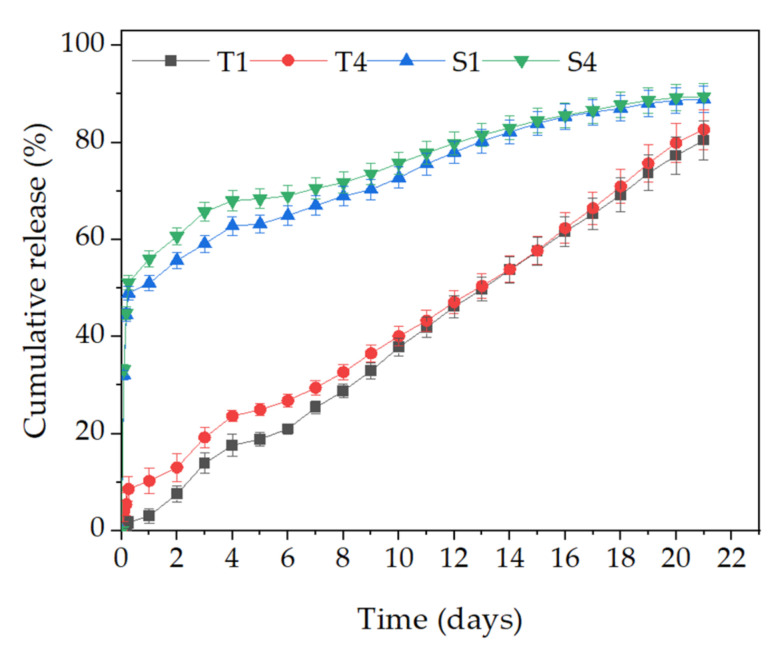
The cumulative BSA release profiles. The graph shows the mean and standard deviations for the BSA release. Each measurement was performed in triplicate.

The BSA cumulative release curves from the emulsion electrospun fibers are presented in Figure 7. The results show that character of BSA release was strongly dependent on the choice of surfactant. The shape of the release curve was similar within one type of surfactant regardless of its concentration, but differed for the two different types of surfactant. In the case of the anionic surfactant (S1 and S4), burst release occurred in the initial stage of incubation (first six hours). The ejection phase (up to ~50% of the initial amount of BSA in the fibers) was followed by the constant release of the peptide over a period of 14 days until reaching ~90% of the initial mass. On the other hand, the T1 and T4 samples exhibited the constant release of a small amount of BSA from the beginning up to the 21st day of incubation.

### 2.6. BSA Release Kinetics

**Figure 8:**
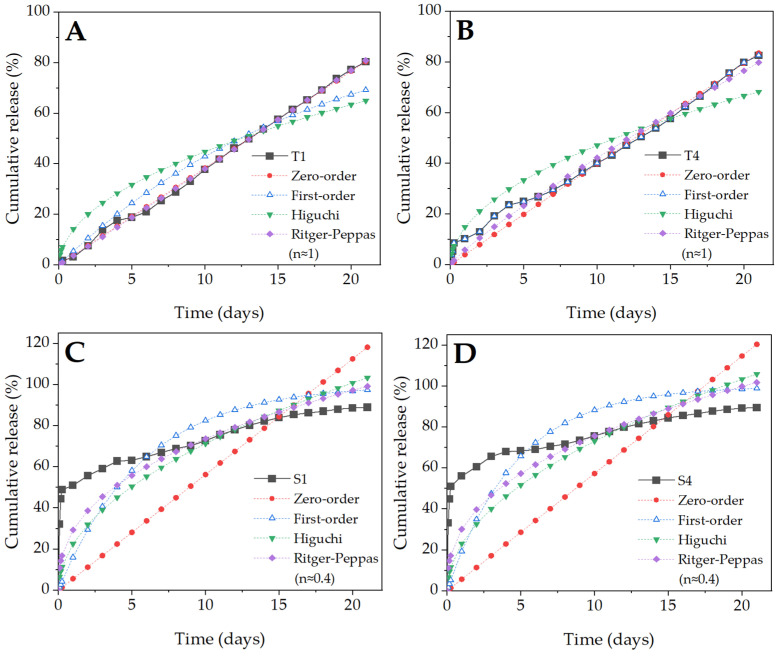
In vitro release profiles of the emulsion electrospun samples: T1 (**A**), T4 (**B**), S1 (**C**), and S4 (**D**) with fitted curves based on four kinetic equations. The experimental data were compared with four models: zero-order, first-order, Higuchi, and Ritger-Peppas. The best fit was obtained by optimizing the K value in order to minimize the mean square error.

**Figure 9:**
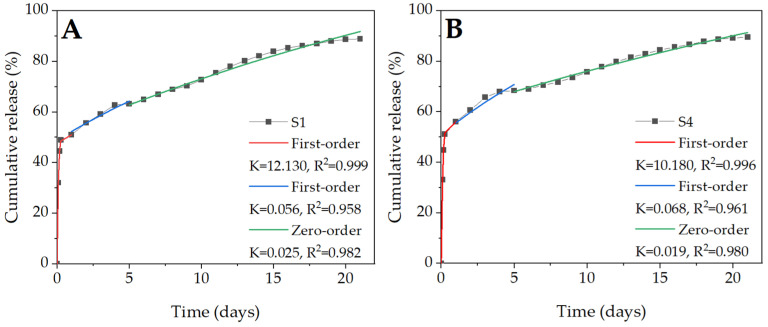
The three-phase model of BSA release for the S1 (**A**) and S4 (**B**) samples. The experimental data were divided into intervals (corresponding to different phases), which were compared with the zero-order, first-order, and Higuchi models. The best fit was obtained by optimizing the K value in order to minimize the mean square error.

The fitted release curves, according to the four kinetic equations (zero-order, first-order, Higuchi, and Ritger-Peppas) are shown in Figure 8. Both samples containing Tween 80 (Figure 8A,B) exhibited linear BSA release. Moreover, for both, the highest R^2^ values were recorded for fit to the zero-order release model (0.999 for T1 and 0.993 for T4). The K-values (the tangent to the graph of the release model), indicating the rate of the burst release phenomenon, did not differ significantly and were rather low (Table 3). As for fibers containing SDS (Figure 8C,D), the release kinetic was completely different and cannot be described by a zero-order model. Unlike the “T” samples, the curve has several inflexion points. Moreover, all R2 values were quite low (the best fitting had values close to 0.999), which indicated an inaccurate fitting of the models (Table 3). All this indicates the more complex nature of the release, which can be divided into several phases controlled by different processes. A significant improvement in the determination coefficient (R^2^ value increased above 0.9) was observed after dividing the model into three phases (Figure 9), which indicates that the release kinetic from the S1 and S4 samples was more complex. This model illustrates much better a sudden burst in the initial phase (high K value) of incubation and a gradual release in the following days.

### 2.7. In Vitro Study

**Figure 10:**
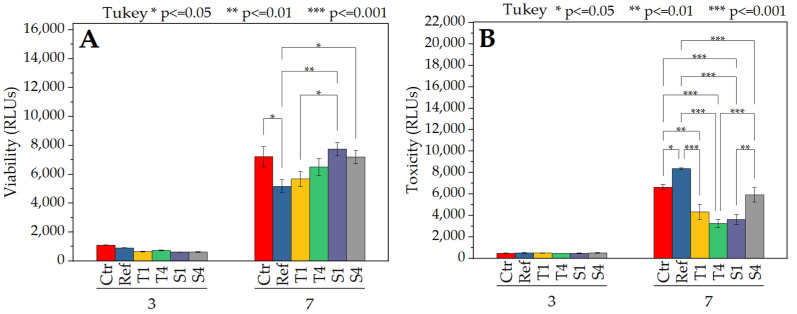
RAW 264.7 mouse macrophages’ viability (**A**) and cytotoxicity (**B**) on all samples. Tests were conducted with the use of tissue culture polystyrene (Ctr), neat PCL fibers (Ref), and samples obtained by the emulsion electrospinning. Results of one-way ANOVA analysis with post hoc Tukey test.

The results of the in vitro test of the samples are presented in Figure 10. On the 3rd day of culture, macrophages viability did not differ in all samples (Figure 10A). There was also no significant difference between the samples and the control or the reference. On the 7th day of culture, the viability increased significantly. The S1 and S4 samples showed the highest viability, which was equal or even higher than the control. In the case of samples T1 and T4, a high increase in viability was also observed. In the case of samples modified with the same surfactant, the viability was similar and fell within the error limit. All emulsion electrospun samples exhibited a higher viability than the reference sample (non-modified PCL fibers).

As with the viability test, the cytotoxicity on the 3rd day of incubation did not differ in all samples (Figure 10B). On the 7th day of culture, the reference sample showed the most detrimental action towards the cells. Samples T1, T4, and S1 showed the lowest cytotoxicity among the samples of electrospun emulsion at the same level of significance. The toxicity of the S4 sample was slightly higher, but it was also comparable to the control. Overall, all BSA-containing nonwovens exhibited the lowest cytotoxicity.

**Figure 11:**
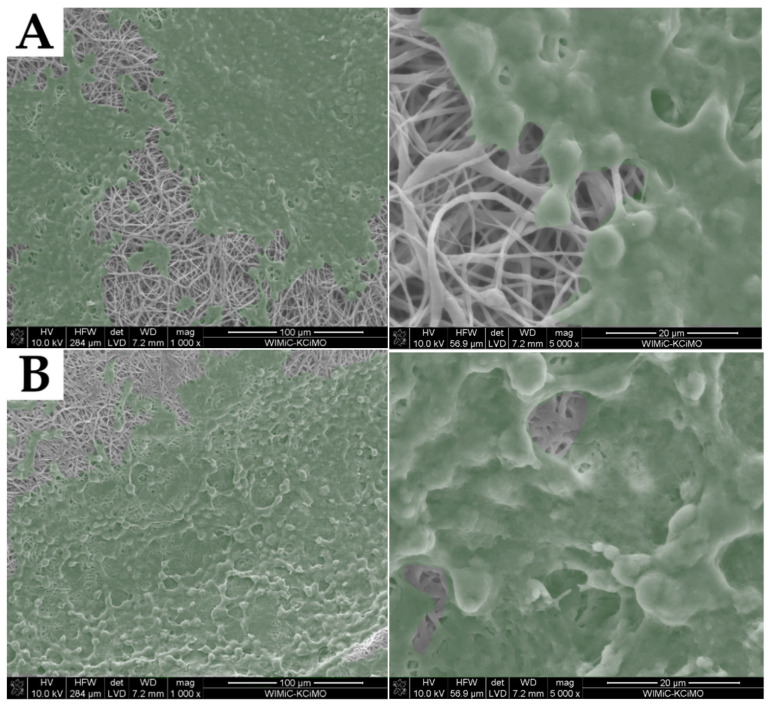

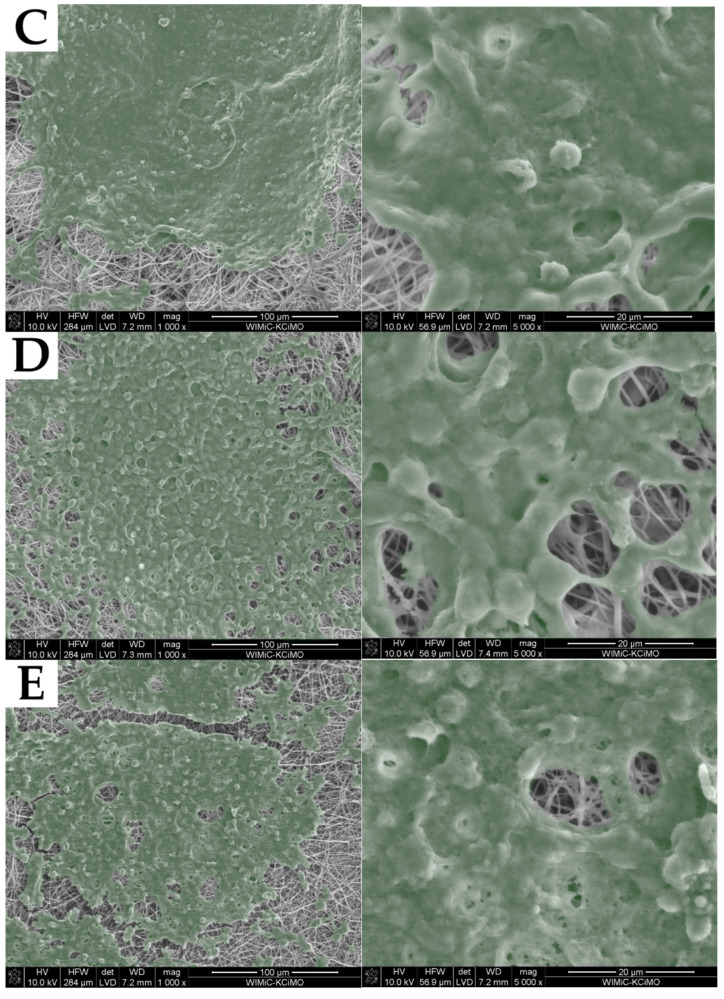
SEM images of RAW 264.7 mouse macrophages present on the surface of sample T1 (**A**), T4 (**B**), S1 (**C**), S4 (**D**), and PCL (**E**). Cells are marked in green.

Interactions of cells with nonwovens after 7 days of incubation are presented on SEM micrographs (Figure 11). Macrophages spread over the surface of each emulsion electrospun sample, regardless of the surfactant used. They had no special surface preferences but were evenly spread across the surface and formed large aggregates almost covering the surface of the fibrous mat. However, cells only flattened on the surface of the samples but did not infiltrate the material. The same effect was shown by neat PCL fibers (Figure 11E). The reason was the low porosity resulting from the small diameter of the fibers.

## 3. Discussion

Emulsion electrospun fibers are excellent carriers for vulnerable biomolecules such as proteins. Due to the presence of a surfactant, the additives are separated from the hazardous environmental influences, which allows them to be delivered to the targeted tissue without losing biological activity. In the present study, BSA was encapsulated in PCL-based core-shell fibers using the emulsion electrospinning method. In order to obtain a stable emulsion, two different types of surfactants (non-ionic and ionic) were used in two different concentrations (0.1% and 0.4%) and assessed their influence on the formulation of the emulsion as well as the properties of nonwovens. As a result, fibrous substrates were obtained differing in physicochemical properties, loading efficiency, and release character.

Overall, the results demonstrated a sequence of dependencies between the type of surfactant, the properties of the emulsion, and the properties of the nonwoven fabric. Depending on the type of surfactant, a different change in the conductance of the emulsions was obtained. The presence of an ionic head group in the SDS structure contributed to a huge increase in the conductivity of the emulsion, while the non-ionic head group of Tween 80 did not make a significant difference. The measurements of the micelle size are strongly correlated with the size of the surfactants themselves. SDS is a low molecular weight surfactant (~288 g/mol) that forms small micelles (~52 nm in DCM/DMF solution), while Tween 80 has a molecular weight of ~1310 g/mol and a micelle size of ~5500 nm. This difference in micelle size contributed to different fiber diameters and encapsulation efficiency. The larger size of the micelles obtained with Tween 80 allowed for more effective encapsulation of BSA (which was higher than that of the modified SDS) but also contributed to the increase in the diameter of the fibers modified by this surfactant. In turn, the size of the micelles affected the viscosity of the emulsions, which strongly depends on the size and concentration as well as the type of surfactant. Due to the caging effect of micelles by the oil phase, the emulsion behaves like a viscoelastic liquid. If the size of the micelle is smaller, more molecules of the continuous phase (PCL) are immobilized on their surface, contributing to the flow of micelles with the continuous phase [22,44]. However, this was observed phenomenon only in the case of the emulsion containing Tween 80. In the case of the emulsion containing SDS, the viscosity did not differ significantly between the S1 and S4 samples, which may be due to too small difference between the surfactant concentrations. In turn, the rheological behavior of the “T” samples was drastically different–the difference in viscosity depending on the surfactant concentration is clearly marked. The T4 sample exhibited significantly higher viscosity than the T1 sample, which is in line with the caging theory. This is due to the structure and concentration of the surfactant and its interaction with the polymer. Only in the case of sample S1 was the concentration of surfactant lower than the CMC value. However, the increase in viscosity proves that the CAC value was exceeded in each case. For both samples consisting of Tween 80, the concentration was many times higher than the CMC value, which allowed the formation of micelles with the formation of the dispersion phase [45]. The higher the concentration of Tween 80, the greater the number of micelles in the emulsion and the higher the solution viscosity. In the case of the S1 sample, despite the lower concentration and the lack of micelles in the solution, the aggregates formed contributed to a partial stabilization of the water phase and an increase in the viscosity of the emulsion in relation to the PCL solution. However, the stability of the emulsion one hour after preparation was reduced, while for the remaining samples, it remained the same. Wang et al. also observed significantly higher viscosity when using a non-ionic than an anionic or cationic surfactant. They attributed this to the weak interaction between the surfactant head and the polymer that “wraps” the micelles and uses it as a cross-linker. As a result, a resistant to deformation temporary network occurs, the cross-linking of which depends on the size of the micelles-the larger micelles, the better the crosslinking and thus the viscosity [46].

During the electrospinning process, the solution is subjected to both electric and shear forces [22]. Therefore, the appropriate rheological properties are an important factor that have a huge influence on the spinnability of the electrospinning precursor and hence the morphology and size of the fibers. The optimal range of viscosity for electrospun solutions is within the range of 0.1–2 Pa∙s. If its value is too low or too high, there is a risk of obtaining the beaded fibers or even electrospraying [31]. The viscosity of all the emulsions obtained right after preparation was in a range of 0.2–2.1 Pa∙s, which is appropriate for obtaining continuous fibers. One hour after preparation, the viscosities of all emulsions, besides T4, had not changed significantly. The decrease in the viscosity of the T4 sample indicates a decrease in the interfacial energy between the droplets due to, for example, coalescence or flocculation. This decrease indirectly indicates that the emulsion started to lose stability [47]. One hour after preparation, this drop in viscosity is more likely to be due to the decomposition of the micelles than to the miscibility of the water and oil phases or the volume fraction of the water phase. The solution of the solvents in the oil phase consists of non-polar and water-insoluble DCM and slightly polar and water-soluble DMF. It is well known that the polar character and water miscibility of the oil phase reduce the interfacial tension between the dispersed and continuous phase, which reduces the stability of the emulsion [48]. However, the low DMF content in relation to DCM and the small volume fraction of the aqueous phase allows for maintaining the emulsion stability of samples T1, S1, and S4, which was confirmed in rheological tests. Moreover, all of the emulsions still managed to maintain the viscosity in the range of the solution spinnability (>0.1 Pa∙s). Giannetti et al. also reported a positive effect of the ionic surfactant on the stability of the emulsion during the electrospinning process [37].

The final diameter of the fibers depends on the whipping instability, which results directly from the conductivity of the electrospinning solution [21]. Since the presence of the ionic head in the SDS structure contributed to its higher conductivity, the fibers diameter of the S1 and S4 samples were smaller than those fabricated with the use of Tween 80. The addition of the water phase to emulsions consisting of Tween 80 contributed to a slight increase in diameter size compared to PCL fibers, while the use of SDS allowed retaining the fiber diameter similar to the reference. The studies also showed the possibility of successfully incorporating BSA into fibers. Regardless of the type of surfactant, all fibers had a core-shell morphology consisting of BSA in the core part, which was confirmed by microscopic observations and XPS analysis.

Another important factor in terms of diffusional release, protein adsorption, and cellular adhesion is the wetting behavior of the substrate. All samples containing the surfactant showed a decrease in the contact angle, which changed the surface character from hydrophobic to hydrophilic. Moreover, as the concentration of the surfactant increased, the value of the contact angle decreased. This was due to the relocation of the surfactant across the surface of the material. During the formation of an emulsion, the surfactant is first located at the oil-water interface. On the other hand, by maintaining the volume of the aqueous phase constant and increasing the surfactant concentration, monomers that are unbound to micelles are located at the air-polymer surface interface [30]. A decrease in the contact angle correlated with an increase in surfactant concentration was also reported by other authors [29,37].

The location of the water phase in the fiber was reflected in the results of the release studies. The samples exhibited a similar release trend within one surfactant. For nonwovens containing Tween 80, the results showed release kinetics characterized by a zero-order model. This model, characteristic of a fibrous carrier, is characterized by a sustained (linear) release of the active biomolecule from the fibers, which is associated with the gradual erosion of the polymer shell, leading to the formation of the micropores and diffusion of the additives through these channels [49,50]. The observed release kinetics result from the complete encapsulation of the protein inside the core of the fiber, which contributed to the dependence of the protein release rate solely on the erosion rate of the polymer matrix. Since the T4 was a less stable emulsion, initial burst release occurred at the beginning of incubation, which could be attributed to the presence of a small amount of the protein at the surface of the fibers [35]. However, during this phase, only 10% of BSA was released and then the samples exhibited a stable and sustained release character. A completely different situation was with the samples containing SDS. A burst release of ~50% of the initial mass was observed at the beginning of incubation for both the S1 and S4 samples, followed by further sustained release. The obtained curve exhibits a more complex release pattern described by a three-phase profile that is characteristic of polymeric carriers with heterogeneous degradation. Phase I (described by the first-order release model) is referred to as the burst release due to the rapid wetting of the BSA that was closest to the surface of the fibers. This is followed by a rapid phase II, which has also been described by a first-order release model and is associated with the release of the drug from the exposed pores. The last phase III occurs simultaneously with polymer degradation and is characterized by a slow release of BSA from the eroding matrix or through the pores [51]. The value of n < 0.45 (characteristic for Fickian diffusion), which was obtained from the Ritger-Peppas model, indicated that diffusion was the dominant release process [52]. The dependence of the obtained release profiles on the type of surfactant may result from the different morphology of the core part of the fibers. As it was mentioned before, due to the highly hydrophilic nature of SDS, the fibers consisting of this surfactant tend to have a pseudo-core-shell morphology. Moreover, the pH of the BSA solution could also affect its distribution. Since the isoelectric point of BSA is ~4.7, the charge of the protein in distilled water is negative [53]. The presence of a charge on both the surfactant and the biomolecule may also influence the movement of micelles toward the surface [35]. This leads to an uneven distribution of albumin within the fiber, also around its surface, which results in the immediate ejection of the protein portion closest to the surface in direct contact with water [1]. Similar results were observed by Yazgan et al., who investigated the effect of humidity on the morphology of core-shell fibers. The highest burst release was observed in the case of spun fibers with the lowest humidity, characterized by subsurface deposition of the additive [29]. The inhomogeneous distribution of BSA can also be attributed to improper encapsulation of micelles inside the fibers, resulting in the aggregation of the protein underneath the surface [37].

The in vitro tests are compatible with the release of BSA to the surrounding medium. In the case of the S1 and S4 samples, the burst release in the initial stage of incubation contributed to its high concentration in the cell environment from the very beginning of the culture, which also led to its positive effect on cell viability. The lower cell viability on the substrates modified with Tween 80 may be caused by the lower protein loading efficiency in the fibers. However, these samples also showed greater sustained release kinetics over time, releasing only 20% of BSA on the 7th day of the cell culture study, while 70% was released for the samples modified with SDS. Since bovine serum albumin acts as an attractor to cells, the viability results for S1 and S4 were better. Therefore, a longer culture time is required to fully assess the overall biocompatibility of samples T1 and T4. This suggests that when a quick response of the organism to the scaffold (e.g., cell adhesion) is required, SDS is the better choice for the preparation of an emulsion. On the other hand, Tween 80 provides the zero-order release character desired for sustained, prolonged release (e.g., anti-inflammatory agents). Moreover, all samples exhibited lower cytotoxicity than the neat PCL fibers (Ref sample) as well as the control (TCPS), which indicates their excellent biocompatibility. Macrophages colonized the entire surface of the material, and moreover, the elongated phenotype indicated good adhesion of cells to the scaffold. This was due to the nanometric size of the fibers, which allowed for the creation of a multi-point connection with the substrate and increased the flexibility of the nonwoven fabric. Other authors also reported good cell viability on the emulsion electrospun scaffolds. Akbarzadeh et al. obtained PCL/PVA-GEL core/shell nanofibers, which induced adhesion of the L929 fibroblasts. Biological studies showed high cell viability (above 80%) after 24 h and 48 h incubation, spindle-like morphology, and no signs of damage (cells debris) [47]. Basar et al., who also conducted research on the same type of cells, reported a positive effect of the emulsion electrospun PCL/gelatin fibers containing ketoprofen on the viability ant phenotype of the L929 fibroblasts [42]. Johnson et al. also obtained a good cellular response (human meniscal fibrochondrocyte–MFCs) to emulsion electrospun scaffolds containing Span 80. All MFCs adhered, spread over the surface of the nonwoven and showed the spindle-like phenotype [30].

## 4. Materials and Methods

Polycaprolactone (PCL) pellets (MW = 80kDa), Tween 80 for synthesis, Bovine Serum Albumin (BSA, MW = 66 kDa, SKU: A4503—purity of the material checked by SDS-PAGE—see supporting information S1 and S2), and glutaraldehyde (25%) were purchased from Sigma-Aldrich (UK, London, Merck). Dichloromethane (DCM) was purchased from Chemland SA (Poland, Stargard Szczeciński). Dimethylformamide (DMF) was purchased from SupraSolv (Germany, Supelco, Merck). Sodium Dodecyl Sulphate (SDS) and Fluorescein were purchased from POCH (Poland, Gliwice, Avante Performance Materials S.A.). Ethanol was purchased from STANLAB (Poland, Lublin). Murine macrophages RAW 264.7 (ATCC TIB–71) were purchased from American Type Culture Collection (ATCC; Manassas, VA, USA). ViaLight^®^Plus and ToxiLight^®^Plus tests for biological studies were purchased from Lonza (BioAssay Kit, Lonza, Walkersville, MD, USA). Exton reagent was purchased from Analab (Poland, Warsaw).

### 4.1. Fabrication of Nonwovens

#### 4.1.1. Preparation of the Emulsion

For the production of core-shell nonwovens, the emulsion electrospinning method was used, in which a water-in-oil emulsion was prepared as an electrospinning solution by dissolving a BSA aqueous solution (2.5% by weight of dry PCL) in 5 mL of PCL solution with the addition of a surfactant. The water phase was a 5% (*w*/*v*) solution of BSA in distilled water. In order to obtain the oil phase, a 10% (*w*/*v*) solution of PCL with DCM:DMF in a ratio of 7:3 (*v*/*v*) was prepared. Two types of surfactants were used as emulsifiers: Tween 80 (non-ionic) and SDS (anionic) in two different concentrations: 0.1% and 0.4% (*v*/*v*) for Tween 80 and 0.1% and 0.4% (*w*/*v*) for SDS (Table 4). Briefly, 0.5 g of PCL was dissolved in 5 mL of a DCM:DMF solution. 5 g and 20 g of SDS were added to polymer solution, and then 250 µL of the BSA solution (consisting of 12.50 mg of protein) was added dropwise to the polymer/surfactant phase and stirred in an ice bath. The same procedure was used for the samples with Tween 80. The volume fraction of the water phase was 5%. The selection of surfactant concentration was based on the literature data concerning safe doses of surfactant [54].

#### 4.1.2. Electrospinning Process

**Figure 12:**
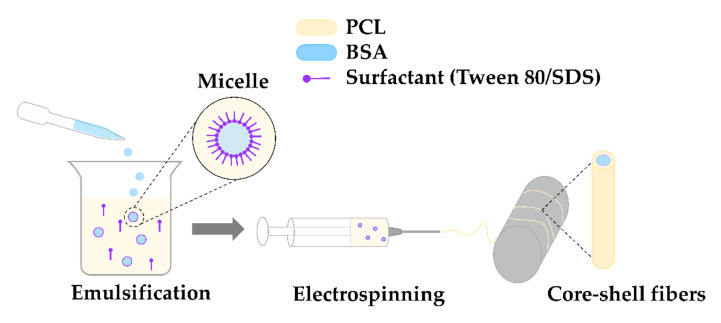
Schema of the electrospinning process.

The electrospinning process was carried out at ambient temperature (~23 °C) and relative humidity in the range of 35–45%. The emulsion was supplied through a 19 G needle at a feeding rate of 1 mL/h and voltage of 14 kV. The fibers were collected on a drum collector with the spinneret-to-collector distance remaining at 10 cm. The method of preparing nonwovens is illustrated in Figure 12.

### 4.2. Methods

#### 4.2.1. Characterization of the Electrospinning Solution

A conductivity meter (Elmetron CC-401, Poland) was used to measure the conductivity of an emulsion without PCL at room temperature (~23 °C). The results presented in the paper are the average of three measurements.

A rheological test (viscosity measurements) was conducted on a Physica MCR-301 rheometer (Anton Paar, Austria, Graz) equipped with a PP25 parallel plate. The gap between plates was 0.2 mm and the shear rate was constant (γ = 1001/s). Measurements were carried out at room temperature (~23 °C), and the results presented in the paper are the average of three measurements.

To determine the presence and size distribution of the dispersed phase, a VHX-6000 digital microscope (Keyence, Japan, Osaka) was used. The emulsions were placed between the microscope slides and immediately observed in the bright field mode. The diameter of 100 micelles for each sample was measured using ImageJ software (Fiji ver. 1.53f, October 2020). On this basis, the micelles size distribution was obtained.

#### 4.2.2. Fibers Morphology

Scanning electron microscopy (SEM) was used to investigate the microstructure of nonwovens. Prior to the observations, the samples were coated with a 10 nm gold layer using a Leica EM ACE600 rotary pump sputter coater (Wetzaler, Germany). Observations were conducted using a NOVA NANO SEM 200 Microscope (FEI, Hillsboro, OR, USA). Using SEM micrographs, the diameter of 100 fibers was measured with ImageJ software (Fiji ver. 1.53f, October 2020) and the fiber size distribution was obtained on this basis.

To study the morphology of the core-shell emulsion electrospun fibers, confirming the effectiveness of PCL fibers modification with micelles, 0.1% (*w*/*v*) fluorescein was added to the BSA solution and observed under a fluorescence microscope (Axiovert 40, Zeiss, Oberkochen, Germany).

#### 4.2.3. Structural Analysis

The surface chemical composition was evaluated for all types of polymer nonwovens: reference (Ref) and emulsion electrospun (T1, T4, S1, S4) via XPS (Vacuum Systems Workshop, Ltd., England, East Grinstead) using Mg Ka X-ray radiation with an energy of 200 W, with electron energy analyzer set to FAT mode and pass energy of 22 eV. The analysis depth was set experimentally to ~5 nm and the spatial resolution to ~3 mm. Therefore, a single measurement was taken as representative of the entire sample area. The spectra were calibrated by assuming the binding energy of C1s is always 284.6 eV. The XPS 4.1 software was used to perform the spectral analysis.

#### 4.2.4. Wettability

To determine the wettability of the nonwovens, the water contact angle was measured using a DSA 25 goniometer (Kruss, Germany, Hamburg). The sessile drop test was carried out at room temperature (~23 °C) by placing a drop (1 µL) of deionized water on the fibrous mat surface. Results are presented as the average of 10 measurements for each sample with the standard deviation.

#### 4.2.5. Efficiency of Encapsulation and Release Studies

To determine the loading efficiency (LE) and encapsulation efficiency (EE), BSA was replaced with fluorescein as a model drug. To measure the amount of fluorescein in the fibers, 4 mg of the samples was dissolved in 1 mL of acetic acid and we placed the 200 µL aliquots in a black 96-well polystyrene plate for fluorescence measurement (λ_ex_ = 494 nm, λ_em_ = 521 nm, FluoStar Omega, BMG Labtech, Germany, Ortenberg). To calculate concentration of the dye, standard curves of the known concentration of the fluorescein in acetic acid (0.2 mg/mL, 0.4 mg/mL, 0.6 mg/mL, 0.8 mg/mL, 1 mg/mL) were used. To eliminate the influence of a potential interaction between fluorescein and PCL or Tween 80/SDS on the results, the fluorescence of neat PCL fibers and PCL with added surfactants was checked. Both theoretical and experimental loading efficiency, as well as encapsulation efficiency, were calculated from the equations (Equations (1) and (2)):

(1) $$\%\mathrm{LE}=\frac{\mathrm{mass}\;\mathrm{of}\;\mathrm{BSA}\;\mathrm{in}\;\mathrm{fibers}}{\mathrm{mass}\;\mathrm{of}\;\mathrm{fibers}}*100\%$$

(2) $$\%\mathrm{EE}=\frac{\mathrm{mass}\;\mathrm{of}\;\mathrm{BSA}\;\mathrm{in}\;\mathrm{fibers}}{\mathrm{mass}\;\mathrm{of}\;\mathrm{BSA}\;\mathrm{initialy}\;\mathrm{added}\;\mathrm{to}\;\mathrm{the}\;\mathrm{spinning}\;\mathrm{solution}}*100\%$$

To determine the amount of released BSA over time, the prepared samples were incubated in 0.15 M NaCl solution for 30 days. To normalize the differences in thickness, all the samples were weighed before testing, cut to the same weight (~40 mg), and placed in 3 mL of NaCl solution. Every day, a full 3 mL of NaCl solution were taken for measurement and replaced with the same amount of fresh solution. To determine the concentration of the released BSA, 1 mL of Exton reagent was added to 1 mL of the aliquots, and after a 10 min wait to obtain a turbid solution, was placed in the spectrophotometer chamber (UV–Vis, Cecil 2520). The measurement was carried out at a wavelength of λ = 445 nm and calculated the BSA concentration using standard curves of the known concentration of BSA in NaCl (0.2 mg/mL, 0.4 mg/mL, 0.6 mg/mL, 0.8 mg/mL, 1 mg/mL).

#### 4.2.6. Mathematical Modeling of BSA Release

To evaluate the mechanism of BSA release from the prepared samples, the experimental data was compared to four established models: zero-order, first-order, Higuchi, and Ritger-Peppas [55], according to the equations:

- Zero-order
  (3) $$\frac{M_{n}}{M_{\infty }}=\mathrm{Kt}$$
- First-order
  (4) $$\frac{M_{n}}{M_{\infty }}=1-e^{-\mathrm{Kt}}$$
- Higuchi
  (5) $$\frac{M_{n}}{M_{\infty }}={\mathrm{Kt}}^{0.5}$$
- Ritger-Peppas
  (6) $$\frac{M_{n}}{M_{\infty }}={\mathrm{Kt}}^{n}$$

M_n_—cumulative amount of the drug released over time t,
M_∞_—initial amount of the drug,
K, n—constants.

To determine the best fit for the mathematical models, the Microsoft Excel 365 with the Solver and Data Analyzer add-in package was used. The best fit of the model was achieved by optimizing the K value, which allowed to minimize the mean square error on the basis of comparing the calculated profiles with the experimental data using R^2^ value.

#### 4.2.7. Biological Studies

Before testing, the samples were cut into 16 mm discs and placed in 24-well plates (Nest Scientific Biotechnology, Wuxi, China). Four samples were prepared for the viability test and four for the cytotoxicity test after 3 and 7 days (in total, 16 samples per variant) and sterilized with UV irradiation for 15 min on each side. RAW 264.7 (ATCC TIB-71) mouse macrophages were used for biological studies. Before testing, the cells were cultured in 75 mL plastic bottles (Nest SB, New Jersey, USA) containing DMEM cell culture medium (Lonza, Walkersville, MD, USA) enriched with L-glutamine, glucose, 10% fetal bovine serum (Gibco, Waltham, MA, USA), and 5% penicillin and streptomycin solution (Sigma-Aldrich, Germany, Schnelldorf). The cells were stored in an incubator (MCO-18AC PhCbi, UK, Loughborough) under conditions of 5% CO_2_ and 37 °C until they formed a confluent layer, and then they were passaged five times by scrapping. The cells were then diluted to obtain 10,000 cells/mL (a Bürker’s hemocytometer was used to count the cells) and added to the plates containing the samples. As control material (ctr), tissue culture polystyrene discs (TCPS) (Menzel Glaser, Germany, Menzel Glaser) were used with neat PCL fibers as the reference sample (Ref). The cell viability was determined using the Vialight^TM^ assay and cytotoxicity using the Toxilight^TM^ assay. Both tests were conducted according the manufacturer’s protocols [56,57]. Then, the plates were placed in a luminometer (FluoStar Omega, BMG Labtech, Germany, Ortenberg).

In order to assess the morphology of cells, after the 7th day of contact with fibrous materials, they were fixed with 2.5% glutaraldehyde and dehydrated them with a series of ethanol solutions (50, 60, 70, 80, and 96%). Then, the samples were attached to the SEM holders using carbon tape and coated them with a 10 nm gold layer (Leica EM ACE600, Wetzaler, Germany). Observations were conducted using a NOVA NANO SEM 200 Microscope (FEI, Hillsboro, OR, USA).

#### 4.2.8. Statistical Analysis

Each experiment during the study was conducted in triplicate, unless otherwise specified, and presented as mean ± standard deviation (SD). The significance level for biological studies was determined using a one-way analysis of variance (ANOVA) followed by Tukey’s post hoc analysis (Origin Pro 2021 software). Probability values lower than 0.05 were considered statistically significant.

## 5. Conclusions

In our research, the influence of emulsion preparation on the encapsulation efficiency, release rate, and cellular response of PCL electrospun fibers was studied. The work focused on investigating the effect of the addition of surfactants of different molecular weights and polarity on the applicability of the substrate in the context of its use in the human body. The obtained results allowed us to broaden the knowledge about the influence of the emulsion preparation on the kinetics of drug release, which directly translates into the biological performance of the implant. It was shown that different types and concentrations of surfactants primarily affect the properties of the emulsion, which in turn affect the physicochemical properties, release kinetics, and biological performance of the nonwoven substrate. The type of surfactant has a great influence on the conductivity of the emulsion, which in turn, affects the micelle size, viscosity, and eventually the fiber microstructure and morphology of the core part. The influence of the surfactant concentration on the wettability is also worth mentioning. Overall, SDS contributed to obtaining smaller fibers with optimal contact angle and encapsulation efficiency resulting in higher viability. However, in the case of these samples, a burst release phenomenon was observed, probably due to the presence of BSA near the surface of the fiber. On the other hand, the use of Tween 80 contributed to a slightly larger fiber diameter and lower contact angle but also exhibited a sustained release of BSA. However, biological studies have revealed that the cells only spread over the surface of the scaffold and do not migrate inside because the pores are too small. In addition, the current research investigates two types of only hydrophilic surfactants–anionic and non-ionic, therefore, the effect of hydrophilicity and charge on release kinetics and proliferation require further research. Moreover, the combination of two different types of fibers with different release patterns during emulsion co-electrospinning appears to be a promising approach worth investigating and exploring.

## Figures and Tables

**Table 1:** Conductance of emulsions and mean diameter of micelles.

Sample	Conductance (µS/cm)	Mean Diameter of Micelle (µm)
Ref *	0.3 ± 0.02	-
T1	1.9 ± 0.02	13.68 ± 5.97
T4	5.4 ± 0.07	11.72 ± 7.13
S1	140.5 ± 0.04	8.80 ± 4.11
S4	183 ± 0.06	7.91 ± 3.95

* The sample without addition of the surfactant.

**Table 2:** Loading and encapsulation efficiency of BSA in samples.

Sample	Loading Efficiency (%)		Encapsulation Efficiency (%)
	Theoretical	Experimental	
T1	9.38	8.38	89.36
T4	9.38	8.57	91.39
S1	18.75	14.51	77.37
S4	18.75	16.38	87.36

**Table 3:** Kinetic parameters of BSA release from the fibers.

Model	T1		T4		S1		S4	
	K	R^2^	K	R^2^	K	R^2^	K	R^2^
Zero-order	0.038	0.999	0.040	0.993	0.056	0.749	0.057	0.682
First-order	0.056	0.971	0.056	0.965	0.174	0.837	0.215	0.815
Higuchi	0.141	0.930	0.149	0.934	0.226	0.874	0.231	0.835
Ritger–Peppas	0.036	0.999	0.058	0.987	0.293	0.719	0.301	0.774

**Table 4:** Samples description.

Sample	Description
Ref	Reference (neat PCL fibers).
T1	PCL/BSA fibers with addition of 0.1% (*v*/*v*) Tween 80.
T4	PCL/BSA fibers with addition of 0.4% (*v*/*v*) Tween 80.
S1	PCL/BSA fibers with addition of 0.1% (*w*/*v*) SDS.
S4	PCL/BSA fibers with addition of 0.4% (*w*/*v*) SDS.

## Data Availability

Not applicable.

## Supplementary Materials

The following supporting information can be downloaded at: https://www.mdpi.com/article/10.3390/molecules27103232/s1, S1: Pro-tein View: ALBU_BOVIN; S2: Peptide Summary Report.

## Sample Availability

Not available.
