# Supplementary material for: Effect of Ionic and Non-Ionic Surfactant on Bovine Serum Albumin Encapsulation and Biological Properties of Emulsion-Electrospun Fibers
Source: Molecules. 2022 May 18;27(10):3232. doi: 10.3390/molecules27103232 (PMC9143061; doi:10.3390/molecules27103232)

MASCOT Search Results

Protein View: ALBU_BOVIN

Albumin OS=Bos taurus OX=9913 GN=ALB PE=1 SV=4

**Database:**	SwissProt
**Score:**	20958
**Monoisotopic mass (M_r):**	71244
**Calculated pI:**	5.82
**Taxonomy:**	Bos taurus

Sequence similarity is available as an NCBI BLAST search of ALBU_BOVIN against nr.

Search parameters

**MS data file:**	`BSA.mgf`
**Enzyme:**	Trypsin: cuts C-term side of KR unless next residue is P.
**Fixed modifications:**	Carbamidomethyl (C)
**Variable modifications:**	Oxidation (M)

Protein sequence coverage: 72%

Matched peptides shown in ***bold red***.

1 MKWVTFISLL LLFSSAYSRG VFRRDTHKSE IAHRFK**DLGE EHFKGLVLIA**
51 FSQYLQQCPF DEHVKLVNEL TEFAKTCVAD ESHAGCEKSL HTLFGDELCK
101 VASLRETYGD MADCCEKQEP ER**NECFLSHK DDSPDLPKLK PDPNTLCDEF**
151 KADEKKFWGK YLYEIAR**RHP YFYAPELLYY ANKYNGVFQE CCQAEDKGAC**
201 LLPKIETMRE KVLASSARQR LRCASIQKFG ER**ALKAWSVA R**LSQK**FPKAE**
251 FVEVTKLVTD LTKVHKECCH GDLLECADDR ADLAKYICDN QDTISSKLKE
301 CCDKPLLEKS HCIAEVEKDA IPENLPPLTA DFAEDKDVCK **NYQEAKDAFL**
351 GSFLYEYSRR HPEYAVSVLL RLAKEYEATL EECCAKDDPH ACYSTVFDKL
401 K**HLVDEPQNL IKQNCDQFEK LGEYGFQNAL IVR**YTR**KVPQ VSTPTLVEVS**
451 RSLGKVGTR**C CTKPESERMP CTEDYLSLIL NRLCVLHEKT PVSEK**VTKCC
501 TESLVNR**RPC FSALTPDETY VPKAFDEKLF TFHADICTLP DTEKQIKKQT**
551 ALVELLKHKP **KATEEQLKTV MENFVAFVDK CCAADDKEAC FAVEGPK**LVV
601 STQTALA

Unformatted sequence string: 607 residues (for pasting into other applications).

Sort by ◉ residue number ○ increasing mass ○ decreasing mass
Show ◉ matched peptides only ○ predicted peptides also

Query	Start – End	Observed	Mr(expt)	Mr(calc)	Delta	M	Score	Expect	Rank	U	Peptide
☐7305	37 – 65	862.9253	3447.6721	3446.6969	0.9752	1	47	0.017	1 Score > **42** indicates **identity** Score > **39** indicates **homology**	U	K.DLGEEHFKGLVLIAFSQYLQQCPFDEHVK.L
☐5944	45 – 65	623.8186	2491.2453	2491.2570	-0.0117	0	56	0.0035	1 Score > **44** indicates **identity** Score > **35** indicates **homology**	U	K.GLVLIAFSQYLQQCPFDEHVK.L
☐5946	45 – 65	624.0688	2492.2461	2491.2570	0.9891	0	56	0.0036	1 Score > **44** indicates **identity** Score > **28** indicates **homology**	U	K.GLVLIAFSQYLQQCPFDEHVK.L
☐5947	45 – 65	1247.1307	2492.2468	2491.2570	0.9899	0	89	2e-06	1 Score > **44** indicates **identity**	U	K.GLVLIAFSQYLQQCPFDEHVK.L

									Score > **25** indicates **homology**		
5948	45 – 65	831.7567	2492.2483	2491.2570	0.9913	0	65	0.00043	1Score > **44** indicates **identity** Score > **24** indicates **homology**	U	K.GLVLIAFSQYLQQCPFDEHVK.L
5949	45 – 65	624.0695	2492.2489	2491.2570	0.9919	0	57	0.0027	1Score > **44** indicates **identity** Score > **28** indicates **homology**	U	K.GLVLIAFSQYLQQCPFDEHVK.L
5950	45 – 65	624.0696	2492.2493	2491.2570	0.9923	0	51	0.012	1Score > **44** indicates **identity** Score > **29** indicates **homology**	U	K.GLVLIAFSQYLQQCPFDEHVK.L
5951	45 – 65	831.7573	2492.2501	2491.2570	0.9931	0	61	0.0012	1Score > **44** indicates **identity** Score > **23** indicates **homology**	U	K.GLVLIAFSQYLQQCPFDEHVK.L
5952	45 – 65	831.7574	2492.2504	2491.2570	0.9934	0	73	7.2e-05	1Score > **44** indicates **identity** Score > **23** indicates **homology**	U	K.GLVLIAFSQYLQQCPFDEHVK.L
5953	45 – 65	624.0699	2492.2505	2491.2570	0.9935	0	51	0.012	1Score > **44** indicates **identity** Score > **22** indicates **homology**	U	K.GLVLIAFSQYLQQCPFDEHVK.L
5954	45 – 65	831.7579	2492.2519	2491.2570	0.9949	0	46	0.036	1Score > **44** indicates **identity** Score > **24** indicates **homology**	U	K.GLVLIAFSQYLQQCPFDEHVK.L
5955	45 – 65	831.7580	2492.2522	2491.2570	0.9952	0	48	0.021	1Score > **44** indicates **identity** Score > **25** indicates **homology**	U	K.GLVLIAFSQYLQQCPFDEHVK.L
5957	45 – 65	831.7582	2492.2528	2491.2570	0.9958	0	56	0.0034	1Score > **44** indicates **identity** Score > **23** indicates **homology**	U	K.GLVLIAFSQYLQQCPFDEHVK.L
5958	45 – 65	831.7584	2492.2534	2491.2570	0.9964	0	62	0.00097	1Score > **44** indicates **identity** Score >	U	K.GLVLIAFSQYLQQCPFDEHVK.L

											**25** indicates **homology**		
□5959	45	–	65	831.7586	2492.2540	2491.2570	0.9970	0	51	0.01	1Score **> 44** indicates **identity** Score **> 25** indicates **homology**	U	K.GLVLIAFSQYLQQCPFDEHVK.L
□5960	45	–	65	831.7586	2492.2540	2491.2570	0.9970	0	53	0.0069	1Score **> 44** indicates **identity** Score **> 26** indicates **homology**	U	K.GLVLIAFSQYLQQCPFDEHVK.L
□5961	45	–	65	1247.1344	2492.2542	2491.2570	0.9973	0	63	0.00069	1Score **> 44** indicates **identity** Score **> 23** indicates **homology**	U	K.GLVLIAFSQYLQQCPFDEHVK.L
□7496	45	–	75	910.2211	3636.8553	3635.8698	0.9855	1	58	0.00099	1Score **> 41** indicates **identity** Score **> 26** indicates **homology**	U	K.GLVLIAFSQYLQQCPFDEHVKLVNELTEFAK.T
□7497	45	–	75	910.2212	3636.8557	3635.8698	0.9859	1	79	9.1e-06	1Score **> 41** indicates **identity** Score **> 25** indicates **homology**	U	K.GLVLIAFSQYLQQCPFDEHVKLVNELTEFAK.T
□7498	45	–	75	910.2215	3636.8569	3635.8698	0.9871	1	68	0.0001	1Score **> 41** indicates **identity** Score **> 33** indicates **homology**	U	K.GLVLIAFSQYLQQCPFDEHVKLVNELTEFAK.T
□7499	45	–	75	910.2228	3636.8621	3635.8698	0.9923	1	64	0.00027	1Score **> 41** indicates **identity** Score **> 25** indicates **homology**	U	K.GLVLIAFSQYLQQCPFDEHVKLVNELTEFAK.T
□7500	45	–	75	910.2228	3636.8621	3635.8698	0.9923	1	73	3.6e-05	1Score **> 41** indicates **identity** Score **> 25** indicates **homology**	U	K.GLVLIAFSQYLQQCPFDEHVKLVNELTEFAK.T
□7501	45	–	75	1213.2960	3636.8662	3635.8698	0.9964	1	67	0.00013	1Score **> 41** indicates **identity** Score **> 26** indicates **homology**	U	K.GLVLIAFSQYLQQCPFDEHVKLVNELTEFAK.T
□3165	66	–	75	582.3158	1162.6170	1162.6234	-0.0063	0	56	0.0074	1Score **> 47** indicates **identity** Score **> 32**	U	K.LVNELTEFAK.T

										indicates **homology**	
☐3166	66	– 75	582.3160	1162.6174	1162.6234	-0.0059	0	52	0.019	1Score **>** **47** indicates **identity** Score **>** **32** indicates **homology**	U K.LVNELTEFAK.T
☐3167	66	– 75	582.3160	1162.6174	1162.6234	-0.0059	0	57	0.0067	1Score **>** **47** indicates **identity** Score **>** **33** indicates **homology**	U K.LVNELTEFAK.T
☐3170	66	– 75	582.3162	1162.6178	1162.6234	-0.0055	0	56	0.0072	1Score **>** **47** indicates **identity** Score **>** **32** indicates **homology**	U K.LVNELTEFAK.T
☐3175	66	– 75	582.3163	1162.6180	1162.6234	-0.0053	0	50	0.034	1Score **>** **47** indicates **identity** Score **>** **32** indicates **homology**	U K.LVNELTEFAK.T
☐3176	66	– 75	1163.6254	1162.6181	1162.6234	-0.0052	0	54	0.011	1Score **>** **47** indicates **identity** Score **>** **37** indicates **homology**	U K.LVNELTEFAK.T
☐3180	66	– 75	582.3165	1162.6184	1162.6234	-0.0049	0	56	0.007	1Score **>** **47** indicates **identity** Score **>** **32** indicates **homology**	U K.LVNELTEFAK.T
☐3181	66	– 75	582.3166	1162.6186	1162.6234	-0.0047	0	49	0.041	1Score **>** **47** indicates **identity** Score **>** **32** indicates **homology**	U K.LVNELTEFAK.T
☐3182	66	– 75	582.3167	1162.6188	1162.6234	-0.0045	0	54	0.012	1Score **>** **47** indicates **identity** Score **>** **33** indicates **homology**	U K.LVNELTEFAK.T
☐3185	66	– 75	582.3168	1162.6190	1162.6234	-0.0043	0	49	0.038	1Score **>** **47** indicates **identity** Score **>** **34** indicates **homology**	U K.LVNELTEFAK.T
☐3186	66	– 75	582.3168	1162.6190	1162.6234	-0.0043	0	48	0.044	1Score **>** **47** indicates **identity** Score **>** **32** indicates	U K.LVNELTEFAK.T

											**homology**		
□3189	66	– 75	582.3169	1162.6192	1162.6234	-0.0041	0	57	0.0068	1	Score > **47** indicates **identity** Score > **33** indicates **homology**	U	K.LVNELTEFAK.T
□3192	66	– 75	582.3171	1162.6196	1162.6234	-0.0037	0	53	0.017	1	Score > **47** indicates **identity** Score > **31** indicates **homology**	U	K.LVNELTEFAK.T
□3194	66	– 75	582.3174	1162.6202	1162.6234	-0.0031	0	53	0.017	1	Score > **47** indicates **identity** Score > **31** indicates **homology**	U	K.LVNELTEFAK.T
□6224	66	– 88	870.4022	2608.1848	2607.1945	0.9903	1	96	2.9e-07	1	Score > **43** indicates **identity** Score > **24** indicates **homology**	U	K.LVNELTEFAKTCVADESHAGCEK.S
□6225	66	– 88	653.0535	2608.1849	2607.1945	0.9904	1	95	3.8e-07	1	Score > **43** indicates **identity** Score > **31** indicates **homology**	U	K.LVNELTEFAKTCVADESHAGCEK.S
□6690	76	– 100	955.7581	2864.2525	2863.2575	0.9949	1	76	2.7e-05	1	Score > **43** indicates **identity** Score > **27** indicates **homology**	U	K.TCVADESHAGCEKSLHTLFGDELCK.V
□3759	89	– 100	710.0826	1418.1506	1418.6864	-0.5358	0	49	0.021	1	Score > **45** indicates **identity** Score > **22** indicates **homology**		K.SLHTLFGDELCK.V
□3769	89	– 100	710.3472	1418.6798	1418.6864	-0.0066	0	54	0.011	1	Score > **47** indicates **identity** Score > **26** indicates **homology**		K.SLHTLFGDELCK.V
□3773	89	– 100	710.3474	1418.6802	1418.6864	-0.0062	0	52	0.017	1	Score > **47** indicates **identity** Score > **26** indicates **homology**		K.SLHTLFGDELCK.V
□3774	89	– 100	710.3474	1418.6802	1418.6864	-0.0062	0	59	0.0032	1	Score > **47** indicates **identity** Score > **32** indicates **homology**		K.SLHTLFGDELCK.V

3775	89	–	100	710.3474	1418.6802	1418.6864	-0.0062	0	55	0.0077	1	Score > **47** indicates **identity** Score > **35** indicates **homology**	K.SLHTLFGDELCK.V
3780	89	–	100	710.3475	1418.6804	1418.6864	-0.0060	0	59	0.0035	1	Score > **47** indicates **identity** Score > **32** indicates **homology**	K.SLHTLFGDELCK.V
3781	89	–	100	710.3475	1418.6804	1418.6864	-0.0060	0	57	0.0052	1	Score > **47** indicates **identity** Score > **36** indicates **homology**	K.SLHTLFGDELCK.V
3785	89	–	100	710.3478	1418.6810	1418.6864	-0.0054	0	56	0.0064	1	Score > **47** indicates **identity** Score > **36** indicates **homology**	K.SLHTLFGDELCK.V
3789	89	–	100	1419.6888	1418.6815	1418.6864	-0.0049	0	66	0.00072	1	Score > **47** indicates **identity** Score > **35** indicates **homology**	K.SLHTLFGDELCK.V
3790	89	–	100	1419.6888	1418.6815	1418.6864	-0.0049	0	64	0.00096	1	Score > **47** indicates **identity** Score > **44** indicates **homology**	K.SLHTLFGDELCK.V
3791	89	–	100	710.3481	1418.6816	1418.6864	-0.0048	0	58	0.0046	1	Score > **47** indicates **identity** Score > **36** indicates **homology**	K.SLHTLFGDELCK.V
3796	89	–	100	710.3484	1418.6822	1418.6864	-0.0042	0	54	0.011	1	Score > **47** indicates **identity** Score > **35** indicates **homology**	K.SLHTLFGDELCK.V
3798	89	–	100	710.3485	1418.6824	1418.6864	-0.0040	0	57	0.0051	1	Score > **47** indicates **identity** Score > **28** indicates **homology**	K.SLHTLFGDELCK.V
3799	89	–	100	710.3489	1418.6832	1418.6864	-0.0032	0	64	0.00094	1	Score > **47** indicates **identity** Score > **36** indicates **homology**	K.SLHTLFGDELCK.V
											1	Score >	

3806	89	–	100	710.8486	1419.6826	1418.6864	0.9962	0	64	0.00096	**47** indicates **identity** Score > **26** indicates **homology**		K.SLHTLFGDELCK.V
3808	89	–	100	710.8488	1419.6830	1418.6864	0.9966	0	50	0.028	1Score > **47** indicates **identity** Score > **29** indicates **homology**		K.SLHTLFGDELCK.V
3809	89	–	100	710.8488	1419.6830	1418.6864	0.9966	0	52	0.016	1Score > **47** indicates **identity** Score > **34** indicates **homology**		K.SLHTLFGDELCK.V
3813	89	–	100	710.8495	1419.6844	1418.6864	0.9980	0	50	0.025	1Score > **47** indicates **identity** Score > **34** indicates **homology**		K.SLHTLFGDELCK.V
5271	89	–	105	973.5074	1945.0002	1945.0091	-0.0089	1	92	1.2e-06	1Score > **45** indicates **identity** Score > **41** indicates **homology**	U	K.SLHTLFGDELCKVASLR.E
5272	89	–	105	649.3412	1945.0018	1945.0091	-0.0074	1	87	3.8e-06	1Score > **45** indicates **identity** Score > **40** indicates **homology**	U	K.SLHTLFGDELCKVASLR.E
5273	89	–	105	649.3415	1945.0027	1945.0091	-0.0065	1	104	8.1e-08	1Score > **45** indicates **identity** Score > **42** indicates **homology**	U	K.SLHTLFGDELCKVASLR.E
5274	89	–	105	973.5129	1945.0112	1945.0091	0.0021	1	94	8.3e-07	1Score > **45** indicates **identity** Score > **43** indicates **homology**	U	K.SLHTLFGDELCKVASLR.E
5278	89	–	105	487.5081	1946.0033	1945.0091	0.9942	1	53	0.0095	1Score > **45** indicates **identity** Score > **33** indicates **homology**	U	K.SLHTLFGDELCKVASLR.E
5279	89	–	105	487.5083	1946.0041	1945.0091	0.9950	1	67	0.00037	1Score > **45** indicates **identity** Score > **35** indicates **homology**	U	K.SLHTLFGDELCKVASLR.E
											1Score > **45**		

□5280	89	–	105	649.6754	1946.0044	1945.0091	0.9952	1	90	1.9e-06	indicates **identity** Score > **41** indicates **homology**	U	K.SLHTLFGDELCKVASLR.E
□5281	89	–	105	487.5087	1946.0057	1945.0091	0.9966	1	63	0.001	1Score > **45** indicates **identity** Score > **33** indicates **homology**	U	K.SLHTLFGDELCKVASLR.E
□5282	89	–	105	487.5091	1946.0073	1945.0091	0.9982	1	49	0.024	1Score > **45** indicates **identity** Score > **28** indicates **homology**	U	K.SLHTLFGDELCKVASLR.E
□5353	101	–	117	1003.4213	2004.8280	2003.8387	0.9894	1	74	6.4e-05	1Score > **45** indicates **identity** Score > **25** indicates **homology**	U	K.VASLRETYGDMADCCEK.Q
□5206	123	–	138	634.6254	1900.8544	1900.8625	-0.0081	1	48	0.028	1Score > **45** indicates **identity** Score > **38** indicates **homology**	U	R.NECFLSHKDDSPDLPK.L
□4453	139	–	151	788.8835	1575.7524	1575.7603	-0.0078	0	61	0.002	1Score > **46** indicates **identity**	U	K.LKPDPNTLCDEFK.A
□4454	139	–	151	788.8835	1575.7524	1575.7603	-0.0078	0	82	1.6e-05	1Score > **46** indicates **identity**	U	K.LKPDPNTLCDEFK.A
□4459	139	–	151	526.5929	1576.7569	1575.7603	0.9966	0	49	0.031	1Score > **46** indicates **identity** Score > **32** indicates **homology**	U	K.LKPDPNTLCDEFK.A
□5373	139	–	155	1010.4843	2018.9540	2018.9619	-0.0078	1	99	2.3e-07	1Score > **45** indicates **identity** Score > **32** indicates **homology**	U	K.LKPDPNTLCDEFKADEK.K
□5374	139	–	155	673.9924	2018.9554	2018.9619	-0.0065	1	75	6.5e-05	1Score > **45** indicates **identity** Score > **28** indicates **homology**	U	K.LKPDPNTLCDEFKADEK.K
□5375	139	–	155	673.9929	2018.9569	2018.9619	-0.0050	1	91	1.6e-06	1Score > **45** indicates **identity** Score > **28** indicates **homology** 1Score > **45** indicates	U	K.LKPDPNTLCDEFKADEK.K

5376	139	– 155	673.9943	2018.9611	2018.9619	-0.0008	1	75	6.5e-05	identity Score > **33** indicates **homology**	U	K.LKPDPNTLCDEFKADEK.K
5380	139	– 155	674.3177	2019.9313	2018.9619	0.9694	1	49	0.024	1Score > **45** indicates **identity** Score > **33** indicates **homology**	U	K.LKPDPNTLCDEFKADEK.K
5381	139	– 155	505.9924	2019.9405	2018.9619	0.9786	1	46	0.043	1Score > **45** indicates **identity** Score > **29** indicates **homology**	U	K.LKPDPNTLCDEFKADEK.K
5382	139	– 155	674.3214	2019.9424	2018.9619	0.9805	1	72	0.00011	1Score > **45** indicates **identity** Score > **28** indicates **homology**	U	K.LKPDPNTLCDEFKADEK.K
5383	139	– 155	1010.9821	2019.9496	2018.9619	0.9878	1	82	1.2e-05	1Score > **45** indicates **identity** Score > **29** indicates **homology**	U	K.LKPDPNTLCDEFKADEK.K
5384	139	– 155	674.3242	2019.9508	2018.9619	0.9889	1	71	0.00016	1Score > **45** indicates **identity** Score > **27** indicates **homology**	U	K.LKPDPNTLCDEFKADEK.K
5386	139	– 155	674.3251	2019.9535	2018.9619	0.9916	1	78	2.9e-05	1Score > **45** indicates **identity** Score > **28** indicates **homology**	U	K.LKPDPNTLCDEFKADEK.K
5387	139	– 155	505.9957	2019.9537	2018.9619	0.9918	1	47	0.039	1Score > **45** indicates **identity** Score > **31** indicates **homology**	U	K.LKPDPNTLCDEFKADEK.K
5388	139	– 155	505.9957	2019.9537	2018.9619	0.9918	1	65	0.00064	1Score > **45** indicates **identity** Score > **29** indicates **homology**	U	K.LKPDPNTLCDEFKADEK.K
5389	139	– 155	1010.9845	2019.9544	2018.9619	0.9926	1	81	1.6e-05	1Score > **45** indicates **identity** Score > **35** indicates **homology**	U	K.LKPDPNTLCDEFKADEK.K
										1Score > **45** indicates **identity**		

☐5390	139	– 155	505.9959	2019.9545	2018.9619	0.9926	1	50	0.018		Score > **30** indicates **homology**	U	K.LKPDPNTLCDEFKADEK.K
☐5391	139	– 155	505.9959	2019.9545	2018.9619	0.9926	1	55	0.006	1	Score > **45** indicates **identity** Score > **28** indicates **homology**	U	K.LKPDPNTLCDEFKADEK.K
☐5392	139	– 155	505.9960	2019.9549	2018.9619	0.9930	1	49	0.023	1	Score > **45** indicates **identity** Score > **37** indicates **homology**	U	K.LKPDPNTLCDEFKADEK.K
☐5393	139	– 155	505.9960	2019.9549	2018.9619	0.9930	1	53	0.0089	1	Score > **45** indicates **identity** Score > **34** indicates **homology**	U	K.LKPDPNTLCDEFKADEK.K
☐5394	139	– 155	674.3256	2019.9550	2018.9619	0.9931	1	74	6.8e-05	1	Score > **45** indicates **identity** Score > **29** indicates **homology**	U	K.LKPDPNTLCDEFKADEK.K
☐5395	139	– 155	674.3256	2019.9550	2018.9619	0.9931	1	74	7e-05	1	Score > **45** indicates **identity** Score > **32** indicates **homology**	U	K.LKPDPNTLCDEFKADEK.K
☐5396	139	– 155	1010.9849	2019.9552	2018.9619	0.9934	1	92	1.2e-06	1	Score > **45** indicates **identity** Score > **29** indicates **homology**	U	K.LKPDPNTLCDEFKADEK.K
☐5397	139	– 155	674.3259	2019.9559	2018.9619	0.9940	1	59	0.0023	1	Score > **45** indicates **identity** Score > **32** indicates **homology**	U	K.LKPDPNTLCDEFKADEK.K
☐5445	168	– 183	1023.0097	2044.0048	2044.0206	-0.0158	1	48	0.026	1	Score > **45** indicates **identity** Score > **29** indicates **homology**		R.RHPYFYAPELLYYANK.Y
☐5450	168	– 183	1023.0146	2044.0146	2044.0206	-0.0060	1	51	0.013	1	Score > **45** indicates **identity** Score > **29** indicates **homology**		R.RHPYFYAPELLYYANK.Y
☐5470	168	– 183	1023.5169	2045.0192	2044.0206	0.9986	1	52	0.012	1	Score > **45** indicates **identity** Score >		R.RHPYFYAPELLYYANK.Y

										**30** indicates **homology**		
☐5184	169	– 183	629.9760	1886.9062	1887.9195	-1.0134	0	47	0.043	1Score **>** **46** indicates **identity** Score **>** **32** indicates **homology**		R.HPYFYAPELLYYANK.Y
☐5932	184	– 204	1244.0533	2486.0920	2486.1028	-0.0108	1	50	0.014	1Score **>** **44** indicates **identity** Score **>** **21** indicates **homology**		K.YNGVFQECCQAEDKGACLLPK.I
☐5933	184	– 204	1244.0533	2486.0920	2486.1028	-0.0108	1	56	0.0036	1Score **>** **44** indicates **identity** Score **>** **26** indicates **homology**		K.YNGVFQECCQAEDKGACLLPK.I
☐5934	184	– 204	1244.5452	2487.0758	2486.1028	0.9730	1	48	0.022	1Score **>** **44** indicates **identity** Score **>** **22** indicates **homology**		K.YNGVFQECCQAEDKGACLLPK.I
☐5935	184	– 204	1244.5475	2487.0804	2486.1028	0.9776	1	71	9.4e-05	1Score **>** **44** indicates **identity** Score **>** **23** indicates **homology**		K.YNGVFQECCQAEDKGACLLPK.I
☐5936	184	– 204	830.0377	2487.0913	2486.1028	0.9884	1	45	0.041	1Score **>** **44** indicates **identity** Score **>** **26** indicates **homology**		K.YNGVFQECCQAEDKGACLLPK.I
☐5938	184	– 204	830.0383	2487.0931	2486.1028	0.9902	1	44	0.047	1Score **>** **44** indicates **identity** Score **>** **25** indicates **homology**		K.YNGVFQECCQAEDKGACLLPK.I
☐2802	233	– 241	501.2960	1000.5774	1000.5818	-0.0043	1	49	0.04	2Score **>** **48** indicates **identity**		R.ALKAWSVAR.L
☐2803	233	– 241	501.2961	1000.5776	1000.5818	-0.0041	1	53	0.017	1Score **>** **48** indicates **identity**		R.ALKAWSVAR.L
☐3427	246	– 256	432.5721	1294.6945	1293.6969	0.9976	1	53	0.014	1Score **>** **47** indicates **identity** Score **>** **33** indicates **homology**	U	K.FPKAEFVEVTK.L
☐4769	249	– 263	846.9695	1691.9244	1691.9346	-0.0101	1	99	3e-07	1Score **>** **46** indicates **identity** Score **>** **42**	U	K.AEFVEVTKLVTDLTK.V

										indicates **homology**		
☐4770	249	– 263	564.9822	1691.9248	1691.9346	-0.0098	1	63	0.0011	1Score **> 46** indicates **identity** Score **> 36** indicates **homology**	U	K.AEFVEVTKLVTDLTK.V
☐4771	249	– 263	564.9822	1691.9248	1691.9346	-0.0098	1	80	2.4e-05	1Score **> 46** indicates **identity** Score **> 30** indicates **homology**	U	K.AEFVEVTKLVTDLTK.V
☐4772	249	– 263	564.9822	1691.9248	1691.9346	-0.0098	1	63	0.0011	1Score **> 46** indicates **identity** Score **> 35** indicates **homology**	U	K.AEFVEVTKLVTDLTK.V
☐4773	249	– 263	846.9699	1691.9252	1691.9346	-0.0093	1	95	7.2e-07	1Score **> 46** indicates **identity** Score **> 37** indicates **homology**	U	K.AEFVEVTKLVTDLTK.V
☐4774	249	– 263	564.9827	1691.9263	1691.9346	-0.0083	1	67	0.00042	1Score **> 46** indicates **identity** Score **> 31** indicates **homology**	U	K.AEFVEVTKLVTDLTK.V
☐4775	249	– 263	564.9827	1691.9263	1691.9346	-0.0083	1	72	0.00015	1Score **> 46** indicates **identity** Score **> 33** indicates **homology**	U	K.AEFVEVTKLVTDLTK.V
☐4776	249	– 263	564.9830	1691.9272	1691.9346	-0.0074	1	75	6.4e-05	1Score **> 46** indicates **identity** Score **> 32** indicates **homology**	U	K.AEFVEVTKLVTDLTK.V
☐4777	249	– 263	846.9709	1691.9272	1691.9346	-0.0073	1	99	2.9e-07	1Score **> 46** indicates **identity** Score **> 42** indicates **homology**	U	K.AEFVEVTKLVTDLTK.V
☐4778	249	– 263	846.9709	1691.9272	1691.9346	-0.0073	1	99	3e-07	1Score **> 46** indicates **identity** Score **> 42** indicates **homology**	U	K.AEFVEVTKLVTDLTK.V
☐4779	249	– 263	846.9711	1691.9276	1691.9346	-0.0069	1	99	2.9e-07	1Score **> 46** indicates **identity** Score **> 42** indicates	U	K.AEFVEVTKLVTDLTK.V

										**homology**		
☐4780	249	– 263	846.9711	1691.9276	1691.9346	-0.0069	1	99	2.8e-07	1Score > **46** indicates **identity** Score > **41** indicates **homology**	U	K.AEFVEVTKLVTDLTK.V
☐4781	249	– 263	564.9832	1691.9278	1691.9346	-0.0068	1	63	0.0011	1Score > **46** indicates **identity** Score > **38** indicates **homology**	U	K.AEFVEVTKLVTDLTK.V
☐4782	249	– 263	564.9832	1691.9278	1691.9346	-0.0068	1	72	0.00015	1Score > **46** indicates **identity** Score > **32** indicates **homology**	U	K.AEFVEVTKLVTDLTK.V
☐4783	249	– 263	846.9712	1691.9278	1691.9346	-0.0067	1	81	1.9e-05	1Score > **46** indicates **identity** Score > **42** indicates **homology**	U	K.AEFVEVTKLVTDLTK.V
☐4785	249	– 263	564.9833	1691.9281	1691.9346	-0.0065	1	67	0.00042	1Score > **46** indicates **identity** Score > **37** indicates **homology**	U	K.AEFVEVTKLVTDLTK.V
☐4786	249	– 263	846.9715	1691.9284	1691.9346	-0.0061	1	99	3e-07	1Score > **46** indicates **identity** Score > **42** indicates **homology**	U	K.AEFVEVTKLVTDLTK.V
☐4787	249	– 263	564.9835	1691.9287	1691.9346	-0.0059	1	80	2.4e-05	1Score > **46** indicates **identity** Score > **39** indicates **homology**	U	K.AEFVEVTKLVTDLTK.V
☐4788	249	– 263	846.9717	1691.9288	1691.9346	-0.0057	1	98	3.4e-07	1Score > **46** indicates **identity** Score > **42** indicates **homology**	U	K.AEFVEVTKLVTDLTK.V
☐4789	249	– 263	564.9836	1691.9290	1691.9346	-0.0056	1	85	7.5e-06	1Score > **46** indicates **identity** Score > **35** indicates **homology**	U	K.AEFVEVTKLVTDLTK.V
☐4790	249	– 263	564.9838	1691.9296	1691.9346	-0.0050	1	56	0.0057	1Score > **46** indicates **identity** Score > **35** indicates	U	K.AEFVEVTKLVTDLTK.V

												**homology**		
☐4792	249	–	263	564.9843	1691.9311	1691.9346	-0.0035	1	67	0.0004	1	Score **>** **46** indicates **identity** Score **>** **41** indicates **homology**	U	K.AEFVEVTKLVTDLTK.V
☐4800	249	–	263	847.4713	1692.9280	1691.9346	0.9935	1	89	2.8e-06	1	Score **>** **46** indicates **identity** Score **>** **42** indicates **homology**	U	K.AEFVEVTKLVTDLTK.V
☐4801	249	–	263	565.3170	1692.9292	1691.9346	0.9946	1	64	0.00093	1	Score **>** **46** indicates **identity** Score **>** **33** indicates **homology**	U	K.AEFVEVTKLVTDLTK.V
☐4802	249	–	263	565.3170	1692.9292	1691.9346	0.9946	1	85	7.6e-06	1	Score **>** **46** indicates **identity** Score **>** **36** indicates **homology**	U	K.AEFVEVTKLVTDLTK.V
☐4803	249	–	263	565.3174	1692.9304	1691.9346	0.9958	1	52	0.014	1	Score **>** **46** indicates **identity** Score **>** **33** indicates **homology**	U	K.AEFVEVTKLVTDLTK.V
☐4804	249	–	263	565.3174	1692.9304	1691.9346	0.9958	1	84	7.9e-06	1	Score **>** **46** indicates **identity** Score **>** **36** indicates **homology**	U	K.AEFVEVTKLVTDLTK.V
☐4805	249	–	263	565.3174	1692.9304	1691.9346	0.9958	1	59	0.0025	1	Score **>** **46** indicates **identity** Score **>** **34** indicates **homology**	U	K.AEFVEVTKLVTDLTK.V
☐4806	249	–	263	565.3176	1692.9310	1691.9346	0.9964	1	63	0.0011	1	Score **>** **46** indicates **identity** Score **>** **38** indicates **homology**	U	K.AEFVEVTKLVTDLTK.V
☐4807	249	–	263	847.4730	1692.9314	1691.9346	0.9969	1	64	0.00087	1	Score **>** **46** indicates **identity** Score **>** **33** indicates **homology**	U	K.AEFVEVTKLVTDLTK.V
☐3156	257	–	266	577.3480	1152.6814	1152.6867	-0.0052	1	56	0.0061	1	Score **>** **47** indicates **identity** Score **>** **34** indicates **homology**	U	K.LVTDLTKVHK.E

3160	257	– 266	385.5687	1153.6843	1152.6867	0.9976	1	65	0.00087	1Score > **47** indicates **identity** Score > **35** indicates **homology**	U	K.LVTDLTKVHK.E
5673	267	– 285	1124.4695	2246.9244	2246.9354	-0.0110	1	78	2.5e-05	1Score > **44** indicates **identity** Score > **24** indicates **homology**		K.ECCHGDLLECADDRADLAK.Y
5678	267	– 285	1124.9740	2247.9334	2246.9354	0.9980	1	67	0.00031	1Score > **44** indicates **identity** Score > **24** indicates **homology**		K.ECCHGDLLECADDRADLAK.Y
5679	267	– 285	750.3186	2247.9340	2246.9354	0.9985	1	46	0.036	1Score > **44** indicates **identity** Score > **26** indicates **homology**		K.ECCHGDLLECADDRADLAK.Y
3978	286	– 297	722.3214	1442.6282	1442.6348	-0.0065	0	74	8.9e-05	1Score > **46** indicates **identity** Score > **32** indicates **homology**	U	K.YICDNQDTISSK.L
3979	286	– 297	722.3219	1442.6292	1442.6348	-0.0055	0	82	1.6e-05	1Score > **46** indicates **identity** Score > **40** indicates **homology**	U	K.YICDNQDTISSK.L
4745	286	– 299	842.9105	1683.8064	1683.8138	-0.0073	1	78	3.2e-05	1Score > **46** indicates **identity** Score > **41** indicates **homology**	U	K.YICDNQDTISSKLK.E
4752	286	– 299	843.4115	1684.8084	1683.8138	0.9947	1	63	0.0011	1Score > **46** indicates **identity** Score > **32** indicates **homology**	U	K.YICDNQDTISSKLK.E
4252	298	– 309	766.8900	1531.7654	1531.7738	-0.0084	1	58	0.0042	1Score > **46** indicates **identity** Score > **45** indicates **homology**		K.LKECCDKPLLEK.S
4257	298	– 309	511.5960	1531.7662	1531.7738	-0.0076	1	56	0.0066	1Score > **46** indicates **identity** Score > **37** indicates **homology**		K.LKECCDKPLLEK.S
										1Score >		

4258	298 – 309	511.5960	1531.7662	1531.7738	-0.0076	1	61	0.0019	**46** indicates **identity** Score > **36** indicates **homology**		K.LKECCDKPLLEK.S
4260	298 – 309	766.8904	1531.7662	1531.7738	-0.0076	1	66	0.00068	1Score > **46** indicates **identity** Score > **46** indicates **homology**		K.LKECCDKPLLEK.S
4261	298 – 309	511.5961	1531.7665	1531.7738	-0.0073	1	59	0.0032	1Score > **46** indicates **identity** Score > **35** indicates **homology**		K.LKECCDKPLLEK.S
4265	298 – 309	766.8911	1531.7676	1531.7738	-0.0062	1	68	0.00038	1Score > **46** indicates **identity**		K.LKECCDKPLLEK.S
4266	298 – 309	766.8911	1531.7676	1531.7738	-0.0062	1	59	0.0034	1Score > **46** indicates **identity**		K.LKECCDKPLLEK.S
4267	298 – 309	511.5965	1531.7677	1531.7738	-0.0061	1	51	0.019	1Score > **46** indicates **identity** Score > **34** indicates **homology**		K.LKECCDKPLLEK.S
4268	298 – 309	511.5967	1531.7683	1531.7738	-0.0055	1	57	0.0054	1Score > **46** indicates **identity** Score > **35** indicates **homology**		K.LKECCDKPLLEK.S
4269	298 – 309	511.5968	1531.7686	1531.7738	-0.0052	1	55	0.0071	1Score > **46** indicates **identity** Score > **35** indicates **homology**		K.LKECCDKPLLEK.S
4270	298 – 309	511.5968	1531.7686	1531.7738	-0.0052	1	54	0.011	1Score > **46** indicates **identity** Score > **33** indicates **homology**		K.LKECCDKPLLEK.S
5734	341 – 359	1151.0381	2300.0616	2300.0749	-0.0133	1	131	1.1e-10	1Score > **44** indicates **identity** Score > **29** indicates **homology**	U	K.NYQEAKDAFLGSFLYEYSR.R
5735	341 – 359	767.6964	2300.0674	2300.0749	-0.0075	1	86	3.6e-06	1Score > **44** indicates **identity** Score > **29** indicates **homology**	U	K.NYQEAKDAFLGSFLYEYSR.R

1Score >

☐5737	341	– 359	768.0284	2301.0634	2300.0749	0.9885	1	87	3.5e-06	45 indicates **identity** Score > **35** indicates **homology**	U	K.NYQEAKDAFLGSFLYEYSR.R
☐5738	341	– 359	768.0288	2301.0646	2300.0749	0.9897	1	89	2.1e-06	1Score > **45** indicates **identity** Score > **30** indicates **homology**	U	K.NYQEAKDAFLGSFLYEYSR.R
☐5739	341	– 359	768.0291	2301.0655	2300.0749	0.9906	1	98	2.8e-07	1Score > **45** indicates **identity** Score > **29** indicates **homology**	U	K.NYQEAKDAFLGSFLYEYSR.R
☐5740	341	– 359	768.0292	2301.0658	2300.0749	0.9909	1	111	1.2e-08	1Score > **45** indicates **identity** Score > **30** indicates **homology**	U	K.NYQEAKDAFLGSFLYEYSR.R
☐5741	341	– 359	768.0298	2301.0676	2300.0749	0.9927	1	90	1.5e-06	1Score > **45** indicates **identity** Score > **30** indicates **homology**	U	K.NYQEAKDAFLGSFLYEYSR.R
☐5742	341	– 359	768.0303	2301.0691	2300.0749	0.9942	1	73	7.2e-05	1Score > **45** indicates **identity** Score > **29** indicates **homology**	U	K.NYQEAKDAFLGSFLYEYSR.R
☐5743	341	– 359	1151.5422	2301.0698	2300.0749	0.9949	1	115	5.6e-09	1Score > **45** indicates **identity** Score > **31** indicates **homology**	U	K.NYQEAKDAFLGSFLYEYSR.R
☐5744	341	– 359	768.0308	2301.0706	2300.0749	0.9957	1	86	4.2e-06	1Score > **45** indicates **identity** Score > **30** indicates **homology**	U	K.NYQEAKDAFLGSFLYEYSR.R
☐4383	347	– 359	1567.7288	1566.7215	1566.7354	-0.0139	0	94	8.3e-07	1Score > **46** indicates **identity** Score > **31** indicates **homology**	U	K.DAFLGSFLYEYSR.R
☐4384	347	– 359	1567.7314	1566.7241	1566.7354	-0.0113	0	91	1.7e-06	1Score > **46** indicates **identity** Score > **35** indicates **homology**	U	K.DAFLGSFLYEYSR.R

1Score >

[4385](#)	347	– 359	523.2493	1566.7261	1566.7354	-0.0094	0	64	0.00088	**46** indicates **identity** Score **>** **42** indicates **homology**	U	K.DAFLGSFLYEYSR.R
[4386](#)	347	– 359	784.3707	1566.7268	1566.7354	-0.0086	0	63	0.0012	1Score **>** **46** indicates **identity** Score **>** **32** indicates **homology**	U	K.DAFLGSFLYEYSR.R
[4387](#)	347	– 359	784.3709	1566.7272	1566.7354	-0.0082	0	71	0.0002	1Score **>** **46** indicates **identity** Score **>** **34** indicates **homology**	U	K.DAFLGSFLYEYSR.R
[4388](#)	347	– 359	784.3712	1566.7278	1566.7354	-0.0076	0	71	0.00021	1Score **>** **46** indicates **identity** Score **>** **35** indicates **homology**	U	K.DAFLGSFLYEYSR.R
[4389](#)	347	– 359	784.3713	1566.7280	1566.7354	-0.0074	0	66	0.00053	1Score **>** **46** indicates **identity** Score **>** **33** indicates **homology**	U	K.DAFLGSFLYEYSR.R
[4390](#)	347	– 359	784.3717	1566.7288	1566.7354	-0.0066	0	71	0.0002	1Score **>** **46** indicates **identity** Score **>** **36** indicates **homology**	U	K.DAFLGSFLYEYSR.R
[4391](#)	347	– 359	784.3718	1566.7290	1566.7354	-0.0064	0	73	0.00011	1Score **>** **46** indicates **identity** Score **>** **35** indicates **homology**	U	K.DAFLGSFLYEYSR.R
[4392](#)	347	– 359	784.3718	1566.7290	1566.7354	-0.0064	0	67	0.00048	1Score **>** **46** indicates **identity** Score **>** **34** indicates **homology**	U	K.DAFLGSFLYEYSR.R
[4394](#)	347	– 359	784.3719	1566.7292	1566.7354	-0.0062	0	55	0.0082	1Score **>** **46** indicates **identity** Score **>** **37** indicates **homology**	U	K.DAFLGSFLYEYSR.R
[4395](#)	347	– 359	784.3719	1566.7292	1566.7354	-0.0062	0	70	0.00022	1Score **>** **46** indicates **identity** Score **>** **35** indicates **homology**	U	K.DAFLGSFLYEYSR.R
										1Score **>**		

4396	347	– 359	784.3719	1566.7292	1566.7354	-0.0062	0	75	6.8e-05		**46** indicates **identity** Score > **38** indicates **homology**	U	K.DAFLGSFLYEYSR.R
4397	347	– 359	784.3719	1566.7292	1566.7354	-0.0062	0	75	6.7e-05	1	Score > **46** indicates **identity** Score > **38** indicates **homology**	U	K.DAFLGSFLYEYSR.R
4398	347	– 359	784.3720	1566.7294	1566.7354	-0.0060	0	63	0.0012	1	Score > **46** indicates **identity** Score > **32** indicates **homology**	U	K.DAFLGSFLYEYSR.R
4399	347	– 359	784.3721	1566.7296	1566.7354	-0.0058	0	70	0.00021	1	Score > **46** indicates **identity** Score > **35** indicates **homology**	U	K.DAFLGSFLYEYSR.R
4400	347	– 359	784.3721	1566.7296	1566.7354	-0.0058	0	71	0.0002	1	Score > **46** indicates **identity** Score > **36** indicates **homology**	U	K.DAFLGSFLYEYSR.R
4401	347	– 359	784.3722	1566.7298	1566.7354	-0.0056	0	67	0.00051	1	Score > **46** indicates **identity** Score > **34** indicates **homology**	U	K.DAFLGSFLYEYSR.R
4402	347	– 359	784.3723	1566.7300	1566.7354	-0.0054	0	67	0.0005	1	Score > **46** indicates **identity** Score > **34** indicates **homology**	U	K.DAFLGSFLYEYSR.R
4403	347	– 359	784.3724	1566.7302	1566.7354	-0.0052	0	75	6.9e-05	1	Score > **46** indicates **identity** Score > **38** indicates **homology**	U	K.DAFLGSFLYEYSR.R
4404	347	– 359	784.3724	1566.7302	1566.7354	-0.0052	0	75	7e-05	1	Score > **46** indicates **identity** Score > **38** indicates **homology**	U	K.DAFLGSFLYEYSR.R
4405	347	– 359	784.3725	1566.7304	1566.7354	-0.0050	0	70	0.00021	1	Score > **46** indicates **identity** Score > **35** indicates **homology**	U	K.DAFLGSFLYEYSR.R
										1	Score > **46**		

4406	347	–	359	784.3725	1566.7304	1566.7354	-0.0050	0	71	0.0002	indicates **identity** Score **>** **36** indicates **homology**	U	K.DAFLGSFLYEYSR.R
4407	347	–	359	784.3725	1566.7304	1566.7354	-0.0050	0	66	0.00054	1Score **>** **46** indicates **identity** Score **>** **33** indicates **homology**	U	K.DAFLGSFLYEYSR.R
4408	347	–	359	784.3725	1566.7304	1566.7354	-0.0050	0	63	0.0012	1Score **>** **46** indicates **identity** Score **>** **32** indicates **homology**	U	K.DAFLGSFLYEYSR.R
4409	347	–	359	784.3726	1566.7306	1566.7354	-0.0048	0	61	0.0019	1Score **>** **46** indicates **identity** Score **>** **35** indicates **homology**	U	K.DAFLGSFLYEYSR.R
4410	347	–	359	784.3726	1566.7306	1566.7354	-0.0048	0	66	0.00064	1Score **>** **46** indicates **identity** Score **>** **33** indicates **homology**	U	K.DAFLGSFLYEYSR.R
4411	347	–	359	784.3726	1566.7306	1566.7354	-0.0048	0	81	1.7e-05	1Score **>** **46** indicates **identity** Score **>** **41** indicates **homology**	U	K.DAFLGSFLYEYSR.R
4412	347	–	359	784.3726	1566.7306	1566.7354	-0.0048	0	75	6.7e-05	1Score **>** **46** indicates **identity** Score **>** **38** indicates **homology**	U	K.DAFLGSFLYEYSR.R
4413	347	–	359	784.3727	1566.7308	1566.7354	-0.0046	0	50	0.023	1Score **>** **46** indicates **identity** Score **>** **37** indicates **homology**	U	K.DAFLGSFLYEYSR.R
4414	347	–	359	784.3727	1566.7308	1566.7354	-0.0046	0	71	0.0002	1Score **>** **46** indicates **identity** Score **>** **34** indicates **homology**	U	K.DAFLGSFLYEYSR.R
4415	347	–	359	784.3729	1566.7312	1566.7354	-0.0042	0	63	0.0012	1Score **>** **46** indicates **identity** Score **>** **32** indicates **homology**	U	K.DAFLGSFLYEYSR.R
											1Score **>** **46** indicates		

4416	347	–	359	784.3731	1566.7316	1566.7354	-0.0038	0	66	0.00053	**identity** Score > **34** indicates **homology**	1	U	K.DAFLGSFLYEYSR.R
4417	347	–	359	784.3733	1566.7320	1566.7354	-0.0034	0	71	0.00021	Score > **46** indicates **identity** Score > **34** indicates **homology**	1	U	K.DAFLGSFLYEYSR.R
4418	347	–	359	784.3734	1566.7322	1566.7354	-0.0032	0	71	0.00018	Score > **46** indicates **identity** Score > **35** indicates **homology**	1	U	K.DAFLGSFLYEYSR.R
4419	347	–	359	784.3735	1566.7324	1566.7354	-0.0030	0	70	0.00021	Score > **46** indicates **identity** Score > **35** indicates **homology**	1	U	K.DAFLGSFLYEYSR.R
4420	347	–	359	784.3737	1566.7328	1566.7354	-0.0026	0	66	0.00054	Score > **46** indicates **identity** Score > **33** indicates **homology**	1	U	K.DAFLGSFLYEYSR.R
4421	347	–	359	784.3741	1566.7336	1566.7354	-0.0018	0	71	0.00019	Score > **46** indicates **identity** Score > **35** indicates **homology**	1	U	K.DAFLGSFLYEYSR.R
4422	347	–	359	784.3754	1566.7362	1566.7354	0.0008	0	75	7.1e-05	Score > **46** indicates **identity** Score > **36** indicates **homology**	1	U	K.DAFLGSFLYEYSR.R
4427	347	–	359	1568.7373	1567.7300	1566.7354	0.9946	0	50	0.026	Score > **46** indicates **identity** Score > **32** indicates **homology**	1	U	K.DAFLGSFLYEYSR.R
4428	347	–	359	784.8735	1567.7324	1566.7354	0.9970	0	70	0.00024	Score > **46** indicates **identity** Score > **36** indicates **homology**	1	U	K.DAFLGSFLYEYSR.R
4429	347	–	359	784.8735	1567.7324	1566.7354	0.9970	0	59	0.0027	Score > **46** indicates **identity** Score > **34** indicates **homology**	1	U	K.DAFLGSFLYEYSR.R
4430	347	–	359	784.8740	1567.7334	1566.7354	0.9980	0	66	0.00059	Score > **46** indicates **identity**		U	K.DAFLGSFLYEYSR.R

											Score > **31** indicates **homology**		
☐4431	347	–	359	784.8746	1567.7346	1566.7354	0.9992	0	56	0.0053	1 Score > **46** indicates **identity** Score > **32** indicates **homology**	U	K.DAFLGSFLYEYSR.R
☐4432	347	–	359	784.8747	1567.7348	1566.7354	0.9994	0	61	0.0018	1 Score > **46** indicates **identity** Score > **35** indicates **homology**	U	K.DAFLGSFLYEYSR.R
☐3848	360	–	371	720.1332	1438.2518	1438.8045	-0.5526	1	50	0.015	1 Score > **45** indicates **identity** Score > **33** indicates **homology**		R.RHPEYAVSVLLR.L
☐3849	360	–	371	720.3298	1438.6450	1438.8045	-0.1594	1	56	0.0067	1 Score > **46** indicates **identity** Score > **34** indicates **homology**		R.RHPEYAVSVLLR.L
☐3850	360	–	371	720.4041	1438.7936	1438.8045	-0.0108	1	58	0.004	1 Score > **47** indicates **identity** Score > **35** indicates **homology**		R.RHPEYAVSVLLR.L
☐3851	360	–	371	720.4051	1438.7956	1438.8045	-0.0088	1	70	0.00027	1 Score > **47** indicates **identity** Score > **35** indicates **homology**		R.RHPEYAVSVLLR.L
☐3852	360	–	371	480.6059	1438.7959	1438.8045	-0.0086	1	50	0.028	1 Score > **47** indicates **identity** Score > **44** indicates **homology**		R.RHPEYAVSVLLR.L
☐3853	360	–	371	480.6061	1438.7965	1438.8045	-0.0080	1	62	0.0016	1 Score > **47** indicates **identity** Score > **36** indicates **homology**		R.RHPEYAVSVLLR.L
☐3854	360	–	371	480.6061	1438.7965	1438.8045	-0.0080	1	61	0.0019	1 Score > **47** indicates **identity** Score > **37** indicates **homology**		R.RHPEYAVSVLLR.L
☐3857	360	–	371	480.6063	1438.7971	1438.8045	-0.0074	1	78	4.3e-05	1 Score > **47** indicates **identity**		R.RHPEYAVSVLLR.L

												Score > **36** indicates **homology**	
☐3858	360	–	371	720.4059	1438.7972	1438.8045	-0.0072	1	55	0.0072	1	Score > **47** indicates **identity** Score > **35** indicates **homology**	R.RHPEYAVSVLLR.L
☐3859	360	–	371	480.6064	1438.7974	1438.8045	-0.0071	1	50	0.023	1	Score > **47** indicates **identity** Score > **31** indicates **homology**	R.RHPEYAVSVLLR.L
☐3861	360	–	371	480.6064	1438.7974	1438.8045	-0.0071	1	62	0.0016	1	Score > **47** indicates **identity** Score > **36** indicates **homology**	R.RHPEYAVSVLLR.L
☐3862	360	–	371	720.4061	1438.7976	1438.8045	-0.0068	1	68	0.00041	1	Score > **47** indicates **identity** Score > **40** indicates **homology**	R.RHPEYAVSVLLR.L
☐3863	360	–	371	480.6065	1438.7977	1438.8045	-0.0068	1	70	0.00028	1	Score > **47** indicates **identity** Score > **34** indicates **homology**	R.RHPEYAVSVLLR.L
☐3864	360	–	371	480.6065	1438.7977	1438.8045	-0.0068	1	55	0.0085	1	Score > **47** indicates **identity** Score > **30** indicates **homology**	R.RHPEYAVSVLLR.L
☐3865	360	–	371	480.6065	1438.7977	1438.8045	-0.0068	1	62	0.0016	1	Score > **47** indicates **identity** Score > **35** indicates **homology**	R.RHPEYAVSVLLR.L
☐3866	360	–	371	480.6065	1438.7977	1438.8045	-0.0068	1	75	8.2e-05	1	Score > **47** indicates **identity** Score > **33** indicates **homology**	R.RHPEYAVSVLLR.L
☐3868	360	–	371	720.4062	1438.7978	1438.8045	-0.0066	1	66	0.00064	1	Score > **47** indicates **identity** Score > **36** indicates **homology**	R.RHPEYAVSVLLR.L
☐3869	360	–	371	720.4062	1438.7978	1438.8045	-0.0066	1	74	8.9e-05	1	Score > **47** indicates **identity** Score >	R.RHPEYAVSVLLR.L

											**38** indicates **homology**	
☐[3871]	360	– 371	480.6066	1438.7980	1438.8045	-0.0065	1	62	0.0016	1	Score > **47** indicates **identity** Score > **35** indicates **homology**	R.RHPEYAVSVLLR.L
☐[3872]	360	– 371	480.6066	1438.7980	1438.8045	-0.0065	1	59	0.0032	1	Score > **47** indicates **identity** Score > **35** indicates **homology**	R.RHPEYAVSVLLR.L
☐[3873]	360	– 371	480.6066	1438.7980	1438.8045	-0.0065	1	62	0.0016	1	Score > **47** indicates **identity** Score > **35** indicates **homology**	R.RHPEYAVSVLLR.L
☐[3874]	360	– 371	480.6066	1438.7980	1438.8045	-0.0065	1	62	0.0017	1	Score > **47** indicates **identity** Score > **36** indicates **homology**	R.RHPEYAVSVLLR.L
☐[3875]	360	– 371	720.4063	1438.7980	1438.8045	-0.0064	1	54	0.0098	1	Score > **47** indicates **identity** Score > **35** indicates **homology**	R.RHPEYAVSVLLR.L
☐[3876]	360	– 371	720.4063	1438.7980	1438.8045	-0.0064	1	70	0.00023	1	Score > **47** indicates **identity** Score > **37** indicates **homology**	R.RHPEYAVSVLLR.L
☐[3879]	360	– 371	480.6067	1438.7983	1438.8045	-0.0062	1	57	0.0055	1	Score > **47** indicates **identity** Score > **33** indicates **homology**	R.RHPEYAVSVLLR.L
☐[3880]	360	– 371	480.6067	1438.7983	1438.8045	-0.0062	1	61	0.0019	1	Score > **47** indicates **identity** Score > **36** indicates **homology**	R.RHPEYAVSVLLR.L
☐[3881]	360	– 371	480.6067	1438.7983	1438.8045	-0.0062	1	65	0.00087	1	Score > **47** indicates **identity** Score > **36** indicates **homology**	R.RHPEYAVSVLLR.L
☐[3882]	360	– 371	480.6067	1438.7983	1438.8045	-0.0062	1	65	0.00082	1	Score > **47** indicates **identity** Score >	R.RHPEYAVSVLLR.L

										**37** indicates **homology**	
3883	360	– 371	480.6067	1438.7983	1438.8045	-0.0062	1	71	0.00019	1Score > **47** indicates **identity** Score > **34** indicates **homology**	R.RHPEYAVSVLLR.L
3884	360	– 371	480.6067	1438.7983	1438.8045	-0.0062	1	65	0.00089	1Score > **47** indicates **identity** Score > **35** indicates **homology**	R.RHPEYAVSVLLR.L
3885	360	– 371	480.6067	1438.7983	1438.8045	-0.0062	1	54	0.01	1Score > **47** indicates **identity** Score > **31** indicates **homology**	R.RHPEYAVSVLLR.L
3886	360	– 371	480.6067	1438.7983	1438.8045	-0.0062	1	70	0.00024	1Score > **47** indicates **identity** Score > **32** indicates **homology**	R.RHPEYAVSVLLR.L
3887	360	– 371	480.6067	1438.7983	1438.8045	-0.0062	1	59	0.0033	1Score > **47** indicates **identity** Score > **34** indicates **homology**	R.RHPEYAVSVLLR.L
3888	360	– 371	480.6067	1438.7983	1438.8045	-0.0062	1	65	0.00075	1Score > **47** indicates **identity** Score > **32** indicates **homology**	R.RHPEYAVSVLLR.L
3890	360	– 371	480.6068	1438.7986	1438.8045	-0.0059	1	56	0.0068	1Score > **47** indicates **identity** Score > **32** indicates **homology**	R.RHPEYAVSVLLR.L
3891	360	– 371	480.6068	1438.7986	1438.8045	-0.0059	1	62	0.0016	1Score > **47** indicates **identity** Score > **32** indicates **homology**	R.RHPEYAVSVLLR.L
3892	360	– 371	480.6068	1438.7986	1438.8045	-0.0059	1	53	0.014	1Score > **47** indicates **identity** Score > **36** indicates **homology**	R.RHPEYAVSVLLR.L
3893	360	– 371	480.6068	1438.7986	1438.8045	-0.0059	1	71	0.0002	1Score > **47** indicates **identity** Score > **34**	R.RHPEYAVSVLLR.L

											indicates **homology**	
3894	360	–	371	480.6068	1438.7986	1438.8045	-0.0059	1	61	0.0019	1Score **> 47** indicates **identity** Score **> 36** indicates **homology**	R.RHPEYAVSVLLR.L
3895	360	–	371	480.6068	1438.7986	1438.8045	-0.0059	1	62	0.0016	1Score **> 47** indicates **identity** Score **> 36** indicates **homology**	R.RHPEYAVSVLLR.L
3896	360	–	371	480.6068	1438.7986	1438.8045	-0.0059	1	54	0.011	1Score **> 47** indicates **identity** Score **> 31** indicates **homology**	R.RHPEYAVSVLLR.L
3897	360	–	371	720.4066	1438.7986	1438.8045	-0.0058	1	68	0.0004	1Score **> 47** indicates **identity** Score **> 35** indicates **homology**	R.RHPEYAVSVLLR.L
3898	360	–	371	720.4067	1438.7988	1438.8045	-0.0056	1	72	0.00017	1Score **> 47** indicates **identity** Score **> 37** indicates **homology**	R.RHPEYAVSVLLR.L
3899	360	–	371	720.4067	1438.7988	1438.8045	-0.0056	1	65	0.00088	1Score **> 47** indicates **identity** Score **> 41** indicates **homology**	R.RHPEYAVSVLLR.L
3900	360	–	371	480.6069	1438.7989	1438.8045	-0.0056	1	65	0.00088	1Score **> 47** indicates **identity** Score **> 36** indicates **homology**	R.RHPEYAVSVLLR.L
3901	360	–	371	480.6069	1438.7989	1438.8045	-0.0056	1	57	0.0055	1Score **> 47** indicates **identity** Score **> 35** indicates **homology**	R.RHPEYAVSVLLR.L
3902	360	–	371	480.6069	1438.7989	1438.8045	-0.0056	1	70	0.00023	1Score **> 47** indicates **identity** Score **> 46** indicates **homology**	R.RHPEYAVSVLLR.L
3903	360	–	371	480.6069	1438.7989	1438.8045	-0.0056	1	74	8.9e-05	1Score **> 47** indicates **identity** Score **> 37** indicates	R.RHPEYAVSVLLR.L

											**homology**	
☐3904	360	–	371	480.6069	1438.7989	1438.8045	-0.0056	1	62	0.0016	1Score > **47** indicates **identity** Score > **35** indicates **homology**	R.RHPEYAVSVLLR.L
☐3907	360	–	371	480.6070	1438.7992	1438.8045	-0.0053	1	68	0.00038	1Score > **47** indicates **identity** Score > **41** indicates **homology**	R.RHPEYAVSVLLR.L
☐3908	360	–	371	480.6070	1438.7992	1438.8045	-0.0053	1	59	0.0032	1Score > **47** indicates **identity** Score > **34** indicates **homology**	R.RHPEYAVSVLLR.L
☐3909	360	–	371	480.6070	1438.7992	1438.8045	-0.0053	1	62	0.0016	1Score > **47** indicates **identity** Score > **35** indicates **homology**	R.RHPEYAVSVLLR.L
☐3910	360	–	371	480.6070	1438.7992	1438.8045	-0.0053	1	61	0.0019	1Score > **47** indicates **identity** Score > **37** indicates **homology**	R.RHPEYAVSVLLR.L
☐3911	360	–	371	480.6070	1438.7992	1438.8045	-0.0053	1	62	0.0015	1Score > **47** indicates **identity** Score > **35** indicates **homology**	R.RHPEYAVSVLLR.L
☐3912	360	–	371	480.6070	1438.7992	1438.8045	-0.0053	1	62	0.0016	1Score > **47** indicates **identity** Score > **35** indicates **homology**	R.RHPEYAVSVLLR.L
☐3913	360	–	371	480.6070	1438.7992	1438.8045	-0.0053	1	62	0.0016	1Score > **47** indicates **identity** Score > **36** indicates **homology**	R.RHPEYAVSVLLR.L
☐3914	360	–	371	480.6070	1438.7992	1438.8045	-0.0053	1	54	0.0094	1Score > **47** indicates **identity** Score > **34** indicates **homology**	R.RHPEYAVSVLLR.L
☐3915	360	–	371	720.4069	1438.7992	1438.8045	-0.0052	1	72	0.00018	1Score > **47** indicates **identity** Score > **38** indicates **homology**	R.RHPEYAVSVLLR.L

3916	360	– 371	720.4069	1438.7992	1438.8045	-0.0052	1	60	0.0023	1Score **> 47** indicates **identity** Score **> 35** indicates **homology**	R.RHPEYAVSVLLR.L
3917	360	– 371	720.4069	1438.7992	1438.8045	-0.0052	1	69	0.00032	1Score **> 47** indicates **identity** Score **> 38** indicates **homology**	R.RHPEYAVSVLLR.L
3918	360	– 371	720.4070	1438.7994	1438.8045	-0.0050	1	66	0.0006	1Score **> 47** indicates **identity** Score **> 36** indicates **homology**	R.RHPEYAVSVLLR.L
3919	360	– 371	480.6071	1438.7995	1438.8045	-0.0050	1	62	0.0016	1Score **> 47** indicates **identity** Score **> 36** indicates **homology**	R.RHPEYAVSVLLR.L
3920	360	– 371	480.6071	1438.7995	1438.8045	-0.0050	1	52	0.016	1Score **> 47** indicates **identity** Score **> 34** indicates **homology**	R.RHPEYAVSVLLR.L
3921	360	– 371	480.6071	1438.7995	1438.8045	-0.0050	1	65	0.00085	1Score **> 47** indicates **identity** Score **> 41** indicates **homology**	R.RHPEYAVSVLLR.L
3922	360	– 371	480.6071	1438.7995	1438.8045	-0.0050	1	62	0.0016	1Score **> 47** indicates **identity** Score **> 36** indicates **homology**	R.RHPEYAVSVLLR.L
3923	360	– 371	480.6071	1438.7995	1438.8045	-0.0050	1	61	0.0018	1Score **> 47** indicates **identity** Score **> 37** indicates **homology**	R.RHPEYAVSVLLR.L
3924	360	– 371	480.6071	1438.7995	1438.8045	-0.0050	1	62	0.0016	1Score **> 47** indicates **identity** Score **> 35** indicates **homology**	R.RHPEYAVSVLLR.L
3925	360	– 371	480.6071	1438.7995	1438.8045	-0.0050	1	57	0.0054	1Score **> 47** indicates **identity** Score **> 33** indicates **homology**	R.RHPEYAVSVLLR.L
										1Score **>**	

												**47** indicates **identity** Score > **34** indicates **homology**	
3926	360	–	371	480.6071	1438.7995	1438.8045	-0.0050	1	59	0.0034			R.RHPEYAVSVLLR.L
3927	360	–	371	480.6071	1438.7995	1438.8045	-0.0050	1	65	0.00087	1	Score > **47** indicates **identity** Score > **36** indicates **homology**	R.RHPEYAVSVLLR.L
3928	360	–	371	480.6071	1438.7995	1438.8045	-0.0050	1	53	0.013	1	Score > **47** indicates **identity** Score > **31** indicates **homology**	R.RHPEYAVSVLLR.L
3929	360	–	371	480.6071	1438.7995	1438.8045	-0.0050	1	60	0.0028	1	Score > **47** indicates **identity** Score > **34** indicates **homology**	R.RHPEYAVSVLLR.L
3930	360	–	371	480.6071	1438.7995	1438.8045	-0.0050	1	60	0.0027	1	Score > **47** indicates **identity** Score > **33** indicates **homology**	R.RHPEYAVSVLLR.L
3931	360	–	371	480.6071	1438.7995	1438.8045	-0.0050	1	60	0.0025	1	Score > **47** indicates **identity** Score > **32** indicates **homology**	R.RHPEYAVSVLLR.L
3932	360	–	371	720.4071	1438.7996	1438.8045	-0.0048	1	75	7.4e-05	1	Score > **47** indicates **identity** Score > **36** indicates **homology**	R.RHPEYAVSVLLR.L
3934	360	–	371	480.6072	1438.7998	1438.8045	-0.0047	1	61	0.0019	1	Score > **47** indicates **identity** Score > **36** indicates **homology**	R.RHPEYAVSVLLR.L
3935	360	–	371	480.6072	1438.7998	1438.8045	-0.0047	1	60	0.0024	1	Score > **47** indicates **identity** Score > **37** indicates **homology**	R.RHPEYAVSVLLR.L
3936	360	–	371	480.6072	1438.7998	1438.8045	-0.0047	1	57	0.0046	1	Score > **47** indicates **identity** Score > **32** indicates **homology**	R.RHPEYAVSVLLR.L
											1	Score > **47**	

☐3937	360	–	371	480.6072	1438.7998	1438.8045	-0.0047	1	62	0.0015	indicates **identity** Score **> 35** indicates **homology**	R.RHPEYAVSVLLR.L
☐3938	360	–	371	480.6072	1438.7998	1438.8045	-0.0047	1	60	0.0022	1Score **> 47** indicates **identity** Score **> 29** indicates **homology**	R.RHPEYAVSVLLR.L
☐3939	360	–	371	480.6072	1438.7998	1438.8045	-0.0047	1	65	0.00088	1Score **> 47** indicates **identity** Score **> 36** indicates **homology**	R.RHPEYAVSVLLR.L
☐3940	360	–	371	720.4073	1438.8000	1438.8045	-0.0044	1	66	0.0006	1Score **> 47** indicates **identity** Score **> 35** indicates **homology**	R.RHPEYAVSVLLR.L
☐3941	360	–	371	720.4073	1438.8000	1438.8045	-0.0044	1	56	0.006	1Score **> 47** indicates **identity** Score **> 31** indicates **homology**	R.RHPEYAVSVLLR.L
☐3942	360	–	371	480.6073	1438.8001	1438.8045	-0.0044	1	57	0.0046	1Score **> 47** indicates **identity** Score **> 33** indicates **homology**	R.RHPEYAVSVLLR.L
☐3943	360	–	371	480.6074	1438.8004	1438.8045	-0.0041	1	65	0.00087	1Score **> 47** indicates **identity** Score **> 36** indicates **homology**	R.RHPEYAVSVLLR.L
☐3944	360	–	371	480.6074	1438.8004	1438.8045	-0.0041	1	54	0.0094	1Score **> 47** indicates **identity** Score **> 33** indicates **homology**	R.RHPEYAVSVLLR.L
☐3946	360	–	371	480.6075	1438.8007	1438.8045	-0.0038	1	62	0.0015	1Score **> 47** indicates **identity** Score **> 36** indicates **homology**	R.RHPEYAVSVLLR.L
☐3947	360	–	371	480.6076	1438.8010	1438.8045	-0.0035	1	72	0.00016	1Score **> 47** indicates **identity** Score **> 33** indicates **homology**	R.RHPEYAVSVLLR.L
											1Score **> 46** indicates	

3948	360	– 371	720.4092	1438.8038	1438.8045	-0.0006	1	63	0.0013	**identity** Score > **38** indicates **homology**	R.RHPEYAVSVLLR.L
3949	360	– 371	720.4095	1438.8044	1438.8045	0.0000	1	64	0.0011	1Score > **46** indicates **identity** Score > **38** indicates **homology**	R.RHPEYAVSVLLR.L
3950	360	– 371	720.9019	1439.7892	1438.8045	0.9848	1	63	0.0014	1Score > **46** indicates **identity** Score > **37** indicates **homology**	R.RHPEYAVSVLLR.L
3951	360	– 371	720.9060	1439.7974	1438.8045	0.9930	1	65	0.00072	1Score > **46** indicates **identity** Score > **34** indicates **homology**	R.RHPEYAVSVLLR.L
3952	360	– 371	720.9064	1439.7982	1438.8045	0.9938	1	62	0.0015	1Score > **46** indicates **identity** Score > **36** indicates **homology**	R.RHPEYAVSVLLR.L
3953	360	– 371	720.9064	1439.7982	1438.8045	0.9938	1	59	0.0031	1Score > **46** indicates **identity** Score > **35** indicates **homology**	R.RHPEYAVSVLLR.L
3954	360	– 371	480.9405	1439.7997	1438.8045	0.9952	1	63	0.0014	1Score > **46** indicates **identity** Score > **36** indicates **homology**	R.RHPEYAVSVLLR.L
3957	360	– 371	480.9406	1439.8000	1438.8045	0.9955	1	61	0.002	1Score > **46** indicates **identity** Score > **37** indicates **homology**	R.RHPEYAVSVLLR.L
3958	360	– 371	720.9075	1439.8004	1438.8045	0.9960	1	62	0.0016	1Score > **46** indicates **identity** Score > **37** indicates **homology**	R.RHPEYAVSVLLR.L
3959	360	– 371	720.9077	1439.8008	1438.8045	0.9964	1	60	0.0026	1Score > **46** indicates **identity** Score > **37** indicates **homology** 1Score > **46** indicates **identity**	R.RHPEYAVSVLLR.L

☐3960	360	– 371	720.9077	1439.8008	1438.8045	0.9964	1	66	0.0007	Score **>** **35** indicates **homology**	R.RHPEYAVSVLLR.L
☐3962	360	– 371	720.9078	1439.8010	1438.8045	0.9966	1	65	0.00079	1Score **>** **46** indicates **identity** Score **>** **37** indicates **homology**	R.RHPEYAVSVLLR.L
☐3963	360	– 371	720.9078	1439.8010	1438.8045	0.9966	1	63	0.0014	1Score **>** **46** indicates **identity** Score **>** **38** indicates **homology**	R.RHPEYAVSVLLR.L
☐3964	360	– 371	720.9080	1439.8014	1438.8045	0.9970	1	68	0.00043	1Score **>** **46** indicates **identity** Score **>** **38** indicates **homology**	R.RHPEYAVSVLLR.L
☐3965	360	– 371	720.9081	1439.8016	1438.8045	0.9972	1	63	0.0013	1Score **>** **46** indicates **identity** Score **>** **37** indicates **homology**	R.RHPEYAVSVLLR.L
☐3967	360	– 371	720.9086	1439.8026	1438.8045	0.9982	1	63	0.0014	1Score **>** **46** indicates **identity** Score **>** **36** indicates **homology**	R.RHPEYAVSVLLR.L
☐3968	360	– 371	720.9088	1439.8030	1438.8045	0.9986	1	63	0.0012	1Score **>** **46** indicates **identity** Score **>** **37** indicates **homology**	R.RHPEYAVSVLLR.L
☐3970	360	– 371	720.9109	1439.8072	1438.8045	1.0028	1	63	0.0012	1Score **>** **46** indicates **identity** Score **>** **35** indicates **homology**	R.RHPEYAVSVLLR.L
☐3385	361	– 371	642.3547	1282.6948	1282.7034	-0.0085	0	53	0.013	1Score **>** **47** indicates **identity** Score **>** **35** indicates **homology**	R.HPEYAVSVLLR.L
☐3387	361	– 371	642.3557	1282.6968	1282.7034	-0.0065	0	56	0.0063	1Score **>** **47** indicates **identity** Score **>** **37** indicates **homology**	R.HPEYAVSVLLR.L
☐3388	361	– 371	642.3561	1282.6976	1282.7034	-0.0057	0	62	0.0015	1Score **>** **47** indicates **identity** Score **>**	R.HPEYAVSVLLR.L

										**37** indicates **homology**		
□[3389]	361	– 371	428.5732	1282.6978	1282.7034	-0.0056	0	49	0.031	1Score > **47** indicates **identity** Score > **38** indicates **homology**		R.HPEYAVSVLLR.L
□[3390]	361	– 371	428.5732	1282.6978	1282.7034	-0.0056	0	58	0.0046	1Score > **47** indicates **identity** Score > **41** indicates **homology**		R.HPEYAVSVLLR.L
□[3391]	361	– 371	642.3568	1282.6990	1282.7034	-0.0043	0	61	0.0023	1Score > **47** indicates **identity** Score > **37** indicates **homology**		R.HPEYAVSVLLR.L
□[3392]	361	– 371	642.3569	1282.6992	1282.7034	-0.0041	0	57	0.0048	1Score > **47** indicates **identity** Score > **38** indicates **homology**		R.HPEYAVSVLLR.L
□[3393]	361	– 371	642.3570	1282.6994	1282.7034	-0.0039	0	57	0.0053	1Score > **47** indicates **identity** Score > **38** indicates **homology**		R.HPEYAVSVLLR.L
□[3394]	361	– 371	428.5739	1282.6999	1282.7034	-0.0035	0	49	0.033	1Score > **47** indicates **identity** Score > **44** indicates **homology**		R.HPEYAVSVLLR.L
□[3397]	361	– 371	642.8580	1283.7014	1282.7034	0.9981	0	50	0.027	1Score > **47** indicates **identity** Score > **33** indicates **homology**		R.HPEYAVSVLLR.L
□[5023]	372	– 386	907.9128	1813.8110	1813.8226	-0.0115	1	61	0.0016	1Score > **46** indicates **identity** Score > **27** indicates **homology**	U	R.LAKEYEATLEECCAK.D
□[5025]	372	– 386	907.9162	1813.8178	1813.8226	-0.0047	1	75	6.5e-05	1Score > **46** indicates **identity** Score > **26** indicates **homology**	U	R.LAKEYEATLEECCAK.D
□[5027]	372	– 386	605.9465	1814.8177	1813.8226	0.9951	1	47	0.04	1Score > **46** indicates **identity** Score > **33**	U	R.LAKEYEATLEECCAK.D

												indicates **homology**		
3463	402	–	412	1305.7092	1304.7019	1304.7088	-0.0069	0	48	0.04	1	Score **> 47** indicates **identity** Score **> 35** indicates **homology**		K.HLVDEPQNLIK.Q
3468	402	–	412	653.3586	1304.7026	1304.7088	-0.0062	0	48	0.039	1	Score **> 47** indicates **identity** Score **> 31** indicates **homology**		K.HLVDEPQNLIK.Q
3471	402	–	412	653.3588	1304.7030	1304.7088	-0.0058	0	51	0.022	1	Score **> 47** indicates **identity** Score **> 26** indicates **homology**		K.HLVDEPQNLIK.Q
3473	402	–	412	653.3589	1304.7032	1304.7088	-0.0056	0	48	0.041	1	Score **> 47** indicates **identity** Score **> 29** indicates **homology**		K.HLVDEPQNLIK.Q
3477	402	–	412	1305.7109	1304.7036	1304.7088	-0.0052	0	48	0.042	1	Score **> 47** indicates **identity** Score **> 35** indicates **homology**		K.HLVDEPQNLIK.Q
3482	402	–	412	653.8584	1305.7022	1304.7088	0.9934	0	55	0.0077	1	Score **> 47** indicates **identity** Score **> 39** indicates **homology**		K.HLVDEPQNLIK.Q
5776	402	–	420	785.7141	2354.1205	2354.1325	-0.0120	1	57	0.0031	1	Score **> 44** indicates **identity** Score **> 25** indicates **homology**	U	K.HLVDEPQNLIKQNCDQFEK.L
5777	402	–	420	785.7141	2354.1205	2354.1325	-0.0120	1	70	0.00015	1	Score **> 44** indicates **identity** Score **> 26** indicates **homology**	U	K.HLVDEPQNLIKQNCDQFEK.L
5778	402	–	420	589.5380	2354.1229	2354.1325	-0.0096	1	54	0.007	1	Score **> 44** indicates **identity** Score **> 29** indicates **homology**	U	K.HLVDEPQNLIKQNCDQFEK.L
5780	402	–	420	786.0457	2355.1153	2354.1325	0.9828	1	64	0.00058	1	Score **> 44** indicates **identity** Score **> 31**	U	K.HLVDEPQNLIKQNCDQFEK.L

											indicates **homology**		
5781	402	–	420	589.7880	2355.1229	2354.1325	0.9904	1	58	0.0024	1Score **> 44** indicates **identity** Score **> 26** indicates **homology**	U	K.HLVDEPQNLIKQNCDQFEK.L
5783	402	–	420	786.0486	2355.1240	2354.1325	0.9915	1	61	0.0012	1Score **> 44** indicates **identity** Score **> 25** indicates **homology**	U	K.HLVDEPQNLIKQNCDQFEK.L
5784	402	–	420	786.0488	2355.1246	2354.1325	0.9921	1	63	0.00081	1Score **> 44** indicates **identity** Score **> 27** indicates **homology**	U	K.HLVDEPQNLIKQNCDQFEK.L
5785	402	–	420	1178.5696	2355.1246	2354.1325	0.9922	1	75	5.5e-05	1Score **> 44** indicates **identity** Score **> 22** indicates **homology**	U	K.HLVDEPQNLIKQNCDQFEK.L
5786	402	–	420	589.7885	2355.1249	2354.1325	0.9924	1	52	0.01	1Score **> 44** indicates **identity** Score **> 27** indicates **homology**	U	K.HLVDEPQNLIKQNCDQFEK.L
5787	402	–	420	589.7889	2355.1265	2354.1325	0.9940	1	52	0.009	1Score **> 44** indicates **identity** Score **> 26** indicates **homology**	U	K.HLVDEPQNLIKQNCDQFEK.L
5789	402	–	420	1178.5719	2355.1292	2354.1325	0.9968	1	79	1.8e-05	1Score **> 44** indicates **identity** Score **> 24** indicates **homology**	U	K.HLVDEPQNLIKQNCDQFEK.L
6061	413	–	433	843.7262	2528.1568	2528.2118	-0.0550	1	49	0.016	2Score **> 44** indicates **identity**	U	K.QNCDQFEKLGEYGFQNALIVR.Y
6068	413	–	433	844.0744	2529.2014	2528.2118	0.9896	1	128	2e-10	1Score **> 44** indicates **identity** Score **> 36** indicates **homology**	U	K.QNCDQFEKLGEYGFQNALIVR.Y
6069	413	–	433	844.0745	2529.2017	2528.2118	0.9899	1	106	3.3e-08	1Score **> 44** indicates **identity**	U	K.QNCDQFEKLGEYGFQNALIVR.Y
6070	413	–	433	844.0745	2529.2017	2528.2118	0.9899	1	103	7.5e-08	1Score **> 44** indicates **identity** Score **> 36**	U	K.QNCDQFEKLGEYGFQNALIVR.Y

											indicates **homology**		
[6071](#)	413	–	433	844.0750	2529.2032	2528.2118	0.9914	1	107	2.8e-08	1Score **>** **44** indicates **identity** Score **>** **33** indicates **homology**	U	K.QNCDQFEKLGEYGFQNALIVR.Y
[6072](#)	413	–	433	1265.6089	2529.2032	2528.2118	0.9915	1	143	6.6e-12	1Score **>** **44** indicates **identity** Score **>** **35** indicates **homology**	U	K.QNCDQFEKLGEYGFQNALIVR.Y
[6073](#)	413	–	433	1265.6090	2529.2034	2528.2118	0.9917	1	127	2.8e-10	1Score **>** **44** indicates **identity** Score **>** **35** indicates **homology**	U	K.QNCDQFEKLGEYGFQNALIVR.Y
[6074](#)	413	–	433	633.3082	2529.2037	2528.2118	0.9919	1	50	0.014	1Score **>** **44** indicates **identity** Score **>** **36** indicates **homology**	U	K.QNCDQFEKLGEYGFQNALIVR.Y
[6075](#)	413	–	433	844.0753	2529.2041	2528.2118	0.9923	1	110	1.4e-08	1Score **>** **44** indicates **identity** Score **>** **37** indicates **homology**	U	K.QNCDQFEKLGEYGFQNALIVR.Y
[6076](#)	413	–	433	844.0754	2529.2044	2528.2118	0.9926	1	106	3.3e-08	1Score **>** **44** indicates **identity**	U	K.QNCDQFEKLGEYGFQNALIVR.Y
[6077](#)	413	–	433	844.0756	2529.2050	2528.2118	0.9932	1	102	8.5e-08	1Score **>** **44** indicates **identity** Score **>** **38** indicates **homology**	U	K.QNCDQFEKLGEYGFQNALIVR.Y
[6078](#)	413	–	433	1265.6100	2529.2054	2528.2118	0.9937	1	122	8.5e-10	1Score **>** **44** indicates **identity** Score **>** **41** indicates **homology**	U	K.QNCDQFEKLGEYGFQNALIVR.Y
[6079](#)	413	–	433	844.0760	2529.2062	2528.2118	0.9944	1	123	6.5e-10	1Score **>** **44** indicates **identity** Score **>** **41** indicates **homology**	U	K.QNCDQFEKLGEYGFQNALIVR.Y
[6080](#)	413	–	433	844.0760	2529.2062	2528.2118	0.9944	1	128	2.1e-10	1Score **>** **44** indicates **identity** Score **>** **43** indicates **homology**	U	K.QNCDQFEKLGEYGFQNALIVR.Y
											1Score **>** **44**		

☐6081	413	–	433	844.0760	2529.2062	2528.2118	0.9944	1	103	7.5e-08	indicates **identity** Score > **36** indicates **homology**	U	K.QNCDQFEKLGEYGFQNALIVR.Y
☐4085	421	–	433	1479.7863	1478.7790	1478.7881	-0.0091	0	80	2.7e-05	1Score > **46** indicates **identity** Score > **42** indicates **homology**		K.LGEYGFQNALIVR.Y
☐4086	421	–	433	740.3972	1478.7798	1478.7881	-0.0083	0	105	6.9e-08	1Score > **46** indicates **identity** Score > **39** indicates **homology**		K.LGEYGFQNALIVR.Y
☐4087	421	–	433	740.3972	1478.7798	1478.7881	-0.0083	0	108	3.4e-08	1Score > **46** indicates **identity** Score > **39** indicates **homology**		K.LGEYGFQNALIVR.Y
☐4088	421	–	433	740.3979	1478.7812	1478.7881	-0.0069	0	102	1.4e-07	1Score > **46** indicates **identity** Score > **38** indicates **homology**		K.LGEYGFQNALIVR.Y
☐4089	421	–	433	740.3981	1478.7816	1478.7881	-0.0065	0	109	3.3e-08	1Score > **46** indicates **identity** Score > **40** indicates **homology**		K.LGEYGFQNALIVR.Y
☐4090	421	–	433	740.3981	1478.7816	1478.7881	-0.0065	0	112	1.4e-08	1Score > **46** indicates **identity** Score > **39** indicates **homology**		K.LGEYGFQNALIVR.Y
☐4091	421	–	433	740.3983	1478.7820	1478.7881	-0.0061	0	112	1.7e-08	1Score > **46** indicates **identity** Score > **41** indicates **homology**		K.LGEYGFQNALIVR.Y
☐4092	421	–	433	740.3983	1478.7820	1478.7881	-0.0061	0	105	6.9e-08	1Score > **46** indicates **identity** Score > **40** indicates **homology**		K.LGEYGFQNALIVR.Y
☐4093	421	–	433	740.3985	1478.7824	1478.7881	-0.0057	0	108	3.8e-08	1Score > **46** indicates **identity** Score > **40** indicates **homology**		K.LGEYGFQNALIVR.Y
											1Score > **46** indicates		

4094	421 – 433	740.3985	1478.7824	1478.7881	-0.0057	0	111	1.7e-08	identity Score > **41** indicates **homology**	K.LGEYGFQNALIVR.Y
4095	421 – 433	740.3985	1478.7824	1478.7881	-0.0057	0	102	1.4e-07	1Score > **46** indicates **identity** Score > **38** indicates **homology**	K.LGEYGFQNALIVR.Y
4096	421 – 433	1479.7900	1478.7827	1478.7881	-0.0054	0	74	9.1e-05	1Score > **46** indicates **identity** Score > **35** indicates **homology**	K.LGEYGFQNALIVR.Y
4097	421 – 433	740.3990	1478.7834	1478.7881	-0.0047	0	90	2.3e-06	1Score > **46** indicates **identity** Score > **37** indicates **homology**	K.LGEYGFQNALIVR.Y
4098	421 – 433	740.3992	1478.7838	1478.7881	-0.0043	0	102	1.4e-07	1Score > **46** indicates **identity** Score > **39** indicates **homology**	K.LGEYGFQNALIVR.Y
4099	421 – 433	740.3992	1478.7838	1478.7881	-0.0043	0	109	3.1e-08	1Score > **46** indicates **identity** Score > **40** indicates **homology**	K.LGEYGFQNALIVR.Y
4100	421 – 433	740.3995	1478.7844	1478.7881	-0.0037	0	108	3.9e-08	1Score > **46** indicates **identity** Score > **40** indicates **homology**	K.LGEYGFQNALIVR.Y
4101	421 – 433	740.3995	1478.7844	1478.7881	-0.0037	0	106	6.4e-08	1Score > **46** indicates **identity** Score > **39** indicates **homology**	K.LGEYGFQNALIVR.Y
4102	421 – 433	740.3997	1478.7848	1478.7881	-0.0033	0	106	6.4e-08	1Score > **46** indicates **identity** Score > **39** indicates **homology**	K.LGEYGFQNALIVR.Y
4103	421 – 433	740.4001	1478.7856	1478.7881	-0.0025	0	82	1.6e-05	1Score > **46** indicates **identity** Score > **45** indicates **homology**	K.LGEYGFQNALIVR.Y
4104	421 – 433	740.4001	1478.7856	1478.7881	-0.0025	0	106	6.4e-08	1Score > **46** indicates **identity**	K.LGEYGFQNALIVR.Y

											Score > **39** indicates **homology**	
☐[4105](#)	421	–	433	740.4005	1478.7864	1478.7881	-0.0017	0	106	6.5e-08	1Score > **46** indicates **identity** Score > **37** indicates **homology**	K.LGEYGFQNALIVR.Y
☐[4106](#)	421	–	433	740.4006	1478.7866	1478.7881	-0.0015	0	92	1.6e-06	1Score > **46** indicates **identity** Score > **38** indicates **homology**	K.LGEYGFQNALIVR.Y
☐[4107](#)	421	–	433	740.4007	1478.7868	1478.7881	-0.0013	0	103	1.3e-07	1Score > **46** indicates **identity** Score > **38** indicates **homology**	K.LGEYGFQNALIVR.Y
☐[4108](#)	421	–	433	740.4011	1478.7876	1478.7881	-0.0005	0	85	7.7e-06	1Score > **46** indicates **identity** Score > **46** indicates **homology**	K.LGEYGFQNALIVR.Y
☐[4109](#)	421	–	433	740.4012	1478.7878	1478.7881	-0.0003	0	90	2.2e-06	1Score > **46** indicates **identity** Score > **37** indicates **homology**	K.LGEYGFQNALIVR.Y
☐[4110](#)	421	–	433	740.7166	1479.4186	1478.7881	0.6305	0	90	1.6e-06	1Score > **45** indicates **identity** Score > **37** indicates **homology**	K.LGEYGFQNALIVR.Y
☐[4111](#)	421	–	433	740.8925	1479.7704	1478.7881	0.9823	0	78	4.2e-05	1Score > **46** indicates **identity** Score > **36** indicates **homology**	K.LGEYGFQNALIVR.Y
☐[4112](#)	421	–	433	740.8944	1479.7742	1478.7881	0.9861	0	74	9.8e-05	1Score > **46** indicates **identity** Score > **43** indicates **homology**	K.LGEYGFQNALIVR.Y
☐[4113](#)	421	–	433	740.8987	1479.7828	1478.7881	0.9947	0	83	1.4e-05	1Score > **46** indicates **identity** Score > **39** indicates **homology**	K.LGEYGFQNALIVR.Y
☐[4114](#)	421	–	433	494.2688	1479.7846	1478.7881	0.9964	0	56	0.0064	1Score > **46** indicates **identity**	K.LGEYGFQNALIVR.Y

												Score > **43** indicates **homology**	
4115	421	–	433	740.8997	1479.7848	1478.7881	0.9967	0	87	4.7e-06	1	Score > **46** indicates **identity** Score > **41** indicates **homology**	K.LGEYGFQNALIVR.Y
4116	421	–	433	740.9005	1479.7864	1478.7881	0.9983	0	87	5.1e-06	1	Score > **46** indicates **identity** Score > **40** indicates **homology**	K.LGEYGFQNALIVR.Y
4117	421	–	433	740.9005	1479.7864	1478.7881	0.9983	0	81	1.8e-05	1	Score > **46** indicates **identity** Score > **45** indicates **homology**	K.LGEYGFQNALIVR.Y
4118	421	–	433	740.9011	1479.7876	1478.7881	0.9995	0	79	2.8e-05	1	Score > **46** indicates **identity** Score > **46** indicates **homology**	K.LGEYGFQNALIVR.Y
4119	421	–	433	740.9017	1479.7888	1478.7881	1.0007	0	79	2.8e-05	1	Score > **46** indicates **identity** Score > **37** indicates **homology**	K.LGEYGFQNALIVR.Y
4611	437	–	451	547.3131	1638.9175	1638.9305	-0.0130	1	88	3.9e-06	1	Score > **46** indicates **identity** Score > **30** indicates **homology**	R.KVPQVSTPTLVEVSR.S
4612	437	–	451	547.3142	1638.9208	1638.9305	-0.0097	1	84	8e-06	1	Score > **46** indicates **identity** Score > **31** indicates **homology**	R.KVPQVSTPTLVEVSR.S
4613	437	–	451	547.3143	1638.9211	1638.9305	-0.0094	1	78	3.7e-05	1	Score > **46** indicates **identity** Score > **35** indicates **homology**	R.KVPQVSTPTLVEVSR.S
4614	437	–	451	547.3143	1638.9211	1638.9305	-0.0094	1	76	5.5e-05	1	Score > **46** indicates **identity** Score > **36** indicates **homology**	R.KVPQVSTPTLVEVSR.S
4615	437	–	451	547.3144	1638.9214	1638.9305	-0.0091	1	94	8e-07	1	Score > **46** indicates **identity** Score >	R.KVPQVSTPTLVEVSR.S

										**33** indicates **homology**	
☐[4616]	437	– 451	547.3144	1638.9214	1638.9305	-0.0091	1	78	3.6e-05	1Score > **46** indicates **identity** Score > **30** indicates **homology**	R.KVPQVSTPTLVEVSR.S
☐[4617]	437	– 451	820.4681	1638.9216	1638.9305	-0.0088	1	125	6.9e-10	1Score > **46** indicates **identity** Score > **40** indicates **homology**	R.KVPQVSTPTLVEVSR.S
☐[4618]	437	– 451	820.4681	1638.9216	1638.9305	-0.0088	1	105	7.6e-08	1Score > **46** indicates **identity** Score > **35** indicates **homology**	R.KVPQVSTPTLVEVSR.S
☐[4619]	437	– 451	820.4681	1638.9216	1638.9305	-0.0088	1	125	6.8e-10	1Score > **46** indicates **identity** Score > **41** indicates **homology**	R.KVPQVSTPTLVEVSR.S
☐[4620]	437	– 451	547.3148	1638.9226	1638.9305	-0.0079	1	67	0.00044	1Score > **46** indicates **identity** Score > **34** indicates **homology**	R.KVPQVSTPTLVEVSR.S
☐[4621]	437	– 451	547.3148	1638.9226	1638.9305	-0.0079	1	74	9.2e-05	1Score > **46** indicates **identity** Score > **36** indicates **homology**	R.KVPQVSTPTLVEVSR.S
☐[4622]	437	– 451	547.3148	1638.9226	1638.9305	-0.0079	1	87	4.3e-06	1Score > **46** indicates **identity** Score > **37** indicates **homology**	R.KVPQVSTPTLVEVSR.S
☐[4623]	437	– 451	820.4686	1638.9226	1638.9305	-0.0078	1	125	6.8e-10	1Score > **46** indicates **identity** Score > **40** indicates **homology**	R.KVPQVSTPTLVEVSR.S
☐[4624]	437	– 451	547.3149	1638.9229	1638.9305	-0.0076	1	66	0.00057	1Score > **46** indicates **identity** Score > **36** indicates **homology**	R.KVPQVSTPTLVEVSR.S
☐[4625]	437	– 451	547.3149	1638.9229	1638.9305	-0.0076	1	85	6.6e-06	1Score > **46** indicates **identity** Score > **32**	R.KVPQVSTPTLVEVSR.S

										indicates **homology**	
4626	437	– 451	547.3149	1638.9229	1638.9305	-0.0076	1	89	2.6e-06	1Score **>** **46** indicates **identity** Score **>** **32** indicates **homology**	R.KVPQVSTPTLVEVSR.S
4627	437	– 451	547.3149	1638.9229	1638.9305	-0.0076	1	97	5e-07	1Score **>** **46** indicates **identity** Score **>** **32** indicates **homology**	R.KVPQVSTPTLVEVSR.S
4628	437	– 451	547.3149	1638.9229	1638.9305	-0.0076	1	92	1.5e-06	1Score **>** **46** indicates **identity** Score **>** **36** indicates **homology**	R.KVPQVSTPTLVEVSR.S
4629	437	– 451	820.4688	1638.9230	1638.9305	-0.0074	1	120	2.2e-09	1Score **>** **46** indicates **identity** Score **>** **40** indicates **homology**	R.KVPQVSTPTLVEVSR.S
4630	437	– 451	547.3150	1638.9232	1638.9305	-0.0073	1	89	2.7e-06	1Score **>** **46** indicates **identity** Score **>** **34** indicates **homology**	R.KVPQVSTPTLVEVSR.S
4631	437	– 451	547.3150	1638.9232	1638.9305	-0.0073	1	85	7.3e-06	1Score **>** **46** indicates **identity** Score **>** **32** indicates **homology**	R.KVPQVSTPTLVEVSR.S
4632	437	– 451	820.4689	1638.9232	1638.9305	-0.0072	1	120	2.2e-09	1Score **>** **46** indicates **identity** Score **>** **41** indicates **homology**	R.KVPQVSTPTLVEVSR.S
4633	437	– 451	820.4690	1638.9234	1638.9305	-0.0070	1	125	6.8e-10	1Score **>** **46** indicates **identity** Score **>** **41** indicates **homology**	R.KVPQVSTPTLVEVSR.S
4634	437	– 451	547.3151	1638.9235	1638.9305	-0.0070	1	86	5.7e-06	1Score **>** **46** indicates **identity** Score **>** **33** indicates **homology**	R.KVPQVSTPTLVEVSR.S
4635	437	– 451	547.3151	1638.9235	1638.9305	-0.0070	1	85	6.8e-06	1Score **>** **46** indicates **identity** Score **>** **31** indicates	R.KVPQVSTPTLVEVSR.S

										**homology**	
4636	437 – 451	547.3151	1638.9235	1638.9305	-0.0070	1	88	3.8e-06	1	Score > **46** indicates **identity** Score > **33** indicates **homology**	R.KVPQVSTPTLVEVSR.S
4637	437 – 451	820.4691	1638.9236	1638.9305	-0.0068	1	120	2.3e-09	1	Score > **46** indicates **identity** Score > **42** indicates **homology**	R.KVPQVSTPTLVEVSR.S
4638	437 – 451	547.3152	1638.9238	1638.9305	-0.0067	1	98	3.7e-07	1	Score > **46** indicates **identity** Score > **38** indicates **homology**	R.KVPQVSTPTLVEVSR.S
4639	437 – 451	547.3152	1638.9238	1638.9305	-0.0067	1	97	4.1e-07	1	Score > **46** indicates **identity** Score > **35** indicates **homology**	R.KVPQVSTPTLVEVSR.S
4640	437 – 451	547.3152	1638.9238	1638.9305	-0.0067	1	90	2.4e-06	1	Score > **46** indicates **identity** Score > **36** indicates **homology**	R.KVPQVSTPTLVEVSR.S
4641	437 – 451	547.3153	1638.9241	1638.9305	-0.0064	1	77	4.3e-05	1	Score > **46** indicates **identity** Score > **31** indicates **homology**	R.KVPQVSTPTLVEVSR.S
4642	437 – 451	547.3153	1638.9241	1638.9305	-0.0064	1	90	2.2e-06	1	Score > **46** indicates **identity** Score > **39** indicates **homology**	R.KVPQVSTPTLVEVSR.S
4643	437 – 451	547.3153	1638.9241	1638.9305	-0.0064	1	76	5.2e-05	1	Score > **46** indicates **identity** Score > **34** indicates **homology**	R.KVPQVSTPTLVEVSR.S
4644	437 – 451	547.3153	1638.9241	1638.9305	-0.0064	1	94	8.7e-07	1	Score > **46** indicates **identity** Score > **36** indicates **homology**	R.KVPQVSTPTLVEVSR.S
4645	437 – 451	547.3153	1638.9241	1638.9305	-0.0064	1	84	8.4e-06	1	Score > **46** indicates **identity** Score > **33** indicates **homology**	R.KVPQVSTPTLVEVSR.S

☐4646	437	– 451	547.3153	1638.9241	1638.9305	-0.0064	1	90	2.5e-06	1 Score **>** **46** indicates **identity** Score **>** **32** indicates **homology**	R.KVPQVSTPTLVEVSR.S
☐4647	437	– 451	820.4694	1638.9242	1638.9305	-0.0062	1	125	7.8e-10	1 Score **>** **46** indicates **identity** Score **>** **38** indicates **homology**	R.KVPQVSTPTLVEVSR.S
☐4648	437	– 451	820.4696	1638.9246	1638.9305	-0.0058	1	125	6.9e-10	1 Score **>** **46** indicates **identity** Score **>** **43** indicates **homology**	R.KVPQVSTPTLVEVSR.S
☐4649	437	– 451	547.3155	1638.9247	1638.9305	-0.0058	1	88	4e-06	1 Score **>** **46** indicates **identity** Score **>** **30** indicates **homology**	R.KVPQVSTPTLVEVSR.S
☐4650	437	– 451	820.4698	1638.9250	1638.9305	-0.0054	1	65	0.00072	1 Score **>** **46** indicates **identity**	R.KVPQVSTPTLVEVSR.S
☐4651	437	– 451	547.3157	1638.9253	1638.9305	-0.0052	1	89	2.8e-06	1 Score **>** **46** indicates **identity** Score **>** **41** indicates **homology**	R.KVPQVSTPTLVEVSR.S
☐4652	437	– 451	547.3160	1638.9262	1638.9305	-0.0043	1	89	2.8e-06	1 Score **>** **46** indicates **identity** Score **>** **38** indicates **homology**	R.KVPQVSTPTLVEVSR.S
☐4653	437	– 451	547.3162	1638.9268	1638.9305	-0.0037	1	87	4.8e-06	1 Score **>** **46** indicates **identity** Score **>** **38** indicates **homology**	R.KVPQVSTPTLVEVSR.S
☐4655	437	– 451	820.7706	1639.5266	1638.9305	0.5962	1	123	7.5e-10	1 Score **>** **45** indicates **identity** Score **>** **35** indicates **homology**	R.KVPQVSTPTLVEVSR.S
☐4656	437	– 451	820.9695	1639.9244	1638.9305	0.9940	1	104	9.4e-08	1 Score **>** **46** indicates **identity** Score **>** **42** indicates **homology**	R.KVPQVSTPTLVEVSR.S
☐4657	437	– 451	547.6488	1639.9246	1638.9305	0.9941	1	77	4.8e-05	1 Score **>** **46** indicates **identity**	R.KVPQVSTPTLVEVSR.S

											Score > **30** indicates **homology**	
4658	437	– 451	820.9699	1639.9252	1638.9305	0.9948	1	104	8.9e-08	1	Score > **46** indicates **identity** Score > **42** indicates **homology**	R.KVPQVSTPTLVEVSR.S
4659	437	– 451	547.6491	1639.9255	1638.9305	0.9950	1	98	3.9e-07	1	Score > **46** indicates **identity** Score > **33** indicates **homology**	R.KVPQVSTPTLVEVSR.S
4660	437	– 451	547.6492	1639.9258	1638.9305	0.9953	1	81	1.7e-05	1	Score > **46** indicates **identity** Score > **31** indicates **homology**	R.KVPQVSTPTLVEVSR.S
4661	437	– 451	820.9703	1639.9260	1638.9305	0.9956	1	104	8.8e-08	1	Score > **46** indicates **identity** Score > **44** indicates **homology**	R.KVPQVSTPTLVEVSR.S
4662	437	– 451	547.6494	1639.9264	1638.9305	0.9959	1	83	1.1e-05	1	Score > **46** indicates **identity** Score > **31** indicates **homology**	R.KVPQVSTPTLVEVSR.S
4663	437	– 451	547.6494	1639.9264	1638.9305	0.9959	1	85	7e-06	1	Score > **46** indicates **identity** Score > **30** indicates **homology**	R.KVPQVSTPTLVEVSR.S
4664	437	– 451	820.9708	1639.9270	1638.9305	0.9966	1	104	9.3e-08	1	Score > **46** indicates **identity** Score > **42** indicates **homology**	R.KVPQVSTPTLVEVSR.S
4665	437	– 451	820.9711	1639.9276	1638.9305	0.9972	1	104	1e-07	1	Score > **46** indicates **identity** Score > **41** indicates **homology**	R.KVPQVSTPTLVEVSR.S
4666	437	– 451	547.6499	1639.9279	1638.9305	0.9974	1	63	0.0012	1	Score > **46** indicates **identity** Score > **31** indicates **homology**	R.KVPQVSTPTLVEVSR.S
4667	437	– 451	547.6499	1639.9279	1638.9305	0.9974	1	96	6.1e-07	1	Score > **46** indicates **identity** Score >	R.KVPQVSTPTLVEVSR.S

										**31** indicates **homology**		
4209	438	– 451	504.6163	1510.8271	1510.8355	-0.0085	0	67	0.00047	1Score **>** **46** indicates **identity** Score **>** **41** indicates **homology**		K.VPQVSTPTLVEVSR.S
4214	438	– 451	756.9231	1511.8316	1510.8355	0.9961	0	88	3.8e-06	1Score **>** **46** indicates **identity** Score **>** **31** indicates **homology**		K.VPQVSTPTLVEVSR.S
6741	460	– 482	958.4371	2872.2895	2871.3023	0.9872	1	50	0.0097	1Score **>** **43** indicates **identity** Score **>** **23** indicates **homology**	U	R.CCTKPESERMPCTEDYLSLILNR.L
6743	460	– 482	719.0801	2872.2913	2871.3023	0.9890	1	49	0.013	1Score **>** **43** indicates **identity** Score **>** **34** indicates **homology**	U	R.CCTKPESERMPCTEDYLSLILNR.L
6744	460	– 482	1437.1538	2872.2930	2871.3023	0.9907	1	73	5.4e-05	1Score **>** **43** indicates **identity** Score **>** **26** indicates **homology**	U	R.CCTKPESERMPCTEDYLSLILNR.L
6745	460	– 482	958.4383	2872.2931	2871.3023	0.9908	1	53	0.0052	1Score **>** **43** indicates **identity** Score **>** **23** indicates **homology**	U	R.CCTKPESERMPCTEDYLSLILNR.L
6746	460	– 482	719.0806	2872.2933	2871.3023	0.9910	1	44	0.043	1Score **>** **43** indicates **identity** Score **>** **26** indicates **homology**	U	R.CCTKPESERMPCTEDYLSLILNR.L
6748	460	– 482	719.0808	2872.2941	2871.3023	0.9918	1	49	0.013	1Score **>** **43** indicates **identity** Score **>** **34** indicates **homology**	U	R.CCTKPESERMPCTEDYLSLILNR.L
6749	460	– 482	958.4387	2872.2943	2871.3023	0.9920	1	56	0.0026	1Score **>** **43** indicates **identity** Score **>** **22** indicates **homology**	U	R.CCTKPESERMPCTEDYLSLILNR.L
6751	460	– 482	958.4413	2872.3021	2871.3023	0.9998	1	58	0.0016	1Score **>** **43** indicates **identity** Score **>**	U	R.CCTKPESERMPCTEDYLSLILNR.L

											**24** indicates **homology**		
☐6777	460	–	482	963.7704	2888.2894	2887.2972	0.9921	1	50	0.012	1Score > **43** indicates **identity** Score > **22** indicates **homology**	U	R.CCTKPESERMPCTEDYLSLILNR.L + Oxidation (M)
☐6780	460	–	482	963.7715	2888.2927	2887.2972	0.9954	1	54	0.0043	1Score > **43** indicates **identity** Score > **27** indicates **homology**	U	R.CCTKPESERMPCTEDYLSLILNR.L + Oxidation (M)
☐4838	469	–	482	862.9150	1723.8154	1723.8273	-0.0119	0	95	6.9e-07	1Score > **46** indicates **identity** Score > **44** indicates **homology**		R.MPCTEDYLSLILNR.L
☐4840	469	–	482	575.6136	1723.8190	1723.8273	-0.0083	0	50	0.02	1Score > **46** indicates **identity** Score > **35** indicates **homology**		R.MPCTEDYLSLILNR.L
☐4841	469	–	482	862.9170	1723.8194	1723.8273	-0.0079	0	92	1.4e-06	1Score > **46** indicates **identity**		R.MPCTEDYLSLILNR.L
☐4842	469	–	482	862.9171	1723.8196	1723.8273	-0.0077	0	98	3.5e-07	1Score > **46** indicates **identity**		R.MPCTEDYLSLILNR.L
☐4843	469	–	482	862.9174	1723.8202	1723.8273	-0.0071	0	84	9.1e-06	1Score > **46** indicates **identity**		R.MPCTEDYLSLILNR.L
☐4844	469	–	482	575.6141	1723.8205	1723.8273	-0.0068	0	49	0.026	1Score > **46** indicates **identity** Score > **32** indicates **homology**		R.MPCTEDYLSLILNR.L
☐4845	469	–	482	862.9176	1723.8206	1723.8273	-0.0067	0	99	3e-07	1Score > **46** indicates **identity** Score > **45** indicates **homology**		R.MPCTEDYLSLILNR.L
☐4846	469	–	482	862.9180	1723.8214	1723.8273	-0.0059	0	92	1.4e-06	1Score > **46** indicates **identity**		R.MPCTEDYLSLILNR.L
☐4847	469	–	482	862.9180	1723.8214	1723.8273	-0.0059	0	84	8.2e-06	1Score > **46** indicates **identity** Score > **44** indicates **homology**		R.MPCTEDYLSLILNR.L
☐4848	469	–	482	862.9181	1723.8216	1723.8273	-0.0057	0	99	3e-07	1Score > **46** indicates **identity**		R.MPCTEDYLSLILNR.L
											1Score >		

4849	469 – 482	862.9182	1723.8218	1723.8273	-0.0055	0	86	5e-06	**46** indicates **identity**	R.MPCTEDYLSLILNR.L
4850	469 – 482	862.9182	1723.8218	1723.8273	-0.0055	0	78	3.6e-05	1Score > **46** indicates **identity** Score > **44** indicates **homology**	R.MPCTEDYLSLILNR.L
4851	469 – 482	862.9191	1723.8236	1723.8273	-0.0037	0	73	0.00012	1Score > **46** indicates **identity** Score > **42** indicates **homology**	R.MPCTEDYLSLILNR.L
4857	469 – 482	863.4149	1724.8152	1723.8273	0.9879	0	78	3.5e-05	1Score > **46** indicates **identity** Score > **35** indicates **homology**	R.MPCTEDYLSLILNR.L
4861	469 – 482	863.4186	1724.8226	1723.8273	0.9953	0	80	1.9e-05	1Score > **46** indicates **identity** Score > **37** indicates **homology**	R.MPCTEDYLSLILNR.L
4883	469 – 482	580.9448	1739.8126	1739.8222	-0.0096	0	51	0.016	1Score > **46** indicates **identity** Score > **40** indicates **homology**	R.MPCTEDYLSLILNR.L + Oxidation (M)
4291	483 – 495	770.4079	1538.8012	1538.8127	-0.0114	1	66	0.00065	1Score > **46** indicates **identity** Score > **35** indicates **homology**	R.LCVLHEKTPVSEK.V
4292	483 – 495	513.9421	1538.8045	1538.8127	-0.0082	1	63	0.0012	1Score > **46** indicates **identity** Score > **35** indicates **homology**	R.LCVLHEKTPVSEK.V
4295	483 – 495	513.9423	1538.8051	1538.8127	-0.0076	1	63	0.0012	1Score > **46** indicates **identity** Score > **35** indicates **homology**	R.LCVLHEKTPVSEK.V
4296	483 – 495	513.9423	1538.8051	1538.8127	-0.0076	1	58	0.0034	1Score > **46** indicates **identity** Score > **33** indicates **homology**	R.LCVLHEKTPVSEK.V
4297	483 – 495	770.4099	1538.8052	1538.8127	-0.0074	1	71	0.00019	1Score > **46** indicates **identity** Score > **32**	R.LCVLHEKTPVSEK.V

											indicates **homology**		
☐[4299](#)	483	–	495	513.9424	1538.8054	1538.8127	-0.0073	1	63	0.0013	1 Score > **46** indicates **identity** Score > **30** indicates **homology**		R.LCVLHEKTPVSEK.V
☐[4300](#)	483	–	495	770.4100	1538.8054	1538.8127	-0.0072	1	51	0.021	1 Score > **46** indicates **identity** Score > **27** indicates **homology**		R.LCVLHEKTPVSEK.V
☐[4301](#)	483	–	495	770.4100	1538.8054	1538.8127	-0.0072	1	71	0.00017	1 Score > **46** indicates **identity** Score > **36** indicates **homology**		R.LCVLHEKTPVSEK.V
☐[4304](#)	483	–	495	385.7087	1538.8057	1538.8127	-0.0070	1	50	0.022	1 Score > **46** indicates **identity** Score > **34** indicates **homology**		R.LCVLHEKTPVSEK.V
☐[4305](#)	483	–	495	513.9426	1538.8060	1538.8127	-0.0067	1	62	0.0014	1 Score > **46** indicates **identity** Score > **34** indicates **homology**		R.LCVLHEKTPVSEK.V
☐[4306](#)	483	–	495	770.4106	1538.8066	1538.8127	-0.0060	1	71	0.0002	1 Score > **46** indicates **identity** Score > **37** indicates **homology**		R.LCVLHEKTPVSEK.V
☐[4307](#)	483	–	495	770.4116	1538.8086	1538.8127	-0.0040	1	59	0.003	1 Score > **46** indicates **identity** Score > **32** indicates **homology**		R.LCVLHEKTPVSEK.V
☐[4309](#)	483	–	495	770.9101	1539.8056	1538.8127	0.9930	1	54	0.0088	1 Score > **46** indicates **identity** Score > **33** indicates **homology**		R.LCVLHEKTPVSEK.V
☐[4311](#)	483	–	495	514.2764	1539.8074	1538.8127	0.9947	1	62	0.0015	1 Score > **46** indicates **identity** Score > **28** indicates **homology**		R.LCVLHEKTPVSEK.V
☐[5899](#)	508	–	528	618.5505	2470.1729	2470.1839	-0.0110	1	48	0.022	1 Score > **44** indicates **identity** Score > **23** indicates	U	R.RPCFSALTPDETYVPKAFDEK.L

											homology	
□5904	508	– 528	824.7322	2471.1748	2470.1839	0.9909	1	46	0.037	1	Score > **44** indicates **identity** Score > **24** indicates **homology**	U R.RPCFSALTPDETYVPKAFDEK.L
□5906	508	– 528	824.7336	2471.1790	2470.1839	0.9951	1	46	0.033	1	Score > **44** indicates **identity** Score > **23** indicates **homology**	U R.RPCFSALTPDETYVPKAFDEK.L
□5907	508	– 528	1236.6000	2471.1854	2470.1839	1.0016	1	45	0.048	1	Score > **44** indicates **identity** Score > **22** indicates **homology**	U R.RPCFSALTPDETYVPKAFDEK.L
□5977	524	– 544	833.3958	2497.1656	2497.1835	-0.0180	1	74	5.1e-05	1	Score > **44** indicates **identity** Score > **25** indicates **homology**	U K.AFDEKLFTFHADICTLPDTEK.Q
□5978	524	– 544	1249.5914	2497.1682	2497.1835	-0.0153	1	76	3.9e-05	1	Score > **44** indicates **identity** Score > **25** indicates **homology**	U K.AFDEKLFTFHADICTLPDTEK.Q
□5981	524	– 544	833.3978	2497.1716	2497.1835	-0.0120	1	46	0.032	1	Score > **44** indicates **identity** Score > **28** indicates **homology**	U K.AFDEKLFTFHADICTLPDTEK.Q
□5982	524	– 544	833.3986	2497.1740	2497.1835	-0.0096	1	50	0.013	1	Score > **44** indicates **identity** Score > **25** indicates **homology**	U K.AFDEKLFTFHADICTLPDTEK.Q
□5983	524	– 544	833.3994	2497.1764	2497.1835	-0.0072	1	76	3.5e-05	1	Score > **44** indicates **identity** Score > **25** indicates **homology**	U K.AFDEKLFTFHADICTLPDTEK.Q
□5984	524	– 544	833.3999	2497.1779	2497.1835	-0.0057	1	79	1.7e-05	1	Score > **44** indicates **identity** Score > **25** indicates **homology**	U K.AFDEKLFTFHADICTLPDTEK.Q
□5986	524	– 544	833.4026	2497.1860	2497.1835	0.0024	1	52	0.008	1	Score > **44** indicates **identity** Score > **27** indicates **homology**	U K.AFDEKLFTFHADICTLPDTEK.Q

5999	524	– 544	625.5507	2498.1737	2497.1835	0.9902	1	48	0.02	1Score **> 44** indicates **identity** Score **> 32** indicates **homology**	U	K.AFDEKLFTFHADICTLPDTEK.Q
6000	524	– 544	833.7321	2498.1745	2497.1835	0.9909	1	68	0.00023	1Score **> 44** indicates **identity** Score **> 21** indicates **homology**	U	K.AFDEKLFTFHADICTLPDTEK.Q
6002	524	– 544	1250.0953	2498.1760	2497.1835	0.9925	1	77	3.1e-05	1Score **> 44** indicates **identity** Score **> 27** indicates **homology**	U	K.AFDEKLFTFHADICTLPDTEK.Q
6003	524	– 544	625.5513	2498.1761	2497.1835	0.9926	1	47	0.029	1Score **> 44** indicates **identity** Score **> 26** indicates **homology**	U	K.AFDEKLFTFHADICTLPDTEK.Q
6004	524	– 544	625.5514	2498.1765	2497.1835	0.9930	1	45	0.044	1Score **> 44** indicates **identity** Score **> 25** indicates **homology**	U	K.AFDEKLFTFHADICTLPDTEK.Q
6005	524	– 544	833.7330	2498.1772	2497.1835	0.9936	1	76	3.4e-05	1Score **> 44** indicates **identity** Score **> 26** indicates **homology**	U	K.AFDEKLFTFHADICTLPDTEK.Q
6011	524	– 544	625.5526	2498.1813	2497.1835	0.9978	1	51	0.011	1Score **> 44** indicates **identity** Score **> 25** indicates **homology**	U	K.AFDEKLFTFHADICTLPDTEK.Q
5214	529	– 544	636.6421	1906.9045	1906.9135	-0.0090	0	46	0.049	1Score **> 45** indicates **identity** Score **> 31** indicates **homology**	U	K.LFTFHADICTLPDTEK.Q
5215	529	– 544	636.6423	1906.9051	1906.9135	-0.0084	0	52	0.013	1Score **> 45** indicates **identity** Score **> 29** indicates **homology**	U	K.LFTFHADICTLPDTEK.Q
5216	529	– 544	636.6429	1906.9069	1906.9135	-0.0066	0	54	0.0072	1Score **> 45** indicates **identity** Score **> 27** indicates **homology**	U	K.LFTFHADICTLPDTEK.Q

1Score **>**

5217	529	–	544	954.9598	1907.9050	1906.9135	0.9915	0	76	4.7e-05	46 indicates **identity** Score > **34** indicates **homology**	U	K.LFTFHADICTLPDTEK.Q
5219	529	–	544	636.9761	1907.9065	1906.9135	0.9930	0	52	0.014	1Score > **46** indicates **identity** Score > **33** indicates **homology**	U	K.LFTFHADICTLPDTEK.Q
5220	529	–	544	636.9765	1907.9077	1906.9135	0.9942	0	54	0.0078	1Score > **46** indicates **identity** Score > **31** indicates **homology**	U	K.LFTFHADICTLPDTEK.Q
5221	529	–	544	636.9774	1907.9104	1906.9135	0.9969	0	47	0.039	1Score > **46** indicates **identity** Score > **37** indicates **homology**	U	K.LFTFHADICTLPDTEK.Q
5222	529	–	544	954.9625	1907.9104	1906.9135	0.9969	0	63	0.00099	1Score > **46** indicates **identity** Score > **35** indicates **homology**	U	K.LFTFHADICTLPDTEK.Q
5697	529	–	547	759.7164	2276.1274	2276.1511	-0.0237	1	49	0.022	1Score > **45** indicates **identity** Score > **30** indicates **homology**	U	K.LFTFHADICTLPDTEKQIK.K
5708	529	–	547	1139.5789	2277.1432	2276.1511	0.9921	1	75	4.5e-05	1Score > **44** indicates **identity** Score > **36** indicates **homology**	U	K.LFTFHADICTLPDTEKQIK.K
5711	529	–	547	1139.5809	2277.1472	2276.1511	0.9961	1	79	2.2e-05	1Score > **44** indicates **identity** Score > **33** indicates **homology**	U	K.LFTFHADICTLPDTEKQIK.K
3106	548	–	557	571.8574	1141.7002	1141.7070	-0.0068	1	62	0.0018	1Score > **47** indicates **identity**		K.KQTALVELLK.H
3108	548	–	557	571.8578	1141.7010	1141.7070	-0.0060	1	66	0.00066	1Score > **47** indicates **identity**		K.KQTALVELLK.H
3109	548	–	557	571.8579	1141.7012	1141.7070	-0.0058	1	70	0.00028	1Score > **47** indicates **identity**		K.KQTALVELLK.H
3110	548	–	557	571.8579	1141.7012	1141.7070	-0.0058	1	70	0.00027	1Score > **47** indicates **identity**		K.KQTALVELLK.H

1Score >

3113	548	– 557	571.8580	1141.7014	1141.7070	-0.0056	1	70	0.00028	47 indicates **identity**	K.KQTALVELLK.H
3114	548	– 557	571.8582	1141.7018	1141.7070	-0.0052	1	70	0.00028	1Score > **47** indicates **identity**	K.KQTALVELLK.H
3115	548	– 557	571.8582	1141.7018	1141.7070	-0.0052	1	67	0.00055	1Score > **47** indicates **identity**	K.KQTALVELLK.H
3116	548	– 557	571.8582	1141.7018	1141.7070	-0.0052	1	64	0.0011	1Score > **47** indicates **identity** Score > **46** indicates **homology**	K.KQTALVELLK.H
3117	548	– 557	571.8582	1141.7018	1141.7070	-0.0052	1	50	0.03	1Score > **47** indicates **identity** Score > **39** indicates **homology**	K.KQTALVELLK.H
3121	548	– 557	571.8583	1141.7020	1141.7070	-0.0050	1	70	0.00027	1Score > **47** indicates **identity**	K.KQTALVELLK.H
3122	548	– 557	571.8583	1141.7020	1141.7070	-0.0050	1	67	0.00054	1Score > **47** indicates **identity**	K.KQTALVELLK.H
3123	548	– 557	571.8584	1141.7022	1141.7070	-0.0048	1	67	0.00052	1Score > **47** indicates **identity**	K.KQTALVELLK.H
3127	548	– 557	571.8585	1141.7024	1141.7070	-0.0046	1	70	0.00027	1Score > **47** indicates **identity**	K.KQTALVELLK.H
3128	548	– 557	571.8585	1141.7024	1141.7070	-0.0046	1	66	0.00066	1Score > **47** indicates **identity** Score > **47** indicates **homology**	K.KQTALVELLK.H
3129	548	– 557	571.8585	1141.7024	1141.7070	-0.0046	1	67	0.00062	1Score > **47** indicates **identity**	K.KQTALVELLK.H
3133	548	– 557	571.8588	1141.7030	1141.7070	-0.0040	1	67	0.00063	1Score > **47** indicates **identity**	K.KQTALVELLK.H
3135	548	– 557	571.8589	1141.7032	1141.7070	-0.0038	1	51	0.022	1Score > **47** indicates **identity** Score > **37** indicates **homology**	K.KQTALVELLK.H
3139	548	– 557	571.8591	1141.7036	1141.7070	-0.0034	1	65	0.00093	1Score > **47** indicates **identity** Score > **46** indicates **homology**	K.KQTALVELLK.H
3140	548	– 557	571.8591	1141.7036	1141.7070	-0.0034	1	56	0.0066	1Score > **47** indicates **identity**	K.KQTALVELLK.H
										1Score >	

☐3141	548	–	557	571.8593	1141.7040	1141.7070	-0.0030	1	70	0.00027	47 indicates **identity**		K.KQTALVELLK.H
☐2923	549	–	557	1014.6146	1013.6073	1013.6121	-0.0048	0	55	0.011	1Score > **48** indicates **identity**		K.QTALVELLK.H
☐2938	549	–	557	1014.6152	1013.6079	1013.6121	-0.0042	0	60	0.0029	1Score > **48** indicates **identity**		K.QTALVELLK.H
☐2949	549	–	557	1014.6168	1013.6095	1013.6121	-0.0026	0	57	0.0063	1Score > **48** indicates **identity**		K.QTALVELLK.H
☐2950	549	–	557	1014.6179	1013.6106	1013.6121	-0.0015	0	51	0.027	1Score > **48** indicates **identity** Score > **45** indicates **homology**		K.QTALVELLK.H
☐5618	562	–	580	1100.0486	2198.0826	2198.0929	-0.0103	1	88	2.8e-06	1Score > **45** indicates **identity** Score > **29** indicates **homology**	U	K.ATEEQLKTVMENFVAFVDK.C
☐5619	562	–	580	1100.0490	2198.0834	2198.0929	-0.0095	1	102	9.5e-08	1Score > **45** indicates **identity** Score > **30** indicates **homology**	U	K.ATEEQLKTVMENFVAFVDK.C
☐5620	562	–	580	733.7052	2198.0938	2198.0929	0.0009	1	85	5.7e-06	1Score > **45** indicates **identity** Score > **26** indicates **homology**	U	K.ATEEQLKTVMENFVAFVDK.C
☐5624	562	–	580	1100.5499	2199.0852	2198.0929	0.9923	1	88	2.8e-06	1Score > **45** indicates **identity** Score > **29** indicates **homology**	U	K.ATEEQLKTVMENFVAFVDK.C
☐5626	562	–	580	734.0358	2199.0856	2198.0929	0.9927	1	57	0.0037	1Score > **45** indicates **identity**	U	K.ATEEQLKTVMENFVAFVDK.C
☐5627	562	–	580	734.0358	2199.0856	2198.0929	0.9927	1	89	1.9e-06	1Score > **45** indicates **identity**	U	K.ATEEQLKTVMENFVAFVDK.C
☐5628	562	–	580	734.0372	2199.0898	2198.0929	0.9969	1	64	0.00068	1Score > **45** indicates **identity**	U	K.ATEEQLKTVMENFVAFVDK.C
☐5629	562	–	580	734.0372	2199.0898	2198.0929	0.9969	1	71	0.00014	1Score > **45** indicates **identity**	U	K.ATEEQLKTVMENFVAFVDK.C
☐5631	562	–	580	734.0389	2199.0949	2198.0929	1.0020	1	63	0.00085	1Score > **45** indicates **identity**	U	K.ATEEQLKTVMENFVAFVDK.C
☐3694	569	–	580	700.3462	1398.6778	1398.6853	-0.0075	0	74	0.00011	1Score > **47** indicates **identity** Score >		K.TVMENFVAFVDK.C

										**36** indicates **homology**		
☐3695	569	– 580	700.3462	1398.6778	1398.6853	-0.0075	0	79	3.7e-05	1Score **>** **47** indicates **identity** Score **>** **37** indicates **homology**		K.TVMENFVAFVDK.C
☐3696	569	– 580	700.3464	1398.6782	1398.6853	-0.0071	0	93	1.5e-06	1Score **>** **47** indicates **identity** Score **>** **46** indicates **homology**		K.TVMENFVAFVDK.C
☐3698	569	– 580	700.3479	1398.6812	1398.6853	-0.0041	0	79	3.7e-05	1Score **>** **47** indicates **identity** Score **>** **37** indicates **homology**		K.TVMENFVAFVDK.C
☐3699	569	– 580	700.3506	1398.6866	1398.6853	0.0013	0	69	0.00032	1Score **>** **47** indicates **identity** Score **>** **34** indicates **homology**		K.TVMENFVAFVDK.C
☐3702	569	– 580	700.8475	1399.6804	1398.6853	0.9951	0	61	0.0023	1Score **>** **47** indicates **identity** Score **>** **37** indicates **homology**		K.TVMENFVAFVDK.C
☐3703	569	– 580	700.8491	1399.6836	1398.6853	0.9983	0	62	0.0018	1Score **>** **47** indicates **identity** Score **>** **34** indicates **homology**		K.TVMENFVAFVDK.C
☐3704	569	– 580	700.8500	1399.6854	1398.6853	1.0001	0	60	0.0025	1Score **>** **47** indicates **identity** Score **>** **35** indicates **homology**		K.TVMENFVAFVDK.C
☐3705	569	– 580	700.8506	1399.6866	1398.6853	1.0013	0	61	0.002	1Score **>** **47** indicates **identity** Score **>** **34** indicates **homology**		K.TVMENFVAFVDK.C
☐5247	581	– 597	964.3969	1926.7792	1926.7910	-0.0118	1	136	4.6e-11	1Score **>** **45** indicates **identity** Score **>** **32** indicates **homology**	U	K.CCAADDKEACFAVEGPK.L
☐5248	581	– 597	964.3976	1926.7806	1926.7910	-0.0104	1	135	4.9e-11	1Score **>** **45** indicates **identity** Score **>**	U	K.CCAADDKEACFAVEGPK.L

												**30** indicates **homology**		
☐[5249](#)	581	–	597	964.3984	1926.7822	1926.7910	-0.0088	1	125	5e-10	1	Score > **45** indicates **identity** Score > **31** indicates **homology**	U	K.CCAADDKEACFAVEGPK.L
☐[5250](#)	581	–	597	643.2681	1926.7825	1926.7910	-0.0085	1	80	1.7e-05	1	Score > **45** indicates **identity** Score > **29** indicates **homology**	U	K.CCAADDKEACFAVEGPK.L
☐[5251](#)	581	–	597	964.3987	1926.7828	1926.7910	-0.0082	1	144	7.1e-12	1	Score > **45** indicates **identity** Score > **32** indicates **homology**	U	K.CCAADDKEACFAVEGPK.L
☐[5252](#)	581	–	597	643.6022	1927.7848	1926.7910	0.9938	1	50	0.015	1	Score > **45** indicates **identity** Score > **24** indicates **homology**	U	K.CCAADDKEACFAVEGPK.L
☐[5253](#)	581	–	597	643.6035	1927.7887	1926.7910	0.9977	1	78	2.8e-05	1	Score > **45** indicates **identity** Score > **26** indicates **homology**	U	K.CCAADDKEACFAVEGPK.L

Error distribution	Error distribution (ppm)

```
ID   ALBU_BOVIN              Reviewed;         607 AA.
AC   P02769; A5PJX3; O02787; P04277; Q3SZR2;
DT   21-JUL-1986, integrated into UniProtKB/Swiss-Prot.
DT   01-FEB-1996, sequence version 4.
DT   23-FEB-2022, entry version 186.
DE   RecName: Full=Albumin;
DE   AltName: Full=BSA;
DE   AltName: Allergen=Bos d 6;
DE   Flags: Precursor;
GN   Name=ALB;
OS   Bos taurus (Bovine).
OC   Eukaryota; Metazoa; Chordata; Craniata; Vertebrata; Euteleostomi; Mammalia;
OC   Eutheria; Laurasiatheria; Artiodactyla; Ruminantia; Pecora; Bovidae;
OC   Bovinae; Bos.
OX   NCBI_TaxID=9913;
RN   [1]
RP   NUCLEOTIDE SEQUENCE [MRNA].
RA   Holowachuk E.W., Stoltenborg J.K., Reed R.G., Peters T. Jr.;
RT   "Bovine serum albumin: cDNA sequence and expression.";
RL   Submitted (AUG-1991) to the EMBL/GenBank/DDBJ databases.
RN   [2]
RP   NUCLEOTIDE SEQUENCE [MRNA], AND VARIANT THR-214.
RC   TISSUE=Liver;
RA   Barry T., Power S., Gannon F.;
RT   "The bovine serum albumin mRNA.";
RL   Submitted (JUL-1994) to the EMBL/GenBank/DDBJ databases.
RN   [3]
RP   NUCLEOTIDE SEQUENCE [MRNA].
RC   TISSUE=Liver;
RX   PubMed=11298124; DOI=10.1046/j.1365-2249.2001.01451.x;
RA   Hilger C., Grigioni F., De Beaufort C., Michel G., Freilinger J.,
RA   Hentges F.;
RT   "Differential binding of IgG and IgA antibodies to antigenic determinants
RT   of bovine serum albumin.";
```

```
RL   Clin. Exp. Immunol. 123:387-394(2001).
RN   [4]
RP   NUCLEOTIDE SEQUENCE [MRNA], AND VARIANT THR-214.
RA   Wu H.T., Huang M.C.;
RT   "The complete cDNA sequence of bovine serum albumin.";
RL   Submitted (AUG-2002) to the EMBL/GenBank/DDBJ databases.
RN   [5]
RP   NUCLEOTIDE SEQUENCE [LARGE SCALE MRNA], AND VARIANT THR-214.
RC   STRAIN=Hereford; TISSUE=Fetal liver, and Testis;
RG   NIH - Mammalian Gene Collection (MGC) project;
RL   Submitted (JUN-2007) to the EMBL/GenBank/DDBJ databases.
RN   [6]
RP   PROTEIN SEQUENCE OF 1-32 (PRECURSOR PROTEIN).
RX   PubMed=488109; DOI=10.1111/j.1432-1033.1979.tb13209.x;
RA   McGillivray R.T.A., Chung D.W., Davie E.W.;
RT   "Biosynthesis of bovine plasma proteins in a cell-free system. Amino-
RT   terminal sequence of preproalbumin.";
RL   Eur. J. Biochem. 98:477-485(1979).
RN   [7]
RP   PROTEIN SEQUENCE OF 19-28.
RX   PubMed=843354; DOI=10.1016/0006-291x(77)91648-5;
RA   Patterson J.E., Geller D.M.;
RT   "Bovine microsomal albumin: amino terminal sequence of bovine proalbumin.";
RL   Biochem. Biophys. Res. Commun. 74:1220-1226(1977).
RN   [8]
RP   PARTIAL PROTEIN SEQUENCE, AND SEQUENCE REVISION TO 118-119 AND 180.
RX   PubMed=2260975; DOI=10.1016/s0006-291x(05)80083-x;
RA   Hirayama K., Akashi S., Furuya M., Fukuhara K.;
RT   "Rapid confirmation and revision of the primary structure of bovine serum
RT   albumin by ESIMS and Frit-FAB LC/MS.";
RL   Biochem. Biophys. Res. Commun. 173:639-646(1990).
RN   [9]
RP   PROTEIN SEQUENCE OF 25-424 AND 429-607, AND VARIANT THR-214.
RA   Brown J.R.;
RT   "Structure of bovine serum albumin.";
RL   Fed. Proc. 34:591-591(1975).
RN   [10]
RP   SEQUENCE REVISION TO 190-195.
RA   Brown J.R.;
RL   Submitted (APR-1975) to the PIR data bank.
RN   [11]
RP   PROTEIN SEQUENCE OF 25-64.
RX   PubMed=2379503; DOI=10.1111/j.1432-1033.1990.tb19092.x;
RA   Strawich E., Glimcher M.J.;
RT   "Tooth 'enamelins' identified mainly as serum proteins. Major 'enamelin' is
RT   albumin.";
RL   Eur. J. Biochem. 191:47-56(1990).
RN   [12]
RP   PROTEIN SEQUENCE OF 25-41.
RX   PubMed=3389500; DOI=10.1016/0003-2697(88)90082-6;
RA   Hsieh J.C., Lin F.P., Tam M.F.;
RT   "Electroblotting onto glass-fiber filter from an analytical
RT   isoelectrofocusing gel: a preparative method for isolating proteins for N-
RT   terminal microsequencing.";
RL   Anal. Biochem. 170:1-8(1988).
RN   [13]
RP   PROTEIN SEQUENCE OF 163-172.
RX   PubMed=2474609;
RA   Carraway R.E., Cochrane D.E., Boucher W., Mitra S.P.;
RT   "Structures of histamine-releasing peptides formed by the action of acid
RT   proteases on mammalian albumin(s).";
RL   J. Immunol. 143:1680-1684(1989).
RN   [14]
RP   PROTEIN SEQUENCE OF 165-173.
RC   TISSUE=Plasma;
RX   PubMed=2437111;
RA   Carraway R.E., Mitra S.P., Cochrane D.E.;
RT   "Structure of a biologically active neurotensin-related peptide obtained
RT   from pepsin-treated albumin(s).";
RL   J. Biol. Chem. 262:5968-5973(1987).
RN   [15]
RP   PROTEIN SEQUENCE OF 402-433.
RX   PubMed=7283978; DOI=10.1042/bj1910867;
RA   Reed R.G., Putnam F.W., Peters T. Jr.;
RT   "Sequence of residues 400-403 of bovine serum albumin.";
RL   Biochem. J. 191:867-868(1980).
RN   [16]
RP   PROTEIN SEQUENCE OF 437-451.
RA   Vilbois F.;
RL   Submitted (AUG-1998) to UniProtKB.
RN   [17]
RP   DISULFIDE BONDS.
RA   Brown J.R.;
RT   "Structure of serum albumin: disulfide bridges.";
RL   Fed. Proc. 33:1389-1389(1974).
RN   [18]
RP   FUNCTION.
RX   PubMed=6234017; DOI=10.1021/bi00305a003;
RA   Konopka K., Neilands J.B.;
RT   "Effect of serum albumin on siderophore-mediated utilization of transferrin
RT   iron.";
RL   Biochemistry 23:2122-2127(1984).
RN   [19] {ECO:0007744|PDB:2L7U}
RP   STRUCTURE BY NMR OF 148-154, AND GLYCATION AT LYS-151.
RX   PubMed=21565706; DOI=10.1016/j.str.2011.02.013;
RA   Xue J., Rai V., Singer D., Chabierski S., Xie J., Reverdatto S., Burz D.S.,
RA   Schmidt A.M., Hoffmann R., Shekhtman A.;
```

```
RT   "Advanced glycation end product recognition by the receptor for AGEs.";
RL   Structure 19:722-732(2011).
RN   [20] {ECO:0007744|PDB:4F5S}
RP   X-RAY CRYSTALLOGRAPHY (2.47 ANGSTROMS) OF 25-607, DISULFIDE BONDS, AND
RP   VARIANT THR-214.
RX   PubMed=22993082; DOI=10.1107/s0907444912027047;
RA   Bujacz A.;
RT   "Structures of bovine, equine and leporine serum albumin.";
RL   Acta Crystallogr. D 68:1278-1289(2012).
RN   [21] {ECO:0007744|PDB:3V03}
RP   X-RAY CRYSTALLOGRAPHY (2.70 ANGSTROMS) OF 25-607 IN COMPLEX WITH CALCIUM,
RP   FUNCTION, AND DISULFIDE BONDS.
RX   PubMed=22677715; DOI=10.1016/j.molimm.2012.05.011;
RA   Majorek K.A., Porebski P.J., Dayal A., Zimmerman M.D., Jablonska K.,
RA   Stewart A.J., Chruszcz M., Minor W.;
RT   "Structural and immunologic characterization of bovine, horse, and rabbit
RT   serum albumins.";
RL   Mol. Immunol. 52:174-182(2012).
RN   [22] {ECO:0007744|PDB:4JK4}
RP   X-RAY CRYSTALLOGRAPHY (2.65 ANGSTROMS) OF 25-607 IN COMPLEX WITH CALCIUM,
RP   AND DISULFIDE BONDS.
RX   PubMed=23769932; DOI=10.1016/j.ijbiomac.2013.06.004;
RA   Sekula B., Zielinski K., Bujacz A.;
RT   "Crystallographic studies of the complexes of bovine and equine serum
RT   albumin with 3,5-diiodosalicylic acid.";
RL   Int. J. Biol. Macromol. 60:316-324(2013).
RN   [23] {ECO:0007744|PDB:4OR0}
RP   X-RAY CRYSTALLOGRAPHY (2.58 ANGSTROMS) OF 25-607, AND DISULFIDE BONDS.
RX   PubMed=24753230; DOI=10.1002/prot.24583;
RA   Bujacz A., Zielinski K., Sekula B.;
RT   "Structural studies of bovine, equine, and leporine serum albumin complexes
RT   with naproxen.";
RL   Proteins 82:2199-2208(2014).
CC   -!- FUNCTION: Binds water, Ca(2+), Na(+), K(+), fatty acids, hormones,
CC       bilirubin and drugs. Its main function is the regulation of the
CC       colloidal osmotic pressure of blood. Major zinc transporter in plasma,
CC       typically binds about 80% of all plasma zinc (By similarity). Major
CC       calcium and magnesium transporter in plasma, binds approximately 45% of
CC       circulating calcium and magnesium in plasma (Probable). Potentially has
CC       more than two calcium-binding sites and might additionally bind calcium
CC       in a non-specific manner (PubMed:22677715). The shared binding site
CC       between zinc and calcium at residue Asp-272 suggests a crosstalk
CC       between zinc and calcium transport in the blood (Probable). The rank
CC       order of affinity is zinc > calcium > magnesium (Probable). Binds to
CC       the bacterial siderophore enterobactin and inhibits enterobactin-
CC       mediated iron uptake of E.coli, and may thereby limit the utilization
CC       of iron and growth of enteric bacteria such as E.coli (PubMed:6234017).
CC       Does not prevent iron uptake by the bacterial siderophore aerobactin
CC       (PubMed:6234017). {ECO:0000250|UniProtKB:P02768,
CC       ECO:0000269|PubMed:22677715, ECO:0000269|PubMed:6234017,
CC       ECO:0000305|PubMed:22677715}.
CC   -!- SUBUNIT: Interacts with FCGRT; this interaction regulates ALB
CC       homeostasis (By similarity). Interacts with TASOR (By similarity). In
CC       plasma, occurs in a covalently-linked complex with chromophore-bound
CC       alpha-1-microglobulin; this interaction does not prevent fatty acid
CC       binding to ALB. {ECO:0000250|UniProtKB:P02768,
CC       ECO:0000250|UniProtKB:P07724}.
CC   -!- INTERACTION:
CC       P02769; P08603: CFH; Xeno; NbExp=10; IntAct=EBI-2296927, EBI-1223708;
CC       P02769; PRO_0000009136 [O00602]: FCN1; Xeno; NbExp=2; IntAct=EBI-2296927, EBI-11784425;
CC   -!- SUBCELLULAR LOCATION: Secreted.
CC   -!- TISSUE SPECIFICITY: Plasma.
CC   -!- PTM: Phosphorylated by FAM20C in the extracellular medium.
CC       {ECO:0000250|UniProtKB:P02768}.
CC   -!- ALLERGEN: Can cause allergic reactions in humans.
CC   -!- SIMILARITY: Belongs to the ALB/AFP/VDB family. {ECO:0000255|PROSITE-
CC       ProRule:PRU00769}.
CC   -!- CAUTION: A peptide arising from positions 165 to 173 was originally
CC       termed neurotensin-related peptide (NRP) and was thought to regulate
CC       fat digestion, lipid absorption, and blood flow.
CC       {ECO:0000305|PubMed:2437111}.
CC   ---------------------------------------------------------------------------
CC   Copyrighted by the UniProt Consortium, see https://www.uniprot.org/terms
CC   Distributed under the Creative Commons Attribution (CC BY 4.0) License
CC   ---------------------------------------------------------------------------
DR   EMBL; M73993; AAA51411.1; -; mRNA.
DR   EMBL; X58989; CAA41735.1; -; mRNA.
DR   EMBL; Y17769; CAA76847.1; -; mRNA.
DR   EMBL; AF542068; AAN17824.1; -; mRNA.
DR   EMBL; BC102742; AAI02743.1; -; mRNA.
DR   EMBL; BC142272; AAI42273.1; -; mRNA.
DR   PIR; A38885; ABBOS.
DR   RefSeq; NP_851335.1; NM_180992.2.
DR   PDB; 2L7U; NMR; -; B=148-154.
DR   PDB; 3V03; X-ray; 2.70 A; A/B=25-607.
DR   PDB; 4F5S; X-ray; 2.47 A; A/B=25-607.
DR   PDB; 4JK4; X-ray; 2.65 A; A/B=25-607.
DR   PDB; 4OR0; X-ray; 2.58 A; A/B=25-607.
DR   PDB; 6QS9; X-ray; 2.80 A; A/B=1-607.
DR   PDB; 6RJV; X-ray; 3.21 A; A/B=25-607.
DR   PDBsum; 2L7U; -.
DR   PDBsum; 3V03; -.
DR   PDBsum; 4F5S; -.
DR   PDBsum; 4JK4; -.
DR   PDBsum; 4OR0; -.
DR   PDBsum; 6QS9; -.
DR   PDBsum; 6RJV; -.
```

```
DR   BMRB; P02769; -.
DR   SASBDB; P02769; -.
DR   SMR; P02769; -.
DR   BioGRID; 158123; 5.
DR   IntAct; P02769; 8.
DR   STRING; 9913.ENSBTAP00000022763; -.
DR   BindingDB; P02769; -.
DR   ChEMBL; CHEMBL3728; -.
DR   DrugCentral; P02769; -.
DR   Allergome; 165; Bos d 6.
DR   Allergome; 3166; Bos d 6.0101.
DR   CarbonylDB; P02769; -.
DR   PaxDb; P02769; -.
DR   PeptideAtlas; P02769; -.
DR   PRIDE; P02769; -.
DR   ABCD; P02769; 3 sequenced antibodies.
DR   GeneID; 280717; -.
DR   KEGG; bta:280717; -.
DR   CTD; 213; -.
DR   eggNOG; ENOG502R7EA; Eukaryota.
DR   HOGENOM; CLU_030161_0_0_1; -.
DR   InParanoid; P02769; -.
DR   OrthoDB; 906547at2759; -.
DR   TreeFam; TF335561; -.
DR   SABIO-RK; P02769; -.
DR   PRO; PR:P02769; -.
DR   Proteomes; UP000009136; Unplaced.
DR   GO; GO:0005576; C:extracellular region; TAS:Reactome.
DR   GO; GO:0005615; C:extracellular space; IEA:InterPro.
DR   GO; GO:0032991; C:protein-containing complex; ISS:UniProtKB.
DR   GO; GO:0003677; F:DNA binding; ISS:UniProtKB.
DR   GO; GO:1903981; F:enterobactin binding; IDA:UniProtKB.
DR   GO; GO:0005504; F:fatty acid binding; ISS:UniProtKB.
DR   GO; GO:0046872; F:metal ion binding; IEA:UniProtKB-KW.
DR   GO; GO:0030170; F:pyridoxal phosphate binding; ISS:UniProtKB.
DR   GO; GO:0015643; F:toxic substance binding; ISS:UniProtKB.
DR   GO; GO:0009267; P:cellular response to starvation; ISS:UniProtKB.
DR   GO; GO:0051659; P:maintenance of mitochondrion location; ISS:UniProtKB.
DR   GO; GO:0043066; P:negative regulation of apoptotic process; ISS:UniProtKB.
DR   CDD; cd00015; ALBUMIN; 3.
DR   InterPro; IPR000264; ALB/AFP/VDB.
DR   InterPro; IPR020858; Serum_albumin-like.
DR   InterPro; IPR021177; Serum_albumin/AFP/Afamin.
DR   InterPro; IPR020857; Serum_albumin_CS.
DR   InterPro; IPR014760; Serum_albumin_N.
DR   PANTHER; PTHR11385; PTHR11385; 1.
DR   Pfam; PF00273; Serum_albumin; 3.
DR   PIRSF; PIRSF002520; Serum_albumin_subgroup; 1.
DR   PRINTS; PR00803; AFETOPROTEIN.
DR   PRINTS; PR00802; SERUMALBUMIN.
DR   SMART; SM00103; ALBUMIN; 3.
DR   SUPFAM; SSF48552; SSF48552; 3.
DR   PROSITE; PS00212; ALBUMIN_1; 3.
DR   PROSITE; PS51438; ALBUMIN_2; 3.
PE   1: Evidence at protein level;
KW   3D-structure; Allergen; Calcium; Cleavage on pair of basic residues;
KW   Copper; Direct protein sequencing; Disulfide bond; Glycation; Glycoprotein;
KW   Lipid-binding; Metal-binding; Methylation; Phosphoprotein;
KW   Reference proteome; Repeat; Secreted; Signal; Zinc.
FT   SIGNAL          1..18
FT                   /evidence="ECO:0000269|PubMed:843354"
FT   PROPEP          19..24
FT                   /id="PRO_0000001057"
FT   CHAIN           25..607
FT                   /note="Albumin"
FT                   /id="PRO_0000001058"
FT   DOMAIN          19..209
FT                   /note="Albumin 1"
FT                   /evidence="ECO:0000255|PROSITE-ProRule:PRU00769"
FT   DOMAIN          210..402
FT                   /note="Albumin 2"
FT                   /evidence="ECO:0000255|PROSITE-ProRule:PRU00769"
FT   DOMAIN          403..600
FT                   /note="Albumin 3"
FT                   /evidence="ECO:0000255|PROSITE-ProRule:PRU00769"
FT   METAL           27
FT                   /note="Copper"
FT                   /evidence="ECO:0000250|UniProtKB:P02770"
FT   METAL           30
FT                   /note="Calcium 1"
FT                   /evidence="ECO:0000269|PubMed:22677715,
FT                   ECO:0007744|PDB:3V03"
FT   METAL           37
FT                   /note="Calcium 2"
FT                   /evidence="ECO:0000269|PubMed:22677715,
FT                   ECO:0000269|PubMed:23769932, ECO:0007744|PDB:3V03,
FT                   ECO:0007744|PDB:4JK4"
FT   METAL           91
FT                   /note="Zinc; via tele nitrogen"
FT                   /evidence="ECO:0000250|UniProtKB:P02768"
FT   METAL           267
FT                   /note="Calcium 1"
FT                   /evidence="ECO:0000269|PubMed:22677715,
FT                   ECO:0000269|PubMed:23769932, ECO:0007744|PDB:3V03,
FT                   ECO:0007744|PDB:4JK4"
FT   METAL           270
FT                   /note="Zinc; via pros nitrogen"
```

```
FT                   /evidence="ECO:0000250|UniProtKB:P02768"
FT   METAL           272
FT                   /note="Calcium 1"
FT                   /evidence="ECO:0000269|PubMed:22677715,
FT                   ECO:0007744|PDB:3V03"
FT   METAL           272
FT                   /note="Zinc"
FT                   /evidence="ECO:0000250|UniProtKB:P02768"
FT   METAL           275
FT                   /note="Calcium 1"
FT                   /evidence="ECO:0000269|PubMed:22677715,
FT                   ECO:0000269|PubMed:23769932, ECO:0007744|PDB:3V03,
FT                   ECO:0007744|PDB:4JK4"
FT   METAL           278
FT                   /note="Calcium 2"
FT                   /evidence="ECO:0000269|PubMed:22677715,
FT                   ECO:0000269|PubMed:23769932, ECO:0007744|PDB:3V03,
FT                   ECO:0007744|PDB:4JK4"
FT   METAL           282
FT                   /note="Calcium 2"
FT                   /evidence="ECO:0000269|PubMed:22677715,
FT                   ECO:0000269|PubMed:23769932, ECO:0007744|PDB:3V03,
FT                   ECO:0007744|PDB:4JK4"
FT   MOD_RES         29
FT                   /note="Phosphoserine"
FT                   /evidence="ECO:0000250|UniProtKB:P02768"
FT   MOD_RES         82
FT                   /note="Phosphoserine"
FT                   /evidence="ECO:0000250|UniProtKB:P02768"
FT   MOD_RES         89
FT                   /note="Phosphoserine"
FT                   /evidence="ECO:0000250|UniProtKB:P02768"
FT   MOD_RES         107
FT                   /note="Phosphothreonine"
FT                   /evidence="ECO:0000250|UniProtKB:P02768"
FT   MOD_RES         228
FT                   /note="N6-succinyllysine"
FT                   /evidence="ECO:0000250|UniProtKB:P07724"
FT   MOD_RES         296
FT                   /note="Phosphoserine"
FT                   /evidence="ECO:0000250|UniProtKB:P07724"
FT   MOD_RES         442
FT                   /note="Phosphoserine"
FT                   /evidence="ECO:0000250|UniProtKB:P02768"
FT   MOD_RES         443
FT                   /note="Phosphothreonine"
FT                   /evidence="ECO:0000250|UniProtKB:P02768"
FT   MOD_RES         445
FT                   /note="Phosphothreonine"
FT                   /evidence="ECO:0000250|UniProtKB:P02768"
FT   MOD_RES         512
FT                   /note="Phosphoserine"
FT                   /evidence="ECO:0000250|UniProtKB:P02768"
FT   MOD_RES         557
FT                   /note="N6-methyllysine"
FT                   /evidence="ECO:0000250|UniProtKB:P02768"
FT   MOD_RES         569
FT                   /note="Phosphothreonine"
FT                   /evidence="ECO:0000250|UniProtKB:P02770"
FT   MOD_RES         587
FT                   /note="N6-succinyllysine"
FT                   /evidence="ECO:0000250|UniProtKB:P07724"
FT   CARBOHYD        151
FT                   /note="N-linked (Glc) (glycation) lysine; in vitro"
FT                   /evidence="ECO:0000269|PubMed:21565706"
FT   DISULFID        77..86
FT                   /evidence="ECO:0000255|PROSITE-ProRule:PRU00769,
FT                   ECO:0000269|PubMed:22677715, ECO:0000269|PubMed:22993082,
FT                   ECO:0000269|PubMed:23769932, ECO:0000269|Ref.17,
FT                   ECO:0007744|PDB:3V03, ECO:0007744|PDB:4F5S,
FT                   ECO:0007744|PDB:4JK4, ECO:0007744|PDB:4OR0"
FT   DISULFID        99..115
FT                   /evidence="ECO:0000255|PROSITE-ProRule:PRU00769,
FT                   ECO:0000269|PubMed:22677715, ECO:0000269|PubMed:22993082,
FT                   ECO:0000269|PubMed:23769932, ECO:0000269|Ref.17,
FT                   ECO:0007744|PDB:3V03, ECO:0007744|PDB:4F5S,
FT                   ECO:0007744|PDB:4JK4, ECO:0007744|PDB:4OR0"
FT   DISULFID        114..125
FT                   /evidence="ECO:0000255|PROSITE-ProRule:PRU00769,
FT                   ECO:0000269|PubMed:22677715, ECO:0000269|PubMed:22993082,
FT                   ECO:0000269|PubMed:23769932, ECO:0000269|Ref.17,
FT                   ECO:0007744|PDB:3V03, ECO:0007744|PDB:4F5S,
FT                   ECO:0007744|PDB:4JK4, ECO:0007744|PDB:4OR0"
FT   DISULFID        147..192
FT                   /evidence="ECO:0000255|PROSITE-ProRule:PRU00769,
FT                   ECO:0000269|PubMed:22677715, ECO:0000269|PubMed:22993082,
FT                   ECO:0000269|PubMed:23769932, ECO:0000269|Ref.17,
FT                   ECO:0007744|PDB:3V03, ECO:0007744|PDB:4F5S,
FT                   ECO:0007744|PDB:4JK4, ECO:0007744|PDB:4OR0"
FT   DISULFID        191..200
FT                   /evidence="ECO:0000255|PROSITE-ProRule:PRU00769,
FT                   ECO:0000269|PubMed:22677715, ECO:0000269|PubMed:22993082,
FT                   ECO:0000269|PubMed:23769932, ECO:0000269|Ref.17,
FT                   ECO:0007744|PDB:3V03, ECO:0007744|PDB:4F5S,
FT                   ECO:0007744|PDB:4JK4, ECO:0007744|PDB:4OR0"
FT   DISULFID        223..269
FT                   /evidence="ECO:0000255|PROSITE-ProRule:PRU00769,
```

```
FT                   ECO:0000269|PubMed:22677715, ECO:0000269|PubMed:22993082,
FT                   ECO:0000269|PubMed:23769932, ECO:0000269|Ref.17,
FT                   ECO:0007744|PDB:3V03, ECO:0007744|PDB:4F5S,
FT                   ECO:0007744|PDB:4JK4, ECO:0007744|PDB:4OR0"
FT   DISULFID        268..276
FT                   /evidence="ECO:0000255|PROSITE-ProRule:PRU00769,
FT                   ECO:0000269|PubMed:22677715, ECO:0000269|PubMed:22993082,
FT                   ECO:0000269|PubMed:23769932, ECO:0000269|Ref.17,
FT                   ECO:0007744|PDB:3V03, ECO:0007744|PDB:4F5S,
FT                   ECO:0007744|PDB:4JK4, ECO:0007744|PDB:4OR0"
FT   DISULFID        288..302
FT                   /evidence="ECO:0000255|PROSITE-ProRule:PRU00769,
FT                   ECO:0000269|PubMed:22677715, ECO:0000269|PubMed:22993082,
FT                   ECO:0000269|PubMed:23769932, ECO:0000269|Ref.17,
FT                   ECO:0007744|PDB:3V03, ECO:0007744|PDB:4F5S,
FT                   ECO:0007744|PDB:4JK4, ECO:0007744|PDB:4OR0"
FT   DISULFID        301..312
FT                   /evidence="ECO:0000255|PROSITE-ProRule:PRU00769,
FT                   ECO:0000269|PubMed:22677715, ECO:0000269|PubMed:22993082,
FT                   ECO:0000269|PubMed:23769932, ECO:0000269|Ref.17,
FT                   ECO:0007744|PDB:3V03, ECO:0007744|PDB:4F5S,
FT                   ECO:0007744|PDB:4JK4, ECO:0007744|PDB:4OR0"
FT   DISULFID        339..384
FT                   /evidence="ECO:0000255|PROSITE-ProRule:PRU00769,
FT                   ECO:0000269|PubMed:22677715, ECO:0000269|PubMed:22993082,
FT                   ECO:0000269|PubMed:23769932, ECO:0000269|Ref.17,
FT                   ECO:0007744|PDB:3V03, ECO:0007744|PDB:4F5S,
FT                   ECO:0007744|PDB:4JK4, ECO:0007744|PDB:4OR0"
FT   DISULFID        383..392
FT                   /evidence="ECO:0000255|PROSITE-ProRule:PRU00769,
FT                   ECO:0000269|PubMed:22677715, ECO:0000269|PubMed:22993082,
FT                   ECO:0000269|PubMed:23769932, ECO:0000269|Ref.17,
FT                   ECO:0007744|PDB:3V03, ECO:0007744|PDB:4F5S,
FT                   ECO:0007744|PDB:4JK4, ECO:0007744|PDB:4OR0"
FT   DISULFID        415..461
FT                   /evidence="ECO:0000255|PROSITE-ProRule:PRU00769,
FT                   ECO:0000269|PubMed:22677715, ECO:0000269|PubMed:22993082,
FT                   ECO:0000269|PubMed:23769932, ECO:0000269|Ref.17,
FT                   ECO:0007744|PDB:3V03, ECO:0007744|PDB:4F5S,
FT                   ECO:0007744|PDB:4JK4, ECO:0007744|PDB:4OR0"
FT   DISULFID        460..471
FT                   /evidence="ECO:0000255|PROSITE-ProRule:PRU00769,
FT                   ECO:0000269|PubMed:22677715, ECO:0000269|PubMed:22993082,
FT                   ECO:0000269|PubMed:23769932, ECO:0000269|Ref.17,
FT                   ECO:0007744|PDB:3V03, ECO:0007744|PDB:4F5S,
FT                   ECO:0007744|PDB:4JK4, ECO:0007744|PDB:4OR0"
FT   DISULFID        484..500
FT                   /evidence="ECO:0000255|PROSITE-ProRule:PRU00769,
FT                   ECO:0000269|PubMed:22677715, ECO:0000269|PubMed:22993082,
FT                   ECO:0000269|PubMed:23769932, ECO:0000269|Ref.17,
FT                   ECO:0007744|PDB:3V03, ECO:0007744|PDB:4F5S,
FT                   ECO:0007744|PDB:4JK4, ECO:0007744|PDB:4OR0"
FT   DISULFID        499..510
FT                   /evidence="ECO:0000255|PROSITE-ProRule:PRU00769,
FT                   ECO:0000269|PubMed:22677715, ECO:0000269|PubMed:22993082,
FT                   ECO:0000269|PubMed:23769932, ECO:0000269|Ref.17,
FT                   ECO:0007744|PDB:3V03, ECO:0007744|PDB:4F5S,
FT                   ECO:0007744|PDB:4JK4, ECO:0007744|PDB:4OR0"
FT   DISULFID        537..582
FT                   /evidence="ECO:0000255|PROSITE-ProRule:PRU00769,
FT                   ECO:0000269|PubMed:22677715, ECO:0000269|PubMed:22993082,
FT                   ECO:0000269|PubMed:23769932, ECO:0000269|Ref.17,
FT                   ECO:0007744|PDB:3V03, ECO:0007744|PDB:4F5S,
FT                   ECO:0007744|PDB:4JK4, ECO:0007744|PDB:4OR0"
FT   DISULFID        581..590
FT                   /evidence="ECO:0000255|PROSITE-ProRule:PRU00769,
FT                   ECO:0000269|PubMed:22677715, ECO:0000269|PubMed:22993082,
FT                   ECO:0000269|PubMed:23769932, ECO:0000269|Ref.17,
FT                   ECO:0007744|PDB:3V03, ECO:0007744|PDB:4F5S,
FT                   ECO:0007744|PDB:4JK4, ECO:0007744|PDB:4OR0"
FT   VARIANT         214
FT                   /note="A -> T"
FT                   /evidence="ECO:0000269|PubMed:22993082, ECO:0000269|Ref.2,
FT                   ECO:0000269|Ref.4, ECO:0000269|Ref.5, ECO:0000269|Ref.9"
FT   CONFLICT        58
FT                   /note="Missing (in Ref. 11; AA sequence)"
FT                   /evidence="ECO:0000305"
FT   CONFLICT        116
FT                   /note="E -> A (in Ref. 5; AAI02743)"
FT                   /evidence="ECO:0000305"
FT   CONFLICT        173
FT                   /note="Y -> L (in Ref. 14; AA sequence)"
FT                   /evidence="ECO:0000305"
FT   CONFLICT        302
FT                   /note="C -> K (in Ref. 9; AA sequence)"
FT                   /evidence="ECO:0000305"
FT   CONFLICT        304..305
FT                   /note="KP -> PC (in Ref. 9; AA sequence)"
FT                   /evidence="ECO:0000305"
FT   CONFLICT        324
FT                   /note="N -> D (in Ref. 9; AA sequence)"
FT                   /evidence="ECO:0000305"
FT   CONFLICT        394..395
FT                   /note="ST -> TS (in Ref. 9; AA sequence)"
FT                   /evidence="ECO:0000305"
FT   CONFLICT        429
FT                   /note="A -> E (in Ref. 5; AAI02743)"
```

```
FT                   /evidence="ECO:0000305"
FT   CONFLICT        437
FT                   /note="K -> R (in Ref. 16; AA sequence)"
FT                   /evidence="ECO:0000305"
FT   CONFLICT        472
FT                   /note="T -> A (in Ref. 5; AAI02743)"
FT                   /evidence="ECO:0000305"
FT   CONFLICT        493..494
FT                   /note="SE -> ES (in Ref. 9; AA sequence)"
FT                   /evidence="ECO:0000305"
FT   CONFLICT        579
FT                   /note="D -> G (in Ref. 5; AAI02743)"
FT                   /evidence="ECO:0000305"
FT   HELIX           30..38
FT                   /evidence="ECO:0007829|PDB:4F5S"
FT   HELIX           40..54
FT                   /evidence="ECO:0007829|PDB:4F5S"
FT   STRAND          56..58
FT                   /evidence="ECO:0007829|PDB:4F5S"
FT   HELIX           60..79
FT                   /evidence="ECO:0007829|PDB:4F5S"
FT   TURN            84..87
FT                   /evidence="ECO:0007829|PDB:4F5S"
FT   HELIX           90..99
FT                   /evidence="ECO:0007829|PDB:4F5S"
FT   HELIX           104..108
FT                   /evidence="ECO:0007829|PDB:4F5S"
FT   HELIX           109..116
FT                   /evidence="ECO:0007829|PDB:4F5S"
FT   HELIX           121..127
FT                   /evidence="ECO:0007829|PDB:4F5S"
FT   HELIX           143..152
FT                   /evidence="ECO:0007829|PDB:4F5S"
FT   HELIX           154..168
FT                   /evidence="ECO:0007829|PDB:4F5S"
FT   HELIX           174..191
FT                   /evidence="ECO:0007829|PDB:4F5S"
FT   STRAND          194..196
FT                   /evidence="ECO:0007829|PDB:4OR0"
FT   HELIX           197..229
FT                   /evidence="ECO:0007829|PDB:4F5S"
FT   HELIX           231..245
FT                   /evidence="ECO:0007829|PDB:4F5S"
FT   HELIX           251..269
FT                   /evidence="ECO:0007829|PDB:4F5S"
FT   HELIX           273..288
FT                   /evidence="ECO:0007829|PDB:4F5S"
FT   HELIX           291..294
FT                   /evidence="ECO:0007829|PDB:4F5S"
FT   HELIX           296..298
FT                   /evidence="ECO:0007829|PDB:4JK4"
FT   HELIX           301..303
FT                   /evidence="ECO:0007829|PDB:4F5S"
FT   HELIX           306..315
FT                   /evidence="ECO:0007829|PDB:4F5S"
FT   HELIX           329..332
FT                   /evidence="ECO:0007829|PDB:4F5S"
FT   HELIX           338..344
FT                   /evidence="ECO:0007829|PDB:4F5S"
FT   HELIX           346..360
FT                   /evidence="ECO:0007829|PDB:4F5S"
FT   HELIX           366..385
FT                   /evidence="ECO:0007829|PDB:4F5S"
FT   STRAND          386..388
FT                   /evidence="ECO:0007829|PDB:4JK4"
FT   HELIX           389..393
FT                   /evidence="ECO:0007829|PDB:4F5S"
FT   HELIX           396..403
FT                   /evidence="ECO:0007829|PDB:4F5S"
FT   TURN            404..407
FT                   /evidence="ECO:0007829|PDB:4F5S"
FT   HELIX           408..437
FT                   /evidence="ECO:0007829|PDB:4F5S"
FT   HELIX           443..460
FT                   /evidence="ECO:0007829|PDB:4F5S"
FT   STRAND          461..463
FT                   /evidence="ECO:0007829|PDB:4F5S"
FT   HELIX           465..489
FT                   /evidence="ECO:0007829|PDB:4F5S"
FT   HELIX           494..502
FT                   /evidence="ECO:0007829|PDB:4F5S"
FT   HELIX           507..512
FT                   /evidence="ECO:0007829|PDB:4F5S"
FT   STRAND          518..520
FT                   /evidence="ECO:0007829|PDB:4JK4"
FT   HELIX           527..530
FT                   /evidence="ECO:0007829|PDB:4F5S"
FT   HELIX           534..537
FT                   /evidence="ECO:0007829|PDB:4F5S"
FT   HELIX           541..558
FT                   /evidence="ECO:0007829|PDB:4F5S"
FT   HELIX           564..583
FT                   /evidence="ECO:0007829|PDB:4F5S"
FT   STRAND          584..586
FT                   /evidence="ECO:0007829|PDB:4OR0"
FT   HELIX           587..606
FT                   /evidence="ECO:0007829|PDB:4F5S"
```

```
SQ   SEQUENCE   607 AA;  69293 MW;  39167DFE768585D4 CRC64;
     MKWVTFISLL LLFSSAYSRG VFRRDTHKSE IAHRFKDLGE EHFKGLVLIA FSQYLQQCPF
     DEHVKLVNEL TEFAKTCVAD ESHAGCEKSL HTLFGDELCK VASLRETYGD MADCCEKQEP
     ERNECFLSHK DDSPDLPKLK PDPNTLCDEF KADEKKFWGK YLYEIARRHP YFYAPELLYY
     ANKYNGVFQE CCQAEDKGAC LLPKIETMRE KVLASSARQR LRCASIQKFG ERALKAWSVA
     RLSQKFPKAE FVEVTKLVTD LTKVHKECCH GDLLECADDR ADLAKYICDN QDTISSKLKE
     CCDKPLLEKS HCIAEVEKDA IPENLPPLTA DFAEDKDVCK NYQEAKDAFL GSFLYEYSRR
     HPEYAVSVLL RLAKEYEATL EECCAKDDPH ACYSTVFDKL KHLVDEPQNL IKQNCDQFEK
     LGEYGFQNAL IVRYTRKVPQ VSTPTLVEVS RSLGKVGTRC CTKPESERMP CTEDYLSLIL
     NRLCVLHEKT PVSEKVTKCC TESLVNRRPC FSALTPDETY VPKAFDEKLF TFHADICTLP
     DTEKQIKKQT ALVELLKHKP KATEEQLKTV MENFVAFVDK CCAADDKEAC FAVEGPKLVV
     STQTALA
```

Mascot: http://www.matrixscience.com/

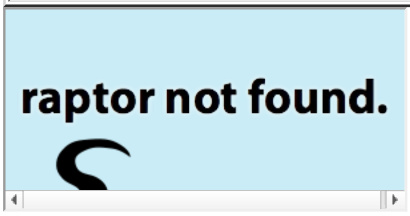

Supplement: Supplementary file 1 [file molecules-27-03232-s001.zip › S1.pdf]
