# Supplementary material for: Effect of Ionic and Non-Ionic Surfactant on Bovine Serum Albumin Encapsulation and Biological Properties of Emulsion-Electrospun Fibers
Source: Molecules. 2022 May 18;27(10):3232. doi: 10.3390/molecules27103232 (PMC9143061; doi:10.3390/molecules27103232)
Supplement: Supplementary file 1 [file molecules-27-03232-s001.zip › S2.pdf]

Mascot Search Results

```
User            : ps
Email           : psuder@agh.edu.pl
Search title    : E_S-Z_albumin
MS data file    : BSA.mgf
Database        : SwissProt 2022_01 (566996 sequences; 204698499 residues)
Timestamp       : 9 May 2022 at 08:24:10 GMT
Protein hits    : ALBU_BOVIN Albumin OS=Bos taurus OX=9913 GN=ALB PE=1 SV=4
                  ALBU_SHEEP Albumin OS=Ovis aries OX=9940 GN=ALB PE=1 SV=1
                  ALBU_FELCA Albumin OS=Felis catus OX=9685 GN=ALB PE=1 SV=1
                  ALBU_PIG   Albumin OS=Sus scrofa OX=9823 GN=ALB PE=1 SV=2
                  ALBU_CANLF Albumin OS=Canis lupus familiaris OX=9615 GN=ALB PE=1 SV=3
                  ALBU_MESAU Albumin OS=Mesocricetus auratus OX=10036 GN=ALB PE=1 SV=1
                  ALBU_MOUSE Albumin OS=Mus musculus OX=10090 GN=Alb PE=1 SV=3
                  A1AG_BOVIN Alpha-1-acid glycoprotein OS=Bos taurus OX=9913 GN=ORM1 PE=2 SV=1
                  K1C9_HUMAN Keratin, type I cytoskeletal 9 OS=Homo sapiens OX=9606 GN=KRT9 PE=1 SV=3
                  ALBU_MERUN Albumin OS=Meriones unguiculatus OX=10047 GN=ALB PE=2 SV=1
                  TRYP_PIG   Trypsin OS=Sus scrofa OX=9823 PE=1 SV=1
                  VTDB_BOVIN Vitamin D-binding protein OS=Bos taurus OX=9913 GN=GC PE=2 SV=1
                  K2C1_HUMAN Keratin, type II cytoskeletal 1 OS=Homo sapiens OX=9606 GN=KRT1 PE=1 SV=6
                  VNN1_BOVIN Pantetheinase OS=Bos taurus OX=9913 GN=VNN1 PE=1 SV=1
                  TRY1_RAT   Serine protease 1 OS=Rattus norvegicus OX=10116 GN=Prss1 PE=1 SV=1
                  CERU_HUMAN Ceruloplasmin OS=Homo sapiens OX=9606 GN=CP PE=1 SV=1
                  LPXD_PARMW UDP-3-O-acylglucosamine N-acyltransferase OS=Parasynechococcus marenigrum (strain WH8102) OX=84588 GN=lpxD PE=3 SV=1
                  MCFM_DICDI Mitochondrial substrate carrier family protein M OS=Dictyostelium discoideum OX=44689 GN=mcfM PE=3 SV=1
```

Mascot Score Histogram

Ions score is -10*Log(P), where P is the probability that the observed match is a random event.
Individual ions scores > 44 indicate identity or extensive homology(p<0.05).
Protein scores are derived from ions scores as a non-probabilistic basis for ranking protein hits.

Score Distribution

Peptide Summary Report

Format As	Peptide Summary		Help
	Significance threshold p< 0.05	Max. number of hits AUTO	
	Standard scoring ○ MudPIT scoring ◉	Display non-significant matches ☐	Show sub-sets 0
	Show pop-ups ◉ Suppress pop-ups ○	Sort unassigned Decreasing Score	Require bold red ☐
	Preferred taxonomy All entries		

Select All | Select None | Search Selected | ☐ **Error tolerant**

1. ALBU_BOVIN **Mass:** 71244 **Score:** 20958 **Matches:** 573(573) **Sequences:** 46(46) **emPAI:** 6.63
Albumin OS=Bos taurus OX=9913 GN=ALB PE=1 SV=4
☐ Check to include this hit in error tolerant search

	Query	Observed	Mr(expt)	Mr(calc)	Delta	Miss	Score	Expect	Rank	Unique	Peptide
	2802	501.2960	1000.5774	1000.5818	-0.0043	1	(49)	0.04	2		R.ALKAWSVAR.L
☒	2803	501.2961	1000.5776	1000.5818	-0.0041	1	53	0.017	1		R.ALKAWSVAR.L
☒	2923	1014.6146	1013.6073	1013.6121	-0.0048	0	(55)	0.011	1		K.QTALVELLK.H
☒	2938	1014.6152	1013.6079	1013.6121	-0.0042	0	60	0.0029	1		K.QTALVELLK.H
☒	2949	1014.6168	1013.6095	1013.6121	-0.0026	0	(57)	0.0063	1		K.QTALVELLK.H
☒	2950	1014.6179	1013.6106	1013.6121	-0.0015	0	(51)	0.027	1		K.QTALVELLK.H
☒	3106	571.8574	1141.7002	1141.7070	-0.0068	1	(62)	0.0018	1		K.KQTALVELLK.H
☒	3108	571.8578	1141.7010	1141.7070	-0.0060	1	(66)	0.00066	1		K.KQTALVELLK.H
☒	3109	571.8579	1141.7012	1141.7070	-0.0058	1	(70)	0.00028	1		K.KQTALVELLK.H
☒	3110	571.8579	1141.7012	1141.7070	-0.0058	1	(70)	0.00027	1		K.KQTALVELLK.H
☒	3113	571.8580	1141.7014	1141.7070	-0.0056	1	(70)	0.00028	1		K.KQTALVELLK.H
☒	3114	571.8582	1141.7018	1141.7070	-0.0052	1	(70)	0.00028	1		K.KQTALVELLK.H
☒	3115	571.8582	1141.7018	1141.7070	-0.0052	1	(67)	0.00055	1		K.KQTALVELLK.H
☒	3116	571.8582	1141.7018	1141.7070	-0.0052	1	(64)	0.0011	1		K.KQTALVELLK.H
☒	3117	571.8582	1141.7018	1141.7070	-0.0052	1	(50)	0.03	1		K.KQTALVELLK.H
☒	3121	571.8583	1141.7020	1141.7070	-0.0050	1	(70)	0.00027	1		K.KQTALVELLK.H
☒	3122	571.8583	1141.7020	1141.7070	-0.0050	1	(67)	0.00054	1		K.KQTALVELLK.H
☒	3123	571.8584	1141.7022	1141.7070	-0.0048	1	(67)	0.00052	1		K.KQTALVELLK.H
☒	3127	571.8585	1141.7024	1141.7070	-0.0046	1	(70)	0.00027	1		K.KQTALVELLK.H
☒	3128	571.8585	1141.7024	1141.7070	-0.0046	1	(66)	0.00066	1		K.KQTALVELLK.H
☒	3129	571.8585	1141.7024	1141.7070	-0.0046	1	(67)	0.00062	1		K.KQTALVELLK.H
☒	3133	571.8588	1141.7030	1141.7070	-0.0040	1	(67)	0.00063	1		K.KQTALVELLK.H
☒	3135	571.8589	1141.7032	1141.7070	-0.0038	1	(51)	0.022	1		K.KQTALVELLK.H
☒	3139	571.8591	1141.7036	1141.7070	-0.0034	1	(65)	0.00093	1		K.KQTALVELLK.H
☒	3140	571.8591	1141.7036	1141.7070	-0.0034	1	(56)	0.0066	1		K.KQTALVELLK.H
☒	3141	571.8593	1141.7040	1141.7070	-0.0030	1	70	0.00027	1		K.KQTALVELLK.H
☒	3156	577.3480	1152.6814	1152.6867	-0.0052	1	(56)	0.0061	1	U	K.LVTDLTKVHK.E
☒	3160	385.5687	1153.6843	1152.6867	0.9976	1	65	0.00087	1	U	K.LVTDLTKVHK.E
☒	3165	582.3158	1162.6170	1162.6234	-0.0063	0	(56)	0.0074	1	U	K.LVNELTEFAK.T
☒	3166	582.3160	1162.6174	1162.6234	-0.0059	0	(52)	0.019	1	U	K.LVNELTEFAK.T
☒	3167	582.3160	1162.6174	1162.6234	-0.0059	0	57	0.0067	1	U	K.LVNELTEFAK.T
☒	3170	582.3162	1162.6178	1162.6234	-0.0055	0	(56)	0.0072	1	U	K.LVNELTEFAK.T
☒	3175	582.3163	1162.6180	1162.6234	-0.0053	0	(50)	0.034	1	U	K.LVNELTEFAK.T
☒	3176	1163.6254	1162.6181	1162.6234	-0.0052	0	(54)	0.011	1	U	K.LVNELTEFAK.T
☒	3180	582.3165	1162.6184	1162.6234	-0.0049	0	(56)	0.007	1	U	K.LVNELTEFAK.T
☒	3181	582.3166	1162.6186	1162.6234	-0.0047	0	(49)	0.041	1	U	K.LVNELTEFAK.T
☒	3182	582.3167	1162.6188	1162.6234	-0.0045	0	(54)	0.012	1	U	K.LVNELTEFAK.T
☒	3185	582.3168	1162.6190	1162.6234	-0.0043	0	(49)	0.038	1	U	K.LVNELTEFAK.T

3186	582.3168	1162.6190	1162.6234	-0.0043	0	(48)	0.044	1	U	K.LVNELTEFAK.T
3189	582.3169	1162.6192	1162.6234	-0.0041	0	(57)	0.0068	1	U	K.LVNELTEFAK.T
3192	582.3171	1162.6196	1162.6234	-0.0037	0	(53)	0.017	1	U	K.LVNELTEFAK.T
3194	582.3174	1162.6202	1162.6234	-0.0031	0	(53)	0.017	1	U	K.LVNELTEFAK.T
3385	642.3547	1282.6948	1282.7034	-0.0085	0	(53)	0.013	1		R.HPEYAVSVLLR.L
3387	642.3557	1282.6968	1282.7034	-0.0065	0	(56)	0.0063	1		R.HPEYAVSVLLR.L
3388	642.3561	1282.6976	1282.7034	-0.0057	0	62	0.0015	1		R.HPEYAVSVLLR.L
3389	428.5732	1282.6978	1282.7034	-0.0056	0	(49)	0.031	1		R.HPEYAVSVLLR.L
3390	428.5732	1282.6978	1282.7034	-0.0056	0	(58)	0.0046	1		R.HPEYAVSVLLR.L
3391	642.3568	1282.6990	1282.7034	-0.0043	0	(61)	0.0023	1		R.HPEYAVSVLLR.L
3392	642.3569	1282.6992	1282.7034	-0.0041	0	(57)	0.0048	1		R.HPEYAVSVLLR.L
3393	642.3570	1282.6994	1282.7034	-0.0039	0	(57)	0.0053	1		R.HPEYAVSVLLR.L
3394	428.5739	1282.6999	1282.7034	-0.0035	0	(49)	0.033	1		R.HPEYAVSVLLR.L
3397	642.8580	1283.7014	1282.7034	0.9981	0	(50)	0.027	1		R.HPEYAVSVLLR.L
3427	432.5721	1294.6945	1293.6969	0.9976	1	53	0.014	1	U	K.FPKAEFVEVTK.L
3463	1305.7092	1304.7019	1304.7088	-0.0069	0	(48)	0.04	1		K.HLVDEPQNLIK.Q
3468	653.3586	1304.7026	1304.7088	-0.0062	0	(48)	0.039	1		K.HLVDEPQNLIK.Q
3471	653.3588	1304.7030	1304.7088	-0.0058	0	(51)	0.022	1		K.HLVDEPQNLIK.Q
3473	653.3589	1304.7032	1304.7088	-0.0056	0	(48)	0.041	1		K.HLVDEPQNLIK.Q
3477	1305.7109	1304.7036	1304.7088	-0.0052	0	(48)	0.042	1		K.HLVDEPQNLIK.Q
3482	653.8584	1305.7022	1304.7088	0.9934	0	55	0.0077	1		K.HLVDEPQNLIK.Q
3694	700.3462	1398.6778	1398.6853	-0.0075	0	(74)	0.00011	1		K.TVMENFVAFVDK.C
3695	700.3462	1398.6778	1398.6853	-0.0075	0	(79)	3.7e-05	1		K.TVMENFVAFVDK.C
3696	700.3464	1398.6782	1398.6853	-0.0071	0	93	1.5e-06	1		K.TVMENFVAFVDK.C
3698	700.3479	1398.6812	1398.6853	-0.0041	0	(79)	3.7e-05	1		K.TVMENFVAFVDK.C
3699	700.3506	1398.6866	1398.6853	0.0013	0	(69)	0.00032	1		K.TVMENFVAFVDK.C
3702	700.8475	1399.6804	1398.6853	0.9951	0	(61)	0.0023	1		K.TVMENFVAFVDK.C
3703	700.8491	1399.6836	1398.6853	0.9983	0	(62)	0.0018	1		K.TVMENFVAFVDK.C
3704	700.8500	1399.6854	1398.6853	1.0001	0	(60)	0.0025	1		K.TVMENFVAFVDK.C
3705	700.8506	1399.6866	1398.6853	1.0013	0	(61)	0.002	1		K.TVMENFVAFVDK.C
3759	710.0826	1418.1506	1418.6864	-0.5358	0	(49)	0.021	1		K.SLHTLFGDELCK.V
3769	710.3472	1418.6798	1418.6864	-0.0066	0	(54)	0.011	1		K.SLHTLFGDELCK.V
3773	710.3474	1418.6802	1418.6864	-0.0062	0	(52)	0.017	1		K.SLHTLFGDELCK.V
3774	710.3474	1418.6802	1418.6864	-0.0062	0	(59)	0.0032	1		K.SLHTLFGDELCK.V
3775	710.3474	1418.6802	1418.6864	-0.0062	0	(55)	0.0077	1		K.SLHTLFGDELCK.V
3780	710.3475	1418.6804	1418.6864	-0.0060	0	(59)	0.0035	1		K.SLHTLFGDELCK.V
3781	710.3475	1418.6804	1418.6864	-0.0060	0	(57)	0.0052	1		K.SLHTLFGDELCK.V
3785	710.3478	1418.6810	1418.6864	-0.0054	0	(56)	0.0064	1		K.SLHTLFGDELCK.V
3789	1419.6888	1418.6815	1418.6864	-0.0049	0	66	0.00072	1		K.SLHTLFGDELCK.V
3790	1419.6888	1418.6815	1418.6864	-0.0049	0	(64)	0.00096	1		K.SLHTLFGDELCK.V
3791	710.3481	1418.6816	1418.6864	-0.0048	0	(58)	0.0046	1		K.SLHTLFGDELCK.V
3796	710.3484	1418.6822	1418.6864	-0.0042	0	(54)	0.011	1		K.SLHTLFGDELCK.V
3798	710.3485	1418.6824	1418.6864	-0.0040	0	(57)	0.0051	1		K.SLHTLFGDELCK.V
3799	710.3489	1418.6832	1418.6864	-0.0032	0	(64)	0.00094	1		K.SLHTLFGDELCK.V
3806	710.8486	1419.6826	1418.6864	0.9962	0	(64)	0.00096	1		K.SLHTLFGDELCK.V
3808	710.8488	1419.6830	1418.6864	0.9966	0	(50)	0.028	1		K.SLHTLFGDELCK.V
3809	710.8488	1419.6830	1418.6864	0.9966	0	(52)	0.016	1		K.SLHTLFGDELCK.V
3813	710.8495	1419.6844	1418.6864	0.9980	0	(50)	0.025	1		K.SLHTLFGDELCK.V
3848	720.1332	1438.2518	1438.8045	-0.5526	1	(50)	0.015	1		R.RHPEYAVSVLLR.L
3849	720.3298	1438.6450	1438.8045	-0.1594	1	(56)	0.0067	1		R.RHPEYAVSVLLR.L
3850	720.4041	1438.7936	1438.8045	-0.0108	1	(58)	0.004	1		R.RHPEYAVSVLLR.L
3851	720.4051	1438.7956	1438.8045	-0.0088	1	(70)	0.00027	1		R.RHPEYAVSVLLR.L
3852	480.6059	1438.7959	1438.8045	-0.0086	1	(50)	0.028	1		R.RHPEYAVSVLLR.L
3853	480.6061	1438.7965	1438.8045	-0.0080	1	(62)	0.0016	1		R.RHPEYAVSVLLR.L
3854	480.6061	1438.7965	1438.8045	-0.0080	1	(61)	0.0019	1		R.RHPEYAVSVLLR.L
3857	480.6063	1438.7971	1438.8045	-0.0074	1	78	4.3e-05	1		R.RHPEYAVSVLLR.L
3858	720.4059	1438.7972	1438.8045	-0.0072	1	(55)	0.0072	1		R.RHPEYAVSVLLR.L
3859	480.6064	1438.7974	1438.8045	-0.0071	1	(50)	0.023	1		R.RHPEYAVSVLLR.L
3861	480.6064	1438.7974	1438.8045	-0.0071	1	(62)	0.0016	1		R.RHPEYAVSVLLR.L
3862	720.4061	1438.7976	1438.8045	-0.0068	1	(68)	0.00041	1		R.RHPEYAVSVLLR.L
3863	480.6065	1438.7977	1438.8045	-0.0068	1	(70)	0.00028	1		R.RHPEYAVSVLLR.L
3864	480.6065	1438.7977	1438.8045	-0.0068	1	(55)	0.0085	1		R.RHPEYAVSVLLR.L
3865	480.6065	1438.7977	1438.8045	-0.0068	1	(62)	0.0016	1		R.RHPEYAVSVLLR.L
3866	480.6065	1438.7977	1438.8045	-0.0068	1	(75)	8.2e-05	1		R.RHPEYAVSVLLR.L
3868	720.4062	1438.7978	1438.8045	-0.0066	1	(66)	0.00064	1		R.RHPEYAVSVLLR.L
3869	720.4062	1438.7978	1438.8045	-0.0066	1	(74)	8.9e-05	1		R.RHPEYAVSVLLR.L
3871	480.6066	1438.7980	1438.8045	-0.0065	1	(62)	0.0016	1		R.RHPEYAVSVLLR.L
3872	480.6066	1438.7980	1438.8045	-0.0065	1	(59)	0.0032	1		R.RHPEYAVSVLLR.L
3873	480.6066	1438.7980	1438.8045	-0.0065	1	(62)	0.0016	1		R.RHPEYAVSVLLR.L
3874	480.6066	1438.7980	1438.8045	-0.0065	1	(62)	0.0017	1		R.RHPEYAVSVLLR.L
3875	720.4063	1438.7980	1438.8045	-0.0064	1	(54)	0.0098	1		R.RHPEYAVSVLLR.L
3876	720.4063	1438.7980	1438.8045	-0.0064	1	(70)	0.00023	1		R.RHPEYAVSVLLR.L
3879	480.6067	1438.7983	1438.8045	-0.0062	1	(57)	0.0055	1		R.RHPEYAVSVLLR.L
3880	480.6067	1438.7983	1438.8045	-0.0062	1	(61)	0.0019	1		R.RHPEYAVSVLLR.L
3881	480.6067	1438.7983	1438.8045	-0.0062	1	(65)	0.00087	1		R.RHPEYAVSVLLR.L
3882	480.6067	1438.7983	1438.8045	-0.0062	1	(65)	0.00082	1		R.RHPEYAVSVLLR.L
3883	480.6067	1438.7983	1438.8045	-0.0062	1	(71)	0.00019	1		R.RHPEYAVSVLLR.L
3884	480.6067	1438.7983	1438.8045	-0.0062	1	(65)	0.00089	1		R.RHPEYAVSVLLR.L
3885	480.6067	1438.7983	1438.8045	-0.0062	1	(54)	0.01	1		R.RHPEYAVSVLLR.L
3886	480.6067	1438.7983	1438.8045	-0.0062	1	(70)	0.00024	1		R.RHPEYAVSVLLR.L
3887	480.6067	1438.7983	1438.8045	-0.0062	1	(59)	0.0033	1		R.RHPEYAVSVLLR.L
3888	480.6067	1438.7983	1438.8045	-0.0062	1	(65)	0.00075	1		R.RHPEYAVSVLLR.L
3890	480.6068	1438.7986	1438.8045	-0.0059	1	(56)	0.0068	1		R.RHPEYAVSVLLR.L
3891	480.6068	1438.7986	1438.8045	-0.0059	1	(62)	0.0016	1		R.RHPEYAVSVLLR.L
3892	480.6068	1438.7986	1438.8045	-0.0059	1	(53)	0.014	1		R.RHPEYAVSVLLR.L
3893	480.6068	1438.7986	1438.8045	-0.0059	1	(71)	0.0002	1		R.RHPEYAVSVLLR.L
3894	480.6068	1438.7986	1438.8045	-0.0059	1	(61)	0.0019	1		R.RHPEYAVSVLLR.L
3895	480.6068	1438.7986	1438.8045	-0.0059	1	(62)	0.0016	1		R.RHPEYAVSVLLR.L
3896	480.6068	1438.7986	1438.8045	-0.0059	1	(54)	0.011	1		R.RHPEYAVSVLLR.L
3897	720.4066	1438.7986	1438.8045	-0.0058	1	(68)	0.0004	1		R.RHPEYAVSVLLR.L
3898	720.4067	1438.7988	1438.8045	-0.0056	1	(72)	0.00017	1		R.RHPEYAVSVLLR.L
3899	720.4067	1438.7988	1438.8045	-0.0056	1	(65)	0.00088	1		R.RHPEYAVSVLLR.L
3900	480.6069	1438.7989	1438.8045	-0.0056	1	(65)	0.00088	1		R.RHPEYAVSVLLR.L
3901	480.6069	1438.7989	1438.8045	-0.0056	1	(57)	0.0055	1		R.RHPEYAVSVLLR.L
3902	480.6069	1438.7989	1438.8045	-0.0056	1	(70)	0.00023	1		R.RHPEYAVSVLLR.L
3903	480.6069	1438.7989	1438.8045	-0.0056	1	(74)	8.9e-05	1		R.RHPEYAVSVLLR.L
3904	480.6069	1438.7989	1438.8045	-0.0056	1	(62)	0.0016	1		R.RHPEYAVSVLLR.L
3907	480.6070	1438.7992	1438.8045	-0.0053	1	(68)	0.00038	1		R.RHPEYAVSVLLR.L
3908	480.6070	1438.7992	1438.8045	-0.0053	1	(59)	0.0032	1		R.RHPEYAVSVLLR.L
3909	480.6070	1438.7992	1438.8045	-0.0053	1	(62)	0.0016	1		R.RHPEYAVSVLLR.L
3910	480.6070	1438.7992	1438.8045	-0.0053	1	(61)	0.0019	1		R.RHPEYAVSVLLR.L

3911	480.6070	1438.7992	1438.8045	-0.0053	1	(62)	0.0015	1		R.RHPEYAVSVLLR.L
3912	480.6070	1438.7992	1438.8045	-0.0053	1	(62)	0.0016	1		R.RHPEYAVSVLLR.L
3913	480.6070	1438.7992	1438.8045	-0.0053	1	(62)	0.0016	1		R.RHPEYAVSVLLR.L
3914	480.6070	1438.7992	1438.8045	-0.0053	1	(54)	0.0094	1		R.RHPEYAVSVLLR.L
3915	720.4069	1438.7992	1438.8045	-0.0052	1	(72)	0.00018	1		R.RHPEYAVSVLLR.L
3916	720.4069	1438.7992	1438.8045	-0.0052	1	(60)	0.0023	1		R.RHPEYAVSVLLR.L
3917	720.4069	1438.7992	1438.8045	-0.0052	1	(69)	0.00032	1		R.RHPEYAVSVLLR.L
3918	720.4070	1438.7994	1438.8045	-0.0050	1	(66)	0.0006	1		R.RHPEYAVSVLLR.L
3919	480.6071	1438.7995	1438.8045	-0.0050	1	(62)	0.0016	1		R.RHPEYAVSVLLR.L
3920	480.6071	1438.7995	1438.8045	-0.0050	1	(52)	0.016	1		R.RHPEYAVSVLLR.L
3921	480.6071	1438.7995	1438.8045	-0.0050	1	(65)	0.00085	1		R.RHPEYAVSVLLR.L
3922	480.6071	1438.7995	1438.8045	-0.0050	1	(62)	0.0016	1		R.RHPEYAVSVLLR.L
3923	480.6071	1438.7995	1438.8045	-0.0050	1	(61)	0.0018	1		R.RHPEYAVSVLLR.L
3924	480.6071	1438.7995	1438.8045	-0.0050	1	(62)	0.0016	1		R.RHPEYAVSVLLR.L
3925	480.6071	1438.7995	1438.8045	-0.0050	1	(57)	0.0054	1		R.RHPEYAVSVLLR.L
3926	480.6071	1438.7995	1438.8045	-0.0050	1	(59)	0.0034	1		R.RHPEYAVSVLLR.L
3927	480.6071	1438.7995	1438.8045	-0.0050	1	(65)	0.00087	1		R.RHPEYAVSVLLR.L
3928	480.6071	1438.7995	1438.8045	-0.0050	1	(53)	0.013	1		R.RHPEYAVSVLLR.L
3929	480.6071	1438.7995	1438.8045	-0.0050	1	(60)	0.0028	1		R.RHPEYAVSVLLR.L
3930	480.6071	1438.7995	1438.8045	-0.0050	1	(60)	0.0027	1		R.RHPEYAVSVLLR.L
3931	480.6071	1438.7995	1438.8045	-0.0050	1	(60)	0.0025	1		R.RHPEYAVSVLLR.L
3932	720.4071	1438.7996	1438.8045	-0.0048	1	(75)	7.4e-05	1		R.RHPEYAVSVLLR.L
3934	480.6072	1438.7998	1438.8045	-0.0047	1	(61)	0.0019	1		R.RHPEYAVSVLLR.L
3935	480.6072	1438.7998	1438.8045	-0.0047	1	(60)	0.0024	1		R.RHPEYAVSVLLR.L
3936	480.6072	1438.7998	1438.8045	-0.0047	1	(57)	0.0046	1		R.RHPEYAVSVLLR.L
3937	480.6072	1438.7998	1438.8045	-0.0047	1	(62)	0.0015	1		R.RHPEYAVSVLLR.L
3938	480.6072	1438.7998	1438.8045	-0.0047	1	(60)	0.0022	1		R.RHPEYAVSVLLR.L
3939	480.6072	1438.7998	1438.8045	-0.0047	1	(65)	0.00088	1		R.RHPEYAVSVLLR.L
3940	720.4073	1438.8000	1438.8045	-0.0044	1	(66)	0.0006	1		R.RHPEYAVSVLLR.L
3941	720.4073	1438.8000	1438.8045	-0.0044	1	(56)	0.006	1		R.RHPEYAVSVLLR.L
3942	480.6073	1438.8001	1438.8045	-0.0044	1	(57)	0.0046	1		R.RHPEYAVSVLLR.L
3943	480.6074	1438.8004	1438.8045	-0.0041	1	(65)	0.00087	1		R.RHPEYAVSVLLR.L
3944	480.6074	1438.8004	1438.8045	-0.0041	1	(54)	0.0094	1		R.RHPEYAVSVLLR.L
3946	480.6075	1438.8007	1438.8045	-0.0038	1	(62)	0.0015	1		R.RHPEYAVSVLLR.L
3947	480.6076	1438.8010	1438.8045	-0.0035	1	(72)	0.00016	1		R.RHPEYAVSVLLR.L
3948	720.4092	1438.8038	1438.8045	-0.0006	1	(63)	0.0013	1		R.RHPEYAVSVLLR.L
3949	720.4095	1438.8044	1438.8045	-0.0000	1	(64)	0.0011	1		R.RHPEYAVSVLLR.L
3950	720.9019	1439.7892	1438.8045	0.9848	1	(63)	0.0014	1		R.RHPEYAVSVLLR.L
3951	720.9060	1439.7974	1438.8045	0.9930	1	(65)	0.00072	1		R.RHPEYAVSVLLR.L
3952	720.9064	1439.7982	1438.8045	0.9938	1	(62)	0.0015	1		R.RHPEYAVSVLLR.L
3953	720.9064	1439.7982	1438.8045	0.9938	1	(59)	0.0031	1		R.RHPEYAVSVLLR.L
3954	480.9405	1439.7997	1438.8045	0.9952	1	(63)	0.0014	1		R.RHPEYAVSVLLR.L
3957	480.9406	1439.8000	1438.8045	0.9955	1	(61)	0.002	1		R.RHPEYAVSVLLR.L
3958	720.9075	1439.8004	1438.8045	0.9960	1	(62)	0.0016	1		R.RHPEYAVSVLLR.L
3959	720.9077	1439.8008	1438.8045	0.9964	1	(60)	0.0026	1		R.RHPEYAVSVLLR.L
3960	720.9077	1439.8008	1438.8045	0.9964	1	(66)	0.0007	1		R.RHPEYAVSVLLR.L
3962	720.9078	1439.8010	1438.8045	0.9966	1	(65)	0.00079	1		R.RHPEYAVSVLLR.L
3963	720.9078	1439.8010	1438.8045	0.9966	1	(63)	0.0014	1		R.RHPEYAVSVLLR.L
3964	720.9080	1439.8014	1438.8045	0.9970	1	(68)	0.00043	1		R.RHPEYAVSVLLR.L
3965	720.9081	1439.8016	1438.8045	0.9972	1	(63)	0.0013	1		R.RHPEYAVSVLLR.L
3967	720.9086	1439.8026	1438.8045	0.9982	1	(63)	0.0014	1		R.RHPEYAVSVLLR.L
3968	720.9088	1439.8030	1438.8045	0.9986	1	(63)	0.0012	1		R.RHPEYAVSVLLR.L
3970	720.9109	1439.8072	1438.8045	1.0028	1	(63)	0.0012	1		R.RHPEYAVSVLLR.L
3978	722.3214	1442.6282	1442.6347	-0.0065	0	(74)	8.9e-05	1	U	K.YICDNQDTISSK.L
3979	722.3219	1442.6292	1442.6347	-0.0055	0	82	1.6e-05	1	U	K.YICDNQDTISSK.L
4085	1479.7863	1478.7790	1478.7881	-0.0091	0	(80)	2.7e-05	1		K.LGEYGFQNALIVR.Y
4086	740.3972	1478.7798	1478.7881	-0.0083	0	(105)	6.9e-08	1		K.LGEYGFQNALIVR.Y
4087	740.3972	1478.7798	1478.7881	-0.0083	0	(108)	3.4e-08	1		K.LGEYGFQNALIVR.Y
4088	740.3979	1478.7812	1478.7881	-0.0069	0	(102)	1.4e-07	1		K.LGEYGFQNALIVR.Y
4089	740.3981	1478.7816	1478.7881	-0.0065	0	(109)	3.3e-08	1		K.LGEYGFQNALIVR.Y
4090	740.3981	1478.7816	1478.7881	-0.0065	0	112	1.4e-08	1		K.LGEYGFQNALIVR.Y
4091	740.3983	1478.7820	1478.7881	-0.0061	0	(112)	1.7e-08	1		K.LGEYGFQNALIVR.Y
4092	740.3983	1478.7820	1478.7881	-0.0061	0	(105)	6.9e-08	1		K.LGEYGFQNALIVR.Y
4093	740.3985	1478.7824	1478.7881	-0.0057	0	(108)	3.8e-08	1		K.LGEYGFQNALIVR.Y
4094	740.3985	1478.7824	1478.7881	-0.0057	0	(111)	1.7e-08	1		K.LGEYGFQNALIVR.Y
4095	740.3985	1478.7824	1478.7881	-0.0057	0	(102)	1.4e-07	1		K.LGEYGFQNALIVR.Y
4096	1479.7900	1478.7827	1478.7881	-0.0054	0	(74)	9.1e-05	1		K.LGEYGFQNALIVR.Y
4097	740.3990	1478.7834	1478.7881	-0.0047	0	(90)	2.3e-06	1		K.LGEYGFQNALIVR.Y
4098	740.3992	1478.7838	1478.7881	-0.0043	0	(102)	1.4e-07	1		K.LGEYGFQNALIVR.Y
4099	740.3992	1478.7838	1478.7881	-0.0043	0	(109)	3.1e-08	1		K.LGEYGFQNALIVR.Y
4100	740.3995	1478.7844	1478.7881	-0.0037	0	(108)	3.9e-08	1		K.LGEYGFQNALIVR.Y
4101	740.3995	1478.7844	1478.7881	-0.0037	0	(106)	6.4e-08	1		K.LGEYGFQNALIVR.Y
4102	740.3997	1478.7848	1478.7881	-0.0033	0	(106)	6.4e-08	1		K.LGEYGFQNALIVR.Y
4103	740.4001	1478.7856	1478.7881	-0.0025	0	(82)	1.6e-05	1		K.LGEYGFQNALIVR.Y
4104	740.4001	1478.7856	1478.7881	-0.0025	0	(106)	6.4e-08	1		K.LGEYGFQNALIVR.Y
4105	740.4005	1478.7864	1478.7881	-0.0017	0	(106)	6.5e-08	1		K.LGEYGFQNALIVR.Y
4106	740.4006	1478.7866	1478.7881	-0.0015	0	(92)	1.6e-06	1		K.LGEYGFQNALIVR.Y
4107	740.4007	1478.7868	1478.7881	-0.0013	0	(103)	1.3e-07	1		K.LGEYGFQNALIVR.Y
4108	740.4011	1478.7876	1478.7881	-0.0005	0	(85)	7.7e-06	1		K.LGEYGFQNALIVR.Y
4109	740.4012	1478.7878	1478.7881	-0.0003	0	(90)	2.2e-06	1		K.LGEYGFQNALIVR.Y
4110	740.7166	1479.4186	1478.7881	0.6305	0	(90)	1.6e-06	1		K.LGEYGFQNALIVR.Y
4111	740.8925	1479.7704	1478.7881	0.9823	0	(78)	4.2e-05	1		K.LGEYGFQNALIVR.Y
4112	740.8944	1479.7742	1478.7881	0.9861	0	(74)	9.8e-05	1		K.LGEYGFQNALIVR.Y
4113	740.8987	1479.7828	1478.7881	0.9947	0	(83)	1.4e-05	1		K.LGEYGFQNALIVR.Y
4114	494.2688	1479.7846	1478.7881	0.9964	0	(56)	0.0064	1		K.LGEYGFQNALIVR.Y
4115	740.8997	1479.7848	1478.7881	0.9967	0	(87)	4.7e-06	1		K.LGEYGFQNALIVR.Y
4116	740.9005	1479.7864	1478.7881	0.9983	0	(87)	5.1e-06	1		K.LGEYGFQNALIVR.Y
4117	740.9005	1479.7864	1478.7881	0.9983	0	(81)	1.8e-05	1		K.LGEYGFQNALIVR.Y
4118	740.9011	1479.7876	1478.7881	0.9995	0	(79)	2.8e-05	1		K.LGEYGFQNALIVR.Y
4119	740.9017	1479.7888	1478.7881	1.0007	0	(79)	2.8e-05	1		K.LGEYGFQNALIVR.Y
4209	504.6163	1510.8271	1510.8355	-0.0085	0	(67)	0.00047	1		K.VPQVSTPTLVEVSR.S
4214	756.9231	1511.8316	1510.8355	0.9961	0	88	3.8e-06	1		K.VPQVSTPTLVEVSR.S
4252	766.8900	1531.7654	1531.7738	-0.0084	1	(58)	0.0042	1		K.LKECCDKPLLEK.S
4257	511.5960	1531.7662	1531.7738	-0.0076	1	(56)	0.0066	1		K.LKECCDKPLLEK.S
4258	511.5960	1531.7662	1531.7738	-0.0076	1	(61)	0.0019	1		K.LKECCDKPLLEK.S
4260	766.8904	1531.7662	1531.7738	-0.0076	1	(66)	0.00068	1		K.LKECCDKPLLEK.S
4261	511.5961	1531.7665	1531.7738	-0.0073	1	(59)	0.0032	1		K.LKECCDKPLLEK.S
4265	766.8911	1531.7676	1531.7738	-0.0062	1	68	0.00038	1		K.LKECCDKPLLEK.S
4266	766.8911	1531.7676	1531.7738	-0.0062	1	(59)	0.0034	1		K.LKECCDKPLLEK.S
4267	511.5965	1531.7677	1531.7738	-0.0061	1	(51)	0.019	1		K.LKECCDKPLLEK.S
4268	511.5967	1531.7683	1531.7738	-0.0055	1	(57)	0.0054	1		K.LKECCDKPLLEK.S

☒	4269	511.5968	1531.7686	1531.7738	-0.0052	1	(55)	0.0071	1		K.LKECCDKPLLEK.S
☒	4270	511.5968	1531.7686	1531.7738	-0.0052	1	(54)	0.011	1		K.LKECCDKPLLEK.S
☒	4291	770.4079	1538.8012	1538.8127	-0.0114	1	(66)	0.00065	1		R.LCVLHEKTPVSEK.V
☒	4292	513.9421	1538.8045	1538.8127	-0.0082	1	(63)	0.0012	1		R.LCVLHEKTPVSEK.V
☒	4295	513.9423	1538.8051	1538.8127	-0.0076	1	(63)	0.0012	1		R.LCVLHEKTPVSEK.V
☒	4296	513.9423	1538.8051	1538.8127	-0.0076	1	(58)	0.0034	1		R.LCVLHEKTPVSEK.V
☒	4297	770.4099	1538.8052	1538.8127	-0.0074	1	(71)	0.00019	1		R.LCVLHEKTPVSEK.V
☒	4299	513.9424	1538.8054	1538.8127	-0.0073	1	(63)	0.0013	1		R.LCVLHEKTPVSEK.V
☒	4300	770.4100	1538.8054	1538.8127	-0.0072	1	(51)	0.021	1		R.LCVLHEKTPVSEK.V
☒	4301	770.4100	1538.8054	1538.8127	-0.0072	1	71	0.00017	1		R.LCVLHEKTPVSEK.V
☒	4304	385.7087	1538.8057	1538.8127	-0.0070	1	(50)	0.022	1		R.LCVLHEKTPVSEK.V
☒	4305	513.9426	1538.8060	1538.8127	-0.0067	1	(62)	0.0014	1		R.LCVLHEKTPVSEK.V
☒	4306	770.4106	1538.8066	1538.8127	-0.0060	1	(71)	0.0002	1		R.LCVLHEKTPVSEK.V
☒	4307	770.4116	1538.8086	1538.8127	-0.0040	1	(59)	0.003	1		R.LCVLHEKTPVSEK.V
☒	4309	770.9101	1539.8056	1538.8127	0.9930	1	(54)	0.0088	1		R.LCVLHEKTPVSEK.V
☒	4311	514.2764	1539.8074	1538.8127	0.9947	1	(62)	0.0015	1		R.LCVLHEKTPVSEK.V
☒	4383	1567.7288	1566.7215	1566.7354	-0.0139	0	94	8.3e-07	1	U	K.DAFLGSFLYEYSR.R
☒	4384	1567.7314	1566.7241	1566.7354	-0.0113	0	(91)	1.7e-06	1	U	K.DAFLGSFLYEYSR.R
☒	4385	523.2493	1566.7261	1566.7354	-0.0094	0	(64)	0.00088	1	U	K.DAFLGSFLYEYSR.R
☒	4386	784.3707	1566.7268	1566.7354	-0.0086	0	(63)	0.0012	1	U	K.DAFLGSFLYEYSR.R
☒	4387	784.3709	1566.7272	1566.7354	-0.0082	0	(71)	0.0002	1	U	K.DAFLGSFLYEYSR.R
☒	4388	784.3712	1566.7278	1566.7354	-0.0076	0	(71)	0.00021	1	U	K.DAFLGSFLYEYSR.R
☒	4389	784.3713	1566.7280	1566.7354	-0.0074	0	(66)	0.00053	1	U	K.DAFLGSFLYEYSR.R
☒	4390	784.3717	1566.7288	1566.7354	-0.0066	0	(71)	0.0002	1	U	K.DAFLGSFLYEYSR.R
☒	4391	784.3718	1566.7290	1566.7354	-0.0064	0	(73)	0.00011	1	U	K.DAFLGSFLYEYSR.R
☒	4392	784.3718	1566.7290	1566.7354	-0.0064	0	(67)	0.00048	1	U	K.DAFLGSFLYEYSR.R
☒	4394	784.3719	1566.7292	1566.7354	-0.0062	0	(55)	0.0082	1	U	K.DAFLGSFLYEYSR.R
☒	4395	784.3719	1566.7292	1566.7354	-0.0062	0	(70)	0.00022	1	U	K.DAFLGSFLYEYSR.R
☒	4396	784.3719	1566.7292	1566.7354	-0.0062	0	(75)	6.8e-05	1	U	K.DAFLGSFLYEYSR.R
☒	4397	784.3719	1566.7292	1566.7354	-0.0062	0	(75)	6.7e-05	1	U	K.DAFLGSFLYEYSR.R
☒	4398	784.3720	1566.7294	1566.7354	-0.0060	0	(63)	0.0012	1	U	K.DAFLGSFLYEYSR.R
☒	4399	784.3721	1566.7296	1566.7354	-0.0058	0	(70)	0.00021	1	U	K.DAFLGSFLYEYSR.R
☒	4400	784.3721	1566.7296	1566.7354	-0.0058	0	(71)	0.0002	1	U	K.DAFLGSFLYEYSR.R
☒	4401	784.3722	1566.7298	1566.7354	-0.0056	0	(67)	0.00051	1	U	K.DAFLGSFLYEYSR.R
☒	4402	784.3723	1566.7300	1566.7354	-0.0054	0	(67)	0.0005	1	U	K.DAFLGSFLYEYSR.R
☒	4403	784.3724	1566.7302	1566.7354	-0.0052	0	(75)	6.9e-05	1	U	K.DAFLGSFLYEYSR.R
☒	4404	784.3724	1566.7302	1566.7354	-0.0052	0	(75)	7e-05	1	U	K.DAFLGSFLYEYSR.R
☒	4405	784.3725	1566.7304	1566.7354	-0.0050	0	(70)	0.00021	1	U	K.DAFLGSFLYEYSR.R
☒	4406	784.3725	1566.7304	1566.7354	-0.0050	0	(71)	0.0002	1	U	K.DAFLGSFLYEYSR.R
☒	4407	784.3725	1566.7304	1566.7354	-0.0050	0	(66)	0.00054	1	U	K.DAFLGSFLYEYSR.R
☒	4408	784.3725	1566.7304	1566.7354	-0.0050	0	(63)	0.0012	1	U	K.DAFLGSFLYEYSR.R
☒	4409	784.3726	1566.7306	1566.7354	-0.0048	0	(61)	0.0019	1	U	K.DAFLGSFLYEYSR.R
☒	4410	784.3726	1566.7306	1566.7354	-0.0048	0	(66)	0.00064	1	U	K.DAFLGSFLYEYSR.R
☒	4411	784.3726	1566.7306	1566.7354	-0.0048	0	(81)	1.7e-05	1	U	K.DAFLGSFLYEYSR.R
☒	4412	784.3726	1566.7306	1566.7354	-0.0048	0	(75)	6.7e-05	1	U	K.DAFLGSFLYEYSR.R
☒	4413	784.3727	1566.7308	1566.7354	-0.0046	0	(50)	0.023	1	U	K.DAFLGSFLYEYSR.R
☒	4414	784.3727	1566.7308	1566.7354	-0.0046	0	(71)	0.0002	1	U	K.DAFLGSFLYEYSR.R
☒	4415	784.3729	1566.7312	1566.7354	-0.0042	0	(63)	0.0012	1	U	K.DAFLGSFLYEYSR.R
☒	4416	784.3731	1566.7316	1566.7354	-0.0038	0	(66)	0.00053	1	U	K.DAFLGSFLYEYSR.R
☒	4417	784.3733	1566.7320	1566.7354	-0.0034	0	(71)	0.00021	1	U	K.DAFLGSFLYEYSR.R
☒	4418	784.3734	1566.7322	1566.7354	-0.0032	0	(71)	0.00018	1	U	K.DAFLGSFLYEYSR.R
☒	4419	784.3735	1566.7324	1566.7354	-0.0030	0	(70)	0.00021	1	U	K.DAFLGSFLYEYSR.R
☒	4420	784.3737	1566.7328	1566.7354	-0.0026	0	(66)	0.00054	1	U	K.DAFLGSFLYEYSR.R
☒	4421	784.3741	1566.7336	1566.7354	-0.0018	0	(71)	0.00019	1	U	K.DAFLGSFLYEYSR.R
☒	4422	784.3754	1566.7362	1566.7354	0.0008	0	(75)	7.1e-05	1	U	K.DAFLGSFLYEYSR.R
☒	4427	1568.7373	1567.7300	1566.7354	0.9946	0	(50)	0.026	1	U	K.DAFLGSFLYEYSR.R
☒	4428	784.8735	1567.7324	1566.7354	0.9970	0	(70)	0.00024	1	U	K.DAFLGSFLYEYSR.R
☒	4429	784.8735	1567.7324	1566.7354	0.9970	0	(59)	0.0027	1	U	K.DAFLGSFLYEYSR.R
☒	4430	784.8740	1567.7334	1566.7354	0.9980	0	(66)	0.00059	1	U	K.DAFLGSFLYEYSR.R
☒	4431	784.8746	1567.7346	1566.7354	0.9992	0	(56)	0.0053	1	U	K.DAFLGSFLYEYSR.R
☒	4432	784.8747	1567.7348	1566.7354	0.9994	0	(61)	0.0018	1	U	K.DAFLGSFLYEYSR.R
☒	4453	788.8835	1575.7524	1575.7603	-0.0078	0	(61)	0.002	1	U	K.LKPDPNTLCDEFK.A
☒	4454	788.8835	1575.7524	1575.7603	-0.0078	0	82	1.6e-05	1	U	K.LKPDPNTLCDEFK.A
☒	4459	526.5929	1576.7569	1575.7603	0.9966	0	(49)	0.031	1	U	K.LKPDPNTLCDEFK.A
☒	4611	547.3131	1638.9175	1638.9305	-0.0130	1	(88)	3.9e-06	1		R.KVPQVSTPTLVEVSR.S
☒	4612	547.3142	1638.9208	1638.9305	-0.0097	1	(84)	8e-06	1		R.KVPQVSTPTLVEVSR.S
☒	4613	547.3143	1638.9211	1638.9305	-0.0094	1	(78)	3.7e-05	1		R.KVPQVSTPTLVEVSR.S
☒	4614	547.3143	1638.9211	1638.9305	-0.0094	1	(76)	5.5e-05	1		R.KVPQVSTPTLVEVSR.S
☒	4615	547.3144	1638.9214	1638.9305	-0.0091	1	(94)	8e-07	1		R.KVPQVSTPTLVEVSR.S
☒	4616	547.3144	1638.9214	1638.9305	-0.0091	1	(78)	3.6e-05	1		R.KVPQVSTPTLVEVSR.S
☒	4617	820.4681	1638.9216	1638.9305	-0.0088	1	(125)	6.9e-10	1		R.KVPQVSTPTLVEVSR.S
☒	4618	820.4681	1638.9216	1638.9305	-0.0088	1	(105)	7.6e-08	1		R.KVPQVSTPTLVEVSR.S
☒	4619	820.4681	1638.9216	1638.9305	-0.0088	1	125	6.8e-10	1		R.KVPQVSTPTLVEVSR.S
☒	4620	547.3148	1638.9226	1638.9305	-0.0079	1	(67)	0.00044	1		R.KVPQVSTPTLVEVSR.S
☒	4621	547.3148	1638.9226	1638.9305	-0.0079	1	(74)	9.2e-05	1		R.KVPQVSTPTLVEVSR.S
☒	4622	547.3148	1638.9226	1638.9305	-0.0079	1	(87)	4.3e-06	1		R.KVPQVSTPTLVEVSR.S
☒	4623	820.4686	1638.9226	1638.9305	-0.0078	1	(125)	6.8e-10	1		R.KVPQVSTPTLVEVSR.S
☒	4624	547.3149	1638.9229	1638.9305	-0.0076	1	(66)	0.00057	1		R.KVPQVSTPTLVEVSR.S
☒	4625	547.3149	1638.9229	1638.9305	-0.0076	1	(85)	6.6e-06	1		R.KVPQVSTPTLVEVSR.S
☒	4626	547.3149	1638.9229	1638.9305	-0.0076	1	(89)	2.6e-06	1		R.KVPQVSTPTLVEVSR.S
☒	4627	547.3149	1638.9229	1638.9305	-0.0076	1	(97)	5e-07	1		R.KVPQVSTPTLVEVSR.S
☒	4628	547.3149	1638.9229	1638.9305	-0.0076	1	(92)	1.5e-06	1		R.KVPQVSTPTLVEVSR.S
☒	4629	820.4688	1638.9230	1638.9305	-0.0074	1	(120)	2.2e-09	1		R.KVPQVSTPTLVEVSR.S
☒	4630	547.3150	1638.9232	1638.9305	-0.0073	1	(89)	2.7e-06	1		R.KVPQVSTPTLVEVSR.S
☒	4631	547.3150	1638.9232	1638.9305	-0.0073	1	(85)	7.3e-06	1		R.KVPQVSTPTLVEVSR.S
☒	4632	820.4689	1638.9232	1638.9305	-0.0072	1	(120)	2.2e-09	1		R.KVPQVSTPTLVEVSR.S
☒	4633	820.4690	1638.9234	1638.9305	-0.0070	1	(125)	6.8e-10	1		R.KVPQVSTPTLVEVSR.S
☒	4634	547.3151	1638.9235	1638.9305	-0.0070	1	(86)	5.7e-06	1		R.KVPQVSTPTLVEVSR.S
☒	4635	547.3151	1638.9235	1638.9305	-0.0070	1	(85)	6.8e-06	1		R.KVPQVSTPTLVEVSR.S
☒	4636	547.3151	1638.9235	1638.9305	-0.0070	1	(88)	3.8e-06	1		R.KVPQVSTPTLVEVSR.S
☒	4637	820.4691	1638.9236	1638.9305	-0.0068	1	(120)	2.3e-09	1		R.KVPQVSTPTLVEVSR.S
☒	4638	547.3152	1638.9238	1638.9305	-0.0067	1	(98)	3.7e-07	1		R.KVPQVSTPTLVEVSR.S
☒	4639	547.3152	1638.9238	1638.9305	-0.0067	1	(97)	4.1e-07	1		R.KVPQVSTPTLVEVSR.S
☒	4640	547.3152	1638.9238	1638.9305	-0.0067	1	(90)	2.4e-06	1		R.KVPQVSTPTLVEVSR.S
☒	4641	547.3153	1638.9241	1638.9305	-0.0064	1	(77)	4.3e-05	1		R.KVPQVSTPTLVEVSR.S
☒	4642	547.3153	1638.9241	1638.9305	-0.0064	1	(90)	2.2e-06	1		R.KVPQVSTPTLVEVSR.S
☒	4643	547.3153	1638.9241	1638.9305	-0.0064	1	(76)	5.2e-05	1		R.KVPQVSTPTLVEVSR.S
☒	4644	547.3153	1638.9241	1638.9305	-0.0064	1	(94)	8.7e-07	1		R.KVPQVSTPTLVEVSR.S
☒	4645	547.3153	1638.9241	1638.9305	-0.0064	1	(84)	8.4e-06	1		R.KVPQVSTPTLVEVSR.S
☒	4646	547.3153	1638.9241	1638.9305	-0.0064	1	(90)	2.5e-06	1		R.KVPQVSTPTLVEVSR.S

☒	4647	820.4694	1638.9242	1638.9305	-0.0062	1	(125)	7.8e-10	1		R.KVPQVSTPTLVEVSR.S
☒	4648	820.4696	1638.9246	1638.9305	-0.0058	1	(125)	6.9e-10	1		R.KVPQVSTPTLVEVSR.S
☒	4649	547.3155	1638.9247	1638.9305	-0.0058	1	(88)	4e-06	1		R.KVPQVSTPTLVEVSR.S
☒	4650	820.4698	1638.9250	1638.9305	-0.0054	1	(65)	0.00072	1		R.KVPQVSTPTLVEVSR.S
☒	4651	547.3157	1638.9253	1638.9305	-0.0052	1	(89)	2.8e-06	1		R.KVPQVSTPTLVEVSR.S
☒	4652	547.3160	1638.9262	1638.9305	-0.0043	1	(89)	2.8e-06	1		R.KVPQVSTPTLVEVSR.S
☒	4653	547.3162	1638.9268	1638.9305	-0.0037	1	(87)	4.8e-06	1		R.KVPQVSTPTLVEVSR.S
☒	4655	820.7706	1639.5266	1638.9305	0.5962	1	(123)	7.5e-10	1		R.KVPQVSTPTLVEVSR.S
☒	4656	820.9695	1639.9244	1638.9305	0.9940	1	(104)	9.4e-08	1		R.KVPQVSTPTLVEVSR.S
☒	4657	547.6488	1639.9246	1638.9305	0.9941	1	(77)	4.8e-05	1		R.KVPQVSTPTLVEVSR.S
☒	4658	820.9699	1639.9252	1638.9305	0.9948	1	(104)	8.9e-08	1		R.KVPQVSTPTLVEVSR.S
☒	4659	547.6491	1639.9255	1638.9305	0.9950	1	(98)	3.9e-07	1		R.KVPQVSTPTLVEVSR.S
☒	4660	547.6492	1639.9258	1638.9305	0.9953	1	(81)	1.7e-05	1		R.KVPQVSTPTLVEVSR.S
☒	4661	820.9703	1639.9260	1638.9305	0.9956	1	(104)	8.8e-08	1		R.KVPQVSTPTLVEVSR.S
☒	4662	547.6494	1639.9264	1638.9305	0.9959	1	(83)	1.1e-05	1		R.KVPQVSTPTLVEVSR.S
☒	4663	547.6494	1639.9264	1638.9305	0.9959	1	(85)	7e-06	1		R.KVPQVSTPTLVEVSR.S
☒	4664	820.9708	1639.9270	1638.9305	0.9966	1	(104)	9.3e-08	1		R.KVPQVSTPTLVEVSR.S
☒	4665	820.9711	1639.9276	1638.9305	0.9972	1	(104)	1e-07	1		R.KVPQVSTPTLVEVSR.S
☒	4666	547.6499	1639.9279	1638.9305	0.9974	1	(63)	0.0012	1		R.KVPQVSTPTLVEVSR.S
☒	4667	547.6499	1639.9279	1638.9305	0.9974	1	(96)	6.1e-07	1		R.KVPQVSTPTLVEVSR.S
☒	4745	842.9105	1683.8064	1683.8138	-0.0073	1	78	3.2e-05	1	U	K.YICDNQDTISSKLK.E
☒	4752	843.4115	1684.8084	1683.8138	0.9947	1	(63)	0.0011	1	U	K.YICDNQDTISSKLK.E
☒	4769	846.9695	1691.9244	1691.9346	-0.0101	1	(99)	3e-07	1	U	K.AEFVEVTKLVTDLTK.V
☒	4770	564.9822	1691.9248	1691.9346	-0.0098	1	(63)	0.0011	1	U	K.AEFVEVTKLVTDLTK.V
☒	4771	564.9822	1691.9248	1691.9346	-0.0098	1	(80)	2.4e-05	1	U	K.AEFVEVTKLVTDLTK.V
☒	4772	564.9822	1691.9248	1691.9346	-0.0098	1	(63)	0.0011	1	U	K.AEFVEVTKLVTDLTK.V
☒	4773	846.9699	1691.9252	1691.9346	-0.0093	1	(95)	7.2e-07	1	U	K.AEFVEVTKLVTDLTK.V
☒	4774	564.9827	1691.9263	1691.9346	-0.0083	1	(67)	0.00042	1	U	K.AEFVEVTKLVTDLTK.V
☒	4775	564.9827	1691.9263	1691.9346	-0.0083	1	(72)	0.00015	1	U	K.AEFVEVTKLVTDLTK.V
☒	4776	564.9830	1691.9272	1691.9346	-0.0074	1	(75)	6.4e-05	1	U	K.AEFVEVTKLVTDLTK.V
☒	4777	846.9709	1691.9272	1691.9346	-0.0073	1	(99)	2.9e-07	1	U	K.AEFVEVTKLVTDLTK.V
☒	4778	846.9709	1691.9272	1691.9346	-0.0073	1	(99)	3e-07	1	U	K.AEFVEVTKLVTDLTK.V
☒	4779	846.9711	1691.9276	1691.9346	-0.0069	1	(99)	2.9e-07	1	U	K.AEFVEVTKLVTDLTK.V
☒	4780	846.9711	1691.9276	1691.9346	-0.0069	1	99	2.8e-07	1	U	K.AEFVEVTKLVTDLTK.V
☒	4781	564.9832	1691.9278	1691.9346	-0.0068	1	(63)	0.0011	1	U	K.AEFVEVTKLVTDLTK.V
☒	4782	564.9832	1691.9278	1691.9346	-0.0068	1	(72)	0.00015	1	U	K.AEFVEVTKLVTDLTK.V
☒	4783	846.9712	1691.9278	1691.9346	-0.0067	1	(81)	1.9e-05	1	U	K.AEFVEVTKLVTDLTK.V
☒	4785	564.9833	1691.9281	1691.9346	-0.0065	1	(67)	0.00042	1	U	K.AEFVEVTKLVTDLTK.V
☒	4786	846.9715	1691.9284	1691.9346	-0.0061	1	(99)	3e-07	1	U	K.AEFVEVTKLVTDLTK.V
☒	4787	564.9835	1691.9287	1691.9346	-0.0059	1	(80)	2.4e-05	1	U	K.AEFVEVTKLVTDLTK.V
☒	4788	846.9717	1691.9288	1691.9346	-0.0057	1	(98)	3.4e-07	1	U	K.AEFVEVTKLVTDLTK.V
☒	4789	564.9836	1691.9290	1691.9346	-0.0056	1	(85)	7.5e-06	1	U	K.AEFVEVTKLVTDLTK.V
☒	4790	564.9838	1691.9296	1691.9346	-0.0050	1	(56)	0.0057	1	U	K.AEFVEVTKLVTDLTK.V
☒	4792	564.9843	1691.9311	1691.9346	-0.0035	1	(67)	0.0004	1	U	K.AEFVEVTKLVTDLTK.V
☒	4800	847.4713	1692.9280	1691.9346	0.9935	1	(89)	2.8e-06	1	U	K.AEFVEVTKLVTDLTK.V
☒	4801	565.3170	1692.9292	1691.9346	0.9946	1	(64)	0.00093	1	U	K.AEFVEVTKLVTDLTK.V
☒	4802	565.3170	1692.9292	1691.9346	0.9946	1	(85)	7.6e-06	1	U	K.AEFVEVTKLVTDLTK.V
☒	4803	565.3174	1692.9304	1691.9346	0.9958	1	(52)	0.014	1	U	K.AEFVEVTKLVTDLTK.V
☒	4804	565.3174	1692.9304	1691.9346	0.9958	1	(84)	7.9e-06	1	U	K.AEFVEVTKLVTDLTK.V
☒	4805	565.3174	1692.9304	1691.9346	0.9958	1	(59)	0.0025	1	U	K.AEFVEVTKLVTDLTK.V
☒	4806	565.3176	1692.9310	1691.9346	0.9964	1	(63)	0.0011	1	U	K.AEFVEVTKLVTDLTK.V
☒	4807	847.4730	1692.9314	1691.9346	0.9969	1	(64)	0.00087	1	U	K.AEFVEVTKLVTDLTK.V
☒	4838	862.9150	1723.8154	1723.8273	-0.0119	0	(95)	6.9e-07	1		R.MPCTEDYLSLILNR.L
☒	4840	575.6136	1723.8190	1723.8273	-0.0083	0	(50)	0.02	1		R.MPCTEDYLSLILNR.L
☒	4841	862.9170	1723.8194	1723.8273	-0.0079	0	(92)	1.4e-06	1		R.MPCTEDYLSLILNR.L
☒	4842	862.9171	1723.8196	1723.8273	-0.0077	0	(98)	3.5e-07	1		R.MPCTEDYLSLILNR.L
☒	4843	862.9174	1723.8202	1723.8273	-0.0071	0	(84)	9.1e-06	1		R.MPCTEDYLSLILNR.L
☒	4844	575.6141	1723.8205	1723.8273	-0.0068	0	(49)	0.026	1		R.MPCTEDYLSLILNR.L
☒	4845	862.9176	1723.8206	1723.8273	-0.0067	0	(99)	3e-07	1		R.MPCTEDYLSLILNR.L
☒	4846	862.9180	1723.8214	1723.8273	-0.0059	0	(92)	1.4e-06	1		R.MPCTEDYLSLILNR.L
☒	4847	862.9180	1723.8214	1723.8273	-0.0059	0	(84)	8.2e-06	1		R.MPCTEDYLSLILNR.L
☒	4848	862.9181	1723.8216	1723.8273	-0.0057	0	99	3e-07	1		R.MPCTEDYLSLILNR.L
☒	4849	862.9182	1723.8218	1723.8273	-0.0055	0	(86)	5e-06	1		R.MPCTEDYLSLILNR.L
☒	4850	862.9182	1723.8218	1723.8273	-0.0055	0	(78)	3.6e-05	1		R.MPCTEDYLSLILNR.L
☒	4851	862.9191	1723.8236	1723.8273	-0.0037	0	(73)	0.00012	1		R.MPCTEDYLSLILNR.L
☒	4857	863.4149	1724.8152	1723.8273	0.9879	0	(78)	3.5e-05	1		R.MPCTEDYLSLILNR.L
☒	4861	863.4186	1724.8226	1723.8273	0.9953	0	(80)	1.9e-05	1		R.MPCTEDYLSLILNR.L
☒	4883	580.9448	1739.8126	1739.8222	-0.0096	0	(51)	0.016	1		R.MPCTEDYLSLILNR.L + Oxidation (M)
☒	5023	907.9128	1813.8110	1813.8226	-0.0115	1	(61)	0.0016	1	U	R.LAKEYEATLEECCAK.D
☒	5025	907.9162	1813.8178	1813.8226	-0.0047	1	75	6.5e-05	1	U	R.LAKEYEATLEECCAK.D
☒	5027	605.9465	1814.8177	1813.8226	0.9951	1	(47)	0.04	1	U	R.LAKEYEATLEECCAK.D
☒	5184	629.9760	1886.9062	1887.9195	-1.0134	0	47	0.043	1		R.HPYFYAPELLYYANK.Y
☒	5206	634.6254	1900.8544	1900.8625	-0.0081	1	48	0.028	1	U	R.NECFLSHKDDSPDLPK.L
☒	5214	636.6421	1906.9045	1906.9135	-0.0090	0	(46)	0.049	1	U	K.LFTFHADICTLPDTEK.Q
☒	5215	636.6423	1906.9051	1906.9135	-0.0084	0	(52)	0.013	1	U	K.LFTFHADICTLPDTEK.Q
☒	5216	636.6429	1906.9069	1906.9135	-0.0066	0	(54)	0.0072	1	U	K.LFTFHADICTLPDTEK.Q
☒	5217	954.9598	1907.9050	1906.9135	0.9915	0	76	4.7e-05	1	U	K.LFTFHADICTLPDTEK.Q
☒	5219	636.9761	1907.9065	1906.9135	0.9930	0	(52)	0.014	1	U	K.LFTFHADICTLPDTEK.Q
☒	5220	636.9765	1907.9077	1906.9135	0.9942	0	(54)	0.0078	1	U	K.LFTFHADICTLPDTEK.Q
☒	5221	636.9774	1907.9104	1906.9135	0.9969	0	(47)	0.039	1	U	K.LFTFHADICTLPDTEK.Q
☒	5222	954.9625	1907.9104	1906.9135	0.9969	0	(63)	0.00099	1	U	K.LFTFHADICTLPDTEK.Q
☒	5247	964.3969	1926.7792	1926.7910	-0.0118	1	(136)	4.6e-11	1	U	K.CCAADDKEACFAVEGPK.L
☒	5248	964.3976	1926.7806	1926.7910	-0.0104	1	(135)	4.9e-11	1	U	K.CCAADDKEACFAVEGPK.L
☒	5249	964.3984	1926.7822	1926.7910	-0.0088	1	(125)	5e-10	1	U	K.CCAADDKEACFAVEGPK.L
☒	5250	643.2681	1926.7825	1926.7910	-0.0085	1	(80)	1.7e-05	1	U	K.CCAADDKEACFAVEGPK.L
☒	5251	964.3987	1926.7828	1926.7910	-0.0082	1	144	7.1e-12	1	U	K.CCAADDKEACFAVEGPK.L
☒	5252	643.6022	1927.7848	1926.7910	0.9938	1	(50)	0.015	1	U	K.CCAADDKEACFAVEGPK.L
☒	5253	643.6035	1927.7887	1926.7910	0.9977	1	(78)	2.8e-05	1	U	K.CCAADDKEACFAVEGPK.L
☒	5271	973.5074	1945.0002	1945.0091	-0.0089	1	(92)	1.2e-06	1	U	K.SLHTLFGDELCKVASLR.E
☒	5272	649.3412	1945.0018	1945.0091	-0.0074	1	(87)	3.8e-06	1	U	K.SLHTLFGDELCKVASLR.E
☒	5273	649.3415	1945.0027	1945.0091	-0.0065	1	104	8.1e-08	1	U	K.SLHTLFGDELCKVASLR.E
☒	5274	973.5129	1945.0112	1945.0091	0.0021	1	(94)	8.3e-07	1	U	K.SLHTLFGDELCKVASLR.E
☒	5278	487.5081	1946.0033	1945.0091	0.9942	1	(53)	0.0095	1	U	K.SLHTLFGDELCKVASLR.E
☒	5279	487.5083	1946.0041	1945.0091	0.9950	1	(67)	0.00037	1	U	K.SLHTLFGDELCKVASLR.E
☒	5280	649.6754	1946.0044	1945.0091	0.9952	1	(90)	1.9e-06	1	U	K.SLHTLFGDELCKVASLR.E
☒	5281	487.5087	1946.0057	1945.0091	0.9966	1	(63)	0.001	1	U	K.SLHTLFGDELCKVASLR.E
☒	5282	487.5091	1946.0073	1945.0091	0.9982	1	(49)	0.024	1	U	K.SLHTLFGDELCKVASLR.E
☒	5353	1003.4213	2004.8280	2003.8387	0.9894	1	74	6.4e-05	1	U	K.VASLRETYGDMADCCEK.Q
☒	5373	1010.4843	2018.9540	2018.9619	-0.0078	1	99	2.3e-07	1	U	K.LKPDPNTLCDEFKADEK.K
☒	5374	673.9924	2018.9554	2018.9619	-0.0065	1	(75)	6.5e-05	1	U	K.LKPDPNTLCDEFKADEK.K
☒	5375	673.9929	2018.9569	2018.9619	-0.0050	1	(91)	1.6e-06	1	U	K.LKPDPNTLCDEFKADEK.K

5376	673.9943	2018.9611	2018.9619	-0.0008	1	(75)	6.5e-05	1	U	K.LKPDPNTLCDEFKADEK.K
5380	674.3177	2019.9313	2018.9619	0.9694	1	(49)	0.024	1	U	K.LKPDPNTLCDEFKADEK.K
5381	505.9924	2019.9405	2018.9619	0.9786	1	(46)	0.043	1	U	K.LKPDPNTLCDEFKADEK.K
5382	674.3214	2019.9424	2018.9619	0.9805	1	(72)	0.00011	1	U	K.LKPDPNTLCDEFKADEK.K
5383	1010.9821	2019.9496	2018.9619	0.9878	1	(82)	1.2e-05	1	U	K.LKPDPNTLCDEFKADEK.K
5384	674.3242	2019.9508	2018.9619	0.9889	1	(71)	0.00016	1	U	K.LKPDPNTLCDEFKADEK.K
5386	674.3251	2019.9535	2018.9619	0.9916	1	(78)	2.9e-05	1	U	K.LKPDPNTLCDEFKADEK.K
5387	505.9957	2019.9537	2018.9619	0.9918	1	(47)	0.039	1	U	K.LKPDPNTLCDEFKADEK.K
5388	505.9957	2019.9537	2018.9619	0.9918	1	(65)	0.00064	1	U	K.LKPDPNTLCDEFKADEK.K
5389	1010.9845	2019.9544	2018.9619	0.9926	1	(81)	1.6e-05	1	U	K.LKPDPNTLCDEFKADEK.K
5390	505.9959	2019.9545	2018.9619	0.9926	1	(50)	0.018	1	U	K.LKPDPNTLCDEFKADEK.K
5391	505.9959	2019.9545	2018.9619	0.9926	1	(55)	0.006	1	U	K.LKPDPNTLCDEFKADEK.K
5392	505.9960	2019.9549	2018.9619	0.9930	1	(49)	0.023	1	U	K.LKPDPNTLCDEFKADEK.K
5393	505.9960	2019.9549	2018.9619	0.9930	1	(53)	0.0089	1	U	K.LKPDPNTLCDEFKADEK.K
5394	674.3256	2019.9550	2018.9619	0.9931	1	(74)	6.8e-05	1	U	K.LKPDPNTLCDEFKADEK.K
5395	674.3256	2019.9550	2018.9619	0.9931	1	(74)	7e-05	1	U	K.LKPDPNTLCDEFKADEK.K
5396	1010.9849	2019.9552	2018.9619	0.9934	1	(92)	1.2e-06	1	U	K.LKPDPNTLCDEFKADEK.K
5397	674.3259	2019.9559	2018.9619	0.9940	1	(59)	0.0023	1	U	K.LKPDPNTLCDEFKADEK.K
5445	1023.0097	2044.0048	2044.0206	-0.0158	1	(48)	0.026	1		R.RHPYFYAPELLYYANK.Y
5450	1023.0146	2044.0146	2044.0206	-0.0060	1	(51)	0.013	1		R.RHPYFYAPELLYYANK.Y
5470	1023.5169	2045.0192	2044.0206	0.9986	1	52	0.012	1		R.RHPYFYAPELLYYANK.Y
5618	1100.0486	2198.0826	2198.0929	-0.0103	1	(88)	2.8e-06	1	U	K.ATEEQLKTVMENFVAFVDK.C
5619	1100.0490	2198.0834	2198.0929	-0.0095	1	102	9.5e-08	1	U	K.ATEEQLKTVMENFVAFVDK.C
5620	733.7052	2198.0938	2198.0929	0.0009	1	(85)	5.7e-06	1	U	K.ATEEQLKTVMENFVAFVDK.C
5624	1100.5499	2199.0852	2198.0929	0.9923	1	(88)	2.8e-06	1	U	K.ATEEQLKTVMENFVAFVDK.C
5626	734.0358	2199.0856	2198.0929	0.9927	1	(57)	0.0037	1	U	K.ATEEQLKTVMENFVAFVDK.C
5627	734.0358	2199.0856	2198.0929	0.9927	1	(89)	1.9e-06	1	U	K.ATEEQLKTVMENFVAFVDK.C
5628	734.0372	2199.0898	2198.0929	0.9969	1	(64)	0.00068	1	U	K.ATEEQLKTVMENFVAFVDK.C
5629	734.0372	2199.0898	2198.0929	0.9969	1	(71)	0.00014	1	U	K.ATEEQLKTVMENFVAFVDK.C
5631	734.0389	2199.0949	2198.0929	1.0020	1	(63)	0.00085	1	U	K.ATEEQLKTVMENFVAFVDK.C
5673	1124.4695	2246.9244	2246.9354	-0.0110	1	78	2.5e-05	1		K.ECCHGDLLECADDRADLAK.Y
5678	1124.9740	2247.9334	2246.9354	0.9980	1	(67)	0.00031	1		K.ECCHGDLLECADDRADLAK.Y
5679	750.3186	2247.9340	2246.9354	0.9985	1	(46)	0.036	1		K.ECCHGDLLECADDRADLAK.Y
5697	759.7164	2276.1274	2276.1511	-0.0237	1	(49)	0.022	1	U	K.LFTFHADICTLPDTEKQIK.K
5708	1139.5789	2277.1432	2276.1511	0.9921	1	(75)	4.5e-05	1	U	K.LFTFHADICTLPDTEKQIK.K
5711	1139.5809	2277.1472	2276.1511	0.9961	1	79	2.2e-05	1	U	K.LFTFHADICTLPDTEKQIK.K
5734	1151.0381	2300.0616	2300.0749	-0.0133	1	131	1.1e-10	1	U	K.NYQEAKDAFLGSFLYEYSR.R
5735	767.6964	2300.0674	2300.0749	-0.0075	1	(86)	3.6e-06	1	U	K.NYQEAKDAFLGSFLYEYSR.R
5737	768.0284	2301.0634	2300.0749	0.9885	1	(87)	3.5e-06	1	U	K.NYQEAKDAFLGSFLYEYSR.R
5738	768.0288	2301.0646	2300.0749	0.9897	1	(89)	2.1e-06	1	U	K.NYQEAKDAFLGSFLYEYSR.R
5739	768.0291	2301.0655	2300.0749	0.9906	1	(98)	2.8e-07	1	U	K.NYQEAKDAFLGSFLYEYSR.R
5740	768.0292	2301.0658	2300.0749	0.9909	1	(111)	1.2e-08	1	U	K.NYQEAKDAFLGSFLYEYSR.R
5741	768.0298	2301.0676	2300.0749	0.9927	1	(90)	1.5e-06	1	U	K.NYQEAKDAFLGSFLYEYSR.R
5742	768.0303	2301.0691	2300.0749	0.9942	1	(73)	7.2e-05	1	U	K.NYQEAKDAFLGSFLYEYSR.R
5743	1151.5422	2301.0698	2300.0749	0.9949	1	(115)	5.6e-09	1	U	K.NYQEAKDAFLGSFLYEYSR.R
5744	768.0308	2301.0706	2300.0749	0.9957	1	(86)	4.2e-06	1	U	K.NYQEAKDAFLGSFLYEYSR.R
5776	785.7141	2354.1205	2354.1325	-0.0120	1	(57)	0.0031	1	U	K.HLVDEPQNLIKQNCDQFEK.L
5777	785.7141	2354.1205	2354.1325	-0.0120	1	(70)	0.00015	1	U	K.HLVDEPQNLIKQNCDQFEK.L
5778	589.5380	2354.1229	2354.1325	-0.0096	1	(54)	0.007	1	U	K.HLVDEPQNLIKQNCDQFEK.L
5780	786.0457	2355.1153	2354.1325	0.9828	1	(64)	0.00058	1	U	K.HLVDEPQNLIKQNCDQFEK.L
5781	589.7880	2355.1229	2354.1325	0.9904	1	(58)	0.0024	1	U	K.HLVDEPQNLIKQNCDQFEK.L
5783	786.0486	2355.1240	2354.1325	0.9915	1	(61)	0.0012	1	U	K.HLVDEPQNLIKQNCDQFEK.L
5784	786.0488	2355.1246	2354.1325	0.9921	1	(63)	0.00081	1	U	K.HLVDEPQNLIKQNCDQFEK.L
5785	1178.5696	2355.1246	2354.1325	0.9922	1	(75)	5.5e-05	1	U	K.HLVDEPQNLIKQNCDQFEK.L
5786	589.7885	2355.1249	2354.1325	0.9924	1	(52)	0.01	1	U	K.HLVDEPQNLIKQNCDQFEK.L
5787	589.7889	2355.1265	2354.1325	0.9940	1	(52)	0.009	1	U	K.HLVDEPQNLIKQNCDQFEK.L
5789	1178.5719	2355.1292	2354.1325	0.9968	1	79	1.8e-05	1	U	K.HLVDEPQNLIKQNCDQFEK.L
5899	618.5505	2470.1729	2470.1839	-0.0110	1	48	0.022	1	U	R.RPCFSALTPDETYVPKAFDEK.L
5904	824.7322	2471.1748	2470.1839	0.9909	1	(46)	0.037	1	U	R.RPCFSALTPDETYVPKAFDEK.L
5906	824.7336	2471.1790	2470.1839	0.9951	1	(46)	0.033	1	U	R.RPCFSALTPDETYVPKAFDEK.L
5907	1236.6000	2471.1854	2470.1839	1.0016	1	(45)	0.048	1	U	R.RPCFSALTPDETYVPKAFDEK.L
5932	1244.0533	2486.0920	2486.1028	-0.0108	1	(50)	0.014	1		K.YNGVFQECCQAEDKGACLLPK.I
5933	1244.0533	2486.0920	2486.1028	-0.0108	1	(56)	0.0036	1		K.YNGVFQECCQAEDKGACLLPK.I
5934	1244.5452	2487.0758	2486.1028	0.9730	1	(48)	0.022	1		K.YNGVFQECCQAEDKGACLLPK.I
5935	1244.5475	2487.0804	2486.1028	0.9776	1	71	9.4e-05	1		K.YNGVFQECCQAEDKGACLLPK.I
5936	830.0377	2487.0913	2486.1028	0.9884	1	(45)	0.041	1		K.YNGVFQECCQAEDKGACLLPK.I
5938	830.0383	2487.0931	2486.1028	0.9902	1	(44)	0.047	1		K.YNGVFQECCQAEDKGACLLPK.I
5944	623.8186	2491.2453	2491.2570	-0.0117	0	(56)	0.0035	1	U	K.GLVLIAFSQYLQQCPFDEHVK.L
5946	624.0688	2492.2461	2491.2570	0.9891	0	(56)	0.0036	1	U	K.GLVLIAFSQYLQQCPFDEHVK.L
5947	1247.1307	2492.2468	2491.2570	0.9899	0	89	2e-06	1	U	K.GLVLIAFSQYLQQCPFDEHVK.L
5948	831.7567	2492.2483	2491.2570	0.9913	0	(65)	0.00043	1	U	K.GLVLIAFSQYLQQCPFDEHVK.L
5949	624.0695	2492.2489	2491.2570	0.9919	0	(57)	0.0027	1	U	K.GLVLIAFSQYLQQCPFDEHVK.L
5950	624.0696	2492.2493	2491.2570	0.9923	0	(51)	0.012	1	U	K.GLVLIAFSQYLQQCPFDEHVK.L
5951	831.7573	2492.2501	2491.2570	0.9931	0	(61)	0.0012	1	U	K.GLVLIAFSQYLQQCPFDEHVK.L
5952	831.7574	2492.2504	2491.2570	0.9934	0	(73)	7.2e-05	1	U	K.GLVLIAFSQYLQQCPFDEHVK.L
5953	624.0699	2492.2505	2491.2570	0.9935	0	(51)	0.012	1	U	K.GLVLIAFSQYLQQCPFDEHVK.L
5954	831.7579	2492.2519	2491.2570	0.9949	0	(46)	0.036	1	U	K.GLVLIAFSQYLQQCPFDEHVK.L
5955	831.7580	2492.2522	2491.2570	0.9952	0	(48)	0.021	1	U	K.GLVLIAFSQYLQQCPFDEHVK.L
5957	831.7582	2492.2528	2491.2570	0.9958	0	(56)	0.0034	1	U	K.GLVLIAFSQYLQQCPFDEHVK.L
5958	831.7584	2492.2534	2491.2570	0.9964	0	(62)	0.00097	1	U	K.GLVLIAFSQYLQQCPFDEHVK.L
5959	831.7586	2492.2540	2491.2570	0.9970	0	(51)	0.01	1	U	K.GLVLIAFSQYLQQCPFDEHVK.L
5960	831.7586	2492.2540	2491.2570	0.9970	0	(53)	0.0069	1	U	K.GLVLIAFSQYLQQCPFDEHVK.L
5961	1247.1344	2492.2542	2491.2570	0.9973	0	(63)	0.00069	1	U	K.GLVLIAFSQYLQQCPFDEHVK.L
5977	833.3958	2497.1656	2497.1835	-0.0180	1	(74)	5.1e-05	1	U	K.AFDEKLFTFHADICTLPDTEK.Q
5978	1249.5914	2497.1682	2497.1835	-0.0153	1	(76)	3.9e-05	1	U	K.AFDEKLFTFHADICTLPDTEK.Q
5981	833.3978	2497.1716	2497.1835	-0.0120	1	(46)	0.032	1	U	K.AFDEKLFTFHADICTLPDTEK.Q
5982	833.3986	2497.1740	2497.1835	-0.0096	1	(50)	0.013	1	U	K.AFDEKLFTFHADICTLPDTEK.Q
5983	833.3994	2497.1764	2497.1835	-0.0072	1	(76)	3.5e-05	1	U	K.AFDEKLFTFHADICTLPDTEK.Q
5984	833.3999	2497.1779	2497.1835	-0.0057	1	79	1.7e-05	1	U	K.AFDEKLFTFHADICTLPDTEK.Q
5986	833.4026	2497.1860	2497.1835	0.0024	1	(52)	0.008	1	U	K.AFDEKLFTFHADICTLPDTEK.Q
5999	625.5507	2498.1737	2497.1835	0.9902	1	(48)	0.02	1	U	K.AFDEKLFTFHADICTLPDTEK.Q
6000	833.7321	2498.1745	2497.1835	0.9909	1	(68)	0.00023	1	U	K.AFDEKLFTFHADICTLPDTEK.Q
6002	1250.0953	2498.1760	2497.1835	0.9925	1	(77)	3.1e-05	1	U	K.AFDEKLFTFHADICTLPDTEK.Q
6003	625.5513	2498.1761	2497.1835	0.9926	1	(47)	0.029	1	U	K.AFDEKLFTFHADICTLPDTEK.Q
6004	625.5514	2498.1765	2497.1835	0.9930	1	(45)	0.044	1	U	K.AFDEKLFTFHADICTLPDTEK.Q
6005	833.7330	2498.1772	2497.1835	0.9936	1	(76)	3.4e-05	1	U	K.AFDEKLFTFHADICTLPDTEK.Q
6011	625.5526	2498.1813	2497.1835	0.9978	1	(51)	0.011	1	U	K.AFDEKLFTFHADICTLPDTEK.Q
6061	843.7262	2528.1568	2528.2118	-0.0550	1	(49)	0.016	2	U	K.QNCDQFEKLGEYGFQNALIVR.Y
6068	844.0744	2529.2014	2528.2118	0.9896	1	(128)	2e-10	1	U	K.QNCDQFEKLGEYGFQNALIVR.Y
6069	844.0745	2529.2017	2528.2118	0.9899	1	(106)	3.3e-08	1	U	K.QNCDQFEKLGEYGFQNALIVR.Y
6070	844.0745	2529.2017	2528.2118	0.9899	1	(103)	7.5e-08	1	U	K.QNCDQFEKLGEYGFQNALIVR.Y

☒	6071	844.0750	2529.2032	2528.2118	0.9914	1	(107)	2.8e-08	1	U	K.QNCDQFEKLGEYGFQNALIVR.Y
☒	6072	1265.6089	2529.2032	2528.2118	0.9915	1	143	6.6e-12	1	U	K.QNCDQFEKLGEYGFQNALIVR.Y
☒	6073	1265.6090	2529.2034	2528.2118	0.9917	1	(127)	2.8e-10	1	U	K.QNCDQFEKLGEYGFQNALIVR.Y
☒	6074	633.3082	2529.2037	2528.2118	0.9919	1	(50)	0.014	1	U	K.QNCDQFEKLGEYGFQNALIVR.Y
☒	6075	844.0753	2529.2041	2528.2118	0.9923	1	(110)	1.4e-08	1	U	K.QNCDQFEKLGEYGFQNALIVR.Y
☒	6076	844.0754	2529.2044	2528.2118	0.9926	1	(106)	3.3e-08	1	U	K.QNCDQFEKLGEYGFQNALIVR.Y
☒	6077	844.0756	2529.2050	2528.2118	0.9932	1	(102)	8.5e-08	1	U	K.QNCDQFEKLGEYGFQNALIVR.Y
☒	6078	1265.6100	2529.2054	2528.2118	0.9937	1	(122)	8.5e-10	1	U	K.QNCDQFEKLGEYGFQNALIVR.Y
☒	6079	844.0760	2529.2062	2528.2118	0.9944	1	(123)	6.5e-10	1	U	K.QNCDQFEKLGEYGFQNALIVR.Y
☒	6080	844.0760	2529.2062	2528.2118	0.9944	1	(128)	2.1e-10	1	U	K.QNCDQFEKLGEYGFQNALIVR.Y
☒	6081	844.0760	2529.2062	2528.2118	0.9944	1	(103)	7.5e-08	1	U	K.QNCDQFEKLGEYGFQNALIVR.Y
☒	6224	870.4022	2608.1848	2607.1945	0.9903	1	96	2.9e-07	1	U	K.LVNELTEFAKTCVADESHAGCEK.S
☒	6225	653.0535	2608.1849	2607.1945	0.9904	1	(95)	3.8e-07	1	U	K.LVNELTEFAKTCVADESHAGCEK.S
☒	6690	955.7581	2864.2525	2863.2575	0.9949	1	76	2.7e-05	1	U	K.TCVADESHAGCEKSLHTLFGDELCK.V
☒	6741	958.4371	2872.2895	2871.3023	0.9871	1	(50)	0.0097	1	U	R.CCTKPESERMPCTEDYLSLILNR.L
☒	6743	719.0801	2872.2913	2871.3023	0.9890	1	(49)	0.013	1	U	R.CCTKPESERMPCTEDYLSLILNR.L
☒	6744	1437.1538	2872.2930	2871.3023	0.9907	1	73	5.4e-05	1	U	R.CCTKPESERMPCTEDYLSLILNR.L
☒	6745	958.4383	2872.2931	2871.3023	0.9908	1	(53)	0.0052	1	U	R.CCTKPESERMPCTEDYLSLILNR.L
☒	6746	719.0806	2872.2933	2871.3023	0.9910	1	(44)	0.043	1	U	R.CCTKPESERMPCTEDYLSLILNR.L
☒	6748	719.0808	2872.2941	2871.3023	0.9918	1	(49)	0.013	1	U	R.CCTKPESERMPCTEDYLSLILNR.L
☒	6749	958.4387	2872.2943	2871.3023	0.9919	1	(56)	0.0026	1	U	R.CCTKPESERMPCTEDYLSLILNR.L
☒	6751	958.4413	2872.3021	2871.3023	0.9998	1	(58)	0.0016	1	U	R.CCTKPESERMPCTEDYLSLILNR.L
☒	6777	963.7704	2888.2894	2887.2972	0.9921	1	(50)	0.012	1	U	R.CCTKPESERMPCTEDYLSLILNR.L + Oxidation (M)
☒	6780	963.7715	2888.2927	2887.2972	0.9954	1	(54)	0.0043	1	U	R.CCTKPESERMPCTEDYLSLILNR.L + Oxidation (M)
☒	7305	862.9253	3447.6721	3446.6969	0.9752	1	47	0.017	1	U	K.DLGEEHFKGLVLIAFSQYLQQCPFDEHVK.L
☒	7496	910.2211	3636.8553	3635.8698	0.9855	1	(58)	0.00099	1	U	K.GLVLIAFSQYLQQCPFDEHVKLVNELTEFAK.T
☒	7497	910.2212	3636.8557	3635.8698	0.9859	1	79	9.1e-06	1	U	K.GLVLIAFSQYLQQCPFDEHVKLVNELTEFAK.T
☒	7498	910.2215	3636.8569	3635.8698	0.9871	1	(68)	0.0001	1	U	K.GLVLIAFSQYLQQCPFDEHVKLVNELTEFAK.T
☒	7499	910.2228	3636.8621	3635.8698	0.9923	1	(64)	0.00027	1	U	K.GLVLIAFSQYLQQCPFDEHVKLVNELTEFAK.T
☒	7500	910.2228	3636.8621	3635.8698	0.9923	1	(73)	3.6e-05	1	U	K.GLVLIAFSQYLQQCPFDEHVKLVNELTEFAK.T
☒	7501	1213.2960	3636.8662	3635.8698	0.9964	1	(67)	0.00013	1	U	K.GLVLIAFSQYLQQCPFDEHVKLVNELTEFAK.T

---

2. ALBU_SHEEP **Mass:** 71139 **Score:** 5838 **Matches:** 222(222) **Sequences:** 17(17) **emPAI:** 1.15

Albumin OS=Ovis aries OX=9940 GN=ALB PE=1 SV=1

☐ Check to include this hit in error tolerant search

Query	Observed	Mr(expt)	Mr(calc)	Delta	Miss	Score	Expect	Rank	Unique	Peptide
2802	501.2960	1000.5774	1000.5818	-0.0043	1	(49)	0.04	2		R.ALKAWSVAR.L
2803	501.2961	1000.5776	1000.5818	-0.0041	1	53	0.017	1		R.ALKAWSVAR.L
2923	1014.6146	1013.6073	1013.6121	-0.0048	0	(55)	0.011	1		K.QTALVELLK.H
2938	1014.6152	1013.6079	1013.6121	-0.0042	0	60	0.0029	1		K.QTALVELLK.H
2949	1014.6168	1013.6095	1013.6121	-0.0026	0	(57)	0.0063	1		K.QTALVELLK.H
2950	1014.6179	1013.6106	1013.6121	-0.0015	0	(51)	0.027	1		K.QTALVELLK.H
3106	571.8574	1141.7002	1141.7070	-0.0068	1	(62)	0.0018	1		K.KQTALVELLK.H
3108	571.8578	1141.7010	1141.7070	-0.0060	1	(66)	0.00066	1		K.KQTALVELLK.H
3109	571.8579	1141.7012	1141.7070	-0.0058	1	(70)	0.00028	1		K.KQTALVELLK.H
3110	571.8579	1141.7012	1141.7070	-0.0058	1	(70)	0.00027	1		K.KQTALVELLK.H
3113	571.8580	1141.7014	1141.7070	-0.0056	1	(70)	0.00028	1		K.KQTALVELLK.H
3114	571.8582	1141.7018	1141.7070	-0.0052	1	(70)	0.00028	1		K.KQTALVELLK.H
3115	571.8582	1141.7018	1141.7070	-0.0052	1	(67)	0.00055	1		K.KQTALVELLK.H
3116	571.8582	1141.7018	1141.7070	-0.0052	1	(64)	0.0011	1		K.KQTALVELLK.H
3117	571.8582	1141.7018	1141.7070	-0.0052	1	(50)	0.03	1		K.KQTALVELLK.H
3121	571.8583	1141.7020	1141.7070	-0.0050	1	(70)	0.00027	1		K.KQTALVELLK.H
3122	571.8583	1141.7020	1141.7070	-0.0050	1	(67)	0.00054	1		K.KQTALVELLK.H
3123	571.8584	1141.7022	1141.7070	-0.0048	1	(67)	0.00052	1		K.KQTALVELLK.H
3127	571.8585	1141.7024	1141.7070	-0.0046	1	(70)	0.00027	1		K.KQTALVELLK.H
3128	571.8585	1141.7024	1141.7070	-0.0046	1	(66)	0.00066	1		K.KQTALVELLK.H
3129	571.8585	1141.7024	1141.7070	-0.0046	1	(67)	0.00062	1		K.KQTALVELLK.H
3133	571.8588	1141.7030	1141.7070	-0.0040	1	(67)	0.00063	1		K.KQTALVELLK.H
3135	571.8589	1141.7032	1141.7070	-0.0038	1	(51)	0.022	1		K.KQTALVELLK.H
3139	571.8591	1141.7036	1141.7070	-0.0034	1	(65)	0.00093	1		K.KQTALVELLK.H
3140	571.8591	1141.7036	1141.7070	-0.0034	1	(56)	0.0066	1		K.KQTALVELLK.H
3141	571.8593	1141.7040	1141.7070	-0.0030	1	70	0.00027	1		K.KQTALVELLK.H
3156	577.3480	1152.6814	1152.6867	-0.0052	1	(56)	0.0061	1	U	K.IVTDLTKVHK.E
3160	385.5687	1153.6843	1152.6867	0.9976	1	65	0.00087	1	U	K.IVTDLTKVHK.E
3385	642.3547	1282.6948	1282.7034	-0.0085	0	(53)	0.013	1		R.HPEYAVSVLLR.L
3387	642.3557	1282.6968	1282.7034	-0.0065	0	(56)	0.0063	1		R.HPEYAVSVLLR.L
3388	642.3561	1282.6976	1282.7034	-0.0057	0	62	0.0015	1		R.HPEYAVSVLLR.L
3389	428.5732	1282.6978	1282.7034	-0.0056	0	(49)	0.031	1		R.HPEYAVSVLLR.L
3390	428.5732	1282.6978	1282.7034	-0.0056	0	(58)	0.0046	1		R.HPEYAVSVLLR.L
3391	642.3568	1282.6990	1282.7034	-0.0043	0	(61)	0.0023	1		R.HPEYAVSVLLR.L
3392	642.3569	1282.6992	1282.7034	-0.0041	0	(57)	0.0048	1		R.HPEYAVSVLLR.L
3393	642.3570	1282.6994	1282.7034	-0.0039	0	(57)	0.0053	1		R.HPEYAVSVLLR.L
3394	428.5739	1282.6999	1282.7034	-0.0035	0	(49)	0.033	1		R.HPEYAVSVLLR.L
3397	642.8580	1283.7014	1282.7034	0.9981	0	(50)	0.027	1		R.HPEYAVSVLLR.L
3463	1305.7092	1304.7019	1304.7088	-0.0069	0	(48)	0.04	1		K.HLVDEPQNLIK.K
3468	653.3586	1304.7026	1304.7088	-0.0062	0	(48)	0.039	1		K.HLVDEPQNLIK.K
3471	653.3588	1304.7030	1304.7088	-0.0058	0	(51)	0.022	1		K.HLVDEPQNLIK.K
3473	653.3589	1304.7032	1304.7088	-0.0056	0	(48)	0.041	1		K.HLVDEPQNLIK.K
3477	1305.7109	1304.7036	1304.7088	-0.0052	0	(48)	0.042	1		K.HLVDEPQNLIK.K
3482	653.8584	1305.7022	1304.7088	0.9934	0	55	0.0077	1		K.HLVDEPQNLIK.K
3694	700.3462	1398.6778	1398.6853	-0.0075	0	(74)	0.00011	1		K.TVMENFVAFVDK.C
3695	700.3462	1398.6778	1398.6853	-0.0075	0	(79)	3.7e-05	1		K.TVMENFVAFVDK.C
3696	700.3464	1398.6782	1398.6853	-0.0071	0	93	1.5e-06	1		K.TVMENFVAFVDK.C
3698	700.3479	1398.6812	1398.6853	-0.0041	0	(79)	3.7e-05	1		K.TVMENFVAFVDK.C
3699	700.3506	1398.6866	1398.6853	0.0013	0	(69)	0.00032	1		K.TVMENFVAFVDK.C
3702	700.8475	1399.6804	1398.6853	0.9951	0	(61)	0.0023	1		K.TVMENFVAFVDK.C
3703	700.8491	1399.6836	1398.6853	0.9983	0	(62)	0.0018	1		K.TVMENFVAFVDK.C
3704	700.8500	1399.6854	1398.6853	1.0001	0	(60)	0.0025	1		K.TVMENFVAFVDK.C
3705	700.8506	1399.6866	1398.6853	1.0013	0	(61)	0.002	1		K.TVMENFVAFVDK.C
3759	710.0826	1418.1506	1418.6864	-0.5358	0	(49)	0.021	1		K.SLHTLFGDELCK.V
3769	710.3472	1418.6798	1418.6864	-0.0066	0	(54)	0.011	1		K.SLHTLFGDELCK.V
3773	710.3474	1418.6802	1418.6864	-0.0062	0	(52)	0.017	1		K.SLHTLFGDELCK.V
3774	710.3474	1418.6802	1418.6864	-0.0062	0	(59)	0.0032	1		K.SLHTLFGDELCK.V
3775	710.3474	1418.6802	1418.6864	-0.0062	0	(55)	0.0077	1		K.SLHTLFGDELCK.V
3780	710.3475	1418.6804	1418.6864	-0.0060	0	(59)	0.0035	1		K.SLHTLFGDELCK.V
3781	710.3475	1418.6804	1418.6864	-0.0060	0	(57)	0.0052	1		K.SLHTLFGDELCK.V
3785	710.3478	1418.6810	1418.6864	-0.0054	0	(56)	0.0064	1		K.SLHTLFGDELCK.V
3789	1419.6888	1418.6815	1418.6864	-0.0049	0	66	0.00072	1		K.SLHTLFGDELCK.V
3790	1419.6888	1418.6815	1418.6864	-0.0049	0	(64)	0.00096	1		K.SLHTLFGDELCK.V
3791	710.3481	1418.6816	1418.6864	-0.0048	0	(58)	0.0046	1		K.SLHTLFGDELCK.V
3796	710.3484	1418.6822	1418.6864	-0.0042	0	(54)	0.011	1		K.SLHTLFGDELCK.V
3798	710.3485	1418.6824	1418.6864	-0.0040	0	(57)	0.0051	1		K.SLHTLFGDELCK.V
3799	710.3489	1418.6832	1418.6864	-0.0032	0	(64)	0.00094	1		K.SLHTLFGDELCK.V

3806	710.8486	1419.6826	1418.6864	0.9962	0	(64)	0.00096	1	K.SLHTLFGDELCK.V
3808	710.8488	1419.6830	1418.6864	0.9966	0	(50)	0.028	1	K.SLHTLFGDELCK.V
3809	710.8488	1419.6830	1418.6864	0.9966	0	(52)	0.016	1	K.SLHTLFGDELCK.V
3813	710.8495	1419.6844	1418.6864	0.9980	0	(50)	0.025	1	K.SLHTLFGDELCK.V
3848	720.1332	1438.2518	1438.8045	-0.5526	1	(50)	0.015	1	R.RHPEYAVSVLLR.L
3849	720.3298	1438.6450	1438.8045	-0.1594	1	(56)	0.0067	1	R.RHPEYAVSVLLR.L
3850	720.4041	1438.7936	1438.8045	-0.0108	1	(58)	0.004	1	R.RHPEYAVSVLLR.L
3851	720.4051	1438.7956	1438.8045	-0.0088	1	(70)	0.00027	1	R.RHPEYAVSVLLR.L
3852	480.6059	1438.7959	1438.8045	-0.0086	1	(50)	0.028	1	R.RHPEYAVSVLLR.L
3853	480.6061	1438.7965	1438.8045	-0.0080	1	(62)	0.0016	1	R.RHPEYAVSVLLR.L
3854	480.6061	1438.7965	1438.8045	-0.0080	1	(61)	0.0019	1	R.RHPEYAVSVLLR.L
3857	480.6063	1438.7971	1438.8045	-0.0074	1	78	4.3e-05	1	R.RHPEYAVSVLLR.L
3858	720.4059	1438.7972	1438.8045	-0.0072	1	(55)	0.0072	1	R.RHPEYAVSVLLR.L
3859	480.6064	1438.7974	1438.8045	-0.0071	1	(50)	0.023	1	R.RHPEYAVSVLLR.L
3861	480.6064	1438.7974	1438.8045	-0.0071	1	(62)	0.0016	1	R.RHPEYAVSVLLR.L
3862	720.4061	1438.7976	1438.8045	-0.0068	1	(68)	0.00041	1	R.RHPEYAVSVLLR.L
3863	480.6065	1438.7977	1438.8045	-0.0068	1	(70)	0.00028	1	R.RHPEYAVSVLLR.L
3864	480.6065	1438.7977	1438.8045	-0.0068	1	(55)	0.0085	1	R.RHPEYAVSVLLR.L
3865	480.6065	1438.7977	1438.8045	-0.0068	1	(62)	0.0016	1	R.RHPEYAVSVLLR.L
3866	480.6065	1438.7977	1438.8045	-0.0068	1	(75)	8.2e-05	1	R.RHPEYAVSVLLR.L
3868	720.4062	1438.7978	1438.8045	-0.0066	1	(66)	0.00064	1	R.RHPEYAVSVLLR.L
3869	720.4062	1438.7978	1438.8045	-0.0066	1	(74)	8.9e-05	1	R.RHPEYAVSVLLR.L
3871	480.6066	1438.7980	1438.8045	-0.0065	1	(62)	0.0016	1	R.RHPEYAVSVLLR.L
3872	480.6066	1438.7980	1438.8045	-0.0065	1	(59)	0.0032	1	R.RHPEYAVSVLLR.L
3873	480.6066	1438.7980	1438.8045	-0.0065	1	(62)	0.0016	1	R.RHPEYAVSVLLR.L
3874	480.6066	1438.7980	1438.8045	-0.0065	1	(62)	0.0017	1	R.RHPEYAVSVLLR.L
3875	720.4063	1438.7980	1438.8045	-0.0064	1	(54)	0.0098	1	R.RHPEYAVSVLLR.L
3876	720.4063	1438.7980	1438.8045	-0.0064	1	(70)	0.00023	1	R.RHPEYAVSVLLR.L
3879	480.6067	1438.7983	1438.8045	-0.0062	1	(57)	0.0055	1	R.RHPEYAVSVLLR.L
3880	480.6067	1438.7983	1438.8045	-0.0062	1	(61)	0.0019	1	R.RHPEYAVSVLLR.L
3881	480.6067	1438.7983	1438.8045	-0.0062	1	(65)	0.00087	1	R.RHPEYAVSVLLR.L
3882	480.6067	1438.7983	1438.8045	-0.0062	1	(65)	0.00082	1	R.RHPEYAVSVLLR.L
3883	480.6067	1438.7983	1438.8045	-0.0062	1	(71)	0.00019	1	R.RHPEYAVSVLLR.L
3884	480.6067	1438.7983	1438.8045	-0.0062	1	(65)	0.00089	1	R.RHPEYAVSVLLR.L
3885	480.6067	1438.7983	1438.8045	-0.0062	1	(54)	0.01	1	R.RHPEYAVSVLLR.L
3886	480.6067	1438.7983	1438.8045	-0.0062	1	(70)	0.00024	1	R.RHPEYAVSVLLR.L
3887	480.6067	1438.7983	1438.8045	-0.0062	1	(59)	0.0033	1	R.RHPEYAVSVLLR.L
3888	480.6067	1438.7983	1438.8045	-0.0062	1	(65)	0.00075	1	R.RHPEYAVSVLLR.L
3890	480.6068	1438.7986	1438.8045	-0.0059	1	(56)	0.0068	1	R.RHPEYAVSVLLR.L
3891	480.6068	1438.7986	1438.8045	-0.0059	1	(62)	0.0016	1	R.RHPEYAVSVLLR.L
3892	480.6068	1438.7986	1438.8045	-0.0059	1	(53)	0.014	1	R.RHPEYAVSVLLR.L
3893	480.6068	1438.7986	1438.8045	-0.0059	1	(71)	0.0002	1	R.RHPEYAVSVLLR.L
3894	480.6068	1438.7986	1438.8045	-0.0059	1	(61)	0.0019	1	R.RHPEYAVSVLLR.L
3895	480.6068	1438.7986	1438.8045	-0.0059	1	(62)	0.0016	1	R.RHPEYAVSVLLR.L
3896	480.6068	1438.7986	1438.8045	-0.0059	1	(54)	0.011	1	R.RHPEYAVSVLLR.L
3897	720.4066	1438.7986	1438.8045	-0.0058	1	(68)	0.0004	1	R.RHPEYAVSVLLR.L
3898	720.4067	1438.7988	1438.8045	-0.0056	1	(72)	0.00017	1	R.RHPEYAVSVLLR.L
3899	720.4067	1438.7988	1438.8045	-0.0056	1	(65)	0.00088	1	R.RHPEYAVSVLLR.L
3900	480.6069	1438.7989	1438.8045	-0.0056	1	(65)	0.00088	1	R.RHPEYAVSVLLR.L
3901	480.6069	1438.7989	1438.8045	-0.0056	1	(57)	0.0055	1	R.RHPEYAVSVLLR.L
3902	480.6069	1438.7989	1438.8045	-0.0056	1	(70)	0.00023	1	R.RHPEYAVSVLLR.L
3903	480.6069	1438.7989	1438.8045	-0.0056	1	(74)	8.9e-05	1	R.RHPEYAVSVLLR.L
3904	480.6069	1438.7989	1438.8045	-0.0056	1	(62)	0.0016	1	R.RHPEYAVSVLLR.L
3907	480.6070	1438.7992	1438.8045	-0.0053	1	(68)	0.00038	1	R.RHPEYAVSVLLR.L
3908	480.6070	1438.7992	1438.8045	-0.0053	1	(59)	0.0032	1	R.RHPEYAVSVLLR.L
3909	480.6070	1438.7992	1438.8045	-0.0053	1	(62)	0.0016	1	R.RHPEYAVSVLLR.L
3910	480.6070	1438.7992	1438.8045	-0.0053	1	(61)	0.0019	1	R.RHPEYAVSVLLR.L
3911	480.6070	1438.7992	1438.8045	-0.0053	1	(62)	0.0015	1	R.RHPEYAVSVLLR.L
3912	480.6070	1438.7992	1438.8045	-0.0053	1	(62)	0.0016	1	R.RHPEYAVSVLLR.L
3913	480.6070	1438.7992	1438.8045	-0.0053	1	(62)	0.0016	1	R.RHPEYAVSVLLR.L
3914	480.6070	1438.7992	1438.8045	-0.0053	1	(54)	0.0094	1	R.RHPEYAVSVLLR.L
3915	720.4069	1438.7992	1438.8045	-0.0052	1	(72)	0.00018	1	R.RHPEYAVSVLLR.L
3916	720.4069	1438.7992	1438.8045	-0.0052	1	(60)	0.0023	1	R.RHPEYAVSVLLR.L
3917	720.4069	1438.7992	1438.8045	-0.0052	1	(69)	0.00032	1	R.RHPEYAVSVLLR.L
3918	720.4070	1438.7994	1438.8045	-0.0050	1	(66)	0.0006	1	R.RHPEYAVSVLLR.L
3919	480.6071	1438.7995	1438.8045	-0.0050	1	(62)	0.0016	1	R.RHPEYAVSVLLR.L
3920	480.6071	1438.7995	1438.8045	-0.0050	1	(52)	0.016	1	R.RHPEYAVSVLLR.L
3921	480.6071	1438.7995	1438.8045	-0.0050	1	(65)	0.00085	1	R.RHPEYAVSVLLR.L
3922	480.6071	1438.7995	1438.8045	-0.0050	1	(62)	0.0016	1	R.RHPEYAVSVLLR.L
3923	480.6071	1438.7995	1438.8045	-0.0050	1	(61)	0.0018	1	R.RHPEYAVSVLLR.L
3924	480.6071	1438.7995	1438.8045	-0.0050	1	(62)	0.0016	1	R.RHPEYAVSVLLR.L
3925	480.6071	1438.7995	1438.8045	-0.0050	1	(57)	0.0054	1	R.RHPEYAVSVLLR.L
3926	480.6071	1438.7995	1438.8045	-0.0050	1	(59)	0.0034	1	R.RHPEYAVSVLLR.L
3927	480.6071	1438.7995	1438.8045	-0.0050	1	(65)	0.00087	1	R.RHPEYAVSVLLR.L
3928	480.6071	1438.7995	1438.8045	-0.0050	1	(53)	0.013	1	R.RHPEYAVSVLLR.L
3929	480.6071	1438.7995	1438.8045	-0.0050	1	(60)	0.0028	1	R.RHPEYAVSVLLR.L
3930	480.6071	1438.7995	1438.8045	-0.0050	1	(60)	0.0027	1	R.RHPEYAVSVLLR.L
3931	480.6071	1438.7995	1438.8045	-0.0050	1	(60)	0.0025	1	R.RHPEYAVSVLLR.L
3932	720.4071	1438.7996	1438.8045	-0.0048	1	(75)	7.4e-05	1	R.RHPEYAVSVLLR.L
3934	480.6072	1438.7998	1438.8045	-0.0047	1	(61)	0.0019	1	R.RHPEYAVSVLLR.L
3935	480.6072	1438.7998	1438.8045	-0.0047	1	(60)	0.0024	1	R.RHPEYAVSVLLR.L
3936	480.6072	1438.7998	1438.8045	-0.0047	1	(57)	0.0046	1	R.RHPEYAVSVLLR.L
3937	480.6072	1438.7998	1438.8045	-0.0047	1	(62)	0.0015	1	R.RHPEYAVSVLLR.L
3938	480.6072	1438.7998	1438.8045	-0.0047	1	(60)	0.0022	1	R.RHPEYAVSVLLR.L
3939	480.6072	1438.7998	1438.8045	-0.0047	1	(65)	0.00088	1	R.RHPEYAVSVLLR.L
3940	720.4073	1438.8000	1438.8045	-0.0044	1	(66)	0.0006	1	R.RHPEYAVSVLLR.L
3941	720.4073	1438.8000	1438.8045	-0.0044	1	(56)	0.006	1	R.RHPEYAVSVLLR.L
3942	480.6073	1438.8001	1438.8045	-0.0044	1	(57)	0.0046	1	R.RHPEYAVSVLLR.L
3943	480.6074	1438.8004	1438.8045	-0.0041	1	(65)	0.00087	1	R.RHPEYAVSVLLR.L
3944	480.6074	1438.8004	1438.8045	-0.0041	1	(54)	0.0094	1	R.RHPEYAVSVLLR.L
3946	480.6075	1438.8007	1438.8045	-0.0038	1	(62)	0.0015	1	R.RHPEYAVSVLLR.L
3947	480.6076	1438.8010	1438.8045	-0.0035	1	(72)	0.00016	1	R.RHPEYAVSVLLR.L
3948	720.4092	1438.8038	1438.8045	-0.0006	1	(63)	0.0013	1	R.RHPEYAVSVLLR.L
3949	720.4095	1438.8044	1438.8045	-0.0000	1	(64)	0.0011	1	R.RHPEYAVSVLLR.L
3950	720.9019	1439.7892	1438.8045	0.9848	1	(63)	0.0014	1	R.RHPEYAVSVLLR.L
3951	720.9060	1439.7974	1438.8045	0.9930	1	(65)	0.00072	1	R.RHPEYAVSVLLR.L
3952	720.9064	1439.7982	1438.8045	0.9938	1	(62)	0.0015	1	R.RHPEYAVSVLLR.L
3953	720.9064	1439.7982	1438.8045	0.9938	1	(59)	0.0031	1	R.RHPEYAVSVLLR.L
3954	480.9405	1439.7997	1438.8045	0.9952	1	(63)	0.0014	1	R.RHPEYAVSVLLR.L
3957	480.9406	1439.8000	1438.8045	0.9955	1	(61)	0.002	1	R.RHPEYAVSVLLR.L
3958	720.9075	1439.8004	1438.8045	0.9960	1	(62)	0.0016	1	R.RHPEYAVSVLLR.L
3959	720.9077	1439.8008	1438.8045	0.9964	1	(60)	0.0026	1	R.RHPEYAVSVLLR.L
3960	720.9077	1439.8008	1438.8045	0.9964	1	(66)	0.0007	1	R.RHPEYAVSVLLR.L
3962	720.9078	1439.8010	1438.8045	0.9966	1	(65)	0.00079	1	R.RHPEYAVSVLLR.L
3963	720.9078	1439.8010	1438.8045	0.9966	1	(63)	0.0014	1	R.RHPEYAVSVLLR.L
3964	720.9080	1439.8014	1438.8045	0.9970	1	(68)	0.00043	1	R.RHPEYAVSVLLR.L

	3965	720.9081	1439.8016	1438.8045	0.9972	1	(63)	0.0013	1		R.RHPEYAVSVLLR.L
	3967	720.9086	1439.8026	1438.8045	0.9982	1	(63)	0.0014	1		R.RHPEYAVSVLLR.L
	3968	720.9088	1439.8030	1438.8045	0.9986	1	(63)	0.0012	1		R.RHPEYAVSVLLR.L
	3970	720.9109	1439.8072	1438.8045	1.0028	1	(63)	0.0012	1		R.RHPEYAVSVLLR.L
	4291	770.4079	1538.8012	1538.8127	-0.0114	1	(66)	0.00065	1		R.LCVLHEKTPVSEK.V
	4292	513.9421	1538.8045	1538.8127	-0.0082	1	(63)	0.0012	1		R.LCVLHEKTPVSEK.V
	4295	513.9423	1538.8051	1538.8127	-0.0076	1	(63)	0.0012	1		R.LCVLHEKTPVSEK.V
	4296	513.9423	1538.8051	1538.8127	-0.0076	1	(58)	0.0034	1		R.LCVLHEKTPVSEK.V
	4297	770.4099	1538.8052	1538.8127	-0.0074	1	(71)	0.00019	1		R.LCVLHEKTPVSEK.V
	4299	513.9424	1538.8054	1538.8127	-0.0073	1	(63)	0.0013	1		R.LCVLHEKTPVSEK.V
	4300	770.4100	1538.8054	1538.8127	-0.0072	1	(51)	0.021	1		R.LCVLHEKTPVSEK.V
	4301	770.4100	1538.8054	1538.8127	-0.0072	1	71	0.00017	1		R.LCVLHEKTPVSEK.V
	4304	385.7087	1538.8057	1538.8127	-0.0070	1	(50)	0.022	1		R.LCVLHEKTPVSEK.V
	4305	513.9426	1538.8060	1538.8127	-0.0067	1	(62)	0.0014	1		R.LCVLHEKTPVSEK.V
	4306	770.4106	1538.8066	1538.8127	-0.0060	1	(71)	0.0002	1		R.LCVLHEKTPVSEK.V
	4307	770.4116	1538.8086	1538.8127	-0.0040	1	(59)	0.003	1		R.LCVLHEKTPVSEK.V
	4309	770.9101	1539.8056	1538.8127	0.9930	1	(54)	0.0088	1		R.LCVLHEKTPVSEK.V
	4311	514.2764	1539.8074	1538.8127	0.9947	1	(62)	0.0015	1		R.LCVLHEKTPVSEK.V
☒	4499	**797.8824**	**1593.7502**	**1594.7668**	**-1.0165**	**0**	**60**	**0.0021**	**1**	**U**	**K.DVFLGSFLYEYSR.R**
	4838	862.9150	1723.8154	1723.8273	-0.0119	0	(95)	6.9e-07	1		R.MPCTEDYLSLILNR.L
	4840	575.6136	1723.8190	1723.8273	-0.0083	0	(50)	0.02	1		R.MPCTEDYLSLILNR.L
	4841	862.9170	1723.8194	1723.8273	-0.0079	0	(92)	1.4e-06	1		R.MPCTEDYLSLILNR.L
	4842	862.9171	1723.8196	1723.8273	-0.0077	0	(98)	3.5e-07	1		R.MPCTEDYLSLILNR.L
	4843	862.9174	1723.8202	1723.8273	-0.0071	0	(84)	9.1e-06	1		R.MPCTEDYLSLILNR.L
	4844	575.6141	1723.8205	1723.8273	-0.0068	0	(49)	0.026	1		R.MPCTEDYLSLILNR.L
	4845	862.9176	1723.8206	1723.8273	-0.0067	0	(99)	3e-07	1		R.MPCTEDYLSLILNR.L
	4846	862.9180	1723.8214	1723.8273	-0.0059	0	(92)	1.4e-06	1		R.MPCTEDYLSLILNR.L
	4847	862.9180	1723.8214	1723.8273	-0.0059	0	(84)	8.2e-06	1		R.MPCTEDYLSLILNR.L
	4848	862.9181	1723.8216	1723.8273	-0.0057	0	99	3e-07	1		R.MPCTEDYLSLILNR.L
	4849	862.9182	1723.8218	1723.8273	-0.0055	0	(86)	5e-06	1		R.MPCTEDYLSLILNR.L
	4850	862.9182	1723.8218	1723.8273	-0.0055	0	(78)	3.6e-05	1		R.MPCTEDYLSLILNR.L
	4851	862.9191	1723.8236	1723.8273	-0.0037	0	(73)	0.00012	1		R.MPCTEDYLSLILNR.L
	4857	863.4149	1724.8152	1723.8273	0.9879	0	(78)	3.5e-05	1		R.MPCTEDYLSLILNR.L
	4861	863.4186	1724.8226	1723.8273	0.9953	0	(80)	1.9e-05	1		R.MPCTEDYLSLILNR.L
	4883	580.9448	1739.8126	1739.8222	-0.0096	0	(51)	0.016	1		R.MPCTEDYLSLILNR.L + Oxidation (M)
	5184	629.9760	1886.9062	1887.9195	-1.0134	0	47	0.043	1		R.HPYFYAPELLYYANK.Y
	5445	1023.0097	2044.0048	2044.0206	-0.0158	1	(48)	0.026	1		R.RHPYFYAPELLYYANK.Y
	5450	1023.0146	2044.0146	2044.0206	-0.0060	1	(51)	0.013	1		R.RHPYFYAPELLYYANK.Y
	5470	1023.5169	2045.0192	2044.0206	0.9986	1	52	0.012	1		R.RHPYFYAPELLYYANK.Y
	5673	1124.4695	2246.9244	2246.9354	-0.0110	1	78	2.5e-05	1		K.ECCHGDLLECADDRADLAK.Y
	5678	1124.9740	2247.9334	2246.9354	0.9980	1	(67)	0.00031	1		K.ECCHGDLLECADDRADLAK.Y
	5679	750.3186	2247.9340	2246.9354	0.9985	1	(46)	0.036	1		K.ECCHGDLLECADDRADLAK.Y
	5932	1244.0533	2486.0920	2486.1028	-0.0108	1	(50)	0.014	1		K.YNGVFQECCQAEDKGACLLPK.I
	5933	1244.0533	2486.0920	2486.1028	-0.0108	1	(56)	0.0036	1		K.YNGVFQECCQAEDKGACLLPK.I
	5934	1244.5452	2487.0758	2486.1028	0.9730	1	(48)	0.022	1		K.YNGVFQECCQAEDKGACLLPK.I
	5935	1244.5475	2487.0804	2486.1028	0.9776	1	71	9.4e-05	1		K.YNGVFQECCQAEDKGACLLPK.I
	5936	830.0377	2487.0913	2486.1028	0.9884	1	(45)	0.041	1		K.YNGVFQECCQAEDKGACLLPK.I
	5938	830.0383	2487.0931	2486.1028	0.9902	1	(44)	0.047	1		K.YNGVFQECCQAEDKGACLLPK.I
☒	6649	**948.1064**	**2841.2974**	**2841.2917**	**0.0056**	**1**	**50**	**0.012**	**1**	**U**	**K.CCAKPESERMPCTEDYLSLILNR.L**

---

3. ALBU_FELCA **Mass:** 70611 **Score:** 5389 **Matches:** 96(96) **Sequences:** 4(4) **emPAI:** 0.19

Albumin OS=Felis catus OX=9685 GN=ALB PE=1 SV=1

☐ Check to include this hit in error tolerant search

Query	Observed	Mr(expt)	Mr(calc)	Delta	Miss	Score	Expect	Rank	Unique	Peptide
4085	1479.7863	1478.7790	1478.7881	-0.0091	0	(80)	2.7e-05	1		K.LGEYGFQNALLVR.Y
4086	740.3972	1478.7798	1478.7881	-0.0083	0	(105)	6.9e-08	1		K.LGEYGFQNALLVR.Y
4087	740.3972	1478.7798	1478.7881	-0.0083	0	(108)	3.4e-08	1		K.LGEYGFQNALLVR.Y
4088	740.3979	1478.7812	1478.7881	-0.0069	0	(102)	1.4e-07	1		K.LGEYGFQNALLVR.Y
4089	740.3981	1478.7816	1478.7881	-0.0065	0	(109)	3.3e-08	1		K.LGEYGFQNALLVR.Y
4090	740.3981	1478.7816	1478.7881	-0.0065	0	112	1.4e-08	1		K.LGEYGFQNALLVR.Y
4091	740.3983	1478.7820	1478.7881	-0.0061	0	(112)	1.7e-08	1		K.LGEYGFQNALLVR.Y
4092	740.3983	1478.7820	1478.7881	-0.0061	0	(105)	6.9e-08	1		K.LGEYGFQNALLVR.Y
4093	740.3985	1478.7824	1478.7881	-0.0057	0	(108)	3.8e-08	1		K.LGEYGFQNALLVR.Y
4094	740.3985	1478.7824	1478.7881	-0.0057	0	(111)	1.7e-08	1		K.LGEYGFQNALLVR.Y
4095	740.3985	1478.7824	1478.7881	-0.0057	0	(102)	1.4e-07	1		K.LGEYGFQNALLVR.Y
4096	1479.7900	1478.7827	1478.7881	-0.0054	0	(74)	9.1e-05	1		K.LGEYGFQNALLVR.Y
4097	740.3990	1478.7834	1478.7881	-0.0047	0	(90)	2.3e-06	1		K.LGEYGFQNALLVR.Y
4098	740.3992	1478.7838	1478.7881	-0.0043	0	(102)	1.4e-07	1		K.LGEYGFQNALLVR.Y
4099	740.3992	1478.7838	1478.7881	-0.0043	0	(109)	3.1e-08	1		K.LGEYGFQNALLVR.Y
4100	740.3995	1478.7844	1478.7881	-0.0037	0	(108)	3.9e-08	1		K.LGEYGFQNALLVR.Y
4101	740.3995	1478.7844	1478.7881	-0.0037	0	(106)	6.4e-08	1		K.LGEYGFQNALLVR.Y
4102	740.3997	1478.7848	1478.7881	-0.0033	0	(106)	6.4e-08	1		K.LGEYGFQNALLVR.Y
4103	740.4001	1478.7856	1478.7881	-0.0025	0	(82)	1.6e-05	1		K.LGEYGFQNALLVR.Y
4104	740.4001	1478.7856	1478.7881	-0.0025	0	(106)	6.4e-08	1		K.LGEYGFQNALLVR.Y
4105	740.4005	1478.7864	1478.7881	-0.0017	0	(106)	6.5e-08	1		K.LGEYGFQNALLVR.Y
4106	740.4006	1478.7866	1478.7881	-0.0015	0	(92)	1.6e-06	1		K.LGEYGFQNALLVR.Y
4107	740.4007	1478.7868	1478.7881	-0.0013	0	(103)	1.3e-07	1		K.LGEYGFQNALLVR.Y
4108	740.4011	1478.7876	1478.7881	-0.0005	0	(85)	7.7e-06	1		K.LGEYGFQNALLVR.Y
4109	740.4012	1478.7878	1478.7881	-0.0003	0	(90)	2.2e-06	1		K.LGEYGFQNALLVR.Y
4110	740.7166	1479.4186	1478.7881	0.6305	0	(90)	1.6e-06	1		K.LGEYGFQNALLVR.Y
4111	740.8925	1479.7704	1478.7881	0.9823	0	(78)	4.2e-05	1		K.LGEYGFQNALLVR.Y
4112	740.8944	1479.7742	1478.7881	0.9861	0	(74)	9.8e-05	1		K.LGEYGFQNALLVR.Y
4113	740.8987	1479.7828	1478.7881	0.9947	0	(83)	1.4e-05	1		K.LGEYGFQNALLVR.Y
4114	494.2688	1479.7846	1478.7881	0.9964	0	(56)	0.0064	1		K.LGEYGFQNALLVR.Y
4115	740.8997	1479.7848	1478.7881	0.9967	0	(87)	4.7e-06	1		K.LGEYGFQNALLVR.Y
4116	740.9005	1479.7864	1478.7881	0.9983	0	(87)	5.1e-06	1		K.LGEYGFQNALLVR.Y
4117	740.9005	1479.7864	1478.7881	0.9983	0	(81)	1.8e-05	1		K.LGEYGFQNALLVR.Y
4118	740.9011	1479.7876	1478.7881	0.9995	0	(79)	2.8e-05	1		K.LGEYGFQNALLVR.Y
4119	740.9017	1479.7888	1478.7881	1.0007	0	(79)	2.8e-05	1		K.LGEYGFQNALLVR.Y
4209	504.6163	1510.8271	1510.8355	-0.0085	0	(67)	0.00047	1		K.VPQVSTPTLVEVSR.S
4214	756.9231	1511.8316	1510.8355	0.9961	0	88	3.8e-06	1		K.VPQVSTPTLVEVSR.S
4611	547.3131	1638.9175	1638.9305	-0.0130	1	(88)	3.9e-06	1		K.KVPQVSTPTLVEVSR.S
4612	547.3142	1638.9208	1638.9305	-0.0097	1	(84)	8e-06	1		K.KVPQVSTPTLVEVSR.S
4613	547.3143	1638.9211	1638.9305	-0.0094	1	(78)	3.7e-05	1		K.KVPQVSTPTLVEVSR.S
4614	547.3143	1638.9211	1638.9305	-0.0094	1	(76)	5.5e-05	1		K.KVPQVSTPTLVEVSR.S
4615	547.3144	1638.9214	1638.9305	-0.0091	1	(94)	8e-07	1		K.KVPQVSTPTLVEVSR.S
4616	547.3144	1638.9214	1638.9305	-0.0091	1	(78)	3.6e-05	1		K.KVPQVSTPTLVEVSR.S
4617	820.4681	1638.9216	1638.9305	-0.0088	1	(125)	6.9e-10	1		K.KVPQVSTPTLVEVSR.S
4618	820.4681	1638.9216	1638.9305	-0.0088	1	(105)	7.6e-08	1		K.KVPQVSTPTLVEVSR.S
4619	820.4681	1638.9216	1638.9305	-0.0088	1	125	6.8e-10	1		K.KVPQVSTPTLVEVSR.S
4620	547.3148	1638.9226	1638.9305	-0.0079	1	(67)	0.00044	1		K.KVPQVSTPTLVEVSR.S
4621	547.3148	1638.9226	1638.9305	-0.0079	1	(74)	9.2e-05	1		K.KVPQVSTPTLVEVSR.S
4622	547.3148	1638.9226	1638.9305	-0.0079	1	(87)	4.3e-06	1		K.KVPQVSTPTLVEVSR.S
4623	820.4686	1638.9226	1638.9305	-0.0078	1	(125)	6.8e-10	1		K.KVPQVSTPTLVEVSR.S

4624	547.3149	1638.9229	1638.9305	-0.0076	1	(66)	0.00057	1		K.KVPQVSTPTLVEVSR.S
4625	547.3149	1638.9229	1638.9305	-0.0076	1	(85)	6.6e-06	1		K.KVPQVSTPTLVEVSR.S
4626	547.3149	1638.9229	1638.9305	-0.0076	1	(89)	2.6e-06	1		K.KVPQVSTPTLVEVSR.S
4627	547.3149	1638.9229	1638.9305	-0.0076	1	(97)	5e-07	1		K.KVPQVSTPTLVEVSR.S
4628	547.3149	1638.9229	1638.9305	-0.0076	1	(92)	1.5e-06	1		K.KVPQVSTPTLVEVSR.S
4629	820.4688	1638.9230	1638.9305	-0.0074	1	(120)	2.2e-09	1		K.KVPQVSTPTLVEVSR.S
4630	547.3150	1638.9232	1638.9305	-0.0073	1	(89)	2.7e-06	1		K.KVPQVSTPTLVEVSR.S
4631	547.3150	1638.9232	1638.9305	-0.0073	1	(85)	7.3e-06	1		K.KVPQVSTPTLVEVSR.S
4632	820.4689	1638.9232	1638.9305	-0.0072	1	(120)	2.2e-09	1		K.KVPQVSTPTLVEVSR.S
4633	820.4690	1638.9234	1638.9305	-0.0070	1	(125)	6.8e-10	1		K.KVPQVSTPTLVEVSR.S
4634	547.3151	1638.9235	1638.9305	-0.0070	1	(86)	5.7e-06	1		K.KVPQVSTPTLVEVSR.S
4635	547.3151	1638.9235	1638.9305	-0.0070	1	(85)	6.8e-06	1		K.KVPQVSTPTLVEVSR.S
4636	547.3151	1638.9235	1638.9305	-0.0070	1	(88)	3.8e-06	1		K.KVPQVSTPTLVEVSR.S
4637	820.4691	1638.9236	1638.9305	-0.0068	1	(120)	2.3e-09	1		K.KVPQVSTPTLVEVSR.S
4638	547.3152	1638.9238	1638.9305	-0.0067	1	(98)	3.7e-07	1		K.KVPQVSTPTLVEVSR.S
4639	547.3152	1638.9238	1638.9305	-0.0067	1	(97)	4.1e-07	1		K.KVPQVSTPTLVEVSR.S
4640	547.3152	1638.9238	1638.9305	-0.0067	1	(90)	2.4e-06	1		K.KVPQVSTPTLVEVSR.S
4641	547.3153	1638.9241	1638.9305	-0.0064	1	(77)	4.3e-05	1		K.KVPQVSTPTLVEVSR.S
4642	547.3153	1638.9241	1638.9305	-0.0064	1	(90)	2.2e-06	1		K.KVPQVSTPTLVEVSR.S
4643	547.3153	1638.9241	1638.9305	-0.0064	1	(76)	5.2e-05	1		K.KVPQVSTPTLVEVSR.S
4644	547.3153	1638.9241	1638.9305	-0.0064	1	(94)	8.7e-07	1		K.KVPQVSTPTLVEVSR.S
4645	547.3153	1638.9241	1638.9305	-0.0064	1	(84)	8.4e-06	1		K.KVPQVSTPTLVEVSR.S
4646	547.3153	1638.9241	1638.9305	-0.0064	1	(90)	2.5e-06	1		K.KVPQVSTPTLVEVSR.S
4647	820.4694	1638.9242	1638.9305	-0.0062	1	(125)	7.8e-10	1		K.KVPQVSTPTLVEVSR.S
4648	820.4696	1638.9246	1638.9305	-0.0058	1	(125)	6.9e-10	1		K.KVPQVSTPTLVEVSR.S
4649	547.3155	1638.9247	1638.9305	-0.0058	1	(88)	4e-06	1		K.KVPQVSTPTLVEVSR.S
4650	820.4698	1638.9250	1638.9305	-0.0054	1	(65)	0.00072	1		K.KVPQVSTPTLVEVSR.S
4651	547.3157	1638.9253	1638.9305	-0.0052	1	(89)	2.8e-06	1		K.KVPQVSTPTLVEVSR.S
4652	547.3160	1638.9262	1638.9305	-0.0043	1	(89)	2.8e-06	1		K.KVPQVSTPTLVEVSR.S
4653	547.3162	1638.9268	1638.9305	-0.0037	1	(87)	4.8e-06	1		K.KVPQVSTPTLVEVSR.S
4655	820.7706	1639.5266	1638.9305	0.5962	1	(123)	7.5e-10	1		K.KVPQVSTPTLVEVSR.S
4656	820.9695	1639.9244	1638.9305	0.9940	1	(104)	9.4e-08	1		K.KVPQVSTPTLVEVSR.S
4657	547.6488	1639.9246	1638.9305	0.9941	1	(77)	4.8e-05	1		K.KVPQVSTPTLVEVSR.S
4658	820.9699	1639.9252	1638.9305	0.9948	1	(104)	8.9e-08	1		K.KVPQVSTPTLVEVSR.S
4659	547.6491	1639.9255	1638.9305	0.9950	1	(98)	3.9e-07	1		K.KVPQVSTPTLVEVSR.S
4660	547.6492	1639.9258	1638.9305	0.9953	1	(81)	1.7e-05	1		K.KVPQVSTPTLVEVSR.S
4661	820.9703	1639.9260	1638.9305	0.9956	1	(104)	8.8e-08	1		K.KVPQVSTPTLVEVSR.S
4662	547.6494	1639.9264	1638.9305	0.9959	1	(83)	1.1e-05	1		K.KVPQVSTPTLVEVSR.S
4663	547.6494	1639.9264	1638.9305	0.9959	1	(85)	7e-06	1		K.KVPQVSTPTLVEVSR.S
4664	820.9708	1639.9270	1638.9305	0.9966	1	(104)	9.3e-08	1		K.KVPQVSTPTLVEVSR.S
4665	820.9711	1639.9276	1638.9305	0.9972	1	(104)	1e-07	1		K.KVPQVSTPTLVEVSR.S
4666	547.6499	1639.9279	1638.9305	0.9974	1	(63)	0.0012	1		K.KVPQVSTPTLVEVSR.S
4667	547.6499	1639.9279	1638.9305	0.9974	1	(96)	6.1e-07	1		K.KVPQVSTPTLVEVSR.S
5673	1124.4695	2246.9244	2246.9354	-0.0110	1	78	2.5e-05	1		K.ECCHGDLLECADDRADLAK.Y
5678	1124.9740	2247.9334	2246.9354	0.9980	1	(67)	0.00031	1		K.ECCHGDLLECADDRADLAK.Y
5679	750.3186	2247.9340	2246.9354	0.9985	1	(46)	0.036	1		K.ECCHGDLLECADDRADLAK.Y

---

4. ALBU_PIG **Mass:** 71643 **Score:** 3121 **Matches:** 88(88) **Sequences:** 7(7) **emPAI:** 0.34

Albumin OS=Sus scrofa OX=9823 GN=ALB PE=1 SV=2

☐ Check to include this hit in error tolerant search

Query	Observed	Mr(expt)	Mr(calc)	Delta	Miss	Score	Expect	Rank	Unique	Peptide
2923	1014.6146	1013.6073	1013.6121	-0.0048	0	(55)	0.011	1		K.QTALVELLK.H
2938	1014.6152	1013.6079	1013.6121	-0.0042	0	60	0.0029	1		K.QTALVELLK.H
2949	1014.6168	1013.6095	1013.6121	-0.0026	0	(57)	0.0063	1		K.QTALVELLK.H
2950	1014.6179	1013.6106	1013.6121	-0.0015	0	(51)	0.027	1		K.QTALVELLK.H
3106	571.8574	1141.7002	1141.7070	-0.0068	1	(62)	0.0018	1		K.KQTALVELLK.H
3108	571.8578	1141.7010	1141.7070	-0.0060	1	(66)	0.00066	1		K.KQTALVELLK.H
3109	571.8579	1141.7012	1141.7070	-0.0058	1	(70)	0.00028	1		K.KQTALVELLK.H
3110	571.8579	1141.7012	1141.7070	-0.0058	1	(70)	0.00027	1		K.KQTALVELLK.H
3113	571.8580	1141.7014	1141.7070	-0.0056	1	(70)	0.00028	1		K.KQTALVELLK.H
3114	571.8582	1141.7018	1141.7070	-0.0052	1	(70)	0.00028	1		K.KQTALVELLK.H
3115	571.8582	1141.7018	1141.7070	-0.0052	1	(67)	0.00055	1		K.KQTALVELLK.H
3116	571.8582	1141.7018	1141.7070	-0.0052	1	(64)	0.0011	1		K.KQTALVELLK.H
3117	571.8582	1141.7018	1141.7070	-0.0052	1	(50)	0.03	1		K.KQTALVELLK.H
3121	571.8583	1141.7020	1141.7070	-0.0050	1	(70)	0.00027	1		K.KQTALVELLK.H
3122	571.8583	1141.7020	1141.7070	-0.0050	1	(67)	0.00054	1		K.KQTALVELLK.H
3123	571.8584	1141.7022	1141.7070	-0.0048	1	(67)	0.00052	1		K.KQTALVELLK.H
3127	571.8585	1141.7024	1141.7070	-0.0046	1	(70)	0.00027	1		K.KQTALVELLK.H
3128	571.8585	1141.7024	1141.7070	-0.0046	1	(66)	0.00066	1		K.KQTALVELLK.H
3129	571.8585	1141.7024	1141.7070	-0.0046	1	(67)	0.00062	1		K.KQTALVELLK.H
3133	571.8588	1141.7030	1141.7070	-0.0040	1	(67)	0.00063	1		K.KQTALVELLK.H
3135	571.8589	1141.7032	1141.7070	-0.0038	1	(51)	0.022	1		K.KQTALVELLK.H
3139	571.8591	1141.7036	1141.7070	-0.0034	1	(65)	0.00093	1		K.KQTALVELLK.H
3140	571.8591	1141.7036	1141.7070	-0.0034	1	(56)	0.0066	1		K.KQTALVELLK.H
3141	571.8593	1141.7040	1141.7070	-0.0030	1	70	0.00027	1		K.KQTALVELLK.H
4085	1479.7863	1478.7790	1478.7881	-0.0091	0	(80)	2.7e-05	1		K.LGEYGFQNALIVR.Y
4086	740.3972	1478.7798	1478.7881	-0.0083	0	(105)	6.9e-08	1		K.LGEYGFQNALIVR.Y
4087	740.3972	1478.7798	1478.7881	-0.0083	0	(108)	3.4e-08	1		K.LGEYGFQNALIVR.Y
4088	740.3979	1478.7812	1478.7881	-0.0069	0	(102)	1.4e-07	1		K.LGEYGFQNALIVR.Y
4089	740.3981	1478.7816	1478.7881	-0.0065	0	(109)	3.3e-08	1		K.LGEYGFQNALIVR.Y
4090	740.3981	1478.7816	1478.7881	-0.0065	0	112	1.4e-08	1		K.LGEYGFQNALIVR.Y
4091	740.3983	1478.7820	1478.7881	-0.0061	0	(112)	1.7e-08	1		K.LGEYGFQNALIVR.Y
4092	740.3983	1478.7820	1478.7881	-0.0061	0	(105)	6.9e-08	1		K.LGEYGFQNALIVR.Y
4093	740.3985	1478.7824	1478.7881	-0.0057	0	(108)	3.8e-08	1		K.LGEYGFQNALIVR.Y
4094	740.3985	1478.7824	1478.7881	-0.0057	0	(111)	1.7e-08	1		K.LGEYGFQNALIVR.Y
4095	740.3985	1478.7824	1478.7881	-0.0057	0	(102)	1.4e-07	1		K.LGEYGFQNALIVR.Y
4096	1479.7900	1478.7827	1478.7881	-0.0054	0	(74)	9.1e-05	1		K.LGEYGFQNALIVR.Y
4097	740.3990	1478.7834	1478.7881	-0.0047	0	(90)	2.3e-06	1		K.LGEYGFQNALIVR.Y
4098	740.3992	1478.7838	1478.7881	-0.0043	0	(102)	1.4e-07	1		K.LGEYGFQNALIVR.Y
4099	740.3992	1478.7838	1478.7881	-0.0043	0	(109)	3.1e-08	1		K.LGEYGFQNALIVR.Y
4100	740.3995	1478.7844	1478.7881	-0.0037	0	(108)	3.9e-08	1		K.LGEYGFQNALIVR.Y
4101	740.3995	1478.7844	1478.7881	-0.0037	0	(106)	6.4e-08	1		K.LGEYGFQNALIVR.Y
4102	740.3997	1478.7848	1478.7881	-0.0033	0	(106)	6.4e-08	1		K.LGEYGFQNALIVR.Y
4103	740.4001	1478.7856	1478.7881	-0.0025	0	(82)	1.6e-05	1		K.LGEYGFQNALIVR.Y
4104	740.4001	1478.7856	1478.7881	-0.0025	0	(106)	6.4e-08	1		K.LGEYGFQNALIVR.Y
4105	740.4005	1478.7864	1478.7881	-0.0017	0	(106)	6.5e-08	1		K.LGEYGFQNALIVR.Y
4106	740.4006	1478.7866	1478.7881	-0.0015	0	(92)	1.6e-06	1		K.LGEYGFQNALIVR.Y
4107	740.4007	1478.7868	1478.7881	-0.0013	0	(103)	1.3e-07	1		K.LGEYGFQNALIVR.Y
4108	740.4011	1478.7876	1478.7881	-0.0005	0	(85)	7.7e-06	1		K.LGEYGFQNALIVR.Y
4109	740.4012	1478.7878	1478.7881	-0.0003	0	(90)	2.2e-06	1		K.LGEYGFQNALIVR.Y
4110	740.7166	1479.4186	1478.7881	0.6305	0	(90)	1.6e-06	1		K.LGEYGFQNALIVR.Y
4111	740.8925	1479.7704	1478.7881	0.9823	0	(78)	4.2e-05	1		K.LGEYGFQNALIVR.Y
4112	740.8944	1479.7742	1478.7881	0.9861	0	(74)	9.8e-05	1		K.LGEYGFQNALIVR.Y
4113	740.8987	1479.7828	1478.7881	0.9947	0	(83)	1.4e-05	1		K.LGEYGFQNALIVR.Y

4114	494.2688	1479.7846	1478.7881	0.9964	0	(56)	0.0064	1		K.LGEYGFQNALIVR.Y
4115	740.8997	1479.7848	1478.7881	0.9967	0	(87)	4.7e-06	1		K.LGEYGFQNALIVR.Y
4116	740.9005	1479.7864	1478.7881	0.9983	0	(87)	5.1e-06	1		K.LGEYGFQNALIVR.Y
4117	740.9005	1479.7864	1478.7881	0.9983	0	(81)	1.8e-05	1		K.LGEYGFQNALIVR.Y
4118	740.9011	1479.7876	1478.7881	0.9995	0	(79)	2.8e-05	1		K.LGEYGFQNALIVR.Y
4119	740.9017	1479.7888	1478.7881	1.0007	0	(79)	2.8e-05	1		K.LGEYGFQNALIVR.Y
4252	766.8900	1531.7654	1531.7738	-0.0084	1	(58)	0.0042	1		K.LKECCDKPLLEK.S
4257	511.5960	1531.7662	1531.7738	-0.0076	1	(56)	0.0066	1		K.LKECCDKPLLEK.S
4258	511.5960	1531.7662	1531.7738	-0.0076	1	(61)	0.0019	1		K.LKECCDKPLLEK.S
4260	766.8904	1531.7662	1531.7738	-0.0076	1	(66)	0.00068	1		K.LKECCDKPLLEK.S
4261	511.5961	1531.7665	1531.7738	-0.0073	1	(59)	0.0032	1		K.LKECCDKPLLEK.S
4265	766.8911	1531.7676	1531.7738	-0.0062	1	68	0.00038	1		K.LKECCDKPLLEK.S
4266	766.8911	1531.7676	1531.7738	-0.0062	1	(59)	0.0034	1		K.LKECCDKPLLEK.S
4267	511.5965	1531.7677	1531.7738	-0.0061	1	(51)	0.019	1		K.LKECCDKPLLEK.S
4268	511.5967	1531.7683	1531.7738	-0.0055	1	(57)	0.0054	1		K.LKECCDKPLLEK.S
4269	511.5968	1531.7686	1531.7738	-0.0052	1	(55)	0.0071	1		K.LKECCDKPLLEK.S
4270	511.5968	1531.7686	1531.7738	-0.0052	1	(54)	0.011	1		K.LKECCDKPLLEK.S
4291	770.4079	1538.8012	1538.8127	-0.0114	1	(66)	0.00065	1		R.LCVLHEKTPVSEK.V
4292	513.9421	1538.8045	1538.8127	-0.0082	1	(63)	0.0012	1		R.LCVLHEKTPVSEK.V
4295	513.9423	1538.8051	1538.8127	-0.0076	1	(63)	0.0012	1		R.LCVLHEKTPVSEK.V
4296	513.9423	1538.8051	1538.8127	-0.0076	1	(58)	0.0034	1		R.LCVLHEKTPVSEK.V
4297	770.4099	1538.8052	1538.8127	-0.0074	1	(71)	0.00019	1		R.LCVLHEKTPVSEK.V
4299	513.9424	1538.8054	1538.8127	-0.0073	1	(63)	0.0013	1		R.LCVLHEKTPVSEK.V
4300	770.4100	1538.8054	1538.8127	-0.0072	1	(51)	0.021	1		R.LCVLHEKTPVSEK.V
4301	770.4100	1538.8054	1538.8127	-0.0072	1	71	0.00017	1		R.LCVLHEKTPVSEK.V
4304	385.7087	1538.8057	1538.8127	-0.0070	1	(50)	0.022	1		R.LCVLHEKTPVSEK.V
4305	513.9426	1538.8060	1538.8127	-0.0067	1	(62)	0.0014	1		R.LCVLHEKTPVSEK.V
4306	770.4106	1538.8066	1538.8127	-0.0060	1	(71)	0.0002	1		R.LCVLHEKTPVSEK.V
4307	770.4116	1538.8086	1538.8127	-0.0040	1	(59)	0.003	1		R.LCVLHEKTPVSEK.V
4309	770.9101	1539.8056	1538.8127	0.9930	1	(54)	0.0088	1		R.LCVLHEKTPVSEK.V
4311	514.2764	1539.8074	1538.8127	0.9947	1	(62)	0.0015	1		R.LCVLHEKTPVSEK.V
5673	1124.4695	2246.9244	2246.9354	-0.0110	1	78	2.5e-05	1		K.ECCHGDLLECADDRADLAK.Y
5678	1124.9740	2247.9334	2246.9354	0.9980	1	(67)	0.00031	1		K.ECCHGDLLECADDRADLAK.Y
5679	750.3186	2247.9340	2246.9354	0.9985	1	(46)	0.036	1		K.ECCHGDLLECADDRADLAK.Y
☒ 6061	843.7262	2528.1568	2527.2529	0.9039	1	53	0.0073	1	U	K.QNCELFEKLGEYGFQNALIVR.Y

---

5. ALBU_CANLF **Mass:** 70556 **Score:** 2496 **Matches:** 62(62) **Sequences:** 4(4) **emPAI:** 0.19

Albumin OS=Canis lupus familiaris OX=9615 GN=ALB PE=1 SV=3

☐ Check to include this hit in error tolerant search

Query	Observed	Mr(expt)	Mr(calc)	Delta	Miss	Score	Expect	Rank	Unique	Peptide
2923	1014.6146	1013.6073	1013.6121	-0.0048	0	(55)	0.011	1		K.QTALVELLK.H
2938	1014.6152	1013.6079	1013.6121	-0.0042	0	60	0.0029	1		K.QTALVELLK.H
2949	1014.6168	1013.6095	1013.6121	-0.0026	0	(57)	0.0063	1		K.QTALVELLK.H
2950	1014.6179	1013.6106	1013.6121	-0.0015	0	(51)	0.027	1		K.QTALVELLK.H
3106	571.8574	1141.7002	1141.7070	-0.0068	1	(62)	0.0018	1		K.KQTALVELLK.H
3108	571.8578	1141.7010	1141.7070	-0.0060	1	(66)	0.00066	1		K.KQTALVELLK.H
3109	571.8579	1141.7012	1141.7070	-0.0058	1	(70)	0.00028	1		K.KQTALVELLK.H
3110	571.8579	1141.7012	1141.7070	-0.0058	1	(70)	0.00027	1		K.KQTALVELLK.H
3113	571.8580	1141.7014	1141.7070	-0.0056	1	(70)	0.00028	1		K.KQTALVELLK.H
3114	571.8582	1141.7018	1141.7070	-0.0052	1	(70)	0.00028	1		K.KQTALVELLK.H
3115	571.8582	1141.7018	1141.7070	-0.0052	1	(67)	0.00055	1		K.KQTALVELLK.H
3116	571.8582	1141.7018	1141.7070	-0.0052	1	(64)	0.0011	1		K.KQTALVELLK.H
3117	571.8582	1141.7018	1141.7070	-0.0052	1	(50)	0.03	1		K.KQTALVELLK.H
3121	571.8583	1141.7020	1141.7070	-0.0050	1	(70)	0.00027	1		K.KQTALVELLK.H
3122	571.8583	1141.7020	1141.7070	-0.0050	1	(67)	0.00054	1		K.KQTALVELLK.H
3123	571.8584	1141.7022	1141.7070	-0.0048	1	(67)	0.00052	1		K.KQTALVELLK.H
3127	571.8585	1141.7024	1141.7070	-0.0046	1	(70)	0.00027	1		K.KQTALVELLK.H
3128	571.8585	1141.7024	1141.7070	-0.0046	1	(66)	0.00066	1		K.KQTALVELLK.H
3129	571.8585	1141.7024	1141.7070	-0.0046	1	(67)	0.00062	1		K.KQTALVELLK.H
3133	571.8588	1141.7030	1141.7070	-0.0040	1	(67)	0.00063	1		K.KQTALVELLK.H
3135	571.8589	1141.7032	1141.7070	-0.0038	1	(51)	0.022	1		K.KQTALVELLK.H
3139	571.8591	1141.7036	1141.7070	-0.0034	1	(65)	0.00093	1		K.KQTALVELLK.H
3140	571.8591	1141.7036	1141.7070	-0.0034	1	(56)	0.0066	1		K.KQTALVELLK.H
3141	571.8593	1141.7040	1141.7070	-0.0030	1	70	0.00027	1		K.KQTALVELLK.H
4085	1479.7863	1478.7790	1478.7881	-0.0091	0	(80)	2.7e-05	1		K.LGEYGFQNALLVR.Y
4086	740.3972	1478.7798	1478.7881	-0.0083	0	(105)	6.9e-08	1		K.LGEYGFQNALLVR.Y
4087	740.3972	1478.7798	1478.7881	-0.0083	0	(108)	3.4e-08	1		K.LGEYGFQNALLVR.Y
4088	740.3979	1478.7812	1478.7881	-0.0069	0	(102)	1.4e-07	1		K.LGEYGFQNALLVR.Y
4089	740.3981	1478.7816	1478.7881	-0.0065	0	(109)	3.3e-08	1		K.LGEYGFQNALLVR.Y
4090	740.3981	1478.7816	1478.7881	-0.0065	0	112	1.4e-08	1		K.LGEYGFQNALLVR.Y
4091	740.3983	1478.7820	1478.7881	-0.0061	0	(112)	1.7e-08	1		K.LGEYGFQNALLVR.Y
4092	740.3983	1478.7820	1478.7881	-0.0061	0	(105)	6.9e-08	1		K.LGEYGFQNALLVR.Y
4093	740.3985	1478.7824	1478.7881	-0.0057	0	(108)	3.8e-08	1		K.LGEYGFQNALLVR.Y
4094	740.3985	1478.7824	1478.7881	-0.0057	0	(111)	1.7e-08	1		K.LGEYGFQNALLVR.Y
4095	740.3985	1478.7824	1478.7881	-0.0057	0	(102)	1.4e-07	1		K.LGEYGFQNALLVR.Y
4096	1479.7900	1478.7827	1478.7881	-0.0054	0	(74)	9.1e-05	1		K.LGEYGFQNALLVR.Y
4097	740.3990	1478.7834	1478.7881	-0.0047	0	(90)	2.3e-06	1		K.LGEYGFQNALLVR.Y
4098	740.3992	1478.7838	1478.7881	-0.0043	0	(102)	1.4e-07	1		K.LGEYGFQNALLVR.Y
4099	740.3992	1478.7838	1478.7881	-0.0043	0	(109)	3.1e-08	1		K.LGEYGFQNALLVR.Y
4100	740.3995	1478.7844	1478.7881	-0.0037	0	(108)	3.9e-08	1		K.LGEYGFQNALLVR.Y
4101	740.3995	1478.7844	1478.7881	-0.0037	0	(106)	6.4e-08	1		K.LGEYGFQNALLVR.Y
4102	740.3997	1478.7848	1478.7881	-0.0033	0	(106)	6.4e-08	1		K.LGEYGFQNALLVR.Y
4103	740.4001	1478.7856	1478.7881	-0.0025	0	(82)	1.6e-05	1		K.LGEYGFQNALLVR.Y
4104	740.4001	1478.7856	1478.7881	-0.0025	0	(106)	6.4e-08	1		K.LGEYGFQNALLVR.Y
4105	740.4005	1478.7864	1478.7881	-0.0017	0	(106)	6.5e-08	1		K.LGEYGFQNALLVR.Y
4106	740.4006	1478.7866	1478.7881	-0.0015	0	(92)	1.6e-06	1		K.LGEYGFQNALLVR.Y
4107	740.4007	1478.7868	1478.7881	-0.0013	0	(103)	1.3e-07	1		K.LGEYGFQNALLVR.Y
4108	740.4011	1478.7876	1478.7881	-0.0005	0	(85)	7.7e-06	1		K.LGEYGFQNALLVR.Y
4109	740.4012	1478.7878	1478.7881	-0.0003	0	(90)	2.2e-06	1		K.LGEYGFQNALLVR.Y
4110	740.7166	1479.4186	1478.7881	0.6305	0	(90)	1.6e-06	1		K.LGEYGFQNALLVR.Y
4111	740.8925	1479.7704	1478.7881	0.9823	0	(78)	4.2e-05	1		K.LGEYGFQNALLVR.Y
4112	740.8944	1479.7742	1478.7881	0.9861	0	(74)	9.8e-05	1		K.LGEYGFQNALLVR.Y
4113	740.8987	1479.7828	1478.7881	0.9947	0	(83)	1.4e-05	1		K.LGEYGFQNALLVR.Y
4114	494.2688	1479.7846	1478.7881	0.9964	0	(56)	0.0064	1		K.LGEYGFQNALLVR.Y
4115	740.8997	1479.7848	1478.7881	0.9967	0	(87)	4.7e-06	1		K.LGEYGFQNALLVR.Y
4116	740.9005	1479.7864	1478.7881	0.9983	0	(87)	5.1e-06	1		K.LGEYGFQNALLVR.Y
4117	740.9005	1479.7864	1478.7881	0.9983	0	(81)	1.8e-05	1		K.LGEYGFQNALLVR.Y
4118	740.9011	1479.7876	1478.7881	0.9995	0	(79)	2.8e-05	1		K.LGEYGFQNALLVR.Y
4119	740.9017	1479.7888	1478.7881	1.0007	0	(79)	2.8e-05	1		K.LGEYGFQNALLVR.Y
5673	1124.4695	2246.9244	2246.9354	-0.0110	1	78	2.5e-05	1		K.ECCHGDLLECADDRADLAK.Y
5678	1124.9740	2247.9334	2246.9354	0.9980	1	(67)	0.00031	1		K.ECCHGDLLECADDRADLAK.Y
5679	750.3186	2247.9340	2246.9354	0.9985	1	(46)	0.036	1		K.ECCHGDLLECADDRADLAK.Y

---

6. ALBU_MESAU **Mass:** 70177 **Score:** 2076 **Matches:** 37(37) **Sequences:** 2(2) **emPAI:** 0.09
Albumin OS=Mesocricetus auratus OX=10036 GN=ALB PE=1 SV=1
☐ Check to include this hit in error tolerant search

Query	Observed	Mr(expt)	Mr(calc)	Delta	Miss	Score	Expect	Rank	Unique	Peptide
4085	1479.7863	1478.7790	1478.7881	-0.0091	0	(80)	2.7e-05	1		K.LGEYGFQNALIVR.Y
4086	740.3972	1478.7798	1478.7881	-0.0083	0	(105)	6.9e-08	1		K.LGEYGFQNALIVR.Y
4087	740.3972	1478.7798	1478.7881	-0.0083	0	(108)	3.4e-08	1		K.LGEYGFQNALIVR.Y
4088	740.3979	1478.7812	1478.7881	-0.0069	0	(102)	1.4e-07	1		K.LGEYGFQNALIVR.Y
4089	740.3981	1478.7816	1478.7881	-0.0065	0	(109)	3.3e-08	1		K.LGEYGFQNALIVR.Y
4090	740.3981	1478.7816	1478.7881	-0.0065	0	112	1.4e-08	1		K.LGEYGFQNALIVR.Y
4091	740.3983	1478.7820	1478.7881	-0.0061	0	(112)	1.7e-08	1		K.LGEYGFQNALIVR.Y
4092	740.3983	1478.7820	1478.7881	-0.0061	0	(105)	6.9e-08	1		K.LGEYGFQNALIVR.Y
4093	740.3985	1478.7824	1478.7881	-0.0057	0	(108)	3.8e-08	1		K.LGEYGFQNALIVR.Y
4094	740.3985	1478.7824	1478.7881	-0.0057	0	(111)	1.7e-08	1		K.LGEYGFQNALIVR.Y
4095	740.3985	1478.7824	1478.7881	-0.0057	0	(102)	1.4e-07	1		K.LGEYGFQNALIVR.Y
4096	1479.7900	1478.7827	1478.7881	-0.0054	0	(74)	9.1e-05	1		K.LGEYGFQNALIVR.Y
4097	740.3990	1478.7834	1478.7881	-0.0047	0	(90)	2.3e-06	1		K.LGEYGFQNALIVR.Y
4098	740.3992	1478.7838	1478.7881	-0.0043	0	(102)	1.4e-07	1		K.LGEYGFQNALIVR.Y
4099	740.3992	1478.7838	1478.7881	-0.0043	0	(109)	3.1e-08	1		K.LGEYGFQNALIVR.Y
4100	740.3995	1478.7844	1478.7881	-0.0037	0	(108)	3.9e-08	1		K.LGEYGFQNALIVR.Y
4101	740.3995	1478.7844	1478.7881	-0.0037	0	(106)	6.4e-08	1		K.LGEYGFQNALIVR.Y
4102	740.3997	1478.7848	1478.7881	-0.0033	0	(106)	6.4e-08	1		K.LGEYGFQNALIVR.Y
4103	740.4001	1478.7856	1478.7881	-0.0025	0	(82)	1.6e-05	1		K.LGEYGFQNALIVR.Y
4104	740.4001	1478.7856	1478.7881	-0.0025	0	(106)	6.4e-08	1		K.LGEYGFQNALIVR.Y
4105	740.4005	1478.7864	1478.7881	-0.0017	0	(106)	6.5e-08	1		K.LGEYGFQNALIVR.Y
4106	740.4006	1478.7866	1478.7881	-0.0015	0	(92)	1.6e-06	1		K.LGEYGFQNALIVR.Y
4107	740.4007	1478.7868	1478.7881	-0.0013	0	(103)	1.3e-07	1		K.LGEYGFQNALIVR.Y
4108	740.4011	1478.7876	1478.7881	-0.0005	0	(85)	7.7e-06	1		K.LGEYGFQNALIVR.Y
4109	740.4012	1478.7878	1478.7881	-0.0003	0	(90)	2.2e-06	1		K.LGEYGFQNALIVR.Y
4110	740.7166	1479.4186	1478.7881	0.6305	0	(90)	1.6e-06	1		K.LGEYGFQNALIVR.Y
4111	740.8925	1479.7704	1478.7881	0.9823	0	(78)	4.2e-05	1		K.LGEYGFQNALIVR.Y
4112	740.8944	1479.7742	1478.7881	0.9861	0	(74)	9.8e-05	1		K.LGEYGFQNALIVR.Y
4113	740.8987	1479.7828	1478.7881	0.9947	0	(83)	1.4e-05	1		K.LGEYGFQNALIVR.Y
4114	494.2688	1479.7846	1478.7881	0.9964	0	(56)	0.0064	1		K.LGEYGFQNALIVR.Y
4115	740.8997	1479.7848	1478.7881	0.9967	0	(87)	4.7e-06	1		K.LGEYGFQNALIVR.Y
4116	740.9005	1479.7864	1478.7881	0.9983	0	(87)	5.1e-06	1		K.LGEYGFQNALIVR.Y
4117	740.9005	1479.7864	1478.7881	0.9983	0	(81)	1.8e-05	1		K.LGEYGFQNALIVR.Y
4118	740.9011	1479.7876	1478.7881	0.9995	0	(79)	2.8e-05	1		K.LGEYGFQNALIVR.Y
4119	740.9017	1479.7888	1478.7881	1.0007	0	(79)	2.8e-05	1		K.LGEYGFQNALIVR.Y
☒ 5898	**824.2992**	**2469.8758**	**2470.2314**	**-0.3557**	**1**	**(81)**	**8.5e-06**	**1**	**U**	**K.ANCELFEKLGEYGFQNALIVR.Y**
☒ 5903	**824.7279**	**2471.1619**	**2470.2314**	**0.9304**	**1**	**102**	**7.9e-08**	**1**	**U**	**K.ANCELFEKLGEYGFQNALIVR.Y**

7. ALBU_MOUSE **Mass:** 70700 **Score:** 1955 **Matches:** 35(35) **Sequences:** 1(1) **emPAI:** 0.04
Albumin OS=Mus musculus OX=10090 GN=Alb PE=1 SV=3
☐ Check to include this hit in error tolerant search

Query	Observed	Mr(expt)	Mr(calc)	Delta	Miss	Score	Expect	Rank	Unique	Peptide
4085	1479.7863	1478.7790	1478.7881	-0.0091	0	(80)	2.7e-05	1	U	K.LGEYGFQNAILVR.Y
4086	740.3972	1478.7798	1478.7881	-0.0083	0	(105)	6.9e-08	1	U	K.LGEYGFQNAILVR.Y
4087	740.3972	1478.7798	1478.7881	-0.0083	0	(108)	3.4e-08	1	U	K.LGEYGFQNAILVR.Y
4088	740.3979	1478.7812	1478.7881	-0.0069	0	(102)	1.4e-07	1	U	K.LGEYGFQNAILVR.Y
4089	740.3981	1478.7816	1478.7881	-0.0065	0	(109)	3.3e-08	1	U	K.LGEYGFQNAILVR.Y
4090	740.3981	1478.7816	1478.7881	-0.0065	0	112	1.4e-08	1	U	K.LGEYGFQNAILVR.Y
4091	740.3983	1478.7820	1478.7881	-0.0061	0	(112)	1.7e-08	1	U	K.LGEYGFQNAILVR.Y
4092	740.3983	1478.7820	1478.7881	-0.0061	0	(105)	6.9e-08	1	U	K.LGEYGFQNAILVR.Y
4093	740.3985	1478.7824	1478.7881	-0.0057	0	(108)	3.8e-08	1	U	K.LGEYGFQNAILVR.Y
4094	740.3985	1478.7824	1478.7881	-0.0057	0	(111)	1.7e-08	1	U	K.LGEYGFQNAILVR.Y
4095	740.3985	1478.7824	1478.7881	-0.0057	0	(102)	1.4e-07	1	U	K.LGEYGFQNAILVR.Y
4096	1479.7900	1478.7827	1478.7881	-0.0054	0	(74)	9.1e-05	1	U	K.LGEYGFQNAILVR.Y
4097	740.3990	1478.7834	1478.7881	-0.0047	0	(90)	2.3e-06	1	U	K.LGEYGFQNAILVR.Y
4098	740.3992	1478.7838	1478.7881	-0.0043	0	(102)	1.4e-07	1	U	K.LGEYGFQNAILVR.Y
4099	740.3992	1478.7838	1478.7881	-0.0043	0	(109)	3.1e-08	1	U	K.LGEYGFQNAILVR.Y
4100	740.3995	1478.7844	1478.7881	-0.0037	0	(108)	3.9e-08	1	U	K.LGEYGFQNAILVR.Y
4101	740.3995	1478.7844	1478.7881	-0.0037	0	(106)	6.4e-08	1	U	K.LGEYGFQNAILVR.Y
4102	740.3997	1478.7848	1478.7881	-0.0033	0	(106)	6.4e-08	1	U	K.LGEYGFQNAILVR.Y
4103	740.4001	1478.7856	1478.7881	-0.0025	0	(82)	1.6e-05	1	U	K.LGEYGFQNAILVR.Y
4104	740.4001	1478.7856	1478.7881	-0.0025	0	(106)	6.4e-08	1	U	K.LGEYGFQNAILVR.Y
4105	740.4005	1478.7864	1478.7881	-0.0017	0	(106)	6.5e-08	1	U	K.LGEYGFQNAILVR.Y
4106	740.4006	1478.7866	1478.7881	-0.0015	0	(92)	1.6e-06	1	U	K.LGEYGFQNAILVR.Y
4107	740.4007	1478.7868	1478.7881	-0.0013	0	(103)	1.3e-07	1	U	K.LGEYGFQNAILVR.Y
4108	740.4011	1478.7876	1478.7881	-0.0005	0	(85)	7.7e-06	1	U	K.LGEYGFQNAILVR.Y
4109	740.4012	1478.7878	1478.7881	-0.0003	0	(90)	2.2e-06	1	U	K.LGEYGFQNAILVR.Y
4110	740.7166	1479.4186	1478.7881	0.6305	0	(90)	1.6e-06	1	U	K.LGEYGFQNAILVR.Y
4111	740.8925	1479.7704	1478.7881	0.9823	0	(78)	4.2e-05	1	U	K.LGEYGFQNAILVR.Y
4112	740.8944	1479.7742	1478.7881	0.9861	0	(74)	9.8e-05	1	U	K.LGEYGFQNAILVR.Y
4113	740.8987	1479.7828	1478.7881	0.9947	0	(83)	1.4e-05	1	U	K.LGEYGFQNAILVR.Y
4114	494.2688	1479.7846	1478.7881	0.9964	0	(56)	0.0064	1	U	K.LGEYGFQNAILVR.Y
4115	740.8997	1479.7848	1478.7881	0.9967	0	(87)	4.7e-06	1	U	K.LGEYGFQNAILVR.Y
4116	740.9005	1479.7864	1478.7881	0.9983	0	(87)	5.1e-06	1	U	K.LGEYGFQNAILVR.Y
4117	740.9005	1479.7864	1478.7881	0.9983	0	(81)	1.8e-05	1	U	K.LGEYGFQNAILVR.Y
4118	740.9011	1479.7876	1478.7881	0.9995	0	(79)	2.8e-05	1	U	K.LGEYGFQNAILVR.Y
4119	740.9017	1479.7888	1478.7881	1.0007	0	(79)	2.8e-05	1	U	K.LGEYGFQNAILVR.Y

8. A1AG_BOVIN **Mass:** 23453 **Score:** 354 **Matches:** 9(9) **Sequences:** 2(2) **emPAI:** 0.29
Alpha-1-acid glycoprotein OS=Bos taurus OX=9913 GN=ORM1 PE=2 SV=1
☐ Check to include this hit in error tolerant search

Query	Observed	Mr(expt)	Mr(calc)	Delta	Miss	Score	Expect	Rank	Unique	Peptide
☒ 3824	**475.9203**	**1424.7391**	**1424.7452**	**-0.0061**	**0**	**(78)**	**4.2e-05**	**1**	**U**	**R.AIQAAFFYLEPR.H**
☒ 3825	**713.3770**	**1424.7394**	**1424.7452**	**-0.0058**	**0**	**(66)**	**0.00061**	**1**	**U**	**R.AIQAAFFYLEPR.H**
☒ 3826	**713.3771**	**1424.7396**	**1424.7452**	**-0.0056**	**0**	**(76)**	**7e-05**	**1**	**U**	**R.AIQAAFFYLEPR.H**
☒ 3827	**475.9209**	**1424.7409**	**1424.7452**	**-0.0043**	**0**	**82**	**1.6e-05**	**1**	**U**	**R.AIQAAFFYLEPR.H**
☒ 3828	**1425.7491**	**1424.7418**	**1424.7452**	**-0.0034**	**0**	**(73)**	**0.00013**	**1**	**U**	**R.AIQAAFFYLEPR.H**
☒ 3829	**713.5464**	**1425.0782**	**1424.7452**	**0.3330**	**0**	**(53)**	**0.0092**	**1**	**U**	**R.AIQAAFFYLEPR.H**
☒ 5589	**723.0342**	**2166.0808**	**2166.0957**	**-0.0149**	**1**	**85**	**5.1e-06**	**1**	**U**	**K.NVGVSFYADKPEVTQEQKK.E**
☒ 5590	**723.3701**	**2167.0885**	**2166.0957**	**0.9928**	**1**	**(80)**	**1.6e-05**	**1**	**U**	**K.NVGVSFYADKPEVTQEQKK.E**
☒ 5591	**723.3707**	**2167.0903**	**2166.0957**	**0.9946**	**1**	**(51)**	**0.013**	**1**	**U**	**K.NVGVSFYADKPEVTQEQKK.E**

9. K1C9_HUMAN **Mass:** 62255 **Score:** 261 **Matches:** 2(2) **Sequences:** 2(2) **emPAI:** 0.10
Keratin, type I cytoskeletal 9 OS=Homo sapiens OX=9606 GN=KRT9 PE=1 SV=3
☐ Check to include this hit in error tolerant search

Query	Observed	Mr(expt)	Mr(calc)	Delta	Miss	Score	Expect	Rank	Unique	Peptide
☒ 6495	902.3852	2704.1338	2704.1539	-0.0201	0	112	7.2e-09	1	U	R.GGGGSFGYSYGGGSGGGFSASSLGGGFGGGSR.G
☒ 7136	1075.4296	3223.2670	3222.2744	0.9926	0	181	5.2e-16	1	U	R.GGSGGSHGGGSGFGGESGGSYGGGEEASGSGGGYGGGSGK.S

---

10. ALBU_MERUN **Mass:** 70892 **Score:** 249 **Matches:** 11(11) **Sequences:** 1(1) **emPAI:** 0.04
Albumin OS=Meriones unguiculatus OX=10047 GN=ALB PE=2 SV=1
☐ Check to include this hit in error tolerant search

Query	Observed	Mr(expt)	Mr(calc)	Delta	Miss	Score	Expect	Rank	Unique	Peptide
☒ 5096	467.7600	1867.0109	1866.9873	0.0236	1	(48)	0.034	1	U	R.VCLLHEKTPVSEQVTK.C
☒ 5097	934.5132	1867.0118	1866.9873	0.0245	1	(55)	0.0056	1	U	R.VCLLHEKTPVSEQVTK.C
☒ 5101	623.3452	1867.0138	1866.9873	0.0264	1	57	0.0035	1	U	R.VCLLHEKTPVSEQVTK.C
☒ 5104	467.7609	1867.0145	1866.9873	0.0272	1	(48)	0.028	1	U	R.VCLLHEKTPVSEQVTK.C
☒ 5112	467.7612	1867.0157	1866.9873	0.0284	1	(49)	0.027	1	U	R.VCLLHEKTPVSEQVTK.C
☒ 5114	934.5154	1867.0162	1866.9873	0.0289	1	(54)	0.0073	1	U	R.VCLLHEKTPVSEQVTK.C
☒ 5116	467.7618	1867.0181	1866.9873	0.0308	1	(47)	0.042	1	U	R.VCLLHEKTPVSEQVTK.C
☒ 5117	623.6792	1868.0158	1866.9873	1.0284	1	(53)	0.0094	1	U	R.VCLLHEKTPVSEQVTK.C
☒ 5118	468.0113	1868.0161	1866.9873	1.0288	1	(47)	0.04	1	U	R.VCLLHEKTPVSEQVTK.C
☒ 5125	935.0159	1868.0172	1866.9873	1.0299	1	(48)	0.034	1	U	R.VCLLHEKTPVSEQVTK.C
☒ 5126	935.0159	1868.0172	1866.9873	1.0299	1	(52)	0.011	1	U	R.VCLLHEKTPVSEQVTK.C

---

11. TRYP_PIG **Mass:** 25078 **Score:** 212 **Matches:** 6(6) **Sequences:** 3(3) **emPAI:** 0.43
Trypsin OS=Sus scrofa OX=9823 PE=1 SV=1
☐ Check to include this hit in error tolerant search

Query	Observed	Mr(expt)	Mr(calc)	Delta	Miss	Score	Expect	Rank	Unique	Peptide
☒ 2579	421.7564	841.4982	841.5022	-0.0039	0	61	0.0023	1	U	R.VATVSLPR.S
☒ 2580	421.7567	841.4988	841.5022	-0.0033	0	(52)	0.02	1	U	R.VATVSLPR.S
☒ 2581	421.7567	841.4988	841.5022	-0.0033	0	(53)	0.013	1	U	R.VATVSLPR.S
☒ 5644	1106.0535	2210.0924	2210.0967	-0.0043	0	120	1.5e-09	1	U	R.LGEHNIDVLEGNEQFINAAK.I
☒ 5645	1106.5548	2211.0950	2210.0967	0.9983	0	(80)	1.6e-05	1	U	R.LGEHNIDVLEGNEQFINAAK.I
☒ 5717	762.0626	2283.1660	2282.1729	0.9931	0	51	0.014	1	U	K.IITHPNFNGNTLDNDIMLIK.L

---

12. VTDB_BOVIN **Mass:** 54904 **Score:** 171 **Matches:** 5(5) **Sequences:** 4(4) **emPAI:** 0.24
Vitamin D-binding protein OS=Bos taurus OX=9913 GN=GC PE=2 SV=1
☐ Check to include this hit in error tolerant search

Query	Observed	Mr(expt)	Mr(calc)	Delta	Miss	Score	Expect	Rank	Unique	Peptide
☒ 3656	691.8688	1381.7230	1381.7242	-0.0011	0	68	0.00039	1	U	K.VLDQYIFELSR.K
☒ 4204	504.2784	1509.8134	1509.8191	-0.0057	1	55	0.0084	1	U	K.VLDQYIFELSRK.T
☒ 6502	903.4031	2707.1875	2706.2020	0.9854	0	67	0.00024	1	U	K.HQPQEFPTYVEPTNDEICEAFR.K
☒ 6658	713.1446	2848.5493	2848.5586	-0.0093	1	60	0.001	1	U	K.FAQKVPTAHLEDVLPLAEDITTILSK.C
☒ 6661	950.8583	2849.5531	2848.5586	0.9945	1	(49)	0.015	1	U	K.FAQKVPTAHLEDVLPLAEDITTILSK.C

---

13. K2C1_HUMAN **Mass:** 66170 **Score:** 150 **Matches:** 3(3) **Sequences:** 3(3) **emPAI:** 0.15
Keratin, type II cytoskeletal 1 OS=Homo sapiens OX=9606 GN=KRT1 PE=1 SV=6
☐ Check to include this hit in error tolerant search

Query	Observed	Mr(expt)	Mr(calc)	Delta	Miss	Score	Expect	Rank	Unique	Peptide
☒ 3448	651.8582	1301.7018	1301.7078	-0.0060	0	50	0.026	1	U	R.SLDLDSIIAEVK.A
4650	820.4698	1638.9250	1637.8525	1.0725	1	47	0.049	2	U	K.SLNNQFASFIDKVR.F
☒ 6845	978.5079	2932.5019	2931.5090	0.9928	1	136	2.6e-11	1	U	R.FLEQQNQVLQTKWELLQQVDTSTR.T

Proteins matching the same set of peptides:
K2C1_PANTR **Mass:** 65621 **Score:** 150 **Matches:** 3(3) **Sequences:** 3(3)
Keratin, type II cytoskeletal 1 OS=Pan troglodytes OX=9598 GN=KRT1 PE=2 SV=1

---

14. VNN1_BOVIN **Mass:** 57594 **Score:** 88 **Matches:** 1(1) **Sequences:** 1(1) **emPAI:** 0.05
Pantetheinase OS=Bos taurus OX=9913 GN=VNN1 PE=1 SV=1
☐ Check to include this hit in error tolerant search

Query	Observed	Mr(expt)	Mr(calc)	Delta	Miss	Score	Expect	Rank	Unique	Peptide
☒ 5427	678.7285	2033.1637	2032.1721	0.9916	0	88	2.6e-06	1	U	R.LFSLKPTSGPVLTVTLFGR.L

Proteins matching the same set of peptides:
VNN1_HUMAN **Mass:** 57716 **Score:** 88 **Matches:** 1(1) **Sequences:** 1(1)
Pantetheinase OS=Homo sapiens OX=9606 GN=VNN1 PE=1 SV=2

---

15. TRY1_RAT **Score:** 82 **Matches:** 2(2) **Sequences:** 1(1)
Serine protease 1 OS=Rattus norvegicus OX=10116 GN=Prss1 PE=1 SV=1
☐ Check to include this hit in error tolerant search

Query	Observed	Mr(expt)	Mr(calc)	Delta	Miss	Score	Expect	Rank	Unique	Peptide
5644	1106.0535	2210.0924	2210.0967	-0.0043	0	(57)	0.0034	2	U	R.LGEHNINVLEGDEQFINAAK.I
5645	1106.5548	2211.0950	2210.0967	0.9983	0	70	0.00017	2	U	R.LGEHNINVLEGDEQFINAAK.I

---

16. CERU_HUMAN **Mass:** 122983 **Score:** 74 **Matches:** 1(1) **Sequences:** 1(1) **emPAI:** 0.02
Ceruloplasmin OS=Homo sapiens OX=9606 GN=CP PE=1 SV=1
☐ Check to include this hit in error tolerant search

Query	Observed	Mr(expt)	Mr(calc)	Delta	Miss	Score	Expect	Rank	Unique	Peptide
☒ 5766	783.0583	2346.1531	2346.1640	-0.0109	1	74	6e-05	1	U	K.MYYSAVDPTKDIFTGLIGPMK.I

---

17. LPXD_PARMW **Mass:** 36244 **Score:** 57 **Matches:** 2(2) **Sequences:** 1(1) **emPAI:** 0.09
UDP-3-O-acylglucosamine N-acyltransferase OS=Parasynechococcus marenigrum (strain WH8102) OX=84588 GN=lpxD PE=3 SV=1
☐ Check to include this hit in error tolerant search

Query	Observed	Mr(expt)	Mr(calc)	Delta	Miss	Score	Expect	Rank	Unique	Peptide
☒ 2802	501.2960	1000.5774	1000.5301	0.0473	0	(52)	0.021	1	U	K.ALQAGESGLR.W
2803	501.2961	1000.5776	1000.5301	0.0475	0	53	0.017	1	U	K.ALQAGESGLR.W

---

18. MCFM_DICDI **Mass:** 34435 **Score:** 55 **Matches:** 2(2) **Sequences:** 1(1) **emPAI:** 0.09
Mitochondrial substrate carrier family protein M OS=Dictyostelium discoideum OX=44689 GN=mcfM PE=3 SV=1

☐ Check to include this hit in error tolerant search

	Query	Observed	Mr(expt)	Mr(calc)	Delta	Miss	Score	Expect	Rank	Unique	Peptide
☒	3218	589.7822	1177.5498	1176.6040	0.9459	1	51	0.023	1	U	K.NEGIKQFWR.G
☒	3219	589.7823	1177.5500	1176.6040	0.9461	1	(51)	0.023	1	U	K.NEGIKQFWR.G

Peptide matches not assigned to protein hits: (no details means no match)

	Query	Observed	Mr(expt)	Mr(calc)	Delta	Miss	Score	Expect	Rank	Unique	Peptide
☒	2804	501.2961	1000.5776	1000.5818	-0.0041	1	48	0.058	1		ALKAWSVAR
☒	3196	1163.6277	1162.6204	1162.6234	-0.0029	0	47	0.056	1		LVNELTEFAK
☒	3157	577.3481	1152.6816	1152.6867	-0.0050	1	47	0.052	1		IVTDLTKVHK
☒	3178	582.3164	1162.6182	1162.6234	-0.0051	0	47	0.064	1		LVNELTEFAK
☒	2918	507.8106	1013.6066	1013.6121	-0.0054	0	46	0.074	1		QTALVELLK
☒	4852	862.9193	1723.8240	1723.8273	-0.0033	0	46	0.051	1		MPCTEDYLSLILNR
☒	4253	766.8901	1531.7656	1531.7738	-0.0082	1	46	0.06	1		LKECCDKPLLEK
☒	4255	511.5959	1531.7659	1531.7738	-0.0079	1	46	0.06	1		LKECCDKPLLEK
☒	3803	710.7768	1419.5390	1418.6864	0.8526	0	46	0.055	1		SLHTLFGDELCK
☒	4254	766.8902	1531.7658	1531.7738	-0.0080	1	46	0.061	1		LKECCDKPLLEK
☒	4259	511.5960	1531.7662	1531.7738	-0.0076	1	46	0.061	1		LKECCDKPLLEK
☒	3460	653.3575	1304.7004	1304.7088	-0.0084	0	46	0.069	1		HLVDEPQNLIK
☒	3177	582.3164	1162.6182	1162.6234	-0.0051	0	46	0.078	1		LVNELTEFAK
☒	3867	360.7067	1438.7977	1438.8045	-0.0068	1	46	0.066	1		RHPEYAVSVLLR
☒	4886	581.2800	1740.8182	1739.8222	0.9960	0	46	0.057	1		MPCTEDYLSLILNR + Oxidation (M)
☒	5111	467.7611	1867.0153	1866.9873	0.0280	1	45	0.055	1		VCLLHEKTPVSEQVTK
☒	5113	467.7612	1867.0157	1866.9873	0.0284	1	45	0.056	1		VCLLHEKTPVSEQVTK
☒	4884	580.9463	1739.8171	1739.8222	-0.0051	0	45	0.064	1		MPCTEDYLSLILNR + Oxidation (M)
☒	5434	680.3373	2037.9901	2038.0007	-0.0107	0	45	0.058	1		NVGVSFYADKPEVTQEQK
☒	2801	501.2958	1000.5770	1000.5818	-0.0047	1	45	0.11	1		ALKAWSVAR
☒	5128	374.6108	1868.0176	1866.9873	1.0303	1	45	0.063	1		VCLLHEKTPVSEQVTK
☒	5371	505.7444	2018.9485	2018.9619	-0.0134	1	45	0.062	1		LKPDPNTLCDEFKADEK
☒	2606	432.2448	862.4750	862.4800	-0.0050	0	45	0.12	1		EFLDVIK
☒	3474	653.3589	1304.7032	1304.7088	-0.0056	0	45	0.094	1		HLVDEPQNLIK
☒	4455	526.2582	1575.7528	1575.7603	-0.0075	0	45	0.084	1		LKPDPNTLCDEFK
☒	3945	360.7074	1438.8005	1438.8045	-0.0040	1	45	0.086	1		RHPEYAVSVLLR
☒	3461	653.3575	1304.7004	1304.7088	-0.0084	0	45	0.095	1		HLVDEPQNLIK
☒	2608	432.2452	862.4758	862.4800	-0.0042	0	45	0.12	1		EFLDVIK
☒	2609	432.2455	862.4764	862.4800	-0.0036	0	45	0.12	1		EFLDVIK
☒	4595	816.8320	1631.6494	1630.8144	0.8351	0	45	0.071	1		HVFLGTFLYEYSR
☒	3386	428.5724	1282.6954	1282.7034	-0.0080	0	45	0.093	1		HPEYAVSVLLR
☒	2607	432.2452	862.4758	862.4800	-0.0042	0	45	0.13	1		EFLDVIK
☒	5154	940.9610	1879.9074	1879.9138	-0.0064	0	45	0.07	1		RPCFSALTPDETYVPK
☒	5105	467.7609	1867.0145	1866.9873	0.0272	1	44	0.069	1		VCLLHEKTPVSEQVTK
☒	5095	467.7470	1866.9589	1866.9873	-0.0284	1	44	0.071	1		VCLLHEKTPVSEQVTK
☒	4887	581.2803	1740.8191	1739.8222	0.9969	0	44	0.079	1		MPCTEDYLSLILNR + Oxidation (M)
☒	3169	582.3162	1162.6178	1162.6234	-0.0055	0	44	0.11	1		LVNELTEFAK
☒	2931	507.8112	1013.6078	1013.6121	-0.0042	0	44	0.12	1		QTALVELLK
☒	5905	618.8018	2471.1781	2470.1839	0.9942	1	44	0.053	1		RPCFSALTPDETYVPKAFDEK
☒	6009	625.5518	2498.1781	2497.1835	0.9946	1	44	0.054	1		AFDEKLFTFHADICTLPDTEK
☒	3812	710.8494	1419.6842	1418.6864	0.9978	0	44	0.097	1		SLHTLFGDELCK
☒	3481	653.3616	1304.7086	1304.7088	-0.0002	0	44	0.1	1		HLVDEPQNLIK
☒	2921	507.8107	1013.6068	1013.6121	-0.0052	0	44	0.12	1		QTALVELLK
☒	5980	625.3000	2497.1709	2497.1835	-0.0126	1	44	0.054	1		AFDEKLFTFHADICTLPDTEK
☒	3137	1142.7109	1141.7036	1141.7070	-0.0034	1	44	0.12	1		KQTALVELLK
☒	4955	893.9655	1785.9164	1785.9261	-0.0097	1	44	0.088	1		VESDREHFVDLLLSK
☒	5625	550.7786	2199.0853	2198.0929	0.9924	1	44	0.068	1		ATEEQLKTVMENFVAFVDK
☒	5937	830.0380	2487.0922	2486.1028	0.9893	1	44	0.052	1		YNGVFQECCQAEDKGACLLPK
☒	3472	653.3588	1304.7030	1304.7088	-0.0058	0	44	0.11	1		HLVDEPQNLIK
☒	3870	720.4062	1438.7978	1438.8045	-0.0066	1	44	0.1	1		RHPEYAVSVLLR
☒	6001	625.5510	2498.1749	2497.1835	0.9914	1	44	0.06	1		AFDEKLFTFHADICTLPDTEK
☒	3765	473.9004	1418.6794	1418.6864	-0.0070	0	44	0.11	1		SLHTLFGDELCK
☒	3164	582.3156	1162.6166	1162.6234	-0.0067	0	44	0.13	1		LVNELTEFAK
☒	5676	750.3174	2247.9304	2246.9354	0.9949	1	44	0.062	1		ECCHGDLLECADDRADLAK
☒	4960	894.4683	1786.9220	1785.9261	0.9959	1	43	0.098	1		VESDREHFVDLLLSK
☒	3889	480.6068	1438.7986	1438.8045	-0.0059	1	43	0.11	1		RHPEYAVSVLLR
☒	3396	428.9074	1283.7004	1282.7034	0.9970	0	43	0.13	1		HPEYAVSVLLR
☒	3464	653.3584	1304.7022	1304.7088	-0.0066	0	43	0.13	1		HLVDEPQNLIK
☒	5119	468.0113	1868.0161	1866.9873	1.0288	1	43	0.097	1		VCLLHEKTPVSEQVTK
☒	3479	653.3591	1304.7036	1304.7088	-0.0052	0	43	0.14	1		HLVDEPQNLIK
☒	3125	381.5747	1141.7023	1141.6343	0.0680	0	43	0.15	1		NGGAEIVELLK
☒	4862	575.9484	1724.8234	1723.8273	0.9961	0	43	0.11	1		MPCTEDYLSLILNR
☒	4315	385.9598	1539.8101	1538.8127	0.9974	1	43	0.13	1		LCVLHEKTPVSEK
☒	3465	653.3585	1304.7024	1304.7088	-0.0064	0	43	0.15	1		HLVDEPQNLIK
☒	6006	625.5516	2498.1773	2497.1835	0.9938	1	43	0.078	1		AFDEKLFTFHADICTLPDTEK
☒	3860	480.6064	1438.7974	1438.8045	-0.0071	1	43	0.14	1		RHPEYAVSVLLR
☒	5998	625.5507	2498.1737	2497.1835	0.9902	1	42	0.081	1		AFDEKLFTFHADICTLPDTEK
☒	2916	507.8103	1013.6060	1013.6121	-0.0060	0	42	0.19	1		QTALVELLK
☒	5468	1023.5157	2045.0168	2044.0206	0.9962	1	42	0.1	1		RHPYFYAPELLYYANK
☒	2925	507.8110	1013.6074	1013.6121	-0.0046	0	42	0.19	1		QTALVELLK
☒	4899	874.8464	1747.6782	1746.6978	0.9805	0	42	0.11	1		YNGVFQECCQAEDK
☒	2930	507.8112	1013.6078	1013.6121	-0.0042	0	42	0.19	1		QTALVELLK
☒	5130	468.0118	1868.0181	1866.9873	1.0308	1	42	0.11	1		VCLLHEKTPVSEQVTK
☒	6008	833.7331	2498.1775	2497.1835	0.9939	1	42	0.084	1		AFDEKLFTFHADICTLPDTEK
☒	2941	507.8114	1013.6082	1013.6121	-0.0038	0	42	0.19	1		QTALVELLK
☒	2939	507.8114	1013.6082	1013.6121	-0.0038	0	42	0.19	1		QTALVELLK
☒	2944	507.8116	1013.6086	1013.6121	-0.0034	0	42	0.19	1		QTALVELLK
☒	5158	940.9619	1879.9092	1879.9138	-0.0046	0	42	0.12	1		RPCFSALTPDETYVPK
☒	2917	507.8104	1013.6062	1013.6121	-0.0058	0	42	0.2	1		QTALVELLK
☒	5156	940.9614	1879.9082	1879.9138	-0.0056	0	42	0.12	1		RPCFSALTPDETYVPK
☒	2920	507.8107	1013.6068	1013.6121	-0.0052	0	42	0.2	1		QTALVELLK
☒	2940	507.8114	1013.6082	1013.6121	-0.0038	0	42	0.2	1		QTALVELLK
☒	2942	507.8115	1013.6084	1013.6121	-0.0036	0	42	0.2	1		QTALVELLK
☒	2947	507.8119	1013.6092	1013.6121	-0.0028	0	42	0.2	1		QTALVELLK
☒	2932	507.8112	1013.6078	1013.6121	-0.0042	0	42	0.2	1		QTALVELLK
☒	3462	653.3577	1304.7008	1304.7088	-0.0080	0	42	0.17	1		HLVDEPQNLIK
☒	2927	507.8110	1013.6074	1013.6121	-0.0046	0	42	0.2	1		QTALVELLK
☒	2937	507.8112	1013.6078	1013.6121	-0.0042	0	42	0.2	1		QTALVELLK
☒	2935	507.8112	1013.6078	1013.6121	-0.0042	0	42	0.2	1		QTALVELLK
☒	6738	958.4313	2872.2721	2871.3023	0.9698	1	42	0.065	1		CCTKPESERMPCTEDYLSLILNR
☒	3878	360.7068	1438.7981	1438.8045	-0.0064	1	42	0.16	1		RHPEYAVSVLLR
☒	4433	784.8754	1567.7362	1566.7354	1.0008	0	42	0.15	1		DAFLGSFLYEYSR

5115	467.7615	1867.0169	1866.9873	0.0296	1	42	0.13	1	VCLLHEKTPVSEQVTK
5788	786.0499	2355.1279	2354.1325	0.9954	1	42	0.1	1	HLVDEPQNLIKQNCDQFEK
3793	473.9013	1418.6821	1418.6864	-0.0043	0	42	0.18	1	SLHTLFGDELCK
5168	941.4620	1880.9094	1879.9138	0.9956	0	42	0.13	1	RPCFSALTPDETYVPK
6750	958.4401	2872.2985	2871.3023	0.9961	1	42	0.074	1	CCTKPESERMPCTEDYLSLILNR
3107	381.5741	1141.7005	1141.7070	-0.0066	1	42	0.2	1	KQTALVELLK
3697	467.2339	1398.6799	1398.6853	-0.0055	0	42	0.19	1	TVMENFVAFVDK
5133	468.0120	1868.0189	1866.9873	1.0316	1	41	0.14	1	VCLLHEKTPVSEQVTK
4298	385.7086	1538.8053	1538.8127	-0.0074	1	41	0.17	1	LCVLHEKTPVSEK
3906	480.6070	1438.7992	1438.8045	-0.0053	1	41	0.18	1	RHPEYAVSVLLR
3190	582.3170	1162.6194	1162.6234	-0.0039	0	41	0.23	1	LVNELTEFAK
4310	770.9107	1539.8068	1538.8127	0.9942	1	41	0.18	1	LCVLHEKTPVSEK
7028	760.5651	3038.2313	3037.2416	0.9897	1	41	0.062	1	EYEATLEECCAKDDPHACYSTVFDK
3475	653.3589	1304.7032	1304.7088	-0.0056	0	41	0.23	1	HLVDEPQNLIK
3193	582.3173	1162.6200	1162.6234	-0.0033	0	41	0.25	1	LVNELTEFAK
2660	453.3413	904.6680	904.4766	0.1914	0	41	0.27	1	INLENFR
3292	625.3109	1248.6072	1248.6139	-0.0066	1	41	0.24	1	FKDLGEEHFK
3311	625.3114	1248.6082	1248.6139	-0.0056	1	41	0.25	1	FKDLGEEHFK
3124	381.5747	1141.7023	1141.6343	0.0680	0	41	0.25	1	NGGAEIVELLK
2934	507.8112	1013.6078	1013.6121	-0.0042	0	41	0.28	1	QTALVELLK
2936	507.8112	1013.6078	1013.6121	-0.0042	0	41	0.29	1	QTALVELLK
5945	831.4269	2491.2589	2491.2570	0.0019	0	41	0.12	1	GLVLIAFSQYLQQCPFDEHVK
2926	507.8110	1013.6074	1013.6121	-0.0046	0	41	0.29	1	QTALVELLK
6747	719.0807	2872.2937	2871.3023	0.9914	1	41	0.095	1	CCTKPESERMPCTEDYLSLILNR
2946	507.8118	1013.6090	1013.6121	-0.0030	0	40	0.29	1	QTALVELLK
3933	360.7072	1438.7997	1438.8045	-0.0048	1	40	0.23	1	RHPEYAVSVLLR
2933	507.8112	1013.6078	1013.6121	-0.0042	0	40	0.3	1	QTALVELLK
4860	575.9478	1724.8216	1723.8273	0.9943	0	40	0.19	1	MPCTEDYLSLILNR
4393	784.3719	1566.7292	1566.7354	-0.0062	0	40	0.21	1	DAFLGSFLYEYSR
3877	360.7068	1438.7981	1438.8045	-0.0064	1	40	0.23	1	RHPEYAVSVLLR
2948	507.8120	1013.6094	1013.6121	-0.0026	0	40	0.3	1	QTALVELLK
2928	507.8112	1013.6078	1013.6121	-0.0042	0	40	0.3	1	QTALVELLK
5107	374.4102	1867.0146	1866.9873	0.0273	1	40	0.18	1	VCLLHEKTPVSEQVTK
2919	507.8107	1013.6068	1013.6121	-0.0052	0	40	0.31	1	QTALVELLK
6775	722.8287	2887.2857	2887.2972	-0.0115	1	40	0.097	1	CCTKPESERMPCTEDYLSLILNR + Oxidation (M)
2943	507.8116	1013.6086	1013.6121	-0.0034	0	40	0.31	1	QTALVELLK
2922	507.8108	1013.6070	1013.6121	-0.0050	0	40	0.31	1	QTALVELLK
3187	582.3168	1162.6190	1162.6234	-0.0043	0	40	0.29	1	LVNELTEFAK
2929	507.8112	1013.6078	1013.6121	-0.0042	0	40	0.31	1	QTALVELLK
3179	582.3164	1162.6182	1162.6234	-0.0051	0	40	0.29	1	LVNELTEFAK
3905	480.6069	1438.7989	1438.8045	-0.0056	1	40	0.25	1	RHPEYAVSVLLR
6740	958.4367	2872.2883	2871.3023	0.9859	1	40	0.11	1	CCTKPESERMPCTEDYLSLILNR
3322	625.3117	1248.6088	1248.6139	-0.0050	1	40	0.29	1	FKDLGEEHFK
3197	582.3185	1162.6224	1162.6234	-0.0009	0	40	0.3	1	LVNELTEFAK
5218	636.9761	1907.9065	1906.9135	0.9930	0	40	0.2	1	LFTFHADICTLPDTEK
5098	467.7604	1867.0125	1866.9873	0.0252	1	40	0.2	1	VCLLHEKTPVSEQVTK
3797	473.9014	1418.6824	1418.6864	-0.0040	0	40	0.27	1	SLHTLFGDELCK
4193	376.9844	1503.9085	1503.9137	-0.0052	1	40	0.24	1	QTALVELLKHKPK
6007	625.5516	2498.1773	2497.1835	0.9938	1	40	0.15	1	AFDEKLFTFHADICTLPDTEK
4858	575.9473	1724.8201	1723.8273	0.9928	0	40	0.22	1	MPCTEDYLSLILNR
4784	564.9833	1691.9281	1691.9346	-0.0065	1	40	0.24	1	AEFVEVTKLVTDLTK
3200	582.8171	1163.6196	1162.6234	0.9963	0	40	0.33	1	LVNELTEFAK
4835	862.4221	1722.8296	1722.8365	-0.0069	1	40	0.23	1	DAFLGSFLYEYSRR
3794	473.9013	1418.6821	1418.6864	-0.0043	0	40	0.29	1	SLHTLFGDELCK
3795	473.9013	1418.6821	1418.6864	-0.0043	0	40	0.29	1	SLHTLFGDELCK
6013	625.5529	2498.1825	2497.1835	0.9990	1	40	0.16	1	AFDEKLFTFHADICTLPDTEK
3804	474.2336	1419.6790	1418.6864	0.9926	0	40	0.28	1	SLHTLFGDELCK
6779	723.0798	2888.2901	2887.2972	0.9929	1	39	0.12	1	CCTKPESERMPCTEDYLSLILNR + Oxidation (M)
6975	751.1026	3000.3813	2999.3940	0.9873	1	39	0.11	1	CCTESLVNRRPCFSALTPDETYVPK
3201	582.8176	1163.6206	1162.6234	0.9973	0	39	0.34	1	LVNELTEFAK
3188	582.3168	1162.6190	1162.6234	-0.0043	0	39	0.35	1	LVNELTEFAK
3778	473.9007	1418.6803	1418.6864	-0.0061	0	39	0.3	1	SLHTLFGDELCK
3961	360.9575	1439.8009	1438.8045	0.9964	1	39	0.29	1	RHPEYAVSVLLR
4191	376.9840	1503.9069	1503.9137	-0.0068	1	39	0.28	1	QTALVELLKHKPK
3784	473.9009	1418.6809	1418.6864	-0.0055	0	39	0.31	1	SLHTLFGDELCK
3131	381.5748	1141.7026	1141.7070	-0.0045	1	39	0.35	1	KQTALVELLK
3145	381.9093	1142.7061	1141.7070	0.9990	1	39	0.36	1	KQTALVELLK
3332	417.2106	1248.6100	1248.6139	-0.0039	1	39	0.36	1	FKDLGEEHFK
5167	941.4619	1880.9092	1879.9138	0.9954	0	39	0.24	1	RPCFSALTPDETYVPK
5703	760.0544	2277.1414	2276.1511	0.9903	1	39	0.2	1	LFTFHADICTLPDTEKQIK
3331	417.2106	1248.6100	1248.6139	-0.0039	1	39	0.37	1	FKDLGEEHFK
4190	502.3092	1503.9058	1503.9137	-0.0079	1	39	0.31	1	QTALVELLKHKPK
3149	573.7880	1145.5614	1145.5658	-0.0044	0	39	0.42	1	WFYIGSAFR
4610	820.2132	1638.4118	1638.9305	-0.5186	1	39	0.21	1	KVPQVSTPTLVEVSR
3777	473.9007	1418.6803	1418.6864	-0.0061	0	39	0.36	1	SLHTLFGDELCK
5461	1023.5146	2045.0146	2044.0206	0.9940	1	39	0.25	1	RHPYFYAPELLYYANK
2924	507.8110	1013.6074	1013.6121	-0.0046	0	39	0.45	1	QTALVELLK
6010	625.5522	2498.1797	2497.1835	0.9962	1	38	0.2	1	AFDEKLFTFHADICTLPDTEK
6776	723.0762	2888.2757	2887.2972	0.9785	1	38	0.14	1	CCTKPESERMPCTEDYLSLILNR + Oxidation (M)
2945	507.8117	1013.6088	1013.6121	-0.0032	0	38	0.47	1	QTALVELLK
3786	473.9011	1418.6815	1418.6864	-0.0049	0	38	0.38	1	SLHTLFGDELCK
3761	473.9001	1418.6785	1418.6864	-0.0079	0	38	0.39	1	SLHTLFGDELCK
6739	958.4313	2872.2721	2871.3023	0.9698	1	38	0.15	1	CCTKPESERMPCTEDYLSLILNR
5134	468.0126	1868.0213	1866.9873	1.0340	1	38	0.29	1	VCLLHEKTPVSEQVTK
3779	473.9007	1418.6803	1418.6864	-0.0061	0	38	0.4	1	SLHTLFGDELCK
3191	582.3170	1162.6194	1162.6234	-0.0039	0	38	0.46	1	LVNELTEFAK
3310	625.3114	1248.6082	1248.6139	-0.0056	1	38	0.44	1	FKDLGEEHFK
3766	473.9004	1418.6794	1418.6864	-0.0070	0	38	0.41	1	SLHTLFGDELCK
3969	360.9585	1439.8049	1438.8045	1.0004	1	38	0.38	1	RHPEYAVSVLLR
3384	641.9431	1281.8716	1282.6088	-0.7372	0	38	0.33	1	HPEMLGAGNVSR + Oxidation (M)
3304	625.3112	1248.6078	1248.6139	-0.0060	1	38	0.46	1	FKDLGEEHFK
3183	582.3167	1162.6188	1162.6234	-0.0045	0	38	0.49	1	LVNELTEFAK
2799	501.2949	1000.5752	1000.5818	-0.0065	1	38	0.56	1	ALKAWSVAR
2525	395.2376	788.4606	788.4644	-0.0037	0	38	0.5	1	IVTDLTK
5090	621.3197	1860.9373	1860.9469	-0.0096	1	38	0.35	1	GKFPDATETDLQELVAK
5700	759.7214	2276.1424	2276.1511	-0.0087	1	38	0.29	1	LFTFHADICTLPDTEKQIK
3966	360.9579	1439.8025	1438.8045	0.9980	1	38	0.44	1	RHPEYAVSVLLR
5127	468.0116	1868.0173	1866.9873	1.0300	1	38	0.34	1	VCLLHEKTPVSEQVTK
4859	575.9474	1724.8204	1723.8273	0.9931	0	38	0.38	1	MPCTEDYLSLILNR
2524	395.2373	788.4600	788.4644	-0.0043	0	37	0.52	1	IVTDLTK
3195	582.3174	1162.6202	1162.6234	-0.0031	0	37	0.55	1	LVNELTEFAK

☒	6660	950.8498	2849.5276	2848.5586	0.9690	1	37	0.21	1	FAQKVPTAHLEDVLPLAEDITTILSK
☒	5132	468.0119	1868.0185	1866.9873	1.0312	1	37	0.37	1	VCLLHEKTPVSEQVTK
☒	3126	381.5747	1141.7023	1141.7070	-0.0048	1	37	0.56	1	KQTALVELLK
☒	6687	716.8195	2863.2489	2863.2575	-0.0086	1	37	0.2	1	TCVADESHAGCEKSLHTLFGDELCK
☒	6012	625.5527	2498.1817	2497.1835	0.9982	1	37	0.27	1	AFDEKLFTFHADICTLPDTEK
☒	4303	385.7087	1538.8057	1538.8127	-0.0070	1	37	0.45	1	LCVLHEKTPVSEK
☒	3171	582.3162	1162.6178	1162.6234	-0.0055	0	37	0.6	1	LVNELTEFAK
☒	6742	719.0798	2872.2901	2871.3023	0.9878	1	37	0.21	1	CCTKPESERMPCTEDYLSLILNR
☒	2953	509.2930	1016.5714	1017.5277	-0.9562	0	37	0.7	1	SMNDALIVR
☒	3173	582.3162	1162.6178	1162.6234	-0.0055	0	37	0.61	1	LVNELTEFAK
☒	3356	631.3610	1260.7074	1260.6826	0.0248	1	37	0.6	1	SNNNFVIKTPK
☒	3150	573.7883	1145.5620	1145.5658	-0.0038	0	37	0.65	1	WFYIGSAFR
☒	3760	473.8993	1418.6761	1418.6864	-0.0103	0	37	0.54	1	SLHTLFGDELCK
☒	3478	653.3591	1304.7036	1304.7088	-0.0052	0	37	0.57	1	HLVDEPQNLIK
☒	2658	453.3411	904.6676	904.4766	0.1910	0	37	0.68	1	INLENFR
☒	3787	473.9011	1418.6815	1418.6864	-0.0049	0	37	0.56	1	SLHTLFGDELCK
☒	3762	473.9002	1418.6788	1418.6864	-0.0076	0	37	0.58	1	SLHTLFGDELCK
☒	4839	575.6136	1723.8190	1723.8273	-0.0083	0	37	0.46	1	MPCTEDYLSLILNR
☒	5152	470.9839	1879.9065	1879.9138	-0.0073	0	37	0.45	1	RPCFSALTPDETYVPK
☒	3855	360.7064	1438.7965	1438.8045	-0.0080	1	36	0.57	1	RHPEYAVSVLLR
☒	3022	532.7273	1063.4400	1062.5128	0.9272	0	36	0.68	1	TDVCSLVNR
☒	3305	625.3113	1248.6080	1248.6139	-0.0058	1	36	0.68	1	FKDLGEEHFK
☒	3956	360.9572	1439.7997	1438.8045	0.9952	1	36	0.58	1	RHPEYAVSVLLR
☒	4358	777.8281	1553.6416	1553.6457	-0.0040	0	36	0.51	1	DDPHACYSTVFDK
☒	3807	474.2349	1419.6829	1418.6864	0.9965	0	36	0.61	1	SLHTLFGDELCK
☒	3466	653.3585	1304.7024	1304.7088	-0.0064	0	36	0.68	1	HLVDEPQNLIK
☒	6778	723.0798	2888.2901	2887.2972	0.9929	1	36	0.26	1	CCTKPESERMPCTEDYLSLILNR + Oxidation (M)
☒	5153	940.9610	1879.9074	1879.9138	-0.0064	0	36	0.5	1	RPCFSALTPDETYVPK
☒	6737	958.4312	2872.2718	2871.3023	0.9695	1	36	0.26	1	CCTKPESERMPCTEDYLSLILNR
☒	5465	512.2612	2045.0157	2044.0206	0.9951	1	36	0.45	1	RHPYFYAPELLYYANK
☒	5997	625.5507	2498.1737	2497.1835	0.9902	1	36	0.36	1	AFDEKLFTFHADICTLPDTEK
☒	3847	719.8403	1437.6660	1438.8045	-1.1384	1	36	0.64	1	RHPEYAVSVLLR
☒	3168	1163.6249	1162.6176	1162.6234	-0.0057	0	36	0.79	1	LVNELTEFAK
☒	4970	599.6124	1795.8154	1794.8247	0.9907	1	36	0.54	1	DDPHACYSTVFDKLK
☒	3792	473.9012	1418.6818	1418.6864	-0.0046	0	36	0.72	1	SLHTLFGDELCK
☒	3335	625.8124	1249.6102	1248.6139	0.9964	1	36	0.79	1	FKDLGEEHFK
☒	3955	360.9572	1439.7997	1438.8045	0.9952	1	35	0.71	1	RHPEYAVSVLLR
☒	3111	381.5744	1141.7014	1141.7070	-0.0057	1	35	0.83	1	KQTALVELLK
☒	6735	718.8274	2871.2805	2871.3023	-0.0218	1	35	0.3	1	CCTKPESERMPCTEDYLSLILNR
☒	5108	467.7610	1867.0149	1866.9873	0.0276	1	35	0.57	1	VCLLHEKTPVSEQVTK
☒	2800	501.2949	1000.5752	1000.5818	-0.0065	1	35	1	1	ALKAWSVAR
☒	5979	625.2997	2497.1697	2497.1835	-0.0138	1	35	0.42	1	AFDEKLFTFHADICTLPDTEK
☒	3770	473.9006	1418.6800	1418.6864	-0.0064	0	35	0.81	1	SLHTLFGDELCK
☒	4314	385.9597	1539.8097	1538.8127	0.9970	1	35	0.74	1	LCVLHEKTPVSEK
☒	2693	464.2480	926.4814	926.4861	-0.0047	0	35	0.96	1	YIYEIAR
☒	4333	773.9463	1545.8780	1545.8878	-0.0098	1	35	0.78	1	LKHLVDEPQNLIK
☒	2703	464.2483	926.4820	926.4861	-0.0041	0	35	0.98	1	YIYEIAR
☒	3810	474.2350	1419.6832	1418.6864	0.9968	0	35	0.83	1	SLHTLFGDELCK
☒	5191	630.6454	1888.9144	1887.9195	0.9948	0	35	0.68	1	HPYFYAPELLYYANK
☒	4192	376.9841	1503.9073	1503.9137	-0.0064	1	35	0.81	1	QTALVELLKHKPK
☒	3768	473.9005	1418.6797	1418.6864	-0.0067	0	35	0.92	1	SLHTLFGDELCK
☒	3772	473.9006	1418.6800	1418.6864	-0.0064	0	34	0.95	1	SLHTLFGDELCK
☒	4337	773.9488	1545.8830	1545.8878	-0.0048	1	34	0.9	1	LKHLVDEPQNLIK
☒	3783	473.9009	1418.6809	1418.6864	-0.0055	0	34	0.97	1	SLHTLFGDELCK
☒	2694	464.2480	926.4814	926.4861	-0.0047	0	34	1.2	1	YIYEIAR
☒	2707	464.2484	926.4822	926.4861	-0.0039	0	34	1.2	1	YIYEIAR
☒	2613	435.7721	869.5296	869.4971	0.0326	0	34	1.1	1	VVEALSPR
☒	3476	435.9084	1304.7034	1304.7088	-0.0055	0	34	1.1	1	HLVDEPQNLIK
☒	2747	480.5956	959.1766	959.4535	-0.2768	0	34	0.98	1	TIWEHMK + Oxidation (M)
☒	3112	381.5744	1141.7014	1141.7070	-0.0057	1	34	1.2	1	KQTALVELLK
☒	3021	532.7264	1063.4382	1062.5128	0.9254	0	34	1.2	1	TDVCSLVNR
☒	3119	381.5746	1141.7020	1141.7070	-0.0051	1	34	1.2	1	KQTALVELLK
☒	2589	423.9659	845.9172	845.4243	0.4930	0	34	0.99	1	QEVEVSR
☒	2567	409.2365	816.4584	816.4453	0.0131	1	34	1.6	1	KAADSVAR
☒	4863	863.4199	1724.8252	1723.8273	0.9979	0	34	0.89	1	MPCTEDYLSLILNR
☒	3788	473.9011	1418.6815	1418.6864	-0.0049	0	34	1.1	1	SLHTLFGDELCK
☒	2715	464.2485	926.4824	926.4861	-0.0037	0	34	1.3	1	YIYEIAR
☒	5810	800.3422	2398.0048	2398.0113	-0.0065	1	34	0.55	1	GGGFGGGSSFGGGSGFSGGGFGGGGFGGGR
☒	3299	625.3110	1248.6074	1248.6139	-0.0064	1	34	1.3	1	FKDLGEEHFK
☒	5458	682.6786	2045.0140	2044.0206	0.9933	1	34	0.79	1	RHPYFYAPELLYYANK
☒	3253	603.3079	1204.6012	1203.6347	0.9666	0	33	1.4	1	SVQTLEADLTK
☒	2698	464.2481	926.4816	926.4861	-0.0045	0	33	1.4	1	YIYEIAR
☒	2712	464.2484	926.4822	926.4861	-0.0039	0	33	1.4	1	YIYEIAR
☒	3172	582.3162	1162.6178	1162.6234	-0.0055	0	33	1.4	1	LVNELTEFAK
☒	2719	927.4919	926.4846	926.4861	-0.0015	0	33	1.4	1	YIYEIAR
☒	6736	958.4304	2872.2694	2871.3023	0.9670	1	33	0.49	1	CCTKPESERMPCTEDYLSLILNR
☒	2713	927.4896	926.4823	926.4861	-0.0038	0	33	1.4	1	YIYEIAR
☒	2689	464.2477	926.4808	926.4861	-0.0053	0	33	1.5	1	YIYEIAR
☒	2695	464.2480	926.4814	926.4861	-0.0047	0	33	1.5	1	YIYEIAR
☒	4284	513.5930	1537.7572	1538.8127	-1.0555	1	33	1.1	1	LCVLHEKTPVSEK
☒	3235	398.2283	1191.6631	1190.6481	1.0149	1	33	1.4	1	MIWEVTKLR + Oxidation (M)
☒	3303	625.3112	1248.6078	1248.6139	-0.0060	1	33	1.4	1	FKDLGEEHFK
☒	6682	1429.1406	2856.2666	2857.2867	-1.0200	1	33	0.52	1	CCAKPESERMPCTEDYLSLILNR + Oxidation (M)
☒	2711	464.2484	926.4822	926.4861	-0.0039	0	33	1.5	1	YIYEIAR
☒	2529	396.8004	791.5862	791.4099	0.1764	0	33	0.94	1	LMLLSES
☒	6723	717.8613	2867.4161	2868.4116	-0.9955	1	33	0.59	1	VDPMAIVVFHQADIGEYVRHEETLT
☒	5120	468.0114	1868.0165	1866.9873	1.0292	1	33	1	1	VCLLHEKTPVSEQVTK
☒	4707	556.9413	1667.8021	1666.8315	0.9706	0	33	1.2	1	IQFSDPAPPSQLDPR
☒	2700	464.2482	926.4818	926.4861	-0.0043	0	33	1.6	1	YIYEIAR
☒	7579	931.7205	3722.8529	3721.7988	1.0541	1	33	0.35	1	FNDLGEEHFRGLVLVAFSQYLQQCPFEDHVK
☒	3174	582.3162	1162.6178	1162.6234	-0.0055	0	33	1.6	1	LVNELTEFAK
☒	3782	473.9008	1418.6806	1418.6864	-0.0058	0	33	1.4	1	SLHTLFGDELCK
☒	2696	464.2480	926.4814	926.4861	-0.0047	0	33	1.7	1	YIYEIAR
☒	3469	653.3586	1304.7026	1304.7088	-0.0062	0	33	1.5	1	HLVDEPQNLIK
☒	2701	927.4893	926.4820	926.4861	-0.0041	0	33	1.7	1	YIYEIAR
☒	5385	505.9952	2019.9517	2018.9619	0.9898	1	33	1	1	LKPDPNTLCDEFKADEK
☒	3118	381.5746	1141.7020	1141.6343	0.0677	0	33	1.6	1	NGGAEIVELLK
☒	3814	474.2359	1419.6859	1418.6864	0.9995	0	33	1.4	1	SLHTLFGDELCK
☒	2704	464.2483	926.4820	926.4861	-0.0041	0	33	1.7	1	YIYEIAR
☒	3120	381.5746	1141.7020	1141.7070	-0.0051	1	32	1.7	1	KQTALVELLK
☒	2415	715.3229	714.3156	714.4388	-0.1232	0	32	0.53	1	SIIIGGR

☒	2699	927.4891	926.4818	926.4861	-0.0043	0	32	1.8	1	YIYEIAR
☒	2716	927.4901	926.4828	926.4861	-0.0033	0	32	1.8	1	YIYEIAR
☒	3764	473.9004	1418.6794	1418.6864	-0.0070	0	32	1.6	1	SLHTLFGDELCK
☒	3078	559.2842	1116.5538	1116.5272	0.0267	1	32	1.9	1	EGNGAAGERTR
☒	6066	844.0704	2529.1894	2528.2118	0.9776	1	32	0.86	1	QNCDQFEKLGEYGFQNALIVR
☒	3144	381.9092	1142.7058	1141.7070	0.9987	1	32	1.9	1	KQTALVELLK
☒	2702	464.2483	926.4820	926.4861	-0.0041	0	32	2	1	YIYEIAR
☒	3470	435.9082	1304.7028	1304.7088	-0.0061	0	32	1.8	1	HLVDEPQNLIK
☒	4293	385.7084	1538.8045	1538.8127	-0.0082	1	32	1.5	1	LCVLHEKTPVSEK
☒	2612	435.7721	869.5296	869.4971	0.0326	0	32	1.9	1	VVEALSPR
☒	6065	1265.6007	2529.1868	2528.2118	0.9751	1	32	0.91	1	QNCDQFEKLGEYGFQNALIVR
☒	4334	516.3008	1545.8806	1545.8878	-0.0073	1	32	1.7	1	LKHLVDEPQNLIK
☒	2690	464.2477	926.4808	926.4861	-0.0053	0	32	2.1	1	YIYEIAR
☒	3307	625.3113	1248.6080	1248.6139	-0.0058	1	32	2	1	FKDLGEEHFK
☒	7576	931.7200	3722.8509	3721.7988	1.0521	1	32	0.47	1	FNDLGEEHFRGLVLVAFSQYLQQCPFEDHVK
☒	4312	385.9595	1539.8089	1538.8127	0.9962	1	32	1.7	1	LCVLHEKTPVSEK
☒	2706	464.2484	926.4822	926.4861	-0.0039	0	31	2.2	1	YIYEIAR
☒	6734	958.0986	2871.2740	2871.3023	-0.0284	1	31	0.74	1	CCTKPESERMPCTEDYLSLILNR
☒	3767	473.9005	1418.6797	1418.6864	-0.0067	0	31	1.9	1	SLHTLFGDELCK
☒	3832	1426.7517	1425.7444	1424.7452	0.9992	0	31	1.9	1	AIQAAFFYLEPR
☒	4174	748.8718	1495.7290	1495.8254	-0.0964	1	31	1.8	1	AMAKLLSIIMFSR + Oxidation (M)
☒	2875	501.7933	1001.5720	1000.5454	1.0267	1	31	2.7	1	ALKWEQAR
☒	2714	927.4897	926.4824	926.4861	-0.0037	0	31	2.4	1	YIYEIAR
☒	4173	748.8711	1495.7276	1495.8254	-0.0978	1	31	1.9	1	AMAKLLSIIMFSR + Oxidation (M)
☒	2697	464.2480	926.4814	926.4861	-0.0047	0	31	2.5	1	YIYEIAR
☒	3047	542.2992	1082.5838	1082.5872	-0.0034	1	31	2.2	1	YIYEIARR
☒	5448	682.3450	2044.0132	2044.0206	-0.0075	1	31	1.5	1	RHPYFYAPELLYYANK
☒	2805	501.7921	1001.5696	1000.5665	1.0031	0	31	2.9	1	ALQSVQVTR
☒	2827	501.7929	1001.5712	1000.5665	1.0047	0	31	2.9	1	ALQSVQVTR
☒	2705	464.2483	926.4820	926.4861	-0.0041	0	31	2.5	1	YIYEIAR
☒	2718	464.2490	926.4834	926.4861	-0.0027	0	31	2.6	1	YIYEIAR
☒	3015	529.2241	1056.4336	1056.5564	-0.1227	0	31	2.6	1	VAQQLIDDR
☒	5102	374.4101	1867.0141	1866.9873	0.0268	1	31	1.6	1	VCLLHEKTPVSEQVTK
☒	2590	424.2164	846.4182	847.4651	-1.0468	0	31	3.1	1	SSSVEVIK
☒	2793	500.3779	998.7412	998.4417	0.2995	0	31	2.1	1	SNHEEVER
☒	2879	501.7934	1001.5722	1000.5665	1.0057	0	31	3	1	ALQSVQVTR
☒	5623	734.0315	2199.0727	2198.0929	0.9798	1	31	1.4	1	ATEEQLKTVMENFVAFVDK
☒	2891	501.7935	1001.5724	1000.5665	1.0059	0	31	3	1	ALQSVQVTR
☒	2846	501.7930	1001.5714	1000.5665	1.0049	0	31	3	1	ALQSVQVTR
☒	3130	381.5748	1141.7026	1141.7070	-0.0045	1	31	2.5	1	KQTALVELLK
☒	2854	501.7931	1001.5716	1000.5665	1.0051	0	31	3.1	1	ALQSVQVTR
☒	3083	561.2367	1120.4588	1120.5659	-0.1070	1	31	2.7	1	MRTEQITAR + Oxidation (M)
☒	2691	464.2477	926.4808	926.4861	-0.0053	0	31	2.7	1	YIYEIAR
☒	3309	417.2100	1248.6082	1248.6139	-0.0057	1	30	2.6	1	FKDLGEEHFK
☒	5159	941.4580	1880.9014	1879.9138	0.9876	0	30	1.8	1	RPCFSALTPDETYVPK
☒	3317	417.2102	1248.6088	1248.6139	-0.0051	1	30	2.7	1	FKDLGEEHFK
☒	5996	625.5501	2498.1713	2497.1835	0.9878	1	30	1.3	1	AFDEKLFTFHADICTLPDTEK
☒	5462	512.2610	2045.0149	2044.0206	0.9943	1	30	1.6	1	RHPYFYAPELLYYANK
☒	2692	464.2477	926.4808	926.4861	-0.0053	0	30	2.9	1	YIYEIAR
☒	5457	512.2607	2045.0137	2044.0206	0.9931	1	30	1.7	1	RHPYFYAPELLYYANK
☒	3043	361.8683	1082.5831	1082.5872	-0.0042	1	30	2.6	1	YIYEIARR
☒	6979	751.1031	3000.3833	2999.3940	0.9893	1	30	0.93	1	CCTESLVNRRPCFSALTPDETYVPK
☒	3143	1143.7104	1142.7031	1141.7070	0.9961	1	30	2.9	1	KQTALVELLK
☒	3042	361.8683	1082.5831	1082.5872	-0.0042	1	30	2.7	1	YIYEIARR
☒	3041	361.8683	1082.5831	1082.5872	-0.0042	1	30	2.7	1	YIYEIARR
☒	3046	542.2992	1082.5838	1082.5872	-0.0034	1	30	2.7	1	YIYEIARR
☒	3805	474.2348	1419.6826	1418.6864	0.9962	0	30	2.5	1	SLHTLFGDELCK
☒	5632	734.3708	2200.0906	2200.0722	0.0184	1	30	1.6	1	ATDEQLKTVMENFVAFVDK + Oxidation (M)
☒	5459	512.2609	2045.0145	2044.0206	0.9939	1	30	1.7	1	RHPYFYAPELLYYANK
☒	3771	473.9006	1418.6800	1418.6864	-0.0064	0	30	2.6	1	SLHTLFGDELCK
☒	3302	417.2098	1248.6076	1248.6139	-0.0063	1	30	2.8	1	FKDLGEEHFK
☒	5705	570.2928	2277.1421	2276.1511	0.9910	1	30	1.5	1	LFTFHADICTLPDTEKQIK
☒	3776	473.9007	1418.6803	1418.6864	-0.0061	0	30	2.6	1	SLHTLFGDELCK
☒	2571	825.4059	824.3986	823.5392	0.8594	0	30	1.8	1	RPALLVR
☒	2783	997.5858	996.5785	996.5240	0.0545	0	30	2.7	1	DPSTTVHIK
☒	3701	700.8416	1399.6686	1398.6853	0.9833	0	30	2.8	1	TVMENFVAFVDK
☒	2709	464.2484	926.4822	926.4861	-0.0039	0	30	3.1	1	YIYEIAR
☒	5455	512.2607	2045.0137	2044.0206	0.9931	1	30	1.8	1	RHPYFYAPELLYYANK
☒	5187	630.3106	1887.9100	1887.9195	-0.0096	0	30	2.1	1	HPYFYAPELLYYANK
☒	3763	473.9002	1418.6788	1418.6864	-0.0076	0	30	2.8	1	SLHTLFGDELCK
☒	3306	625.3113	1248.6080	1248.6139	-0.0058	1	30	3	1	FKDLGEEHFK
☒	5161	627.9764	1880.9074	1879.9138	0.9935	0	30	2.1	1	RPCFSALTPDETYVPK
☒	4313	385.9596	1539.8093	1538.8127	0.9966	1	30	2.5	1	LCVLHEKTPVSEK
☒	5467	682.6794	2045.0164	2044.0206	0.9957	1	30	1.9	1	RHPYFYAPELLYYANK
☒	2786	499.2976	996.5806	997.6284	-1.0477	1	30	2.9	1	GARLVEILK
☒	2984	1041.2002	1040.1929	1039.5451	0.6479	1	30	2	1	RSGDIAFFK
☒	3467	435.9081	1304.7025	1304.7088	-0.0064	0	30	3	1	HLVDEPQNLIK
☒	4163	498.5791	1492.7155	1491.7538	0.9617	1	30	2.7	1	ISTVRAPMLEGMR + 2 Oxidation (M)
☒	2528	396.7999	791.5852	791.4099	0.1754	0	30	2.1	1	LMLLSES
☒	2721	464.7502	927.4858	926.4861	0.9997	0	29	3.8	1	YIYEIAR
☒	6220	868.7657	2603.2753	2603.2910	-0.0157	1	29	1.5	1	MPCTEDYLSLILNRLCVLHEK
☒	2788	499.2981	996.5816	997.6284	-1.0467	1	29	3.1	1	GARLVEILK
☒	4157	498.2454	1491.7144	1491.7538	-0.0394	1	29	2.8	1	ISTVRAPMLEGMR + 2 Oxidation (M)
☒	2789	499.2982	996.5818	997.6284	-1.0465	1	29	3.1	1	GARLVEILK
☒	5122	374.6106	1868.0166	1866.9873	1.0293	1	29	2.3	1	VCLLHEKTPVSEQVTK
☒	2787	997.5889	996.5816	996.5240	0.0576	0	29	3.1	1	DPSTTVHIK
☒	6733	958.0968	2871.2686	2871.3023	-0.0338	1	29	1.2	1	CCTKPESERMPCTEDYLSLILNR
☒	4162	498.5786	1492.7140	1491.7538	0.9602	1	29	2.9	1	ISTVRAPMLEGMR + 2 Oxidation (M)
☒	6482	896.2914	2685.8524	2686.3094	-0.4571	0	29	1	1	AMFAISGEILITADHGNAEQMINPK + Oxidation (M)
☒	3811	474.2351	1419.6835	1418.6864	0.9971	0	29	3.1	1	SLHTLFGDELCK
☒	3184	582.3168	1162.6190	1162.6234	-0.0043	0	29	3.7	1	LVNELTEFAK
☒	3334	417.2112	1248.6118	1248.6139	-0.0021	1	29	3.6	1	FKDLGEEHFK
☒	5463	682.6790	2045.0152	2044.0206	0.9945	1	29	2.3	1	RHPYFYAPELLYYANK
☒	3556	673.6513	1345.2880	1344.7289	0.5591	0	29	2.4	1	DIVNDTLFPALK
☒	6719	717.8604	2867.4125	2868.4116	-0.9991	1	29	1.5	1	VDPMAIVVFHQADIGEYVRHEETLT
☒	4272	384.1816	1532.6973	1533.8376	-1.1403	1	29	3.2	1	NEPSGSLHGLLRVR
☒	3088	561.7383	1121.4620	1121.5968	-0.1348	0	29	3.8	1	ELYESILQK
☒	3199	582.8079	1163.6012	1162.6234	0.9779	0	29	3.9	1	LVNELTEFAK
☒	5674	562.9891	2247.9273	2246.9354	0.9919	1	29	1.8	1	ECCHGDLLECADDRADLAK
☒	2556	404.2250	806.4354	806.3956	0.0398	0	29	3	1	VELSGMR + Oxidation (M)
	3355	631.3603	1260.7060	1261.6700	-0.9639	1	29	3.9	1	KCANLVSELTK

3323	417.2103	1248.6091	1248.6139	-0.0048	1	29	3.9	1	FKDLGEEHFK
4363	778.3322	1554.6498	1553.6457	1.0042	0	29	3.1	1	DDPHACYSTVFDK
7577	745.5777	3722.8521	3721.7988	1.0534	1	29	0.91	1	FNDLGEEHFRGLVLVAFSQYLQQCPFEDHVK
4558	540.8870	1619.6392	1620.7644	-1.1253	1	29	2.8	1	QPKAGQHTGYEYSR
2708	464.2484	926.4822	926.4861	-0.0039	0	29	4.3	1	YIYEIAR
4242	381.2042	1520.7877	1519.7896	0.9981	0	29	3.4	1	AHNGVASTWLSHLK
3321	417.2102	1248.6088	1248.6139	-0.0051	1	29	4.1	1	FKDLGEEHFK
6786	579.6486	2893.2066	2892.5021	0.7045	1	29	1.3	1	EFVEGWTLAQTLGEGAYGEVKLLINR
7121	799.6124	3194.4205	3193.4784	0.9421	1	28	1.2	1	TVLGNFAAFVQKCCAAPDHEACFAVEGPK
2301	643.1212	642.1139	642.3449	-0.2310	0	28	0.092	1	AGGSVPR
5466	682.6794	2045.0164	2044.0206	0.9957	1	28	2.6	1	RHPYFYAPELLYYANK
4302	385.7087	1538.8057	1538.8127	-0.0070	1	28	3.4	1	LCVLHEKTPVSEK
5372	505.7452	2018.9517	2018.9619	-0.0102	1	28	2.8	1	LKPDPNTLCDEFKADEK
2710	464.2484	926.4822	926.4861	-0.0039	0	28	4.6	1	YIYEIAR
4853	862.9221	1723.8296	1722.8365	0.9931	1	28	3.2	1	DAFLGSFLYEYSRR
5456	512.2607	2045.0137	2044.0206	0.9931	1	28	2.7	1	RHPYFYAPELLYYANK
5444	682.3422	2044.0048	2044.0206	-0.0159	1	28	2.7	1	RHPYFYAPELLYYANK
3132	571.8587	1141.7028	1141.7070	-0.0042	1	28	4.4	1	KQTALVELLK
4972	449.9615	1795.8169	1794.8247	0.9922	1	28	3.2	1	DDPHACYSTVFDKLK
4336	516.3010	1545.8812	1545.8878	-0.0067	1	28	3.8	1	LKHLVDEPQNLIK
3313	417.2101	1248.6085	1248.6139	-0.0054	1	28	4.5	1	FKDLGEEHFK
3480	435.9089	1304.7049	1304.7088	-0.0040	0	28	4.3	1	HLVDEPQNLIK
4184	752.0222	1502.0298	1502.7630	-0.7332	0	28	2.9	1	HGEYGFQNALIVR
3294	417.2097	1248.6073	1248.6139	-0.0066	1	28	4.6	1	FKDLGEEHFK
5149	627.6421	1879.9045	1879.9138	-0.0094	0	28	3.2	1	RPCFSALTPDETYVPK
5024	605.6122	1813.8148	1813.8226	-0.0078	1	28	3.2	1	LAKEYEATLEECCAK
3319	417.2102	1248.6088	1248.6139	-0.0051	1	28	4.7	1	FKDLGEEHFK
3336	417.5444	1249.6114	1248.6139	0.9975	1	28	4.6	1	FKDLGEEHFK
2566	409.0077	816.0008	816.4857	-0.4849	1	28	4.1	1	KFLANPK
2790	499.2983	996.5820	997.6284	-1.0463	1	28	4.4	1	GARLVEILK
3325	417.2103	1248.6091	1248.6139	-0.0048	1	28	4.8	1	FKDLGEEHFK
3044	361.8683	1082.5831	1082.5872	-0.0042	1	28	4.7	1	YIYEIARR
4073	738.3802	1474.7458	1474.7337	0.0122	1	28	4.3	1	LKEEEMQAQIEK
2717	464.2488	926.4830	926.4861	-0.0031	0	28	5.2	1	YIYEIAR
3300	417.2098	1248.6076	1248.6139	-0.0063	1	28	4.9	1	FKDLGEEHFK
5331	398.6151	1988.0391	1987.0044	1.0347	1	28	3.3	1	VDASMKLIDQVPESNSVR
5449	512.0107	2044.0137	2044.0206	-0.0069	1	28	3.2	1	RHPYFYAPELLYYANK
5454	682.6783	2045.0131	2044.0206	0.9924	1	28	3.1	1	RHPYFYAPELLYYANK
6822	584.8715	2919.3211	2920.3993	-1.0782	1	28	1.8	1	LRLMYEANPMAMLVEQAGGAATNGEQR
2720	464.7497	927.4848	926.4861	0.9987	0	28	5.9	1	YIYEIAR
3315	417.2101	1248.6085	1248.6139	-0.0054	1	28	5.1	1	FKDLGEEHFK
2557	404.2259	806.4372	806.3956	0.0416	0	28	4	1	VELSGMR + Oxidation (M)
3312	417.2101	1248.6085	1248.6139	-0.0054	1	28	5.2	1	FKDLGEEHFK
2853	501.7931	1001.5716	1000.5665	1.0051	0	27	6.3	1	ALQSVQVTR
2752	966.0995	965.0922	964.6182	0.4741	1	27	3.6	1	HLSLLVRK
3134	381.5750	1141.7032	1141.6343	0.0689	0	27	5.2	1	NGGAEIVELLK
3316	417.2101	1248.6085	1248.6139	-0.0054	1	27	5.2	1	FKDLGEEHFK
4161	498.5780	1492.7122	1491.7538	0.9584	1	27	4.4	1	ISTVRAPMLEGMR + 2 Oxidation (M)
3314	417.2101	1248.6085	1248.6139	-0.0054	1	27	5.3	1	FKDLGEEHFK
3045	542.2990	1082.5834	1082.5872	-0.0038	1	27	5.1	1	YIYEIARR
6717	717.8604	2867.4125	2868.4116	-0.9991	1	27	2.2	1	VDPMAIVVFHQADIGEYVRHEETLT
2867	501.7932	1001.5718	1000.5665	1.0053	0	27	6.5	1	ALQSVQVTR
3296	417.2097	1248.6073	1248.6139	-0.0066	1	27	5.3	1	FKDLGEEHFK
2807	501.7922	1001.5698	1000.5665	1.0033	0	27	6.6	1	ALQSVQVTR
3293	417.2097	1248.6073	1248.6139	-0.0066	1	27	5.4	1	FKDLGEEHFK
2857	501.7931	1001.5716	1000.5665	1.0051	0	27	6.6	1	ALQSVQVTR
2583	423.2401	844.4656	845.5044	-1.0388	1	27	7.4	1	MVKDIIK
6191	646.7845	2583.1089	2582.3850	0.7239	1	27	2.2	1	NELLLAMAGSLVRNTVQLIEENR
4230	506.5828	1516.7266	1517.8202	-1.0936	1	27	4.8	1	SFDAEKQLAIAAVR
2409	713.3130	712.3057	712.3868	-0.0810	0	27	0.88	1	LAAADPR
6688	717.0693	2864.2481	2863.2575	0.9906	1	27	2.1	1	TCVADESHAGCEKSLHTLFGDELCK
4686	413.9552	1651.7917	1650.8837	0.9080	1	27	4.3	1	MYLKEMGTVPLLTR
4152	498.2434	1491.7084	1491.7538	-0.0454	1	27	4.8	1	ISTVRAPMLEGMR + 2 Oxidation (M)
3097	566.4250	1130.8354	1130.6560	0.1794	1	27	4.5	1	HALLIHGDKK
3291	417.2093	1248.6061	1248.6139	-0.0078	1	27	5.7	1	FKDLGEEHFK
2791	997.5903	996.5830	996.5240	0.0590	0	27	5.3	1	DPSTTVHIK
3561	675.1689	1348.3232	1348.8078	-0.4846	1	27	3.6	1	KEVAPAANLVIPK
6527	547.4407	2732.1671	2731.3851	0.7821	1	27	2.1	1	AFLETVQAIAQALGVSDATPEEGKMR
6982	751.1053	3000.3921	2999.3940	0.9981	1	27	2	1	CCTESLVNRRPCFSALTPDETYVPK
4151	498.2433	1491.7081	1491.7538	-0.0457	1	27	5.1	1	ISTVRAPMLEGMR + 2 Oxidation (M)
6689	717.0695	2864.2489	2863.2575	0.9914	1	27	2.2	1	TCVADESHAGCEKSLHTLFGDELCK
6188	517.4304	2582.1156	2582.3203	-0.2047	0	27	2.5	1	LANSCFDFLGYLSSVPQSPLLVR
2956	510.3058	1018.5970	1019.4706	-0.8735	0	27	7.4	1	ALEGQEMAR + Oxidation (M)
6637	565.0187	2820.0571	2819.3106	0.7466	1	27	1.8	1	EFKAGAANETVEAGACPADAMILDAGPK + Oxidation (M)
3038	361.8678	1082.5816	1082.5872	-0.0057	1	27	6.1	1	YIYEIARR
2546	401.2605	800.5064	799.4010	1.1054	0	27	7.1	1	LGNPMPR + Oxidation (M)
3085	561.2372	1120.4598	1120.6175	-0.1577	1	27	6.8	1	FVRMSLGGVR
5469	682.6797	2045.0173	2044.0206	0.9966	1	26	4	1	RHPYFYAPELLYYANK
5064	615.9568	1844.8486	1844.0156	0.8330	1	26	4.6	1	VDGDQILYIIAREALR
2768	490.7041	979.3936	980.4749	-1.0813	0	26	6.6	1	MPSAFSVSR
3216	392.1310	1173.3712	1173.5850	-0.2139	1	26	5.3	1	RNQQADLSSR
2872	501.7933	1001.5720	1000.5665	1.0055	0	26	8.2	1	ALQSVQVTR
3308	417.2100	1248.6082	1248.6139	-0.0057	1	26	6.8	1	FKDLGEEHFK
2881	501.7934	1001.5722	1000.5665	1.0057	0	26	8.3	1	ALQSVQVTR
5103	374.4101	1867.0141	1866.9873	0.0268	1	26	4.6	1	VCLLHEKTPVSEQVTK
3288	624.0486	1246.0826	1245.6816	0.4011	1	26	4.6	1	LSIKEVTAEEK
5453	682.6783	2045.0131	2044.0206	0.9924	1	26	4.2	1	RHPYFYAPELLYYANK
2522	394.5828	787.1510	787.4188	-0.2677	1	26	4.9	1	DRADLAK
2542	800.4476	799.4403	800.5119	-1.0716	1	26	6.5	1	AIKASIAK
3290	417.2090	1248.6052	1248.6139	-0.0087	1	26	7.1	1	FKDLGEEHFK
5160	627.9750	1880.9032	1879.9138	0.9893	0	26	4.9	1	RPCFSALTPDETYVPK
3048	361.8686	1082.5840	1082.5872	-0.0033	1	26	7	1	YIYEIARR
3301	417.2098	1248.6076	1248.6139	-0.0063	1	26	7.3	1	FKDLGEEHFK
3087	1121.4686	1120.4613	1119.5594	0.9019	0	26	7.6	1	VLSSMSLDPR + Oxidation (M)
6530	547.4415	2732.1711	2731.3851	0.7861	1	26	2.6	1	AFLETVQAIAQALGVSDATPEEGKMR
6059	632.2806	2525.0933	2524.3981	0.6952	1	26	3	1	MTRPIQASLDLQVMKQNLAIVR
3324	417.2103	1248.6091	1248.6139	-0.0048	1	26	7.4	1	FKDLGEEHFK
3328	417.2104	1248.6094	1248.6139	-0.0045	1	26	7.4	1	FKDLGEEHFK
4294	385.7085	1538.8049	1538.8127	-0.0078	1	26	6	1	LCVLHEKTPVSEK

2866	501.7932	1001.5718	1001.5141	0.0577	0	26	9.2	1	AIQASVEER
2823	501.7929	1001.5712	1001.5141	0.0571	0	26	9.2	1	AIQASVEER
3326	417.2103	1248.6091	1248.6139	-0.0048	1	26	7.7	1	FKDLGEEHFK
3329	417.2104	1248.6094	1248.6139	-0.0045	1	26	7.7	1	FKDLGEEHFK
3053	543.8088	1085.6030	1085.6080	-0.0050	1	26	8.5	1	NDAALDVLKK
2649	449.2180	896.4214	897.5470	-1.1255	0	26	7	1	IMPAIVVR
3056	546.8375	1091.6604	1091.5645	0.0959	0	26	8.3	1	MQLTVLTDR + Oxidation (M)
3198	582.8073	1163.6000	1162.6234	0.9767	0	26	8.1	1	LVNELTEFAK
5100	374.4099	1867.0131	1866.9873	0.0258	1	26	5.3	1	VCLLHEKTPVSEQVTK
7527	729.9208	3644.5676	3644.8860	-0.3184	1	26	1.6	1	IVGVTGTDGKTTTTFLTSVALEAGGAITGLMGTVDFK + Oxidation (M)
2963	512.7834	1023.5522	1023.5423	0.0099	0	25	8	1	IVVSFCTAK
3412	430.1927	1287.5563	1286.6329	0.9234	1	25	8.3	1	QFKDINTFMK + Oxidation (M)
2785	997.5871	996.5798	996.5240	0.0558	0	25	7.8	1	DPSTTVHIK
6532	547.4417	2732.1721	2731.3851	0.7871	1	25	3.1	1	AFLETVQAIAQALGVSDATPEEGKMR
5460	512.2609	2045.0145	2044.0206	0.9939	1	25	5.2	1	RHPYFYAPELLYYANK
3320	417.2102	1248.6088	1248.6139	-0.0051	1	25	8.5	1	FKDLGEEHFK
3333	417.2109	1248.6109	1248.6139	-0.0030	1	25	8.6	1	FKDLGEEHFK
3039	361.8681	1082.5825	1082.5872	-0.0048	1	25	8.4	1	YIYEIARR
3318	417.2102	1248.6088	1248.6139	-0.0051	1	25	8.7	1	FKDLGEEHFK
3732	705.4270	1408.8394	1408.7674	0.0720	0	25	7.4	1	DNPIDVQLLGIGR
4811	565.9451	1694.8135	1695.7556	-0.9421	0	25	6.6	1	EASGNMSQITEMLNR + Oxidation (M)
2957	510.3071	1018.5996	1019.4706	-0.8709	0	25	11	1	ALEGQEMAR + Oxidation (M)
5451	512.0112	2044.0157	2044.0206	-0.0049	1	25	5.5	1	RHPYFYAPELLYYANK
4688	414.2037	1652.7857	1653.9375	-1.1518	0	25	6.7	1	QIEMIIISPQLLEK
2755	487.7299	973.4452	973.4505	-0.0053	0	25	11	1	DLGEEHFK
3327	417.2104	1248.6094	1248.6139	-0.0045	1	25	8.9	1	FKDLGEEHFK
3498	658.2427	1314.4708	1314.6779	-0.2071	0	25	7.3	1	EELEVSIINNR
4369	779.5710	1557.1274	1556.9039	0.2236	1	25	5	1	RSIVVGVIGYPNVGK
4944	593.5853	1777.7341	1777.9686	-0.2346	0	25	6	1	ASLPDLQNGPASLIVGAR
2784	499.2966	996.5786	997.6284	-1.0497	1	25	8.4	1	GARLVEILK
5110	374.4103	1867.0151	1866.9873	0.0278	1	25	6.1	1	VCLLHEKTPVSEQVTK
4061	738.3790	1474.7434	1474.7337	0.0098	1	25	8.1	1	LKEEEMQAQIEK
5176	377.6088	1883.0076	1883.9880	-0.9804	0	25	6.1	1	APEPLPSPEESPLPVPTK
5887	494.0313	2465.1201	2466.2808	-1.1607	1	25	4.3	1	HPTPLHRDFLTTDFQYIPIR
4158	498.2549	1491.7429	1491.7357	0.0071	0	25	7.8	1	NIWNAYIEVENK
2914	505.2668	1008.5190	1008.4546	0.0645	1	25	8.9	1	TEEMEAKR + Oxidation (M)
3295	417.2097	1248.6073	1248.6139	-0.0066	1	25	9.2	1	FKDLGEEHFK
3298	417.2097	1248.6073	1248.6139	-0.0066	1	25	9.2	1	FKDLGEEHFK
6198	518.4296	2587.1116	2587.1465	-0.0349	1	25	3.8	1	LNVSQAMATMSTGECWEVSKNGR + 2 Oxidation (M)
3438	651.3689	1300.7232	1300.6485	0.0747	0	25	9	1	AALLSFFETMR + Oxidation (M)
6172	514.4370	2567.1486	2566.3069	0.8417	1	25	4.1	1	RYVMMIDQNIILNDNMAILVR + 2 Oxidation (M)
3297	417.2097	1248.6073	1248.6139	-0.0066	1	25	9.3	1	FKDLGEEHFK
3352	630.7975	1259.5804	1260.6272	-1.0467	0	25	9.6	1	GDGSEMVIIELV
2723	466.2748	930.5350	931.5087	-0.9736	0	25	12	1	ALQSVSATR
4458	526.5909	1576.7509	1576.7953	-0.0444	1	25	7.9	1	SVMVENIEKIMER
5121	374.6106	1868.0166	1866.9873	1.0293	1	25	6.4	1	VCLLHEKTPVSEQVTK
3338	417.5447	1249.6123	1248.6139	0.9984	1	25	9.3	1	FKDLGEEHFK
3688	697.1465	1392.2784	1392.7361	-0.4577	1	25	5.6	1	SSRALLSVEEFR
3330	417.2105	1248.6097	1248.6139	-0.0042	1	25	9.6	1	FKDLGEEHFK
6713	574.4896	2867.4116	2868.3820	-0.9704	1	25	3.9	1	LVGQAIETFNMIEDGDRLMVCLSGGK + Oxidation (M)
3214	587.5790	1173.1434	1172.6666	0.4769	1	25	7	1	VTFPDGRILR
4716	838.0018	1673.9890	1672.8883	1.1007	0	25	7.3	1	SDVQGSLEAITEALLK
4971	599.6129	1795.8169	1794.8247	0.9922	1	25	7	1	DDPHACYSTVFDKLK
5011	453.7564	1810.9965	1809.8688	1.1277	1	25	6.9	1	GMFVQLDPGAMMSVRR + Oxidation (M)
3013	352.8371	1055.4895	1056.5564	-1.0669	0	25	10	1	VAQAAGEVGQK
5354	669.2858	2004.8356	2003.8387	0.9969	1	25	5.7	1	VASLRETYGDMADCCEK
3337	417.5445	1249.6117	1248.6139	0.9978	1	25	9.7	1	FKDLGEEHFK
4144	497.9203	1490.7391	1491.7357	-0.9967	0	25	8.5	1	NIWNAYIEVENK
6531	547.4417	2732.1721	2731.3851	0.7871	1	25	3.6	1	AFLETVQAIAQALGVSDATPEEGKMR
2744	477.2570	952.4994	951.5099	0.9895	0	25	9.2	1	MGLYEVLK
5325	662.3591	1984.0555	1983.9910	0.0645	1	25	6.5	1	AMAILADVFYGAPSQKMR + Oxidation (M)
4577	543.2539	1626.7399	1626.9192	-0.1793	1	25	8.1	1	VTTSLGDNIKLEIPK
2743	477.2562	952.4978	951.5099	0.9879	0	25	9.3	1	MGLYEVLK
3058	547.3387	1092.6628	1091.5645	1.0983	0	25	10	1	MQLTVLTDR + Oxidation (M)
6783	723.3336	2889.3053	2890.4110	-1.1057	1	25	3.7	1	AGCILCGYLKLTPMFLMVMPGMISR + 2 Oxidation (M)
6974	751.1026	3000.3813	3001.3732	-0.9919	1	25	3.5	1	CCSESLVNRRPCFSGLEVDETYVPK
4210	378.7424	1510.9405	1510.8970	0.0435	1	24	7.5	1	VNVDELLKVELLK
7117	1065.4785	3193.4137	3193.4784	-0.0647	1	24	3	1	TVLGNFAAFVQKCCAAPDHEACFAVEGPK
2859	501.7932	1001.5718	1000.5553	1.0166	1	24	13	1	AIQKELGDK
3210	585.5594	1169.1042	1169.5941	-0.4899	1	24	7.1	1	FASFAGASRTR
4854	575.6177	1723.8313	1722.8365	0.9947	1	24	7.8	1	DAFLGSFLYEYSRR
2455	745.3483	744.3410	745.3719	-1.0308	0	24	6.8	1	ETGGLNR
6880	987.1950	2958.5632	2958.6517	-0.0886	1	24	4	1	ARVFIELPLSIAGMAIPQQLFFSGVVR
2629	443.7819	885.5492	885.5535	-0.0042	0	24	14	1	TAIIDILK
3562	675.5486	1349.0826	1349.5955	-0.5129	1	24	6.6	1	CMKLDDTAGPIN + Oxidation (M)
2471	378.7412	755.4678	756.5109	-1.0430	0	24	4.2	1	SILALLK
3991	723.6868	1445.3590	1445.8354	-0.4764	1	24	6.8	1	LYDISARLIINR
5877	819.3941	2455.1605	2456.1783	-1.0178	1	24	5.5	1	SSLVLAMLQQMLMEDKADMVR + 3 Oxidation (M)
4264	383.9491	1531.7673	1531.7738	-0.0065	1	24	9.6	1	LKECCDKPLLEK
2962	512.7827	1023.5508	1023.5574	-0.0065	1	24	11	1	LVDATHGRR
3383	641.8785	1281.7424	1280.5891	1.1533	1	24	10	1	ARMASANSSSQR + Oxidation (M)
2630	443.7822	885.5498	885.5535	-0.0036	0	24	14	1	TAIIDILK
4924	588.5968	1762.7686	1763.9128	-1.1442	1	24	8.4	1	VTVEMTPIYSIDPKR + Oxidation (M)
3213	585.8555	1169.6964	1170.6608	-0.9644	0	24	11	1	DSGLEVIIGLR
4473	529.5677	1585.6813	1584.8471	0.8341	1	24	9	1	TELELLQVDRVDR
2746	480.5954	959.1762	959.4535	-0.2772	0	24	9.9	1	TIWEHMK + Oxidation (M)
2748	480.5958	959.1770	959.4535	-0.2764	0	24	9.9	1	TIWEHMK + Oxidation (M)
3212	585.8549	1169.6952	1170.6608	-0.9656	0	24	12	1	DSGLEVIIGLR
4273	384.1999	1532.7705	1532.7003	0.0702	1	24	9.7	1	KEPAVPAMPAGGMDF + Oxidation (M)
3457	435.5921	1303.7545	1302.7468	1.0076	0	24	11	1	MSLLELVEILK + Oxidation (M)
2893	501.7936	1001.5726	1001.5141	0.0585	0	24	14	1	AIQASVEER
5876	819.3922	2455.1548	2456.1783	-1.0235	1	24	5.8	1	SSLVLAMLQQMLMEDKADMVR + 3 Oxidation (M)
3057	547.3152	1092.6158	1091.4917	1.1241	0	24	12	1	KPVDDEMSR + Oxidation (M)
2638	445.1184	888.2222	887.4865	0.7358	0	24	11	1	FAPDVALR
6584	698.9162	2791.6357	2792.5032	-0.8675	0	24	3.9	1	TANLSILLNDISNTLTINNSNIIHGK
5446	682.3435	2044.0087	2044.0206	-0.0120	1	24	7.6	1	RHPYFYAPELLYYANK
6247	522.8657	2609.2921	2609.3352	-0.0431	1	24	5.6	1	WFLKGYQNANYLSLFLMVATSK + Oxidation (M)
5150	627.6422	1879.9048	1879.9138	-0.0091	0	24	8.7	1	RPCFSALTPDETYVPK
4186	752.4416	1502.8686	1502.7630	0.1056	0	24	11	1	HGEYGFQNALIVR
2844	501.7930	1001.5714	1000.5665	1.0049	0	24	15	1	ALQSVQVTR
2895	501.7936	1001.5726	1000.5665	1.0061	0	24	15	1	ALQSVQVTR

☒	2833	501.7930	1001.5714	1000.5665	1.0049	0	24	16	1	ALQSVQVTR
☒	2841	501.7930	1001.5714	1001.5141	0.0573	0	24	16	1	AIQASVEER
☒	2813	501.7927	1001.5708	1000.5665	1.0043	0	24	16	1	ALQSVQVTR
☒	2582	844.0710	843.0637	842.4974	0.5663	0	24	11	1	SANAVILR
☒	2824	501.7929	1001.5712	1000.5665	1.0047	0	24	16	1	ALQSVQVTR
☒	2900	501.7938	1001.5730	1000.5454	1.0277	1	24	16	1	ALKWEQAR
☒	3086	1121.4680	1120.4607	1120.5149	-0.0542	0	24	14	1	LYEQSGGDPR
☒	2809	501.7924	1001.5702	1000.5665	1.0037	0	24	16	1	ALQSVQVTR
☒	2870	501.7933	1001.5720	1000.5454	1.0267	1	24	16	1	ALKWEQAR
☒	2887	501.7935	1001.5724	1000.5665	1.0059	0	24	16	1	ALQSVQVTR
☒	4912	438.4973	1749.9601	1748.8661	1.0940	1	24	9.6	1	YGDIYYKLLDDFPK
☒	2816	501.7928	1001.5710	1000.5665	1.0045	0	24	16	1	ALQSVQVTR
☒	2876	501.7933	1001.5720	1000.5665	1.0055	0	24	16	1	ALQSVQVTR
☒	2899	501.7937	1001.5728	1000.5665	1.0063	0	24	16	1	ALQSVQVTR
☒	2826	501.7929	1001.5712	1000.5665	1.0047	0	23	16	1	ALQSVQVTR
☒	2898	501.7937	1001.5728	1000.5665	1.0063	0	23	16	1	ALQSVQVTR
☒	2831	501.7929	1001.5712	1000.5665	1.0047	0	23	16	1	ALQSVQVTR
☒	2871	501.7933	1001.5720	1000.5665	1.0055	0	23	16	1	ALQSVQVTR
☒	2829	501.7929	1001.5712	1000.5665	1.0047	0	23	16	1	ALQSVQVTR
☒	5710	570.2932	2277.1437	2276.1511	0.9926	1	23	7.1	1	LFTFHADICTLPDTEKQIK
☒	6229	435.8884	2609.2867	2608.1819	1.1049	1	23	5.9	1	ASENAEVDIPQAMVDTELDRMMK + Oxidation (M)
☒	2839	501.7930	1001.5714	1000.5665	1.0049	0	23	16	1	ALQSVQVTR
☒	2869	501.7933	1001.5720	1000.5665	1.0055	0	23	16	1	ALQSVQVTR
☒	2855	501.7931	1001.5716	1000.5665	1.0051	0	23	16	1	ALQSVQVTR
☒	4188	752.5729	1503.1312	1502.7399	0.3913	0	23	7.8	1	SVALDCDGDALLVR
☒	6533	547.4419	2732.1731	2731.3851	0.7881	1	23	4.8	1	AFLETVQAIAQALGVSDATPEEGKMR
☒	2906	501.7940	1001.5734	1000.5665	1.0069	0	23	16	1	ALQSVQVTR
☒	2842	501.7930	1001.5714	1000.5665	1.0049	0	23	16	1	ALQSVQVTR
☒	2910	501.7943	1001.5740	1000.5665	1.0075	0	23	16	1	ALQSVQVTR
☒	2819	501.7928	1001.5710	1000.5665	1.0045	0	23	16	1	ALQSVQVTR
☒	2861	501.7932	1001.5718	1000.5665	1.0053	0	23	16	1	ALQSVQVTR
☒	6978	1501.1989	3000.3832	2999.3940	0.9893	1	23	4.5	1	CCTESLVNRRPCFSALTPDETYVPK
☒	4900	583.5818	1747.7236	1746.8505	0.8731	1	23	9.4	1	GGPGMREMLAATAALGGR + 2 Oxidation (M)
☒	2847	501.7930	1001.5714	1000.5665	1.0049	0	23	16	1	ALQSVQVTR
☒	2851	501.7931	1001.5716	1000.5665	1.0051	0	23	16	1	ALQSVQVTR
☒	2902	501.7938	1001.5730	1000.5665	1.0065	0	23	16	1	ALQSVQVTR
☒	6405	667.5848	2666.3101	2665.2150	1.0951	0	23	5.9	1	NHGNNEADAALQGLAQQGVDMEDLR
☒	2626	442.2628	882.5110	882.5174	-0.0064	0	23	12	1	LEVIPANK
☒	7097	791.3647	3161.4297	3160.5677	0.8620	1	23	4.2	1	VTVSDERDYAVSFVVAETDAAPAPAAAPVSR
☒	2860	501.7932	1001.5718	1000.5665	1.0053	0	23	16	1	ALQSVQVTR
☒	4056	492.2457	1473.7153	1473.8377	-0.1225	1	23	12	1	LQPLFKDMLITR
☒	4749	843.3900	1684.7654	1683.8753	0.8901	0	23	11	1	SVMPEGQLVEPILEK + Oxidation (M)
☒	7572	931.7173	3722.8401	3721.7988	1.0413	1	23	3.1	1	FNDLGEEHFRGLVLVAFSQYLQQCPFEDHVK
☒	2812	501.7927	1001.5708	1000.5665	1.0043	0	23	17	1	ALQSVQVTR
☒	2828	501.7929	1001.5712	1000.5665	1.0047	0	23	17	1	ALQSVQVTR
☒	6462	445.5598	2667.3151	2667.3465	-0.0314	1	23	6	1	MSSFVQELDKAFEGQLQVLLEEK
☒	2843	501.7930	1001.5714	1000.5665	1.0049	0	23	17	1	ALQSVQVTR
☒	5245	481.9272	1923.6797	1922.8826	0.7971	0	23	6.5	1	GDAQGCVSAGNTAALMGLSK + Oxidation (M)
☒	2374	690.5862	689.5789	689.3456	0.2333	0	23	1.6	1	SSGGLNR
☒	3623	685.0361	1368.0576	1367.7045	0.3532	0	23	8.3	1	QGEGPVLDPLTSR
☒	5099	467.7605	1867.0129	1866.9873	0.0256	1	23	9.3	1	VCLLHEKTPVSEQVTK
☒	4693	553.5346	1657.5820	1657.7262	-0.1443	0	23	8.1	1	DMTDYMLQGMGKPR + Oxidation (M)
☒	2834	501.7930	1001.5714	1001.5757	-0.0042	1	23	17	1	IAKSLDEVK
☒	2837	501.7930	1001.5714	1000.5665	1.0049	0	23	17	1	ALQSVQVTR
☒	4739	842.8882	1683.7618	1683.9083	-0.1465	1	23	11	1	LKFPEPQEVVEELK
☒	4894	582.9399	1745.7979	1745.9577	-0.1598	1	23	11	1	AVGKYHTFSAGLIIGGR
☒	4123	741.8984	1481.7822	1480.6538	1.1285	1	23	12	1	QCDPEKMITESK + Oxidation (M)
☒	4750	562.5958	1684.7656	1685.9603	-1.1948	1	23	11	1	ALWTKELETLLLEK
☒	2903	501.7939	1001.5732	1001.5757	-0.0024	1	23	18	1	IAKSLDEVK
☒	2838	501.7930	1001.5714	1001.5757	-0.0042	1	23	18	1	IAKSLDEVK
☒	2964	512.7835	1023.5524	1023.5423	0.0101	0	23	14	1	IVVSFCTAK
☒	3508	660.8342	1319.6538	1319.7198	-0.0659	0	23	14	1	GTITITGAGFLNR
☒	2588	423.9568	845.8990	845.4283	0.4707	0	23	12	1	YQAVPTAP
☒	2765	490.7040	979.3934	979.5563	-0.1629	1	23	15	1	SGKHIPVSR
☒	2730	470.4492	938.8838	938.4895	0.3943	0	23	8.9	1	EQMLPPPK
☒	4256	383.9488	1531.7661	1531.7738	-0.0077	1	23	12	1	LKECCDKPLLEK
☒	2892	501.7935	1001.5724	1002.5498	-0.9773	1	23	18	1	LAKWAAESK
☒	4124	741.8984	1481.7822	1480.6538	1.1285	1	23	12	1	QCDPEKMITESK + Oxidation (M)
☒	6595	562.4521	2807.2241	2806.3344	0.8897	1	23	5.4	1	EGDPVNEMLFIIRGHLDSYTTNGGR + Oxidation (M)
☒	3728	705.2375	1408.4604	1408.7674	-0.3070	0	23	9.5	1	DNPIDVQLLGIGR
☒	4756	564.2835	1689.8287	1688.7148	1.1139	1	23	12	1	KAINNGDGMWHCDR + Oxidation (M)
☒	2965	512.7836	1023.5526	1023.5423	0.0103	0	23	14	1	IVVSFCTAK
☒	5234	480.9893	1919.9281	1921.0012	-1.0731	1	23	10	1	MISMNNDTLAVIKQSIK + Oxidation (M)
☒	4154	498.2438	1491.7096	1490.7585	0.9511	1	23	13	1	EEMRLMDSVIIR
☒	5612	548.7339	2190.9065	2190.8335	0.0730	1	23	7.6	1	KDSGMPAMPGGMGGMGGMGGMDY
☒	3036	541.2729	1080.5312	1081.4896	-0.9583	1	23	14	1	SIKMDEGMR + Oxidation (M)
☒	3591	454.8933	1361.6581	1361.6761	-0.0181	0	23	14	1	HLSTILECAYR
☒	5123	374.6106	1868.0166	1866.9873	1.0293	1	23	10	1	VCLLHEKTPVSEQVTK
☒	6574	556.0472	2775.1996	2774.4129	0.7868	1	23	5.7	1	ITLNRDIDINMMDILTTPSMAKPR + Oxidation (M)
☒	3284	622.8306	1243.6466	1242.5880	1.0586	0	23	16	1	GGLEGIYYSER
☒	4288	770.2275	1538.4404	1538.8490	-0.4086	1	23	8.3	1	GVTPLMHKTAEVLK + Oxidation (M)
☒	5447	512.0098	2044.0101	2044.0206	-0.0105	1	23	9.8	1	RHPYFYAPELLYYANK
☒	6839	586.2491	2926.2091	2926.4721	-0.2630	1	23	4.9	1	SSSSMDYFLFRLFCSLALASLLVQR + Oxidation (M)
☒	6635	470.8927	2819.3125	2819.4892	-0.1766	0	23	6.3	1	SPALDAVVIGAGVTGIYQAFLINQAGMK + Oxidation (M)
☒	8661	1207.8195	4827.2489	4828.4160	-1.1671	1	23	1.5	1	LPMQATVISVIVAGDFVLSFLSLGGACSTASVAVLLMDAGEKQCDR + 2 Oxidation (M)
☒	2973	518.3152	1034.6158	1034.5760	0.0398	0	23	18	1	GYLDSLIVR
☒	3407	644.2849	1286.5552	1286.6329	-0.0777	1	23	16	1	QFKDINTFMK + Oxidation (M)
☒	6272	523.4366	2612.1466	2611.2986	0.8480	1	23	6.5	1	QGQGVVTIGAMAKGDSEFMASTVLAK + Oxidation (M)
☒	6981	1001.1354	3000.3844	2999.3940	0.9904	1	23	5.5	1	CCTESLVNRRPCFSALTPDETYVPK
☒	2722	466.2745	930.5344	931.5087	-0.9742	0	23	21	1	ALQSVSATR
☒	6355	531.0262	2650.0946	2649.2915	0.8031	1	23	5.8	1	TLLAMQAALLGSSSEEELESENRR + Oxidation (M)
☒	5124	374.6107	1868.0171	1866.9873	1.0298	1	22	11	1	VCLLHEKTPVSEQVTK
☒	6709	717.8600	2867.4109	2868.4116	-1.0007	1	22	6.7	1	VDPMAIVVFHQADIGEYVRHEETLT
☒	5192	945.4705	1888.9264	1887.9195	1.0069	0	22	12	1	HPYFYAPELLYYANK
☒	4729	420.7257	1678.8737	1678.9478	-0.0741	1	22	13	1	RTAVSGIQGQPLPLSR
☒	6086	633.5550	2530.1909	2529.2607	0.9302	1	22	8.1	1	LDLWNVLIDMEMKQDDPSIVR
☒	6267	523.2363	2611.1451	2611.2986	-0.1535	1	22	7	1	QGQGVVTIGAMAKGDSEFMASTVLAK + Oxidation (M)
☒	4704	556.6057	1666.7953	1666.8315	-0.0362	0	22	13	1	IQFSDPAPPSQLDPR
☒	2830	501.7929	1001.5712	1000.5665	1.0047	0	22	21	1	ALQSVQVTR
☒	3211	585.5594	1169.1042	1169.5941	-0.4899	1	22	11	1	FASFAGASRTR
☒	3501	658.7206	1315.4266	1315.7095	-0.2829	0	22	12	1	TALQTSLEALNR

4836	575.2849	1722.8329	1722.8365	-0.0037	1	22	13	1	DAFLGSFLYEYSRR
2873	501.7933	1001.5720	1000.5665	1.0055	0	22	21	1	ALQSVQVTR
3001	352.1701	1053.4885	1054.5519	-1.0635	1	22	16	1	RAEAGAEVPR
3429	649.2910	1296.5674	1296.7078	-0.1403	1	22	16	1	EALPKDWITPK
5163	627.9766	1880.9080	1879.9138	0.9941	0	22	12	1	RPCFSALTPDETYVPK
2810	501.7924	1001.5702	1000.5665	1.0037	0	22	22	1	ALQSVQVTR
5658	556.0375	2220.1209	2220.0369	0.0840	1	22	10	1	DADVDTMLDTLQKQQADWK
6722	956.8116	2867.4130	2868.4116	-0.9987	1	22	7.3	1	VDPMAIVVFHQADIGEYVRHEETLT
2758	488.9435	975.8724	975.6117	0.2608	1	22	15	1	TVAFKVLAK
4810	848.4120	1694.8094	1693.6677	1.1417	1	22	14	1	DRDDSDGSWSGGGGGGGR
3539	671.3093	1340.6040	1339.7572	0.8469	1	22	17	1	TAANAASPLGLAKR
5895	617.8320	2467.2989	2467.2202	0.0787	0	22	9.1	1	GLGITFITVGLMSLGFMSFGGMSI + 2 Oxidation (M)
7104	634.0816	3165.3716	3164.4357	0.9360	0	22	5.1	1	EQQQNMQAVIDIVNFYQDVADHTGEEK + Oxidation (M)
5892	823.4149	2467.2229	2468.4162	-1.1933	1	22	9.3	1	LGIARVSLPGGCAIGARPVNLHIK
3665	692.9847	1383.9548	1384.7497	-0.7948	0	22	13	1	SLPVVIVCASGGAR
4600	409.0083	1632.0041	1632.7777	-0.7736	0	22	13	1	AETANDIAAVMAEVGR + Oxidation (M)
2959	511.3257	1020.6368	1021.4498	-0.8130	0	22	21	1	AADGSVAEMR + Oxidation (M)
3532	667.8365	1333.6584	1334.6830	-1.0246	1	22	18	1	VQVDGATKNFEK
5544	529.4752	2113.8717	2112.8775	0.9941	1	22	9.6	1	VHKECCHGDLLECADDR
3845	719.3306	1436.6466	1437.7650	-1.1183	0	22	16	1	SFTSGMVTILLNR
3972	721.2405	1440.4664	1440.7222	-0.2558	1	22	12	1	RHGIGYPVTGDGGR
4559	540.8870	1619.6392	1620.7644	-1.1253	1	22	13	1	QPKAGQHTGYEYSR
4992	363.0058	1809.9926	1810.7899	-0.7973	0	22	13	1	DAAGMSEDDIITMMVGR
3450	435.5917	1303.7533	1302.7547	0.9986	0	22	18	1	AIVFSALDNLIK
4689	551.9385	1652.7937	1652.8482	-0.0545	1	22	14	1	ISNAAGARYSIQTSSK
4817	851.8478	1701.6810	1702.7906	-1.1096	0	22	13	1	LTTLQCQYSTVMDK + Oxidation (M)
2302	643.7950	642.7877	642.3813	0.4064	0	22	0.54	1	AGAGLVR
2874	501.7933	1001.5720	1000.5665	1.0055	0	22	23	1	ALQSVQVTR
6883	987.8636	2960.5690	2960.4882	0.0808	1	22	6.8	1	TQLMGLTAAEMTVLVGGMRVLGTNHGGSK + 2 Oxidation (M)
4376	781.8824	1561.7502	1561.6864	0.0638	0	22	16	1	MHSSEEMAEILNR + Oxidation (M)
2675	457.2694	912.5242	913.5596	-1.0354	1	22	21	1	LAKLEGVGK
6718	717.8604	2867.4125	2868.4116	-0.9991	1	22	7.9	1	VDPMAIVVFHQADIGEYVRHEETLT
7183	662.2750	3306.3386	3305.5841	0.7546	1	22	4.4	1	TPFYGESGGQIGDTGYIATDLAAFRINDTQK
4290	770.3073	1538.6000	1538.9072	-0.3071	0	22	14	1	NWTIISIIEIPIK
5109	374.4103	1867.0151	1866.9001	0.1150	1	22	13	1	ESAIISMLGEMDLMRR + Oxidation (M)
4590	408.1931	1628.7433	1627.8464	0.8969	1	22	16	1	QMADNGRLQPLLTR + Oxidation (M)
5032	608.6361	1822.8865	1822.9789	-0.0924	1	22	14	1	TLAEALADTPAPDGKVVR
2572	414.7599	827.5052	826.4661	1.0392	0	22	20	1	AGLGSPLGR
3040	542.2988	1082.5830	1082.5872	-0.0042	1	22	19	1	YIYEIARR
3362	633.9963	1265.9780	1266.6092	-0.6311	1	22	12	1	QDLTGDFESKK
6474	895.1311	2682.3715	2681.3330	1.0385	1	22	8.8	1	ENLGSTQEDALNHINGMIEDLIKK
6294	523.4373	2612.1501	2611.2986	0.8515	1	22	8.4	1	QGQGVVTIGAMAKGDSEFMASTVLAK + Oxidation (M)
2969	516.3123	1030.6100	1031.4892	-0.8792	0	22	24	1	MGVMDLPGGR
3451	435.5917	1303.7533	1302.7468	1.0064	0	22	20	1	MSLLELVEILK + Oxidation (M)
7396	708.1283	3535.6051	3535.7612	-0.1561	1	22	5	1	EGTFDALQTLSLCLAAMTGMVRDLEPVPEVLK + 2 Oxidation (M)
4925	441.6995	1762.7689	1763.8546	-1.0857	0	22	15	1	SDLSGVDVTMLNMLNR
3582	679.9364	1357.8582	1356.8493	1.0089	0	22	18	1	GAVAVVISILLFR
3082	561.2367	1120.4588	1120.6175	-0.1587	1	21	22	1	FVRMSLGGVR
2952	1016.9592	1015.9519	1016.5138	-0.5619	1	21	17	1	AGDKAEIDAK
4285	769.9025	1537.7904	1538.8780	-1.0876	1	21	17	1	GEGGEIVLREILVR
5637	734.6761	2201.0065	2202.0602	-1.0537	0	21	12	1	FLTLWSAQDVCVSFGIGMR + Oxidation (M)
3578	679.6402	1357.2658	1357.7751	-0.5093	0	21	13	1	ISAMGALSLGLGLR
3757	709.9376	1417.8606	1417.6797	0.1809	0	21	18	1	SGNSVGEDTNALVR
3377	639.1302	1276.2458	1276.6412	-0.3953	0	21	14	1	NGSDEFLLIGGR
5774	589.5022	2353.9797	2355.1715	-1.1918	1	21	9.6	1	MELGSLIRATNLWGYTDLMR + Oxidation (M)
2806	501.7921	1001.5696	1000.5917	0.9780	1	21	26	1	LAKADGIVSK
2848	1002.5789	1001.5716	1001.4964	0.0752	0	21	26	1	NPTASIPMR + Oxidation (M)
2878	501.7934	1001.5722	1000.5665	1.0057	0	21	26	1	ALQSVQVTR
2904	501.7939	1001.5732	1000.5665	1.0067	0	21	26	1	ALQSVQVTR
3148	382.4793	1144.4161	1143.6248	0.7913	0	21	21	1	AVSNSLTISPR
4064	738.3797	1474.7448	1475.7000	-0.9551	0	21	19	1	LMELPQPAEMTGK + 2 Oxidation (M)
5579	538.0200	2148.0509	2149.0764	-1.0255	0	21	12	1	QASHGDLTISSVPAAPTPSASR
2587	846.0851	845.0778	844.4654	0.6124	1	21	19	1	DGKEVAVK
2845	501.7930	1001.5714	1000.5665	1.0049	0	21	26	1	ALQSVQVTR
3406	429.8589	1286.5549	1286.6982	-0.1434	1	21	21	1	LWRLLSENEK
2599	428.2589	854.5032	854.5702	-0.0669	1	21	18	1	KAVGIVIR
4327	515.5747	1543.7023	1543.7954	-0.0931	1	21	19	1	ALELSGNSKNELNR
7570	745.3749	3721.8381	3721.7988	0.0394	1	21	5	1	FNDLGEEHFRGLVLVAFSQYLQQCPFEDHVK
2840	501.7930	1001.5714	1000.5665	1.0049	0	21	27	1	ALQSVQVTR
3084	561.2370	1120.4594	1120.5513	-0.0919	0	21	23	1	DVDGFSLVNR
2586	845.5907	844.5834	843.5065	1.0769	0	21	30	1	LLASLAEK
4071	492.5892	1474.7458	1475.7694	-1.0236	0	21	19	1	ISNAMDFVPLLEK
5848	811.3631	2431.0675	2430.3053	0.7621	1	21	10	1	VSINTVNLTAGPQVMEVTVFRR
2951	508.9229	1015.8312	1015.5451	0.2862	1	21	18	1	KTTWLDPR
5230	639.7133	1916.1181	1915.9163	0.2018	1	21	13	1	WTGEKSDKPTEADDIPK
2852	501.7931	1001.5716	1000.5665	1.0051	0	21	27	1	ALQSVQVTR
4185	752.3911	1502.7676	1501.7372	1.0304	0	21	20	1	AVESLAEGGGSEIQR
2796	500.3782	998.7418	999.4920	-0.7501	1	21	19	1	MAKEVNHR + Oxidation (M)
2865	501.7932	1001.5718	1000.5665	1.0053	0	21	27	1	ALQSVQVTR
3014	352.8374	1055.4904	1055.5975	-0.1071	0	21	23	1	ALEAELAALR
2817	501.7928	1001.5710	1000.5665	1.0045	0	21	27	1	ALQSVQVTR
2858	501.7931	1001.5716	1000.5665	1.0051	0	21	27	1	ALQSVQVTR
2868	501.7933	1001.5720	1000.5665	1.0055	0	21	27	1	ALQSVQVTR
2885	501.7935	1001.5724	1000.5665	1.0059	0	21	27	1	ALQSVQVTR
2907	501.7940	1001.5734	1000.5665	1.0069	0	21	27	1	ALQSVQVTR
5210	477.2469	1904.9585	1903.9107	1.0478	1	21	15	1	TVGMLSNMIAFYDMRR
2889	501.7935	1001.5724	1000.5665	1.0059	0	21	27	1	ALQSVQVTR
2890	501.7935	1001.5724	1000.5665	1.0059	0	21	27	1	ALQSVQVTR
2894	501.7936	1001.5726	1000.5665	1.0061	0	21	27	1	ALQSVQVTR
4262	383.9489	1531.7665	1531.7738	-0.0073	1	21	19	1	LKECCDKPLLEK
5879	616.2446	2460.9493	2460.1075	0.8418	0	21	8.3	1	SLEDGTFNNTTDSCIGLSTSSVR
2877	501.7934	1001.5722	1000.5665	1.0057	0	21	27	1	ALQSVQVTR
8663	1208.3229	4829.2625	4828.4160	0.8465	1	21	2.2	1	LPMQATVISVIVAGDFVLSFLSLGGACSTASVAVLLMDAGEKQCDR + 2 Oxidation (M)
2886	501.7935	1001.5724	1000.5665	1.0059	0	21	28	1	ALQSVQVTR
3405	429.8587	1286.5543	1286.6982	-0.1440	1	21	22	1	LWRLLSENEK
5635	441.2004	2200.9656	2200.0332	0.9324	1	21	12	1	TPGGGGGGGGISPNGGAPLGGKGFSM
6846	980.1655	2937.4747	2938.4859	-1.0112	1	21	8.7	1	VLKNSSLAEFVQSLSQTMGFPQDQIR + Oxidation (M)
5464	682.6790	2045.0152	2044.0206	0.9945	1	21	14	1	RHPYFYAPELLYYANK
2821	501.7928	1001.5710	1000.5665	1.0045	0	21	28	1	ALQSVQVTR
2880	501.7934	1001.5722	1000.5665	1.0057	0	21	28	1	ALQSVQVTR
4902	584.3262	1749.9568	1749.9302	0.0266	0	21	17	1	FSLVQVFEGTQGLTPK

☒	2864	501.7932	1001.5718	1000.5665	1.0053	0	21	28	1	ALQSVQVTR
☒	3161	387.1428	1158.4066	1158.6145	-0.2080	1	21	21	1	RVLEEGGFPR
☒	5704	570.2928	2277.1421	2276.1511	0.9910	1	21	12	1	LFTFHADICTLPDTEKQIK
☒	2835	501.7930	1001.5714	1000.5665	1.0049	0	21	28	1	ALQSVQVTR
☒	2825	501.7929	1001.5712	1000.5665	1.0047	0	21	28	1	ALQSVQVTR
☒	6781	723.3298	2889.2901	2889.4178	-0.1277	1	21	8.3	1	MTADTAVSIPPYQVDDQDVIAELRAR + Oxidation (M)
☒	3278	618.6613	1235.3080	1235.5816	-0.2735	0	21	15	1	MSGTEEAILGGR + Oxidation (M)
☒	8298	744.0189	4458.0697	4457.2738	0.7959	1	21	3	1	YNHMLSATVKIIQMLQHFEHLAPVLVAAVSLWATDYGMK + 2 Oxidation (M)
☒	2563	813.5002	812.4929	812.4578	0.0351	0	21	17	1	MSPLPLR
☒	2863	501.7932	1001.5718	1001.5141	0.0577	0	21	28	1	AIQASVEER
☒	3856	360.7064	1438.7965	1438.8045	-0.0080	1	21	20	1	RHPEYAVSVLLR
☒	2733	471.2376	940.4606	939.5138	0.9469	0	21	23	1	GPQLGEALR
☒	2908	501.7941	1001.5736	1000.5665	1.0071	0	21	28	1	ALQSVQVTR
☒	2968	516.3117	1030.6088	1029.5455	1.0634	0	21	28	1	AVLGEASEVR
☒	3758	710.0737	1418.1328	1418.6864	-0.5536	0	21	14	1	SLHTLFGDELCK
☒	2911	501.7943	1001.5740	1000.5665	1.0075	0	21	29	1	ALQSVQVTR
☒	5823	482.4261	2407.0941	2406.1486	0.9456	1	21	12	1	TLVSGSGDRTVCLWDVEAGEQK
☒	2909	501.7942	1001.5738	1000.5665	1.0073	0	21	29	1	ALQSVQVTR
☒	6271	523.4366	2612.1466	2611.2986	0.8480	1	21	9.6	1	QGQGVVTIGAMAKGDSEFMASTVLAK + Oxidation (M)
☒	2884	501.7935	1001.5724	1000.5665	1.0059	0	21	29	1	ALQSVQVTR
☒	3030	535.6406	1069.2666	1069.6244	-0.3577	1	21	15	1	VLRLVEEGR
☒	5543	529.4752	2113.8717	2112.8775	0.9941	1	21	12	1	VHKECCHGDLLECADDR
☒	5542	1057.5104	2113.0062	2113.1024	-0.0961	1	21	14	1	MATGVDHNRISLLMAVLEK + Oxidation (M)
☒	2368	686.9057	685.8984	686.4075	-0.5091	0	21	2.9	1	VAGVVSR
☒	2905	501.7940	1001.5734	1000.5665	1.0069	0	21	29	1	ALQSVQVTR
☒	7165	1093.9573	3278.8501	3279.5844	-0.7343	1	21	6	1	AQSFLHNQVRSIVGSLEHVGSGAWAPDDMR + Oxidation (M)
☒	5164	627.9766	1880.9080	1879.9138	0.9941	0	21	16	1	RPCFSALTPDETYVPK
☒	6098	635.2844	2537.1085	2537.1604	-0.0520	1	21	10	1	YGINGKMDWNALLEDQASNENR
☒	3229	592.8633	1183.7120	1184.6149	-0.9029	0	21	23	1	ASTLEVNPQAR
☒	4726	420.2251	1676.8713	1675.9257	0.9456	1	21	19	1	VVVYRDQLTIEVSR
☒	4010	726.8576	1451.7006	1451.7595	-0.0588	1	21	21	1	AFKQMPVIPSYR + Oxidation (M)
☒	5060	615.3369	1842.9889	1842.9159	0.0730	1	21	18	1	NGNSRAGWLPVCGQVTK
☒	3645	690.4174	1378.8202	1379.7157	-0.8954	1	21	21	1	SALRSLSGEFSAR
☒	3846	719.3328	1436.6510	1437.7650	-1.1139	0	21	21	1	SFTSGMVTILLNR
☒	8278	1115.2676	4457.0413	4457.2738	-0.2325	1	21	3.2	1	YNHMLSATVKIIQMLQHFEHLAPVLVAAVSLWATDYGMK + 2 Oxidation (M)
☒	3626	685.9323	1369.8500	1370.7050	-0.8550	1	21	21	1	MSLKSYMQLVR + Oxidation (M)
☒	4506	399.6995	1594.7689	1595.7726	-1.0037	1	21	19	1	EATEAFQRQMITR + Oxidation (M)
☒	6791	724.8066	2895.1973	2895.4946	-0.2973	1	21	7.6	1	SLGSDADSAGSLIQPMQIPGIIMPGLRR + Oxidation (M)
☒	2408	356.7198	711.4250	712.4119	-0.9869	0	21	2.8	1	DGAPLLK
☒	2972	518.2718	1034.5290	1035.5349	-1.0058	0	21	28	1	FDAQSSIIR
☒	4263	383.9490	1531.7669	1532.7003	-0.9334	1	21	21	1	KEPAVPAMPAGGMDF + Oxidation (M)
☒	4599	409.0082	1632.0037	1631.8631	0.1406	0	21	18	1	TAAPAPAPVPASAVEQR
☒	4685	551.6044	1651.7914	1651.9032	-0.1118	0	21	19	1	QLEPLNDLLDLLEK
☒	5487	515.2958	2057.1541	2058.0303	-0.8762	0	21	14	1	LDTVALSPEIDALMNDIGR + Oxidation (M)
☒	7820	670.6158	4017.6511	4016.7191	0.9320	0	21	3.4	1	SLQEGAEGGQGCSEAWATLGWAAAGGFGGGGGACTAGGGGGGYR
☒	4070	738.3801	1474.7456	1474.7701	-0.0245	1	21	22	1	VRDEVLIDMLEK + Oxidation (M)
☒	5662	1115.2737	2228.5328	2227.4759	1.0570	0	21	9.8	1	LLQLLLQLLLLLLLLAAGGAR
☒	4717	419.5056	1673.9933	1674.9226	-0.9293	1	21	19	1	LTCLSKSGDIVELLK
☒	8280	1115.2695	4457.0489	4457.2738	-0.2249	1	21	3.4	1	YNHMLSATVKIIQMLQHFEHLAPVLVAAVSLWATDYGMK + 2 Oxidation (M)
☒	5155	627.6433	1879.9081	1879.9138	-0.0058	1	21	18	1	RPCFSALTPDETYVPK
☒	4974	899.4188	1796.8230	1796.8978	-0.0748	0	21	18	1	MTTQFSVADIELSLSR
☒	4941	593.2517	1776.7333	1777.9322	-1.1990	1	21	17	1	SALRSLSGQFSEEVLR
☒	2815	1002.5782	1001.5709	1001.5142	0.0568	0	21	31	1	SPQALSNGTK
☒	4714	557.9266	1670.7580	1670.8549	-0.0969	1	21	20	1	VIDHAIETEKMIEK + Oxidation (M)
☒	2547	401.2607	800.5068	799.4552	1.0517	1	21	29	1	TPKGEIR
☒	2681	457.7712	913.5278	914.5549	-1.0270	1	21	31	1	ALKSVLER
☒	4768	423.9657	1691.8337	1691.8301	0.0036	0	21	20	1	MQQLQNVIEAAFER + Oxidation (M)
☒	4890	582.2462	1743.7168	1742.9163	0.8005	1	21	18	1	DLGIDINVITGTGKDGR
☒	5702	570.2925	2277.1409	2276.1511	0.9898	1	21	14	1	LFTFHADICTLPDTEKQIK
☒	2795	500.3779	998.7412	997.6284	1.1128	0	20	22	1	ANLTVVLLR
☒	3975	361.2082	1440.8037	1440.8817	-0.0780	1	20	22	1	SKWSVVAILGILR
☒	5240	481.2393	1920.9281	1919.9952	0.9329	1	20	17	1	GYDSAGVAILNDGKINVSK
☒	5779	589.5388	2354.1261	2354.1325	-0.0064	1	20	14	1	HLVDEPQNLIKQNCDQFEK
☒	6231	522.8647	2609.2871	2609.3352	-0.0481	1	20	12	1	WFLKGYQNANYLSLFLMVATSK + Oxidation (M)
☒	4827	571.2671	1710.7795	1710.9152	-0.1358	1	20	19	1	GINGVKIDEQVTVDPK
☒	4856	863.4142	1724.8138	1723.9944	0.8194	1	20	19	1	RLTELLGAQDGALLVR
☒	5657	556.0364	2220.1165	2220.1426	-0.0261	1	20	15	1	WLAQGTIYPDVIESAGSKTGK
☒	5538	528.0212	2108.0557	2109.1075	-1.0518	0	20	16	1	QAVPLMRPEIPLVGTGMER + Oxidation (M)
☒	6391	534.0693	2665.3101	2665.2150	0.0951	0	20	12	1	NHGNNEADAALQGLAQQGVDMEDLR
☒	5188	944.9690	1887.9234	1887.9195	0.0039	0	20	19	1	HPYFYAPELLYYANK
☒	4715	558.6714	1672.9924	1672.9036	0.0888	1	20	19	1	TPVFDIDERVELLK
☒	2995	525.8384	1049.6622	1049.4746	0.1876	1	20	27	1	MRGTSHSMK + Oxidation (M)
☒	4126	741.8987	1481.7828	1481.7838	-0.0010	0	20	22	1	TDVHEIVLVGGSTR
☒	3663	692.4933	1382.9720	1383.6890	-0.7170	0	20	18	1	MISGMYLGEIVR + Oxidation (M)
☒	4132	742.4007	1482.7868	1483.7783	-0.9915	1	20	22	1	EEGVIFKNHINGK
☒	7078	781.1074	3120.4005	3119.5274	0.8731	1	20	8.3	1	NEHPKFPDGGLMTQYLDSLPVGAYIDVK + Oxidation (M)
☒	6268	523.2363	2611.1451	2611.2986	-0.1535	1	20	11	1	QGQGVVTIGAMAKGDSEFMASTVLAK + Oxidation (M)
☒	5241	481.2397	1920.9297	1919.9662	0.9635	0	20	18	1	MIAVQYLLLDEQSEPR + Oxidation (M)
☒	4670	412.2227	1644.8617	1644.7202	0.1415	0	20	22	1	YVSFQDIDCTGNAR
☒	4721	419.9544	1675.7885	1675.7909	-0.0024	1	20	22	1	MQSVPLSEEMHKTK + 2 Oxidation (M)
☒	6977	1001.1348	3000.3826	2999.3940	0.9886	1	20	9.3	1	CCTESLVNRRPCFSALTPDETYVPK
☒	5699	570.0423	2276.1401	2276.1511	-0.0110	1	20	15	1	LFTFHADICTLPDTEKQIK
☒	4331	773.4825	1544.9504	1543.7671	1.1834	1	20	22	1	AAFFEDYLDGIRK
☒	2653	450.5571	899.0996	899.5440	-0.4444	0	20	21	1	PTIIVTTR
☒	8417	1129.7804	4515.0925	4514.2140	0.8785	1	20	3.5	1	MEAPPVTMMPVTGGTINMMEYLLQGSVLDHSLESLIHRLR + 3 Oxidation (M)
☒	3010	528.2489	1054.4832	1054.5706	-0.0873	1	20	27	1	IAVMGKHQR + Oxidation (M)
☒	3281	621.2499	1240.4852	1241.5823	-1.0970	1	20	24	1	TCTYESLGRR
☒	4570	541.6475	1621.9207	1621.8199	0.1008	1	20	22	1	LAVPADGPEKAETPVE
☒	2662	453.3418	904.6690	903.4960	1.1730	1	20	32	1	GGVKGALMR + Oxidation (M)
☒	3262	610.1808	1218.3470	1217.5789	0.7681	0	20	20	1	AGRPQFDDQGK
☒	3678	694.8071	1387.5996	1386.7759	0.8238	0	20	26	1	EVLLEGPSGVLFK
☒	4014	727.7715	1453.5284	1452.8123	0.7162	0	20	20	1	LDQQIPMLLVQR
☒	5336	664.0215	1989.0427	1987.9707	1.0720	0	20	19	1	INLEGLACETVPTPMATR + Oxidation (M)
☒	7099	791.8459	3163.3545	3163.4334	-0.0789	1	20	7.6	1	MDQIVCDHLKLDVPAADMTEWSAMVEK + 2 Oxidation (M)
☒	4581	407.6929	1626.7425	1627.8464	-1.1039	1	20	23	1	QMADNGRLQPLLTR + Oxidation (M)
☒	4682	413.9548	1651.7901	1651.9661	-0.1760	0	20	22	1	LFQLQPLALLGTPGGK
☒	6237	522.8649	2609.2881	2608.1819	1.1063	1	20	13	1	ASENAEVDIPQAMVDTELDRMMK + Oxidation (M)
☒	3730	705.2715	1408.5284	1409.6973	-1.1688	0	20	22	1	MLYVVGIGSGNER + Oxidation (M)
☒	6196	647.5354	2586.1125	2586.2642	-0.1517	1	20	12	1	AACNCLKNAAAGVSGLNAGNAASIPSK
☒	7175	657.0894	3280.4106	3279.5470	0.8636	1	20	7.7	1	YNLNYIKMEGGIGCMVNGAGLAMATMDIIK + 2 Oxidation (M)
☒	8244	736.4940	4412.9203	4413.0736	-0.1532	1	20	3.4	1	EENPIEVEAKEVGLNYVDLDGTVGCMVNGAGLAMATMDLIK + 3 Oxidation (M)

⊠ 2764	978.4300	977.4227	976.5454	0.8773	1	20	34	1	FDSLLARR
⊠ 7578	931.7205	3722.8529	3721.7988	1.0541	1	20	6.6	1	FNDLGEEHFRGLVLVAFSQYLQQCPFEDHVK
⊠ 4767	423.9655	1691.8329	1691.7495	0.0834	0	20	23	1	CYGIVTMSSSTEVSR + Oxidation (M)
⊠ 5157	627.6436	1879.9090	1879.9138	-0.0049	0	20	20	1	RPCFSALTPDETYVPK
⊠ 4234	380.4400	1517.7309	1518.7386	-1.0077	1	20	25	1	NTTETQVQSARER
⊠ 3801	473.9151	1418.7235	1419.9064	-1.1830	0	20	27	1	LSAVKPLIPILEK
⊠ 5012	363.2066	1810.9966	1811.8571	-0.8605	0	20	21	1	MSSLSTTAIQSLDEASR + Oxidation (M)
⊠ 5560	1066.5314	2131.0482	2129.9908	1.0574	1	20	18	1	MDFSSTRVLCIGDVMLDR + Oxidation (M)
⊠ 3404	429.5883	1285.7431	1285.7129	0.0302	0	20	29	1	VEAGESIVEIIK
⊠ 2505	779.3552	778.3479	778.4259	-0.0779	0	20	14	1	SLLATMK + Oxidation (M)
⊠ 5162	627.9766	1880.9080	1879.9138	0.9941	0	20	20	1	RPCFSALTPDETYVPK
⊠ 2462	373.6923	745.3700	744.4382	0.9319	0	20	18	1	GVTVIEK
⊠ 4231	759.3727	1516.7308	1516.8031	-0.0723	0	20	26	1	SLTLRPEGTAAMVR + Oxidation (M)
⊠ 4566	541.2223	1620.6451	1620.7644	-0.1194	1	20	21	1	QPKAGQHTGYEYSR
⊠ 6162	512.0334	2555.1306	2554.0815	1.0492	0	20	13	1	HFSMMILSDDGVVCYNSDYAAK + 2 Oxidation (M)
⊠ 7047	764.5813	3054.2961	3055.4790	-1.1829	1	20	8.9	1	EGRMHILGIMNQCISAPNAELSQYAPR
⊠ 2888	501.7935	1001.5724	1000.6281	0.9444	1	20	37	1	ALKTNTIIK
⊠ 3564	676.1118	1350.2090	1350.6966	-0.4875	0	20	19	1	VVSGYCDAIVIR
⊠ 6724	717.8615	2867.4169	2867.1426	0.2743	1	20	12	1	MSNSCMSATSPRSSMASSASSSQEHSK + 3 Oxidation (M)
⊠ 8331	895.1977	4470.9521	4470.1644	0.7877	1	20	3.4	1	NPKQFDVLLTGNMFGDILSDEAAMLTGSIGMLPSASLNVEDK + 2 Oxidation (M)
⊠ 2670	907.8326	906.8253	906.4303	0.3950	0	20	23	1	MCAVVSPK + Oxidation (M)
⊠ 4737	421.9476	1683.7613	1683.7410	0.0203	0	20	23	1	FSESMNQELNVNEK + Oxidation (M)
⊠ 6699	717.8577	2867.4017	2868.4116	-1.0099	1	20	12	1	VDPMAIVVFHQADIGEYVRHEETLT
⊠ 7585	931.9696	3723.8493	3723.7978	0.0515	1	20	7	1	LYYGGGISNAEQAKEMAQYADTVVVGNIIYDDIK + Oxidation (M)
⊠ 6369	531.2281	2651.1041	2650.2333	0.8708	0	20	11	1	TLSQCEPVYEAVQGWQEDISGVR
⊠ 6980	1001.1353	3000.3841	3000.5219	-0.1379	1	20	11	1	TGITFSKITELSEYLYVSTLYDSSGVK
⊠ 6050	631.5430	2522.1429	2523.1767	-1.0338	0	20	14	1	AAGSNALGIIMTGMGDDGAAGLLEMR + 2 Oxidation (M)
⊠ 4537	804.2921	1606.5696	1607.7614	-1.1917	0	20	19	1	ELGFVQPSGVTDGMR + Oxidation (M)
⊠ 2508	390.7325	779.4504	778.4014	1.0491	0	20	14	1	AVSLGFW
⊠ 5213	636.6395	1906.8967	1906.9135	-0.0168	0	20	21	1	LFTFHADICTLPDTEK
⊠ 3742	707.9708	1413.9270	1412.7987	1.1283	1	20	24	1	LEAATVADLGVAKR
⊠ 6324	526.8354	2629.1406	2630.1267	-0.9861	1	20	13	1	DAMICAGASGVSSCMGDSGGPLVCKK + Oxidation (M)
⊠ 2550	804.1991	803.1918	803.4137	-0.2219	1	20	25	1	TAKEEAR
⊠ 3242	598.7440	1195.4734	1194.6357	0.8378	0	20	27	1	NGEVPPTLVNR
⊠ 5148	626.6122	1876.8148	1877.0047	-0.1899	0	20	20	1	EIEFFVQTQQLILGGR
⊠ 6250	522.8658	2609.2926	2608.1819	1.1108	1	20	14	1	ASENAEVDIPQAMVDTELDRMMK + Oxidation (M)
⊠ 5891	1234.6185	2467.2224	2467.2668	-0.0444	0	20	16	1	IGLVSQEPVLFSSSIMENIGYGK
⊠ 7580	931.7209	3722.8545	3721.7988	1.0557	1	20	7.2	1	FNDLGEEHFRGLVLVAFSQYLQQCPFEDHVK
⊠ 4033	488.5694	1462.6864	1461.7180	0.9683	1	20	28	1	EAVAAVAICCRSR
⊠ 4075	738.3804	1474.7462	1474.7701	-0.0239	1	20	28	1	VRDEVLIDMLEK + Oxidation (M)
⊠ 6111	509.2375	2541.1511	2541.2897	-0.1386	1	20	14	1	LMYQVKPGETLESIAADFRTTR + Oxidation (M)
⊠ 7388	590.1077	3534.6025	3533.5393	1.0632	1	20	7.8	1	IIQHTSFQEMKNNPSTNYTMMPEEMMNQK + 2 Oxidation (M)
⊠ 2745	477.4993	952.9840	952.5706	0.4135	0	20	19	1	ILDPAIVGR
⊠ 3455	435.5919	1303.7539	1304.6799	-0.9260	1	20	31	1	DLMQGILQKFP + Oxidation (M)
⊠ 4565	541.2220	1620.6442	1620.7644	-0.1203	1	20	23	1	QPKAGQHTGYEYSR
⊠ 5687	566.7567	2262.9977	2264.1536	-1.1559	1	20	17	1	ANIDEFTETTSKAIEVIGGAAK
⊠ 6921	600.1080	2995.5036	2996.4960	-0.9924	0	20	12	1	YAQNHDIPFYIVSGGVDFFIEPLVEK
⊠ 2798	501.2438	1000.4730	1001.5618	-1.0887	1	20	38	1	VTLARADTR
⊠ 4791	564.9840	1691.9302	1690.8097	1.1205	1	20	24	1	LMAAHTGQDYDKVAR + Oxidation (M)
⊠ 6628	470.7257	2818.3105	2819.4640	-1.1535	1	20	13	1	GVIDGIINGVGLAGFCIGEEIKYVGGGR
⊠ 4189	376.9425	1503.7409	1503.7277	0.0132	1	20	28	1	SASPPDSASVRSTSR
⊠ 4954	596.3110	1785.9112	1785.9261	-0.0149	1	20	24	1	VESDREHFVDLLLSK
⊠ 5826	603.0161	2408.0353	2409.2151	-1.1798	1	20	15	1	QMFRYTKPAGTFATFTAASAVK + Oxidation (M)
⊠ 4591	543.9218	1628.7436	1627.8464	0.8972	1	20	26	1	QMADNGRLQPLLTR + Oxidation (M)
⊠ 2610	434.7573	867.5000	868.3927	-0.8926	0	19	27	1	DSQGTFSK
⊠ 3092	375.5699	1123.6879	1123.6713	0.0166	1	19	30	1	QKNLVIPNAK
⊠ 6624	564.4175	2817.0511	2816.4419	0.6093	1	19	9.3	1	VTHVIRNYLDDLDFIDVETPMLAK
⊠ 5769	588.0618	2348.2181	2349.2176	-0.9995	0	19	17	1	INSAGPSIPAEILGLSEVPSAGDR
⊠ 6683	715.0742	2856.2677	2855.5255	0.7422	1	19	12	1	MVEYQVKIDTFEGPLDLLLHLINR
⊠ 5360	670.0098	2007.0076	2007.0571	-0.0496	1	19	21	1	LSSLGIAMTQSSVSRWLR + Oxidation (M)
⊠ 3419	647.2523	1292.4900	1291.6958	0.7942	1	19	28	1	DGTKIPMFIVR + Oxidation (M)
⊠ 6290	523.4371	2612.1491	2611.2986	0.8505	1	19	14	1	QGQGVVTIGAMAKGDSEFMASTVLAK + Oxidation (M)
⊠ 5606	1095.5159	2189.0172	2188.0872	0.9300	0	19	19	1	DGGAAAASAGAPVSQAAALGAYVSR
⊠ 3844	718.8129	1435.6112	1434.8082	0.8031	1	19	28	1	TSKTPLSIYLANK
⊠ 6575	1390.7166	2779.4186	2780.4313	-1.0127	1	19	14	1	VQSGMVVGLGSGSTAALMIQYLGDRVR + Oxidation (M)
⊠ 5930	497.8332	2484.1296	2484.2683	-0.1386	1	19	16	1	GAFTKEVDAALLAGEADLAVHCVK
⊠ 5701	570.0434	2276.1445	2277.1674	-1.0230	1	19	19	1	AELEKHHEGLTVELLPMSTK + Oxidation (M)
⊠ 3669	693.8125	1385.6104	1386.6562	-1.0457	0	19	31	1	TMNATVQTGSPHK + Oxidation (M)
⊠ 3989	723.4841	1444.9536	1444.7773	0.1764	0	19	24	1	TTSSPGSAEIIALAK
⊠ 2659	453.3412	904.6678	903.4960	1.1718	1	19	37	1	GGVKGALMR + Oxidation (M)
⊠ 5418	508.2719	2029.0585	2029.9276	-0.8691	0	19	21	1	TGIYHGGEPLCDNVNTQR
⊠ 5813	801.7253	2402.1541	2402.1610	-0.0069	1	19	17	1	EMASDNKMLGEFDLVGIPPAPR + Oxidation (M)
⊠ 6523	908.4015	2722.1827	2721.3830	0.7997	1	19	13	1	LTMTTPYLVPSDDLLRAICTAAQR + Oxidation (M)
⊠ 3459	435.9074	1304.7004	1304.7088	-0.0085	0	19	32	1	HLVDEPQNLIK
⊠ 2750	481.4905	960.9664	960.4335	0.5330	0	19	28	1	TTQAEHMK + Oxidation (M)
⊠ 6084	844.4036	2530.1890	2530.3519	-0.1629	1	19	16	1	VVMGLFIGSLYWQQPLDPRGVR
⊠ 4683	413.9549	1651.7905	1650.9596	0.8309	0	19	26	1	EDLIPFLPKPILEK
⊠ 6514	544.6421	2718.1741	2719.3156	-1.1415	1	19	13	1	VEMERSAGVNNTELEELEMLLAAR + Oxidation (M)
⊠ 5677	562.9899	2247.9305	2246.9354	0.9951	1	19	17	1	ECCHGDLLECADDRADLAK
⊠ 7010	605.0681	3020.3041	3019.4848	0.8193	0	19	11	1	YIFNSLDDIVHVNTVMDLEGGDLLAEK
⊠ 4155	498.2445	1491.7117	1490.8569	0.8548	0	19	30	1	LLATEGVAAVHAALR
⊠ 2818	501.7928	1001.5710	1000.5665	1.0045	0	19	42	1	ALQSVQVTR
⊠ 5106	374.4102	1867.0146	1866.9873	0.0273	1	19	23	1	VCLLHEKTPVSEQVTK
⊠ 2808	501.7923	1001.5700	1000.5454	1.0247	1	19	43	1	ALKWEQAR
⊠ 2856	501.7931	1001.5716	1000.5665	1.0051	0	19	43	1	ALQSVQVTR
⊠ 5681	564.0070	2251.9989	2253.1675	-1.1686	1	19	19	1	VEGTMVTAAAGASYIEVTKIAR + Oxidation (M)
⊠ 2430	728.0206	727.0133	726.4752	0.5381	0	19	5.4	1	VLLAGVR
⊠ 4121	741.4785	1480.9424	1480.8474	0.0951	1	19	25	1	NGGIRLGKPAETIR
⊠ 4725	420.2247	1676.8697	1676.8594	0.0103	1	19	28	1	QFLSSQPRSGAGLSSR
⊠ 6295	523.4373	2612.1501	2611.2986	0.8515	1	19	15	1	QGQGVVTIGAMAKGDSEFMASTVLAK + Oxidation (M)
⊠ 2862	501.7932	1001.5718	1000.5454	1.0265	1	19	43	1	ALKWEQAR
⊠ 4738	562.2612	1683.7618	1683.8398	-0.0780	1	19	27	1	MGLTPMMKQYLEVK + Oxidation (M)
⊠ 5709	570.2932	2277.1437	2276.1511	0.9926	1	19	19	1	LFTFHADICTLPDTEKQIK
⊠ 4741	562.2615	1683.7627	1683.9083	-0.1456	1	19	27	1	LKFPEPQEVVEELK
⊠ 5355	402.0074	2005.0006	2004.9979	0.0028	1	19	23	1	MIDDESVPWIEKYRPK
⊠ 2836	501.7930	1001.5714	1000.5665	1.0049	0	19	44	1	ALQSVQVTR
⊠ 4486	531.5711	1591.6915	1592.8059	-1.1145	0	19	28	1	GAGALWALADGGELHR
⊠ 4470	397.1765	1584.6769	1585.8100	-1.1331	0	19	28	1	DIGVEYVILGHSER
⊠ 6581	558.2737	2786.3321	2786.3738	-0.0417	0	19	15	1	AYHTVFQYMNQLNTTPVLYDQLK
⊠ 2737	473.5822	945.1498	944.5080	0.6419	0	19	32	1	FAVALPDGR
⊠ 4471	529.2330	1584.6772	1584.8909	-0.2137	0	19	28	1	MLELGLKPSDILTR

☒	5201	633.4423	1897.3051	1896.1594	1.1457	1	19	16	1	RMALVELAVLVVIIVSR + Oxidation (M)
☒	5909	825.0687	2472.1843	2471.2696	0.9147	1	19	18	1	DASIEAYNKPGSEKFVFLLSTR
☒	2954	510.2878	1018.5610	1018.6076	-0.0466	0	19	44	1	WLPVHVLR
☒	6856	735.6288	2938.4861	2938.4092	0.0769	0	19	14	1	LADDMFETMYDAPGIGLAAIQIAEPVR + 2 Oxidation (M)
☒	4068	738.3800	1474.7454	1474.8065	-0.0610	1	19	32	1	LKAIETAMTQIEK
☒	2450	737.9213	736.9140	736.3504	0.5636	0	19	4	1	ADAGFTR
☒	2820	501.7928	1001.5710	1000.5917	0.9794	0	19	44	1	LAAAGTVTLGK
☒	5175	471.7591	1883.0073	1883.9880	-0.9807	0	19	24	1	APEPLPSPEESPLPVPTK
☒	2619	438.4972	874.9798	874.3967	0.5832	0	19	34	1	MSQEQPR
☒	4603	409.5087	1634.0057	1633.8423	0.1634	1	19	26	1	LEDAISAYERGAALR
☒	3673	694.3129	1386.6112	1386.6562	-0.0449	0	19	33	1	TMNATVQTGSPHK + Oxidation (M)
☒	4942	445.4405	1777.7329	1778.9097	-1.1768	1	19	25	1	FVNSGGEATMSAIRLAR
☒	5014	453.7566	1810.9973	1811.9378	-0.9405	1	19	26	1	IDDKVSIPGPVTGTGNSR
☒	2665	453.3419	904.6692	903.5211	1.1481	0	19	41	1	ATALLMR + Oxidation (M)
☒	2955	510.2885	1018.5624	1018.6076	-0.0452	0	19	45	1	WLPVHVLR
☒	6063	633.2789	2529.0865	2528.2251	0.8614	0	19	16	1	ADVTTICVGTAASMASFVLAGGTQGK + Oxidation (M)
☒	8300	892.6230	4458.0786	4459.2699	-1.1913	1	19	4.9	1	LDGSIPPALADLNSLGYLNISYNNLSGEIPFKGHLVTFDER
☒	5484	412.4371	2057.1491	2057.0537	0.0954	0	19	22	1	VDMNPAGGMLVTPTITIPAK + 2 Oxidation (M)
☒	4127	494.9352	1481.7838	1481.7838	-0.0001	0	19	31	1	TDVHEIVLVGGSTR
☒	5129	374.6108	1868.0176	1866.9873	1.0303	1	19	25	1	VCLLHEKTPVSEQVTK
☒	6367	663.7816	2651.0973	2652.2616	-1.1643	1	19	13	1	KASNGIRPPWSGDESHFLTHCTR
☒	5761	586.0109	2340.0145	2339.1110	0.9035	1	19	18	1	YPQPFSISDISKFFEYESR
☒	5974	500.4276	2497.1016	2498.2329	-1.1313	0	19	17	1	EGDVITIIGVPEPGWFEGELEGR
☒	2811	501.7925	1001.5704	1000.5665	1.0039	0	19	46	1	ALQSVQVTR
☒	3977	721.7355	1441.4564	1440.7222	0.7342	1	19	23	1	RHGIGYPVTGDGGR
☒	3361	633.3085	1264.6024	1265.7343	-1.1319	0	19	36	1	ELLAGDPAILVR
☒	4490	531.5794	1591.7164	1591.7487	-0.0323	0	19	30	1	NMTASQGPCALLWK + Oxidation (M)
☒	3063	553.2987	1104.5828	1104.5849	-0.0020	0	19	42	1	LMAVSEQVTK
☒	4959	447.7373	1786.9201	1785.8455	1.0746	1	19	28	1	YGDSMTEADKTKPLSK + Oxidation (M)
☒	2732	471.2376	940.4606	940.4879	-0.0272	0	19	37	1	YLAQHPGR
☒	3628	686.3936	1370.7726	1371.6942	-0.9216	1	19	35	1	RGHHGCPLVSPR
☒	4587	543.5869	1627.7389	1628.8304	-1.0915	0	19	30	1	QEMIAQSSRPEVVR
☒	7251	677.5266	3382.5966	3382.6795	-0.0829	1	19	10	1	MVSRPGDLPALEAGPGSSEGLLGGMSIPAGMTRR + Oxidation (M)
☒	6701	574.4885	2867.4061	2866.5058	0.9003	0	19	15	1	MLHPVAGRPMVQHVVDQLQQVNLNK + Oxidation (M)
☒	3418	646.5129	1291.0112	1291.7248	-0.7136	0	19	24	1	GDQLHILLEVR
☒	4382	783.3471	1564.6796	1564.7807	-0.1010	0	19	29	1	ISYIDSNLMVEGPK
☒	2366	686.2808	685.2735	685.3871	-0.1136	0	19	6	1	NAIAAAR
☒	4219	758.0785	1514.1424	1514.8457	-0.7032	0	19	22	1	GQLSPFISDALVLR
☒	2496	770.7244	769.7171	769.4082	0.3089	0	19	13	1	AALAGDPR
☒	4488	398.9361	1591.7153	1592.7212	-1.0059	1	19	31	1	DNNGNRMASSAISEK
☒	4753	422.4454	1685.7525	1685.8698	-0.1173	0	19	30	1	CGDIDPAIVPFLIEK
☒	6773	577.8401	2884.1641	2884.3153	-0.1512	0	19	12	1	NVIAVIGDGAMSAGMAYEAMNNAGALDAR + 2 Oxidation (M)
☒	4160	374.1851	1492.7113	1493.8579	-1.1466	1	19	33	1	VPPPPARPPPRSAR
☒	4472	397.1766	1584.6773	1585.7988	-1.1215	1	19	30	1	SSITYEEFNKQLK
☒	3268	610.1821	1218.3496	1218.5299	-0.1802	0	19	28	1	THLDQMSSER + Oxidation (M)
☒	3538	671.2990	1340.5834	1341.6136	-1.0301	1	19	35	1	STAWMRQGFDK + Oxidation (M)
☒	4036	733.5977	1465.1808	1465.7260	-0.5452	1	19	22	1	DTSATQTTELSKGK
☒	4215	756.9435	1511.8724	1512.6735	-0.8010	0	19	32	1	MTVCNLSIEMGAR + 2 Oxidation (M)
☒	4681	551.6039	1651.7899	1652.8483	-1.0585	1	19	30	1	KFLLPEEFMLNEK + Oxidation (M)
☒	5031	608.6350	1822.8832	1822.9789	-0.0957	1	19	29	1	TLAEALADTPAPDGKVVR
☒	4496	797.3818	1592.7490	1592.7392	0.0098	1	19	32	1	IVMKEEWGDDVEK + Oxidation (M)
☒	2990	524.8252	1047.6358	1047.5025	0.1333	0	19	44	1	TYYSPVYR
☒	2912	502.2951	1002.5756	1002.5498	0.0259	1	19	48	1	LAKWAAESK
☒	8293	744.0180	4458.0643	4457.2738	0.7905	1	19	5.1	1	YNHMLSATVKIIQMLQHFEHLAPVLVAAVSLWATDYGMK + 2 Oxidation (M)
☒	3525	665.2912	1328.5678	1328.6837	-0.1158	0	19	38	1	ENLTTAQFLHR
☒	5366	673.1331	2016.3775	2017.0579	-0.6804	0	19	17	1	DLTTDLIINETIVTTEAR
☒	3035	541.2728	1080.5310	1080.4692	0.0619	0	19	38	1	AMQAAAEAMR + 2 Oxidation (M)
☒	3510	661.0779	1320.1412	1320.6245	-0.4832	1	19	25	1	VYHEQQMKSR + Oxidation (M)
☒	2832	1002.5787	1001.5714	1001.4964	0.0750	0	19	49	1	NPTASIPMR + Oxidation (M)
☒	2991	524.8254	1047.6362	1047.6077	0.0286	0	19	44	1	HTILVQPQL
☒	3435	650.4542	1298.8938	1298.7670	0.1268	1	19	30	1	VAVNNSLSIVKR
☒	5001	363.0066	1809.9966	1808.8727	1.1240	1	19	28	1	DIKASNIMLDAEFNGR + Oxidation (M)
☒	5179	472.0102	1884.0117	1883.9642	0.0475	1	19	27	1	FAWKGSNENGRPPLPSK
☒	7994	838.0058	4184.9926	4186.0681	-1.0755	1	19	6.5	1	IFARLGVHYVIVSAVSGAMGGSASEEFLAESEVGEDTFVR
☒	7052	765.6020	3058.3789	3057.1871	1.1918	1	19	13	1	MEDNDDVQNVYANFDISEEEMEKMM + 2 Oxidation (M)
☒	3971	721.0666	1440.1186	1439.7303	0.3884	1	19	24	1	RMLGASAEEHLAR
☒	4710	557.2585	1668.7537	1668.9662	-0.2125	0	19	32	1	VSLADLIVLAGSAAIEK
☒	8346	895.3989	4471.9581	4473.0311	-1.0730	1	19	4.5	1	IDPDSSIAAALMDMYQSMGDALAQQYGGSAAHNTVFPERQGK + 2 Oxidation (M)
☒	6318	526.4351	2627.1391	2626.2229	0.9162	1	19	16	1	WGLDFMSELNGIIPESEMQMRK + Oxidation (M)
☒	5068	617.6346	1849.8820	1848.9621	0.9198	0	19	29	1	GEAQGEFFLVAVAAIAEK
☒	2418	361.2214	720.4282	720.3476	0.0806	0	19	6.3	1	LAEMGGK + Oxidation (M)
☒	3074	557.7567	1113.4988	1114.6168	-1.1180	1	19	43	1	EVLNRLMPK + Oxidation (M)
☒	5993	500.6283	2498.1051	2498.2329	-0.1278	0	19	19	1	EGDVITIIGVPEPGWFEGELEGR
☒	6204	647.7869	2587.1185	2586.2642	0.8543	1	19	17	1	AACNCLKNAAAGVSGLNAGNAASIPSK
☒	7575	745.5771	3722.8491	3721.7988	1.0504	1	19	9.4	1	FNDLGEEHFRGLVLVAFSQYLQQCPFEDHVK
☒	4879	870.1187	1738.2228	1739.0743	-0.8514	1	18	20	1	SLAGLILMPLTVLKVR + Oxidation (M)
☒	4063	492.5888	1474.7446	1474.7701	-0.0255	1	18	37	1	VRDEVLIDMLEK + Oxidation (M)
☒	4239	507.5511	1519.6315	1519.8470	-0.2156	0	18	34	1	ELGAAHVTALASRPK
☒	5328	994.4174	1986.8202	1985.8459	0.9744	1	18	23	1	DAEDAMDFVATDLMRDR + Oxidation (M)
☒	6170	642.2801	2565.0913	2564.3745	0.7168	1	18	17	1	IATTNSGELLSLTPVEHVCRLVR
☒	2814	501.7927	1001.5708	1001.5757	-0.0049	0	18	51	1	LVVSTQTALA
☒	2663	453.3418	904.6690	904.5130	0.1560	1	18	46	1	DVQKFLR
☒	3594	454.8934	1361.6584	1362.6999	-1.0415	1	18	39	1	ANCKILGDTLMK
☒	3747	708.8205	1415.6264	1414.7277	0.8988	1	18	38	1	ATAARVAGAAGDTQR
☒	6199	518.4301	2587.1141	2587.1465	-0.0324	1	18	17	1	LNVSQAMATMSTGECWEVSKNGR + 2 Oxidation (M)
☒	2664	453.3419	904.6692	903.4960	1.1732	1	18	46	1	GGVKGALMR + Oxidation (M)
☒	6273	436.3651	2612.1469	2612.1860	-0.0391	1	18	17	1	QVTGINGNYGGMAQVYGREGEPCR
☒	6489	899.7515	2696.2327	2695.3276	0.9051	0	18	18	1	MSSEPPPPPQPPTHQTSIGLLDTPR + Oxidation (M)
☒	2419	361.2214	720.4282	721.3983	-0.9701	1	18	6.5	1	KGAPGHR
☒	2738	473.7821	945.5496	945.4590	0.0907	0	18	55	1	IPEVDMAR + Oxidation (M)
☒	3981	722.3364	1442.6582	1442.7729	-0.1146	0	18	37	1	DSLQATAELLQVR
☒	3138	571.8591	1141.7036	1142.6117	-0.9081	1	18	43	1	KMNEPNGIIK
☒	6804	582.8419	2909.1731	2908.4038	0.7693	1	18	12	1	AGCILCGYLKVMPMFLIVMMGMVSR + 2 Oxidation (M)
☒	8361	898.3953	4486.9401	4486.1937	0.7464	1	18	4.6	1	MTVVTITPGAMSFADWRAIYEGASAALTVGAWDAIDASAAAVAR + 2 Oxidation (M)
☒	5875	613.0044	2447.9885	2447.2267	0.7618	1	18	17	1	ADSKPQSRYPVNLLDTPFPMR + Oxidation (M)
☒	3096	565.2646	1128.5146	1128.6866	-0.1720	0	18	46	1	LITGLSIVASR
☒	4148	497.9207	1490.7403	1491.8119	-1.0716	1	18	36	1	DCIIDLFSRIIK
☒	3706	701.3432	1400.6718	1400.5706	0.1013	0	18	40	1	GYGGGGGGGYESGGGR
☒	3604	454.8941	1361.6605	1360.7285	0.9320	1	18	40	1	MFLNLLDAPRR + Oxidation (M)
☒	5017	363.4061	1811.9941	1810.8712	1.1229	1	18	30	1	ACSITAPYFYKAYTR
☒	8317	1118.4949	4469.9505	4469.9455	0.0050	1	18	4.9	1	VASTMSDGANTMIEHDDTLPSQLGTMVINADDEEEEGTMKR + 2 Oxidation (M)

⊠	2971	518.2713	1034.5280	1033.5478	0.9803	0	18	48	1	ETSGLMVVAK
⊠	6973	751.1021	3000.3793	2999.3940	0.9853	1	18	15	1	CCTESLVNRRPCFSALTPDETYVPK
⊠	7228	670.4874	3347.4006	3347.7482	-0.3475	1	18	9.6	1	INAALATLACHAALRAPHYLTIEEMEALLR + Oxidation (M)
⊠	6803	970.7294	2909.1664	2908.3681	0.7982	0	18	12	1	CLNTAELSESDMVPGTILVTEEELSR + Oxidation (M)
⊠	2666	453.3421	904.6696	904.5130	0.1566	1	18	49	1	DVQKFLR
⊠	3605	454.8941	1361.6605	1362.7581	-1.0976	1	18	41	1	DGFTMTKLLPLK
⊠	6681	715.0734	2856.2645	2856.0419	0.2226	0	18	16	1	SAAPADAGMGGMGGMGGMGGMGGMGGMGMF + Oxidation (M)
⊠	2767	490.7040	979.3934	978.4077	0.9858	0	18	44	1	MPSPSDSSR + Oxidation (M)
⊠	4940	889.3739	1776.7332	1775.9096	0.8237	1	18	30	1	MKILSNMMEAIDKPR
⊠	6317	657.7920	2627.1389	2626.2618	0.8771	1	18	18	1	LTDQMEAEIEAVIARIDAAGGMYK + 2 Oxidation (M)
⊠	3593	454.8934	1361.6584	1362.7693	-1.1110	0	18	41	1	IVGAMVQIITYR
⊠	5754	778.5274	2332.5604	2333.1031	-0.5427	0	18	16	1	SHSYILINMLEPVEADAEMR + Oxidation (M)
⊠	6245	435.8892	2609.2915	2609.2722	0.0194	1	18	20	1	HLDGIEHTKEDVVTEQIDFSAAR
⊠	6725	717.8621	2867.4193	2868.4116	-0.9923	1	18	18	1	VDPMAIVVFHQADIGEYVRHEETLT
⊠	7116	799.3604	3193.4125	3193.4784	-0.0659	1	18	13	1	TVLGNFAAFVQKCCAAPDHEACFAVEGPK
⊠	5242	481.4895	1921.9289	1920.9979	0.9310	1	18	29	1	AMVQLFEGSSGINLEKAK
⊠	8329	1118.7451	4470.9513	4470.1145	0.8367	0	18	5	1	ASAQMVSYEIAMGFCLVVVLMVSGSLNMTDIVMSQGSGMAASK
⊠	2774	492.7866	983.5586	984.6080	-1.0493	1	18	45	1	LAAGASVLKR
⊠	5433	680.0051	2036.9935	2038.0946	-1.1011	0	18	28	1	LSVSLNLAADLPVQAVGDEK
⊠	6363	531.0664	2650.2956	2649.1831	1.1126	0	18	20	1	NNDSASSFSIDEFSQLLQDETFR
⊠	4669	548.5864	1642.7374	1642.9116	-0.1742	1	18	36	1	AGEMLGVKLVWAELK
⊠	2766	490.7040	979.3934	978.5181	0.8753	0	18	45	1	VRPHMPSR
⊠	3026	533.8190	1065.6234	1066.4567	-0.8332	1	18	42	1	FKEANQDSE
⊠	5295	490.7790	1959.0869	1958.9825	0.1044	1	18	28	1	RSVEMLYYSKPHFFR
⊠	2641	445.1187	888.2228	888.4665	-0.2436	0	18	43	1	LGATSTSPR
⊠	2773	492.5897	983.1648	983.5651	-0.4003	0	18	28	1	EQKPIELK
⊠	5915	825.4014	2473.1824	2474.2363	-1.0539	1	18	22	1	SSRSESTEMYLVSLGFIGSKPSI
⊠	6788	724.5601	2894.2113	2895.3089	-1.0976	1	18	14	1	IIDADGGMLAMEWEDKCTQLANAPSK + 2 Oxidation (M)
⊠	3822	712.9265	1423.8384	1424.8463	-1.0079	1	18	39	1	AAIQATTKPIQRK
⊠	4450	788.1469	1574.2792	1574.7610	-0.4817	0	18	25	1	DQALSISAMEELPR + Oxidation (M)
⊠	4520	800.6596	1599.3046	1598.7545	0.5502	1	18	24	1	SRGEVASAIQCYMK
⊠	3025	533.3386	1064.6626	1064.7070	-0.0443	0	18	44	1	GKPAVLLVIR
⊠	3458	652.8846	1303.7546	1304.7088	-0.9542	0	18	44	1	HLVDEPQNLIK
⊠	4519	400.7061	1598.7953	1599.8290	-1.0337	1	18	37	1	CDKDEIQLILPTR
⊠	5436	680.8601	2039.5585	2040.0714	-0.5129	0	18	19	1	NMGAHAVVLELLQIPYEK + Oxidation (M)
⊠	6909	748.4921	2989.9393	2989.4174	0.5219	1	18	11	1	YQRMQGNAVNFVGADDAHGAPIMIAAEK + Oxidation (M)
⊠	3375	638.7938	1275.5730	1274.6176	0.9554	0	18	45	1	VMDAIESLPER + Oxidation (M)
⊠	6021	626.5671	2502.2393	2502.1888	0.0505	1	18	22	1	GGASTGTQRFAPLNSWPDNGNLDK
⊠	6650	711.5771	2842.2793	2841.2917	0.9875	1	18	17	1	CCAKPESERMPCTEDYLSLILNR
⊠	6875	740.3982	2957.5637	2957.5393	0.0244	1	18	16	1	RPSTRIYVGNVPIGDGAPIAVQSMTNTK + Oxidation (M)
⊠	6122	638.5382	2550.1237	2549.2546	0.8691	0	18	20	1	VDIWSLGIMALEMIDGEPPYLR + 2 Oxidation (M)
⊠	7510	910.4736	3637.8653	3637.7168	0.1485	1	18	11	1	YFGDGFQYIPGNHGGGAYHHEFTDLQWLKVGK
⊠	5174	628.3703	1882.0891	1880.9046	1.1845	1	18	29	1	KCIAVGMAMDLVLDDSK + Oxidation (M)
⊠	5956	831.7582	2492.2528	2491.2570	0.9958	0	18	23	1	GLVLIAFSQYLQQCPFDEHVK
⊠	6772	962.1683	2883.4831	2883.4874	-0.0044	1	18	18	1	IVVIDASQTMAEVHKDILAVLQAMAW + 2 Oxidation (M)
⊠	6894	746.8327	2983.3017	2984.4325	-1.1308	0	18	15	1	VTFEYIMLNEVNDGVEQAQELADLTK + Oxidation (M)
⊠	8073	607.5770	4245.9881	4245.2528	0.7353	1	18	7.1	1	ELLDVLSVLSLMERNSPIVISPLLMNLTVENDFSTTVK + Oxidation (M)
⊠	6127	639.2998	2553.1701	2554.1720	-1.0019	0	18	21	1	GVTETTPSFMYPYGTSSSQMVLR + Oxidation (M)
⊠	4744	421.9482	1683.7637	1683.8753	-0.1116	0	18	36	1	SVMPEGQLVEPILEK + Oxidation (M)
⊠	6869	986.7994	2957.3764	2958.5459	-1.1696	0	18	17	1	ILNHLLNIGTHALDIGAMTMVLYTFR + 2 Oxidation (M)
⊠	7833	808.3606	4036.7666	4036.8760	-0.1094	1	18	7.4	1	DGLLQNVVADVNNGEEFLSESESEASLEIDSDIKDEK
⊠	2882	501.7934	1001.5722	1000.5665	1.0057	0	18	58	1	ALQSVQVTR
⊠	2461	373.6921	745.3696	744.4017	0.9679	0	18	29	1	EAGVLEK
⊠	4244	508.5836	1522.7290	1523.7984	-1.0694	0	18	39	1	TEEGHPFKPLLEK
⊠	2634	445.1176	888.2206	887.4753	0.7454	0	18	45	1	VPSFVDPK
⊠	3357	631.8724	1261.7302	1260.6826	1.0476	1	18	48	1	SNNNFVIKTPK
⊠	5059	615.3359	1842.9859	1842.9159	0.0700	1	18	35	1	NGNSRAGWLPVCGQVTK
⊠	6651	711.5787	2842.2857	2841.2917	0.9939	1	18	18	1	CCAKPESERMPCTEDYLSLILNR
⊠	4548	808.1529	1614.2912	1614.9055	-0.6142	0	18	26	1	VWDMLILLSGIDLK
⊠	5142	624.5959	1870.7659	1870.9247	-0.1588	1	18	29	1	LTSSQMASYWADLVKR + Oxidation (M)
⊠	2369	687.1843	686.1770	686.3711	-0.1941	0	18	7.2	1	AVGTPSR
⊠	6516	680.8128	2719.2221	2718.3646	0.8575	1	18	19	1	MNEQQLLENIASLAGAINQYKNEK
⊠	4989	363.0057	1809.9921	1810.9036	-0.9115	1	18	34	1	NDQFSLFAMLGKIDGR
⊠	6289	523.4371	2612.1491	2611.2986	0.8505	1	18	20	1	QGQGVVTIGAMAKGDSEFMASTVLAK + Oxidation (M)
⊠	5408	506.0121	2020.0193	2018.9619	1.0574	1	18	31	1	LKPDPNTLCDEFKADEK
⊠	8454	753.8578	4517.1031	4518.1171	-1.0140	1	18	6	1	LDDAPVPQIQEEPSQPQPQPQPQPQTEDEKPTEQRQDDR
⊠	2883	501.7935	1001.5724	1000.5665	1.0059	0	18	60	1	ALQSVQVTR
⊠	4532	535.8986	1604.6740	1605.6915	-1.0176	1	18	38	1	ACEKLSGYNFMDR + Oxidation (M)
⊠	4938	887.8027	1773.5908	1773.9876	-0.3968	1	18	26	1	KIEDNNTLLFIVDLK
⊠	2901	501.7938	1001.5730	1000.5665	1.0065	0	18	60	1	ALQSVQVTR
⊠	5532	1050.7417	2099.4688	2100.0885	-0.6196	1	18	20	1	IVTIDASQPEAMVADKIQR + Oxidation (M)
⊠	6200	647.7863	2587.1161	2586.2642	0.8519	1	18	20	1	AACNCLKNAAAGVSGLNAGNAASIPSK
⊠	6372	664.5668	2654.2381	2654.4214	-0.1833	1	18	21	1	LQSLILNGVLHDMVALNINYDKR + Oxidation (M)
⊠	4594	816.3284	1630.6422	1630.8580	-0.2157	1	18	35	1	KHGEYGFQNALIVR
⊠	5718	762.0639	2283.1699	2284.1270	-0.9571	1	18	27	1	HTGPARVFESEEPAMAAILSR + Oxidation (M)
⊠	7107	792.6320	3166.4989	3166.5182	-0.0193	1	18	16	1	AWQLPLWDEYQDLLKSNFADMANIGGR + Oxidation (M)
⊠	6182	515.2316	2571.1216	2570.2210	0.9006	1	18	21	1	KMDDEYQNFLAELGGTVPESSLK
⊠	3029	356.8478	1067.5216	1066.5964	0.9252	0	18	46	1	WLLVFFSR
⊠	5712	570.2941	2277.1473	2276.1511	0.9962	1	18	27	1	LFTFHADICTLPDTEKQIK
⊠	6478	895.4056	2683.1950	2682.3759	0.8191	1	18	20	1	IPLHRQVTVSADSSSSMNSTTPLVR
⊠	6466	894.4691	2680.3855	2680.3431	0.0423	1	18	21	1	NKPGVYTKVCNYVNWIQQTIAAN
⊠	3668	693.8111	1385.6076	1386.6562	-1.0485	0	18	46	1	TMNATVQTGSPHK + Oxidation (M)
⊠	6174	642.7969	2567.1585	2568.2742	-1.1157	1	18	23	1	KVVSSDVQCTVESFETALDSLVR
⊠	3599	454.8936	1361.6590	1361.7959	-0.1369	0	18	47	1	LLFISLSFPPTK
⊠	4758	846.4075	1690.8004	1690.6442	0.1563	0	18	38	1	NEDENEGEDAEPSEK
⊠	6275	436.3652	2612.1475	2613.3075	-1.1599	1	18	21	1	AVFAETGLADFLSHIGISDHEKEK
⊠	3071	1110.7806	1109.7733	1108.6353	1.1381	0	18	37	1	LILSHANSVR
⊠	4546	807.3797	1612.7448	1613.8373	-1.0924	1	18	41	1	LTQLEGDKEALNQR
⊠	5243	481.4896	1921.9293	1923.0439	-1.1146	1	18	34	1	HRPSVDVLFRSVANAAGK
⊠	7035	1016.4517	3046.3333	3046.3907	-0.0574	1	18	16	1	RSSITQMTPDGSHTCPQAPGVTTSIDMR + Oxidation (M)
⊠	2585	423.2737	844.5328	845.4429	-0.9101	1	18	70	1	TPKNQMK
⊠	6029	838.4005	2512.1797	2512.2672	-0.0875	1	18	25	1	LGVSAVFAPKIGEMYPYEIEQR + Oxidation (M)
⊠	2463	373.9345	745.8544	745.4446	0.4098	1	18	20	1	TAAKSLR
⊠	4129	494.9353	1481.7841	1480.6538	1.1303	1	18	43	1	QCDPEKMITESK + Oxidation (M)
⊠	5479	412.2369	2056.1481	2056.0549	0.0932	0	18	30	1	DDNGKPQIAATLNGTLATTR
⊠	5707	570.2930	2277.1429	2276.1511	0.9918	1	18	28	1	LFTFHADICTLPDTEKQIK
⊠	8311	893.0205	4460.0661	4461.1695	-1.1034	1	18	6.4	1	MASDFLRGEGATSYGGGAAQIFFLTLMGVAEGVLLVLMSYDR + 3 Oxidation (M)
⊠	2636	445.1179	888.2212	888.5181	-0.2969	0	18	49	1	GLFNVLAR
⊠	2967	514.2772	1026.5398	1027.6026	-1.0627	1	18	52	1	RVLTPSDLK
⊠	5885	493.8315	2464.1211	2465.2526	-1.1314	1	18	25	1	RMVFVIGGPYGFSPAVQEAVADR
	6051	505.4907	2522.4171	2521.3064	1.1107	0	18	22	1	AFPIGDGQTISQPYTVASQTSLLK

☒

☒	4993	363.0059	1809.9931	1808.9528	1.0403	1	17	36	1	TLVMALIKQMFGAGADK + Oxidation (M)
☒	5759	585.5098	2338.0101	2339.1110	-1.1009	1	17	25	1	YPQPFSISDISKFFEYESR
☒	3243	598.8154	1195.6162	1194.5954	1.0208	0	17	48	1	MGGEAVYDLLK
☒	2646	894.4138	893.4065	892.4324	0.9741	0	17	55	1	LLSEEMR + Oxidation (M)
☒	3672	694.3128	1386.6110	1386.6562	-0.0451	0	17	47	1	TMNATVQTGSPHK + Oxidation (M)
☒	6910	748.8350	2991.3109	2991.6718	-0.3609	1	17	17	1	SLYPMDALSLIGFLEILSKGLSILNIR + Oxidation (M)
☒	7591	931.9707	3723.8537	3723.7978	0.0559	1	17	12	1	LYYGGGISNAEQAKEMAQYADTVVVGNIIYDDIK + Oxidation (M)
☒	5139	935.8876	1869.7606	1870.9247	-1.1641	1	17	32	1	LTSSQMASYWADLVKR + Oxidation (M)
☒	6436	445.3927	2666.3125	2665.1735	1.1390	0	17	23	1	NGVLPDCLTDGSDVVSDLEQEEMK + Oxidation (M)
☒	4251	766.8545	1531.6944	1532.7406	-1.0461	0	17	45	1	MTTGGGYQIDHVVR
☒	6357	531.0266	2650.0966	2649.2155	0.8812	0	17	19	1	DGTGISDGSSVTGDAAAGFPAGATQAPGSK
☒	5990	500.6280	2498.1036	2498.2329	-0.1293	0	17	24	1	EGDVITIIGVPEPGWFEGELEGR
☒	3976	721.6616	1441.3086	1440.8049	0.5038	1	17	30	1	DSLIGRIGVENLR
☒	5562	534.4775	2133.8809	2132.8094	1.0715	1	17	27	1	KDEPAMGGAGGMGGMGGMGGMDF + Oxidation (M)
☒	6304	436.5321	2613.1489	2612.1860	0.9629	1	17	22	1	QVTGINGNYGGMAQVYGREGEPCR
☒	6305	523.6371	2613.1491	2612.1860	0.9631	1	17	22	1	QVTGINGNYGGMAQVYGREGEPCR
☒	4916	878.9208	1755.8270	1754.8574	0.9696	0	17	39	1	NEPLDLDELQDVDLK
☒	5004	453.7552	1810.9917	1809.8688	1.1229	1	17	37	1	GMFVQLDPGAMMSVRR + Oxidation (M)
☒	4245	763.0380	1524.0614	1523.9035	0.1579	1	17	31	1	LLLGEIQPTSGKIR
☒	2958	511.3253	1020.6360	1021.5015	-0.8654	0	17	61	1	FAGGPSAIMR + Oxidation (M)
☒	6323	526.8353	2629.1401	2630.1267	-0.9866	1	17	22	1	DAMICAGASGVSSCMGDSGGPLVCKK + Oxidation (M)
☒	3098	568.2771	1134.5396	1133.5652	0.9745	0	17	58	1	LGLSSMQGWR
☒	5131	374.6109	1868.0181	1866.9873	1.0308	1	17	36	1	VCLLHEKTPVSEQVTK
☒	5518	694.8566	2081.5480	2080.9632	0.5848	1	17	22	1	SVDKMGIIDGWMGMDIGPK + 2 Oxidation (M)
☒	7601	748.1527	3735.7271	3734.8336	0.8935	0	17	12	1	GAQGPNGTAGAPGIPGHPGPMGHQGEQGVPGITGKPGPPGK + Oxidation (M)
☒	4250	511.5712	1531.6918	1530.8266	0.8651	1	17	46	1	QVIGQAAADLRAYR
☒	6460	534.4699	2667.3131	2667.3465	-0.0334	1	17	24	1	MSSFVQELDKAFEGQLQVLLEEK
☒	5661	742.9952	2225.9638	2225.1263	0.8375	1	17	28	1	KLNIEDLLSQMHHYNAGVK + Oxidation (M)
☒	4876	579.8984	1736.6734	1736.8324	-0.1591	1	17	33	1	AELEKVGSMCDLLEK + Oxidation (M)
☒	2407	356.7196	711.4246	712.4119	-0.9873	0	17	6.3	1	DGAPLLK
☒	6253	522.8662	2609.2946	2608.1819	1.1128	1	17	25	1	ASENAEVDIPQAMVDTELDRMMK + Oxidation (M)
☒	3600	681.8369	1361.6592	1361.6761	-0.0169	0	17	51	1	HLSTILECAYR
☒	6368	531.2277	2651.1021	2651.3849	-0.2828	0	17	20	1	SGLMNLPMLLGVILCSIISGYGVGR + 2 Oxidation (M)
☒	5757	779.7197	2336.1373	2335.1768	0.9605	1	17	29	1	ALGDSAVTYGAGSTAQKDGVAIGAR
☒	2565	408.1916	814.3686	813.4960	0.8727	0	17	66	1	SKPILEK
☒	5517	694.6014	2080.7824	2080.9632	-0.1808	1	17	26	1	SVDKMGIIDGWMGMDIGPK + 2 Oxidation (M)
☒	3230	592.8635	1183.7124	1184.6149	-0.9025	0	17	54	1	ASTLEVNPQAR
☒	5073	927.3725	1852.7304	1852.9539	-0.2235	1	17	31	1	DKSVMMASQSLIQLFR
☒	7043	611.6651	3053.2891	3052.4928	0.7963	0	17	16	1	MMAMCAAFGDTPGVNLEILIGQMVNLLK + Oxidation (M)
☒	4700	416.9779	1663.8825	1662.7771	1.1054	0	17	42	1	VMNETGVINELDEGK + Oxidation (M)
☒	4736	842.8869	1683.7592	1683.8398	-0.0805	1	17	43	1	MGLTPMMKQYLEVK + Oxidation (M)
☒	6244	522.8655	2609.2911	2608.1819	1.1093	1	17	25	1	ASENAEVDIPQAMVDTELDRMMK + Oxidation (M)
☒	7292	858.6277	3430.4817	3431.4985	-1.0168	1	17	13	1	NIDSGNEEEGDIRQFDDDELTGALGDHQTNK
☒	5751	774.2247	2319.6523	2319.2409	0.4114	1	17	20	1	LVGYIRTLIPEETRPFMER
☒	4922	881.0128	1760.0110	1759.8927	0.1184	0	17	39	1	LGMHAEGALPALPQATPA + Oxidation (M)
☒	5598	544.2603	2173.0121	2172.0707	0.9414	1	17	32	1	VYILMAIMNDEGYRNINK + Oxidation (M)
☒	6652	948.4647	2842.3723	2842.2724	0.0998	1	17	22	1	DGTYPPDDPPTNGLATARMMAMVSYR + Oxidation (M)
☒	7420	714.3243	3566.5851	3566.7791	-0.1940	1	17	13	1	MCSVARHMESIMLFTLLGLCVGLAAGTEAAVVK + 2 Oxidation (M)
☒	3073	557.2567	1112.4988	1112.6441	-0.1453	0	17	56	1	TVSVQPLLEK
☒	6599	703.0647	2808.2297	2809.3671	-1.1374	0	17	21	1	EPNAQVSRPIPDIGGGYIPTEEEWR
☒	5706	570.2930	2277.1429	2277.2072	-0.0643	1	17	31	1	LVSCSATLQELAGNLSLMKVK + Oxidation (M)
☒	3228	592.7737	1183.5328	1182.7046	0.8283	1	17	56	1	ILKLMVPPEK + Oxidation (M)
☒	2507	390.7317	779.4488	780.3654	-0.9165	0	17	25	1	ASTDPYK
☒	5244	961.9835	1921.9524	1920.9251	1.0274	1	17	37	1	EEALLKMYNPIEDSNR
☒	6155	426.8619	2555.1277	2555.4363	-0.3086	0	17	24	1	HPPLILFGSVPDPVATQLTIELAK
☒	4484	796.1512	1590.2878	1589.8559	0.4320	1	17	30	1	SSLLRSMNDALIVR + Oxidation (M)
☒	7134	645.4852	3222.3896	3221.5099	0.8797	1	17	15	1	DIDADEDLEIIGSSDNKAIDSNGEGFLIEK
☒	4684	551.6044	1651.7914	1651.8570	-0.0656	0	17	44	1	NGSETFGLTFPLITR
☒	4814	567.6069	1699.7989	1700.7419	-0.9431	1	17	44	1	MVDKSCLGSSDEVMK + Oxidation (M)
☒	5347	1001.9196	2001.8246	2001.0605	0.7642	1	17	32	1	ALDHKSVLTFMSLLSDPK
☒	4766	423.9583	1691.8041	1690.8414	0.9627	1	17	44	1	RLEDEFEPTVLSEK
☒	5403	405.0099	2020.0131	2020.0333	-0.0202	1	17	37	1	TMTSPAKAAPVASGASGMLLK + 2 Oxidation (M)
☒	7298	688.3087	3436.5071	3436.6133	-0.1062	0	17	13	1	DVLDLQWSPDDAYLISGSVDNSCIIWDVNK
☒	4563	811.3282	1620.6418	1620.7644	-0.1226	1	17	41	1	QPKAGQHTGYEYSR
☒	6276	436.3652	2612.1475	2612.2839	-0.1364	1	17	23	1	CGILSSGNNLFFNEDGLRMLVTR
☒	6044	631.3593	2521.4081	2522.2509	-0.8428	1	17	25	1	MSQETIISNLIDMLKVSAGWDR + Oxidation (M)
☒	5555	1066.0237	2130.0328	2130.1837	-0.1509	0	17	34	1	FIINSLTIPLAVQNAFQNK
☒	6015	834.0425	2499.1057	2500.1969	-1.0912	0	17	27	1	ENTEGEYSGLEHEVVPGVVESLK
☒	7169	656.8887	3279.4071	3278.7286	0.6785	1	17	15	1	LIISTGGLGPTYDDKTSEGLALALGVSFEINK
☒	8292	1115.5233	4458.0641	4457.2738	0.7903	1	17	7.5	1	YNHMLSATVKIIQMLQHFEHLAPVLVAAVSLWATDYGMK + 2 Oxidation (M)
☒	3366	636.2498	1270.4850	1270.6993	-0.2143	1	17	50	1	VINLSRDPTTR
☒	5967	833.3713	2497.0921	2498.1846	-1.0926	1	17	25	1	LESGETLDDVMSEAFAVVREASK + Oxidation (M)
☒	6347	662.5469	2646.1585	2645.2908	0.8677	0	17	24	1	VGAVDMTQHQSVGGPYNVQGFPTLK + Oxidation (M)
☒	4933	590.2993	1767.8761	1767.8106	0.0654	1	17	43	1	MATGIGDPQCFKGMAGK
☒	3249	401.9067	1202.6983	1202.6619	0.0364	0	17	61	1	STGASANTLLIR
☒	6286	436.3654	2612.1487	2612.1860	-0.0373	1	17	24	1	QVTGINGNYGGMAQVYGREGEPCR
☒	5041	916.3901	1830.7656	1831.9039	-1.1383	1	17	38	1	YLFSITRDNIFCAGR
☒	5071	617.9774	1850.9104	1851.9876	-1.0773	1	17	40	1	LEMLRESNGPIHSLIK + Oxidation (M)
☒	8656	802.3767	4808.2165	4807.1164	1.1002	0	17	5.7	1	YFGGSGPMAMPVPMMNVINGGAHANNSLDIQECMIMPVSMTSFR + 3 Oxidation (M)
☒	4961	447.7379	1786.9225	1785.8455	1.0770	1	17	44	1	YGDSMTEADKTKPLSK + Oxidation (M)
☒	4062	492.5888	1474.7446	1474.7701	-0.0255	1	17	52	1	VRDEVLIDMLEK + Oxidation (M)
☒	4175	749.6550	1497.2954	1497.7133	-0.4179	1	17	32	1	EMDDYNLVLKSR + Oxidation (M)
☒	4588	407.9422	1627.7397	1628.8046	-1.0649	0	17	47	1	SLHDFIEGLDLNEK
☒	3002	352.1702	1053.4888	1053.5277	-0.0389	1	17	55	1	MSFSVAERK
☒	7217	1111.5947	3331.7623	3331.5415	0.2207	1	17	17	1	EAVFIYETPKVVMPADGGGGNNSDEGHAIDAR + Oxidation (M)
☒	2661	453.3418	904.6690	905.4640	-0.7950	1	17	66	1	KVDEIMR + Oxidation (M)
☒	6494	900.8745	2699.6017	2698.5091	1.0926	0	17	19	1	ATAKPMLGIVGDLSALYDLNALALLR
☒	2822	501.7929	1001.5712	1000.5917	0.9796	0	17	72	1	LAAAGTVTLGK
☒	3520	664.3489	1326.6832	1326.8499	-0.1667	1	17	55	1	LHVNKILHLLK
☒	4505	798.3714	1594.7282	1593.6949	1.0333	0	17	46	1	AAAAMVNVCPSCEK + Oxidation (M)
☒	4598	409.0078	1632.0021	1630.8461	1.1560	1	17	43	1	INLGNGAMGLGRSLDK + Oxidation (M)
☒	7170	820.8592	3279.4077	3279.5470	-0.1393	1	17	15	1	YNLNYIKMEGGIGCMVNGAGLAMATMDIIK + 2 Oxidation (M)
☒	4937	444.2517	1772.9777	1771.9138	1.0639	1	17	44	1	DNTIAIMLDTKGPEVR
☒	5911	1237.1010	2472.1874	2473.0825	-0.8950	0	17	29	1	NEVFVWCGMGDGSVCVINSATR + Oxidation (M)
☒	2464	374.0967	746.1788	747.3511	-1.1723	0	17	26	1	GGEQTTR
☒	6401	534.2692	2666.3096	2667.3465	-1.0369	1	17	27	1	MSSFVQELDKAFEGQLQVLLEEK
☒	3101	568.7788	1135.5430	1135.5655	-0.0225	0	17	63	1	MSNISSIVNR + Oxidation (M)
☒	5481	515.2323	2056.9001	2056.9259	-0.0258	1	17	35	1	MDKLDANVSSEEGFGSVEK + Oxidation (M)
☒	5881	493.6305	2463.1161	2462.2947	0.8215	0	17	28	1	MMGLDLLVSLLALSVSPCIAATR + 2 Oxidation (M)
☒	5713	570.5402	2278.1317	2278.1879	-0.0562	0	17	33	1	QADGAVLATMGETVVLATAVFAK + Oxidation (M)

☒ 4489	531.5792	1591.7158	1591.7487	-0.0329	0	17	48	1	NMTASQGPCALLWK + Oxidation (M)
☒ 4579	407.6927	1626.7417	1627.8464	-1.1047	1	17	49	1	QMADNGRLQPLLTR + Oxidation (M)
☒ 6592	936.6302	2806.8688	2806.2578	0.6109	0	17	17	1	MGFSEHNLVWLDLEMTGLDPTDTR + Oxidation (M)
☒ 8669	1211.0753	4840.2721	4840.4026	-0.1305	1	17	5.8	1	GYNEDPNFISHLPILMDATCNGLQHLSAMVNDFVLAEKVNLLK
☒ 2724	932.5461	931.5388	931.5702	-0.0314	1	17	80	1	AIKSALTTK
☒ 3800	473.9118	1418.7136	1418.6638	0.0498	0	17	56	1	EQVGTGASGEQAGTK
☒ 6190	861.7128	2582.1166	2582.4083	-0.2917	1	17	26	1	WYEMLPLLHLSQTGKHFILLK + Oxidation (M)
☒ 3587	681.8354	1361.6562	1361.6761	-0.0199	0	17	57	1	HLSTILECAYR
☒ 4905	438.4969	1749.9585	1748.8192	1.1393	1	17	46	1	VKNWYDSHMPESLK + Oxidation (M)
☒ 6192	646.7881	2583.1233	2582.3203	0.8030	0	17	26	1	LANSCFDFLGYLSSVPQSPLLVR
☒ 4053	491.5379	1471.5919	1472.7446	-1.1527	1	17	49	1	DGHIKIADFGLCK
☒ 4680	413.9545	1651.7889	1651.8821	-0.0932	1	17	47	1	QYKVEEGAVLFIEK
☒ 6366	884.7050	2651.0932	2652.2238	-1.1306	1	17	22	1	TFAPNSSVNPWDTMSSAKDANIQR + Oxidation (M)
☒ 4213	756.7748	1511.5350	1512.6879	-1.1528	0	17	39	1	EMTGGGTYDALGVAR + Oxidation (M)
☒ 5890	617.5616	2466.2173	2467.2451	-1.0278	1	17	31	1	GNGIKIGTSTGYTTPMMEVLLPR + 2 Oxidation (M)
☒ 7306	1150.5481	3448.6225	3448.6144	0.0080	1	17	16	1	HPSHSESEFESLTYHHVSQLSNSQDALARR
☒ 3603	681.8372	1361.6598	1361.6761	-0.0163	0	17	58	1	HLSTILECAYR
☒ 5593	723.6509	2167.9309	2169.1066	-1.1757	1	17	34	1	ALSEGGAEHEIFVDEALRVK
☒ 5969	500.4268	2497.0976	2498.2098	-1.1121	1	17	28	1	QISKILEEFDVEEQSSTMLEK + Oxidation (M)
☒ 4567	811.3317	1620.6488	1620.8294	-0.1805	0	17	45	1	NLVITFGGGVNEVMR + Oxidation (M)
☒ 5020	454.0073	1812.0001	1812.9218	-0.9217	0	17	44	1	GNPTVEVEVVLESGATGR
☒ 4580	543.2546	1626.7420	1626.9192	-0.1772	1	17	50	1	VTTSLGDNIKLEIPK
☒ 2433	729.4763	728.4690	728.4545	0.0146	0	17	26	1	TLIAGVR
☒ 4159	374.1851	1492.7113	1491.7966	0.9146	1	17	53	1	LKSISSIEDMTLR
☒ 6377	667.2720	2665.0589	2665.3720	-0.3131	0	17	21	1	ERPGVMFADMELIGVPHTIVIGER
☒ 3589	454.8931	1361.6575	1361.6207	0.0368	1	17	59	1	MEFKIMSTTDK + 2 Oxidation (M)
☒ 6568	922.8275	2765.4607	2765.1219	0.3388	0	17	26	1	VNPMGDMMGQMGIDMSSLMGDLMPK + 5 Oxidation (M)
☒ 8291	744.0179	4458.0637	4457.2738	0.7899	1	17	8.2	1	YNHMLSATVKIIQMLQHFEHLAPVLVAAVSLWATDYGMK + 2 Oxidation (M)
☒ 3370	638.0162	1274.0178	1273.7183	0.2996	0	17	43	1	LLVVYPWTQR
☒ 6541	685.7854	2739.1125	2738.3911	0.7214	0	17	21	1	LFVQSVDSPLEMVMDVAAIFLSSPK + Oxidation (M)
☒ 4067	369.6936	1474.7453	1474.7853	-0.0400	1	17	57	1	AAAIEKMVAWLEK + Oxidation (M)
☒ 7821	670.6158	4017.6511	4017.9816	-0.3305	1	17	8.7	1	SVAMSPDFLAGGEHYALEVRGDSMIEAGILDGDLVVIR + Oxidation (M)
☒ 3834	714.6640	1427.3134	1427.7806	-0.4672	0	17	38	1	LLSGMNIGLEIPR + Oxidation (M)
☒ 4125	494.9347	1481.7823	1481.7838	-0.0016	0	17	53	1	TDVHEIVLVGGSTR
☒ 6189	646.5362	2582.1157	2581.2192	0.8965	1	17	27	1	LLQIAYAYEEATGCHSKMPDLGV + Oxidation (M)
☒ 4437	786.3453	1570.6760	1571.7362	-1.0601	0	17	51	1	ALGNSEEATMLQHR + Oxidation (M)
☒ 5870	611.2948	2441.1501	2442.0726	-0.9225	0	17	31	1	EMAMMQEGEHTVTSHSSIIHR + 2 Oxidation (M)
☒ 4943	889.8740	1777.7334	1776.9635	0.7699	1	17	43	1	DGLDIGTRAFLPHLPR
☒ 7038	1017.1335	3048.3787	3049.3701	-0.9914	0	17	21	1	SATMPPPGGASPASAHMGAAGMPMVPPGAMGAR + 2 Oxidation (M)
☒ 5985	625.3021	2497.1793	2497.1835	-0.0042	1	17	31	1	AFDEKLFTFHADICTLPDTEK
☒ 6696	574.2888	2866.4076	2865.4297	0.9779	1	16	27	1	GQPSITATEVSASHFIPESSGRLGYFK
☒ 3052	362.8456	1085.5150	1084.6968	0.8182	1	16	72	1	LILGTRTLAK
☒ 3430	649.5221	1297.0296	1297.5939	-0.5642	0	16	39	1	NEGEVGFEVYR
☒ 4906	438.4969	1749.9585	1749.8753	0.0832	1	16	49	1	CVSLIMQQTLREEK + Oxidation (M)
☒ 5036	457.2527	1824.9817	1823.8182	1.1635	0	16	46	1	SCDVMVENFAPGVLDR + Oxidation (M)
☒ 7361	703.3378	3511.6526	3512.7167	-1.0640	1	16	16	1	IGNEIRSLILNPDFQEMDEMTEILLYAASR + 2 Oxidation (M)
☒ 4691	415.0026	1655.9813	1655.8804	0.1009	0	16	48	1	MLEKPELSNPEIIK + Oxidation (M)
☒ 5400	506.0093	2020.0081	2021.0694	-1.0613	1	16	42	1	VNFGSRKPAPQTTYQVTK
☒ 4023	486.5493	1456.6261	1457.6569	-1.0308	0	16	56	1	MSVAFAGNSTSGSSR
☒ 2365	684.9756	683.9683	684.4283	-0.4599	0	16	2.9	1	GAVVALR
☒ 6283	523.4370	2612.1486	2611.2986	0.8500	1	16	27	1	QGQGVVTIGAMAKGDSEFMASTVLAK + Oxidation (M)
☒ 6409	445.3924	2666.3107	2665.4448	0.8659	1	16	29	1	GLFMTTNPVPVKAALGMLGLNVGPPR + Oxidation (M)
☒ 5228	479.0090	1912.0069	1912.9822	-0.9753	1	16	44	1	IVFLDFEKGIEEFEAK
☒ 6333	660.0355	2636.1129	2635.4077	0.7052	1	16	25	1	QAPDVIMVSLDMSPHLVKQLSLAK + Oxidation (M)
☒ 6573	555.8467	2774.1971	2775.3320	-1.1348	1	16	24	1	GLGEMNADQLWETTMNPETRTLIR
☒ 7370	587.2762	3517.6135	3518.7159	-1.1023	1	16	17	1	KVPPSAVSPAPGSGSSASSGTSTSSSNGIIDVQSMLR + Oxidation (M)
☒ 2544	401.2602	800.5058	801.4643	-0.9585	1	16	73	1	VRAGMLR
☒ 3569	677.0434	1352.0722	1352.5150	-0.4428	0	16	38	1	GEAEGDASDEAMR + Oxidation (M)
☒ 2578	421.2579	840.5012	840.4164	0.0849	0	16	54	1	TGYTMLR
☒ 2756	487.7308	973.4470	973.4505	-0.0035	0	16	85	1	DLGEEHFK
☒ 3613	455.2281	1362.6625	1361.6761	0.9863	0	16	61	1	HLSTILECAYR
☒ 4009	726.8264	1451.6382	1452.6701	-1.0319	0	16	56	1	SGLTGVEMQTMQR + Oxidation (M)
☒ 5078	619.3337	1854.9793	1854.7425	0.2368	1	16	46	1	SMEDDAEEDDERSSIK
☒ 2996	1051.5028	1050.4955	1051.5406	-1.0450	0	16	66	1	IMQVIAMSK + 2 Oxidation (M)
☒ 5402	506.0105	2020.0129	2021.0694	-1.0565	1	16	43	1	VNFGSRKPAPQTTYQVTK
☒ 2896	501.7937	1001.5728	1002.5896	-1.0167	1	16	82	1	AKLSAICLK
☒ 2849	1002.5789	1001.5716	1001.4964	0.0752	0	16	82	1	NPTASIPMR + Oxidation (M)
☒ 3061	368.1875	1101.5407	1102.5692	-1.0286	0	16	76	1	SGIVEGGMLPK + Oxidation (M)
☒ 2794	500.3779	998.7412	999.5362	-0.7950	1	16	58	1	QVHFSARR
☒ 5675	562.9893	2247.9281	2246.9354	0.9927	1	16	32	1	ECCHGDLLECADDRADLAK
☒ 6114	636.5056	2541.9933	2541.4691	0.5242	1	16	23	1	MSLHHALHLVTTFMVKILLIPK
☒ 6855	490.7549	2938.4857	2937.4753	1.0104	1	16	26	1	SPSGNSVLYNAVVSKMAEVTLQSLDANK + Oxidation (M)
☒ 3627	686.1651	1370.3156	1370.7769	-0.4613	0	16	41	1	AVSDSIILDVALR
☒ 6522	545.0526	2720.2266	2719.3375	0.8892	1	16	27	1	ALRGLEVGATGFDEMVDLDSPDALTK
☒ 8169	1089.2306	4352.8933	4353.1151	-0.2218	1	16	8.2	1	NGNAEAVDDGASLHEMALRCILLEKPDSAAITAQCVAAISR + Oxidation (M)
☒ 8259	883.9932	4414.9296	4413.9557	0.9740	1	16	7.9	1	VASTMSGGANTMIEHGDTLPSQLGTMVINTEDEEEEGTMKR + 3 Oxidation (M)
☒ 2734	471.2377	940.4608	940.4879	-0.0270	0	16	67	1	YLAQHPGR
☒ 3077	559.2791	1116.5436	1116.6291	-0.0855	1	16	75	1	AFRGTVLPEK
☒ 3285	623.3459	1244.6772	1244.6361	0.0412	1	16	69	1	QTIDDAPKTTR
☒ 5401	405.0094	2020.0106	2018.9554	1.0552	0	16	44	1	TVNFMVGGPEGLAPEVCAR + Oxidation (M)
☒ 5745	768.3561	2302.0465	2301.0549	0.9915	1	16	36	1	YGVSGYPTLKDGEEAGAYDGPR
☒ 3436	650.8035	1299.5924	1298.7670	0.8254	1	16	65	1	VAVNNSLSIVKR
☒ 4339	774.3293	1546.6440	1545.6381	1.0060	1	16	56	1	WDMDMTRWYDK
☒ 6125	639.0493	2552.1681	2551.2238	0.9443	0	16	31	1	IMQQSLLVSADNAHGVHPNYADR + Oxidation (M)
☒ 6697	574.2891	2866.4091	2865.4708	0.9383	1	16	28	1	LQLAALGYVFPDRAMDHLHALAAGGSR + Oxidation (M)
☒ 2324	656.3813	655.3740	656.3969	-1.0229	0	16	2.3	1	AAIAGVR
☒ 3251	603.2597	1204.5048	1204.6200	-0.1152	0	16	70	1	AVFEVSNAVNR
☒ 4076	492.5904	1474.7494	1474.7701	-0.0207	1	16	61	1	VRDEVLIDMLEK + Oxidation (M)
☒ 4977	451.9756	1803.8733	1802.8621	1.0112	0	16	51	1	FGLCLEPNADAAIAEGR
☒ 5265	486.2761	1941.0753	1940.0625	1.0128	1	16	44	1	RPMSDVIGRVGAAGSNLIK
☒ 4743	421.9481	1683.7633	1683.9658	-0.2025	0	16	53	1	ILIGSDLLAETADLLK
☒ 4017	485.9381	1454.7925	1453.6395	1.1530	0	16	59	1	LEFEEGMDVDVR + Oxidation (M)
☒ 6165	427.0291	2556.1309	2556.2682	-0.1373	1	16	30	1	YPRGNGIGVPLMEEGWEPLEIGK + Oxidation (M)
☒ 5723	762.3909	2284.1509	2282.9566	1.1943	0	16	38	1	VVCEEAGESMMVAAGAGNNDTR + Oxidation (M)
☒ 6030	838.4006	2512.1800	2512.2632	-0.0832	1	16	33	1	KFLDSNALGLTVAALSSYDPQMR + Oxidation (M)
☒ 5181	472.2094	1884.8085	1884.9880	-0.1795	1	16	44	1	DPWTAVPGVRMTLTVAR + Oxidation (M)
☒ 5698	570.0421	2276.1393	2276.1511	-0.0118	1	16	39	1	LFTFHADICTLPDTEKQIK
☒ 5896	618.0309	2468.0945	2467.3397	0.7548	1	16	33	1	SVLFFATTVGCSSLVKDSVILPK
☒ 3606	454.8942	1361.6608	1362.5874	-0.9266	0	16	65	1	MTGGNYSLADYR + Oxidation (M)
☒ 4069	492.5891	1474.7455	1474.7701	-0.0246	1	16	62	1	VRDEVLIDMLEK + Oxidation (M)
☒ 5081	619.7816	1856.3230	1855.8089	0.5141	1	16	33	1	KMMLFYGMSSASAMNR + 2 Oxidation (M)

☒	2674	457.2688	912.5230	911.4092	1.1138	0	16	76	1	TIAGEMMK + 2 Oxidation (M)
☒	2987	523.2360	1044.4574	1044.5651	-0.1076	1	16	84	1	HVIYMARR
☒	5026	605.6665	1813.9777	1813.8821	0.0955	1	16	49	1	ETIGDFWQMIFQRK + Oxidation (M)
☒	5615	731.9030	2192.6872	2193.1140	-0.4268	1	16	26	1	SYANTLVLISSSRMETFFK
☒	6316	526.4347	2627.1371	2626.2450	0.8922	1	16	28	1	VQFEMMGKMGPLQQVMSMLPGAGK + 2 Oxidation (M)
☒	6396	667.5832	2666.3037	2665.2150	1.0887	0	16	31	1	NHGNNEADAALQGLAQQGVDMEDLR
☒	8021	699.5005	4190.9593	4191.0989	-0.1395	1	16	11	1	AIRLIQACVDVLSSNGWLSPALAAMELAQMVTQAMWSK + 2 Oxidation (M)
☒	7687	764.7821	3818.8741	3817.9205	0.9536	1	16	15	1	DSALLWQLLVLLCRQNGSMVGSDIAELLMQDWK + Oxidation (M)
☒	3007	528.2487	1054.4828	1054.6134	-0.1306	0	16	68	1	AINANAAAIVK
☒	5760	780.9037	2339.6893	2340.1328	-0.4435	1	16	26	1	KDPFLFHYNFPPYSVGEAGR
☒	7545	919.6828	3674.7021	3673.7136	0.9885	0	16	16	1	QVLGGMALMAAFSAPVLAAECSVDIAGTDQMQFDK + 2 Oxidation (M)
☒	8364	641.9996	4486.9463	4487.2810	-0.3348	0	16	7.6	1	FFALHFLLPFMIAGLTLIHLTFLHETGSNNPLGVSSNCDK + Oxidation (M)
☒	2573	414.7605	827.5064	826.4773	1.0291	1	16	72	1	SRAAGLPR
☒	3729	705.2711	1408.5276	1408.6259	-0.0982	0	16	54	1	YNPDPYGAGAAGEK
☒	4368	519.8973	1556.6701	1555.7785	0.8915	1	16	57	1	SMTRPRLMFMVR + 2 Oxidation (M)
☒	5173	628.3680	1882.0822	1881.0108	1.0714	1	16	44	1	SLFATRQTLLDLNYAR
☒	4578	407.6923	1626.7401	1627.8464	-1.1063	1	16	57	1	QMADNGRLQPLLTR + Oxidation (M)
☒	5319	991.6645	1981.3144	1980.9000	0.4145	1	16	32	1	QDLFSMPDPEQRFAER + Oxidation (M)
☒	2615	436.7721	871.5296	872.5080	-0.9783	1	16	91	1	GSVEVVKR
☒	3643	689.7006	1377.3866	1376.7194	0.6672	1	16	44	1	GNRVIAVGTTSMR + Oxidation (M)
☒	4690	552.9945	1655.9617	1655.8155	0.1462	0	16	53	1	GLANAGSDVSFGTYGLK
☒	5321	661.5733	1981.6981	1981.0017	0.6964	1	16	33	1	SLSEARHSGALSWLGPDAK
☒	2323	656.3510	655.3437	656.3969	-1.0532	0	16	2.4	1	AAIAGVR
☒	3579	453.5453	1357.6141	1356.7361	0.8780	0	16	67	1	QNQLAATLASNVK
☒	3598	454.8936	1361.6590	1362.8095	-1.1506	1	16	67	1	IGLHAVAQSRALK
☒	6185	644.0388	2572.1261	2572.1938	-0.0677	1	16	30	1	MLNELDSVVRASNSCNLVFEFE
☒	2999	526.7849	1051.5552	1051.5121	0.0432	0	16	66	1	FGGGSGQMIAK
☒	4235	759.8742	1517.7338	1516.8031	0.9307	0	16	62	1	SLTLRPEGTAAMVR + Oxidation (M)
☒	6382	534.0195	2665.0611	2664.3467	0.7144	0	16	24	1	VPVQIHNGVLPSPSSSSSSSSAAATAAR
☒	3592	681.8364	1361.6582	1361.6761	-0.0179	0	16	67	1	HLSTILECAYR
☒	3692	699.1865	1396.3584	1395.6374	0.7210	0	16	43	1	DVIIAMGACTDSK + Oxidation (M)
☒	8299	744.0189	4458.0697	4457.2738	0.7959	1	16	9.4	1	YNHMLSATVKIIQMLQHFEHLAPVLVAAVSLWATDYGMK + 2 Oxidation (M)
☒	3382	641.5373	1281.0600	1280.7340	0.3261	0	16	43	1	ALPLLEEVLER
☒	3586	681.4565	1360.8984	1361.6761	-0.7777	0	16	57	1	HLSTILECAYR
☒	4072	492.5892	1474.7458	1474.7701	-0.0243	1	16	64	1	VRDEVLIDMLEK + Oxidation (M)
☒	4477	795.3461	1588.6776	1588.5877	0.0899	0	16	57	1	ESSPSPMSACMMDK + 2 Oxidation (M)
☒	4697	555.3008	1662.8806	1662.8035	0.0770	0	16	56	1	MSGLTSVAQAEDLWR
☒	3544	671.8918	1341.7690	1342.8006	-1.0316	0	16	67	1	LGVLMIALTSAVR
☒	5239	641.3166	1920.9280	1919.9411	0.9869	1	16	48	1	MSTLDLNHLADLYDRK + Oxidation (M)
☒	6917	599.9088	2994.5076	2995.5232	-1.0156	1	16	26	1	QFPWAVQTWYDSIGAINKIQDFLQK
☒	4904	438.4969	1749.9585	1749.8832	0.0753	1	16	54	1	HADTAAKLHSLCEAVK
☒	7956	1032.2271	4124.8793	4124.2079	0.6713	1	16	11	1	NFLLTVTLICIVVAGVGGQSPISSPTKSPTTPSAPTTSPTK
☒	2343	671.8342	670.8269	670.4126	0.4143	0	16	4.4	1	VALGGVR
☒	4340	516.6077	1546.8013	1545.8878	0.9134	1	16	63	1	LKHLVDEPQNLIK
☒	3287	623.6821	1245.3496	1244.6109	0.7387	1	16	51	1	SEGLGDEVQRR
☒	5289	489.4966	1953.9573	1952.9513	1.0060	0	16	48	1	FQLVNDVMDELITSSAR + Oxidation (M)
☒	4122	494.9344	1481.7814	1481.7838	-0.0025	0	16	61	1	TDVHEIVLVGGSTR
☒	6053	631.7526	2522.9813	2524.1726	-1.1913	1	16	25	1	ENIYGAQFHPEKSGDIGMQMLK + 2 Oxidation (M)
☒	3232	593.3614	1184.7082	1185.6717	-0.9635	1	16	75	1	GNQTLIKLGDK
☒	5869	489.2240	2441.0836	2441.2537	-0.1701	1	16	34	1	EELEAVEVDGVKAVDALQELSAK
☒	6422	667.5852	2666.3117	2666.2729	0.0388	0	16	33	1	QFLNYTLTGVGGFMAASMLMPMVR + 2 Oxidation (M)
☒	3821	712.8520	1423.6894	1422.6674	1.0221	1	16	65	1	EAHSKHAQEVCK
☒	2371	689.6694	688.6621	688.3504	0.3117	0	16	9.2	1	DAATGVR
☒	5298	491.4897	1961.9297	1960.8870	1.0427	0	16	49	1	QHIGNTLGSMIEEEMEK + Oxidation (M)
☒	5178	628.7025	1883.0857	1882.9645	0.1212	1	16	47	1	FSLKTVLMTADQMLNR + Oxidation (M)
☒	5880	616.7856	2463.1133	2463.2694	-0.1561	0	16	35	1	FYFLTGFGAMLQLGMLQLAAQK + Oxidation (M)
☒	8397	753.5198	4515.0751	4514.1644	0.9108	1	16	9.1	1	SGQMMLAQGLLLHLMPTDWRWSDCHALTDVDFEVLKPR + 3 Oxidation (M)
☒	4935	591.2125	1770.6157	1769.9709	0.6447	1	16	39	1	ELLVDMIAVQDLQKR
☒	8580	771.8326	4624.9519	4626.1242	-1.1723	0	16	6.7	1	ITGTGCMLSAMTAAYISANVDSPLEATLASVCAMGICGELAYNR + Oxidation (M)
☒	3247	401.1958	1200.5656	1200.6476	-0.0820	1	16	79	1	ERHVVPAHTR
☒	4915	877.1964	1752.3782	1751.8301	0.5482	1	16	36	1	QLHAFVDMKFEESR + Oxidation (M)
☒	3267	610.1821	1218.3496	1219.5040	-1.1543	0	16	53	1	GMENNFAEHR + Oxidation (M)
☒	7252	677.5270	3382.5986	3382.6795	-0.0809	1	16	20	1	MVSRPGDLPALEAGPGSSEGLLGGMSIPAGMTRR + Oxidation (M)
☒	5286	974.9141	1947.8136	1947.9109	-0.0972	1	16	46	1	AADKDNCFATEGPNLVAR
☒	5502	689.3897	2065.1473	2064.0384	1.1089	0	16	46	1	SFPMNVLMQINISNEVTK
☒	7232	838.3643	3349.4281	3348.4485	0.9796	1	16	18	1	NEDYPEGIGMVSMEMAGDPRNGYVNPAYTR + Oxidation (M)
☒	3372	425.8823	1274.6251	1273.6779	0.9472	1	16	77	1	GVDPAGAIQRYK
☒	4282	769.4011	1536.7876	1535.7229	1.0647	0	16	60	1	HHEDHHEDILVR
☒	4891	873.4431	1744.8716	1745.9312	-1.0596	1	16	58	1	AGITGLVDVEFDVQKR
☒	4981	602.6038	1804.7896	1804.0611	0.7285	1	16	54	1	QAKSLHIFAPIPLLEK
☒	5320	661.5222	1981.5448	1981.1208	0.4240	0	16	33	1	GLVSGLLNSVTGLLGNLAGGGL
☒	5089	930.8019	1859.5892	1859.9352	-0.3460	1	16	36	1	ENLLVFGRYFQMSTR
☒	4442	524.8989	1571.6749	1572.7532	-1.0784	0	16	61	1	GDPTWVQSTIANER
☒	7630	948.7239	3790.8665	3791.9880	-1.1215	1	16	17	1	MATSTETISSLAQPFVHLENPINSPLVKETIRPR + Oxidation (M)
☒	6459	445.5587	2667.3085	2667.3465	-0.0380	1	16	33	1	MSSFVQELDKAFEGQLQVLLEEK
☒	6970	600.5098	2997.5126	2997.4457	0.0669	0	16	27	1	TITVMNSDTASEVINMSLQMLGITGSER
☒	6202	647.7867	2587.1177	2586.2642	0.8535	1	16	32	1	AACNCLKNAAAGVSGLNAGNAASIPSK
☒	6337	880.8121	2639.4145	2638.3101	1.1043	1	16	33	1	LGGSSAFVGKFGDDEFGHMLVDILK
☒	5224	637.5924	1909.7554	1909.9533	-0.1980	1	16	43	1	LNFSNVEEREIYLER
☒	5258	484.2437	1932.9457	1933.9424	-0.9967	1	16	52	1	MAAIGGDGRMPMGVAIEVK + 2 Oxidation (M)
☒	7322	869.6503	3474.5721	3475.6485	-1.0764	1	16	19	1	RGSSLEEMTGVSAGVEGSQQATPTLSAAPAGEAGTR + Oxidation (M)
☒	6449	534.2703	2666.3151	2667.3465	-1.0314	1	16	34	1	MSSFVQELDKAFEGQLQVLLEEK
☒	4740	421.9479	1683.7625	1683.7199	0.0426	0	16	59	1	LSYGEDLQMDWDGR
☒	6319	877.0535	2628.1387	2629.1357	-0.9971	1	16	31	1	NNMNAGGNGSNSNTNKSMPEPLGHR + 2 Oxidation (M)
☒	6432	534.2697	2666.3121	2667.3465	-1.0344	1	16	34	1	MSSFVQELDKAFEGQLQVLLEEK
☒	6857	588.9023	2939.4751	2939.5037	-0.0286	0	16	29	1	VVDAMMPVDQYIGGIEHAILHLLYSR
☒	5912	825.0699	2472.1879	2471.2696	0.9183	1	16	38	1	DASIEAYNKPGSEKFVFLLSTR
☒	4487	398.9358	1591.7141	1592.8158	-1.1017	1	16	61	1	KTAYAAGLAAQNASEK
☒	5638	1102.7644	2203.5142	2204.1153	-0.6011	1	16	30	1	LYYYNNITFKDVAGLEGPK
☒	7233	671.0959	3350.4431	3349.5774	0.8657	1	16	19	1	EMFNDIMPIEDFAGKLSLEYVDYSLGEPK
☒	5575	717.0237	2148.0493	2148.0231	0.0262	1	16	45	1	MSGTNTEKEQMLLVNFFK + 2 Oxidation (M)
☒	7059	616.4909	3077.4181	3076.6664	0.7517	1	16	26	1	GMAPAAMINLKSEPIVAVGAIISDIPLVDR + Oxidation (M)
☒	8584	927.8267	4634.0971	4635.2486	-1.1515	1	16	8.4	1	SNPGPGANGNNGPLRSVVAMSANTPQVMVVTSDGNFYVFNIDLSK
☒	3835	714.7468	1427.4790	1428.6707	-1.1917	0	16	50	1	TDNSPIGMLYFR + Oxidation (M)
☒	4083	493.5580	1477.6522	1478.8457	-1.1935	0	16	65	1	ELLVSAGVALGPTPR
☒	4831	860.0148	1718.0150	1718.8410	-0.8259	0	16	55	1	LTSINQLHSFMEQR + Oxidation (M)
☒	3347	629.2827	1256.5508	1256.6725	-0.1216	0	16	79	1	ALEGQDLSAVVR
☒	5827	482.6145	2408.0361	2408.2120	-0.1759	1	16	37	1	VYGFLESGNYGMVKTGMIVLSK + Oxidation (M)
☒	5489	687.2343	2058.6811	2059.0013	-0.3202	1	16	33	1	DAMLGPAPEVGMGRMLVAGR + 2 Oxidation (M)
☒	7213	555.6042	3327.5815	3326.5033	1.0782	1	16	22	1	KIVMFLDYDGTLSPIVDDPDSAFMSDTMR + 3 Oxidation (M)
☒	2560	809.7505	808.7432	809.4395	-0.6963	0	16	31	1	QLGLDHK

☒	2530	795.6356	794.6283	794.4763	0.1521	0	16	30	1	AHSLLVR
☒	2988	523.6387	1045.2628	1044.5386	0.7243	0	16	65	1	AMQAAGLEVR
☒	3280	619.8666	1237.7186	1238.6619	-0.9432	0	16	72	1	SLPPPSSQTAVR
☒	3231	593.3612	1184.7078	1184.7129	-0.0050	1	16	81	1	GTQGKIVELLK
☒	5537	703.3492	2107.0258	2106.1181	0.9076	1	16	49	1	ALQAKNGGTITPSQTQINHK
☒	6770	577.2885	2881.4061	2882.3401	-0.9340	1	16	32	1	IAIVGMAGRGPGCDNVDEFWDLIMSK + 2 Oxidation (M)
☒	7514	910.4742	3637.8677	3637.8219	0.0458	1	16	18	1	TLGCYPLTGAVESTANTLPEVIQEMLLTKTSER + Oxidation (M)
☒	3990	723.5008	1444.9870	1444.7521	0.2349	0	16	53	1	LSLNISAESAVEGR
☒	5006	453.7557	1810.9937	1809.8911	1.1026	1	16	56	1	QPIYQRTSTYGHFGR
☒	5963	831.7620	2492.2642	2491.2570	1.0072	0	16	39	1	GLVLIAFSQYLQQCPFDEHVK
☒	3723	704.3400	1406.6654	1405.8041	0.8613	1	16	72	1	DIDIHQIKIGVR
☒	6091	844.7389	2531.1949	2530.2811	0.9138	1	16	38	1	VEPGEPFEIEGLAVGLIRNTMLM + Oxidation (M)
☒	6265	436.1979	2611.1437	2612.1860	-1.0423	1	16	33	1	QVTGINGNYGGMAQVYGREGEPCR
☒	5049	612.9261	1835.7565	1836.0430	-0.2866	0	16	50	1	CVPLELTSIPKPLEIK
☒	4128	494.9353	1481.7841	1481.7838	0.0002	0	16	66	1	TDVHEIVLVGGSTR
☒	4438	524.5660	1570.6762	1569.9031	0.7730	1	16	64	1	LGVTGNIKIHPFFK
☒	5140	936.3887	1870.7628	1870.9247	-0.1619	1	16	48	1	LTSSQMASYWADLVKR + Oxidation (M)
☒	4552	405.2431	1616.9433	1616.8807	0.0626	1	16	62	1	MGPKITIDSATLLNK + Oxidation (M)
☒	4560	540.8884	1619.6434	1620.7644	-1.1211	1	16	57	1	QPKAGQHTGYEYSR
☒	6033	838.7296	2513.1670	2513.2696	-0.1027	1	16	38	1	NIMLEIRAGAGGDEASIWAGDLVR
☒	6476	895.4023	2683.1851	2682.3759	0.8092	1	16	32	1	IPLHRQVTVSADSSSSMNSTTPLVR
☒	8076	850.2053	4245.9901	4247.0919	-1.1018	1	16	12	1	VHEMLPCTILGAKPEPKVVTLGNDYDYVWDISAAAGSSK + Oxidation (M)
☒	3142	571.8594	1141.7042	1142.5091	-0.8049	0	16	81	1	DEYSTSSNIK
☒	3596	454.8936	1361.6590	1361.6761	-0.0172	0	16	75	1	HLSTILECAYR
☒	5647	738.3362	2211.9868	2213.0596	-1.0728	0	16	44	1	IDASVTMMQVEEKPDITYK + Oxidation (M)
☒	6475	895.3993	2683.1761	2682.3759	0.8002	1	16	32	1	IPLHRQVTVSADSSSSMNSTTPLVR
☒	5225	956.3854	1910.7562	1910.8680	-0.1117	0	16	44	1	MEVLEEPAPGPGGADAAER + Oxidation (M)
☒	7600	621.8178	3724.8631	3723.9472	0.9159	1	16	18	1	RNPTVITLYELAQALGVSHVELVTPTDETPFGTR
☒	3350	629.8188	1257.6230	1258.6265	-1.0035	1	16	83	1	EERAGEVLSNR
☒	7623	1254.9464	3761.8174	3762.8258	-1.0084	1	16	18	1	KSINEYHDTVSTNSSALTETAQDISMAAPAAPVLSK + Oxidation (M)
☒	8362	898.3956	4486.9416	4486.1937	0.7479	1	16	8.7	1	MTVVTITPGAMSFADWRAIYEGASAALTVGAWDAIDASAAAVAR + 2 Oxidation (M)
☒	4512	532.9313	1595.7721	1596.8121	-1.0400	1	16	65	1	RSASNGHVPGTPVYR
☒	2775	492.7885	983.5624	984.6080	-1.0455	1	16	82	1	LAAGASVLKR
☒	6219	651.5330	2602.1029	2602.2796	-0.1767	1	16	33	1	LSENKLDLVDAMVIAADPTATDER + Oxidation (M)
☒	3237	597.8529	1193.6912	1193.5425	0.1488	1	16	76	1	FEENTADGRR
☒	6793	725.0724	2896.2605	2896.5159	-0.2554	1	16	28	1	MTGNLRTFILMAALTALVMGMGGLIGGR + 2 Oxidation (M)
☒	8227	881.0162	4400.0446	4399.8871	0.1575	0	16	11	1	VFDWSNQMTGYDDPELDIDPQAASMEILGYAYQMADER + Oxidation (M)
☒	2416	359.8079	717.6012	718.3497	-0.7485	0	15	22	1	GEATDVK
☒	2494	385.7216	769.4286	769.4334	-0.0047	0	15	36	1	EVIPNAK
☒	5378	404.8096	2019.0116	2020.0048	-0.9931	0	15	53	1	EAGQNIVIYEGGVVMAQAR + Oxidation (M)
☒	5850	811.3647	2431.0723	2431.2814	-0.2092	1	15	39	1	LIKEEGLLCGGSSGSAMVGALLAAK
☒	5886	493.8319	2464.1231	2465.1823	-1.0592	0	15	40	1	STPFSIPSHHSDTPTTLTSHSTK
☒	4353	776.0073	1550.0000	1550.7828	-0.7827	0	15	54	1	SGSGELSIYATELPK
☒	6197	518.2305	2586.1161	2585.3458	0.7703	1	15	35	1	LSMIPSGTFVHNVEMKVGGGGIIAR + Oxidation (M)
☒	2598	426.9680	851.9214	852.3647	-0.4433	0	15	45	1	ESMEVSR + Oxidation (M)
☒	5642	553.2847	2209.1097	2210.0130	-0.9033	1	15	47	1	SGMSSASLCMENTLDPKQVR
☒	2488	384.2342	766.4538	766.4086	0.0453	0	15	21	1	GAHIDVR
☒	4137	743.7409	1485.4672	1484.8351	0.6321	0	15	51	1	VEQFPVASLLINR
☒	4735	842.4730	1682.9314	1683.8791	-0.9477	1	15	62	1	KIQQGETVELPTGER
☒	5033	365.6060	1822.9936	1822.9321	0.0615	1	15	60	1	MLDTRLFDCPLTITK
☒	6026	838.3995	2512.1767	2512.2301	-0.0534	1	15	40	1	ISGLSENVSEMGYKAALGAIEMAR + Oxidation (M)
☒	7390	884.6582	3534.6037	3535.5456	-0.9419	0	15	20	1	ATMAHANTSHPLYVAGMAGNTNHMSPWGEDAHK + 2 Oxidation (M)
☒	3534	669.5402	1337.0658	1336.6405	0.4254	1	15	50	1	SSANMDQITAKR + Oxidation (M)
☒	6360	531.0287	2650.1071	2649.2129	0.8942	1	15	30	1	SWADAEKFCTEQANGGHLVSIDSK
☒	6716	717.8603	2867.4121	2867.2531	0.1590	0	15	34	1	MFYGIFGDSNGDSPQVTGEASWLMAR + 2 Oxidation (M)
☒	4521	800.7780	1599.5414	1598.9396	0.6019	1	15	48	1	DEIFAPVLSIIRVK
☒	4153	373.9344	1491.7085	1490.7552	0.9533	0	15	72	1	HDLSFLSTVMLGR + Oxidation (M)
☒	3607	1362.6696	1361.6623	1361.6761	-0.0138	0	15	79	1	HLSTILECAYR
☒	4742	842.8887	1683.7628	1683.9481	-0.1852	1	15	65	1	GDDITMVLILPKLEK
☒	2725	467.2993	932.5840	931.4545	1.1295	1	15	1e+02	1	HSAGKMSAK + Oxidation (M)
☒	3650	691.0851	1380.1556	1380.7401	-0.5845	1	15	50	1	FLGSEDGLKLFR
☒	6806	728.5667	2910.2377	2911.2090	-0.9713	0	15	27	1	MDDPGHDNEFPDLSSFDDESSVISTR
☒	7216	833.9478	3331.7621	3330.6918	1.0703	1	15	24	1	KPKIVEQQHVKPVDLSYMNLSQNTDFEK + Oxidation (M)
☒	6796	581.2804	2901.3656	2901.5851	-0.2195	1	15	32	1	EAGLIDLHAGWAKIGIVPLLETTDELK
☒	3374	638.6786	1275.3426	1275.6823	-0.3396	1	15	57	1	EFAKEVDGLLR
☒	6349	442.3937	2648.3185	2647.4441	0.8745	1	15	38	1	LQNINLMSGGEKGLSAIALLFAMLK + Oxidation (M)
☒	3062	368.1875	1101.5407	1102.5692	-1.0286	0	15	96	1	SGIVEGGMLPK + Oxidation (M)
☒	3595	681.8366	1361.6586	1361.6761	-0.0175	0	15	79	1	HLSTILECAYR
☒	5485	515.2950	2057.1509	2057.0728	0.0781	1	15	49	1	FARMVTALGGPADLIDAPAR + Oxidation (M)
☒	6032	838.7278	2513.1616	2512.0819	1.0797	0	15	40	1	TSPYDYYQYWINTDDQDVAR
☒	4526	802.5423	1603.0700	1602.8399	0.2301	0	15	50	1	SNLELMQTIIQAAR + Oxidation (M)
☒	3233	593.7296	1185.4446	1185.5850	-0.1404	1	15	80	1	RAQAAQEEQR
☒	6427	534.2697	2666.3121	2666.2000	0.1122	0	15	38	1	CSHGMVEANGLIYVCGGSLGNNVSR + Oxidation (M)
☒	2596	426.6936	851.3726	850.3855	0.9872	0	15	73	1	TLDAEMR + Oxidation (M)
☒	3136	381.5751	1141.7035	1140.6139	1.0896	0	15	86	1	EVDLVPLGGSR
☒	4218	757.8946	1513.7746	1513.7810	-0.0064	1	15	74	1	TVHEMEKTLGELK
☒	5654	555.7859	2219.1145	2220.1426	-1.0281	1	15	48	1	WLAQGTIYPDVIESAGSKTGK
☒	6175	857.7335	2570.1787	2571.3077	-1.1290	0	15	39	1	YVTVMFTGVQNLMDTLELGIGVR + Oxidation (M)
☒	8408	1129.7792	4515.0877	4514.1644	0.9233	1	15	11	1	SGQMMLAQGLLLHLMPTDWRWSDCHALTDVDFEVLKPR + 3 Oxidation (M)
☒	2545	401.2604	800.5062	799.4552	1.0511	1	15	95	1	TPKGEIR
☒	4344	516.9275	1547.7607	1547.8447	-0.0840	1	15	72	1	DKSFLDLVIELEK
☒	6714	574.4896	2867.4116	2868.3820	-0.9704	1	15	35	1	LVGQAIETFNMIEDGDRLMVCLSGGK + Oxidation (M)
☒	6726	718.1090	2868.4069	2867.2531	1.1538	0	15	34	1	MFYGIFGDSNGDSPQVTGEASWLMAR + 2 Oxidation (M)
☒	2561	406.2468	810.4790	809.4872	0.9919	1	15	45	1	IIKSHGR
☒	5721	762.3887	2284.1443	2282.9566	1.1877	0	15	48	1	VVCEEAGESMMVAAGAGNNDTR + Oxidation (M)
☒	8024	841.7694	4203.8106	4203.9010	-0.0903	1	15	12	1	TTDGAQTSRWAAMQIGMSFISAYAMCAGEAAVADLSFAAK + 3 Oxidation (M)
☒	2749	481.2400	960.4654	959.4535	1.0120	0	15	1.1e+02	1	TIWEHMK + Oxidation (M)
☒	4469	529.2328	1584.6766	1584.7380	-0.0614	0	15	66	1	SLQGSHQAEPETSSK
☒	6410	445.3924	2666.3107	2667.3465	-1.0358	1	15	38	1	MSSFVQELDKAFEGQLQVLLEEK
☒	8228	881.2139	4401.0331	4400.0679	0.9652	0	15	12	1	VVYDELMSLLENMTNMCTLLMLEANSQPEPMIGPILDK + 3 Oxidation (M)
☒	3283	622.4830	1242.9514	1242.6390	0.3124	1	15	59	1	AGNMPKDILER
☒	5419	677.3639	2029.0699	2027.9688	1.1011	1	15	54	1	DIFSNDLKSEQLDEFTK
☒	6984	751.3516	3001.3773	3001.3732	0.0041	1	15	30	1	CCSESLVNRRPCFSGLEVDETYVPK
☒	3368	425.1952	1272.5638	1272.6496	-0.0858	1	15	89	1	KILEVSGCDPR
☒	3601	454.8937	1361.6593	1360.7285	0.9308	1	15	81	1	MFLNLLDAPRR + Oxidation (M)
☒	5318	496.1822	1980.6997	1980.9752	-0.2755	1	15	40	1	AEERDAEGTEALIAELHK
☒	4903	584.3262	1749.9568	1748.8064	1.1503	1	15	65	1	GASKSGDAEETEPSVGTK
☒	6430	534.2697	2666.3121	2667.3465	-1.0344	1	15	39	1	MSSFVQELDKAFEGQLQVLLEEK
☒	4589	543.5882	1627.7428	1626.8577	0.8851	1	15	70	1	QIEEDLRNAGLLEK
☒	3437	650.9859	1299.9572	1300.6735	-0.7162	1	15	59	1	SQQEINADIKR

☒	5767	470.4497	2347.2121	2346.2552	0.9570	1	15	47	1	STLLMLVSALAGHDLIMRAYR + Oxidation (M)
☒	5942	623.0432	2488.1437	2487.3367	0.8070	1	15	41	1	MSINTLIGNLETVPVTTRGATVGK + Oxidation (M)
☒	7022	757.5947	3026.3497	3027.4654	-1.1157	1	15	28	1	HYDELNTGMREGLPMDLARPLNVSQR + Oxidation (M)
☒	6183	515.2328	2571.1276	2570.3890	0.7386	1	15	37	1	GIEAPKQAAVLGAGIMGGGIAYQSALK
☒	6326	526.8361	2629.1441	2629.2581	-0.1140	1	15	36	1	HPGEFSMATLGVEITEKAELDDPK + Oxidation (M)
☒	3514	662.7874	1323.5602	1322.6918	0.8685	0	15	76	1	NGMVLKPHFHK + Oxidation (M)
☒	7026	1013.0956	3036.2650	3035.3820	0.8830	0	15	25	1	ISEGRPNIMDLMMNGEISMAINTSDHK + 2 Oxidation (M)
☒	7904	1021.7742	4083.0677	4083.1128	-0.0451	1	15	15	1	EDAAAMGKTMTVFLGSVAAISLLVGGIGVMNIMLVSVTER + 2 Oxidation (M)
☒	5256	645.2790	1932.8152	1931.9485	0.8667	1	15	54	1	MKFTNILESSFLMNNK + Oxidation (M)
☒	3398	643.1331	1284.2516	1283.7211	0.5306	0	15	58	1	HHERPLLQVR
☒	5180	377.8098	1884.0126	1883.9703	0.0423	1	15	60	1	NFTITDLMKLPDIGYK + Oxidation (M)
☒	5323	992.4598	1982.9050	1984.0663	-1.1612	0	15	57	1	MAATAAAALAVTDELALPLR + Oxidation (M)
☒	4983	906.0019	1809.9892	1809.8719	0.1173	1	15	63	1	TKEMNDAAVFGHALYK + Oxidation (M)
☒	5329	994.4188	1986.8230	1985.8459	0.9772	1	15	50	1	DAEDAMDFVATDLMRDR + Oxidation (M)
☒	5506	1036.9396	2071.8646	2071.0152	0.8495	1	15	48	1	IRLVYLDEMGGMAISMPK + 3 Oxidation (M)
☒	4196	503.2523	1506.7351	1506.7388	-0.0037	0	15	75	1	FPNMSAGAVDLLEK + Oxidation (M)
☒	6350	663.2812	2649.0957	2648.2686	0.8270	1	15	32	1	NRMFSHGTLMLNSDLDEVQNALK + Oxidation (M)
☒	6521	545.0522	2720.2246	2719.3462	0.8785	1	15	36	1	ELSRMNFEIHPIGAVVPLMESYR + 2 Oxidation (M)
☒	6232	435.8885	2609.2873	2608.1819	1.1055	1	15	40	1	ASENAEVDIPQAMVDTELDRMMK + Oxidation (M)
☒	7123	799.8597	3195.4097	3195.6684	-0.2587	1	15	25	1	LGFRPPPRNGVTTVLSRPGTAVTMQNNSGR + Oxidation (M)
☒	4134	742.8894	1483.7642	1484.7583	-0.9941	0	15	76	1	QQLGLSPQDSLGSR
☒	4748	562.5914	1684.7524	1683.7199	1.0325	0	15	69	1	LSYGEDLQMDWDGR
☒	4763	846.4093	1690.8040	1690.8638	-0.0598	1	15	68	1	KPTVVSYQENDRQK
☒	4066	492.5890	1474.7452	1474.7887	-0.0436	1	15	80	1	MEQIQMVKVLEK
☒	3266	610.1819	1218.3492	1218.6581	-0.3089	1	15	64	1	EVNPRLAHQR
☒	5914	825.3897	2473.1473	2472.3046	0.8427	1	15	43	1	MYSVSIIGASGYSGAELTRLLLR + Oxidation (M)
☒	6164	512.2328	2556.1276	2556.3846	-0.2570	1	15	39	1	SLFSRHGIAVGSTGNLGLSIGIMAAK
☒	3012	528.7495	1055.4844	1056.5498	-1.0654	1	15	93	1	MSGAPGRLPR + Oxidation (M)
☒	6205	647.7871	2587.1193	2588.2185	-1.0992	1	15	37	1	IGCGACEPEVKMAGGQAAAALPTWK + Oxidation (M)
☒	2735	472.2861	942.5576	941.5182	1.0394	0	15	1.1e+02	1	GPQATGVTLV
☒	3065	553.8278	1105.6410	1104.5411	1.1000	1	15	99	1	RAEALSESDK
☒	4275	767.8275	1533.6404	1532.6842	0.9563	0	15	72	1	GDLDLGNLDSEEEK
☒	3034	540.3289	1078.6432	1077.5277	1.1155	0	15	92	1	GPMSAISFPR + Oxidation (M)
☒	3749	473.2375	1416.6907	1416.7653	-0.0746	1	15	85	1	YSYLIVEKGFAK
☒	4439	786.3454	1570.6762	1571.7362	-1.0599	0	15	73	1	ALGNSEEATMLQHR + Oxidation (M)
☒	4448	788.0709	1574.1272	1573.8610	0.2663	1	15	52	1	SSLLRSMNDALIVR
☒	6234	435.8886	2609.2879	2608.1819	1.1061	1	15	41	1	ASENAEVDIPQAMVDTELDRMMK + Oxidation (M)
☒	3220	394.2234	1179.6484	1180.5975	-0.9492	1	15	86	1	GDKDIETYLK
☒	3491	657.4491	1312.8836	1313.6939	-0.8102	0	15	73	1	IAEAEIANLSGAR
☒	4286	769.9031	1537.7916	1536.7208	1.0708	0	15	73	1	LWSNENFEINSGK
☒	5345	501.2952	2001.1517	1999.9613	1.1904	1	15	54	1	HSHVRNGQPTPPPYGDNK
☒	4446	787.4600	1572.9054	1573.8610	-0.9555	1	15	74	1	SAILRSMNDALIVR + Oxidation (M)
☒	5330	398.4149	1987.0381	1987.9707	-0.9326	0	15	58	1	INLEGLACETVPTPMATR + Oxidation (M)
☒	4060	738.1277	1474.2408	1473.8164	0.4244	1	15	54	1	ATEALRHHLASIR
☒	6334	880.0414	2637.1024	2638.0959	-0.9935	1	15	34	1	QHVDPDETGGNDSYCNLMMQRR + Oxidation (M)
☒	4130	371.4533	1481.7841	1482.7427	-0.9586	1	15	76	1	SVHGEGEDSKVAIR
☒	5429	510.0087	2036.0057	2034.9792	1.0265	1	15	58	1	EAMEEQIKAANLQNQFR + Oxidation (M)
☒	5816	602.3157	2405.2337	2406.0654	-0.8317	1	15	48	1	EIDALGGEMGKFADACMIQYR + 2 Oxidation (M)
☒	5889	1234.1150	2466.2154	2467.2668	-1.0514	0	15	46	1	IGLVSQEPVLFSSSIMENIGYGK
☒	7368	879.4176	3513.6413	3514.7146	-1.0733	1	15	23	1	DIMEKLSPIGPVYQAGTMSGNPVVMAAGYTTLR + 3 Oxidation (M)
☒	5492	687.9756	2060.9050	2061.1106	-0.2057	1	15	54	1	TVTFTPVSTANIKIPDTTR
☒	5548	707.0216	2118.0430	2117.0252	1.0178	0	15	57	1	HSIPLPVQDGFPTTSCFSK
☒	6849	588.7034	2938.4806	2937.3046	1.1760	0	15	35	1	MSSATANSFTGGNNPNITSNTSSSAASFSK
☒	6378	534.0192	2665.0596	2666.2568	-1.1971	1	15	30	1	IVDTPCNEMNTDTFLEEINKVGK
☒	6507	542.6320	2708.1236	2709.3028	-1.1792	1	15	33	1	DGASATEGFCADGVRIGSLLVSSEGVR
☒	6194	647.0334	2584.1045	2583.4860	0.6185	1	15	38	1	TKPTQHSVAELRGIGILPDALVLR
☒	5300	491.7404	1962.9325	1963.9599	-1.0274	0	15	60	1	NPELEQASESGGLVQGPPR
☒	7173	657.0889	3280.4081	3279.5470	0.8611	1	15	25	1	YNLNYIKMEGGIGCMVNGAGLAMATMDIIK + 2 Oxidation (M)
☒	6277	523.4368	2612.1476	2611.2986	0.8490	1	15	38	1	QGQGVVTIGAMAKGDSEFMASTVLAK + Oxidation (M)
☒	3553	672.9553	1343.8960	1342.7204	1.1756	1	15	76	1	DALDTALAAAKQR
☒	5535	702.2452	2103.7138	2103.0419	0.6719	1	15	39	1	DATSKVALVYGQMNEPPGAR
☒	2414	357.7401	713.4656	713.4436	0.0221	0	15	23	1	LAGNVLK
☒	6221	652.3260	2605.2749	2604.3329	0.9420	1	15	43	1	IGIASKALGISQAALDCAVNYAENR
☒	6805	728.3016	2909.1773	2909.5222	-0.3449	1	15	27	1	FRLTPTPFHTTSHMEALVEALLAVGR + Oxidation (M)
☒	2575	417.1430	832.2714	833.3888	-1.1173	0	15	1.1e+02	1	GMPMNIR + Oxidation (M)
☒	5498	517.0419	2064.1385	2064.0384	0.1001	0	15	57	1	SFPMNVLMQINISNEVTK
☒	3146	572.7280	1143.4414	1142.4815	0.9600	0	15	95	1	CPTEYFGNR
☒	4513	399.9503	1595.7721	1596.8222	-1.0501	0	15	75	1	FGFSSVMQVPLIEK + Oxidation (M)
☒	6464	534.6679	2668.3031	2669.2697	-0.9666	0	15	42	1	VVVQGIWVGGWCWGEDVPNVEER
☒	6941	600.1089	2995.5081	2995.5232	-0.0151	1	15	34	1	QFPWAVQTWYDSIGAINKIQDFLQK
☒	5255	644.9863	1931.9371	1932.9550	-1.0179	0	15	64	1	GLPFVGSMSLMSNTLAHR + Oxidation (M)
☒	6365	663.7800	2651.0909	2650.5536	0.5373	1	15	33	1	VAVLFAVALFAYLLDLGSKMLVVAK
☒	4907	584.3268	1749.9586	1749.8971	0.0614	0	15	71	1	MTTPDLTGALFEIVAR + Oxidation (M)
☒	5819	602.3168	2405.2381	2404.3359	0.9022	1	15	49	1	LGASLGSLSSGRISIISMSVVNLK + Oxidation (M)
☒	6370	531.2288	2651.1076	2650.3562	0.7514	1	15	35	1	ATGTSTINTGANGLQRLDYVISAAEK
☒	8197	1091.2670	4361.0389	4362.0786	-1.0397	0	15	13	1	FYNESPMEDYTLATTLLRPAPVMAFIGIMDIPGAPETDK + 3 Oxidation (M)
☒	7067	1036.4628	3106.3666	3105.4785	0.8880	1	15	29	1	EVAVSHKPDAFLRESDMGSNVTSNSSSVR
☒	3434	650.2887	1298.5628	1298.6731	-0.1103	0	15	89	1	DAGLLVHQAGYR
☒	6325	658.2926	2629.1413	2629.3976	-0.2563	0	15	39	1	TLADLGYAQGVIPPHERPNIEALR
☒	2577	421.2578	840.5010	840.4566	0.0445	1	15	78	1	TPGAPSRR
☒	6795	581.0802	2900.3646	2899.3950	0.9696	0	15	36	1	HVEVSELVTYFDLVDFDVTNMLNGK + Oxidation (M)
☒	4001	725.5278	1449.0410	1449.7212	-0.6801	0	15	58	1	SLGNVNFSVSAEAR
☒	4501	798.3192	1594.6238	1593.7708	0.8530	1	15	65	1	MTNWTEIDEIAKK + Oxidation (M)
☒	5771	470.8515	2349.2211	2350.1120	-0.8908	0	15	50	1	EQANTVETIVLMAVHCMNFK + Oxidation (M)
☒	4712	557.5933	1669.7581	1669.9919	-0.2338	1	15	74	1	IFEIRPVKWLLEK
☒	5171	628.2614	1881.7624	1882.9458	-1.1835	1	15	56	1	AYIDSVINTCKNGSITK
☒	7573	621.4816	3722.8459	3723.9472	-1.1013	1	15	22	1	RNPTVITLYELAQALGVSHVELVTPTDETPFGTR
☒	2386	704.8072	703.7999	703.3613	0.4386	0	15	12	1	TSGGLNR
☒	3239	399.2118	1194.6136	1195.5552	-0.9416	0	15	88	1	QMLMVMHYK + Oxidation (M)
☒	3676	694.5840	1387.1534	1386.7831	0.3704	0	15	59	1	SGATILGGISIGVSR
☒	5054	613.5916	1837.7530	1837.9210	-0.1680	1	15	60	1	ELQELNDRFASFIEK
☒	7404	708.3244	3536.5856	3536.6843	-0.0987	1	15	23	1	EMNFITENRASVVYELPLAEMVGDFFDQLK + 2 Oxidation (M)
☒	3254	603.4268	1204.8390	1205.6140	-0.7749	0	15	80	1	ADTTTPTTSIAK
☒	6058	632.2791	2525.0873	2524.3021	0.7852	1	15	40	1	VVDSLGNPIDGKGELGNSLTSPVEK
☒	6145	512.0326	2555.1266	2554.0815	1.0452	0	15	41	1	HFSMMILSDDGVVCYNSDYAAK + 2 Oxidation (M)
☒	8328	895.1975	4470.9511	4472.1120	-1.1609	1	15	11	1	MFTVLLDSNASEHAARTMAMQIATDNANDLLQDLTVMYNK + Oxidation (M)
☒	3256	606.3673	1210.7200	1211.6874	-0.9673	0	15	87	1	THLLTALSTQK
☒	6698	574.2892	2866.4096	2865.4708	0.9388	1	15	40	1	LQLAALGYVFPDRAMDHLHALAAGGSR + Oxidation (M)
☒	3817	711.8315	1421.6484	1422.6371	-0.9886	0	15	85	1	GDIEMDAIIMDGK + Oxidation (M)
☒	5884	617.0368	2464.1181	2464.1578	-0.0397	1	15	47	1	EAESGGQSPEHPQESTTQRPGKK
☒	4380	521.9281	1562.7625	1562.7260	0.0365	1	15	80	1	RKPAYMGPDGNEGR + Oxidation (M)

☒	4467	791.8826	1581.7506	1580.8167	0.9340	1	15	76	1	MFRIVSMEGIINR + Oxidation (M)
☒	3004	528.2483	1054.4820	1053.4947	0.9874	1	15	95	1	MTNLEKMR + 2 Oxidation (M)
☒	6534	547.4421	2732.1741	2731.2768	0.8973	1	15	35	1	DTSRSPLFSVMFNMQNMEIPSLR + 2 Oxidation (M)
☒	6261	523.2354	2611.1406	2612.1860	-1.0454	1	15	40	1	QVTGINGNYGGMAQVYGREGEPCR
☒	6421	534.2696	2666.3116	2665.4448	0.8668	1	15	43	1	GLFMTTNPVPVKAALGMLGLNVGPPR + Oxidation (M)
☒	3403	643.8607	1285.7068	1284.6674	1.0395	0	15	97	1	VEGADDVVNVIR
☒	5609	730.9753	2189.9041	2191.0790	-1.1750	1	15	49	1	TGLETERLMEVEQVLDSAR + Oxidation (M)
☒	6258	523.0660	2610.2936	2609.3352	0.9584	1	15	44	1	WFLKGYQNANYLSLFLMVATSK + Oxidation (M)
☒	7479	904.8889	3615.5265	3615.7276	-0.2011	0	15	19	1	SPAPTTSAGPASSGIPPSSLPTSTVTEDDTKPSSSSSK
☒	5409	506.2424	2020.9405	2019.9798	0.9607	1	15	61	1	GECDGFTLFKVLFCTVK
☒	6022	1254.5535	2507.0924	2506.2923	0.8002	1	15	43	1	IYREEELVAELLNALAVGMAMR + Oxidation (M)
☒	7681	764.5858	3817.8926	3816.7950	1.0977	0	15	21	1	ETSVAIMLGASAGFINPFSYQCNLMVYAAGNYSVR + Oxidation (M)
☒	6634	564.8693	2819.3101	2819.4640	-0.1539	1	15	39	1	GVIDGIINGVGLAGFCIGEEIKYVGGGR
☒	4757	564.6073	1690.8001	1690.8196	-0.0195	1	15	75	1	EKPNVRTETLDMDK + Oxidation (M)
☒	5177	628.6771	1883.0095	1883.8584	-0.8490	0	15	66	1	HVAGLAGANSTEFDAHCK
☒	6640	943.4295	2827.2667	2828.3983	-1.1316	1	15	38	1	IKHIEATEANLLIGADMGCLMNIGGR + 2 Oxidation (M)
☒	4338	774.2730	1546.5314	1545.6922	0.8392	0	15	62	1	WQTLFFDASMER + Oxidation (M)
☒	5232	640.9824	1919.9254	1918.8870	1.0384	0	15	66	1	ADLYTGEAEYDTIAMIK + Oxidation (M)
☒	7261	678.1277	3385.6021	3385.6460	-0.0438	1	15	26	1	SGHSELSHIDAELNYIAVKTQDELTAAMAQK + Oxidation (M)
☒	7369	879.4230	3513.6629	3514.6748	-1.0120	1	15	25	1	EVIARVTSSGGFPPLMLDPIDFEAMYEQQSR + 2 Oxidation (M)
☒	8337	895.2001	4470.9641	4470.0243	0.9399	1	15	11	1	MLPEFMGGSADLAPSNLTMWSGSKSLEASDFSGNYIHYGVR + 3 Oxidation (M)
☒	3244	599.3759	1196.7372	1197.6652	-0.9279	1	15	93	1	MERPGPVLKR + Oxidation (M)
☒	3059	548.3441	1094.6736	1093.6356	1.0380	1	15	90	1	HTLVNNLRK
☒	5511	691.9985	2072.9737	2073.1140	-0.1403	1	15	60	1	GIMVNGVDEATILDLLTKR + Oxidation (M)
☒	5608	730.8291	2189.4655	2188.9828	0.4827	1	15	40	1	SSSMGNITDTMKSSTIPGSEK + 2 Oxidation (M)
☒	7082	781.6085	3122.4049	3123.3763	-0.9714	0	15	32	1	MIVEGECDALPEQAFYMVGTIDEAFEK + 2 Oxidation (M)
☒	7899	1017.9883	4067.9241	4068.9455	-1.0214	0	15	18	1	VNPMAPDYPVTFNYLELLVDLPWGENSTDNFDLVR + Oxidation (M)
☒	5420	407.0215	2030.0711	2030.9367	-0.8656	1	15	62	1	WQGQMLQKEPEEEQQK + Oxidation (M)
☒	5987	625.3121	2497.2193	2496.2788	0.9405	0	15	49	1	LGPAPLNLEVPPYEFTGDDVVVVG
☒	3590	454.8932	1361.6578	1360.7285	0.9293	1	15	94	1	MFLNLLDAPRR + Oxidation (M)
☒	4998	453.5058	1809.9941	1808.9931	1.0010	1	15	70	1	VGIPAKSGVGGGIMASVPGR
☒	6520	681.0628	2720.2221	2721.2271	-1.0050	1	15	40	1	MFDLTGKNVCYVADCGGIALETCK
☒	4995	453.5056	1809.9933	1809.8535	0.1398	1	15	70	1	MTGRLALVMGAEGEGMR + 2 Oxidation (M)
☒	6028	1257.0966	2512.1786	2512.2672	-0.0885	1	15	49	1	LGVSAVFAPKIGEMYPYEIEQR + Oxidation (M)
☒	6847	490.5881	2937.4849	2937.4753	0.0096	1	15	39	1	SPSGNSVLYNAVVSKMAEVTLQSLDANK + Oxidation (M)
☒	8285	1115.2740	4457.0669	4457.2738	-0.2069	1	15	14	1	YNHMLSATVKIIQMLQHFEHLAPVLVAAVSLWATDYGMK + 2 Oxidation (M)
☒	3700	700.5226	1399.0306	1398.7831	0.2476	1	15	64	1	QKDLTDLALGLGR
☒	6315	526.2645	2626.2861	2626.4186	-0.1325	1	15	44	1	LALLRAVGCVLANGLSLLGVSAPESM + Oxidation (M)
☒	7187	828.1207	3308.4537	3307.7664	0.6873	1	15	26	1	TEIGAGAFIGSNSSLVAPVKIGTGAYVGSGSVITK
☒	6180	515.2315	2571.1211	2570.2210	0.9001	1	15	43	1	KMDDEYQNFLAELGGTVPESSLK
☒	5501	689.3892	2065.1458	2064.0384	1.1074	0	15	61	1	SFPMNVLMQINISNEVTK
☒	6241	522.8654	2609.2906	2609.3352	-0.0446	1	15	46	1	WFLKGYQNANYLSLFLMVATSK + Oxidation (M)
☒	3154	575.8411	1149.6676	1150.6822	-1.0146	1	15	1e+02	1	KPVSLKHSQK
☒	3535	670.0775	1338.1404	1338.7004	-0.5599	1	15	60	1	TNSDEHRILVR
☒	6908	996.1277	2985.3613	2986.4391	-1.0778	1	15	35	1	ISVMGHGMGAPSASIYFHELMTTYKVK + 2 Oxidation (M)
☒	2974	518.7998	1035.5850	1035.4906	0.0944	0	15	1.1e+02	1	GDGAEMSLIK + Oxidation (M)
☒	6400	534.2691	2666.3091	2667.3465	-1.0374	1	15	45	1	MSSFVQELDKAFEGQLQVLLEEK
☒	6672	1428.6409	2855.2672	2854.4265	0.8408	1	15	37	1	KYEENPLHFFMGVWGSAFSILINR
☒	7587	745.7776	3723.8516	3725.0044	-1.1528	1	15	23	1	LLREFTVTLSVAIGISLLVSLTLTPMMCGWMLK + 2 Oxidation (M)
☒	3581	679.8161	1357.6176	1356.5986	1.0190	0	15	96	1	TFDAGEFAGWEK
☒	5655	741.0077	2220.0013	2219.0086	0.9927	0	15	56	1	ELEEEVGLAPADVEVMGCTR + Oxidation (M)
☒	7058	770.3610	3077.4149	3076.5031	0.9118	1	15	34	1	LAPDIITMDINMPDLDGFATTRQIMER
☒	7732	658.1135	3942.6373	3942.9633	-0.3259	1	15	14	1	QDGTQAGEVELADSIFAIEPNEAVVTDAVLMQRASLR
☒	8322	895.1952	4470.9396	4470.1988	0.7408	1	15	12	1	MTVVTITPGAMSFADWRAIYEGASAALTVGAWDAIDASAAAVAR + Oxidation (M)
☒	5867	611.0454	2440.1525	2439.3243	0.8282	1	15	50	1	HFGMVAGGTGITPMLQVIRAIVR + Oxidation (M)
☒	6373	667.0220	2664.0589	2663.2102	0.8487	1	15	34	1	DADGVAMAGDGQVTMGQSVVMKHTAR + 2 Oxidation (M)
☒	3245	599.3766	1196.7386	1197.5924	-0.8538	0	14	96	1	CNLVTIEHGR
☒	4592	408.2226	1628.8613	1627.7096	1.1517	1	14	84	1	CPHQVYHRDGGMR + Oxidation (M)
☒	4676	550.1861	1647.5365	1648.6974	-1.1609	1	14	56	1	DTSHRWMMEPDTK + Oxidation (M)
☒	5413	675.7233	2024.1481	2024.0248	0.1232	1	14	62	1	MNRDIYTQIETVGVLEK + Oxidation (M)
☒	5653	739.3713	2215.0921	2216.2173	-1.1253	1	14	61	1	MRLVIGISGASGVVLGYHMLK + Oxidation (M)
☒	6835	732.3093	2925.2081	2924.5120	0.6961	1	14	32	1	MQDVVTPAGGVWVHWRDYLTLTKPR
☒	4026	730.0646	1458.1146	1457.7799	0.3347	0	14	63	1	EMIQESPILAAIK + Oxidation (M)
☒	6679	952.7697	2855.2873	2854.4573	0.8299	0	14	39	1	VVAALGDSLTTAVGARPNNSSDLPTSWR
☒	5616	549.5052	2193.9917	2194.1919	-0.2002	1	14	57	1	LGSVTTIVVSSADVAKEMFLK
☒	5824	482.4556	2407.2416	2408.1682	-0.9266	0	14	54	1	SLEGWLTNAMSDFPQQVIQTK + Oxidation (M)
☒	6707	574.4894	2867.4106	2868.3820	-0.9714	1	14	42	1	LVGQAIETFNMIEDGDRLMVCLSGGK + Oxidation (M)
☒	7309	691.9341	3454.6341	3455.6957	-1.0616	0	14	27	1	HTSATPSISSTTPASFAQIDSTPPAFAQIPGTNR
☒	7464	899.4162	3593.6357	3593.7238	-0.0881	1	14	26	1	RAKPVLLEPMMAVEVETPEEFMGNVMGDLSSR + 2 Oxidation (M)
☒	5797	788.0558	2361.1456	2362.1735	-1.0279	1	14	56	1	GLGNVTFMKTFAAMLVQVMEK + 3 Oxidation (M)
☒	6214	520.0359	2595.1431	2595.1700	-0.0269	1	14	43	1	GGQIDFVLGHFTDEDWKTMDER
☒	6303	523.6368	2613.1476	2612.1451	1.0025	0	14	44	1	VGNLFSADLFYSPDGDMFDVMEK + Oxidation (M)
☒	6407	534.2694	2666.3106	2665.2150	1.0956	0	14	46	1	NHGNNEADAALQGLAQQGVDMEDLR
☒	6437	534.2698	2666.3126	2667.3465	-1.0339	1	14	46	1	MSSFVQELDKAFEGQLQVLLEEK
☒	7050	612.5199	3057.5631	3057.4457	0.1174	1	14	35	1	NAEAIMESRSMISSMPTQTPSLGVVYDK + Oxidation (M)
☒	7053	766.6022	3062.3797	3063.4542	-1.0745	1	14	33	1	GEACQQFAENLRNLLQLEVVMWDER + Oxidation (M)
☒	4150	373.9342	1491.7077	1490.8569	0.8508	0	14	89	1	LLATEGVAAVHAALR
☒	5053	460.1973	1836.7601	1836.0430	0.7171	0	14	66	1	CVPLELTSIPKPLEIK
☒	8301	892.6237	4458.0821	4458.4782	-0.3960	1	14	14	1	DQLKVPMTSFLILSVFMILMPILLMNLLIGLAVGDIESVR + Oxidation (M)
☒	3588	454.8931	1361.6575	1361.6761	-0.0187	0	14	98	1	HLSTILECAYR
☒	3752	473.5798	1417.7176	1418.7405	-1.0229	1	14	96	1	EIEEVIRFQEK
☒	5918	619.5444	2474.1485	2473.2313	0.9172	0	14	50	1	MFVASVLYLLYLVQFDYMNL + 2 Oxidation (M)
☒	6510	678.0405	2708.1329	2707.2508	0.8821	1	14	38	1	SEQQTCVIDNGSGVVKAGFSGEDAPR
☒	7178	658.1121	3285.5241	3284.6764	0.8477	0	14	30	1	HSLYQSPHLLLLQGYNQQDCLVYLLNR
☒	7592	745.7781	3723.8541	3723.9472	-0.0931	1	14	24	1	RNPTVITLYELAQALGVSHVELVTPTDETPFGTR
☒	3454	652.8841	1303.7536	1302.6126	1.1411	0	14	1e+02	1	DGGTICVVEEPK
☒	4582	814.3848	1626.7550	1625.8373	0.9178	1	14	84	1	STTSSNLSAAFSRLGK
☒	6414	534.2695	2666.3111	2665.4448	0.8663	1	14	46	1	GLFMTTNPVPVKAALGMLGLNVGPPR + Oxidation (M)
☒	6444	534.2700	2666.3136	2667.3465	-1.0329	1	14	46	1	MSSFVQELDKAFEGQLQVLLEEK
☒	2470	756.3409	755.3336	754.3684	0.9653	0	14	41	1	ISFGGMK + Oxidation (M)
☒	4679	551.6033	1651.7881	1651.8352	-0.0471	1	14	81	1	LQKMAIQTTNSDFR
☒	3597	454.8936	1361.6590	1360.7285	0.9305	1	14	98	1	MFLNLLDAPRR + Oxidation (M)
☒	4346	774.9611	1547.9076	1547.8494	0.0583	1	14	85	1	NKFLIIDGMNLVR + Oxidation (M)
☒	5165	627.9768	1880.9086	1879.9138	0.9947	0	14	71	1	RPCFSALTPDETYVPK
☒	6126	851.7334	2552.1784	2553.2839	-1.1055	0	14	48	1	VQWFDMFPHTDHAEVLTLLVR
☒	3496	658.1094	1314.2042	1314.7143	-0.5100	1	14	71	1	ENLTADNKVIAK
☒	4348	775.5147	1549.0148	1549.7567	-0.7419	1	14	67	1	LMAGGKCIEWMVR
☒	6067	506.8461	2529.1941	2528.2511	0.9430	1	14	50	1	DPKPMPPPMKLTSEMVQVMGGVK + 2 Oxidation (M)
☒	3076	559.2787	1116.5428	1117.6053	-1.0624	0	14	1.2e+02	1	DVAEAMLLLK + Oxidation (M)
☒	4534	535.8997	1604.6773	1605.8263	-1.1491	0	14	82	1	AFPPAVPQDAPAAGAAR

☒	6034	1257.5968	2513.1790	2513.2544	-0.0753	1	14	50	1	IEQLGAVRIGDDVEIGANTCIDR
☒	6115	509.4067	2541.9971	2542.2672	-0.2701	1	14	37	1	EHPSMRGGLFELDSVIEMVRPK + Oxidation (M)
☒	6246	522.8657	2609.2921	2609.3352	-0.0431	1	14	48	1	WFLKGYQNANYLSLFLMVATSK + Oxidation (M)
☒	6428	534.2697	2666.3121	2667.3465	-1.0344	1	14	47	1	MSSFVQELDKAFEGQLQVLLEEK
☒	7039	610.6913	3048.4201	3049.4466	-1.0265	1	14	36	1	GVKEPQATFSACFGQAFMTLHPTYYAK
☒	8224	880.8156	4399.0416	4400.0679	-1.0263	0	14	14	1	VVYDELMSLLENMTNMCTLLMLEANSQPEPMIGPILDK + 3 Oxidation (M)
☒	2997	351.1738	1050.4996	1051.5298	-1.0302	0	14	1e+02	1	TGTFAEALSR
☒	5301	491.7407	1962.9337	1963.1036	-0.1699	1	14	69	1	ENALPAGLNKPRLQAMLK
☒	6285	523.4370	2612.1486	2611.2986	0.8500	1	14	44	1	QGQGVVTIGAMAKGDSEFMASTVLAK + Oxidation (M)
☒	2413	357.7396	713.4646	714.4640	-0.9993	0	14	26	1	LTAGLLK
☒	3020	531.8159	1061.6172	1061.5328	0.0845	0	14	1.2e+02	1	GPMAAISFPR + Oxidation (M)
☒	3223	590.8638	1179.7130	1180.6386	-0.9256	1	14	99	1	MKPPAPEQRK
☒	4031	731.1847	1460.3548	1459.8035	0.5514	0	14	65	1	QETFTLTVPIGVR
☒	5910	825.0690	2472.1852	2471.1858	0.9993	0	14	52	1	TGGAAAMCAEMEVPLLGSLPLDPR + Oxidation (M)
☒	7776	797.1557	3980.7421	3980.6863	0.0558	1	14	17	1	NMIQCAGTRTWTSYIGYGCYCGYGGSGTPVDELDR + Oxidation (M)
☒	8507	1136.9994	4543.9685	4544.3424	-0.3739	1	14	11	1	TLMYLCCILMLLAPIMVISSSYTLILHLIHRMNSAAGHR + 2 Oxidation (M)
☒	4893	437.4448	1745.7501	1746.7553	-1.0052	0	14	78	1	ANSVGQNLYMMGSSEK + 2 Oxidation (M)
☒	8294	1115.5234	4458.0645	4457.0654	0.9991	1	14	14	1	FFQERGFVYTNTPIITGSDCEGAGEMFQLTTMDLNNIPK + Oxidation (M)
☒	3452	652.8840	1303.7534	1302.6853	1.0681	0	14	1e+02	1	NEIVAGMAEIIK + Oxidation (M)
☒	2392	709.0741	708.0668	708.3443	-0.2774	0	14	5.5	1	NPPLDGP
☒	2584	423.2732	844.5318	843.4814	1.0504	1	14	1.5e+02	1	GPKSNTLK
☒	3217	588.9054	1175.7962	1176.5622	-0.7660	0	14	1e+02	1	TSDSAETTPLR
☒	3238	399.2112	1194.6118	1195.5552	-0.9434	0	14	98	1	QMLMVMHYK + Oxidation (M)
☒	7162	816.8714	3263.4565	3264.5292	-1.0727	1	14	31	1	SHSVDHLPRATNMLEMVWDDELAEVAQR + Oxidation (M)
☒	8084	1062.5057	4245.9937	4245.1371	0.8566	1	14	17	1	AIVIVALDNLTKGSSGQALQNANLMLGEDETAGLMMAPLFP + 2 Oxidation (M)
☒	4476	795.2761	1588.5376	1587.8369	0.7007	0	14	64	1	GFVHTLNSTAIATTR
☒	6094	634.5295	2534.0889	2534.2864	-0.1975	1	14	45	1	NEELEQQLQLLLLPKDPDDER
☒	3625	457.5488	1369.6246	1370.8020	-1.1775	1	14	98	1	AKLESLVEELIK
☒	4602	817.4462	1632.8778	1631.8995	0.9784	1	14	85	1	SNTIAIFINQVREK
☒	5568	714.7000	2141.0782	2140.2653	0.8129	1	14	63	1	AQNKTIAVALLATLVAVMGK + Oxidation (M)
☒	8350	1491.9941	4472.9605	4472.2243	0.7361	0	14	12	1	AGSNVSMLLSLLAFVSTQNHQTTEEMDNLGLIYDLFSILR + 2 Oxidation (M)
☒	2493	385.7084	769.4022	770.4538	-1.0515	0	14	47	1	VIPVSEK
☒	4913	438.7480	1750.9629	1750.9829	-0.0200	1	14	79	1	GDLGVEIPAEKVVVAQK
☒	6390	445.2255	2665.3093	2664.2815	1.0278	1	14	48	1	MYSKLAGMTGTAVTEAGELWEIYK + Oxidation (M)
☒	6998	1004.5269	3010.5589	3011.5101	-0.9512	1	14	38	1	ARAENDHVIVTIFVNPTQFGPSEDLSR
☒	8123	857.7939	4283.9331	4283.8829	0.0502	1	14	15	1	MRNAVIMEPYMIEIDNSMEQLSFDHDESYLTMLDR + 3 Oxidation (M)
☒	8589	928.0319	4635.1231	4635.2486	-0.1255	1	14	11	1	SNPGPGANGNNGPLRSVVAMSANTPQVMVVTSDGNFYVFNIDLSK
☒	4029	730.6852	1459.3558	1458.7500	0.6058	1	14	66	1	GKDIDAACGQLAIK
☒	5841	808.0604	2421.1594	2420.2257	0.9337	0	14	55	1	ELLSGVASNAEYLDGLMAPVLSR + Oxidation (M)
☒	6090	844.4078	2530.2016	2530.2056	-0.0040	0	14	52	1	MAAMSLAAAAAWATGAAAAADGAPAAQR + Oxidation (M)
☒	3258	608.3086	1214.6026	1213.5939	1.0088	0	14	1.1e+02	1	QPANIDNISDK
☒	6446	534.2701	2666.3141	2666.3084	0.0057	0	14	48	1	NLQLLVLMDNGFFGSIPESLGDCK
☒	8230	881.2163	4401.0451	4400.0679	0.9772	0	14	15	1	VVYDELMSLLENMTNMCTLLMLEANSQPEPMIGPILDK + 3 Oxidation (M)
☒	5296	654.6135	1960.8187	1960.9524	-0.1337	1	14	64	1	LTGLGQKDEADAAAVEAMR + Oxidation (M)
☒	8098	708.8400	4246.9963	4247.2321	-0.2357	1	14	17	1	ELLDVLSVLSLMERNSPVVISPLLMNLTVENDFSTTVK + 2 Oxidation (M)
☒	2426	363.2065	724.3984	725.3820	-0.9836	0	14	11	1	APNPATR
☒	3611	455.2277	1362.6613	1362.7693	-0.1081	0	14	1e+02	1	IVGAMVQIITYR
☒	4524	801.8322	1601.6498	1600.8421	0.8078	1	14	81	1	VTITDGSDKNSPVLR
☒	5040	457.7552	1826.9917	1825.9608	1.0309	1	14	75	1	HVLENPDMISKTVLSK + Oxidation (M)
☒	8485	755.8354	4528.9687	4529.0549	-0.0862	0	14	12	1	SIAMPDVHSGYGFCIGNVAAFDMSDPAAVVSPGGVGFDINCGVR + Oxidation (M)
☒	2772	492.5896	983.1646	982.6175	0.5471	0	14	68	1	AVLTPLITR
☒	3630	687.3493	1372.6840	1371.6969	0.9871	1	14	1e+02	1	RFCFSQSVTLK
☒	6892	745.6299	2978.4905	2977.5616	0.9289	1	14	39	1	MKLLTFINLSGNNLSGSIPQSLLNMEK + Oxidation (M)
☒	3031	536.1639	1070.3132	1069.6244	0.6889	1	14	84	1	IQLRDLANK
☒	6112	509.2377	2541.1521	2542.1620	-1.0099	0	14	50	1	YDNICSYFDMPIQHASNNILK
☒	6572	694.3082	2773.2037	2773.3114	-0.1077	1	14	41	1	NNNNNSSNGNSISPEGSIALKSDDVVK
☒	6705	717.8598	2867.4101	2868.3171	-0.9070	1	14	45	1	VGDSPIPGAGAYANKFGGAAATGDGDVMMR + Oxidation (M)
☒	3415	430.5275	1288.5607	1289.6649	-1.1042	1	14	1.1e+02	1	KAIEQISQDMK
☒	4016	728.2543	1454.4940	1453.7823	0.7117	1	14	70	1	REIMHGSLLNLR + Oxidation (M)
☒	5182	629.5099	1885.5079	1886.0408	-0.5329	1	14	51	1	VRGAADVTIVGLMVSLNR + Oxidation (M)
☒	6416	534.2695	2666.3111	2667.3465	-1.0354	1	14	49	1	MSSFVQELDKAFEGQLQVLLEEK
☒	8330	639.7147	4470.9520	4470.1988	0.7532	1	14	13	1	MTVVTITPGAMSFADWRAIYEGASAALTVGAWDAIDASAAAVAR + Oxidation (M)
☒	6827	585.4735	2922.3311	2923.4426	-1.1115	0	14	40	1	YQNLPYNAPLTQTQPSTFGLIPMEGK + Oxidation (M)
☒	2850	501.7931	1001.5716	1002.5896	-1.0179	1	14	1.4e+02	1	AKLSAICLK
☒	3653	691.4375	1380.8604	1381.6772	-0.8168	0	14	92	1	AALETSGACPIHR
☒	4276	767.8279	1533.6412	1533.7133	-0.0721	0	14	88	1	EAIHVISCGYEEK
☒	6107	509.2373	2541.1501	2541.2744	-0.1243	1	14	50	1	TLAEALDMPSSTPEQAQIKDAAVR
☒	6425	667.5853	2666.3121	2665.2150	1.0971	0	14	49	1	NHGNNEADAALQGLAQQGVDMEDLR
☒	7429	895.9681	3579.8433	3579.8714	-0.0281	1	14	29	1	RIAIAGILAMNPEIIVLDEPTSGLDPMGASQIMK + Oxidation (M)
☒	4914	438.7481	1750.9633	1750.8638	0.0995	1	14	82	1	TREYINTHFNEVTK
☒	3263	610.1814	1218.3482	1217.6081	0.7402	0	14	80	1	VQHIPSVEFY
☒	4873	868.0693	1734.1240	1734.7804	-0.6563	0	14	63	1	APESLSQAEMDEVMAK
☒	4999	453.5059	1809.9945	1809.0261	0.9684	1	14	78	1	LPPGVAGTIVDVRVFNR
☒	5010	453.7563	1810.9961	1810.9538	0.0423	0	14	79	1	GTLHGGQTATLTDVITAR
☒	7581	621.6482	3723.8455	3723.9472	-0.1017	1	14	26	1	RNPTVITLYELAQALGVSHVELVTPTDETPFGTR
☒	7718	650.2761	3895.6129	3895.1332	0.4798	0	14	17	1	MVFVFIMMILIFLMNNWQTYAVGIILIFIILK + 3 Oxidation (M)
☒	6794	726.0512	2900.1757	2900.5425	-0.3668	1	14	33	1	VEEMMLDKASLLGLSVPEMTVLIGGLR
☒	4232	759.5071	1516.9996	1515.8443	1.1554	1	14	76	1	TEAGAMVGKIAISIR
☒	4986	453.5050	1809.9909	1810.8697	-0.8788	1	14	79	1	YNSDSEKITQTLQER
☒	6095	845.7283	2534.1631	2534.2283	-0.0652	0	14	52	1	QQVTQTMNAGIVSSGEVTSTEPVR + Oxidation (M)
☒	6600	703.0651	2808.2313	2807.2385	0.9928	0	14	42	1	NYYIGTTGIDTFGTMQFTSDFQER + Oxidation (M)
☒	6993	751.6024	3002.3805	3001.4671	0.9134	1	14	39	1	IMMHQPSAGIGGTAVDIAIQAESLERMK + 3 Oxidation (M)
☒	7373	707.7253	3533.5901	3534.7703	-1.1802	1	14	27	1	EQAEVAGVKPNHVLTTGGVQADASRTLWEESQK
☒	4483	795.9535	1589.8924	1590.6914	-0.7990	0	14	91	1	TAMTYVCGPGIMMK + 2 Oxidation (M)
☒	6438	534.2698	2666.3126	2667.3465	-1.0339	1	14	50	1	MSSFVQELDKAFEGQLQVLLEEK
☒	7071	624.1135	3115.5311	3114.4022	1.1289	1	14	38	1	NQGLSMSSDAHADEYSRYIVDGAAVAEMK
☒	7204	832.3704	3325.4525	3325.5668	-0.1144	1	14	29	1	VMIHQPASSFYEGQAGECMIEAEEVLKLK + 2 Oxidation (M)
☒	7571	621.4806	3722.8399	3723.9472	-1.1073	1	14	26	1	RNPTVITLYELAQALGVSHVELVTPTDETPFGTR
☒	5818	602.3163	2405.2361	2404.3359	0.9002	1	14	59	1	LGASLGSLSSGRISIISMSVVNLK + Oxidation (M)
☒	6577	696.1082	2780.4037	2779.3276	1.0761	1	14	48	1	GSLPDIVGDYFGLVPMDFHNRTSDK
☒	6766	719.3331	2873.3033	2872.3583	0.9450	0	14	42	1	AETNPETGQTLISGMGELHLDIMVER + 2 Oxidation (M)
☒	7056	767.1076	3064.4013	3063.5712	0.8301	1	14	38	1	HIPVAERHIGQPILWAPSCEQEQQLK
☒	7238	671.2983	3351.4551	3350.6197	0.8355	1	14	28	1	TAGAMPQIFGTITISDGISMGTEGMKYSLVSR + 2 Oxidation (M)
☒	7254	677.5285	3382.6061	3382.6795	-0.0734	1	14	31	1	MVSRPGDLPALEAGPGSSEGLLGGMSIPAGMTRR + Oxidation (M)
☒	7593	745.7783	3723.8551	3724.7458	-0.8907	0	14	26	1	TEVDGSLYTGALFFILMMLMFNGMSELSMTIAK + 4 Oxidation (M)
☒	2536	399.2448	796.4750	797.3378	-0.8627	0	14	51	1	GMGENYK
☒	2726	467.2997	932.5848	931.5021	1.0827	1	14	1.4e+02	1	MAAGARLAR + Oxidation (M)
☒	3511	661.1491	1320.2836	1319.6504	0.6333	1	14	72	1	GCDVRTTIVDGK
☒	4316	771.2928	1540.5710	1539.7906	0.7804	1	14	76	1	SFSHNSSLIKHQR
☒	8287	1115.2748	4457.0701	4457.2738	-0.2037	1	14	15	1	YNHMLSATVKIIQMLQHFEHLAPVLVAAVSLWATDYGMK + 2 Oxidation (M)
☒	8368	642.0021	4486.9638	4487.4388	-0.4750	1	14	13	1	LRADLSTLTADLPHLAVLSVNIQPVHQAILEGEQEVILTDR

☒	3277	618.1467	1234.2788	1233.6387	0.6401	1	14	74	1	SDSLIARDVMK
☒	4021	486.5486	1456.6240	1455.7180	0.9059	1	14	98	1	KMAFATSQTTGWK
☒	4181	750.4016	1498.7886	1498.6834	0.1052	0	14	96	1	MEHISGNSPEQVR + Oxidation (M)
☒	4441	524.8940	1571.6602	1571.7903	-0.1302	0	14	90	1	AVNADALQSTPTGTAR
☒	4271	767.3544	1532.6942	1531.8102	0.8840	1	14	95	1	KVVEMVPSIEMVR + Oxidation (M)
☒	4706	834.9073	1667.8000	1666.8724	0.9276	1	14	90	1	RPRRPGGSGGSGGSGGLR
☒	5499	517.0428	2064.1421	2064.9382	-0.7961	1	14	69	1	EVMINDENGKTVASTNDGR + Oxidation (M)
☒	6601	703.3137	2809.2257	2809.4037	-0.1780	1	14	43	1	GRMIIPANINHANLEPMAIGIASSCK + 2 Oxidation (M)
☒	2490	384.2946	766.5746	766.3466	0.2281	0	14	26	1	MAATAMR + Oxidation (M)
☒	3008	528.2488	1054.4830	1055.6161	-1.1331	0	14	1.1e+02	1	SPAIGIVMLR
☒	3024	533.3385	1064.6624	1063.4716	1.1908	1	14	1.1e+02	1	MAESRSPDR + Oxidation (M)
☒	3633	687.5504	1373.0862	1372.8112	0.2751	1	14	73	1	LTELLSMIVAKR
☒	4987	453.5052	1809.9917	1810.8697	-0.8780	1	14	80	1	YNSDSEKITQTLQER
☒	5346	1001.9188	2001.8230	2002.0153	-0.1923	0	14	65	1	SLLSSMDNHTTLLLQTGR + Oxidation (M)
☒	7988	833.3587	4161.7571	4161.0187	0.7384	1	14	14	1	SAVMDFAAATILREAGFEQFDVVAGGETAGIPFAAMLAER + 2 Oxidation (M)
☒	8662	966.4609	4827.2681	4828.4160	-1.1479	1	14	11	1	LPMQATVISVIVAGDFVLSFLSLGGACSTASVAVLLMDAGEKQCDR + 2 Oxidation (M)
☒	4078	738.6356	1475.2566	1475.6674	-0.4108	0	14	69	1	GDQDLMAAGNAIER + Oxidation (M)
☒	5862	488.6202	2438.0646	2438.1471	-0.0825	1	14	53	1	EMGFIGGKMALHHGPAEGEEGLR + Oxidation (M)
☒	3158	577.3644	1152.7142	1153.6931	-0.9789	1	14	1e+02	1	KGLVNQILNR
☒	4443	786.8635	1571.7124	1572.7243	-1.0118	0	14	95	1	VFTPFMQADSSTSR
☒	4978	602.2994	1803.8764	1803.9441	-0.0677	1	14	86	1	VPFMLVIGEKEVNEGK + Oxidation (M)
☒	6518	544.8527	2719.2271	2718.3646	0.8625	1	14	46	1	MNEQQLLENIASLAGAINQYKNEK
☒	6930	600.1084	2995.5056	2996.6488	-1.1431	1	14	42	1	LLTPTHFEFVFKNATPAVSPNLTLLAR
☒	4222	758.3732	1514.7318	1514.7213	0.0106	1	14	99	1	EDGEGSVQVKQDPK
☒	3275	617.0364	1232.0582	1231.6496	0.4087	1	14	83	1	LWEVVMQRR + Oxidation (M)
☒	4958	596.6105	1786.8097	1785.9407	0.8690	0	14	86	1	RPDVLQQLTVSIMDR + Oxidation (M)
☒	6161	512.0333	2555.1301	2554.0815	1.0487	0	14	50	1	HFSMMILSDDGVVCYNSDYAAK + 2 Oxidation (M)
☒	8238	631.2809	4411.9154	4411.2034	0.7119	1	14	13	1	TRAFITHGGANGIYEAIYHGIPMVGIPLFFDQPDNIAHMK
☒	2992	525.5901	1049.1656	1048.4897	0.6759	1	14	84	1	TRQTDDSAR
☒	3756	473.5815	1417.7227	1417.7639	-0.0412	1	14	1.1e+02	1	EKMGPVELQFIK
☒	5873	611.5466	2442.1573	2442.0726	0.0847	0	14	57	1	EMAMMQEGEHTVTSHSSIIHR + 2 Oxidation (M)
☒	6854	490.7549	2938.4857	2938.4771	0.0086	1	14	44	1	SLTDLTSLETIIETAFDNRDGVNVSTK
☒	8069	1062.5028	4245.9821	4245.1371	0.8450	1	14	18	1	AIVIVALDNLTKGSSGQALQNANLMLGEDETAGLMMAPLFP + 2 Oxidation (M)
☒	3662	692.4868	1382.9590	1382.7082	0.2509	0	14	79	1	SGIFGEAYGLIEK
☒	4367	779.1226	1556.2306	1556.8787	-0.6480	1	14	66	1	RLLNSITHPEIHK
☒	4465	791.1315	1580.2484	1579.7366	0.5119	0	14	62	1	TEDDVPLSSVYGNGK
☒	6062	633.2783	2529.0841	2530.1832	-1.0991	1	14	49	1	TEADLAMFNEFMISLGRDAAANK + Oxidation (M)
☒	6580	558.2736	2786.3316	2787.3133	-0.9817	1	14	49	1	LNVDLEAAKMAESNAHSLSNEWQSK + Oxidation (M)
☒	3005	528.2485	1054.4824	1054.5771	-0.0946	0	14	1.1e+02	1	AILQAEPSAR
☒	5414	1014.2149	2026.4152	2027.0034	-0.5881	0	14	49	1	EAGLFDVSHMGEIIVEGPK
☒	6147	512.0327	2555.1271	2554.0815	1.0457	0	14	50	1	HFSMMILSDDGVVCYNSDYAAK + 2 Oxidation (M)
☒	3754	473.5814	1417.7224	1418.6929	-0.9705	0	14	1.1e+02	1	VYDQADELPLEK
☒	5856	812.7510	2435.2312	2436.3297	-1.0986	0	14	58	1	LGLVEAEAIPQEEALASILLMAR
☒	6203	863.3801	2587.1185	2586.2642	0.8543	1	14	49	1	AACNCLKNAAAGVSGLNAGNAASIPSK
☒	7108	638.1390	3185.6586	3185.5009	0.1577	1	14	37	1	EALEFFSDMIAAGHKSDEVSMTSIIAASGR + Oxidation (M)
☒	2777	493.8301	985.6456	984.4876	1.1580	0	14	1.4e+02	1	IDLADDPAR
☒	5883	617.0344	2464.1085	2463.1248	0.9836	1	14	56	1	GSEGDNISLSSLSEETEEKSHTK
☒	3100	379.5056	1135.4950	1136.4889	-0.9939	1	14	1.2e+02	1	CMGCGAKTPR
☒	3524	664.8629	1327.7112	1328.6837	-0.9724	0	14	1.1e+02	1	ENLTTAQFLHR
☒	6403	667.5848	2666.3101	2665.2150	1.0951	0	14	52	1	NHGNNEADAALQGLAQQGVDMEDLR
☒	8409	753.5219	4515.0877	4514.1644	0.9234	1	14	15	1	SGQMMLAQGLLLHLMPTDWRWSDCHALTDVDFEVLKPR + 3 Oxidation (M)
☒	8057	708.3270	4243.9183	4243.0089	0.9094	0	14	17	1	ELEPNVTLITEGNAEDVCIWFVMTGTLAVYQSNADATR + Oxidation (M)
☒	4703	833.9621	1665.9096	1665.9050	0.0047	1	14	90	1	ISSSVSKNTYAVVVGR
☒	4705	834.4070	1666.7994	1666.7694	0.0300	1	14	92	1	MAETKDLPFEEAMR
☒	7704	775.1687	3870.8071	3869.9305	0.8766	1	14	24	1	LDEMGVERASVTLHVGAGTFKPIQGPVADHVMHAER + Oxidation (M)
☒	3602	454.8937	1361.6593	1360.7285	0.9308	1	14	1.1e+02	1	MFLNLLDAPRR + Oxidation (M)
☒	3748	708.8390	1415.6634	1415.7045	-0.0411	0	14	1.1e+02	1	TFGVQEGEAITHK
☒	4928	882.7571	1763.4996	1763.8876	-0.3879	1	14	61	1	AVVAPTAREENVYMAK + Oxidation (M)
☒	4969	449.7107	1794.8137	1794.8247	-0.0110	1	14	85	1	DDPHACYSTVFDKLK
☒	6395	667.5829	2666.3025	2667.3292	-1.0267	0	14	53	1	HWETTILPSGYGLEARPVAEANEK
☒	6858	490.9213	2939.4841	2938.4771	1.0070	1	14	45	1	SLTDLTSLETIIETAFDNRDGVNVSTK
☒	5520	697.6415	2089.9027	2091.0266	-1.1240	0	14	68	1	TDSAQVAEIVAVMGNASVASR + Oxidation (M)
☒	5666	744.7498	2231.2276	2232.0878	-0.8603	1	14	66	1	VMDKAMEVGAPVIGLNDSAGAR + 2 Oxidation (M)
☒	6551	553.6363	2763.1451	2762.4386	0.7066	1	14	41	1	ESGTAVMVTGGGTNLIVSDAGFRGLVVR
☒	6881	987.4604	2959.3594	2960.4418	-1.0825	1	14	43	1	KALYEFFSALMEPWDGPALISFTDGR
☒	4597	817.0017	1631.9888	1631.8301	0.1588	1	14	87	1	SLRMNGVTLDLEER
☒	3152	574.2594	1146.5042	1145.5537	0.9505	1	14	1.3e+02	1	NGSDLRNQSR
☒	3502	659.5410	1317.0674	1317.6929	-0.6254	0	14	78	1	LGVNTAAFPVSDK
☒	5929	497.8330	2484.1286	2484.2943	-0.1657	1	14	57	1	RALIDCFICIDTVQFMIILK + Oxidation (M)
☒	6137	511.8329	2554.1281	2553.1412	0.9869	0	14	51	1	FIAASEYALDFMCGMLTMNPQK + Oxidation (M)
☒	6870	592.4834	2957.3806	2957.4481	-0.0675	1	14	43	1	VTLLDGQYHDVDSSELAFKIAGSMAFK + Oxidation (M)
☒	7166	1094.1420	3279.4042	3279.5470	-0.1428	1	14	31	1	YNLNYIKMEGGIGCMVNGAGLAMATMDIIK + 2 Oxidation (M)
☒	8242	883.5909	4412.9181	4411.9840	0.9341	1	14	14	1	DGALGIDDRPVETEHEDAKSYSQQETFDEDIASFAEIER
☒	4956	596.3141	1785.9205	1784.8689	1.0516	0	14	92	1	LLDMVVELDDHVMEK
☒	6657	712.8278	2847.2821	2848.3839	-1.1018	1	14	47	1	SGEELSGSETEGDAAVRFVLVAAVADNR
☒	7068	777.8378	3107.3221	3107.5148	-0.1927	1	14	34	1	KFNEAPSADGVWHHCHLLPAADPAQAIR
☒	5359	669.7759	2006.3059	2006.9585	-0.6526	0	14	55	1	ASFNTEELEDLEGVLWR
☒	5913	619.0543	2472.1881	2472.3224	-0.1343	1	14	59	1	FVEAGKLDGAAAITEDALVASGLVR
☒	6545	551.2703	2751.3151	2752.4615	-1.1464	1	14	50	1	LKEAMLAAGALGAVMSGAGPSVLGVVPDR + Oxidation (M)
☒	7037	762.8259	3047.2745	3047.5888	-0.3143	0	14	34	1	QAVNGASTNLAVAGSHLPTTQVTQVDIVEK
☒	4343	387.7285	1546.8849	1545.8878	0.9970	1	14	1e+02	1	LKHLVDEPQNLIK
☒	5804	596.0584	2380.2045	2380.1515	0.0530	1	14	62	1	TAEACFMPLTVGGGVRSVEDIR + Oxidation (M)
☒	6331	528.2286	2636.1066	2635.3713	0.7353	0	14	46	1	MGNALPLTDMPLGTAIHNIEITLGK + Oxidation (M)
☒	6547	690.8186	2759.2453	2758.5018	0.7435	0	14	50	1	QPVLLLGGGSNVLFTTNFEGTVILNR
☒	3736	706.1719	1410.3292	1410.8922	-0.5630	1	14	71	1	GVLLLNSVLTVKR
☒	3986	723.0786	1444.1426	1444.7456	-0.6030	1	14	72	1	ADMREQALLQVR + Oxidation (M)
☒	4895	582.9539	1745.8399	1745.8618	-0.0219	1	14	94	1	LQQEMRATIEADDVK
☒	5727	763.0524	2286.1354	2287.1267	-0.9913	1	14	67	1	AGVHSGDSMAAYPPQTLSQKVK + Oxidation (M)
☒	6769	960.8033	2879.3881	2879.4092	-0.0212	1	14	48	1	VEPGSTCAVFGLGGVGLAAIMGCKAAGASR + Oxidation (M)
☒	6987	751.3526	3001.3813	3001.3732	0.0081	1	14	42	1	CCSESLVNRRPCFSGLEVDETYVPK
☒	2763	489.4239	976.8332	976.5124	0.3209	1	14	98	1	RLAMASANK + Oxidation (M)
☒	3410	429.9050	1286.6932	1286.6103	0.0829	0	14	1.2e+02	1	HSDASLTDTVNK
☒	4365	389.7082	1554.8037	1555.7188	-0.9151	0	14	1e+02	1	FMDITDTPSLGSTR + Oxidation (M)
☒	5038	609.7927	1826.3563	1825.9284	0.4279	1	14	57	1	SDMDFLVRIFDEVIK
☒	6119	637.2979	2545.1625	2546.2356	-1.0731	1	14	56	1	QDPDIIMLGEIRDEESAMIALR + 2 Oxidation (M)
☒	4728	839.5691	1677.1236	1677.8686	-0.7449	1	14	67	1	HVENGKQVEVSDIPK
☒	4892	873.4440	1744.8734	1745.8407	-0.9672	0	14	93	1	AGGPSASMEPGIPFSIGR + Oxidation (M)
☒	6695	574.2888	2866.4076	2865.4708	0.9368	1	14	51	1	LQLAALGYVFPDRAMDHLHALAAGGSR + Oxidation (M)
☒	2754	974.4133	973.4060	973.4716	-0.0656	0	14	1.6e+02	1	AADADLAEAK
☒	4963	596.9820	1787.9242	1786.9465	0.9777	0	14	92	1	FDPVDIQATLVELTAR
☒	5443	682.3408	2044.0006	2045.0616	-1.0610	1	14	76	1	LMFGSLGRASGATSVIFTSK + Oxidation (M)

☒	6181	515.2316	2571.1216	2570.3169	0.8047	1	14	52	1	KAFELFTAFDSQYAQSHQLIVK
☒	3536	670.3226	1338.6306	1339.6368	-1.0062	0	14	1.1e+02	1	TGNTASGFEVTTR
☒	2656	452.2763	902.5380	901.4981	1.0399	1	14	1.6e+02	1	SGRLEGVGK
☒	5202	950.0977	1898.1808	1897.0165	1.1643	0	14	66	1	ALAMLDVIVLDHMIVGR + 2 Oxidation (M)
☒	6179	643.7869	2571.1185	2571.2738	-0.1553	1	14	51	1	TDLDKLVMDIETNGVVDPEEAIR
☒	5084	465.5018	1857.9781	1856.8437	1.1344	0	14	88	1	YPHAPEMEPTAMINPK + 2 Oxidation (M)
☒	5334	663.8728	1988.5966	1988.0435	0.5531	0	14	55	1	ALLLSCQMTDEGVLLVAR
☒	6342	662.0400	2644.1309	2643.3769	0.7540	1	14	48	1	QQLDYLGQRVIAADSGEAALALWR
☒	2736	472.2864	942.5582	942.5134	0.0448	0	14	1.5e+02	1	LLLAGGDER
☒	6236	522.8649	2609.2881	2608.2268	1.0613	1	14	56	1	YFGGMINKSIDFNTIDGFQGQEK
☒	3286	623.3852	1244.7558	1244.6183	0.1375	0	14	1.2e+02	1	NPSDMLSQVVR
☒	3726	705.0854	1408.1562	1407.7721	0.3841	0	14	72	1	RPPDDIEALLAAK
☒	4699	832.6522	1663.2898	1662.9053	0.3846	1	14	63	1	ALSPNEEKLHSILGR
☒	6281	523.4369	2612.1481	2611.2986	0.8495	1	14	51	1	QGQGVVTIGAMAKGDSEFMASTVLAK + Oxidation (M)
☒	8035	841.9764	4204.8456	4204.1384	0.7072	1	14	17	1	MQVDHGCADALAALTGSLHRYPVIPVFINSVAPPMATLR + Oxidation (M)
☒	2527	789.7131	788.7058	788.4028	0.3030	0	14	81	1	TAQEVNK
☒	6129	511.8315	2554.1211	2554.0815	0.0397	0	14	52	1	HFSMMILSDDGVVCYNSDYAAK + 2 Oxidation (M)
☒	2770	491.4899	980.9652	981.4073	-0.4421	0	14	77	1	EMVSEDTR + Oxidation (M)
☒	3081	560.2805	1118.5464	1119.5520	-1.0056	1	14	1.4e+02	1	VKDAQGGGSSSK
☒	3221	394.2235	1179.6487	1180.5547	-0.9060	0	14	1.2e+02	1	DGQMNVYGGIK
☒	3522	664.4661	1326.9176	1326.6238	0.2939	1	14	91	1	ANMYLTEGEKR + Oxidation (M)
☒	2621	879.4231	878.4158	879.5290	-1.1132	0	14	1.4e+02	1	TAIVIAHR
☒	5522	419.2131	2091.0291	2089.9449	1.0842	0	14	77	1	FVNLNDAYCSTSSIMPQK + Oxidation (M)
☒	6767	959.7841	2876.3305	2875.5516	0.7789	1	14	46	1	LIQVPLSQEVAPSGVNVQRSSVNGELR
☒	5581	538.0202	2148.0517	2149.0764	-1.0247	0	14	73	1	QASHGDLTISSVPAAPTPSASR
☒	3612	682.3384	1362.6622	1361.6761	0.9861	0	14	1.1e+02	1	HLSTILECAYR
☒	4156	373.9353	1491.7121	1492.8110	-1.0989	1	14	1.1e+02	1	ESHRAQALAELLR
☒	4910	584.3270	1749.9592	1749.8468	0.1124	0	14	93	1	SAFGASSGGLSGAPMRPGK + Oxidation (M)
☒	7269	851.8626	3403.4213	3403.4746	-0.0533	0	14	27	1	QEDFVTTASAELMGGQEDEDSDNDNAHLLPR
☒	8730	927.9803	5561.8381	5561.6164	0.2217	1	14	7.5	1	MTDNDALSLPPDVFAAEDLAAEIDEETSCVVVQTPDAFGNLRDLSPLAAACK
☒	3351	630.5247	1259.0348	1259.5605	-0.5256	0	14	85	1	FGYGTMDIGSGR
☒	7297	859.8841	3435.5073	3435.5857	-0.0784	1	14	29	1	GFQDVEAQAATCNHTVMALMASLDAEKAQGQK + Oxidation (M)
☒	3018	529.8588	1057.7030	1058.5906	-0.8876	1	14	1.4e+02	1	LMRGINVEK
☒	5822	602.3174	2405.2405	2405.2914	-0.0509	1	14	65	1	NINTQLSTSGGTSDGRFIALILK
☒	6376	667.2715	2665.0569	2664.2887	0.7682	0	14	41	1	VIIAYGPSECTVGCTINNEIALDR
☒	3261	609.6690	1217.3234	1216.7027	0.6208	0	14	91	1	TQTQVSIISIK
☒	3658	692.2794	1382.5442	1381.7275	0.8167	1	14	1e+02	1	MLSGKGDVLYVGK + Oxidation (M)
☒	4949	594.9775	1781.9107	1781.9635	-0.0529	1	14	91	1	QLLEDNKQALDGALVR
☒	5263	387.9719	1934.8231	1934.9367	-0.1136	1	14	77	1	SAITAGNKEEAASAMGVANK + Oxidation (M)
☒	6143	852.7161	2555.1265	2556.3197	-1.1932	1	14	54	1	GDLADPQAVRHALAGIDAVFHNAAK
☒	5193	945.9620	1889.9094	1890.9106	-1.0011	0	14	89	1	NVMSTSSTVTTELVNHR + Oxidation (M)
☒	4823	569.2893	1704.8461	1703.8916	0.9545	1	14	97	1	YMDSLGAKHLVETLK
☒	4964	894.9974	1787.9802	1786.9070	1.0733	0	14	94	1	MCLGIPGQIVAITDAGR + Oxidation (M)
☒	6700	717.8580	2867.4029	2868.4116	-1.0087	1	14	51	1	VDPMAIVVFHQADIGEYVRHEETLT
☒	8773	964.1293	5778.7321	5777.7408	0.9913	1	14	7.2	1	LSGEVPFVHMLNATMCATTRVICAILENNQTEEGINVPTAIQQWMPENYR + 2 Oxidation (M)
☒	4561	540.9610	1619.8612	1619.7977	0.0634	0	14	1e+02	1	SEAPFVGTGMEAVVAR
☒	6240	435.8890	2609.2903	2608.1819	1.1085	1	14	57	1	ASENAEVDIPQAMVDTELDRMMK + Oxidation (M)
☒	6653	711.6018	2842.3781	2841.4443	0.9338	1	14	51	1	ALAKMWGSEANDPITVANGRPTLATNK + Oxidation (M)
☒	6799	970.1113	2907.3121	2906.5649	0.7471	1	14	45	1	LIAYSIVVDKVSNMLCEWGNIILIK + Oxidation (M)
☒	2669	453.7469	905.4792	904.4502	1.0291	0	14	1.6e+02	1	DVSENITK
☒	4225	758.8132	1515.6118	1516.7807	-1.1688	0	14	99	1	TSNSAVIEMLPVQI + Oxidation (M)
☒	5872	814.7983	2441.3731	2441.1290	0.2440	1	14	57	1	RLCTEHGSVLIFDEVMCGFR + Oxidation (M)
☒	7986	693.6152	4155.6475	4156.8423	-1.1948	1	14	15	1	ATAQPQMETVESSQTEEKTDAVEETKPTESAQQEEMK + 2 Oxidation (M)
☒	2472	379.2469	756.4792	757.4195	-0.9402	0	14	70	1	VRPSSGR
☒	3252	603.2598	1204.5050	1205.5962	-1.0911	0	14	1.3e+02	1	ISEELQICSK
☒	3260	609.6689	1217.3232	1216.7027	0.6206	0	14	92	1	TQTQVSIISIK
☒	4867	433.2476	1728.9613	1728.9271	0.0342	1	14	97	1	SSGPYALVTQQPLRGR
☒	5426	1017.4598	2032.9050	2033.0038	-0.0988	1	14	79	1	STPEQRTAAQNELAHQPR
☒	4351	775.7932	1549.5718	1549.7658	-0.1939	1	14	84	1	MDETVSELKDLVR + Oxidation (M)
☒	5257	645.2802	1932.8188	1931.9445	0.8743	1	14	79	1	RSLGSPTVAEPAMIAECK + Oxidation (M)
☒	5507	518.9865	2071.9169	2071.8221	0.0948	1	14	75	1	KNTGAGGMPPGGGGMGGMGGMDF + 2 Oxidation (M)
☒	6052	841.9995	2522.9767	2522.1644	0.8123	1	14	44	1	YVMILSGDHIYRMDYGDMLAK + 2 Oxidation (M)
☒	6278	523.4368	2612.1476	2612.1860	-0.0384	1	14	53	1	QVTGINGNYGGMAQVYGREGEPCR
☒	6293	654.0448	2612.1501	2611.3255	0.8246	1	14	53	1	RPQLIHFLETHESHERAGPTEK
☒	6823	730.8380	2919.3229	2919.6038	-0.2809	1	14	46	1	LGIPVVCVGNLTAGGAGKTPVALAVMDALR
☒	4198	503.2526	1506.7360	1507.8147	-1.0787	1	14	1.1e+02	1	LRWPIPEDLDVR
☒	4818	852.1426	1702.2706	1702.7953	-0.5246	1	14	67	1	SIKANHPLNMEMMR + 2 Oxidation (M)
☒	3693	699.6101	1397.2056	1397.7449	-0.5392	1	14	79	1	AEKMLSAVGLSHR
☒	5377	404.8096	2019.0116	2018.1928	0.8188	0	14	83	1	EHLGLALALNVPVFVVVTK
☒	6874	592.4854	2957.3906	2957.3362	0.0544	1	14	46	1	GGSTLRDFSNAQGEAGHFQLDAMVYDR + Oxidation (M)
☒	7467	721.7391	3603.6591	3603.7623	-0.1032	1	14	30	1	MKFGVSEGMILSAGTGGSDLFLLSADNGVTAGMQVK + Oxidation (M)
☒	2757	488.5692	975.1238	975.5978	-0.4739	1	14	1.1e+02	1	HTKLKPPR
☒	6412	534.2695	2666.3111	2666.3010	0.0101	1	14	57	1	DRDYVVVGATPEQMIAQNYTPVGK + Oxidation (M)
☒	7253	677.5272	3382.5996	3382.6795	-0.0799	1	14	35	1	MVSRPGDLPALEAGPGSSEGLLGGMSIPAGMTRR + Oxidation (M)
☒	8725	1113.3715	5561.8211	5562.8353	-1.0142	0	14	7.9	1	MNSIAILEAVNTSYVPFNGQHVLTAMVAGVAYVAMKPVVDNIGLSWSSQVQK + Oxidation (M)
☒	3431	649.6273	1297.2400	1297.6522	-0.4122	1	14	77	1	YCGIIIEKMR + Oxidation (M)
☒	6328	878.4092	2632.2058	2631.5437	0.6621	1	14	55	1	MLLPSLFESYKGLIIIISLGAVVR
☒	4586	814.7303	1627.4460	1626.8300	0.6160	0	14	70	1	ATWAVAVGGPNVMGAAR
☒	5077	619.3336	1854.9790	1853.8717	1.1073	0	14	90	1	FMVSDDGTELGISNIEK
☒	6945	600.1097	2995.5121	2994.4824	1.0297	1	14	47	1	SSMGMAISAVKTGEADAALSAGNTGALMAIAK
☒	8495	648.1461	4529.9718	4531.1622	-1.1904	1	14	14	1	DLDKASNSLISGLSQDEEDPPLPPTPMNSLVDECPLDQGLPK
☒	3099	379.5054	1135.4944	1136.6050	-1.1106	1	13	1.4e+02	1	HELENLRAR
☒	5209	476.7219	1902.8585	1902.0761	0.7824	0	13	87	1	MLQLLLAVFIGGGTGSVAR
☒	6228	522.8646	2609.2866	2609.3352	-0.0486	1	13	59	1	WFLKGYQNANYLSLFLMVATSK + Oxidation (M)
☒	7762	794.5849	3967.8881	3968.9297	-1.0416	1	13	24	1	MLKCIAAVGTVVWMTMFLFLYSQLSNNQSGGDSIR + 2 Oxidation (M)
☒	3359	632.1406	1262.2666	1262.5748	-0.3081	0	13	88	1	NGGICSTCGLPK
☒	3566	676.5014	1350.9882	1351.7936	-0.8053	0	13	78	1	ALVAAGGVAANVAIR
☒	4723	559.9368	1676.7886	1675.7909	0.9976	1	13	1e+02	1	MQSVPLSEEMHKTK + 2 Oxidation (M)
☒	5649	1107.0555	2212.0964	2211.1171	0.9793	1	13	75	1	QFISSLEDAETIKSFNNLR
☒	6546	689.3902	2753.5317	2753.4044	0.1272	0	13	51	1	ADDMIIELLSPEDLVITADIPLADR + Oxidation (M)
☒	6886	990.7990	2969.3752	2969.3488	0.0264	0	13	48	1	DQVMETLAFIEAGIDFPEDDVESLDR + Oxidation (M)
☒	6399	445.3920	2666.3083	2665.3179	0.9905	1	13	58	1	MLHFPVATVLEKQHNMGEAPCLK + Oxidation (M)
☒	8947	836.4961	6683.9106	6683.2664	0.6442	1	13	3.6	1	SSFPSPRLSLPTDSLSPDGGSIELEFYLAPEPFSMPSLLGAPPYSGLGGVGDPYVPLMVLMCR + Oxidation (M)
☒	3734	705.7716	1409.5286	1408.7198	0.8089	1	13	97	1	TQTLESKAFQEK
☒	5417	677.0029	2027.9869	2028.8881	-0.9012	0	13	84	1	MPSPSPEASSSMSQPGPPSR + Oxidation (M)
☒	6103	636.0448	2540.1501	2540.0849	0.0652	1	13	60	1	CDSPNTKFCYYNNYNLSQPR
☒	7546	615.1045	3684.5833	3684.6924	-0.1090	1	13	25	1	VNGLDTPGHEDFSEDTYRTLMAVDAAVMVVDSAK + 2 Oxidation (M)
☒	2564	814.3766	813.3693	813.4960	-0.1266	0	13	1.2e+02	1	SKPILEK
☒	3830	713.8160	1425.6174	1424.7776	0.8399	0	13	1.1e+02	1	SQIHISQPQFLK
☒	4379	782.3333	1562.6520	1563.8001	-1.1480	0	13	1e+02	1	VDVMINGTVTSAMVK
☒	7599	932.2219	3724.8585	3725.7270	-0.8685	1	13	30	1	AGMDRVQADYTGMLGTVMNALVMADALQHAGVDTR + 3 Oxidation (M)

☒	8631	1176.3083	4701.2041	4701.3081	-0.1040	1	13	15	1	YLDIPDIIEYAMNEVDYINNPTVEQILSTQKIVTDMIESR + Oxidation (M)
☒	3526	443.8943	1328.6611	1329.6652	-1.0041	0	13	1.3e+02	1	FAPKPPAWMNR + Oxidation (M)
☒	3575	678.2852	1354.5558	1354.6881	-0.1323	0	13	1.1e+02	1	IQGLYFTETQR
☒	2739	475.2385	948.4624	948.4851	-0.0227	0	13	1.6e+02	1	YGCIPIAR
☒	5972	625.2823	2497.1001	2497.1478	-0.0477	1	13	59	1	EQCPSGHQVMKETFGPPDGLQR
☒	8124	1072.2417	4284.9377	4286.0008	-1.0631	1	13	19	1	ETFTHYQPQGNSDPAHTATAPGGLSAKAPAMTPLMLDTSSR + 2 Oxidation (M)
☒	5189	630.6115	1888.8127	1887.9976	0.8151	1	13	88	1	VTPRIDDVATMLATLEK + Oxidation (M)
☒	6140	512.0322	2555.1246	2556.2278	-1.1032	1	13	57	1	DAPDYQNMLNGVPPINKNEVNAK + Oxidation (M)
☒	2513	782.3330	781.3257	782.3844	-1.0587	0	13	48	1	TMTISSK + Oxidation (M)
☒	4485	796.4548	1590.8950	1590.8036	0.0915	1	13	1.1e+02	1	NAQGVGKATVTMPSSK + Oxidation (M)
☒	5335	995.4536	1988.8926	1988.0295	0.8632	1	13	86	1	AQYFLKVVELFDEYPK
☒	4654	820.5318	1639.0490	1638.9305	0.1186	1	13	83	1	KVPQVSTPTLVEVSR
☒	5352	401.8067	2003.9971	2003.9622	0.0349	1	13	87	1	EMVDYISQYLSTVRER + Oxidation (M)
☒	7823	670.6162	4017.6535	4016.8878	0.7658	1	13	18	1	SDALVGIQDMGAAGLTSSSAEMASKAGSGIEMNLDHVPQR + Oxidation (M)
☒	5792	786.3813	2356.1221	2355.9663	0.1558	1	13	74	1	GGSSRGGGQPSGGFSNDMDDDIPF
☒	6603	563.0848	2810.3876	2810.3834	0.0042	1	13	56	1	LAQSLDLGAYLYISQSEENNNGRQK
☒	7168	656.8885	3279.4061	3279.5470	-0.1409	1	13	35	1	YNLNYIKMEGGIGCMVNGAGLAMATMDIIK + 2 Oxidation (M)
☒	7409	590.6084	3537.6067	3537.9017	-0.2950	1	13	32	1	MNLPTVGGKIILSLDHLLEYKPNQVDLFNTR
☒	2627	442.2645	882.5144	882.5174	-0.0030	0	13	1.2e+02	1	LEVIPANK
☒	3344	628.3795	1254.7444	1255.5503	-0.8059	0	13	1.3e+02	1	DGGTQFEVMTR + Oxidation (M)
☒	7085	1046.1597	3135.4573	3136.4416	-0.9843	1	13	43	1	VVTIFSAPNYCYRCGNMASILEVDDSR
☒	3442	651.7669	1301.5192	1302.7183	-1.1991	0	13	1.2e+02	1	TQEILSQLPFK
☒	5348	501.4929	2001.9425	2001.9868	-0.0443	1	13	87	1	SKQGIPPTLEENHNSSHK
☒	7188	828.1233	3308.4641	3308.7187	-0.2546	1	13	36	1	IITHPNFNGNTLDNDIMLIKLSSPATLNSR
☒	7360	703.3378	3511.6526	3510.7634	0.8892	1	13	34	1	LLAEICLDIQEPMMYVLPYNSKCDLLTLR
☒	7887	812.6263	4058.0951	4058.1000	-0.0049	1	13	22	1	FVAEAKEAGVDGLIIVDLPPEHDAELATPAQAAGIDFIR
☒	2970	518.2070	1034.3994	1035.4693	-1.0699	1	13	1.5e+02	1	RESSNSGGSR
☒	3102	568.8506	1135.6866	1136.5938	-0.9072	0	13	1.4e+02	1	GPAGSVPASAPAR
☒	3680	695.0621	1388.1096	1387.7129	0.3967	1	13	84	1	ITSPEGLGMSPKR + Oxidation (M)
☒	5430	679.6770	2036.0092	2037.1180	-1.1089	0	13	85	1	VSLGITTVLTMTFLGLEAR + Oxidation (M)
☒	2676	457.2697	912.5248	911.5804	0.9444	1	13	1.5e+02	1	TKGLVAPVK
☒	4133	495.5422	1483.6048	1482.8518	0.7530	1	13	1.1e+02	1	TGVEIAGKNAVVIGR
☒	5022	605.5873	1813.7401	1812.9006	0.8395	1	13	85	1	TSKGPEEEHPSVTLFR
☒	3543	671.8122	1341.6098	1340.6143	0.9955	0	13	1.3e+02	1	GMVPGGEHEGSLR + Oxidation (M)
☒	4197	503.2525	1506.7357	1505.8098	0.9258	0	13	1.1e+02	1	LMSGILAVGPMFVR + Oxidation (M)
☒	4855	863.4116	1724.8086	1724.8623	-0.0537	1	13	99	1	VNKTTSKPVQMMSMK + Oxidation (M)
☒	4909	584.3269	1749.9589	1749.8468	0.1121	0	13	1e+02	1	SAFGASSGGLSGAPMRPGK + Oxidation (M)
☒	5074	927.3752	1852.7358	1852.9539	-0.2181	1	13	79	1	DKSVMMASQSLIQLFR
☒	5500	689.3281	2064.9625	2066.1023	-1.1398	1	13	84	1	VPAGAYGVIPIFSDVMRFK
☒	7164	817.8615	3267.4169	3266.5120	0.9049	1	13	36	1	RPQMSFLKMDMTQMEFPDASFQVVLDK + 3 Oxidation (M)
☒	3222	590.8632	1179.7118	1180.6061	-0.8942	1	13	1.3e+02	1	SKPHGESRQR
☒	4055	491.8724	1472.5954	1472.6855	-0.0902	1	13	1.1e+02	1	IGEVSGDDSSHRSK
☒	4799	424.2166	1692.8373	1693.7213	-0.8840	0	13	1e+02	1	NTQNPDSTTMDEVSR
☒	6222	869.5001	2605.4785	2606.3024	-0.8239	0	13	54	1	MTNPIHIVGGGLAGSEAAWQIAQGGR + Oxidation (M)
☒	3753	473.5809	1417.7209	1417.7300	-0.0092	1	13	1.3e+02	1	LKDSGDVSDIIEK
☒	3983	722.7947	1443.5748	1444.7423	-1.1674	0	13	1.1e+02	1	IDGQQGGAGFLGIGR
☒	4711	835.8612	1669.7078	1669.6997	0.0081	0	13	99	1	VEEGTAMVECEICK + Oxidation (M)
☒	4994	453.5056	1809.9933	1808.8363	1.1570	1	13	95	1	DYQRMLAAGASDTAPDK
☒	5212	636.3292	1905.9658	1906.0193	-0.0536	1	13	93	1	MEASALTSSAVTSVAKVVR
☒	5991	500.6281	2498.1041	2498.2098	-0.1056	1	13	63	1	QISKILEEFDVEEQSSTMLEK + Oxidation (M)
☒	6270	654.0439	2612.1465	2613.3398	-1.1933	1	13	56	1	DSSLHELQITLGGLGYEDLEIRR
☒	8283	1115.2725	4457.0609	4457.2738	-0.2129	1	13	18	1	YNHMLSATVKIIQMLQHFEHLAPVLVAAVSLWATDYGMK + 2 Oxidation (M)
☒	4675	824.7750	1647.5354	1646.7981	0.7374	0	13	75	1	YIYGGSGAVEPYPFK
☒	5406	405.0100	2020.0136	2021.0290	-1.0154	1	13	88	1	RNNGVPLVQHESLQSDTK
☒	6415	534.2695	2666.3111	2667.3465	-1.0354	1	13	61	1	MSSFVQELDKAFEGQLQVLLEEK
☒	6481	672.4055	2685.5929	2685.3540	0.2389	0	13	47	1	TAMALALITSGLNAVGSVVSMMFVDR + 2 Oxidation (M)
☒	6868	984.7560	2951.2462	2950.7003	0.5459	0	13	43	1	VGGLMVLAAAALVIILGVYPDPMINLALK + Oxidation (M)
☒	3717	703.3409	1404.6672	1405.6507	-0.9835	0	13	1.3e+02	1	DTTDAEMINLQR
☒	5083	465.5016	1857.9773	1858.8962	-0.9189	0	13	98	1	GSLSHFDDLFTTLGHGR
☒	5853	812.7501	2435.2285	2436.3297	-1.1013	0	13	69	1	LGLVEAEAIPQEEALASILLMAR
☒	7974	827.7552	4133.7396	4132.8381	0.9015	1	13	18	1	AHPTVGTCSGMFTANTMGSMSEALGMALPGSASPTATSSRR + 3 Oxidation (M)
☒	3682	695.6284	1389.2422	1389.6810	-0.4387	0	13	87	1	VLEMDISDLSPR + Oxidation (M)
☒	4138	496.6349	1486.8829	1485.8126	1.0703	1	13	1.2e+02	1	VLIGSMKIPGHYR + Oxidation (M)
☒	4696	832.4018	1662.7890	1662.8730	-0.0839	0	13	1.1e+02	1	GVINEGYWSTVLGLR
☒	6314	876.4196	2626.2370	2625.2679	0.9690	1	13	59	1	DRFMWTNGLLEMNETLVIQQR + 2 Oxidation (M)
☒	3998	725.3538	1448.6930	1448.6938	-0.0007	1	13	1.2e+02	1	MLMGKTMAEHLR + 2 Oxidation (M)
☒	4929	589.9242	1766.7508	1767.9367	-1.1859	0	13	95	1	EVQIPETITVGDLAQR
☒	5613	548.7340	2190.9069	2190.1314	0.7755	1	13	69	1	SNGMAILTELAKASSQSIPTR + Oxidation (M)
☒	8726	927.9782	5561.8255	5562.5281	-0.7025	0	13	8.4	1	RPNPAAATQEESLYASVEDMQTEDGVELNSWTPPEEDPQGETYAQVKPSR + Oxidation (M)
☒	5908	825.0637	2472.1693	2472.0389	0.1304	0	13	68	1	GAQGGESMMDSPEMIAMQQLSSR + 2 Oxidation (M)
☒	6730	718.1115	2868.4169	2868.4116	0.0053	1	13	55	1	VDPMAIVVFHQADIGEYVRHEETLT
☒	5332	498.0172	1988.0397	1987.0044	1.0353	1	13	91	1	VDASMKLIDQVPESNSVR
☒	5888	617.5386	2466.1253	2465.0983	1.0270	0	13	67	1	ENIDTSQGDVTFAGDGQEATPWK
☒	7554	615.6072	3687.5995	3686.7902	0.8094	1	13	27	1	TLNYGMGFVWSMNFLRGSNFIGLGYDDGTVVLK + Oxidation (M)
☒	3070	555.8475	1109.6804	1108.5369	1.1436	1	13	1.2e+02	1	AMQAAMKSGAK + Oxidation (M)
☒	4542	806.4767	1610.9388	1611.8443	-0.9055	1	13	1.1e+02	1	SNVIDAMLFVFGKR + Oxidation (M)
☒	4984	906.0021	1809.9896	1808.9097	1.0799	0	13	98	1	AWVSWEQTFNITVTK
☒	6284	523.4370	2612.1486	2613.3083	-1.1597	1	13	57	1	KLQFPVEYLFNQMMNNILNNK + Oxidation (M)
☒	3003	528.2475	1054.4804	1054.6134	-0.1330	0	13	1.4e+02	1	AINANAAAIVK
☒	4335	516.3009	1545.8809	1546.7483	-0.8675	0	13	1.2e+02	1	MTVIIPGMSAENER
☒	4875	868.9248	1735.8350	1735.8709	-0.0358	1	13	1e+02	1	LSCMANSVIKNLQSR + Oxidation (M)
☒	6146	512.0327	2555.1271	2554.3586	0.7685	1	13	60	1	FNILMGISCVTFMIIGSNILRR
☒	6408	445.3924	2666.3107	2665.3463	0.9644	1	13	62	1	MIMELTMNQKTWEQMLQILLR + Oxidation (M)
☒	6457	445.5568	2667.2971	2666.1604	1.1368	0	13	61	1	GFEVLHPMGWDAFGMPAENAAMEK + 2 Oxidation (M)
☒	7395	708.1282	3535.6046	3536.6513	-1.0467	1	13	34	1	LLMENPYDGPPVGMQEEAPGLKYSMEDLLER + Oxidation (M)
☒	5223	637.3160	1908.9262	1909.8363	-0.9102	1	13	95	1	EREEEPLFDIDSEMR + Oxidation (M)
☒	6148	512.0327	2555.1271	2555.2189	-0.0917	0	13	60	1	TYDIGIMPADVAINYGWSGPMLR + Oxidation (M)
☒	2683	458.7859	915.5572	914.5549	1.0023	0	13	1.9e+02	1	NVLLTLSR
☒	2966	513.7714	1025.5282	1025.6597	-0.1315	0	13	1.3e+02	1	NVVIITVLR
☒	3069	554.2845	1106.5544	1106.6084	-0.0539	1	13	1.5e+02	1	ANYEKILTR
☒	5660	741.9942	2222.9608	2222.2133	0.7475	0	13	74	1	DGAIAAALSFLAALTLMFR + Oxidation (M)
☒	6339	529.2641	2641.2841	2641.2934	-0.0093	1	13	63	1	WHVVMHDVVREMALWIASDFGK + Oxidation (M)
☒	2897	501.7937	1001.5728	1002.5498	-0.9769	1	13	1.7e+02	1	LAKWAAESK
☒	5556	533.5178	2130.0421	2130.2201	-0.1780	1	13	84	1	AILRAPVDLLFNGGIGTYIK
☒	5685	1130.0292	2258.0438	2257.9919	0.0520	1	13	76	1	SYCGIHDPACVVYCNTSKK
☒	7418	892.3986	3565.5653	3566.7021	-1.1369	0	13	33	1	GPDLQLGYVENGTMLLGSPNVVNIGDQNFMNTK + 2 Oxidation (M)
☒	8383	751.3287	4501.9285	4500.9765	0.9521	1	13	14	1	VASTMTDGASTMIEHDDTLPSQLGTMVINTEDEEEEGTMKR + 2 Oxidation (M)
☒	2309	647.2881	646.2808	646.3035	-0.0226	0	13	5.6	1	EGGGATR
☒	6356	531.0262	2650.0946	2650.2003	-0.1057	1	13	51	1	DMHIATLDDDGKLYMEINVDNGR + Oxidation (M)
☒	5367	673.1788	2016.5146	2015.9660	0.5485	1	13	59	1	TRSNQTEPDIANAPPYSR
☒	3531	667.5717	1333.1288	1333.6911	-0.5623	1	13	89	1	ELIAKATANMEK + Oxidation (M)

☒	4451	788.3925	1574.7704	1573.8610	0.9095	1	13	1.2e+02	1	SAILRSMNDALIVR + Oxidation (M)
☒	7394	708.1267	3535.5971	3535.7823	-0.1852	0	13	34	1	LLIEATLELMATGAVAAIQDMGAAGLTSSSVEMAGK + Oxidation (M)
☒	5070	926.4517	1850.8888	1851.9223	-1.0334	0	13	98	1	IFADMATGITVKPDAMR + Oxidation (M)
☒	5398	674.3391	2019.9955	2019.9935	0.0019	1	13	91	1	LFSGSLTRGMEVYTSGTAK + Oxidation (M)
☒	7703	775.1672	3870.7996	3869.9305	0.8691	1	13	28	1	LDEMGVERASVTLHVGAGTFKPIQGPVADHVMHAER + Oxidation (M)
☒	4074	369.6938	1474.7461	1473.5727	1.1734	0	13	1.3e+02	1	SWFEPMMEDMR + Oxidation (M)
☒	4945	593.6074	1777.8004	1777.9323	-0.1319	1	13	1e+02	1	LNEEGTLLDHKVTPGR
☒	5837	806.5714	2416.6924	2417.1571	-0.4647	0	13	50	1	SNELNLSGNSGALALQWSSGEQR
☒	5995	500.6285	2498.1061	2498.2224	-0.1162	1	13	66	1	GLINDPHMDGSFDVEAGLQIARK + Oxidation (M)
☒	6374	889.0656	2664.1750	2664.1690	0.0060	1	13	58	1	DGPRECSQQLGQEQELQMNMVR + 2 Oxidation (M)
☒	7224	835.4429	3337.7425	3336.6595	1.0830	1	13	40	1	RGRPSTHMIFGTEQTINSANFLLIDVMEK + 2 Oxidation (M)
☒	7313	864.6671	3454.6393	3453.5592	1.0801	1	13	37	1	EEGKPDDMTDDIADALKTIGEVANYWMSDPK
☒	2977	519.1362	1036.2578	1036.5012	-0.2433	0	13	1e+02	1	MPFTIDSAR
☒	3515	663.7668	1325.5190	1326.6424	-1.1234	1	13	1.2e+02	1	LIRDMFETMR + Oxidation (M)
☒	4997	453.5057	1809.9937	1810.9538	-0.9601	0	13	1e+02	1	GTLHGGQTATLTDVITAR
☒	7263	678.3206	3386.5666	3387.5963	-1.0296	1	13	38	1	QDGSALAATSFVARNLFEMTPEMLIADGASDK + 2 Oxidation (M)
☒	5917	825.7213	2474.1421	2475.2726	-1.1305	1	13	68	1	AVSEMNGKMLDNRPLYPVALAQR
☒	6043	631.2900	2521.1309	2520.1625	0.9684	0	13	64	1	GMVDMLNAQDDLYHVNLQGLDK + 2 Oxidation (M)
☒	7060	774.0438	3092.1461	3091.3823	0.7638	1	13	35	1	MSSTGSARTEGDSWDLASSVGATATMVAASR + 2 Oxidation (M)
☒	3354	630.9979	1259.9812	1259.6470	0.3342	0	13	98	1	ATVVAGVGTNDTR
☒	6686	572.5013	2857.4701	2857.4262	0.0439	1	13	56	1	GLAAGIAAHAAAGRAVLGVCGGFQMLCSR + Oxidation (M)
☒	5235	640.9835	1919.9287	1918.9355	0.9932	1	13	96	1	VMPMSASFTKSQSMFIK
☒	5686	754.1624	2259.4654	2260.1739	-0.7086	0	13	55	1	FETQLGTLAQFPNTLLGDPAK
☒	6916	599.9075	2994.5011	2993.6761	0.8251	1	13	52	1	ANAIAAAAAVAGVSVPAVASNLITSSDITKLK
☒	3072	556.2729	1110.5312	1109.6089	0.9223	1	13	1.3e+02	1	AVLVKMFMR + Oxidation (M)
☒	3105	570.8906	1139.7666	1140.5121	-0.7455	0	13	1.2e+02	1	EMDDYIIAR + Oxidation (M)
☒	5604	729.6840	2186.0302	2186.0563	-0.0261	1	13	87	1	SRGSLESIAEHVDASLSGSER
☒	5692	757.5597	2269.6573	2269.0692	0.5881	1	13	55	1	DSGCLSRDVGPAVVHCSAGIGR
☒	2978	519.1367	1036.2588	1035.5998	0.6591	1	13	1.1e+02	1	MSKTISLLK + Oxidation (M)
☒	4199	754.4214	1506.8282	1507.8181	-0.9898	1	13	1.2e+02	1	YAIILAAGQGTRMK + Oxidation (M)
☒	4529	802.8237	1603.6328	1603.6831	-0.0503	1	13	1e+02	1	LCRGDSSGHNVCDK
☒	4538	804.4770	1606.9394	1607.7950	-0.8556	1	13	1.1e+02	1	QPMGAAAGGQVHANRK + Oxidation (M)
☒	3580	679.8147	1357.6148	1357.7453	-0.1304	1	13	1.4e+02	1	KLAGLDLSDGEIK
☒	4837	862.9101	1723.8056	1724.8623	-1.0567	1	13	1.1e+02	1	VNKTTSKPVQMMSMK + Oxidation (M)
☒	4991	453.5054	1809.9925	1809.8455	0.1470	0	13	1e+02	1	ADELFSILMGEDVESR
☒	5034	608.9700	1823.8882	1824.8808	-0.9927	1	13	1.1e+02	1	SRHHGSLLTFFSHYH
☒	6582	558.2740	2786.3336	2786.3619	-0.0283	0	13	62	1	IAIVGSCFNQAMADALVSGTQETFLK + Oxidation (M)
☒	6838	732.5593	2926.2081	2926.4043	-0.1962	1	13	45	1	EEEGVEVEEEESAEVPLVETVVGGARR
☒	7894	1017.4801	4065.8913	4065.0576	0.8337	1	13	25	1	VEQSQLEGIETVGHVMELTLDLVARLATASAADKPEAAS + Oxidation (M)
☒	8122	714.8288	4282.9291	4283.9070	-0.9779	0	13	20	1	LQMEGEGGGETPEQPGLNGAAAAAAGAPDEAAEALGSADCELSAK + Oxidation (M)
☒	3208	584.8353	1167.6560	1166.5828	1.0733	0	13	1.4e+02	1	GISMAAMFVPK + Oxidation (M)
☒	3735	705.8891	1409.7636	1409.6133	0.1504	0	13	1.3e+02	1	DYMASQPDINEK
☒	7258	564.9404	3383.5987	3383.7336	-0.1349	1	13	39	1	ATGMKDGDFEKPIIAIANSFTQFVPGHVHLK + Oxidation (M)
☒	5092	621.9731	1862.8975	1861.9859	0.9116	0	13	1e+02	1	AISASLLMEQVLTPDFK
☒	5847	811.3616	2431.0630	2430.2478	0.8152	1	13	68	1	GMLTGPVTILQWSFVRNDQPR + Oxidation (M)
☒	6381	667.2723	2665.0601	2665.3720	-0.3119	0	13	48	1	ERPGVMFADMELIGVPHTIVIGER
☒	7586	745.7775	3723.8511	3725.0044	-1.1533	1	13	33	1	LLREFTVTLSVAIGISLLVSLTLTPMMCGWMLK + 2 Oxidation (M)
☒	5227	638.1576	1911.4510	1912.1002	-0.6492	1	13	65	1	HIAMVMTKAILVTLLSR + Oxidation (M)
☒	5306	656.2777	1965.8113	1965.9895	-0.1782	1	13	84	1	KDLTDLDFITIDNTNTK
☒	5828	1205.0256	2408.0366	2408.2600	-0.2234	1	13	69	1	LELYGSINPNHKLNHSFLANK
☒	3027	534.2529	1066.4912	1067.4593	-0.9681	0	13	1.4e+02	1	AGYAGEEMPK + Oxidation (M)
☒	4329	773.0847	1544.1548	1544.8960	-0.7411	1	13	85	1	LIGLITMSDLVARK + Oxidation (M)
☒	4342	387.7283	1546.8841	1547.7912	-0.9071	1	13	1.2e+02	1	LRALEMQSGLSGMR
☒	6178	858.0466	2571.1180	2571.3981	-0.2802	1	13	61	1	QYPTIAKTATEIINLEAIMNLPK
☒	3499	658.2935	1314.5724	1315.6481	-1.0756	1	13	1.5e+02	1	VNEDGDGVTRVR
☒	2677	913.5323	912.5250	913.5457	-1.0207	1	13	1.6e+02	1	AIQASIRR
☒	3153	575.8408	1149.6670	1148.6223	1.0447	1	13	1.5e+02	1	KVMAAAGGSTLK + Oxidation (M)
☒	4168	748.3270	1494.6394	1494.7864	-0.1470	0	13	1.2e+02	1	GADLKPAMIIHSDK
☒	6383	667.2726	2665.0613	2664.2887	0.7726	0	13	49	1	VIIAYGPSECTVGCTINNEIALDR
☒	6458	534.4689	2667.3081	2667.3465	-0.0384	1	13	65	1	MSSFVQELDKAFEGQLQVLLEEK
☒	7064	1035.7999	3104.3779	3105.3664	-0.9885	0	13	45	1	VWNQLMNTNASETVCYSQMASNLGMPK + 2 Oxidation (M)
☒	7700	774.9698	3869.8126	3869.9305	-0.1179	1	13	30	1	LDEMGVERASVTLHVGAGTFKPIQGPVADHVMHAER + Oxidation (M)
☒	2541	800.3515	799.3442	799.4552	-0.1110	1	13	1.4e+02	1	RADITPK
☒	3541	671.3469	1340.6792	1339.6186	1.0607	0	13	1.4e+02	1	LTDEMVMIMGGK + Oxidation (M)
☒	4289	770.2656	1538.5166	1539.6698	-1.1531	0	13	85	1	ALGLDCGPMSGFDGK + Oxidation (M)
☒	5576	430.6172	2148.0496	2149.0990	-1.0494	1	13	86	1	ILALHNMVQFSQSKDFQK + Oxidation (M)
☒	6710	574.4895	2867.4111	2868.3389	-0.9277	1	13	60	1	FFDSTTAYQGYGRGLDLDMLAEINR + Oxidation (M)
☒	8717	1391.2130	5560.8229	5559.7452	1.0777	1	13	8.9	1	MTSAVGTSGTAITSRVHSLNRPNMVSVGTIVWLSSELMFFAGLFAMYFTAR + 3 Oxidation (M)
☒	2616	436.7722	871.5298	872.5192	-0.9893	1	13	1.9e+02	1	ATLARSVR
☒	3009	528.2488	1054.4830	1053.5203	0.9628	1	13	1.4e+02	1	DEAEIKGHR
☒	3289	625.2803	1248.5460	1248.6714	-0.1254	0	13	1.5e+02	1	LGGDLGTYVINK
☒	6096	634.7875	2535.1209	2535.2956	-0.1747	0	13	66	1	DLTLSAEDIAASFQASVVEVLTEK
☒	5746	768.6993	2303.0761	2302.0947	0.9814	1	13	79	1	SNVSMKAMLEAGVHFGHQTSR + Oxidation (M)
☒	7398	1179.5426	3535.6060	3535.5456	0.0604	0	13	36	1	ATMAHANTSHPLYVAGMAGNTNHMSPWGEDAHK + 2 Oxidation (M)
☒	8159	861.6011	4302.9691	4302.8612	0.1079	1	13	21	1	MMQMLQGQGGGGGGGLPGLGGMDLQSMMSQMSGLMGGGGGGGGGRGR + 3 Oxidation (M)
☒	5842	810.3297	2427.9673	2427.2356	0.7317	0	13	60	1	DAIIVTTVSCIYGLGSPETYLR
☒	6104	848.0038	2540.9896	2540.1887	0.8009	1	13	53	1	RSTGLMSSFLEMGSSLFGVSNSSK + 2 Oxidation (M)
☒	8924	1225.7888	6123.9076	6124.0281	-0.1204	1	13	6.8	1	ILGLGDQGIGGMGIPIGKLALYTACGGISPAYCLPILLDVGTNNQQLLDDPMYMGWR + 3 Oxidation (M)
☒	3667	693.3677	1384.7208	1383.7946	0.9262	1	13	1.4e+02	1	AAVIAVTRSAQAAR
☒	5730	767.0369	2298.0889	2298.1314	-0.0426	1	13	80	1	IAFRETFMVAALSGDGVDDLR + Oxidation (M)
☒	6536	684.0514	2732.1765	2733.2812	-1.1047	0	13	54	1	MSTISEDTVWIEVGPHAVCTNMIK + Oxidation (M)
☒	8621	937.1998	4680.9626	4679.7674	1.1952	0	13	12	1	LMGGMGMGGMGGLEGLGGMGGMGGMGGMGGMGGMEEFEDSDDEEETAK + 2 Oxidation (M)
☒	2604	430.2627	858.5108	857.4719	1.0389	1	13	2.1e+02	1	QPVSSGKR
☒	3574	678.0921	1354.1696	1353.7980	0.3717	0	13	89	1	GIVLASALVENLR
☒	7645	764.3862	3816.8946	3816.7434	0.1512	1	13	31	1	LLCGGEALKGDGFDNGAWVAPTVFTDCSDEMTIVR + Oxidation (M)
☒	2983	520.7678	1039.5210	1038.6114	0.9097	0	13	1.4e+02	1	SLVVSLFFK
☒	3381	640.3868	1278.7590	1279.6343	-0.8752	1	13	1.4e+02	1	QAMFSGEKINR
☒	3560	674.6929	1347.3712	1346.6612	0.7100	1	13	96	1	AVVLDDQMERR + Oxidation (M)
☒	4816	567.9329	1700.7769	1699.8491	0.9278	1	13	1.2e+02	1	LAKEYGLPFMETSAK + Oxidation (M)
☒	4874	868.9186	1735.8226	1735.8709	-0.0482	1	13	1.1e+02	1	LSCMANSVIKNLQSR + Oxidation (M)
☒	6484	538.8853	2689.3901	2690.2952	-0.9050	0	13	64	1	QFAFPVKPHWDLGVAMDGLDFER + Oxidation (M)
☒	6989	751.3546	3001.3893	3002.2863	-0.8970	0	13	52	1	IEDLPTTEEDEEEMDDFFSNLVNQK + Oxidation (M)
☒	8488	648.0037	4528.9750	4529.9520	-0.9770	1	13	17	1	ALSEEMGDTLEEGSASPTSPDCSLDSPGPEKMALAFTEQEER + 2 Oxidation (M)
☒	3364	635.1118	1268.2090	1268.6473	-0.4382	0	13	94	1	ATLQNNLPNER
☒	6169	856.0369	2565.0889	2566.1073	-1.0184	1	13	61	1	MSGTPRTMPPLMMDWAEQPNGR + 4 Oxidation (M)
☒	6542	917.0850	2748.2332	2749.4122	-1.1791	1	13	59	1	QAMLFPQPAQPGDAFHQVLNGLARK + Oxidation (M)
☒	3545	672.3175	1342.6204	1342.7609	-0.1404	0	13	1.4e+02	1	LFGPATAILNQAK
☒	4308	770.5735	1539.1324	1539.6698	-0.5373	0	13	84	1	ALGLDCGPMSGFDGK + Oxidation (M)
☒	5664	1115.7776	2229.5406	2230.2620	-0.7213	1	13	59	1	VVRAMQVHKPILLEGSPGVGK + Oxidation (M)
☒	8200	1092.0133	4364.0241	4365.0466	-1.0225	1	13	22	1	FNIPCDAAQVYAGDDMSIDYVASVKPSFNMIEHLMKLK + 3 Oxidation (M)
☒	6097	508.2300	2536.1136	2536.1879	-0.0743	1	13	66	1	IGGKAGVMWDVPAGESHMGYPAYK + Oxidation (M)

⊠	4332	773.6951	1545.3756	1545.7821	-0.4064	1	13	88	1	NEATVPSMIGVEKR + Oxidation (M)
⊠	6392	889.6906	2666.0500	2666.3441	-0.2942	1	13	51	1	MDVIRTAIGLMSGTSMDGIDVALIR + 2 Oxidation (M)
⊠	6512	679.0720	2712.2589	2711.1682	1.0907	0	13	63	1	DSAQLSDSGSADEVDEFEIQDADIR
⊠	6685	572.3010	2856.4686	2857.4262	-0.9576	1	13	61	1	GLAAGIAAHAAAGRAVLGVCGGFQMLCSR + Oxidation (M)
⊠	3519	664.2982	1326.5818	1327.5934	-1.0116	1	13	1.4e+02	1	TKGSMVCEICK + Oxidation (M)
⊠	5833	805.6879	2414.0419	2415.1636	-1.1218	1	13	71	1	APLLYSQSWLTMAKMCLADGK + 2 Oxidation (M)
⊠	6704	956.8102	2867.4088	2868.3820	-0.9732	1	13	62	1	LVGQAIETFNMIEDGDRLMVCLSGGK + Oxidation (M)
⊠	4539	806.2021	1610.3896	1609.8100	0.5796	0	13	81	1	GGYFSVNLNEEILR
⊠	4911	875.9871	1749.9596	1750.8520	-0.8923	1	13	1.1e+02	1	TASLRDMADVTVETAR + Oxidation (M)
⊠	7140	648.2897	3236.4121	3236.5799	-0.1678	1	13	41	1	LDMDGDGQVDFEEFVTLLGPKLSTSGIPEK
⊠	8088	1062.5063	4245.9961	4245.1371	0.8590	1	13	24	1	AIVIVALDNLTKGSSGQALQNANLMLGEDETAGLMMAPLFP + 2 Oxidation (M)
⊠	6106	509.2365	2541.1461	2540.2331	0.9130	1	13	69	1	EFLPYLQELEKMPESLMHFK + 2 Oxidation (M)
⊠	6515	680.8124	2719.2205	2720.3414	-1.1209	1	13	61	1	MILSWLSEPTMAGLQKTQHNFTR + 2 Oxidation (M)
⊠	6525	909.0928	2724.2566	2724.2964	-0.0399	0	13	63	1	AITGYNAVGGSNADTNGHGTHVAGTIGGR
⊠	7177	822.1146	3284.4293	3284.6203	-0.1910	1	13	41	1	ATADYIGMIATVMNAMTLQDSLERIGVQTR + Oxidation (M)
⊠	7597	932.2207	3724.8537	3725.7270	-0.8733	1	13	34	1	AGMDRVQADYTGMLGTVMNALVMADALQHAGVDTR + 3 Oxidation (M)
⊠	4322	771.7801	1541.5456	1540.7957	0.7499	1	13	97	1	GPQTAGELRLNTER
⊠	8373	642.1432	4487.9515	4487.2498	0.7016	1	13	17	1	LMGSAHMVAILQSTLRSSILSWGLNDVAGTNTTMSDPELLNK + Oxidation (M)
⊠	3984	722.8254	1443.6362	1443.8310	-0.1948	1	13	1.3e+02	1	LHIRTVPLGEGPR
⊠	5488	686.9228	2057.7466	2057.1190	0.6275	1	13	67	1	SLGIQPDAIVLRTEMAISK + Oxidation (M)
⊠	5540	704.4035	2110.1887	2110.1456	0.0431	0	13	85	1	LAMVAGEPSGDLLAASLLGGLR
⊠	6439	667.5855	2666.3129	2665.2150	1.0979	0	13	68	1	NHGNNEADAALQGLAQQGVDMEDLR
⊠	6450	534.2703	2666.3151	2665.3381	0.9770	0	13	68	1	VAAGALEVVDALANQAAEHSSTIMPGK + Oxidation (M)
⊠	6995	752.8381	3007.3233	3007.2160	0.1073	1	13	50	1	DMGLVNSGTKCGEGMVCSNGECIEMEK + Oxidation (M)
⊠	5573	716.6914	2147.0524	2147.0319	0.0205	1	13	92	1	EIFPNTLLAEDFMVYKMA + Oxidation (M)
⊠	5971	500.4271	2497.0991	2496.1156	0.9836	1	13	69	1	RTDCGTIQGISVNTQNGMMPER + 2 Oxidation (M)
⊠	7383	590.1075	3534.6013	3535.7691	-1.1677	1	13	38	1	ELTVANPAVLGTNMGPVNDQAAFDKVMSYVAIGK + Oxidation (M)
⊠	5570	1071.5514	2141.0882	2141.2104	-0.1222	0	13	91	1	MWQLLLPTALLLLVSAGMR + Oxidation (M)
⊠	6646	710.8112	2839.2157	2838.4514	0.7643	0	13	54	1	DKPFFEDLIEYVTSGPVVVMIVEGR
⊠	6914	998.4462	2992.3168	2992.5845	-0.2677	0	13	50	1	LHATILTVFPEMFPGTLGHSLAGQALQK + Oxidation (M)
⊠	8174	623.1344	4354.8899	4354.9633	-0.0734	1	13	18	1	QDNKPEMDDNQLGEPQEGLVISRFGQHADVEAEDGSTQR + Oxidation (M)
⊠	2771	492.5888	983.1630	982.5196	0.6435	0	13	97	1	HLDQTLTR
⊠	3257	606.8696	1211.7246	1211.5526	0.1721	1	13	1.4e+02	1	MMDALAASDKK + 2 Oxidation (M)
⊠	4713	836.3624	1670.7102	1669.8570	0.8533	1	13	1.1e+02	1	MATKTNHIAVVAENR + Oxidation (M)
⊠	2454	371.0998	740.1850	741.3770	-1.1919	0	13	20	1	GPQPSTR
⊠	2543	801.2048	800.1975	799.4664	0.7311	1	13	1.1e+02	1	RSVVPSR
⊠	5057	615.0016	1841.9830	1842.9840	-1.0010	1	13	1.1e+02	1	VLFTPDDVKNDVGVGLR
⊠	6171	856.3898	2566.1476	2567.1632	-1.0157	0	13	69	1	TDGLTPVEMQNLEPGTCYQVSGR + Oxidation (M)
⊠	6706	574.4894	2867.4106	2866.5058	0.9048	0	13	63	1	MLHPVAGRPMVQHVVDQLQQVNLNK + Oxidation (M)
⊠	3540	671.3452	1340.6758	1340.6581	0.0178	1	13	1.4e+02	1	VGQAMMELYRK + Oxidation (M)
⊠	4040	734.3687	1466.7228	1465.7525	0.9703	0	13	1.3e+02	1	TGTGTTSLFGQQIR
⊠	6361	663.5505	2650.1729	2649.2601	0.9128	1	13	63	1	SEYLKGMGVPCLMAVHQDPSGITK + 2 Oxidation (M)
⊠	6375	889.1143	2664.3211	2664.2411	0.0800	0	13	69	1	SNMTSPTLLDANPMENPALFNDIK + 2 Oxidation (M)
⊠	2684	458.7862	915.5578	914.4531	1.1047	0	13	2.1e+02	1	LAAPPMDGK + Oxidation (M)
⊠	3719	703.6066	1405.1986	1404.7613	0.4374	1	13	99	1	LSLEQQLFDGKK
⊠	6504	678.0375	2708.1209	2708.2898	-0.1689	1	13	56	1	NSPTCKYQVLSSGEGGEIICGVPTR
⊠	7141	1079.9910	3236.9512	3236.3493	0.6018	1	13	35	1	QCMMHFSDAAEAYIEMRNLDEPYMPR + 2 Oxidation (M)
⊠	7691	643.3138	3853.8391	3852.6705	1.1686	1	13	33	1	YFEYSMYYSLIDSLAAEHSARMQAMDNATNNAK + 3 Oxidation (M)
⊠	2457	746.3433	745.3360	745.3831	-0.0471	1	13	95	1	RGAEGTR
⊠	5696	759.6739	2275.9999	2275.1096	0.8903	0	13	82	1	SAMATEHFPPVTAPEFAFLGR
⊠	6195	862.3818	2584.1236	2584.2340	-0.1104	0	13	67	1	QNNSNTLLGGMSPSEAAVQWPLDR
⊠	6711	956.8110	2867.4112	2868.4116	-1.0005	1	13	64	1	VDPMAIVVFHQADIGEYVRHEETLT
⊠	7763	794.5861	3967.8941	3967.7299	0.1642	0	13	29	1	AFEETQIDIPGGGSHGGSSMSLWEVMQSNMIPEDQR + 3 Oxidation (M)
⊠	3584	680.5333	1359.0520	1359.6266	-0.5746	0	13	1e+02	1	SEAGLSAEGPEGTR
⊠	4531	535.6448	1603.9126	1604.7100	-0.7975	0	13	1.2e+02	1	MTNSSIAEETHDVR + Oxidation (M)
⊠	5058	461.5031	1841.9833	1842.0146	-0.0313	1	13	1.1e+02	1	VVLRSATAGTGIIAGGPMR + Oxidation (M)
⊠	2632	888.1668	887.1595	886.4760	0.6835	0	13	1.5e+02	1	ENGEVVLK
⊠	3550	672.8293	1343.6440	1343.7660	-0.1220	0	13	1.5e+02	1	TLGSPITLTVTNK
⊠	7074	780.6077	3118.4017	3117.5918	0.8099	1	13	49	1	TVQVVPHITDAVQEWVMNQAKVPVDGNK + Oxidation (M)
⊠	3547	448.5571	1342.6495	1343.7197	-1.0703	1	13	1.5e+02	1	RNPGADIIFDVK
⊠	6826	731.3352	2921.3117	2920.5528	0.7589	1	13	56	1	VVGLAHFTVGYFLTYAAFLIASTAGKFG
⊠	4556	540.5353	1618.5841	1617.8362	0.7478	1	13	97	1	IVAFGDTSPEGLREK
⊠	6727	574.6896	2868.4116	2868.3820	0.0296	1	13	63	1	LVGQAIETFNMIEDGDRLMVCLSGGK + Oxidation (M)
⊠	3091	562.3412	1122.6678	1123.5617	-0.8939	0	13	1.5e+02	1	DILMSITMGK + Oxidation (M)
⊠	3274	411.2069	1230.5989	1230.6204	-0.0215	1	13	1.7e+02	1	AEGIDKNDLTR
⊠	5602	1091.5488	2181.0830	2180.0276	1.0555	1	13	92	1	MIGGQEDMIVDVALDMAKGR + 2 Oxidation (M)
⊠	5640	736.4973	2206.4701	2206.1126	0.3575	1	13	63	1	AVEASAVSNMLVAGLMAKDWK + Oxidation (M)
⊠	8464	1132.9995	4527.9689	4528.9985	-1.0296	1	13	18	1	NDNAQGEYYLPDVIGIMKDAGEIVAAYEMADFSESMGVNDR + 2 Oxidation (M)
⊠	8728	1113.3729	5561.8281	5561.6164	0.2117	1	13	9.6	1	MTDNDALSLPPDVFAAEDLAAEIDEETSCVVVQTPDAFGNLRDLSPLAAACK
⊠	3248	601.7076	1201.4006	1201.6125	-0.2119	0	13	1.3e+02	1	ISGPMAGVAIDR + Oxidation (M)
⊠	4035	733.2273	1464.4400	1464.7177	-0.2777	1	13	96	1	MMGRSLVDIQQR + 2 Oxidation (M)
⊠	5605	729.7424	2186.2054	2187.0655	-0.8601	1	13	91	1	IDIDDDGTIKVASVDADAAQR
⊠	4877	435.1758	1736.6741	1737.8719	-1.1979	1	13	97	1	KYEGQATQVGEIIMR + Oxidation (M)
⊠	8922	1225.5908	6122.9176	6122.1861	0.7316	1	13	7.4	1	SVTYSISLSLTMNKHQGHSLLQTVSGLNYHTLSVGTGLSYVSLIIFDLPSNLLMTR + Oxidation (M)
⊠	2727	467.2998	932.5850	933.4516	-0.8665	1	13	2e+02	1	SSSKTPDGR
⊠	4059	738.1102	1474.2058	1473.6817	0.5242	1	13	94	1	RMETPSSMHGGIR + Oxidation (M)
⊠	4247	764.8217	1527.6288	1527.6848	-0.0560	1	13	1.2e+02	1	ENQRQHEAVDMR + Oxidation (M)
⊠	4480	795.3730	1588.7314	1588.8793	-0.1478	1	13	1.3e+02	1	GLTRGLMLLNMLNK + Oxidation (M)
⊠	6335	880.7029	2639.0869	2640.1510	-1.0642	1	13	58	1	DQYGNPMEGPTDSYGVQRAPSAER + Oxidation (M)
⊠	7114	799.1111	3192.4153	3193.4718	-1.0565	1	13	46	1	GMNVLHPMGWDAFGMPAENAAMQNKVHPK + Oxidation (M)
⊠	8349	895.5989	4472.9581	4473.0311	-0.0730	1	13	18	1	IDPDSSIAAALMDMYQSMGDALAQQYGGSAAHNTVFPERQGK + 2 Oxidation (M)
⊠	3075	559.2333	1116.4520	1117.6277	-1.1757	1	13	1.7e+02	1	AKIAIVGMSGR + Oxidation (M)
⊠	3202	582.9225	1163.8304	1164.6251	-0.7946	0	13	1.2e+02	1	LEAEGHGALIR
⊠	5015	453.7568	1810.9981	1810.8697	0.1284	1	13	1.1e+02	1	YNSDSEKITQTLQER
⊠	6320	526.6357	2628.1421	2628.2788	-0.1367	1	13	65	1	CGILSSGNNLFFNEDGLRMLVTR + Oxidation (M)
⊠	6708	574.4894	2867.4106	2868.3820	-0.9714	1	13	65	1	LVGQAIETFNMIEDGDRLMVCLSGGK + Oxidation (M)
⊠	2382	701.3253	700.3180	701.4072	-1.0891	1	13	40	1	AATKSPK
⊠	3006	528.2485	1054.4824	1054.5407	-0.0582	0	13	1.6e+02	1	ALNNIEPER
⊠	4761	564.6077	1690.8013	1690.8196	-0.0183	1	13	1.2e+02	1	EKPNVRTETLDMDK + Oxidation (M)
⊠	4528	802.7539	1603.4932	1603.7801	-0.2869	1	13	88	1	KANAEGTTSQEIAER
⊠	8091	850.4045	4246.9861	4247.2321	-0.2459	1	13	25	1	ELLDVLSVLSLMERNSPVVISPLLMNLTVENDFSTTVK + 2 Oxidation (M)
⊠	8207	730.6851	4378.0669	4379.2359	-1.1690	1	13	22	1	VSVFVEVEGAGDFLPPYAGNLDIMTAAATKVGEVIAGQITSAR + Oxidation (M)
⊠	8461	647.7176	4526.9723	4527.9874	-1.0151	1	13	18	1	VASTMTDGANTMIEHDDTLPSQLGTMVINTEDEEEEGTMKR + 2 Oxidation (M)
⊠	3369	425.5322	1273.5748	1274.6653	-1.0905	1	13	1.6e+02	1	VLSAGCGETIRL
⊠	4364	389.7075	1554.8009	1555.7269	-0.9260	1	13	1.3e+02	1	VCSVLMGCRTTTR + Oxidation (M)
⊠	6413	534.2695	2666.3111	2665.2150	1.0961	0	13	72	1	NHGNNEADAALQGLAQQGVDMEDLR
⊠	6556	553.8361	2764.1441	2763.3836	0.7605	1	13	55	1	GRTPLHVSIMAGNEYVFSQLLQCK + Oxidation (M)
⊠	7771	664.2977	3979.7425	3980.0760	-0.3334	1	13	27	1	LVTDSLSMMLIVAFLIALVGHYLMGGIRAGNQMTGQK + 2 Oxidation (M)
⊠	5005	604.6717	1810.9933	1809.8349	1.1584	1	13	1.1e+02	1	MVEARAESMSTQEAVR + Oxidation (M)
⊠	5233	640.9828	1919.9266	1918.9571	0.9695	0	13	1.1e+02	1	SLPFSMSHISSASATAAVR

☒	4733	841.9720	1681.9294	1682.7967	-0.8673	1	13	1.2e+02	1	TSDELRDTMSLMIR + Oxidation (M)
☒	6623	705.0781	2816.2833	2815.4228	0.8605	1	13	64	1	AINLDTSNLPFKGHIAFQHMYNLR + Oxidation (M)
☒	7331	696.5228	3477.5776	3476.5889	0.9887	1	13	40	1	EIETTLRQNAGLIAEQFLDDGGPEEDAAGAAEM + Oxidation (M)
☒	3037	541.7601	1081.5056	1081.3957	0.1099	0	12	1.5e+02	1	WDEEGMCR
☒	4149	746.5250	1491.0354	1490.7035	0.3320	1	12	97	1	ERNVMQETEVEK
☒	5566	713.9998	2138.9776	2139.9612	-0.9836	1	12	93	1	AYRAMATTHEQNTMAMSAR
☒	5672	749.7046	2246.0920	2245.1500	0.9420	1	12	94	1	SIHSIGLMWWMLSREVLR + 2 Oxidation (M)
☒	4674	824.7744	1647.5342	1647.6934	-0.1591	0	12	89	1	ASTSAPDSSGPEPMVEA + Oxidation (M)
☒	5503	1033.9937	2065.9728	2065.8912	0.0817	1	12	1e+02	1	SSYDMYHLSREGEAHER
☒	6490	540.4658	2697.2926	2697.2480	0.0447	1	12	71	1	MREIVTLQLDQESYFTYSSDEK + Oxidation (M)
☒	3346	628.4746	1254.9346	1254.5802	0.3545	0	12	1.1e+02	1	EMDDYNLVLK + Oxidation (M)
☒	4796	424.2079	1692.8025	1693.8378	-1.0353	1	12	1.2e+02	1	ILSEQKAMINAMDSK + Oxidation (M)
☒	5452	682.6313	2044.8721	2045.0542	-0.1821	1	12	92	1	RPGTPASDLPDTTPEHVKK
☒	8360	898.1986	4485.9566	4486.1937	-0.2371	1	12	18	1	MTVVTITPGAMSFADWRAIYEGASAALTVGAWDAIDASAAAVAR + 2 Oxidation (M)
☒	4057	737.9645	1473.9144	1472.7623	1.1521	0	12	1.3e+02	1	AWADLIESTLAQR
☒	4182	751.1045	1500.1944	1499.7766	0.4179	1	12	95	1	MGLEGPTAKEVVNR
☒	6585	933.1156	2796.3250	2796.4303	-0.1053	1	12	70	1	TAFDILMSGEATPSQIGGFLMALRVR + Oxidation (M)
☒	4813	567.2533	1698.7381	1697.8948	0.8433	1	12	1.2e+02	1	GPRSGIILTNDEDLAK
☒	5284	649.9498	1946.8276	1946.1200	0.7075	1	12	1e+02	1	SGLSADLLAKHLPIDVVAK
☒	6139	512.0322	2555.1246	2554.1976	0.9270	1	12	70	1	VIFLGDYAYGGETTIDGYSSDRR
☒	6354	663.5309	2650.0945	2649.2679	0.8266	1	12	59	1	GCLIAQPYDTEKGAGTMSPHTFLR
☒	6852	588.7041	2938.4841	2938.4092	0.0749	0	12	63	1	LADDMFETMYDAPGIGLAAIQIAEPVR + 2 Oxidation (M)
☒	7210	555.4391	3326.5909	3326.5033	0.0876	1	12	46	1	KIVMFLDYDGTLSPIVDDPDSAFMSDTMR + 3 Oxidation (M)
☒	7845	808.5609	4037.7681	4036.8830	0.8852	1	12	26	1	GNLSCCSSITGLILEHSGENGNKTVYPSGGNMVNLWR + Oxidation (M)
☒	8659	802.5442	4809.2215	4808.2010	1.0205	1	12	16	1	ALQEAKCDVDFSGSEFVATINDNFVANAMAAFAPYLAEATGDSHK + Oxidation (M)
☒	3837	715.3745	1428.7344	1427.6602	1.0742	1	12	1.5e+02	1	MTEEEKQSVFGK + Oxidation (M)
☒	6728	718.1102	2868.4117	2868.4116	0.0001	1	12	66	1	VDPMAIVVFHQADIGEYVRHEETLT
☒	3104	570.2932	1138.5718	1137.5852	0.9866	1	12	1.6e+02	1	LELDFAKMR + Oxidation (M)
☒	3615	455.2285	1362.6637	1362.7581	-0.0944	1	12	1.5e+02	1	DGFTMTKLLPLK
☒	5405	506.0106	2020.0133	2020.0048	0.0085	0	12	1.1e+02	1	EAGQNIVIYEGGVVMAQAR + Oxidation (M)
☒	5858	812.7513	2435.2321	2436.1712	-0.9391	1	12	83	1	SMLDRLSEAFQMALISMNAHR + Oxidation (M)
☒	7090	786.8414	3143.3365	3143.3382	-0.0017	1	12	45	1	KDLSYSMMNNNDHVYDPNGMIEAADQR + Oxidation (M)
☒	4947	889.9098	1777.8050	1778.8873	-1.0822	0	12	1.2e+02	1	MAIAESGAFEINVNLGK + Oxidation (M)
☒	5690	567.2579	2265.0025	2264.1549	0.8476	1	12	87	1	LNTFDDKAQALGALQNVQYR
☒	6330	660.0339	2636.1065	2635.4077	0.6988	1	12	63	1	QAPDVIMVSLDMSPHLVKQLSLAK + Oxidation (M)
☒	7315	692.5342	3457.6346	3458.6844	-1.0497	1	12	45	1	LIAVDNTGAIVDGDSMMAICGTNLNEKHILNK + 2 Oxidation (M)
☒	5361	670.4166	2008.2280	2008.9814	-0.7534	1	12	83	1	ATDGLALSSRNGYLSAEER
☒	6233	522.8648	2609.2876	2608.1819	1.1058	1	12	75	1	ASENAEVDIPQAMVDTELDRMMK + Oxidation (M)
☒	6620	939.4594	2815.3564	2814.5783	0.7781	1	12	68	1	GITPEAIWSLILFVGIKETDATISLK
☒	8609	779.1883	4669.0861	4670.2679	-1.1818	1	12	17	1	HVTMNLPAIAVEFLDVFQPLSSTCAPASEQRCNASAAAVVNER
☒	2370	687.4281	686.4208	686.3711	0.0497	0	12	31	1	AGVVDAR
☒	4751	562.6094	1684.8064	1684.8468	-0.0404	1	12	1.3e+02	1	RVGSVPHAGFGMGLER + Oxidation (M)
☒	5002	906.3990	1810.7834	1809.9771	0.8064	0	12	1.1e+02	1	MSSVLERPAAPAILPSR + Oxidation (M)
☒	5494	1031.8840	2061.7534	2061.9612	-0.2077	0	12	74	1	IQTVSFAGGPMPQNSPMER + Oxidation (M)
☒	6358	663.5316	2650.0973	2651.2391	-1.1418	0	12	60	1	NPFILDSEEPEEDFQAFLAGEVR
☒	6837	976.1221	2925.3445	2924.3251	1.0194	0	12	63	1	AVIGFAMTVSSMSCCIFVSEMVTPER + Oxidation (M)
☒	5829	603.0165	2408.0369	2409.1780	-1.1411	1	12	79	1	KHMYANILLSGGNTMLSGISDR + 2 Oxidation (M)
☒	6385	534.0196	2665.0616	2665.2914	-0.2298	1	12	55	1	EVIAFPKTQSAACVMTQAPGMVDAK + Oxidation (M)
☒	6463	668.0808	2668.2941	2669.2697	-0.9756	0	12	74	1	VVVQGIWVGGWCWGEDVPNVEER
☒	6630	705.7716	2819.0573	2819.5150	-0.4577	1	12	48	1	GSQISMLAQLGVPVRVPITTNLMHNK + Oxidation (M)
☒	6932	600.1085	2995.5061	2995.5232	-0.0171	1	12	61	1	QFPWAVQTWYDSIGAINKIQDFLQK
☒	7625	756.1428	3775.6776	3775.8994	-0.2218	1	12	33	1	FGGSTRQQTMGMTLGALTFAVIVFFFTDPVYTLK + 2 Oxidation (M)
☒	3246	599.8683	1197.7220	1197.6064	0.1157	1	12	1.7e+02	1	SLSGMKAFVDK + Oxidation (M)
☒	4006	726.3621	1450.7096	1450.7351	-0.0254	1	12	1.5e+02	1	MQTSFSPATRLGR
☒	8480	755.6708	4527.9811	4527.9874	-0.0062	1	12	19	1	VASTMTDGANTMIEHDDTLPSQLGTMVINTEDEEEEGTMKR + 2 Oxidation (M)
☒	4939	593.1065	1776.2977	1776.8750	-0.5773	0	12	85	1	ADMLINMLEEVNITR + Oxidation (M)
☒	5435	680.6378	2038.8916	2040.0714	-1.1798	0	12	1e+02	1	KPTTNYVIATVMAFNAGVK + Oxidation (M)
☒	5656	741.0287	2220.0643	2220.1208	-0.0565	1	12	95	1	NNDALGSKQAMPIYLASLNGK + Oxidation (M)
☒	4760	423.7075	1690.8009	1691.7859	-0.9850	0	12	1.3e+02	1	CDSVISLSDLTMAHK + Oxidation (M)
☒	5198	632.9344	1895.7814	1895.8782	-0.0969	1	12	1e+02	1	TSLGEVSKGDTMENLDGK + Oxidation (M)
☒	6108	636.2949	2541.1505	2540.2983	0.8522	1	12	76	1	DQLEELQGLLQSWNERVNLTR
☒	4797	424.2164	1692.8365	1693.8231	-0.9866	1	12	1.3e+02	1	SLLASTGNNSDSSAKSR
☒	5086	620.6672	1858.9798	1859.9993	-1.0195	1	12	1.2e+02	1	FKALDLASKPEGTGTPTK
☒	5476	685.6260	2053.8562	2053.0731	0.7830	0	12	93	1	IVINYNSAPTALGYSIETK
☒	6242	522.8654	2609.2906	2608.1819	1.1088	1	12	77	1	ASENAEVDIPQAMVDTELDRMMK + Oxidation (M)
☒	2549	803.3882	802.3809	803.3410	-0.9600	0	12	1.9e+02	1	GDQGNEGK
☒	4462	790.2590	1578.5034	1578.7889	-0.2854	1	12	95	1	EEAPKAEAAPAAEAPK
☒	6540	912.3981	2734.1725	2734.2365	-0.0641	0	12	60	1	AHGTAVGLPSEDDMGNSEVGHNALGAGR + Oxidation (M)
☒	7236	671.2966	3351.4466	3351.4033	0.0433	1	12	41	1	VSGQVGSRSPSMISNDSETLEECMVCSDMK + 2 Oxidation (M)
☒	8418	1129.7804	4515.0925	4514.1644	0.9281	1	12	21	1	SGQMMLAQGLLLHLMPTDWRWSDCHALTDVDFEVLKPR + 3 Oxidation (M)
☒	3103	568.8516	1135.6886	1135.5768	0.1119	1	12	1.8e+02	1	VGIMGRSASSR + Oxidation (M)
☒	3831	713.8176	1425.6206	1426.6551	-1.0344	0	12	1.5e+02	1	LPANGFYMQNEK + Oxidation (M)
☒	5688	566.9872	2263.9197	2265.0556	-1.1359	1	12	80	1	MGRGGDSSGQAHPAAELAVPSDR
☒	5166	627.9769	1880.9089	1880.9965	-0.0876	1	12	1.1e+02	1	LQPPAVVPMELRTVCR + Oxidation (M)
☒	3744	707.9874	1413.9602	1413.7398	0.2205	1	12	1.2e+02	1	RIEAPMQLAQNK + Oxidation (M)
☒	4695	831.3241	1660.6336	1661.7897	-1.1560	0	12	1.1e+02	1	GEGEFDVATIDKPER
☒	5811	600.9884	2399.9245	2399.3285	0.5960	1	12	66	1	SSRGSVVSRPVSSFKPISPNIAK
☒	3746	708.5839	1415.1532	1415.7772	-0.6240	0	12	1.1e+02	1	NSSLILSLHAFSK
☒	5927	828.7476	2483.2210	2484.3522	-1.1313	1	12	86	1	MHPTAARTGVLLINLGTPEAPTPK
☒	6060	632.2872	2525.1197	2526.2748	-1.1551	1	12	74	1	VSTDQESGQTILRGMGELHLDIK
☒	6389	667.3338	2665.3061	2664.3008	1.0053	1	12	75	1	VNVQSPHRAMAALGLMLTCMYTGK + Oxidation (M)
☒	7030	760.5895	3038.3289	3038.4929	-0.1640	1	12	55	1	AIQRAATIVWNGPMGVFEMKPFCNGTK + Oxidation (M)
☒	5013	604.6730	1810.9972	1811.8117	-0.8145	1	12	1.2e+02	1	MWDVRTDMCVATVGR + Oxidation (M)
☒	4834	860.5326	1719.0506	1718.8727	0.1780	1	12	1.1e+02	1	EGDKLGFLVPAETTDK
☒	5404	674.3450	2020.0132	2021.0694	-1.0563	1	12	1.1e+02	1	VNFGSRKPAPQTTYQVTK
☒	3505	660.5443	1319.0740	1319.7132	-0.6392	1	12	1.1e+02	1	RVLGLMPHPER + Oxidation (M)
☒	6774	722.5875	2886.3209	2887.3117	-0.9908	1	12	63	1	EKGCIMLGEFSDGPDTHDEVVNPIGR + Oxidation (M)
☒	7260	484.3792	3383.6035	3384.6256	-1.0221	0	12	47	1	GHEAGQVADCDVVVYSSAVRPDANVEISEALK
☒	8672	969.4654	4842.2906	4841.5665	0.7241	1	12	18	1	STFLNHLIPFDDNINFHKLIAVAIAVISLLHTALHMLCNYPR
☒	3988	723.3630	1444.7114	1443.6929	1.0186	1	12	1.6e+02	1	VMRDFVGSYAAGR + Oxidation (M)
☒	4317	771.3002	1540.5858	1540.8249	-0.2391	1	12	1.2e+02	1	LQSSYLFSEIAKR
☒	4809	848.1981	1694.3816	1693.8556	0.5260	1	12	88	1	SISTSDQMLQKLSQL + Oxidation (M)
☒	2778	493.8312	985.6478	985.5080	0.1398	0	12	2e+02	1	AASLLDDPGK
☒	3506	660.6962	1319.3778	1318.7245	0.6534	1	12	1.1e+02	1	KLLAYGADIEAR
☒	5229	639.6484	1915.9234	1915.1142	0.8091	1	12	1.2e+02	1	TPLRIVPEPTGTPLLSPK
☒	6064	506.8246	2529.0866	2528.2628	0.8238	1	12	73	1	AMQAALDSVVCHTPLNNLGFSRK
☒	6731	574.6907	2868.4171	2867.5770	0.8401	1	12	69	1	KTILFVPSHNQANTLASAIPGSVPLHR
☒	4170	748.4931	1494.9716	1493.8202	1.1515	1	12	1.2e+02	1	EQGKQQPALLPSAK
☒	6340	661.5789	2642.2865	2643.4274	-1.1409	0	12	78	1	ALGIAQLACLIGNSMGGMTALALLLR + Oxidation (M)
☒	5641	1104.4686	2206.9226	2206.0001	0.9226	0	12	88	1	WPGAVVEAWDSSEEMVTAAR + Oxidation (M)
	5840	605.7676	2419.0413	2419.1625	-0.1212	1	12	79	1	TFRHNLCVCVDGDTTVLLDGK

☒

☒	6116	848.3875	2542.1407	2543.2236	-1.0829	1	12	77	1	RHWMEVTDFIAPMIPCGLAAGR + Oxidation (M)
☒	7588	931.9702	3723.8517	3723.8598	-0.0081	1	12	40	1	TPSSVSLSSLSNVNSSMPHKPASQSNVGPVRISSNR + Oxidation (M)
☒	3570	677.0712	1352.1278	1351.6408	0.4870	0	12	1e+02	1	YGEEVDWIVSR
☒	6088	844.4065	2530.1977	2530.3519	-0.1542	1	12	83	1	VVMGLFIGSLYWQQPLDPRGVR
☒	7308	864.6648	3454.6301	3453.6697	0.9604	1	12	46	1	HGRVDDSPYGGGAGMLLMPQPIFDAIADLPEAK + Oxidation (M)
☒	5437	681.0448	2040.1126	2041.0858	-0.9732	1	12	1.1e+02	1	TAVTRFAPSPTGFLHVGGAR
☒	7830	673.8009	4036.7617	4036.1458	0.6160	1	12	27	1	FSLTIGNVRLQTTFIELVNDGLMTFFFLLIGLEMK + Oxidation (M)
☒	5571	430.4165	2147.0461	2148.0157	-0.9696	1	12	1e+02	1	ERGLSPLGPMTQEFEEGQK + Oxidation (M)
☒	7708	775.9889	3874.9081	3873.9564	0.9517	1	12	36	1	LGTNILDPGESASIVGIQRSQSNITMSMSQLNELVR + Oxidation (M)
☒	5326	662.5137	1984.5193	1984.0126	0.5066	0	12	78	1	LHLPSHTTIGGDPENLQR
☒	7656	764.3870	3816.8986	3816.7434	0.1552	1	12	36	1	LLCGGEALKGDGFDNGAWVAPTVFTDCSDEMTIVR + Oxidation (M)
☒	8303	1115.7715	4459.0569	4458.4782	0.5787	1	12	22	1	DQLKVPMTSFLILSVFMILMPILLMNLLIGLAVGDIESVR + Oxidation (M)
☒	6083	507.0265	2530.0961	2530.1508	-0.0547	0	12	76	1	DNTSFHSVIFPAMEMGQSLPYK + 2 Oxidation (M)
☒	6157	639.7894	2555.1285	2555.3570	-0.2285	1	12	75	1	REEFFLGSVINAIVSQAMFLLR + Oxidation (M)
☒	6785	965.0987	2892.2743	2892.2939	-0.0196	1	12	61	1	NNNGVDMMLDAPSNMNDEDLKELGLK + Oxidation (M)
☒	8400	1129.7778	4515.0821	4514.1644	0.9177	1	12	22	1	SGQMMLAQGLLLHLMPTDWRWSDCHALTDVDFEVLKPR + 3 Oxidation (M)
☒	4177	500.2662	1497.7768	1498.8031	-1.0264	0	12	1.5e+02	1	DIYLVSHLPVSEK
☒	5423	678.3073	2031.9001	2032.0340	-0.1339	1	12	1.1e+02	1	FIAGGMTKGLSVVYYSDPK
☒	5724	762.3914	2284.1524	2282.9566	1.1958	0	12	98	1	VVCEEAGESMMVAAGAGNNDTR + Oxidation (M)
☒	5758	585.2728	2337.0621	2337.2436	-0.1815	0	12	92	1	VFMGDVGSLALGAMLAAISITLR + 2 Oxidation (M)
☒	5863	814.1140	2439.3202	2440.3360	-1.0158	1	12	84	1	VTITEVAKHAGVSVTTVSMVLGNK
☒	6150	512.0328	2555.1276	2555.1745	-0.0468	1	12	75	1	ADPRDTFSSGSAIGLMMGLGNVGDR + 2 Oxidation (M)
☒	6501	542.4323	2707.1251	2708.2754	-1.1503	1	12	62	1	CGNMLGKGASLDEALAQMGMVVEGVR + Oxidation (M)
☒	7013	756.1104	3020.4125	3021.4753	-1.0628	0	12	61	1	LVELVNAVTNELQNYEYANAMHLTEK + Oxidation (M)
☒	5513	519.4741	2073.8673	2074.7676	-0.9003	0	12	95	1	DAAPAMPGGGGMGGMGGMGGMGF + 3 Oxidation (M)
☒	8229	881.2141	4401.0341	4400.1140	0.9201	1	12	24	1	SPAMAGGLFAIERDFFFELGLYDPGLQIWGGENFEISYK + Oxidation (M)
☒	2761	489.3130	976.6114	975.4154	1.1961	0	12	2.2e+02	1	AEMPEMPR + Oxidation (M)
☒	4274	767.7254	1533.4362	1533.6625	-0.2263	1	12	99	1	TREGNMLYMEMK + 2 Oxidation (M)
☒	7488	726.7195	3628.5611	3629.6694	-1.1083	1	12	37	1	VRHGSSNMAEGPAMTMDETLQAIMTGVSLAHEAR + 2 Oxidation (M)
☒	8302	744.1830	4459.0543	4458.4782	0.5762	1	12	23	1	DQLKVPMTSFLILSVFMILMPILLMNLLIGLAVGDIESVR + Oxidation (M)
☒	8548	764.6554	4581.8887	4582.0611	-0.1724	1	12	16	1	GLLENEEVIIEVEQAPKANPMGDMMGQMGMDMSSMLGDMLPK + Oxidation (M)
☒	2762	489.3132	976.6118	976.4800	0.1318	0	12	2.2e+02	1	ILAMGWDR + Oxidation (M)
☒	3264	610.1816	1218.3486	1217.6615	0.6871	0	12	1.3e+02	1	STLAELSTQLR
☒	5549	530.5181	2118.0433	2118.0450	-0.0017	0	12	1.1e+02	1	VTTPGGCTIGGLMVLEEGGVR + Oxidation (M)
☒	7504	910.4728	3637.8621	3637.7433	0.1188	1	12	41	1	ELNCQTLELQVYMNDLFGNDFNTLFKGLSSK
☒	7961	1033.2738	4129.0661	4129.8989	-0.8328	1	12	29	1	MATMTVEAAAAAAAHAVAEAEAIMAEAEAAAREAEAAEAEAR + 2 Oxidation (M)
☒	4147	746.3774	1490.7402	1490.8205	-0.0803	0	12	1.5e+02	1	GLLAPLNHTETAVR
☒	5482	686.6719	2056.9939	2057.0501	-0.0563	1	12	1.1e+02	1	GEVVAQVSAAAEISDAQRTR
☒	6471	537.4681	2682.3041	2682.2649	0.0392	0	12	78	1	QFGHMLEAFEYGAPPHGGIAPGIDR + Oxidation (M)
☒	6627	564.6689	2818.3081	2817.3790	0.9292	1	12	70	1	LFSGATMASSRGALVTVGQLSCYDQAK
☒	7069	623.0841	3110.3841	3109.5179	0.8662	1	12	54	1	NSTQLSFDGDVQIQACWLLNKASAPFIS
☒	7307	690.7450	3448.6886	3447.7634	0.9253	1	12	48	1	NLQGTGHFNLDIVNINDENTIRIPEEIEIK
☒	2913	1007.4782	1006.4709	1005.6011	0.8698	0	12	2e+02	1	IPIFTFLR
☒	4833	860.3926	1718.7706	1717.9111	0.8595	1	12	1.3e+02	1	DGEVIKQPIGNNVHAK
☒	6830	975.7792	2924.3158	2923.4030	0.9128	1	12	64	1	MIEAAEAYPMNLGFLAKGNCGTPAPLR + 2 Oxidation (M)
☒	3711	702.1871	1402.3596	1401.8303	0.5293	1	12	1.1e+02	1	STLLSALRTATLR
☒	5528	525.2377	2096.9217	2096.9138	0.0079	0	12	1.1e+02	1	VDMVEPDMIELVEMEMR + 2 Oxidation (M)
☒	6123	851.3649	2551.0729	2550.2233	0.8496	1	12	72	1	SMYGSLTMKAISIAEEIGYDVEK + Oxidation (M)
☒	6814	730.0412	2916.1357	2915.4964	0.6393	1	12	50	1	YQLHQGVFSLDPRTVAEGEMALIVAR + Oxidation (M)
☒	2376	694.2954	693.2881	694.3650	-1.0769	0	12	9.8	1	SSFGIGK
☒	4525	802.2893	1602.5640	1601.8447	0.7194	0	12	1.1e+02	1	ALAITDECTLAGIVR
☒	4533	803.3445	1604.6744	1604.7906	-0.1162	1	12	1.4e+02	1	ASQSVNEISHKYSR
☒	4885	871.1315	1740.2484	1740.8730	-0.6245	1	12	90	1	WAPVGDVGARDMAVAAR
☒	6579	697.5813	2786.2961	2785.5312	0.7649	1	12	75	1	GNKPMIWKILQEVIEEHPILLNR + Oxidation (M)
☒	6952	999.6295	2995.8667	2995.6189	0.2478	1	12	46	1	ANAAASAAAVAGITLPASAANLIVSSDETKIK
☒	6120	637.2993	2545.1681	2545.1902	-0.0221	1	12	83	1	MIPFEVGMTLDKAYEQEEMLR + Oxidation (M)
☒	6398	534.2688	2666.3076	2667.3465	-1.0389	1	12	80	1	MSSFVQELDKAFEGQLQVLLEEK
☒	6860	588.9046	2939.4866	2938.4603	1.0264	0	12	67	1	DEGTALMSSCIVAAQVVMVPVAYVVGTR + Oxidation (M)
☒	7280	855.6439	3418.5465	3419.5254	-0.9789	1	12	45	1	AMRESDSCKPAIFAMSNPTLNAECTAADAFK + Oxidation (M)
☒	7685	764.5895	3817.9111	3816.7434	1.1677	1	12	38	1	LLCGGEALKGDGFDNGAWVAPTVFTDCSDEMTIVR + Oxidation (M)
☒	7864	811.3752	4051.8396	4051.9702	-0.1306	1	12	30	1	VLFQNWGIEPIDNVMGMIGILPDMTPSTVMSMRGVR + 3 Oxidation (M)
☒	4492	796.9990	1591.9834	1590.8076	1.1759	1	12	1.3e+02	1	KGEMPDFSEALIVR
☒	4926	882.4692	1762.9238	1761.9083	1.0155	1	12	1.4e+02	1	QETLAIRWMLESAAK + Oxidation (M)
☒	7029	760.5764	3038.2765	3037.6600	0.6164	1	12	53	1	LEVGDNHLVFVAPGAFAGLAKLSTLTLER
☒	2760	489.3124	976.6102	975.5138	1.0965	1	12	2.2e+02	1	TRLSEWGK
☒	3147	573.0314	1144.0482	1143.5520	0.4963	0	12	1.3e+02	1	VEGAAVAENR
☒	3517	663.8712	1325.7278	1326.7296	-1.0017	0	12	1.6e+02	1	EHLLLSAEFLR
☒	3739	707.6357	1413.2568	1412.6837	0.5732	0	12	1.1e+02	1	DLDWLWPNAQR
☒	5505	689.8318	2066.4736	2067.0531	-0.5795	1	12	74	1	NATGVVLGLPKNMNNSEGPR
☒	5861	813.3369	2436.9889	2437.2610	-0.2721	1	12	74	1	FIPKLNGLRPIVNMSYGMDTR + Oxidation (M)
☒	6102	509.0372	2540.1496	2540.2331	-0.0835	1	12	82	1	EFLPYLQELEKMPESLMHFK + 2 Oxidation (M)
☒	6644	944.4189	2830.2349	2829.3829	0.8519	1	12	66	1	ASIYNAMPLEGVIALTDFMADFERR
☒	8355	897.9984	4484.9556	4485.9404	-0.9848	1	12	20	1	VASTMSDGANTMIEHDDTLPSQLGTMVINADDEEEEGTMKR + 3 Oxidation (M)
☒	2453	371.0997	740.1848	740.4181	-0.2332	0	12	24	1	GPTITPR
☒	4478	795.3611	1588.7076	1588.8031	-0.0955	0	12	1.5e+02	1	NMIALTGIGYDIHR + Oxidation (M)
☒	5425	1017.1504	2032.2862	2033.0251	-0.7389	1	12	85	1	ENNQMPPISYKQGLALSK + Oxidation (M)
☒	6611	563.2860	2811.3936	2810.4603	0.9334	1	12	75	1	EIISALDHVIIDAAISDLNGRDFWK
☒	7066	622.2733	3106.3301	3106.3423	-0.0122	0	12	51	1	GSIANPDASYHMEMTADYEEYLDDLIK + Oxidation (M)
☒	7324	696.1202	3475.5646	3474.6784	0.8862	0	12	45	1	SMLTASTTLNLQLQQQIDTISSNLSNSNTTGYK + Oxidation (M)
☒	6964	600.3094	2996.5106	2997.3307	-0.8201	0	12	66	1	VSFQALNLDVTGCENMNYFVCQSTGK + Oxidation (M)
☒	7356	702.9210	3509.5686	3508.8206	0.7480	0	12	43	1	GGVVGATATMGVIVGSDIPMFLGAMIMGPLGGYLIK + Oxidation (M)
☒	3023	532.8460	1063.6774	1063.4968	0.1807	0	12	1.8e+02	1	MATPSLDSAR + Oxidation (M)
☒	3411	644.4200	1286.8254	1286.6830	0.1424	1	12	1.7e+02	1	VTIGIEEENKR
☒	7540	733.9382	3664.6546	3664.7251	-0.0705	1	12	40	1	FCLYPMTKEQFQWIQTALNEALALDMSGESK + Oxidation (M)
☒	8150	861.2023	4300.9751	4300.8422	0.1329	0	12	26	1	FGSAPGAGMPGGDIFGGMGGMGGVGFDPMDIFEQLFGGAAGMGGR + 4 Oxidation (M)
☒	3802	355.6884	1418.7245	1419.9064	-1.1820	0	12	1.7e+02	1	LSAVKPLIPILEK
☒	4491	531.5795	1591.7167	1592.7729	-1.0563	1	12	1.4e+02	1	RTGFPNVTMAPGSSR + Oxidation (M)
☒	2998	526.7836	1051.5526	1050.6437	0.9089	0	12	1.7e+02	1	QIVGPIIPSK
☒	3609	681.8432	1361.6718	1361.6431	0.0287	0	12	1.7e+02	1	GMQMLAEDIAAGR
☒	6442	534.2699	2666.3131	2667.3465	-1.0334	1	12	81	1	MSSFVQELDKAFEGQLQVLLEEK
☒	4345	387.9482	1547.7637	1547.8170	-0.0533	0	12	1.5e+02	1	ASLLAFWDLIPMR + Oxidation (M)
☒	4460	526.9268	1577.7586	1578.8253	-1.0667	1	12	1.5e+02	1	GQGAGSKLLTYVEEK
☒	8380	899.2191	4491.0591	4491.1897	-0.1306	1	12	23	1	RCNLASQSSPGDSSSNIDFLQVKPSTALEVPIASAESDLCLK
☒	4065	369.6935	1474.7449	1473.5727	1.1722	0	12	1.6e+02	1	SWFEPMMEDMR + Oxidation (M)
☒	5407	506.0109	2020.0145	2021.0694	-1.0549	1	12	1.2e+02	1	VNFGSRKPAPQTTYQVTK
☒	6046	505.2901	2521.4141	2521.2224	0.1917	0	12	79	1	AAAAFIAYTTYTDGSAVGVDDTLTK
☒	6602	703.6035	2810.3849	2811.2024	-0.8175	0	12	76	1	MGHHGQMHAQHGVTMPADSPTGQPDR + 2 Oxidation (M)
☒	7131	645.4845	3222.3861	3222.6934	-0.3072	1	12	47	1	IDGSIAGAMATMAGVLFALVYLFSPKHGVVR + 2 Oxidation (M)
☒	8217	880.6013	4397.9701	4398.0527	-0.0826	0	12	22	1	DTFSSGSATGFLIGVGNVGEFLTPMDDPSTATFLSTDGGVTWK + Oxidation (M)
☒	2654	900.4222	899.4149	899.6055	-0.1906	0	12	2.2e+02	1	IILITSIK

☒	3521	664.3517	1326.6888	1327.7361	-1.0472	0	12	1.7e+02	1	THHIDVAPLVAR
☒	5428	509.7578	2035.0021	2034.0853	0.9168	1	12	1.2e+02	1	MKIAVAMSGGVDSSVAAALLK + Oxidation (M)
☒	5599	1088.5117	2175.0088	2175.1575	-0.1487	0	12	1e+02	1	NGEEVAFIAEVQQAGIFLIK
☒	6312	874.1133	2619.3181	2619.2317	0.0863	1	12	85	1	MFMTSSCKPGNKVSMIWNSLTGK + Oxidation (M)
☒	7340	696.7233	3478.5801	3478.6782	-0.0981	1	12	45	1	LTLPFVDMSKAAAMDGTSTTLNYATALMGGNLR + 3 Oxidation (M)
☒	7589	621.6494	3723.8527	3724.8705	-1.0178	1	12	42	1	QAEALGYQAVVLSAAMQGDVKSMAQFYGLLAHVAR + 2 Oxidation (M)
☒	8243	736.4940	4412.9203	4411.9840	0.9363	1	12	21	1	DGALGIDDRPVETEHEDAKSYSQQETFDEDIASFAEIER
☒	8721	1391.2159	5560.8345	5561.6164	-0.7819	1	12	11	1	MTDNDALSLPPDVFAAEDLAAEIDEETSCVVVQTPDAFGNLRDLSPLAAACK
☒	2509	391.1963	780.3780	780.3072	0.0708	0	12	69	1	ASADDMR + Oxidation (M)
☒	5473	1024.0159	2046.0172	2045.0952	0.9220	1	12	1.1e+02	1	MQNAEAAPALLAAHGLVGRR
☒	5725	762.3932	2284.1578	2283.9987	0.1590	1	12	1e+02	1	NEDDNDIMETLCKNDFALK
☒	3709	701.8909	1401.7672	1400.6064	1.1608	0	12	1.7e+02	1	IQEMFDNMVSR + 2 Oxidation (M)
☒	5082	620.3307	1857.9703	1858.8672	-0.8970	0	12	1.3e+02	1	GFSMPPPGTSDLHFPTR + Oxidation (M)
☒	5379	674.0115	2019.0127	2019.9691	-0.9564	1	12	1.2e+02	1	SFTFRELHVYTDGFSSK
☒	6158	852.7168	2555.1286	2556.2240	-1.0954	0	12	79	1	IGELWMSLDLLEHALDQMPEGK + 2 Oxidation (M)
☒	6429	534.2697	2666.3121	2667.3465	-1.0344	1	12	82	1	MSSFVQELDKAFEGQLQVLLEEK
☒	7889	812.8225	4059.0761	4060.0232	-0.9470	1	12	32	1	FVDVKANGPSSVGLDFSLHGFEHLYGIPQHAESHQLK
☒	3348	1258.0923	1257.0850	1257.7081	-0.6231	0	12	1.3e+02	1	VVVFKPAIDDR
☒	3456	652.8843	1303.7540	1303.6806	0.0735	1	12	1.8e+02	1	QKSIENVDMLK
☒	4530	802.8237	1603.6328	1603.7772	-0.1443	1	12	1.3e+02	1	ASLMMYEKLSMIR + 2 Oxidation (M)
☒	5199	949.2489	1896.4832	1896.0145	0.4687	0	12	85	1	IWYVDIISINDLAPHK
☒	5931	829.0510	2484.1312	2484.1487	-0.0176	0	12	88	1	EALEGVNICMVVHQPSYTMYK + Oxidation (M)
☒	7223	834.4491	3333.7673	3333.7444	0.0229	1	12	52	1	LLAGRYAVMNLIPYNTVDGLAFQRPAWER
☒	2452	371.0996	740.1846	741.3770	-1.1923	0	12	24	1	AGPGSPTR
☒	3980	722.3290	1442.6434	1441.7063	0.9372	1	12	1.6e+02	1	RHGVADGDVDVFR
☒	6230	522.8647	2609.2871	2609.3352	-0.0481	1	12	84	1	WFLKGYQNANYLSLFLMVATSK + Oxidation (M)
☒	4350	775.6725	1549.3304	1549.6678	-0.3374	1	12	1e+02	1	RSGECDEEEIQAK
☒	7557	738.5289	3687.6081	3687.9368	-0.3287	1	12	36	1	ERLVITPLTDICYITLSQALGMFLGGAPAGPAGTGK
☒	7921	1028.2224	4108.8605	4109.7399	-0.8794	0	12	29	1	YDQFGHAGVGSSAASGGGQYGAGASDFSDIFSAFNDMFGGGK + Oxidation (M)
☒	4352	775.8147	1549.6148	1549.7810	-0.1662	0	12	1.4e+02	1	GEDVICVYVAIGQK
☒	4819	852.3487	1702.6828	1702.9465	-0.2637	1	12	1.3e+02	1	AKSTVSITVVGASGDLAK
☒	5007	604.6720	1810.9942	1809.9659	1.0283	0	12	1.3e+02	1	GEAPQLVQMVNALGAVVV + Oxidation (M)
☒	5846	608.5596	2430.2093	2431.2062	-0.9969	0	12	95	1	LNCVTSLPPMFLGCTAVTIGHK + Oxidation (M)
☒	8045	847.2023	4230.9751	4231.0262	-0.0511	1	12	28	1	FGDGAHLAFAGHVDVVPPGEGWQSEPFTPLMKDGFIYAR + Oxidation (M)
☒	4143	746.3739	1490.7332	1490.7770	-0.0437	0	12	1.6e+02	1	VSGDVWFGEGVVLK
☒	6048	631.3613	2521.4161	2521.1927	0.2234	1	12	81	1	KMQVPTAEEMSSLTPESSPELAK + 2 Oxidation (M)
☒	2687	924.4839	923.4766	924.5756	-1.0990	1	12	1.8e+02	1	RILPADIK
☒	3240	399.2121	1194.6145	1195.6635	-1.0490	1	12	1.7e+02	1	AMITKASLAFK + Oxidation (M)
☒	6113	636.2960	2541.1549	2540.3282	0.8267	1	12	85	1	NPTHVTESARLGAHVATMPPAVIR + Oxidation (M)
☒	7968	827.3562	4131.7446	4132.1337	-0.3891	1	12	25	1	QLGLPGKGPVAIITDLCIMEPEAGSNEFIVTSLHPGVTR + Oxidation (M)
☒	4734	561.6512	1681.9318	1680.8505	1.0813	1	12	1.4e+02	1	NDVLMITAGSDFRVK + Oxidation (M)
☒	5047	459.5063	1833.9961	1833.9692	0.0269	1	12	1.3e+02	1	QMLGLEMLTERGILAK + 2 Oxidation (M)
☒	6133	511.8326	2554.1266	2554.3586	-0.2320	1	12	80	1	FNILMGISCVTFMIIGSNILRR
☒	7976	1037.0291	4144.0873	4144.1808	-0.0936	1	12	32	1	VSYFLMGIGAISLLVAGIGIGNVMLMSVVERTTEIGVMR + 3 Oxidation (M)
☒	7992	833.9681	4164.8041	4165.1720	-0.3678	0	12	25	1	MSTTTEILAHHWAFGLFLIIAVGLCVFMLTGGFLLGGR + Oxidation (M)
☒	8407	904.0247	4515.0871	4515.2058	-0.1187	1	12	23	1	LNQIGAGDQGLMFGYATNETEELMPLPIMLSHHLMQRIAK + 3 Oxidation (M)
☒	3414	645.2876	1288.5606	1289.6438	-1.0831	1	12	1.9e+02	1	EYMPELPKQR
☒	3997	725.2118	1448.4090	1447.7462	0.6629	1	12	1.1e+02	1	VVCITRGMPMLR + Oxidation (M)
☒	4281	769.1269	1536.2392	1535.6886	0.5507	0	12	1e+02	1	SSNLEDPQMGSSIR + Oxidation (M)
☒	5587	721.2419	2160.7039	2161.1604	-0.4565	1	12	74	1	VGGGLLVQGPDARNVQPSELR
☒	2465	752.8336	751.8263	752.5160	-0.6897	0	12	24	1	LIVALPK
☒	3707	701.3436	1400.6726	1400.6857	-0.0131	0	12	1.8e+02	1	MEIDSKPEPLDK
☒	4024	729.4434	1456.8722	1457.8453	-0.9731	1	12	1.6e+02	1	KSLVNALTSLGVEK
☒	6473	537.4689	2682.3081	2683.2781	-0.9700	1	12	83	1	IQYYQLDEVFVFPYDMGSRWK
☒	7582	621.6485	3723.8473	3723.9472	-0.0999	1	12	43	1	RNPTVITLYELAQALGVSHVELVTPTDETPFGTR
☒	7598	932.2216	3724.8573	3725.7270	-0.8697	1	12	42	1	AGMDRVQADYTGMLGTVMNALVMADALQHAGVDTR + 3 Oxidation (M)
☒	8419	1129.7804	4515.0925	4514.1644	0.9281	1	12	24	1	SGQMMLAQGLLLHLMPTDWRWSDCHALTDVDFEVLKPR + 3 Oxidation (M)
☒	7465	899.9211	3595.6553	3595.7044	-0.0491	1	12	47	1	DQYATHMRDVVMAMLGLTHTMNTLVGNEFIR + 2 Oxidation (M)
☒	8499	907.2217	4531.0721	4532.1516	-1.0795	1	12	23	1	EFGVMVSVFDLFQASTLGGMAAKIDGSTGADNQPIIWEEETR + Oxidation (M)
☒	8390	1128.2458	4508.9541	4509.9718	-1.0177	0	12	20	1	GIMAMNSYNHMGQGIHHATSSLLTSMMAETFEELAVDYMK + 4 Oxidation (M)
☒	3652	691.3448	1380.6750	1380.6707	0.0043	0	12	1.7e+02	1	VFAVMGISEADAR + Oxidation (M)
☒	4871	577.9655	1730.8747	1730.9488	-0.0741	1	12	1.5e+02	1	GEVADLTVLLAEKVMK + Oxidation (M)
☒	5299	981.9733	1961.9320	1960.9023	1.0298	1	12	1.3e+02	1	ICDLMADFNEFSTRVK + Oxidation (M)
☒	6055	842.3822	2524.1248	2523.2461	0.8786	1	12	83	1	ILMSRPGNLLPSNKTDDCVYSK + Oxidation (M)
☒	7731	986.6634	3942.6245	3943.0482	-0.4237	1	12	26	1	GIIGEVMASGFDGIFLVATNPVDILTYATWKFSGLPK + Oxidation (M)
☒	5574	430.6138	2148.0326	2148.0231	0.0095	1	12	1.1e+02	1	MSGTNTEKEQMLLVNFFK + 2 Oxidation (M)
☒	6047	631.3613	2521.4161	2520.4866	0.9295	1	12	83	1	LAIVPILRAGIGMVDGFLSLVPAAK
☒	6193	1292.5698	2583.1250	2582.3018	0.8232	1	12	80	1	RYVMMIDQNIILNDNMAILVR + 3 Oxidation (M)
☒	6426	667.5853	2666.3121	2665.2150	1.0971	0	12	85	1	NHGNNEADAALQGLAQQGVDMEDLR
☒	7070	779.6392	3114.5277	3115.7059	-1.1782	1	12	64	1	EMKLTALAILVTPALVLMGTALALMTDAGR + 2 Oxidation (M)
☒	7174	821.1096	3280.4093	3280.7241	-0.3148	1	12	51	1	MTMNRLLLLILPAIIMLAAMFATSGMEQR + 2 Oxidation (M)
☒	8148	717.6586	4299.9079	4299.9860	-0.0780	1	12	26	1	QLPQGPGEVPMASSVTTPTPTEEGECQSLVQSGRQEETSR + Oxidation (M)
☒	3095	564.7739	1127.5332	1127.5723	-0.0391	1	12	2e+02	1	LAGKADPDWR
☒	6662	713.6459	2850.5545	2851.4552	-0.9007	1	12	73	1	TIRIWDVSVGQCLLTLNGHDNWVR
☒	6828	975.4816	2923.4230	2924.4444	-1.0214	1	12	73	1	AEGSGPTYEPAPPTPTLATTASATAKFYK
☒	7256	677.5304	3382.6156	3383.7619	-1.1463	0	12	53	1	AGASTVAELATIGRPAIMVPLPGAIDDHQTANAR
☒	3360	632.4285	1262.8424	1262.6255	0.2170	0	12	1.6e+02	1	AAAIYGAEVNER
☒	6605	703.6049	2810.3905	2810.3035	0.0870	0	12	80	1	EAAGATWSDEVSNAWEVAYDGLSAAIK
☒	7294	859.3573	3433.4001	3432.8958	0.5043	0	12	38	1	IGVLFGPVMVTWFLVLGALGVYGIMQSPEVLK
☒	4870	866.4354	1730.8562	1730.8331	0.0231	1	12	1.5e+02	1	DADAKLEPMVGAIMDR
☒	8342	746.3318	4471.9471	4471.3939	0.5533	1	12	21	1	MLPYEMVIAVLVYLSPAQILNLNLPFAYQKSVLFASNSAK + Oxidation (M)
☒	5087	620.6672	1858.9798	1857.9982	0.9815	1	12	1.3e+02	1	LTDTTRAQLVQAMPGLK + Oxidation (M)
☒	5585	720.6169	2158.8289	2159.0602	-0.2314	1	12	88	1	EEDVPTHAITMGIGSIMKAK + 2 Oxidation (M)
☒	6167	512.4544	2557.2356	2556.2716	0.9640	0	12	91	1	LVNLVAADGHPAEIMDMSFALQAK + Oxidation (M)
☒	7653	764.3867	3816.8971	3816.7434	0.1537	1	12	40	1	LLCGGEALKGDGFDNGAWVAPTVFTDCSDEMTIVR + Oxidation (M)
☒	8147	860.9882	4299.9046	4299.9860	-0.0814	1	12	26	1	QLPQGPGEVPMASSVTTPTPTEEGECQSLVQSGRQEETSR + Oxidation (M)
☒	4515	799.3187	1596.6228	1595.8923	0.7306	1	12	1.3e+02	1	LYVAVTGDKVQLYK
☒	6238	522.8649	2609.2881	2608.3530	0.9351	1	12	88	1	ALMEEDRYTGIVTISVNLSKPTR + Oxidation (M)
☒	6388	667.3334	2665.3045	2664.3008	1.0037	1	12	85	1	VNVQSPHRAMAALGLMLTCMYTGK + Oxidation (M)
☒	6771	577.4892	2882.4096	2882.3401	0.0695	1	12	78	1	IAIVGMAGRGPGCDNVDEFWDLIMSK + 2 Oxidation (M)
☒	8528	761.8259	4564.9117	4565.1863	-0.2746	0	12	18	1	GSQLDLVVAGTEAAVLMVESEADQLSEEVMLGAVVFGHTEMQK + 3 Oxidation (M)
☒	2975	1036.9408	1035.9335	1035.4655	0.4680	1	12	1.4e+02	1	TACNKADEK
☒	3548	672.3380	1342.6614	1343.7482	-1.0868	1	12	1.9e+02	1	IVEDKQIIQMK
☒	4975	601.6669	1801.9789	1800.8853	1.0935	1	12	1.4e+02	1	EAREESVDAILDLADR
☒	5422	678.0304	2031.0694	2031.1914	-0.1221	1	12	1.2e+02	1	VGVLTAVINHMLPDIAKIK
☒	5630	734.0384	2199.0934	2199.0253	0.0681	0	12	1.1e+02	1	DEVGMTATITSSEYIPNLDK + Oxidation (M)
☒	2562	406.2473	810.4800	810.3984	0.0816	0	12	1e+02	1	GPGAAPDAR
☒	5553	1062.5160	2123.0174	2122.0980	0.9194	0	12	1.2e+02	1	GVIDLAEVEAVAPGTPTMGAPK
☒	5670	748.4980	2242.4722	2242.0138	0.4584	0	12	74	1	EQAAAEGEETPAQEWEVVNR
☒	3670	693.8799	1385.7452	1384.6636	1.0816	0	12	1.8e+02	1	LQNAFWEHGAGR
☒	4976	451.9546	1803.7893	1804.9361	-1.1468	1	12	1.4e+02	1	TRMLLMPSMKPEALR + 2 Oxidation (M)

☒	5364	672.2570	2013.7492	2014.8434	-1.0943	0	12	93	1	VMAEAPTQEACDMYVER + Oxidation (M)
☒	6517	544.8518	2719.2226	2719.4043	-0.1817	0	12	78	1	LAVEHKPTMIIAGFSAYSGIVDWAK + Oxidation (M)
☒	6576	927.7752	2780.3038	2779.3341	0.9697	0	12	81	1	FSLFSTSGADLFTNFQLEAEAELSR
☒	7596	621.8152	3724.8475	3724.0426	0.8050	1	12	44	1	VLTPQIFADFLMLIHNNEISSASGKLLLFHVLK + Oxidation (M)
☒	8305	744.1854	4459.0687	4459.2699	-0.2012	1	12	25	1	LDGSIPPALADLNSLGYLNISYNNLSGEIPFKGHLVTFDER
☒	8381	899.4210	4492.0686	4491.1475	0.9211	1	12	24	1	YVEENNSVVGAPQMHPEDLPVFDTATAMPGQRVLSPESHVK + Oxidation (M)
☒	3731	705.3722	1408.7298	1409.7878	-1.0580	1	12	1.7e+02	1	EDVPPGTSILRVK
☒	4545	807.3469	1612.6792	1612.8897	-0.2104	1	12	1.5e+02	1	IEGAGTSRIVVEGVAR
☒	3151	574.0579	1146.1012	1146.5703	-0.4691	0	12	1.4e+02	1	EPSVVVMQSR + Oxidation (M)
☒	5035	608.9760	1823.9062	1823.8571	0.0490	0	12	1.4e+02	1	GTGSGLIGASGATMPTDTSK + Oxidation (M)
☒	5871	814.7272	2441.1598	2440.3339	0.8259	1	12	97	1	VILLNLLDNAWREHLHTLDR
☒	6402	667.5847	2666.3097	2665.2150	1.0947	0	12	87	1	NHGNNEADAALQGLAQQGVDMEDLR
☒	6526	911.3975	2731.1707	2730.2629	0.9078	1	12	73	1	NVNDVDTALSFYHMAGASIDKATMK + 2 Oxidation (M)
☒	4516	799.4024	1596.7902	1596.7163	0.0739	1	12	1.6e+02	1	FEAMKAMESDPIASA
☒	5882	616.7864	2463.1165	2462.2230	0.8935	1	12	93	1	FITFEGIDGAGKSTHIEWVAER
☒	5922	825.7548	2474.2426	2474.2223	0.0202	1	12	96	1	QLREELQQPLAVDDYVNAMAR + Oxidation (M)
☒	6332	528.2293	2636.1101	2636.2588	-0.1487	0	12	75	1	VMLVGAMSGPLGGDHFEVGAHAANGAR + Oxidation (M)
☒	4356	517.8964	1550.6674	1549.7875	0.8799	0	12	1.6e+02	1	LVKPESSYEELEK
☒	8745	1148.7416	5738.6716	5738.0772	0.5944	1	12	11	1	LGIDGLSIGPILLTGFITTLATLAAWPVTRNSQLFHFLMLAMYSAQIGLFSSR + Oxidation (M)
☒	5260	484.4629	1933.8225	1933.8874	-0.0649	0	12	1.2e+02	1	SANDVGTGLVGAPACGDVMK + Oxidation (M)
☒	5496	688.4362	2062.2868	2061.1721	1.1147	0	12	89	1	AAEAGIVIPEIIPAGLELSAK
☒	5748	578.7662	2311.0357	2312.1244	-1.0887	1	12	1e+02	1	EGSDKAADAPAAAPQQVASSATAAK
☒	6404	667.5848	2666.3101	2667.3292	-1.0191	0	12	88	1	HWETTILPSGYGLEARPVAEANEK
☒	6782	964.1067	2889.2983	2889.4807	-0.1824	1	12	71	1	MHHPFRVAIPAASSDQLLSALDQELK + Oxidation (M)
☒	6961	600.3091	2996.5091	2997.5746	-1.0655	1	12	71	1	HIVHPTTLDSVIQAAYTVLPYAGTRMK + Oxidation (M)
☒	7323	696.1199	3475.5631	3475.6639	-0.1008	1	12	49	1	AMVSGQEATDFAVKINAEVPLSEMFGYATDLR + Oxidation (M)
☒	7689	960.9759	3839.8745	3838.8923	0.9822	1	12	41	1	SIFPLQYFSVGASLIPFLEHNDANRALMGSNMQR + Oxidation (M)
☒	3497	658.1335	1314.2524	1313.7554	0.4970	1	12	1.3e+02	1	QLLKLEESLNK
☒	3507	660.7758	1319.5370	1319.6689	-0.1319	1	12	1.8e+02	1	LMKNAIGEGMTR
☒	5592	723.4852	2167.4338	2168.1987	-0.7649	1	12	79	1	ATTAGILSTLSHCIDILVKR
☒	6406	667.5848	2666.3101	2665.2150	1.0951	0	12	88	1	NHGNNEADAALQGLAQQGVDMEDLR
☒	6807	971.4230	2911.2472	2910.3892	0.8580	1	12	64	1	VDGILMDLGVSSPQLDEPERGFSFMR + Oxidation (M)
☒	7702	968.7054	3870.7925	3871.0382	-0.2457	1	12	39	1	LYNIIYENNNINTIPNNILMFKYLVGWSIQLK + Oxidation (M)
☒	8376	642.2859	4488.9504	4488.1081	0.8422	0	12	22	1	EFAHHVEVPDNSNFVDIVGTGGDGAHTFNISTASMFVTAAAGAK
☒	4557	810.8258	1619.6370	1620.7388	-1.1018	0	12	1.4e+02	1	YDIMNDVMSAGLHR
☒	4564	811.3284	1620.6422	1621.7711	-1.1289	0	12	1.4e+02	1	MEPWVPQWPPPSR + Oxidation (M)
☒	5617	733.6486	2197.9240	2197.0620	0.8620	1	12	1e+02	1	MNGSNGEERVVHEVAMVVPK + Oxidation (M)
☒	5836	805.7272	2414.1598	2413.1736	0.9862	1	12	1e+02	1	WNVSPYYSTAACVEALLDARK
☒	6282	871.7234	2612.1484	2613.2455	-1.0971	1	12	82	1	KVDDYPYGGGAGMVMAAQPIVDSIK + 2 Oxidation (M)
☒	6903	747.0953	2984.3521	2983.2496	1.1024	0	12	68	1	VKPPYTSCADCMYPTAGGTPEAYMER + 2 Oxidation (M)
☒	3066	553.8279	1105.6412	1106.4775	-0.8362	0	12	2.2e+02	1	GVQGNSMDQR + Oxidation (M)
☒	3395	642.4279	1282.8412	1281.6816	1.1596	1	12	1.6e+02	1	KDLLSSYDTLK
☒	3750	709.4660	1416.9174	1417.7388	-0.8213	1	12	1.6e+02	1	MDQPKSFAIPLR + Oxidation (M)
☒	7106	634.2809	3166.3681	3167.4805	-1.1123	1	12	55	1	NYPVLFDEHLAPFMNDLQVSRTMDSGR + Oxidation (M)
☒	4719	838.5124	1675.0102	1674.8974	0.1128	1	12	1.5e+02	1	ILDKGLAGTATLAEMR + Oxidation (M)
☒	5720	1143.0769	2284.1392	2285.1863	-1.0470	1	12	1.1e+02	1	GDGTTGENITENVRTISAIPLK
☒	6149	512.0327	2555.1271	2556.2682	-1.1411	1	12	85	1	YPRGNGIGVPLMEEGWEPLEIGK + Oxidation (M)
☒	7391	884.6586	3534.6053	3534.5798	0.0255	1	12	49	1	AYEESLLTDVDGGYEDRVYDDDLADVVIDDR
☒	8077	850.2055	4245.9911	4246.0336	-0.0425	0	12	31	1	DNPTAQALSPALATGVSDGSNFSLSDSLQPQLNSMPQGSVTK + Oxidation (M)
☒	4145	373.6921	1490.7393	1490.7836	-0.0444	1	12	1.7e+02	1	MEQIQMVKVLEK + Oxidation (M)
☒	5834	1208.0704	2414.1262	2414.1433	-0.0170	0	12	1e+02	1	QFGMPTVSTVPIGIGATEMFCR + Oxidation (M)
☒	4502	798.3195	1594.6244	1593.7708	0.8536	1	12	1.4e+02	1	MTNWTEIDEIAKK + Oxidation (M)
☒	4549	808.3605	1614.7064	1615.7923	-1.0858	1	12	1.6e+02	1	SRQQAMHIASSLCK
☒	4759	423.7074	1690.8005	1689.7992	1.0013	0	12	1.5e+02	1	AEGLGMVNEDGTVINR + Oxidation (M)
☒	5809	799.1650	2394.4732	2394.1972	0.2760	1	12	78	1	LFKFSILYMMLLCAAMVGDR + Oxidation (M)
☒	7912	683.6116	4095.6259	4095.0923	0.5336	1	12	25	1	IVQMCSDYLANLSDILIFVLWAGGLELTTMRYFVK + Oxidation (M)
☒	7040	763.1132	3048.4237	3047.3409	1.0828	0	12	69	1	YLGEQIGNGMTLEEIESEMDMVAEGVR + 3 Oxidation (M)
☒	7325	870.1470	3476.5589	3476.5766	-0.0177	0	12	49	1	GLGSHQLVMSEAYDPEVNSWSPVSDGMVAGWR + Oxidation (M)
☒	2447	368.7185	735.4224	735.3511	0.0713	0	12	45	1	QSTSASR
☒	3259	406.5188	1216.5346	1217.6842	-1.1496	0	12	2.1e+02	1	ALLTGLGMPFAK
☒	3276	617.6346	1233.2546	1233.6387	-0.3841	1	12	1.4e+02	1	SDSLIARDVMK
☒	4005	726.0957	1450.1768	1450.7490	-0.5721	0	12	1.2e+02	1	CFLLTSNAGVIEK
☒	6609	563.2853	2811.3901	2811.4476	-0.0575	1	12	83	1	YVELLEANRTAQQNFIDSSMILLK + Oxidation (M)
☒	6720	574.4898	2867.4126	2866.4515	0.9611	0	12	82	1	ITASRPLAFYAYGVGVGGEQLGGSHFGR
☒	7133	806.6043	3222.3881	3221.3327	1.0554	1	12	52	1	NSSSINISFDNGRSSDEDGMDEEDEAFVR
☒	2480	763.3280	762.3207	762.4640	-0.1432	0	12	97	1	SFAVVIK
☒	3572	677.8524	1353.6902	1354.7681	-1.0778	1	12	1.8e+02	1	RAQQALASGAALAK
☒	3637	687.8795	1373.7444	1373.7667	-0.0222	1	12	1.9e+02	1	VGISKSTDLLWR
☒	3840	477.8539	1430.5399	1431.6487	-1.1088	0	12	1.6e+02	1	HLGQTGMVDMTDK
☒	6616	704.8251	2815.2713	2814.5652	0.7061	0	12	78	1	MISLGHLLALGAVLFCISLAGIFLNR + Oxidation (M)
☒	2537	399.2449	796.4752	795.3796	1.0956	1	12	91	1	MASEKSK + Oxidation (M)
☒	2759	489.3122	976.6098	977.4535	-0.8437	1	12	2.5e+02	1	MPGRMGGTR + Oxidation (M)
☒	4795	565.2711	1692.7915	1691.7859	1.0056	0	12	1.5e+02	1	CDSVISLSDLTMAHK + Oxidation (M)
☒	6554	553.8359	2764.1431	2764.2763	-0.1332	1	12	68	1	GQETFPSRSMDDILSFLHGPQSGDK + Oxidation (M)
☒	8500	907.2242	4531.0846	4532.2388	-1.1542	1	12	25	1	DNTHVLVVGDGVNDAPAMAAAHTSIAMGRAGADLTVQTADVVTIR + 2 Oxidation (M)
☒	2779	494.2992	986.5838	987.4807	-0.8969	0	12	2.5e+02	1	MAQEAPAVR + Oxidation (M)
☒	2792	500.2587	998.5028	999.5349	-1.0320	0	12	2.1e+02	1	LGEGELQVR
☒	7004	603.5182	3012.5546	3013.5769	-1.0223	1	12	71	1	ASTLSKYGVHGFPTIILMNSTMLVVYR + Oxidation (M)
☒	8617	781.0019	4679.9677	4680.3752	-0.4075	1	12	17	1	RPSVCNAAETLLIDAAIADHTMPRLVAALQGAGVTVHGGLSGDPDK + Oxidation (M)
☒	3691	698.8374	1395.6602	1394.6976	0.9626	0	12	1.8e+02	1	MDHLPQLVAEAR + Oxidation (M)
☒	4375	781.4390	1560.8634	1560.7453	0.1181	0	12	1.7e+02	1	MDPTTGESAAALLER
☒	5921	1238.1277	2474.2408	2475.3164	-1.0756	1	12	1e+02	1	RAAVLLLCGGATVSALVMMVFHK + 2 Oxidation (M)
☒	6732	574.6923	2868.4251	2868.3820	0.0431	1	12	82	1	LVGQAIETFNMIEDGDRLMVCLSGGK + Oxidation (M)
☒	6967	999.8463	2996.5171	2995.4498	1.0673	0	12	74	1	MDIAHDLQVIAAQEHTLVFPQFDADR + Oxidation (M)
☒	8413	1129.7798	4515.0901	4514.1644	0.9257	1	12	26	1	SGQMMLAQGLLLHLMPTDWRWSDCHALTDVDFEVLKPR + 3 Oxidation (M)
☒	4167	748.2406	1494.4666	1493.8202	0.6465	1	12	1.2e+02	1	EQGKQQPALLPSAK
☒	5533	701.5117	2101.5133	2102.1194	-0.6061	0	12	84	1	FGDAMAIGLSQALAIIPGVSR + Oxidation (M)
☒	6212	649.6050	2594.3909	2595.3043	-0.9134	1	12	89	1	VEPVFIAEKEYLMLEVSASAPHH
☒	7562	740.7403	3698.6651	3698.9937	-0.3286	1	12	45	1	GITTKWSVEFTFAPIHGFGNTQALLELVTRPLR
☒	4765	846.7094	1691.4042	1690.7944	0.6098	1	12	1.1e+02	1	SDLLESQQQNKCNK
☒	6664	714.0946	2852.3493	2851.3342	1.0151	0	12	81	1	LAEMIFLEQNAVEEAMGMYQELHR
☒	6715	717.8602	2867.4117	2868.3820	-0.9703	1	12	83	1	LVGQAIETFNMIEDGDRLMVCLSGGK + Oxidation (M)
☒	7343	696.7236	3478.5816	3479.6848	-1.1032	1	12	50	1	NAKLFMNLFAMMVSTGIPELQSMEDLNYLK + 2 Oxidation (M)
☒	7444	719.3375	3591.6511	3591.8429	-0.1917	1	12	50	1	IEAIVRAEMDAAGAIELLMPVVQPAELWQESGR
☒	7697	645.9746	3869.8039	3869.8784	-0.0745	0	12	42	1	DHVEIGDNAVIMMGAVINIGAVVGEGTMIDMNAVLGGR + 2 Oxidation (M)
☒	8540	914.8114	4569.0206	4568.3207	0.6999	0	12	22	1	LLLPTDVVVTAEISADAESSVASIADMPNNLIGVDIGPETAAAYR
☒	4146	746.3771	1490.7396	1489.8154	0.9243	1	12	1.8e+02	1	FAPQIRVNLNYR
☒	4341	774.4096	1546.8046	1545.8766	0.9280	1	12	1.8e+02	1	ENNLFSDLLKLIK
☒	4444	786.9590	1571.9034	1572.7566	-0.8531	0	12	1.7e+02	1	LSTMHADSAEAGVIR + Oxidation (M)
☒	4527	802.7535	1603.4924	1603.8028	-0.3103	0	12	1.1e+02	1	MYGGAAIDILPEAQR
☒	5483	515.2785	2057.0849	2056.9499	0.1350	0	12	1.2e+02	1	HLMYMPVTFFDQNSVGR + Oxidation (M)

☒	6955	500.4247	2996.5045	2997.5741	-1.0696	1	12	74	1	MNILMLTFIICGLLTQVTKGSFEPQK + Oxidation (M)
☒	7970	827.5506	4132.7166	4133.6658	-0.9492	1	12	26	1	CDNCPYNHNPDQADTDKNGEGDACAVDIDGDGILNER
☒	8875	989.2828	5929.6531	5930.8492	-1.1961	0	12	9	1	QFLPFMLILSAICMLGFSASMGAGSTSLFLMIAFYALSGFFQSTGGSCSYSTITK + Oxidation (M)
☒	3234	594.7283	1187.4420	1188.4942	-1.0521	0	11	2e+02	1	SEHSQGMQNR + Oxidation (M)
☒	4764	846.6576	1691.3006	1691.8521	-0.5514	1	11	1.1e+02	1	SVMIVGAGKMCELAAR
☒	6539	547.6423	2733.1751	2734.3157	-1.1406	0	11	74	1	LISTAGAAESNESFNTAAAAAAAAAEQAR
☒	7609	940.4805	3757.8929	3757.7406	0.1523	1	11	44	1	KLLFMGGEFGQQGEWNHDAMLQWSLLDDPAHR + 2 Oxidation (M)
☒	5508	519.2243	2072.8681	2073.1802	-0.3121	1	11	1.1e+02	1	LVRNISTGMAIAGIMLLLR + 2 Oxidation (M)
☒	5973	500.4275	2497.1011	2498.2329	-1.1318	0	11	92	1	EGDVITIIGVPEPGWFEGELEGR
☒	7484	1208.5551	3622.6435	3621.9743	0.6692	1	11	48	1	DGQEVLPVADLPPGVHPANAAAALLAAEALLRHLGR
☒	5803	595.7776	2379.0813	2379.2185	-0.1372	0	11	1e+02	1	SDILTSIAVMAFFGFTFAQIGK + Oxidation (M)
☒	7112	798.8814	3191.4965	3190.5578	0.9387	0	11	65	1	NFMNQTQDKPPVNPAATNVLSGQSPHLNR + Oxidation (M)
☒	7644	764.3862	3816.8946	3815.9606	0.9340	0	11	43	1	MVVVMIVAFMVAWTPYAAFSIVVTAHPSIHLDPR + 3 Oxidation (M)
☒	3342	626.4427	1250.8708	1250.5813	0.2896	0	11	1.5e+02	1	SLLNSMDNVDK + Oxidation (M)
☒	3367	1272.9688	1271.9615	1271.6146	0.3469	0	11	1.4e+02	1	YSVYATQDAVR
☒	4569	541.6442	1621.9108	1621.8199	0.0909	1	11	1.6e+02	1	LAVPADGPEKAETPVE
☒	5261	967.9302	1933.8458	1934.9367	-1.0908	1	11	1.3e+02	1	LLNQMEAESNLASERSK + Oxidation (M)
☒	7713	778.1257	3885.5921	3884.9514	0.6408	1	11	32	1	AGDLVGFVAGQVGGKGGGRPDMAMGGGTDLAALPAAVASVQK + 2 Oxidation (M)
☒	6434	667.5854	2666.3125	2665.2150	1.0975	0	11	92	1	NHGNNEADAALQGLAQQGVDMEDLR
☒	7722	785.3480	3921.7036	3920.8531	0.8505	1	11	35	1	IAMSALVMGCASDTPVKVDDTAFIATSFPDFIPMMR + Oxidation (M)
☒	3552	672.8320	1343.6494	1344.6020	-0.9525	0	11	2e+02	1	NFTEAEIAYCK
☒	3567	676.7616	1351.5086	1350.6714	0.8372	1	11	1.6e+02	1	MDAFRDAILQR + Oxidation (M)
☒	5145	936.9644	1871.9142	1872.0040	-0.0897	1	11	1.5e+02	1	RPKIGVIPMGTVNDFGR + Oxidation (M)
☒	6121	849.7299	2546.1679	2547.3254	-1.1575	0	11	99	1	LMVTAAVAGGISNIQAGYEIPELSK + Oxidation (M)
☒	6168	512.6342	2558.1346	2557.2040	0.9306	0	11	91	1	ASTSASGSADLLMSFGCDLLNVTPK + Oxidation (M)
☒	6451	889.7796	2666.3170	2665.2150	1.1019	0	11	93	1	NHGNNEADAALQGLAQQGVDMEDLR
☒	6544	551.0482	2750.2046	2749.3601	0.8445	0	11	78	1	ITMLEASVAPGGGAWLGGQLMSGMVIR + 3 Oxidation (M)
☒	6834	586.0481	2925.2041	2926.2822	-1.0780	0	11	65	1	NMADDETMEGSTGSGGHIAPPGPAAGTVQR + Oxidation (M)
☒	7211	1110.1749	3327.5029	3328.5737	-1.0709	1	11	54	1	EQVFDASDNLMLALAAMTGMLTDLTANRER + 2 Oxidation (M)
☒	7349	696.7260	3478.5936	3477.6973	0.8963	1	11	52	1	NSSSYPQKPALIDSDTNETLSFSQFKSMVIK + Oxidation (M)
☒	7595	621.7997	3724.7545	3725.8062	-1.0517	1	11	45	1	FPELYKDLDSYPDLGIENYGVSMTTLNEVFLK + Oxidation (M)
☒	5080	928.9438	1855.8730	1855.9792	-0.1062	0	11	1.5e+02	1	GALPLIGVGGIGDGAEAYAR
☒	6606	563.0854	2810.3906	2809.3627	1.0280	1	11	87	1	LTDDQGPVLMTTVAMPVFSKQNETR + 2 Oxidation (M)
☒	3250	602.7407	1203.4668	1202.7275	0.7394	0	11	2.1e+02	1	ILFILVDSGVK
☒	3487	656.3845	1310.7544	1309.6547	1.0997	0	11	1.9e+02	1	LSMSSIETASLR + Oxidation (M)
☒	5344	667.9865	2000.9377	2000.9626	-0.0249	1	11	1.4e+02	1	SVGEAMAIGRTFQESFQK + Oxidation (M)
☒	7212	476.3759	3327.5804	3326.6257	0.9547	1	11	58	1	EFRENIEAGMLGVNIGVPAPMAFFPFSGWK + Oxidation (M)
☒	8674	695.2059	4859.3904	4860.5219	-1.1316	1	11	21	1	SQGVAAVLSGAGPAVLALTTVDLPDSAVKYAEDQGFSLVAMAVSAGVSVR + Oxidation (M)
☒	3241	598.3155	1194.6164	1193.6292	0.9873	1	11	1.9e+02	1	LGKGATYDEIK
☒	3999	725.4726	1448.9306	1449.6340	-0.7034	0	11	1.6e+02	1	AGQSEAGCCLLER
☒	4227	759.0016	1515.9886	1515.7317	0.2569	0	11	1.5e+02	1	DGHAVSIEEYLQR
☒	5066	616.9174	1847.7304	1847.9233	-0.1930	0	11	1.3e+02	1	NLSTAEIVQQVMMVNR + Oxidation (M)
☒	5490	515.9442	2059.7477	2059.0053	0.7424	0	11	94	1	IGIVGCGFVGATAAYAMVMR + Oxidation (M)
☒	7354	876.6166	3502.4373	3502.7868	-0.3496	1	11	43	1	VRHVMGWMLAIMYGLIFLNYGLAIWQGYR + 2 Oxidation (M)
☒	3493	657.5992	1313.1838	1312.7537	0.4302	1	11	1.4e+02	1	TPTTVPMVIARK
☒	3555	673.1973	1344.3800	1343.7044	0.6756	1	11	1.4e+02	1	ALEEALRASQEK
☒	6056	632.0391	2524.1273	2525.2308	-1.1035	1	11	92	1	QGSTILLMGCSNGWASQRWVFK
☒	6101	847.7229	2540.1469	2540.1538	-0.0070	1	11	95	1	MNFGVDVGFYPLGSCTMKYNPK + Oxidation (M)
☒	7603	625.1107	3744.6205	3743.7677	0.8529	1	11	39	1	NVSGQDRIGPGSGFSNFNPNSNPSAWPALVQEGTSR
☒	7953	1031.9633	4123.8241	4123.8370	-0.0129	0	11	29	1	YGSEDNIQAMVTLANDVIMDEETAPTQMPLVDMSHDK + Oxidation (M)
☒	8620	781.1671	4680.9589	4681.2748	-0.3158	1	11	17	1	LMDLGLDDFTVLSGNDNLTLPMISMGAEGVISVVANVDPARMSR + 3 Oxidation (M)
☒	4990	604.3380	1809.9922	1810.7654	-0.7733	0	11	1.5e+02	1	MFSQQAPPEDPCYNK
☒	3402	429.2435	1284.7087	1285.6197	-0.9110	1	11	2.1e+02	1	QKAMDGQTHVR + Oxidation (M)
☒	4324	771.8450	1541.6754	1541.8202	-0.1447	0	11	1.7e+02	1	SPVVEHNLQTPPPK
☒	3546	448.5565	1342.6477	1343.7197	-1.0721	1	11	2e+02	1	RNPGADIIFDVK
☒	3712	702.1876	1402.3606	1402.7126	-0.3520	1	11	1.3e+02	1	GEDKMDVIVLER
☒	5075	618.5862	1852.7368	1851.9183	0.8185	1	11	1.2e+02	1	MKVLAAMSGGVDSSVAAAR + 2 Oxidation (M)
☒	6151	639.7892	2555.1277	2556.2047	-1.0770	1	11	91	1	EVTSATMTVASASVECEVGLESKR + Oxidation (M)
☒	6201	518.4306	2587.1166	2588.2646	-1.1480	0	11	87	1	DFLPSTLEYALSENSDYGVLISR
☒	6607	563.0854	2810.3906	2809.3627	1.0280	1	11	88	1	LTDDQGPVLMTTVAMPVFSKQNETR + 2 Oxidation (M)
☒	6871	740.3531	2957.3833	2958.5004	-1.1171	1	11	76	1	TGVEMEALMSVSIGLLTIYDMVKAIDK + 2 Oxidation (M)
☒	6877	987.1329	2958.3769	2958.5776	-0.2007	1	11	77	1	IMEEIPTPIIIVSAAWVSTEVEKTFR
☒	7926	822.7799	4108.8631	4109.8344	-0.9712	1	11	33	1	AKMVYEMALQLIESESTESPESFESPESSESSEAEVK + Oxidation (M)
☒	8111	1066.4702	4261.8517	4261.1187	0.7330	1	11	27	1	NWGRVPALGTEPMFISDLADAVVESLPYVGAMAVSNLEAR + Oxidation (M)
☒	3408	644.3531	1286.6916	1286.6579	0.0338	1	11	2.1e+02	1	LLDDSRGGEGLR
☒	6045	631.3605	2521.4129	2522.2509	-0.8380	1	11	92	1	MSQETIISNLIDMLKVSAGWDR + Oxidation (M)
☒	6694	717.6068	2866.3981	2867.4595	-1.0614	1	11	88	1	ASMLDEAIEYLKQLQLQVQMLTMR + Oxidation (M)
☒	3049	542.3267	1082.6388	1083.6513	-1.0124	1	11	2e+02	1	QQLATVLRR
☒	5812	601.2398	2400.9301	2400.1375	0.7926	1	11	85	1	MDSAWTALDMETQTMLKTAIK + Oxidation (M)
☒	6132	639.5387	2554.1257	2555.3067	-1.1810	1	11	90	1	SPVTSFAVVHLNGSVDWMVAQRR
☒	7102	792.3494	3165.3685	3166.4926	-1.1241	0	11	59	1	DCPPFAMILMGNFTEHPLINGTQYQLK + 2 Oxidation (M)
☒	7690	771.7740	3853.8336	3852.8440	0.9896	0	11	44	1	DMVEIGEGAVIMMGAVINVGAVIGEGTMIDMNAVIGGR + 4 Oxidation (M)
☒	5968	625.2805	2497.0929	2497.1067	-0.0138	1	11	94	1	ENIDTSKGDVMFAGDGQEASPWK + Oxidation (M)
☒	6353	530.8688	2649.3076	2649.1952	0.1125	1	11	95	1	DHADLGEALKGQGGFPMMDFEAAAR + Oxidation (M)
☒	7929	1028.2233	4108.8641	4109.7399	-0.8758	0	11	33	1	YDQFGHAGVGSSAASGGGQYGAGASDFSDIFSAFNDMFGGGK + Oxidation (M)
☒	5728	766.3093	2295.9061	2296.1798	-0.2737	1	11	91	1	DATTPPSTTSTSTISLAKLNYK
☒	8234	736.1592	4410.9115	4411.2034	-0.2919	1	11	25	1	TRAFITHGGANGIYEAIYHGIPMVGIPLFFDQPDNIAHMK
☒	8254	631.5683	4413.9272	4414.2026	-0.2754	1	11	26	1	MGMSWWLACAAAFSALCVLKASSLDTFLAAVYEHAVILPK + Oxidation (M)
☒	3841	716.4717	1430.9288	1431.6855	-0.7566	1	11	1.7e+02	1	RIHSDTFASGGER
☒	4142	746.0051	1489.9956	1489.7194	0.2762	1	11	1.4e+02	1	ALEAMAESLDRER
☒	5600	726.2292	2175.6658	2176.0834	-0.4176	0	11	83	1	QVQGMNAEIAIASLEFTPNK + Oxidation (M)
☒	5845	608.5432	2430.1437	2430.3006	-0.1569	1	11	1.1e+02	1	AAENGKQIFVEKPLTLSLEESK
☒	6947	600.1100	2995.5136	2995.6688	-0.1552	1	11	78	1	RPPTPTTTRPVMRPRGPVALPPVMTVR + Oxidation (M)
☒	3513	662.0516	1322.0886	1322.5408	-0.4522	0	11	1.3e+02	1	GSTDEMSAPGAER + Oxidation (M)
☒	4452	788.6823	1575.3500	1575.8290	-0.4790	1	11	1.2e+02	1	ASAALGEAMKVVQSSK
☒	4881	580.6065	1738.7977	1737.8515	0.9462	1	11	1.6e+02	1	GADIFGYFFGKAFGNK
☒	4948	594.9765	1781.9077	1781.9635	-0.0559	1	11	1.6e+02	1	QLLEDNKQALDGALVR
☒	5472	1024.0055	2045.9964	2045.2248	0.7716	1	11	1.3e+02	1	LTPEVGIPGAARLIVDALLK
☒	6663	951.4622	2851.3648	2850.4487	0.9161	0	11	86	1	VATAQAWTHLGMKPPANAVGLGQTGLFD
☒	6956	500.4248	2996.5051	2996.5356	-0.0304	1	11	78	1	AIHDLDISSVGQLEPWHPTELDQKIR
☒	3494	438.8483	1313.5231	1313.7303	-0.2072	1	11	2e+02	1	AELDAAVLKATGR
☒	4897	874.1924	1746.3702	1745.8696	0.5006	1	11	1.1e+02	1	AEIEHDLQEEHIKER
☒	5072	926.9233	1851.8320	1851.0465	0.7855	0	11	1.4e+02	1	DIGASLADPSKPLLLSVR
☒	7826	804.7361	4018.6441	4017.9816	0.6625	1	11	30	1	SVAMSPDFLAGGEHYALEVRGDSMIEAGILDGDLVVIR + Oxidation (M)
☒	2639	445.1185	888.2224	888.4930	-0.2705	1	11	2.1e+02	1	LGFGRSPR
☒	4194	753.5217	1505.0288	1505.2805	-0.2517	0	11	1.3e+02	1	QDLITNLYGBLNK
☒	4503	798.3197	1594.6248	1593.7708	0.8540	1	11	1.5e+02	1	MTNWTEIDEIAKK + Oxidation (M)
☒	5368	1009.4697	2016.9248	2017.0480	-0.1231	1	11	1.4e+02	1	SDQLASILLKEAGWTETR
☒	5768	470.4504	2347.2156	2348.2046	-0.9890	1	11	1.1e+02	1	AGLMEGVLGFTGEVNVQGESVKK
☒	4277	768.3144	1534.6142	1533.6625	0.9517	1	11	1.7e+02	1	TREGNMLYMEMK + 2 Oxidation (M)
☒	8715	1112.9745	5559.8361	5559.7452	0.0909	1	11	14	1	MTSAVGTSGTAITSRVHSLNRPNMVSVGTIVWLSSELMFFAGLFAMYFTAR + 3 Oxidation (M)

☒	5525	1048.4425	2094.8704	2094.9508	-0.0803	0	11	1.2e+02	1	YPFGGGAQSDEAHHQPLER
☒	5865	489.0233	2440.0801	2440.2705	-0.1904	1	11	1e+02	1	QLVMGTLEHIEEIDAMIGKVSK
☒	7818	1005.4196	4017.6493	4016.8878	0.7615	1	11	29	1	SDALVGIQDMGAAGLTSSSAEMASKAGSGIEMNLDHVPQR + Oxidation (M)
☒	7144	650.3315	3246.6211	3247.6541	-1.0329	1	11	67	1	GMLGLATKPAIGLLDMTSNVSEGIRNSTDVR + 2 Oxidation (M)
☒	4828	857.1354	1712.2562	1712.9131	-0.6568	0	11	1.1e+02	1	IALPKPSIDTGMGLER + Oxidation (M)
☒	5000	604.3394	1809.9964	1810.9287	-0.9324	1	11	1.5e+02	1	MLYPEKFQGIGISNAK + Oxidation (M)
☒	7375	589.9415	3533.6053	3532.9302	0.6751	1	11	54	1	LAVANVPAPFRGNAIALITAGLMSLAFMGFSGLVK + Oxidation (M)
☒	8103	850.4082	4247.0046	4247.8322	-0.8276	1	11	34	1	IGMDGGTGYVVEFCGEAIEALSMEGRMTVCNMAIEMGAK + 4 Oxidation (M)
☒	3512	662.0056	1321.9966	1321.6799	0.3167	0	11	1.3e+02	1	SLEAILVDMTSK + Oxidation (M)
☒	5821	802.7538	2405.2396	2405.0917	0.1478	0	11	1.1e+02	1	GQSVSGQQRPSSSMSLYGEYNK + Oxidation (M)
☒	4988	453.5053	1809.9921	1810.8697	-0.8776	1	11	1.5e+02	1	YNSDSEKITQTLQER
☒	5689	755.6644	2263.9714	2263.1518	0.8196	1	11	1.1e+02	1	GGELILKEAIQTCDIDYVAR
☒	6680	714.8688	2855.4461	2855.3322	0.1139	0	11	88	1	APESVAAESNIANDAAFEGQQAPQSPTR
☒	7054	1021.8013	3062.3821	3062.3096	0.0725	0	11	70	1	HMDDIISGEFSSTMMADWANGDVNLFK + 2 Oxidation (M)
☒	4220	758.2361	1514.4576	1513.8446	0.6131	0	11	1.3e+02	1	LPYIHFFHTVLK
☒	4946	889.9089	1777.8032	1778.8873	-1.0840	0	11	1.6e+02	1	MAIAESGAFEINVNLGK + Oxidation (M)
☒	5962	831.7588	2492.2546	2491.2570	0.9976	0	11	1.1e+02	1	GLVLIAFSQYLQQCPFDEHVK
☒	6308	523.6379	2613.1531	2612.2071	0.9460	1	11	93	1	QKLLDQGGSTPAHCRPSDMDEVR + Oxidation (M)
☒	7471	722.5295	3607.6111	3608.7590	-1.1478	1	11	49	1	MLNADLKQQLQQLLELMEGDVEFVASLGSDDK + 2 Oxidation (M)
☒	3996	725.1829	1448.3512	1447.7089	0.6423	1	11	1.3e+02	1	RVLGESGEMDALR + Oxidation (M)
☒	4554	810.2587	1618.5028	1618.8349	-0.3320	1	11	1.2e+02	1	MGGKIIVGDLEATGSR + Oxidation (M)
☒	8320	746.1603	4470.9181	4472.1078	-1.1897	0	11	24	1	LNTTGQLAESLGDALMENGAVSVTFQDSHDNPVFEPLPGETR + Oxidation (M)
☒	4081	739.3282	1476.6418	1477.7810	-1.1392	1	11	1.9e+02	1	RSLEAILVDMTSK + Oxidation (M)
☒	4536	803.8472	1605.6798	1604.7715	0.9083	1	11	1.7e+02	1	LEEEEEAKAALMSR
☒	5195	946.7657	1891.5168	1890.9882	0.5286	1	11	1e+02	1	KFAVMGVIPLGCLPMSR + Oxidation (M)
☒	7694	645.8091	3868.8109	3868.0563	0.7546	0	11	45	1	GSVLFSKPLAWAIFSGDQAIAYLTVAGVAAAAQSAAFAK
☒	7890	812.8236	4059.0816	4060.0232	-0.9415	1	11	38	1	FVDVKANGPSSVGLDFSLHGFEHLYGIPQHAESHQLK
☒	8202	1092.2631	4365.0233	4366.0306	-1.0073	1	11	31	1	KLEEVGAMAYTIVVVASASESAAMQYIAPYSGCTMGEYFR + Oxidation (M)
☒	8851	976.1429	5850.8137	5851.8612	-1.0475	1	11	12	1	GGEPVVCLTAYTTPMARLVDAHCDIALVGDSLGMVLHGLPSTIGVSMEMMILHGK + 2 Oxidation (M)
☒	2540	799.8797	798.8724	798.4824	0.3900	1	11	1.3e+02	1	ATKPRAR
☒	3488	657.0889	1312.1632	1312.6081	-0.4449	1	11	1.4e+02	1	AMSDQDRSLFK + Oxidation (M)
☒	4510	798.8127	1595.6108	1595.7802	-0.1693	0	11	1.5e+02	1	YLTLVVFGLMMFM + 2 Oxidation (M)
☒	5293	653.5283	1957.5631	1957.0740	0.4891	1	11	96	1	AIMKEVMNLLQPLNVTK + Oxidation (M)
☒	5350	1001.9817	2001.9488	2001.0895	0.8594	1	11	1.4e+02	1	RLNPTAIILSGGPSSVYEK
☒	5440	1021.1560	2040.2974	2040.9310	-0.6335	0	11	1e+02	1	LASIEDNSFGSNDMVADLK + Oxidation (M)
☒	6944	600.1094	2995.5106	2994.4977	1.0130	1	11	80	1	MKSVFTLSASLAISLLLCCTAQANDHK + Oxidation (M)
☒	7152	814.1154	3252.4325	3251.5923	0.8402	1	11	61	1	ATADYMGMLATVMNALALQDAMRHIGLVSR + 2 Oxidation (M)
☒	3444	434.8471	1301.5195	1302.4778	-0.9583	0	11	2e+02	1	MNEIMDMDEK + 3 Oxidation (M)
☒	5806	798.6981	2393.0725	2393.1903	-0.1178	1	11	1.1e+02	1	YDYGLELYLISVDEGIRGYR
☒	7181	1099.1486	3294.4240	3293.6822	0.7418	1	11	57	1	IVDLMDALSKLPTEGVKPMSHAVDMAIPQR + 2 Oxidation (M)
☒	8850	976.1418	5850.8071	5851.8612	-1.0541	1	11	12	1	GGEPVVCLTAYTTPMARLVDAHCDIALVGDSLGMVLHGLPSTIGVSMEMMILHGK + 2 Oxidation (M)
☒	3343	627.1434	1252.2722	1251.6095	0.6627	0	11	1.4e+02	1	ESGGLYVVGSER
☒	4178	750.1292	1498.2438	1497.7133	0.5305	1	11	1.2e+02	1	EMDDYNLVLKSR + Oxidation (M)
☒	6100	635.5349	2538.1105	2537.8563	0.2542	0	11	95	1	CGNGYLEDGEECDCGEEEECK
☒	7179	1097.8188	3290.4346	3291.5653	-1.1307	0	11	58	1	THLVDIVGTGGDGSHTFNISTCSMFVAAAAGAK
☒	7249	484.0924	3381.5959	3381.6524	-0.0566	1	11	61	1	GFFPVQDNGIIQGTLQAPQSSSYASMAQRQR
☒	7982	1039.1718	4152.6581	4153.0209	-0.3628	1	11	26	1	TAASAQAASQSQAQDVRVEGAFPVTMLPGDGVGPELMHAVK + 2 Oxidation (M)
☒	8158	861.5986	4302.9566	4301.9602	0.9964	1	11	31	1	REMEDTEEIMDSPNRPGNNFLMQVLSFIFIMGNHAR + 4 Oxidation (M)
☒	4596	544.9631	1631.8675	1631.8188	0.0487	1	11	1.8e+02	1	AIEEKLANMEAEIR + Oxidation (M)
☒	5577	538.0198	2148.0501	2148.1399	-0.0898	1	11	1.3e+02	1	NPIASGIRGQELTPQNALNR
☒	6614	704.8235	2815.2649	2814.5652	0.6997	0	11	86	1	MISLGHLLVLGAALFCISLAGIFLNR + Oxidation (M)
☒	6931	600.1085	2995.5061	2996.4979	-0.9918	0	11	81	1	SISAGNIGVIPGYETDFQTGDTLVSSAVAK
☒	7227	1114.6610	3340.9612	3341.6577	-0.6965	1	11	46	1	GPTQKLLWAVEPLDGCGPLHPSWDSLMPPK + Oxidation (M)
☒	5855	812.7509	2435.2309	2436.3297	-1.0989	0	11	1.1e+02	1	LGLVEAEAIPQEEALASILLMAR
☒	6712	574.4896	2867.4116	2868.3820	-0.9704	1	11	91	1	LVGQAIETFNMIEDGDRLMVCLSGGK + Oxidation (M)
☒	6963	600.3093	2996.5101	2996.6488	-0.1386	1	11	81	1	LLTPTHFEFVFKNATPAVSPNLTLLAR
☒	3485	655.8351	1309.6556	1310.6322	-0.9766	1	11	2e+02	1	TVMMPAKATSSR + 2 Oxidation (M)
☒	4241	761.3358	1520.6570	1521.8079	-1.1508	1	11	1.8e+02	1	KIIFAYGEDPEIK
☒	5208	634.9692	1901.8858	1901.9847	-0.0989	0	11	1.5e+02	1	SNVAEALFGVVPASSGEIR
☒	5311	657.9962	1970.9668	1971.0612	-0.0944	1	11	1.4e+02	1	VPLLLERQGMGDFLVER
☒	3973	721.3458	1440.6770	1441.8214	-1.1443	1	11	1.9e+02	1	LMEEIPSALKLAK
☒	3985	722.9108	1443.8070	1444.7245	-0.9175	1	11	2e+02	1	IFRGSHPSIMER + Oxidation (M)
☒	3685	696.5233	1391.0320	1391.7152	-0.6832	0	11	1.4e+02	1	ADMNVAVAITMLK + Oxidation (M)
☒	4355	776.2141	1550.4136	1549.6420	0.7716	0	11	1.2e+02	1	EPEDAVEDFEDGAK
☒	4762	564.6086	1690.8040	1690.8196	-0.0156	1	11	1.7e+02	1	EKPNVRTETLDMDK + Oxidation (M)
☒	6792	580.2562	2896.2446	2897.3937	-1.1491	0	11	75	1	MAQNNEQQQQGQGLIEAGVLDDIINR + Oxidation (M)
☒	8222	1100.7557	4398.9937	4398.0527	0.9410	0	11	29	1	DTFSSGSATGFLIGVGNVGEFLTPMDDPSTATFLSTDGGVTWK + Oxidation (M)
☒	8370	898.5969	4487.9481	4489.0260	-1.0779	1	11	25	1	IDPDSSIAAALMDMYQSMGDALAQQYGGSAAHNTVFPERQGK + 3 Oxidation (M)
☒	3345	628.3807	1254.7468	1253.7092	1.0377	0	11	2.1e+02	1	HLSLLTTLSNR
☒	3447	651.7678	1301.5210	1301.6299	-0.1088	1	11	2.1e+02	1	EYPHRLSGGMR
☒	4082	739.6611	1477.3076	1476.7507	0.5569	0	11	1.3e+02	1	ENSIIMHPAPVNR
☒	4568	811.8488	1621.6830	1620.8583	0.8247	1	11	1.7e+02	1	EKQQHIEQLLAER
☒	6762	719.3323	2873.3001	2873.3840	-0.0839	0	11	84	1	NPFNTATLYSSLPTMNIFGQMQQVR + Oxidation (M)
☒	2289	636.3972	635.3899	636.2901	-0.9002	0	11	2.1	1	MGGGTAK + Oxidation (M)
☒	2535	797.2564	796.2491	797.4395	-1.1904	0	11	79	1	QVLHSSK
☒	3349	629.5735	1257.1324	1256.5666	0.5658	1	11	1.6e+02	1	MATASESSSSKR + Oxidation (M)
☒	3833	714.3260	1426.6374	1426.6367	0.0008	1	11	2e+02	1	GMMMLTRDTAER + Oxidation (M)
☒	7222	1112.1581	3333.4525	3333.5315	-0.0791	1	11	58	1	DWDIEMINQLLQCKKPNDDNGGMVIDEK + Oxidation (M)
☒	5893	823.4158	2467.2256	2468.4162	-1.1906	1	11	1.2e+02	1	LGIARVSLPGGCAIGARPVNLHIK
☒	7120	799.6115	3194.4169	3193.5260	0.8909	0	11	65	1	DVAVSFYYFFLMVAGHPNPGSFPEFVEK
☒	7184	828.1193	3308.4481	3307.7664	0.6817	1	11	59	1	TEIGAGAFIGSNSSLVAPVKIGTGAYVGSGSVITK
☒	7888	1015.7756	4059.0733	4059.0227	0.0506	1	11	39	1	HITLFMDVSMNTEAAQPNAALSQDLMGVELRGIALVR + 3 Oxidation (M)
☒	2982	520.7672	1039.5198	1038.5319	0.9880	1	11	2.2e+02	1	ADGLAGSRHR
☒	3265	610.1816	1218.3486	1218.5968	-0.2481	1	11	1.6e+02	1	FWDMIARHK + Oxidation (M)
☒	6935	749.8840	2995.5069	2995.4671	0.0398	0	11	83	1	VINEPTSLMDIYPTLSYIGGGMLPQDR + Oxidation (M)
☒	4136	743.5372	1485.0598	1485.7762	-0.7164	1	11	1.4e+02	1	IHDCRLFGLDIK
☒	5358	502.5083	2006.0041	2006.1023	-0.0982	1	11	1.5e+02	1	LKQGVVDFVASLLMAPYR
☒	5820	602.3171	2405.2393	2405.2202	0.0191	1	11	1.2e+02	1	MPPFDSKAFLLSVTHQPGVYR + Oxidation (M)
☒	6311	655.8083	2619.2041	2620.3022	-1.0981	1	11	1e+02	1	MQPTNRCLSTLECAAVALSILEK + Oxidation (M)
☒	7262	847.4124	3385.6205	3384.6428	0.9777	1	11	61	1	FHATYSGDEWQAFLQLNQANRAPFSAFLR
☒	5596	724.9910	2171.9512	2172.0375	-0.0863	1	11	1.3e+02	1	ASVEYDTKVQEANWELYK
☒	7676	764.5839	3817.8831	3816.7434	1.1397	1	11	48	1	LLCGGEALKGDGFDNGAWVAPTVFTDCSDEMTIVR + Oxidation (M)
☒	8038	845.6010	4222.9686	4224.0258	-1.0572	0	11	35	1	LVTATEMPVYVMGVTSTQTPFSFSNASPLLTFHWSMSK + 2 Oxidation (M)
☒	8155	861.4016	4301.9716	4302.8612	-0.8896	1	11	32	1	MMQMLQGQGGGGGGLPGLGGMDLQSMMSQMSGLMGGGGGGGGRGR + 3 Oxidation (M)
☒	2516	392.7474	783.4802	783.2779	0.2024	0	11	1.2e+02	1	DIDSGMM + Oxidation (M)
☒	3661	692.4070	1382.7994	1383.7106	-0.9112	1	11	2.1e+02	1	AKEAEAGAAAVGPSR
☒	4279	768.7237	1535.4328	1535.7845	-0.3516	1	11	1.3e+02	1	VFFDIRIGNGDAGR
☒	6184	644.0386	2572.1253	2572.1475	-0.0222	0	11	97	1	GLGGAMVWALDLDDFAGFSCNQGR + Oxidation (M)
☒	7911	683.4436	4094.6179	4094.9646	-0.3466	1	11	29	1	SGIIPGSYSFESVLSAVADFKAPVFEPGGSMQQGMYTPK + Oxidation (M)
☒	3619	683.6170	1365.2194	1364.6871	0.5324	0	11	1.3e+02	1	LGGLPGPGPMSNPR + Oxidation (M)
☒	4041	734.3690	1466.7234	1465.6871	1.0363	0	11	1.9e+02	1	TDWQSSCAILASK

⊠	5583	539.7535	2154.9849	2154.0813	0.9036	1	11	1.3e+02	1	QVFKSAVTSMADVAAEIMTR
⊠	5794	786.3820	2356.1242	2355.0761	1.0481	1	11	1.3e+02	1	ENKENAGMDEQPQPQLQQQK + Oxidation (M)
⊠	6031	838.4018	2512.1836	2511.2356	0.9480	0	11	1.1e+02	1	FEHLPFMELPLVVGYTGSSGSTK + Oxidation (M)
⊠	7900	814.7744	4068.8356	4067.7942	1.0414	0	11	37	1	SNSYPTSWNPSSQVSMPHNPGSTSYPTTSMAGPEPYPK
⊠	6639	942.7593	2825.2561	2826.4343	-1.1782	1	11	87	1	MSMALIVRPFFVPGSAGISGSRNICK + 2 Oxidation (M)
⊠	7096	791.3624	3161.4205	3161.4232	-0.0027	0	11	71	1	NLHVDDQMAVIQYSWMGLMVFAMGWR + 4 Oxidation (M)
⊠	7783	797.1573	3980.7501	3980.9257	-0.1756	1	11	38	1	VVSTFVPGNLNELIMCFDMVNNLPETNAIQNGMRK + Oxidation (M)
⊠	2574	417.1426	832.2706	832.4039	-0.1333	0	11	2.6e+02	1	SGGGEGLTR
⊠	4504	798.3201	1594.6256	1593.7708	0.8548	1	11	1.6e+02	1	MTNWTEIDEIAKK + Oxidation (M)
⊠	6397	667.5836	2666.3053	2666.2729	0.0324	0	11	1e+02	1	QFLNYTLTGVGGFMAASMLMPMVR + 2 Oxidation (M)
⊠	4140	745.0244	1488.0342	1486.9599	1.0743	0	11	1.4e+02	1	VLRPDVLLIKPPK
⊠	4165	747.9652	1493.9158	1493.7766	0.1393	1	11	1.8e+02	1	KYGEPLPTVFSEK
⊠	7156	1085.1558	3252.4456	3251.5923	0.8533	1	11	65	1	ATADYMGMLATVMNALALQDAMRHIGLVSR + 2 Oxidation (M)
⊠	7838	673.9673	4037.7601	4037.1408	0.6194	1	11	35	1	DELLADLLAAVQATPEAGPHAGPMLFRAGIAGPEAVALLR + Oxidation (M)
⊠	5172	628.3101	1881.9085	1881.9295	-0.0210	0	11	1.6e+02	1	RPCFSALTVDETYVPK
⊠	7584	745.7769	3723.8481	3725.0044	-1.1563	1	11	53	1	LLREFTVTLSVAIGISLLVSLTLTPMMCGWMLK + 2 Oxidation (M)
⊠	7803	667.9494	4001.6527	4001.9797	-0.3270	0	11	32	1	SDRPTVTLLGEMFPADPMVIGAMLAPMGLAVGPTVPMR + 4 Oxidation (M)
⊠	3733	705.6328	1409.2510	1409.7878	-0.5368	1	11	1.4e+02	1	EDVPPGTSILRVK
⊠	4497	797.5978	1593.1810	1592.8246	0.3565	0	11	1.3e+02	1	VIFLSCSHPLHQR
⊠	4936	886.7872	1771.5598	1771.0607	0.4991	1	11	1.2e+02	1	VGSLQYLALTALVAPKK
⊠	5753	777.3602	2329.0588	2328.2437	0.8150	1	11	1.2e+02	1	VVPVEYAEIVASIASRSAGPGTR
⊠	6128	639.3160	2553.2349	2552.2065	1.0284	0	11	1.1e+02	1	TQDGILSGVEEFSTLSMPQDVQR + Oxidation (M)
⊠	6470	671.3635	2681.4249	2681.2798	0.1451	1	11	99	1	GLVIEMVLGTDMTNHFQQMKAMR + 2 Oxidation (M)
⊠	4507	798.5919	1595.1692	1594.7780	0.3913	1	11	1.2e+02	1	KDIFAWGDDPYLR
⊠	4708	834.9361	1667.8576	1666.8566	1.0010	1	11	1.8e+02	1	EFEEGRLSYLSLPK
⊠	5607	1095.5234	2189.0322	2188.0391	0.9931	0	11	1.4e+02	1	EVTEEEMLQALMTAHEAIK + Oxidation (M)
⊠	8599	778.0252	4662.1075	4661.1719	0.9356	1	11	24	1	VGSWIDGDGNHVEMGLHVFFGCYYQLFELMNKVGAFSHLR + 2 Oxidation (M)
⊠	8614	669.5734	4679.9629	4679.4341	0.5288	1	11	20	1	APGSLAGARIVVAMSGGVDSSVVAALAAASGAETIGVTLQLYDHGAAVGR + Oxidation (M)
⊠	4044	735.2337	1468.4528	1468.7088	-0.2559	1	11	1.4e+02	1	MIKSDLEISMMR + Oxidation (M)
⊠	6057	842.6677	2524.9813	2524.2122	0.7691	0	11	80	1	AYVDPETGNELSSFPADPFILSR
⊠	6802	970.4625	2908.3657	2908.4222	-0.0566	1	11	87	1	DKINSLNTNSSLELSESLDILEAEMK + Oxidation (M)
⊠	7153	814.1160	3252.4349	3251.5923	0.8426	1	11	65	1	ATADYMGMLATVMNALALQDAMRHIGLVSR + 2 Oxidation (M)
⊠	2741	476.2275	950.4404	951.4266	-0.9862	0	11	2.5e+02	1	CLSAAGAMR + Oxidation (M)
⊠	5302	982.4746	1962.9346	1962.0547	0.8799	1	11	1.5e+02	1	IQPNEAIILGARGNFHGR
⊠	7019	757.3245	3025.2689	3024.4498	0.8191	1	11	67	1	LYDSMIQELDGVDNGVERYPSDIKPR + Oxidation (M)
⊠	8130	859.6101	4293.0141	4294.0187	-1.0046	1	11	34	1	TTDVTGSCELPEGVEMVMPGDNVKMTVSLIAPIAMEDGLR + 2 Oxidation (M)
⊠	6142	512.0325	2555.1261	2555.1962	-0.0701	0	11	1e+02	1	YEQPNGNLQCQYSSQDIISALK
⊠	7730	788.7683	3938.8051	3937.9596	0.8455	1	11	42	1	ASKAMVEIAGVDGIOIGVGDPMGMPISHIMASGMTGIR + 2 Oxidation (M)
⊠	8206	876.6185	4378.0561	4379.2359	-1.1798	1	11	32	1	VSVFVEVEGAGDFLPPYAGNLDIMTAATKVGEVIAGQITSAR + Oxidation (M)
⊠	3651	691.3361	1380.6576	1381.7315	-1.0739	0	11	2.1e+02	1	MIAFIEPFLASK + Oxidation (M)
⊠	5137	468.2626	1869.0213	1867.9574	1.0639	1	11	1.6e+02	1	CEVLSKAVNEISNHLR
⊠	2502	776.2659	775.2586	775.4076	-0.1489	0	11	1.7e+02	1	ATASALDK
⊠	3571	677.7636	1353.5126	1353.6962	-0.1836	1	11	1.8e+02	1	NEVMSFVKSGLK + Oxidation (M)
⊠	4965	895.8234	1789.6322	1789.7941	-0.1618	0	11	1.3e+02	1	NNQAFMFSDTNSSAIK + Oxidation (M)
⊠	6753	1437.6528	2873.2910	2872.3583	0.9328	0	11	87	1	AETNPETGQTLISGMGELHLDIMVER + 2 Oxidation (M)
⊠	6958	750.1338	2996.5061	2997.6331	-1.1271	1	11	86	1	KMWPLVMPITIILLVMIFMISSYSR + Oxidation (M)
⊠	2487	767.2376	766.2303	766.3280	-0.0976	0	11	53	1	DVSGSMR + Oxidation (M)
⊠	3722	704.3284	1406.6422	1407.6412	-0.9990	1	11	2.1e+02	1	EASARVTEDMAGR + Oxidation (M)
⊠	4139	744.9490	1487.8834	1486.8759	1.0075	0	11	2e+02	1	LVLILEHDTPIPK
⊠	5559	427.2165	2131.0461	2129.9721	1.0740	1	11	1.4e+02	1	DGGMNMLAALGDKSGNEPPPK + 2 Oxidation (M)
⊠	5966	833.0378	2496.0916	2496.2867	-0.1951	1	11	1.1e+02	1	QTTSNSGLMNKANIQALIHGLNR + Oxidation (M)
⊠	6135	852.3829	2554.1269	2553.3472	0.7797	0	11	1e+02	1	GQINAALEVMVELGLNDANLVGVAK + Oxidation (M)
⊠	3509	660.8353	1319.6560	1320.6707	-1.0147	0	11	2.3e+02	1	MASTSNLLDVVR + Oxidation (M)
⊠	4463	790.3265	1578.6384	1578.8042	-0.1658	1	11	1.7e+02	1	YDGGHKLTGYIVEK
⊠	5493	1031.8833	2061.7520	2061.0338	0.7182	1	11	1.1e+02	1	ENSITVSQGTLEQDVSKAR
⊠	5524	1048.2513	2094.4880	2095.1026	-0.6145	1	11	97	1	ITILGAGKVGMATAVMLMMR + 2 Oxidation (M)
⊠	5669	560.2664	2237.0365	2236.1257	0.9108	0	11	1.3e+02	1	TETSIGTGAVSVSSAAVELAQMK
⊠	6472	537.4688	2682.3076	2682.4138	-0.1062	1	11	1e+02	1	LNAAVFPGAQGGPLMHVIAAKAVCFK + Oxidation (M)
⊠	7327	580.6027	3477.5725	3477.8922	-0.3197	1	11	58	1	IMLLRSALGMAIVMLLMGMAQNIWQFLILR + 2 Oxidation (M)
⊠	4798	847.4259	1692.8372	1691.9069	0.9303	1	11	1.8e+02	1	AIMGFPKDFVIPVSR + Oxidation (M)
⊠	7046	611.8654	3054.2906	3053.4851	0.8055	1	11	70	1	SFMHWIDARAVVPVIQDLHESSETMR
⊠	3614	455.2282	1362.6628	1361.6761	0.9866	0	11	2.2e+02	1	HLSTILECAYR
⊠	4052	491.5375	1471.5907	1472.7835	-1.1928	0	11	1.9e+02	1	IGSGVIVLGSSNQDK
⊠	8094	850.4056	4246.9916	4247.0919	-0.1003	1	11	37	1	VHEMLPCTILGAKPEPKVVTLGNDYDYVWDISAAAGSSK + Oxidation (M)
⊠	6927	600.1082	2995.5046	2994.4977	1.0070	1	11	87	1	MKSVFTLSASLAISLLLCCTAQANDHK + Oxidation (M)
⊠	7800	801.1376	4000.6516	3999.9120	0.7396	0	11	32	1	SFTNGQLANYRPGINTILDNTSELSSPTSTTNQMSPK + Oxidation (M)
⊠	7947	825.0234	4120.0806	4120.1390	-0.0584	1	11	40	1	ALPAMRALVDVWHPDLVIAEPMELAGPAAATNAGVPWVR + Oxidation (M)
⊠	8129	1074.0131	4292.0233	4292.0573	-0.0340	1	11	35	1	TTDVTGVVKLPEGTEMVMPGDNVDMSVTLIQPVAMDEGLR + 3 Oxidation (M)
⊠	8526	913.9859	4564.8931	4565.3496	-0.4564	1	11	22	1	LLELTIDPEIMDDVDMVQDLVVAAVNKALEASAQLAQSEIQK
⊠	8644	790.1567	4734.8965	4735.3010	-0.4045	1	11	20	1	DELTKTTAVAEHHVGVNNGGMDQAASICGIEGHALYVEFKPELK
⊠	3206	584.3549	1166.6952	1167.6248	-0.9295	0	11	2.2e+02	1	SRPAPSPESIK
⊠	4326	772.6647	1543.3148	1542.6959	0.6190	0	11	1.4e+02	1	WGSMMEQYLINR + Oxidation (M)
⊠	5021	907.1089	1812.2032	1811.9604	0.2429	1	11	1.2e+02	1	LLILTFDSFGERMVR + Oxidation (M)
⊠	5926	621.7883	2483.1241	2484.2642	-1.1401	1	11	1.2e+02	1	VMVEAEQVETAERAAQELAAALR
⊠	3159	577.3646	1152.7146	1153.6091	-0.8945	1	11	2.1e+02	1	TYSSNSIKVR
⊠	3583	680.0141	1358.0136	1357.7565	0.2572	1	11	1.5e+02	1	STQAAAGIAALKEK
⊠	3713	702.3781	1402.7416	1402.7721	-0.0305	1	11	2.2e+02	1	GLWKVAYVQPSR
⊠	3755	355.4379	1417.7225	1418.7405	-1.0180	1	11	2.2e+02	1	DQGSKFLGLTPEK
⊠	5545	1058.0148	2114.0150	2113.1024	0.9127	1	11	1.5e+02	1	MATGVDHNRISLLMAVLEK + Oxidation (M)
⊠	6352	442.5568	2649.2971	2650.2659	-0.9687	1	11	1.1e+02	1	MYGKLSGMTGTAVTEAGELWEIYK + Oxidation (M)
⊠	7424	596.6439	3573.8197	3574.7760	-0.9563	0	11	61	1	NTGLTQYPTLDAAGIGAQCDLALVVGGDGTMLGIGR
⊠	8307	892.8211	4459.0691	4458.1051	0.9640	1	11	31	1	ELPLPMMNILNGGAHADNNVDIQEFMIMPAGARCFAEALR + 2 Oxidation (M)
⊠	8716	927.6468	5559.8371	5559.9090	-0.0719	1	11	15	1	MNPLIFIMLILTIILGTSIILTSTHWFMIWLGFEMNMMAMIPVLMKK + 4 Oxidation (M)
⊠	5421	508.7745	2031.0689	2030.0909	0.9780	1	11	1.5e+02	1	RTIVALLNDSWQTSTLGR
⊠	6177	643.7865	2571.1169	2572.1475	-1.0306	0	11	1e+02	1	GLGGAMVWALDLDDFAGFSCNQGR + Oxidation (M)
⊠	6469	671.3333	2681.3041	2680.2625	1.0416	1	11	1.1e+02	1	GPGTGKTLPEEAYVMMGDHVGYALR + 2 Oxidation (M)
⊠	6491	540.6671	2698.2991	2698.4961	-0.1970	0	11	1e+02	1	YAAISGIVAFVGLMFGWVIFPAILK + Oxidation (M)
⊠	7683	955.4827	3817.9017	3818.7042	-0.8025	1	11	51	1	NGKGMDAATADYIGMLGTVMNAMALQDAMEAAGQPTR + 3 Oxidation (M)
⊠	7764	993.2294	3968.8885	3968.7139	0.1746	0	11	45	1	AFEETQIDIPGGGSHGGSSMSLWEVMQSDMIPEDQR + 3 Oxidation (M)
⊠	7901	817.5698	4082.8126	4083.9425	-1.1299	1	11	37	1	RLNYDGIYTPPSENGTLVFPGNLGVFEWGGMSVNPDR + Oxidation (M)
⊠	5200	633.3079	1896.9019	1896.0251	0.8767	0	11	1.6e+02	1	LVGGGSMQIVNGTVPQALR
⊠	6571	694.3047	2773.1897	2774.3255	-1.1359	0	11	88	1	QTVEPEPQPQPMPVDDGGPGCLGIIK + Oxidation (M)
⊠	8256	883.7934	4413.9306	4413.9557	-0.0250	1	11	29	1	VASTMSGGANTMIEHGDTLPSQLGTMVINTEDEEEEGTMKR + 3 Oxidation (M)
⊠	5584	719.9476	2156.8210	2157.1946	-0.3736	1	11	1.1e+02	1	VPNRDAIVGAFVAALLFEAGK
⊠	7084	625.5508	3122.7176	3122.5733	0.1443	1	11	75	1	ELVTFVVATRDDIEIPDEFIMLSVDEK
⊠	8560	923.0194	4610.0606	4610.1734	-0.1128	0	11	25	1	QPIINGGINVEIGNPSYNMYEVDHDHSDGGLLEPSFMIDPVK
⊠	3380	640.3205	1278.6264	1278.6013	0.0251	0	11	2.3e+02	1	SIEISEEMPTK + Oxidation (M)
⊠	5916	825.7192	2474.1358	2475.2493	-1.1135	1	11	1.2e+02	1	KPIPDDQKLPPDAKPSASEGEEK
⊠	8245	1104.2374	4412.9205	4414.1112	-1.1907	1	11	28	1	MRNEIQNETAQTDQTQGSMFSFFNLFPFLLPMFEVIK + Oxidation (M)
⊠	6461	667.8357	2667.3137	2666.2729	1.0408	0	11	1.1e+02	1	QFLNYTLTGVGGFMAAGMLMPMLR + 3 Oxidation (M)

☒	7981	1039.1697	4152.6497	4152.0945	0.5552	1	11	28	1	LGEGVSFAGIGGESMRAEGLTSLFPMTELSVMGLVEVLPR + Oxidation (M)
☒	5246	964.1576	1926.3006	1926.9146	-0.6139	1	11	1.1e+02	1	SFRDDPAMIGGIGYLDGK + Oxidation (M)
☒	5807	599.2755	2393.0729	2392.1580	0.9149	1	11	1.2e+02	1	VQLADKYLNEAALGEANEDAMK
☒	6597	562.6526	2808.2266	2807.2641	0.9626	1	11	91	1	HNASRTPSATELAHANNAAMQAEDER + Oxidation (M)
☒	7017	757.0800	3024.2909	3023.4303	0.8606	1	11	74	1	FCYLMYTSGSTGKPKGVCGTEQGLLNR
☒	7350	580.9378	3479.5831	3479.6234	-0.0402	1	11	60	1	VWLSGSIAFQQTCMDSFGEIKSNLMQDMLK + Oxidation (M)
☒	4672	824.5170	1647.0194	1645.8788	1.1407	1	11	1.7e+02	1	AALRIYDVDGLGLDR
☒	4793	564.9848	1691.9326	1691.8591	0.0735	1	11	1.9e+02	1	NDNNIGVGASHEPLKK
☒	5399	674.3402	2019.9988	2020.0161	-0.0174	1	11	1.6e+02	1	RVYLLMEYLVNGEMYK
☒	7138	806.8577	3223.4017	3223.6871	-0.2854	1	11	66	1	VGTKTIDSVQATQDLVGCTHVEGSLILNLR
☒	7285	684.9172	3419.5496	3419.5254	0.0242	1	11	63	1	AMRESDSCKPAIFAMSNPTLNAECTAADAFK + Oxidation (M)
☒	3563	675.8671	1349.7196	1350.6303	-0.9107	0	11	2.2e+02	1	VTFADVAGADEEK
☒	4869	866.4345	1730.8544	1731.8138	-0.9593	1	11	1.9e+02	1	MYTAEAVEYVADSRK
☒	5292	653.4871	1957.4395	1957.9355	-0.4960	1	11	1.1e+02	1	NGDRGETGPAGPAGPPGPAGAR
☒	7284	684.9170	3419.5486	3420.5798	-1.0311	0	11	63	1	GGNAADAMVATMFCIGVVGMYHSGIGGGGFMLIK + 2 Oxidation (M)
☒	6208	432.8902	2591.2975	2592.4289	-1.1313	1	11	1.1e+02	1	RQVDFLIHALLNNVSVTFTLHR
☒	6940	600.1089	2995.5081	2994.4977	1.0105	1	11	89	1	MKSVFTLSASLAISLLLCCTAQANDHK + Oxidation (M)
☒	6969	600.5094	2997.5106	2998.5434	-1.0327	1	11	88	1	MSPSAVDDAPKATGAAISVKPNIGVFTNPK + Oxidation (M)
☒	7105	634.2806	3166.3666	3166.4926	-0.1260	0	11	68	1	DCPPFAMILMGNFTEHPLINGTQYQLK + 2 Oxidation (M)
☒	8396	1129.7755	4515.0729	4514.1644	0.9085	1	11	30	1	SGQMMLAQGLLLHLMPTDWRWSDCHALTDVDFEVLKPR + 3 Oxidation (M)
☒	8629	940.4003	4696.9651	4696.4583	0.5068	1	11	20	1	IMEMIAPLGPVYQAGTLSGNPVAMAAGLATLKLIQAPGFYEHLSR
☒	5578	430.6173	2148.0501	2146.9550	1.0951	1	11	1.4e+02	1	LAEQAERYEEMVEFMEK + Oxidation (M)
☒	7229	837.8579	3347.4025	3346.6261	0.7764	1	11	55	1	MNVGQILETHLGWACAGMGRQIGELIDAYK + Oxidation (M)
☒	7463	899.4153	3593.6321	3592.7098	0.9223	1	11	62	1	KDPTSTMSALQLAAVTQPGTGGSVTGGGGAAGGGGSAAGMK + 2 Oxidation (M)
☒	8724	927.8155	5560.8493	5559.9090	0.9403	1	11	15	1	MNPLIFIMLILTIILGTSIILTSTHWFMIWLGFEMNMMAMIPVLMKK + 4 Oxidation (M)
☒	2347	675.3679	674.3606	673.3507	1.0099	0	11	31	1	TGGGLNR
☒	4694	554.2529	1659.7369	1658.9104	0.8265	1	11	2e+02	1	SEVARLVLTASGGPFR
☒	5269	648.9808	1943.9206	1942.9380	0.9826	1	11	1.6e+02	1	MTEEEIYARDMGLLLK + 2 Oxidation (M)
☒	5580	717.0244	2148.0514	2147.0319	1.0195	1	11	1.4e+02	1	EIFPNTLLAEDFMVYKMA + Oxidation (M)
☒	7733	789.7319	3943.6231	3942.9132	0.7099	1	11	35	1	EDENPKVAEVIYPIMQVNDIHYLGVDVAVGGMEQR + Oxidation (M)
☒	4908	875.9867	1749.9588	1749.7952	0.1637	1	11	1.9e+02	1	SKLMQGSQNGQNGEEK + Oxidation (M)
☒	5791	590.0374	2356.1205	2356.2346	-0.1142	1	11	1.4e+02	1	IHLTTRTVSSTLEASNSDGIVR
☒	3657	692.0889	1382.1632	1381.7275	0.4357	1	11	1.5e+02	1	MELRLYDTLTK
☒	3987	723.3388	1444.6630	1445.7110	-1.0480	0	11	2.2e+02	1	ATSAAQGAALDESVR
☒	4000	725.5031	1448.9916	1449.8052	-0.8135	1	11	1.6e+02	1	ATELQGHIQKVAR
☒	5923	620.0452	2476.1517	2477.1077	-0.9560	0	11	1.2e+02	1	HQANPNRPDMMLGSDNPNWGAR
☒	6387	534.0208	2665.0676	2665.2914	-0.2238	1	11	83	1	EVIAFPKTQSAACVMTQAPGMVDAK + Oxidation (M)
☒	7208	666.3223	3326.5751	3327.4765	-0.9014	0	11	69	1	DDDVVFLNYGYEEDPPMGIPLSESDELNR
☒	2272	626.8878	625.8805	626.3500	-0.4695	0	11	1.1	1	GAPIGGR
☒	3225	592.6474	1183.2802	1182.7158	0.5644	1	11	1.6e+02	1	IAQKLCPLLK
☒	4523	801.7379	1601.4612	1600.9044	0.5568	1	11	1.4e+02	1	NQIQMMLKILLPK + 2 Oxidation (M)
☒	5093	932.4568	1862.8990	1862.9924	-0.0934	0	11	1.8e+02	1	AQGLLQAFDLTAMLVTR + Oxidation (M)
☒	5561	533.9750	2131.8709	2131.0087	0.8622	1	11	1.3e+02	1	NFPCGKSMMYGANMIILGK
☒	7799	667.7820	4000.6483	3999.8830	0.7654	1	11	33	1	QDLSTGQVQILSESGTDESPSLAPNGKMAIYAMEYGGR
☒	2468	377.8099	753.6052	753.3116	0.2937	0	11	43	1	MQAAFGN + Oxidation (M)
☒	2645	445.2863	888.5580	889.4658	-0.9077	0	11	3.3e+02	1	YGLSPPTR
☒	3443	651.7670	1301.5194	1301.6299	-0.1104	1	11	2.3e+02	1	EYPHRLSGGMR
☒	5486	686.7245	2057.1517	2056.0549	1.0968	0	11	1.4e+02	1	DDNGKPQIAATLNGTLATTR
☒	5814	802.7379	2405.1919	2405.2015	-0.0097	1	11	1.3e+02	1	ELADIFNSFYHAEPVLRAEGK
☒	4202	755.8356	1509.6566	1510.7627	-1.1061	0	11	2e+02	1	DQLAAIDPDSLTPR
☒	5141	624.5952	1870.7638	1870.7868	-0.0230	0	11	1.5e+02	1	GVAVVMEASHMCMVMR + 4 Oxidation (M)
☒	5795	787.0328	2358.0766	2357.1243	0.9523	0	11	1.3e+02	1	ENSYDINVMMTSLLTNLVER + Oxidation (M)
☒	8914	1528.2297	6108.8897	6109.8523	-0.9626	0	11	11	1	VVSVIGDGAMTGGMVFEAMNHAGDLHNDMLVVLNDNEMSISENVGALNNHLAQLMSGR + Oxidation (M)
☒	5044	611.5931	1831.7575	1831.7934	-0.0360	0	11	1.7e+02	1	EMSDFFEATVAQGADAK + Oxidation (M)
☒	5313	986.9622	1971.9098	1971.8495	0.0603	0	11	1.6e+02	1	WPGCESICEDFGQFLK
☒	2657	452.7037	903.3928	903.4848	-0.0919	0	11	3.3e+02	1	MGSIIVER
☒	5312	986.7430	1971.4714	1971.9659	-0.4944	0	11	1.1e+02	1	MTALQHVAVLMGGWSSER
☒	5531	525.4891	2097.9273	2097.1252	0.8021	1	11	1.5e+02	1	MDKGVGSLTTVIVQNGTLHK
☒	6218	520.2676	2596.3016	2595.2713	1.0303	0	11	1.2e+02	1	MNPTQKPEPVYDMVILGASGFTGK + Oxidation (M)
☒	6249	653.3304	2609.2925	2609.3701	-0.0776	0	11	1.1e+02	1	DPNAPGIQLPGQVTISGSELYNVIK
☒	6435	667.5854	2666.3125	2665.2150	1.0975	0	11	1.1e+02	1	NHGNNEADAALQGLAQQGVDMEDLR
☒	6477	895.4054	2683.1944	2684.2938	-1.0994	1	11	1e+02	1	QEELNAQNLVKMQELMSNFFQK + Oxidation (M)
☒	7684	955.4835	3817.9049	3817.9593	-0.0544	1	11	53	1	ALVAALEGVNIDEAIQKAAMPMAVAAAPAAAAAAPAEEAK + 2 Oxidation (M)
☒	8235	883.3843	4411.8851	4411.0072	0.8780	1	11	28	1	TALEDAASVASLLITTEAMIADKPEPKSPAGGPGMGGMGGMDGMM + 3 Oxidation (M)
☒	3400	643.2668	1284.5190	1285.5465	-1.0274	0	11	2.3e+02	1	MMMSSSQTPVR + 2 Oxidation (M)
☒	4435	785.5208	1569.0270	1568.8596	0.1675	0	11	1.6e+02	1	TAEAAKPAGVMLGLPK + Oxidation (M)
☒	8465	647.8600	4527.9691	4527.0148	0.9543	1	11	28	1	LNPEDFSVDIEGEASCTGKTGVEMEAMTAVSVAGLTVYDMCK + Oxidation (M)
☒	4370	779.8700	1557.7254	1557.7756	-0.0501	0	11	2.1e+02	1	IQAICVVCGNPATR
☒	5315	658.6641	1972.9705	1972.9888	-0.0183	1	11	1.6e+02	1	EKGLTTVLVTHSMEDAAR + Oxidation (M)
☒	6215	520.2207	2596.0671	2597.2447	-1.1776	1	11	93	1	AAAAVARTIAEVVMMCHSNGVMHR + Oxidation (M)
☒	6248	435.8893	2609.2921	2608.2625	1.0296	1	11	1.1e+02	1	YVLCQTDVKSGNGQSGAQPVLSMGL
☒	7766	794.7855	3968.8911	3968.7139	0.1772	0	11	47	1	AFEETQIDIPGGGSHGGSSMSLWEVMQSDMIPEDQR + 3 Oxidation (M)
☒	7795	999.7139	3994.8265	3995.8968	-1.0703	0	11	43	1	AIQLHPLVCAAFNADFDGDQMAVHVPLSIEAQMEAR + 2 Oxidation (M)
☒	4217	757.6644	1513.3142	1513.7446	-0.4304	0	11	1.5e+02	1	LPEGEPIEAGMVTR + Oxidation (M)
☒	5610	548.4839	2189.9065	2189.1341	0.7724	1	11	1.3e+02	1	HEGKLHLIAGAFQDAGNAVNK
☒	5667	745.9999	2234.9779	2235.1868	-0.2089	1	11	1.4e+02	1	LVQPVIEQMMAARGLETPPR
☒	5763	586.4819	2341.8985	2342.0817	-0.1832	1	11	99	1	SDGAQANLRCAAYQALMDMIK + Oxidation (M)
☒	7091	630.5070	3147.4986	3146.5263	0.9723	1	11	84	1	ALEEKFGVSAAPAVMAGAVMAAPAGEAAAAEEK + 2 Oxidation (M)
☒	7185	1103.8246	3308.4520	3307.5277	0.9243	0	11	66	1	LSSGFGGGGGGSGSGGGSGGGGLGSSSHPLSSLLSMWR
☒	7671	764.3884	3816.9056	3817.9309	-1.0253	0	11	53	1	VSLTGIQPYLTDTINAIPSAAGQTANCTQGPFGSLVR
☒	7692	964.4698	3853.8501	3853.9600	-0.1099	1	11	53	1	LTYLDYYLDNIIDLFNCLANASSFSLVSVNIKR
☒	7832	808.3602	4036.7646	4037.1408	-0.3762	1	11	40	1	DELLADLLAAVQATPEAGPHAGPMLFRAGIAGPEAVALLR + Oxidation (M)
☒	3028	534.2536	1066.4926	1065.5567	0.9359	1	11	2.4e+02	1	TTVYGRVDR
☒	4195	753.9082	1505.8018	1506.8089	-1.0071	1	11	2.2e+02	1	VCGTPILAGKHAQR
☒	4724	559.9634	1676.8684	1677.8573	-0.9890	1	11	2e+02	1	TFDISSISEPKDAIR
☒	5782	786.0485	2355.1237	2354.1325	0.9912	1	11	1.4e+02	1	HLVDEPQNLIKQNCDQFEK
☒	7480	724.1165	3615.5461	3615.8445	-0.2983	1	11	50	1	KMGGLHSAAPITSVCFFIGSAALMGVPFLAGFFSK
☒	7664	637.1578	3816.9031	3816.7434	0.1598	1	11	53	1	LLCGGEALKGDGFDNGAWVAPTVFTDCSDEMTIVR + Oxidation (M)
☒	8384	901.3965	4501.9461	4501.1294	0.8168	1	11	27	1	DIISGEVFGHKLGGYAGVINVGMDDTWLGSHLAMSNMFAYGR + Oxidation (M)
☒	2495	385.7221	769.4296	768.4858	0.9439	1	11	1.1e+02	1	QVLKGPK
☒	3050	542.3269	1082.6392	1083.5495	-0.9103	0	11	2.4e+02	1	QQLHTMAQK
☒	3376	638.9370	1275.8594	1275.5475	0.3119	0	11	2.1e+02	1	AMVTQYGMSEK + 2 Oxidation (M)
☒	3654	691.7360	1381.4574	1380.6521	0.8053	1	11	1.7e+02	1	VKEQESVDYER
☒	3708	701.6490	1401.2834	1401.7212	-0.4377	1	11	1.6e+02	1	IAEEINSDSRLR
☒	5550	530.7689	2119.0465	2118.0481	0.9984	1	11	1.5e+02	1	DDGPAIAIDLTGFSEKNDLK
☒	5565	1069.7559	2137.4972	2136.9818	0.5154	1	11	1e+02	1	RSQQGLSHVTSTGSSSSMER + Oxidation (M)
☒	3516	663.7816	1325.5486	1326.6932	-1.1445	0	11	2.2e+02	1	LHVDELEGFLR
☒	6263	523.2360	2611.1436	2612.3018	-1.1582	1	11	1e+02	1	MNIKVPIYFSSGLTIQANMYYK + 2 Oxidation (M)
☒	7335	580.6039	3477.5797	3476.5889	0.9908	1	11	63	1	EIETTLRQNAGLIAEQFLDDGGPEEDAAGAAEM + Oxidation (M)
☒	3033	540.3286	1078.6426	1079.5862	-0.9436	1	11	2.6e+02	1	LEEIAKSYK
☒	3282	414.5966	1240.7680	1241.5169	-0.7489	0	11	2.4e+02	1	GTSTGWMPMSR + 2 Oxidation (M)

5009	453.7563	1810.9961	1811.9378	-0.9417	1	11	1.8e+02	1	IDDKVSIPGPVTGTGNSR
5365	673.0943	2016.2611	2017.0112	-0.7501	0	11	1.2e+02	1	MGVPLSLGEGSIIEPDIMK + 2 Oxidation (M)
5752	776.1392	2325.3958	2326.2016	-0.8058	1	11	1.1e+02	1	DSRILILDEPTASLTPAETER
6124	638.7755	2551.0729	2550.3549	0.7180	1	11	1e+02	1	LKPSAAKGTYLQAMALATTMGPGIK + 2 Oxidation (M)
8604	934.4233	4667.0801	4667.1514	-0.0713	1	11	26	1	MMGMALSETPLMDFTHTACKTEGWAEQVINQWEITAAEVPK + Oxidation (M)
2731	471.1999	940.3852	940.5202	-0.1350	1	11	2.4e+02	1	AEGGKRPAR
3439	651.5893	1301.1640	1301.6212	-0.4571	0	11	1.7e+02	1	ENSGGGVEGAGIQK
5515	693.9329	2078.7769	2079.9275	-1.1506	1	11	1.2e+02	1	ATQADLMELDMAMEPDRK + Oxidation (M)
2570	412.7451	823.4756	822.3541	1.1215	0	11	1.7e+02	1	MAEATER + Oxidation (M)
3644	460.5590	1378.6552	1377.7061	0.9491	0	11	2.3e+02	1	VLETMLGEELTK + Oxidation (M)
4607	819.2979	1636.5812	1635.8733	0.7080	1	11	1.5e+02	1	SIGADAFALFVKNQR
3453	652.8841	1303.7536	1302.6860	1.0677	0	11	2.5e+02	1	YFPDPDLLPVK
6093	844.7433	2531.2081	2530.3135	0.8946	0	11	1.2e+02	1	TLSNMGILQALEVILGMDDVQVR + Oxidation (M)
6348	530.6669	2648.2981	2647.2119	1.0862	0	11	1.2e+02	1	GVMNAEDAYMAIGAGADGIVVSNHGGR + Oxidation (M)
6758	719.3312	2873.2957	2872.5545	0.7411	1	11	95	1	QILGDNIYSSGETLLEEVVANILVKR
7132	645.4848	3222.3876	3221.3327	1.0549	1	11	66	1	NSSSINISFDNGRSSDEDGMDEEDEAFVR
8720	1391.2157	5560.8337	5560.8134	0.0203	1	11	15	1	AITDSGESLLTILNDILDYSAIEAGGKNVSVSDEPFEPRPLLESTLQLMSGR
2640	445.1186	888.2226	888.4930	-0.2703	1	10	2.5e+02	1	LGFGRSPR
2769	981.4499	980.4426	979.4644	0.9782	1	10	2.5e+02	1	MEETKAAGK + Oxidation (M)
6085	844.4041	2530.1905	2530.2241	-0.0336	0	10	1.2e+02	1	TTQHEFPAHDGEVWSLPVPVER
6558	553.8365	2764.1461	2763.3246	0.8215	1	10	88	1	TEGDSWDLANSVGATATMVAAARAAATR
7231	837.8617	3347.4177	3348.5860	-1.1683	1	10	59	1	DTQYYFVLPAGEHLDNYKVMVSTEITDGK + Oxidation (M)
8498	1133.7526	4530.9813	4530.0919	0.8894	1	10	28	1	LIMAGNYEEALSVAKMQVEMGAQVLDINMDDGMLDGPSAMTR + Oxidation (M)
3648	690.6679	1379.3212	1379.7231	-0.4019	0	10	1.5e+02	1	MIGYLQGSLAGVR + Oxidation (M)
4038	734.2841	1466.5536	1465.6936	0.8600	0	10	1.9e+02	1	ENTYEGVEAVIDK
5534	1052.0043	2101.9940	2103.0895	-1.0955	1	10	1.6e+02	1	LHAGEVTMPGVKNGQPVDVR
5536	703.3062	2106.8968	2107.0561	-0.1593	1	10	1.5e+02	1	SPHVLIPMGGDFAFKNAYK + Oxidation (M)
6787	965.4100	2893.2082	2892.4513	0.7568	1	10	82	1	SSEMQIGQLAILKAGAAYVPMDPAYPR + Oxidation (M)
7282	855.8922	3419.5397	3418.5282	1.0115	0	10	66	1	HPTTVAVSSPNMEAAQCIQDVFMNHHYFR + 2 Oxidation (M)
3094	564.4179	1126.8212	1125.6393	1.1819	0	10	2e+02	1	QTPNIELLAK
4015	727.8322	1453.6498	1452.6957	0.9542	1	10	2.2e+02	1	QNTEEHSLRDPK
7995	838.4023	4186.9751	4186.1125	0.8626	1	10	41	1	WVIASFSILTISYMITWTSISNAYPIFAQKSYESPR + Oxidation (M)
8336	639.7162	4470.9625	4472.1271	-1.1646	1	10	30	1	VSGLDIARRPSENLLSNMNCSDNGNGGNMLNSFVNSALPPPK + Oxidation (M)
3559	674.5510	1347.0874	1346.6540	0.4334	0	10	1.7e+02	1	SDVYIFMLSTR + Oxidation (M)
5894	617.8320	2467.2989	2468.1312	-0.8323	1	10	1.3e+02	1	DMLDRFMGVDIEQIGGVPSDSR + 2 Oxidation (M)
3440	434.8463	1301.5171	1300.5904	0.9267	1	10	2.4e+02	1	MVEGSCKVYGR + Oxidation (M)
5197	474.2670	1893.0389	1891.9098	1.1291	1	10	1.7e+02	1	EGSGIREEIPSMPGVYR + Oxidation (M)
7523	1215.8522	3644.5348	3643.6934	0.8414	1	10	49	1	MQKMAMLMACLACMGLAAAFSPATLGVNKPSHR + 5 Oxidation (M)
8014	838.9609	4189.7681	4188.9418	0.8263	0	10	34	1	FDDLNGIPMTSLFPQNVSTCSGFGFACTGAVHMVIPSSK
7649	764.3864	3816.8956	3816.7434	0.1522	1	10	54	1	LLCGGEALKGDGFDNGAWVAPTVFTDCSDEMTIVR + Oxidation (M)
7673	764.3892	3816.9096	3818.0328	-1.1232	1	10	54	1	TSIVFMGTPAFAAPILESLIKEQYQVLGVVTQPDR
5800	792.9385	2375.7937	2375.1241	0.6696	1	10	93	1	LASGESVEIDGEEIDPERFQR
8247	736.4951	4412.9269	4413.2270	-0.3000	1	10	31	1	TSAALARMSEEGLPFISILTDPTMGGVSASLAMLGDIHIAEPK + Oxidation (M)
3537	671.0892	1340.1638	1340.6911	-0.5272	0	10	1.6e+02	1	DPIAIGAFHAMAK
4283	769.7372	1537.4598	1536.7784	0.6815	1	10	1.4e+02	1	TTPSIVGFTSKDER
5043	916.8805	1831.7464	1832.9129	-1.1664	1	10	1.7e+02	1	RNNDELVATYGNLAQR
5529	700.3087	2097.9043	2097.0776	0.8267	0	10	1.5e+02	1	YVNSVVVSAAGTNGVITATMK + Oxidation (M)
7721	780.5319	3897.6231	3896.8336	0.7895	1	10	40	1	FAVLSDILGDEDHLGDMDFKVAGTDEGVTSLQMDIK + Oxidation (M)
8081	850.2056	4245.9916	4245.9028	0.0888	1	10	41	1	FSQSAQSMEEASLQADEPKTPTTQGFMVMETEQQTPPK + 2 Oxidation (M)
8670	1211.0826	4840.3013	4840.4026	-0.1013	1	10	26	1	GYNEDPNFISHLPILMDATCNGLQHLSAMVNDFVLAEKVNLLK
6465	670.2740	2677.0669	2676.3471	0.7198	1	10	86	1	TLLITMDMNKIAQMSCNPAVGGIAK
7302	689.9283	3444.6051	3444.7195	-0.1144	0	10	71	1	ITVVGEYGQGESVGELEVMTESTRPATLHAIR + Oxidation (M)
3090	562.3412	1122.6678	1121.5676	1.1002	1	10	2.5e+02	1	LSSSSPSSKSR
4223	758.3961	1514.7776	1514.7147	0.0629	0	10	2.3e+02	1	EMANSHLGIVTDGR + Oxidation (M)
5835	805.7252	2414.1538	2414.1900	-0.0362	0	10	1.4e+02	1	GSGQVVTYGLNTFTNMEAQLIR + Oxidation (M)
6642	566.6929	2828.4281	2828.3850	0.0431	1	10	1.1e+02	1	MKNVLSIQSHVVFGHAGNGASEFPMR + Oxidation (M)
7160	816.3799	3261.4905	3260.7670	0.7235	1	10	77	1	TLGHVIFAVPELVEQLVRFVAAGSEQAGAPR
3000	351.5257	1051.5553	1050.5566	0.9987	1	10	2.4e+02	1	GMKVIAMTGK + Oxidation (M)
4205	755.9611	1509.9076	1508.7179	1.1897	1	10	2e+02	1	RSGSAGTTATSTEAGR
4440	786.6600	1571.3054	1570.7814	0.5241	0	10	1.5e+02	1	QSGAVLYNSFGIMGK
4466	791.3159	1580.6172	1581.6651	-1.0478	1	10	1.8e+02	1	CCLEKLEDEDSGK
4544	807.3463	1612.6780	1613.8559	-1.1778	1	10	2e+02	1	TLMLKPERSASPER
7265	680.8943	3399.4351	3398.5799	0.8552	0	10	57	1	AMIISWVMPLNEDGSNVVTYWIASSDGSDNK
2474	379.2473	756.4800	757.4082	-0.9282	1	10	1.5e+02	1	APGKGGGSK
6424	667.5853	2666.3121	2665.2150	1.0971	0	10	1.2e+02	1	NHGNNEADAALQGLAQQGVDMEDLR
7574	621.4818	3722.8471	3721.9502	0.8969	1	10	61	1	VVSQKQIATNIFELTLHGELVQDMTPGQFVHVK + Oxidation (M)
3205	584.3547	1166.6948	1165.6204	1.0745	1	10	2.4e+02	1	PGVANPGPSKSR
3273	411.2065	1230.5977	1229.6979	0.8998	1	10	2.8e+02	1	AKELASSVAGAVK
4482	795.8633	1589.7120	1589.5868	0.1252	1	10	2.1e+02	1	DSDMGSRSESMGCR + Oxidation (M)
6359	663.5317	2650.0977	2651.2829	-1.1852	1	10	96	1	VMSGVLEGSNVNAVAAMSDMIASARR + Oxidation (M)
6675	952.7646	2855.2720	2854.4573	0.8146	0	10	98	1	VVAALGDSLTTAVGARPNNSSDLPTSWR
5491	1031.1781	2060.3416	2060.1016	0.2400	1	10	1.1e+02	1	LNFDAFLPMLKEVDALPK
2066	602.1901	601.1828	601.3184	-0.1356	0	10	5.7	1	GGNGGIK
3019	531.8154	1061.6162	1060.5149	1.1014	0	10	3e+02	1	AQLSTAADER
5547	1060.0093	2118.0040	2118.0779	-0.0739	1	10	1.6e+02	1	DGQPVRVTLGMLGEEAYGVK
6552	553.8353	2764.1401	2765.2942	-1.1540	0	10	89	1	GGYHFCGGSIYSPEIIVTAAHCLEK
7466	720.4065	3596.9961	3597.8470	-0.8509	0	10	55	1	VCQAMLDVAASQGWLVTTLNITHLIQMVIQGR + 2 Oxidation (M)
8309	1115.7754	4459.0725	4460.2686	-1.1961	1	10	35	1	NSATLNLIYDGLVDASLDRGSFILALGGGVIGDMAGFAAASYLR + Oxidation (M)
4746	562.2783	1683.8131	1684.8468	-1.0337	1	10	2.1e+02	1	RVGSVPHAGFGMGLER + Oxidation (M)
6017	625.8004	2499.1725	2500.1825	-1.0100	1	10	1.3e+02	1	ALEQMKQSFELDTENAVMVSAK + 2 Oxidation (M)
6089	844.4070	2530.1992	2530.2241	-0.0249	0	10	1.3e+02	1	TTQHEFPAHDGEVWSLPVPVER
7367	879.1713	3512.6561	3511.8861	0.7700	1	10	67	1	VVVGLSGGVDSSVAAALLHRQGYAVEGLTLWLMK + Oxidation (M)
8553	919.4035	4591.9811	4592.1316	-0.1505	1	10	27	1	LVAMTGDGTNDAPALAQADIAVAMNSGTQAAKEAGNMVDLDSNPTK + 3 Oxidation (M)
3819	711.9631	1421.9116	1421.6465	0.2651	0	10	2e+02	1	MMAGGDCILVPSR + Oxidation (M)
4434	785.2509	1568.4872	1568.0681	0.4192	0	10	1.5e+02	1	LLLVSVVLLVLLFK
4593	815.7776	1629.5406	1628.7902	0.7504	1	10	1.5e+02	1	TRTAVAMYDMTPLK + 2 Oxidation (M)
7433	898.1368	3588.5181	3587.8341	0.6840	1	10	53	1	GAIVDQLGGDLNSTPLHWATRQGHLSMVVQLMK + Oxidation (M)
2511	391.2826	780.5506	781.4446	-0.8940	0	10	97	1	AGPKPGGAK
3495	658.0448	1314.0750	1314.7085	-0.6334	1	10	1.8e+02	1	RIIGFDPPSWK
3743	707.9724	1413.9302	1413.7769	0.1534	1	10	2.1e+02	1	SVWPDAFPKVLR
4511	798.8178	1595.6210	1594.7773	0.8437	1	10	1.8e+02	1	QEGVLNSKVGMDYR
5056	461.5029	1841.9825	1841.8863	0.0962	1	10	1.9e+02	1	MTSTDILRMAESLNTK + 2 Oxidation (M)
7248	846.4047	3381.5897	3381.7901	-0.2004	1	10	73	1	EMRPMVTEQVIPFLSSHVGDLQIISRVLR + 2 Oxidation (M)
2637	445.1182	888.2218	888.4930	-0.2711	1	10	2.6e+02	1	LGFGRSPR
6371	663.7988	2651.1661	2650.2308	0.9353	0	10	1.1e+02	1	CASTFPPLNQLPFMNASAEQHYK
7707	969.4842	3873.9077	3873.8780	0.0297	1	10	55	1	QVVAFLAAMEIMAEQFPGAFSGYKLEVMESHQATK + Oxidation (M)
7819	804.5374	4017.6506	4017.9306	-0.2800	1	10	37	1	DGVITVEDGQGLQDELAVVEGMQFDRGYLSPYFINK + Oxidation (M)
7950	825.5422	4122.6746	4122.9192	-0.2446	1	10	31	1	DGLVTPEEFMNYYAGVSASIDTDVYFIIMMTTAWKL + 2 Oxidation (M)
3551	672.8315	1343.6484	1342.7972	0.8512	1	10	2.6e+02	1	KFLLSSISAHLK
5854	609.8147	2435.2297	2435.3723	-0.1426	1	10	1.3e+02	1	VAVLGQFPLPVEVVPMARTAIGR + Oxidation (M)
3683	696.1420	1390.2694	1390.6663	-0.3969	0	10	1.7e+02	1	VGNTPLDMNQFR

4020	729.0601	1456.1056	1456.6293	-0.5236	0	10	1.6e+02	1	TYHLGEMSDFNK + Oxidation (M)
4481	795.8629	1589.7112	1589.7468	-0.0355	1	10	2.2e+02	1	MGQATSNIDPQEKR + Oxidation (M)
5076	619.0008	1853.9806	1853.8434	0.1372	1	10	1.9e+02	1	SCIDLSMTCQEKGGIR
2595	852.2651	851.2578	851.4865	-0.2287	0	10	2.1e+02	1	DGLVLAHK
3608	681.8397	1361.6648	1361.6398	0.0251	0	10	2.5e+02	1	HTVMPTDINYR + Oxidation (M)
5633	551.2481	2200.9633	2200.7510	0.2123	1	10	1.5e+02	1	EEKASDMGSGGMGGMGGMNGMM + 5 Oxidation (M)
6099	846.7157	2537.1253	2536.3360	0.7893	1	10	1.2e+02	1	KEPVLVVGGGLITDVVGFACSTYR
6310	872.7293	2615.1661	2615.2901	-0.1240	0	10	1.2e+02	1	ILEMEQYADAVVGVPGEGLNVEQR
8543	764.3208	4579.8811	4579.9851	-0.1040	1	10	25	1	NPCNITSRVYGGHTSPTFDNNGSMMFLDMSYNMLSGYIPK + 4 Oxidation (M)
2473	379.2469	756.4792	757.3970	-0.9178	0	10	1.5e+02	1	TPQIGDK
3423	648.1453	1294.2760	1294.6742	-0.3981	1	10	1.6e+02	1	DQLEIRTHQR
4206	756.0123	1510.0100	1509.6922	0.3178	0	10	1.7e+02	1	VTWDSSFCAVNPK
6256	436.0551	2610.2869	2611.2808	-0.9938	1	10	1.2e+02	1	TRMLQGLIEGMNEQGCLIIFEK + 2 Oxidation (M)
7206	832.4028	3325.5821	3326.6565	-1.0744	1	10	79	1	FSLISLVPSNDDHQTPIMNSVTVRQDLER + Oxidation (M)
8082	850.2056	4245.9916	4245.1371	0.8545	1	10	42	1	AIVIVALDNLTKGSSGQALQNANLMLGEDETAGLMMAPLFP + 2 Oxidation (M)
8132	1074.2629	4293.0225	4293.7930	-0.7705	0	10	39	1	VGMGPGNQMGGMMGGPNQMQGPGAGMVGGMPGQMGVGMGPGGMSGGK + Oxidation (M)
2667	453.5041	904.9936	904.4866	0.5071	0	10	2.4e+02	1	ITATDGSLK
3272	615.6502	1229.2858	1229.4983	-0.2124	0	10	1.9e+02	1	MESAGSDFQSR + Oxidation (M)
3504	659.8978	1317.7810	1318.6816	-0.9006	0	10	2.6e+02	1	VQVHPGVCAKPAG
4032	731.5452	1461.0758	1461.6735	-0.5977	1	10	1.7e+02	1	KESSNSLYESYR
4868	865.6922	1729.3698	1729.8999	-0.5300	1	10	1.5e+02	1	KDLILGDQYNHSSLK
7180	659.8914	3294.4206	3294.6112	-0.1905	1	10	69	1	SPEMGVAQALDALESKLVDFSALTADLNMSR + Oxidation (M)
7393	708.1260	3535.5936	3535.7803	-0.1867	1	10	66	1	QNVEMPICEQIYQILYQGKSAQDAALALLGR + Oxidation (M)
5691	756.5294	2266.5664	2266.1567	0.4097	1	10	1e+02	1	GALRHQDSAGNSGVIYPGLAQR
6087	1266.1046	2530.1946	2531.2140	-1.0193	1	10	1.3e+02	1	VSDDNASTDFVAEPVKLPENQTR
6344	662.0679	2644.2425	2643.2859	0.9566	1	10	1.2e+02	1	GIVIGEGMVMPDKFLAMNTGFVNR + 3 Oxidation (M)
4046	736.1569	1470.2992	1469.7660	0.5332	1	10	1.6e+02	1	LTEPAGVRMLPDR + Oxidation (M)
4381	782.9260	1563.8374	1562.7663	1.0711	1	10	2.2e+02	1	WNPKMSPYISANR
5333	498.0175	1988.0409	1988.9989	-0.9581	0	10	1.8e+02	1	CSFSNPSLPISPNSLITR
6252	653.3307	2609.2937	2610.2354	-0.9417	1	10	1.2e+02	1	IAYVNKMDIMGANFFNVIEMMK + 2 Oxidation (M)
6336	528.8362	2639.1446	2640.1068	-0.9622	0	10	1.1e+02	1	SMVGDLAAGGGGGSGGGGVEEEEDMFPR + Oxidation (M)
7377	884.4122	3533.6197	3533.7972	-0.1775	1	10	69	1	RTAAIMYAGGADTTVSTIQSFVLAMMVYPEVLK
8277	743.6849	4456.0657	4456.9785	-0.9127	0	10	37	1	MDYATPAAAQEISDTEPELSDEEMDLLADLVDASELEAATR + 2 Oxidation (M)
4201	503.5864	1507.7374	1508.8239	-1.0865	1	10	2.3e+02	1	EKLDLQVLNYFK
6498	542.4321	2707.1241	2706.2054	0.9187	0	10	97	1	MDPEGTPGEGVGCTGWFNVEAIVER
7493	726.9211	3629.5691	3629.8388	-0.2696	1	10	57	1	NEYFGHMQFLIEHGVQLNAPAAFQLVRTMLR
8089	850.2065	4245.9961	4247.0919	-1.0958	1	10	43	1	VHEMLPCTILGAKPEPKVVTLGNDYDYVWDISAAAGSSK + Oxidation (M)
3616	682.5645	1363.1144	1363.8551	-0.7407	1	10	1.7e+02	1	LLKVPVGEALIGR
4583	814.3849	1626.7552	1625.8373	0.9180	1	10	2.2e+02	1	STTSSNLSAAFSRLGK
6166	853.0844	2556.2314	2557.2442	-1.0128	1	10	1.3e+02	1	SGNATVLKGGSDAHESNTAIVECIK
6443	667.5856	2666.3133	2665.2150	1.0983	0	10	1.2e+02	1	NHGNNEADAALQGLAQQGVDMEDLR
6797	726.3505	2901.3729	2900.4889	0.8840	1	10	1e+02	1	VGTVGTPVGTITLSCAYRINLAFMSTR + Oxidation (M)
7835	808.5563	4037.7451	4036.8497	0.8954	0	10	41	1	LGAPFSGYEMHIGATEGADTARPFAELDGAGAEGAVSADGR + Oxidation (M)
8087	850.2064	4245.9956	4245.1371	0.8585	1	10	43	1	AIVIVALDNLTKGSSGQALQNANLMLGEDETAGLMMAPLFP + 2 Oxidation (M)
8446	753.6889	4516.0897	4515.2010	0.8888	1	10	34	1	VRAMLDENGKPCNEAGPSIPVEIQGLGDVPAAGESVQVVPDER + Oxidation (M)
8722	1113.1759	5560.8431	5561.6164	-0.7733	1	10	16	1	MTDNDALSLPPDVFAAEDLAAEIDEETSCVVVQTPDAFGNLRDLSPLAAACK
7767	993.4824	3969.9005	3968.7139	1.1866	0	10	52	1	AFEETQIDIPGGGSHGGSSMSLWEVMQSDMIPEDQR + 3 Oxidation (M)
8531	914.4091	4567.0091	4566.2383	0.7708	1	10	30	1	ITPAHDANDFEAGQRLGLKPLLVMNPNGTMNENAGIYQGLDR + Oxidation (M)
2330	661.2811	660.2738	660.3806	-0.1068	0	10	19	1	ATAGTLK
3017	529.8585	1057.7024	1058.5291	-0.8267	1	10	3.1e+02	1	DCVERPRK
3710	702.1039	1402.1932	1402.6365	-0.4432	1	10	1.7e+02	1	VSRFGYSDTDEK
8652	951.8199	4754.0631	4753.3576	0.7055	1	10	25	1	PLGAPALLALALGLGLWLGALAGDPGRGCGPCPLPCFCGPAPDAACR
5254	644.7186	1931.1340	1931.9887	-0.8548	0	10	1.7e+02	1	STILPDMIGHTIAVHDGR
5339	996.1967	1990.3788	1989.9136	0.4653	1	10	1.2e+02	1	SCHSSLPVAEKMSEDVAK + Oxidation (M)
6586	700.0923	2796.3401	2797.2106	-0.8705	0	10	1.2e+02	1	GMEGLISPTEAMGNSNGASSSCPGWLR + 2 Oxidation (M)
7954	825.7762	4123.8446	4124.0922	-0.2476	1	10	40	1	MKYLLPSTAAGLLLLAAQPTMAANTGGYATTDGGNVAGAVNK + 2 Oxidation (M)
2981	520.7672	1039.5198	1040.6091	-1.0892	1	10	2.6e+02	1	AIDQRGLLR
3080	560.0015	1117.9884	1117.5954	0.3931	1	10	2.1e+02	1	DPKMLPVYR
3378	639.2427	1276.4708	1275.6493	0.8216	1	10	2.4e+02	1	MQAAIAKVDDSK
3646	690.5300	1379.0454	1378.7932	0.2522	1	10	1.7e+02	1	NPSLPKDIILNR
7715	972.6597	3886.6097	3886.0548	0.5549	1	10	43	1	DAGANARPVANGGSGSSPGKPVLEQSPIKSLSNLVSSPIK
8357	898.1946	4485.9366	4486.1937	-0.2571	1	10	30	1	MTVVTITPGAMSFADWRAIYEGASAALTVGAWDAIDASAAAVAR + 2 Oxidation (M)
3542	671.6450	1341.2754	1341.6235	-0.3480	0	10	1.7e+02	1	TTMIAGYPSETR + Oxidation (M)
6440	667.5855	2666.3129	2665.2150	1.0979	0	10	1.2e+02	1	NHGNNEADAALQGLAQQGVDMEDLR
7728	788.1926	3935.9266	3937.0402	-1.1136	1	10	55	1	VDQEMLYEIILAANYLNIKPLLDAGCKVVAEMIR + 2 Oxidation (M)
8326	639.7142	4470.9485	4471.1026	-0.1542	1	10	32	1	NLFNPENIFISKEGGGAGNNWASGYTQGEAVQETLLDMIDR + Oxidation (M)
4825	855.7015	1709.3884	1708.9723	0.4161	1	10	1.4e+02	1	TAKGNVLEPQGLIEIK
5294	490.4698	1957.8501	1957.0666	0.7835	1	10	1.7e+02	1	MLTGDIIPSSGNILIRNK + Oxidation (M)
6583	931.0877	2790.2413	2789.2427	0.9985	1	10	1.1e+02	1	MKAGATSMWASCCGLLNEVMGTGAVR + 2 Oxidation (M)
7127	1066.8101	3197.4085	3197.4566	-0.0481	1	10	80	1	VEIYPSLMEDDKEMLEDLIAAACNDAAR + Oxidation (M)
4540	806.2552	1610.4958	1610.8628	-0.3669	1	10	1.5e+02	1	ELTAAKVGDTVTHAAK
8395	1129.5280	4514.0829	4514.1644	-0.0815	1	10	34	1	SGQMMLAQGLLLHLMPTDWRWSDCHALTDVDFEVLKPR + 3 Oxidation (M)
3424	648.2098	1294.4050	1294.7357	-0.3307	1	10	1.8e+02	1	RSLDAPIPSALR
4224	758.4362	1514.8578	1514.7882	0.0697	1	10	2.4e+02	1	INSIDTQFRFFK
5597	544.2466	2172.9573	2172.1460	0.8113	1	10	1.5e+02	1	AIESGKPANIVEKMVSGSVEK
7793	999.7138	3994.8261	3994.8176	0.0085	1	10	48	1	KPMASVPSCHEVQGFMPGRLEFETEFENEAEGPVK + 2 Oxidation (M)
8040	846.7836	4228.8816	4227.9940	0.8876	1	10	40	1	SAYDALPSSTIVSMASCASGSTKGYDELVPHQISVVSEER
6919	600.1077	2995.5021	2994.4211	1.0810	1	10	1e+02	1	YMTQGLDMSGVFMEMVKASATVDIVQK + Oxidation (M)
7430	1194.6235	3580.8487	3580.9478	-0.0991	1	10	71	1	VIPHHPQGNGGTPGITLVNASIAPLSTPVTPASPKR
5439	1021.1542	2040.2938	2041.0844	-0.7905	0	10	1.3e+02	1	QIIDANPILEAFGNAQTVK
6729	574.6901	2868.4141	2868.3820	0.0321	1	10	1.1e+02	1	LVGQAIETFNMIEDGDRLMVCLSGGK + Oxidation (M)
6816	730.2904	2917.1325	2916.4296	0.7029	1	10	79	1	GADPNLCCVPMQVLFLAVKAGDVDGVR + Oxidation (M)
6920	500.2578	2995.5031	2995.4920	0.0111	1	10	1e+02	1	QVAMHIAATNPLALTAEDVDSSAIEREK + Oxidation (M)
8185	872.8045	4358.9861	4358.3744	0.6117	1	10	38	1	RPVLLTGAIGMAVSQLLVAVLGTTTTGQDSRGNIIVHDAAAQK + Oxidation (M)
3492	657.5247	1313.0348	1313.7303	-0.6955	0	10	1.9e+02	1	QTQSAALDILVR
3666	693.0283	1384.0420	1383.7511	0.2910	0	10	1.8e+02	1	LGNPTWVQVATAK
4474	794.3215	1586.6284	1585.7624	0.8660	0	10	2.1e+02	1	GDTVTVEFDTFLSR
4668	821.9026	1641.7906	1640.8266	0.9640	1	10	2.3e+02	1	MIKVVVDDSAFMR + 2 Oxidation (M)
5648	738.3665	2212.0777	2211.1171	0.9605	1	10	1.6e+02	1	QFISSLEDAETIKSFNNLR
7257	564.9391	3383.5909	3382.6935	0.8975	0	10	76	1	QAMQSIDVPGMSVNIIHSGVGLISENDIMLAK + Oxidation (M)
8165	866.2067	4325.9971	4325.9457	0.0514	0	10	40	1	SVDQNGLGAAWDEVQQGGPMPPMGPMFEPVQPTFEGPPQR + 3 Oxidation (M)
8323	895.1957	4470.9421	4470.0243	0.9179	1	10	32	1	MLPEFMGGSADLAPSNLTMWSGSKSLEASDFSGNYIHYGVR + 3 Oxidation (M)
8333	746.1661	4470.9529	4471.3939	-0.4409	1	10	33	1	MLPYEMVIAVLVYLSPAQILNLNLPFAYQKSVLFASNSAK + Oxidation (M)
8535	1143.0067	4567.9977	4567.2464	0.7513	0	10	30	1	YWGAPIPMMTLEDGTVVPTPEDQLPVVLPEDVVMDGISSPLK + 2 Oxidation (M)
6674	952.7645	2855.2717	2856.4109	-1.1393	0	10	1e+02	1	AISLPMDLDAHISSLLSSGASCVAAAQR + Oxidation (M)
6811	972.4645	2914.3717	2914.5261	-0.1544	1	10	1.1e+02	1	SRGPAASLLVQNGTLNVGDSIVVGNTYGR
7221	667.5604	3332.7656	3332.6301	0.1355	1	10	79	1	MAEASLTIVSHEEALMKAENSLSALQGEMVK + Oxidation (M)
7419	714.3237	3566.5821	3566.7021	-0.1200	0	10	65	1	GPDLQLGYVENGTMLLGSPNVVNIGDQNFMNTK + 2 Oxidation (M)
2466	376.9834	751.9522	751.4956	0.4567	1	10	37	1	GPKLLPK
4901	875.3467	1748.6788	1747.8121	0.8668	0	10	1.8e+02	1	MQIVTDPAEMQATADK
5362	1006.9716	2011.9286	2011.0408	0.8878	1	10	1.8e+02	1	IKVEPNSLGALMGETAHTK + Oxidation (M)

☒	6117	636.5456	2542.1533	2541.1780	0.9753	0	10	1.3e+02	1	MLVYEYVNSGNLEQWLHGAMR + 2 Oxidation (M)
☒	8205	730.6802	4378.0375	4379.2359	-1.1984	1	10	38	1	VSVFVEVEGAGDFLPPYAGNLDIMTAAATKVGEVIAGQITSAR + Oxidation (M)
☒	4550	808.3613	1614.7080	1615.8781	-1.1700	0	10	2.2e+02	1	LAADSILAGIESSLTR
☒	5016	604.6735	1810.9987	1810.0676	0.9311	1	10	2e+02	1	NIALVNVSLQVKGNTIK
☒	5603	728.6766	2183.0080	2183.1222	-0.1143	0	10	1.6e+02	1	ATSSTFSSLLPSPPALLSDHR
☒	7289	685.3214	3421.5706	3421.5190	0.0516	1	10	74	1	NEEIVCNFCNTKYEFTNDEIGELLNNSR
☒	7717	778.7277	3888.6021	3887.7492	0.8529	1	10	43	1	MSAEEYIEVSSSPDIFTDDDDMITIEPELNKNPK + Oxidation (M)
☒	2576	840.4041	839.3968	838.3821	1.0147	0	10	2.1e+02	1	DGDLTYR
☒	3339	626.2698	1250.5250	1250.5853	-0.0602	0	10	2.6e+02	1	ALEEFMPDGVK + Oxidation (M)
☒	3490	657.1683	1312.3220	1312.6809	-0.3588	0	10	1.8e+02	1	LQHSLAEAGMLK + Oxidation (M)
☒	6647	947.4620	2839.3642	2840.3862	-1.0220	0	10	1.2e+02	1	VSSGAEMVLYSVNAITGGTEAQGEVTVR + Oxidation (M)
☒	8025	841.7738	4203.8326	4203.9010	-0.0683	1	10	39	1	TTDGAQTSRWAAMQIGMSFISAYAMCAGEAAVADLSFAAK + 3 Oxidation (M)
☒	8047	1059.7599	4235.0105	4234.0425	0.9680	1	10	44	1	DGNSFELEADGLLAICIQHEMDHLVGKLFIDYLSPMK + Oxidation (M)
☒	4824	854.0323	1706.0500	1704.8835	1.1665	1	10	1.9e+02	1	TLYDKIFDAHVVER
☒	5558	711.3550	2131.0432	2130.9635	0.0797	0	10	1.7e+02	1	AMSAMQLGDMELAQSYLEK + Oxidation (M)
☒	6538	684.3004	2733.1725	2732.1377	1.0348	1	10	1e+02	1	DDLQEMIDNDHGAEAVCRFCGHK + Oxidation (M)
☒	8257	1104.4901	4413.9313	4413.9817	-0.0504	1	10	35	1	TALQNAASVASLLLTTEAMVAEAPKDESAAPAMPGGMGGMGDMGM + 4 Oxidation (M)
☒	8537	914.6100	4568.0136	4569.1428	-1.1292	0	10	31	1	LVMQLLPFLASTAAAAAAIDSTSSSNGSDHHGSSFQAECESFK + Oxidation (M)
☒	8668	968.0027	4834.9771	4835.1527	-0.1756	0	10	22	1	FGPTMMVGDGMNDAAALAAADVGVSMGISGSAAAMETSHATLMSSDVLR + 4 Oxidation (M)
☒	2476	758.0644	757.0571	756.3766	0.6805	0	10	1.2e+02	1	NNVEGPK
☒	4012	485.2053	1452.5941	1451.6755	0.9185	0	10	2.3e+02	1	FVVDVCGWEVDK
☒	6557	553.8362	2764.1446	2763.3656	0.7790	1	10	97	1	EDIDYVNLYKPQFAGFVFAESRR
☒	6976	751.1029	3000.3825	3001.5253	-1.1428	0	10	98	1	SSLGTNILSVMAAFAGTAILLMDFGVTNR + 2 Oxidation (M)
☒	7376	884.4089	3533.6065	3533.7972	-0.1907	1	10	71	1	RTAAIMYAGGADTTVSTIQSFVLAMMVYPEVLK
☒	7380	707.9273	3534.6001	3535.7691	-1.1690	1	10	71	1	ELTVANPAVLGTNMGPVNDQAAFDKVMSYVAIGK + Oxidation (M)
☒	5169	941.4711	1880.9276	1880.9818	-0.0542	1	10	2e+02	1	QLGAMALFGEKYGEIVR
☒	6217	866.4410	2596.3012	2596.3860	-0.0849	1	10	1.3e+02	1	DSLEEVIEPYLLQQGLIQRTPR
☒	6341	882.3829	2644.1269	2645.2717	-1.1448	1	10	1.1e+02	1	GLYCTKDIEAGEMVIEYAGELIR + Oxidation (M)
☒	6509	542.6334	2708.1306	2709.2810	-1.1503	1	10	1e+02	1	LSTDPFESHFNLPTEDYLAKEEK
☒	7825	670.7812	4018.6435	4019.1958	-0.5523	1	10	40	1	ADMVIAPHVALFPAVAIFLTVLAFNLLGDGLRDALDPK + Oxidation (M)
☒	7951	1031.6782	4122.6837	4122.9192	-0.2356	1	10	34	1	DGLVTPEEFMNYYAGVSASIDTDVYFIIMMTTAWKL + 2 Oxidation (M)
☒	2994	525.7536	1049.4926	1049.4634	0.0293	0	10	3e+02	1	MAMGNPELR + 2 Oxidation (M)
☒	3738	707.0621	1412.1096	1411.7307	0.3790	1	10	1.8e+02	1	ASLPELPDEKSAR
☒	5343	501.0443	2000.1481	1999.9613	0.1868	1	10	1.7e+02	1	HSHVRNGQPTPPPYGDNK
☒	6235	653.3293	2609.2881	2610.3006	-1.0125	1	10	1.3e+02	1	AGHRPIALVGGATGMIGDPSMKSAER + 2 Oxidation (M)
☒	6824	584.8727	2919.3271	2918.5713	0.7558	1	10	1e+02	1	VKAHDEVETAGVGDLVLIAETRPLSATK
☒	6863	736.8486	2943.3653	2943.5709	-0.2056	1	10	1.1e+02	1	MSRLYAIILIALVFNVIMTIMPDMK + 3 Oxidation (M)
☒	8607	934.4260	4667.0936	4667.4395	-0.3459	1	10	29	1	NVFPVPSAGIRPGIVPQVLGDYGRNVILNAGTGIMDHPSGPASGVR + Oxidation (M)
☒	2510	781.3902	780.3829	779.3483	1.0346	0	10	1.1e+02	1	ATADQMK + Oxidation (M)
☒	4953	892.4276	1782.8406	1783.8424	-1.0017	0	10	2.1e+02	1	GMRPSFSGGAHPVEAER
☒	6848	735.6270	2938.4789	2939.4798	-1.0009	1	10	1.1e+02	1	ALEAGMRIDAEVVDTVPLGVDTPADLEK + Oxidation (M)
☒	6891	745.0947	2976.3497	2977.4215	-1.0718	1	10	97	1	FGVEVRGCMSALGEIDVPFVDWSHVR + Oxidation (M)
☒	8068	1062.2568	4244.9981	4245.1371	-0.1390	1	10	46	1	AIVIVALDNLTKGSSGQALQNANLMLGEDETAGLMMAPLFP + 2 Oxidation (M)
☒	8752	1148.7469	5738.6981	5739.6926	-0.9945	1	10	16	1	IIKDGLWENNGVLCMLLGMCPTMAMTGTATNGLGMGLATAAVMAASNLMVAMFR + 4 Oxidation (M)
☒	6186	645.2664	2577.0365	2576.2826	0.7539	1	10	1e+02	1	LIENETAADLLIEDGRCIGVMTK + Oxidation (M)
☒	7805	1001.4210	4001.6549	4001.9867	-0.3318	1	10	40	1	SVAMSPDFLAGGEHYALEVRGDSMIEAGILDGDLVVIR
☒	8487	648.0035	4528.9736	4527.9874	0.9862	1	10	32	1	VASTMTDGANTMIEHDDTLPSQLGTMVINTEDEEEEGTMKR + 2 Oxidation (M)
☒	2449	737.5181	736.5108	736.3504	0.1604	0	10	47	1	ADAGFTR
☒	3500	658.4453	1314.8760	1314.7395	0.1366	0	10	2.5e+02	1	IIGDNLTVETLK
☒	4187	752.5621	1503.1096	1502.7188	0.3909	0	10	1.7e+02	1	VSGAGAPGPQPMGYAK + Oxidation (M)
☒	6226	653.0839	2608.3065	2609.1949	-0.8884	1	10	1.3e+02	1	LMENAGKMDATQEVNTNDENILK + 2 Oxidation (M)
☒	6948	600.1100	2995.5136	2995.5835	-0.0698	1	10	1.1e+02	1	SMTTLALLIFAVGSAISGAARDTGMLLAGR + 2 Oxidation (M)
☒	7392	707.9286	3534.6066	3534.8895	-0.2829	1	10	72	1	VELQALTINDFELILTEPKASVTMQYQALLK + Oxidation (M)
☒	7677	764.5842	3817.8846	3818.9553	-1.0707	1	10	61	1	FSIMENKEVVDQYLAPNISQFLSLLHDGDLIVR + Oxidation (M)
☒	8503	758.3342	4543.9615	4544.1880	-0.2264	1	10	31	1	AEDVAAAAAVTVAPPPAPAGAKSFTMAEVETHTTMESAWFVVDGK + Oxidation (M)
☒	7042	611.0933	3050.4301	3050.4253	0.0049	1	10	1e+02	1	GLEAVKEADLVYAEFYTSCLMGTNPEK + Oxidation (M)
☒	8545	917.1841	4580.8841	4581.4316	-0.5475	1	10	27	1	LSFIQFLVRLVAICSFLVYCASWIFTLLDMALITIMGPK + 2 Oxidation (M)
☒	3727	705.2067	1408.3988	1408.8289	-0.4301	1	10	1.7e+02	1	EVADQVKAPLVLK
☒	4930	884.4117	1766.8088	1767.9302	-1.1213	1	10	2.1e+02	1	KVETGGLHPVTMTINR + Oxidation (M)
☒	6760	719.3317	2873.2977	2872.3194	0.9783	0	10	1.1e+02	1	GYQATHGIDPMILIDALASCMGHEEK + Oxidation (M)
☒	5943	830.7000	2489.0782	2490.1553	-1.0771	0	10	1.3e+02	1	GLGMNPTLEEMLTACQGIGGPGQK + 2 Oxidation (M)
☒	6433	889.7780	2666.3122	2665.3381	0.9741	0	10	1.3e+02	1	VAAGALEVVDALANQAAEHSSTIMPGK + Oxidation (M)
☒	6879	592.6850	2958.3886	2957.3362	1.0524	1	10	1.1e+02	1	GGSTLRDFSNAQGEAGHFQLDAMVYDR + Oxidation (M)
☒	7402	884.9090	3535.6069	3536.8045	-1.1977	1	10	72	1	APVTEMPPAGSFLNQQQQLEVLTQQQKVNAAR + Oxidation (M)
☒	4228	759.0159	1516.0172	1516.7998	-0.7825	1	10	1.9e+02	1	VPQDQNFTTIARK
☒	6703	574.4890	2867.4086	2866.4515	0.9571	0	10	1.2e+02	1	ITASRPLAFYAYGVGVGGEQLGGSHFGR
☒	3642	689.3453	1376.6760	1375.7646	0.9115	0	10	2.7e+02	1	MISFQIILQQR
☒	3820	712.6973	1423.3800	1422.8922	0.4879	1	10	1.8e+02	1	ASLTPLLLAIQKR
☒	4221	758.3347	1514.6548	1514.7001	-0.0453	0	10	2.5e+02	1	GVVSNDDLYGHDPK
☒	4278	768.5164	1535.0182	1535.7943	-0.7761	1	10	1.8e+02	1	SRQILDAAGISYDK
☒	5838	806.6691	2416.9855	2418.1716	-1.1862	0	10	1.2e+02	1	ENGGQYTHAATWVVLAFAAQER
☒	6109	509.2374	2541.1506	2541.2433	-0.0927	1	10	1.3e+02	1	TKLIIMMMCLIMSFIFMQMK + 2 Oxidation (M)
☒	6959	600.3088	2996.5076	2996.4258	0.0819	1	10	1.1e+02	1	LVCSGENDNHGQIANLPSAVTSDQKSQK
☒	7245	676.0803	3375.3651	3376.4390	-1.0738	1	10	62	1	GEELAEMGDMKEAGAVAFSDDGMPVMNAGLMR + 3 Oxidation (M)
☒	3815	474.2506	1419.7300	1419.7214	0.0086	1	10	2.6e+02	1	GISMMKEAGIEVR
☒	5211	636.2953	1905.8641	1906.9472	-1.0831	1	10	2e+02	1	WDPAHRLGCTLLDPTR
☒	5342	667.5863	1999.7371	1999.0965	0.6406	1	10	1.4e+02	1	IKIAFQYVMPQLYLTR + Oxidation (M)
☒	5530	525.4886	2097.9253	2097.1252	0.8001	1	10	1.7e+02	1	MDKGVGSLTTVIVQNGTLHK
☒	6453	667.5875	2666.3209	2667.3616	-1.0407	1	10	1.3e+02	1	LQAQLNELQAQLSQKEQAAEHYK
☒	6479	537.6685	2683.3061	2684.2422	-0.9360	1	10	1.3e+02	1	DQMDRFGEIDIMVNNIAETISNK + 2 Oxidation (M)
☒	6612	939.4254	2815.2544	2816.3217	-1.0673	1	10	1.1e+02	1	MAMPDLTHDLTGKLLIAMPGMEDPR + 4 Oxidation (M)
☒	7299	574.9397	3443.5945	3442.6968	0.8977	1	10	81	1	NLMTVSNLGVIFGPTLMRSQEETVAAMMNIK + 3 Oxidation (M)
☒	8006	699.1688	4188.9691	4189.1479	-0.1788	1	10	49	1	ILGLKPTVIFADQIMYTGFAYAARSGASVGIDDMVIPEK + 2 Oxidation (M)
☒	7088	785.6048	3138.3901	3138.4492	-0.0591	1	10	89	1	NNEFLEMSSNLLDFSKDDQFLNDFIK + Oxidation (M)
☒	7103	1056.1307	3165.3703	3164.4907	0.8796	0	10	83	1	WRPDGAVAATEMGAEVIIMDDGFQNGSIAK + Oxidation (M)
☒	4475	794.6160	1587.2174	1586.7399	0.4775	1	10	1.7e+02	1	RDQLFDVDFSMSK
☒	5190	945.4579	1888.9012	1888.1298	0.7714	1	10	2.1e+02	1	LTVAWDKHKPLLIGVAK
☒	8171	871.7848	4353.8876	4353.1151	0.7725	1	10	35	1	NGNAEAVDDGASLHEMALRCILLEKPDSAAITAQCVAAISR + Oxidation (M)
☒	5143	936.3904	1870.7662	1871.9200	-1.1537	1	10	1.8e+02	1	SVRPFRSSEAYVEAMK + Oxidation (M)
☒	7505	910.4728	3637.8621	3637.8219	0.0402	1	10	70	1	TLGCYPLTGAVESTANTLPEVIQEMLLTKTSER + Oxidation (M)

☒	8484	648.0028	4528.9687	4527.9874	0.9813	1	10	33	1	VASTMTDGANTMIEHDDTLPSQLGTMVINTEDEEEEGTMKR + 2 Oxidation (M)
☒	4034	488.9285	1463.7637	1463.8136	-0.0500	1	10	2.7e+02	1	DRFLFVQEAIVK
☒	4535	803.3464	1604.6782	1605.8376	-1.1593	1	10	2.3e+02	1	HHGWIDSTIKSGLR
☒	6467	537.2659	2681.2931	2681.2166	0.0765	0	10	1.3e+02	1	LMNGYDDIDLTLQHEDDIAAYEK
☒	3155	384.5372	1150.5898	1149.5924	0.9973	1	10	2.9e+02	1	MVRATGSVASR + Oxidation (M)
☒	7273	852.1151	3404.4313	3404.4950	-0.0637	1	10	66	1	TNEFQDSSESEPELFISGDELMNQSQGSRK + Oxidation (M)
☒	6923	749.8832	2995.5037	2994.3773	1.1264	0	10	1.1e+02	1	GSSGQALQNANLMLGEDETAGLMMAPLFP + 2 Oxidation (M)
☒	2600	856.3711	855.3638	856.4879	-1.1241	1	10	2.9e+02	1	DGRVAAIR
☒	2776	986.3099	985.3026	984.5604	0.7422	0	10	2.7e+02	1	GDVLLAEIR
☒	3745	708.5020	1414.9894	1414.7820	0.2074	0	10	2e+02	1	LLGDSVPFEAVLR
☒	4207	756.0950	1510.1754	1510.6933	-0.5179	1	10	1.6e+02	1	MSLSDSPSKSGNTGK + Oxidation (M)

☒	4606	818.3569	1634.6992	1635.7563	-1.0570	0	10	2.3e+02	1	TIFSLHEATDEMAR + Oxidation (M)
☒	5276	973.9200	1945.8254	1945.9390	-0.1135	0	10	1.9e+02	1	NVPHVLAEMTMEGVYTR
☒	5351	1001.9822	2001.9498	2001.0895	0.8604	1	10	2e+02	1	RLNPTAIILSGGPSSVYEK
☒	5516	694.6004	2080.7794	2079.9275	0.8519	1	10	1.4e+02	1	ATQADLMELDMAMEPDRK + Oxidation (M)
☒	5857	1218.6233	2435.2320	2435.3723	-0.1402	1	10	1.5e+02	1	VAVLGQFPLPVEVVPMARTAIGR + Oxidation (M)
☒	6386	889.3614	2665.0624	2664.2973	0.7651	1	10	1e+02	1	CGIRPEMQQGQQQPLPQLGGDRR + Oxidation (M)
☒	3836	714.8250	1427.6354	1426.6948	0.9406	1	10	2.7e+02	1	MIGGMIKEEFTR + Oxidation (M)
☒	4233	759.8384	1517.6622	1517.7627	-0.1004	1	10	2.6e+02	1	DFLANFAGPRGEPK
☒	5747	769.7460	2306.2162	2305.3153	0.9008	0	10	1.6e+02	1	LLILTMVFAVICAGSTLALGVK + Oxidation (M)
☒	8344	639.8580	4471.9551	4473.0533	-1.0983	0	10	34	1	SPALMNFMMTGSWMTIGATFAAMIGAGMLVQSISYEQSPGPK + 4 Oxidation (M)
☒	8547	917.1849	4580.8881	4581.4316	-0.5435	1	10	28	1	LSFIQFLVRLVAICSFLVYCASWIFTLLDMALITIMGPK + 2 Oxidation (M)
☒	3568	676.7616	1351.5086	1352.6684	-1.1598	1	10	2.3e+02	1	DAEGRLVPDPER
☒	5868	611.2773	2441.0801	2440.1699	0.9102	0	10	1.4e+02	1	IAEQEFLEWAEFAGNLYGTPR
☒	5994	625.5336	2498.1053	2499.2502	-1.1449	0	10	1.4e+02	1	TVQELATDMIPFEQMGLQHIAK
☒	7998	699.1317	4188.7465	4188.0272	0.7194	0	10	39	1	TEVMFGPAGVAYVYLIYGMHHCFNVVTDATGAGAAVLVR + 2 Oxidation (M)
☒	3995	724.8329	1447.6512	1448.6826	-1.0313	0	10	2.7e+02	1	MMHIVMIGVNEK + 3 Oxidation (M)
☒	5438	681.1021	2040.2845	2040.9874	-0.7029	1	10	1.4e+02	1	IACFGSGSCPQFLAAATKR
☒	7225	835.6835	3338.7049	3338.8570	-0.1521	1	10	87	1	DILLLMVGLLLGAGGVFVMLRNQSNQALVER
☒	7746	1317.6866	3950.0380	3949.9924	0.0455	0	10	56	1	IVGIVNDLFGAGFDTVSTALSWSVMYLVAHPEIQER + Oxidation (M)
☒	3530	666.5934	1331.1722	1330.5091	0.6632	0	10	2e+02	1	DMEDMTVAEMK + 2 Oxidation (M)
☒	3647	690.6042	1379.1938	1379.6602	-0.4664	1	10	1.8e+02	1	TVEMKTEGSASPK + Oxidation (M)
☒	5309	985.2351	1968.4556	1969.0012	-0.5456	1	10	1.3e+02	1	MTQLEYALSGIVTKEMR
☒	6255	653.5789	2610.2865	2609.2643	1.0222	1	10	1.4e+02	1	SMPERSDIAGDINESLIVELYSR + Oxidation (M)
☒	6548	691.3029	2761.1825	2761.1079	0.0746	1	10	1.1e+02	1	IIHECEDGGAREEMAIDNEAGGDEE + Oxidation (M)
☒	6604	937.8036	2810.3890	2811.3776	-0.9886	0	10	1.3e+02	1	EDVIDFIAGMSVLELSELIGEFEEK
☒	2417	360.9575	719.9004	719.4078	0.4926	1	10	38	1	AGAKFAR
☒	3079	559.9987	1117.9828	1117.4532	0.5296	0	10	2.3e+02	1	MEMNSPHEK + Oxidation (M)
☒	3974	721.3477	1440.6808	1439.8208	0.8600	1	10	2.6e+02	1	SQVLAQLEQLRR
☒	5062	922.9304	1843.8462	1842.9992	0.8470	1	10	2.2e+02	1	GYIDTFYRISLIGGIR
☒	5138	935.8841	1869.7536	1870.0411	-0.2875	1	10	1.8e+02	1	ITQQLSLLDEKDNLIK
☒	5799	792.2133	2373.6181	2374.0576	-0.4395	0	10	1.1e+02	1	FFEQEVYVPAGWSGTMPNGDK + Oxidation (M)
☒	7745	790.1336	3945.6316	3946.8207	-1.1891	1	10	44	1	GGELTIEWAGRSDAPVWMTGPAESVYVGEIDLPEQGD + Oxidation (M)
☒	8101	850.4080	4247.0036	4247.2321	-0.2284	1	10	47	1	ELLDVLSVLSLMERNSPVVISPLLMNLTVENDFSTTVK + 2 Oxidation (M)
☒	8435	1130.0278	4516.0821	4515.1369	0.9452	1	10	37	1	DWALGTPEGDWVSMAVPSQGEYGIPEGIVYSFPVTAKDGAYR
☒	4932	884.9175	1767.8204	1767.9843	-0.1639	0	10	2.3e+02	1	GSLTVNGLSVGLSIAQPR
☒	5671	1123.5217	2245.0288	2244.3609	0.6679	0	10	1.7e+02	1	LIPLPSAPFPQIITALVSVLR
☒	7239	673.2805	3361.3661	3361.6145	-0.2484	1	10	63	1	DEMGLDVLMFIDNIFRFAQSGSEMSALLGR
☒	7556	922.9083	3687.6041	3688.8036	-1.1995	1	10	59	1	TNDNAGDGTTTATVLAQSIINVGMKNVAAGANPMDLK + Oxidation (M)
☒	6054	842.3707	2524.0903	2525.2697	-1.1794	1	10	1.3e+02	1	THSVPNIGFVSLGCPKNLVDSER
☒	6213	866.0541	2595.1405	2595.3036	-0.1632	1	10	1.3e+02	1	FATMEVSVSDGINEAKAIMQLSVR
☒	7101	792.3488	3165.3661	3164.5891	0.7770	1	10	85	1	WGQGGATVHATLWAVGLSDPADTITELRDK
☒	5079	619.6072	1855.7998	1855.9527	-0.1529	0	10	2e+02	1	VAAEIEDIIGIDASDAVR
☒	5755	1167.5408	2333.0670	2332.1079	0.9591	0	10	1.6e+02	1	GEPPTIPGMELTDNLMAFVER + Oxidation (M)
☒	5919	825.7534	2474.2384	2475.2792	-1.0408	1	10	1.5e+02	1	NVIDAGGDVLAISQFTLLADCRK
☒	6014	625.7823	2499.1001	2497.9073	1.1928	1	10	1.4e+02	1	DACGCCPMCARGEGEPCGGGGAGR
☒	6141	639.7887	2555.1257	2556.1097	-0.9840	0	10	1.3e+02	1	GFPAGICPGLGAGGHFSGGGYGNMMR + 2 Oxidation (M)
☒	4025	729.5891	1457.1636	1456.7310	0.4326	1	10	1.9e+02	1	VDGDGPISEVWKR
☒	4135	743.4991	1484.9836	1485.5864	-0.6028	0	10	2.2e+02	1	DPETGEMNAGMYR + Oxidation (M)
☒	4732	841.7758	1681.5370	1680.7525	0.7845	1	10	1.6e+02	1	ENLMNEFRQDQNK + Oxidation (M)
☒	6813	584.2343	2916.1351	2917.2818	-1.1467	0	10	87	1	TTSQSPDAQASCGHPQTDSVAGPAMATTK + Oxidation (M)
☒	8092	1062.7543	4246.9881	4247.2321	-0.2440	1	10	47	1	ELLDVLSVLSLMERNSPVVISPLLMNLTVENDFSTTVK + 2 Oxidation (M)
☒	3445	651.7673	1301.5200	1302.6238	-1.1037	0	10	2.8e+02	1	EAMANQQAVPTK + Oxidation (M)
☒	3681	695.1108	1388.2070	1388.7446	-0.5375	1	10	1.9e+02	1	TMELLRGVGLER + Oxidation (M)
☒	4447	788.0542	1574.0938	1573.8689	0.2250	1	10	1.8e+02	1	DGIHVGVPARVLDAR
☒	8016	839.0012	4189.9696	4191.0989	-1.1292	1	10	50	1	AIRLIQACVDVLSSNGWLSPALAAMELAQMVTQAMWSK + 2 Oxidation (M)
☒	2553	403.4362	804.8578	805.4116	-0.5538	1	10	1.8e+02	1	DGLKAMR + Oxidation (M)
☒	3565	676.3836	1350.7526	1350.5795	0.1731	0	10	2.8e+02	1	ATAMTASSYAMSK + 2 Oxidation (M)
☒	4050	736.6925	1471.3704	1470.7467	0.6237	0	10	1.8e+02	1	ALVGFVTAGDPDPGR
☒	3433	650.1437	1298.2728	1298.6942	-0.4214	1	10	1.9e+02	1	KDIEATDRPVR
☒	4080	739.3002	1476.5858	1476.6588	-0.0730	0	10	2.5e+02	1	SNNMSPPEMLVDK + Oxidation (M)
☒	4896	582.9547	1745.8423	1746.9853	-1.1430	1	10	2.4e+02	1	VGPGSLAITGGSRLLGHR
☒	6118	510.0391	2545.1591	2546.2812	-1.1221	1	10	1.4e+02	1	SKPREMRPPPHSLFPIGDEGGAR + Oxidation (M)
☒	7399	884.9088	3535.6061	3536.6843	-1.0782	1	10	76	1	EMNFITENRASVVYELPLAEMVGDFFDQLK + 2 Oxidation (M)
☒	8433	1130.0276	4516.0813	4515.1369	0.9444	1	10	38	1	DWALGTPEGDWVSMAVPSQGEYGIPEGIVYSFPVTAKDGAYR
☒	4371	779.8785	1557.7424	1557.7756	-0.0331	0	10	2.6e+02	1	IQAICVVCGNPATR
☒	4574	812.8305	1623.6464	1623.7926	-0.1462	1	10	2.2e+02	1	YKDIGDLGEIAMGAR + Oxidation (M)
☒	5338	664.3079	1989.9019	1989.8713	0.0306	1	10	2e+02	1	RLQEGYECADWYICK
☒	6861	588.9048	2939.4876	2938.4092	1.0784	0	10	1.2e+02	1	LADDMFETMYDAPGIGLAAIQIAEPVR + 2 Oxidation (M)
☒	7167	820.8585	3279.4049	3278.7333	0.6716	1	10	80	1	TIGTITSQVNYRAALNTLWASLMLEELVR + Oxidation (M)
☒	7999	838.7586	4188.7566	4187.8073	0.9494	0	10	40	1	NDLEQIDQILEHMQSTVYENSGSSPGAHYGSGSGYDMR + Oxidation (M)
☒	8075	708.6722	4245.9895	4247.0126	-1.0230	1	10	48	1	VAADNAIAEWGQPKSNITHLIFCTSSGIDMPGADWALMK + 2 Oxidation (M)
☒	8550	918.1882	4585.9046	4585.1961	0.7085	1	10	30	1	AMTLAIGDGANDVSMIQVADVGVGISGQEGMQAVMASDFAVPKFR + 2 Oxidation (M)
☒	2438	731.5472	730.5399	731.4290	-0.8890	1	10	1.6e+02	1	RLTSGAK
☒	3032	537.6682	1073.3218	1072.5625	0.7593	1	10	2.7e+02	1	LLAGTGRDDR
☒	6488	674.8027	2695.1817	2696.2615	-1.0798	0	10	1.2e+02	1	AGEDLIGGMFGLTLGGAFFAESMFSR + Oxidation (M)
☒	6519	907.4200	2719.2382	2718.3622	0.8760	1	10	1.3e+02	1	YECAAVILHSPLMSGLRVAFPDTR + Oxidation (M)
☒	6524	908.4335	2722.2787	2723.2929	-1.0142	1	10	1.3e+02	1	LVDVAQDCIITQDDCGTSLGIKMR + Oxidation (M)
☒	6608	938.1367	2811.3883	2811.3776	0.0107	0	10	1.3e+02	1	EDVIDFIAGMSVLELSELIGEFEEK
☒	6618	704.8259	2815.2745	2814.3270	0.9475	0	10	1.2e+02	1	SEGLEVDDASFGLELDMLYGGQVNLK + Oxidation (M)
☒	6187	861.7116	2582.1130	2582.3228	-0.2098	0	10	1.3e+02	1	QDFSAGEVAVVLLLPDDETPLAQR
☒	7971	1034.1906	4132.7333	4132.8381	-0.1048	1	10	40	1	AHPTVGTCSGMFTANTMGSMSEALGMALPGSASPTATSSRR + 3 Oxidation (M)
☒	2679	914.3913	913.3840	912.5392	0.8448	0	10	3.8e+02	1	ENIAVVIR
☒	3671	694.0775	1386.1404	1386.7103	-0.5699	0	10	1.9e+02	1	TNSPAPTTSGVVTR
☒	4962	447.9615	1787.8169	1788.8782	-1.0613	0	10	2.3e+02	1	VTFPSDEDIVSGAVEPK
☒	5028	910.7657	1819.5168	1818.9853	0.5315	0	10	1.5e+02	1	VAVLGFGSQGHAHALSLR
☒	5551	707.6894	2120.0464	2120.9783	-0.9319	0	10	1.9e+02	1	LEEQDEIVMSSVEQPSASK + Oxidation (M)
☒	6020	626.0573	2500.2001	2501.2180	-1.0179	0	10	1.5e+02	1	ETGVSQATVAANTHSVAIANMANTK + Oxidation (M)
☒	7515	910.4747	3637.8697	3637.8895	-0.0198	1	10	73	1	SMVVPEMIGLTIAVHNGRQHVPILVTENMVGHK + 2 Oxidation (M)
☒	7883	1352.5312	4054.5718	4054.9998	-0.4280	1	10	39	1	QVSDGIAHVHASFNNTIVTITDRQGNALSWATAGGSGFR
☒	4553	809.5883	1617.1620	1617.7594	-0.5974	0	10	1.7e+02	1	ATGLSSTPTGDEAQAGR
☒	4584	814.5223	1627.0300	1626.8325	0.1975	1	10	2.1e+02	1	TAAKTGEGLSESAIHR
☒	5569	1071.5491	2141.0836	2141.2104	-0.1268	0	10	1.8e+02	1	MWQLLLPTALLLLVSAGMR + Oxidation (M)
☒	5726	572.4753	2285.8721	2285.1514	0.7207	0	10	1.3e+02	1	MFYNSLIPSEQQFLVNAIR + Oxidation (M)
☒	6206	863.4077	2587.2013	2586.3330	0.8683	0	10	1.4e+02	1	FDVVGAPYSLLSVSLSASQNLYTR
☒	8338	1118.9240	4471.6669	4470.9753	0.6916	0	10	30	1	QQQYLQNMIGNTTSGSDMSVLGFSQTEMNGMSDIFSFFSK + Oxidation (M)
☒	8479	906.6033	4527.9801	4528.1859	-0.2058	1	10	35	1	QTGSASSMVNGVVRLLTKPWDVVPMVTQMAMTDTTPFGQQR + 4 Oxidation (M)
☒	2617	872.6062	871.5989	870.5109	1.0880	1	10	3.9e+02	1	LGRMLGPK
☒	4985	604.3375	1809.9907	1808.9097	1.0809	0	10	2.2e+02	1	AWVSWEQTFNITVTK
☒	5003	906.4636	1810.9126	1809.8819	1.0308	0	10	2.2e+02	1	MQDTSVSLGYQLDLPK + Oxidation (M)
☒	5085	465.5021	1857.9793	1857.9182	0.0611	1	10	2.2e+02	1	VVFTTNMEEALFDSKK
☒	5736	576.2180	2300.8429	2301.1199	-0.2770	0	10	1.2e+02	1	GFTDSYLLVMILENEVGETR + Oxidation (M)

☒	8250	883.7912	4413.9196	4413.2468	0.6729	0	10	38	1	EIADEVGAYLFVDMAHVAGLIAAGVYPSPLPHAHVVTTTTHK + Oxidation (M)
☒	8253	883.7927	4413.9271	4413.9557	-0.0285	1	10	38	1	VASTMSGGANTMIEHGDTLPSQLGTMVINTEDEEEEGTMKR + 3 Oxidation (M)
☒	7334	580.6039	3477.5797	3476.6713	0.9084	0	10	78	1	VICNGPNETIPGRPTVPQCCYGFCVDLLIK
☒	8429	753.6873	4516.0801	4517.2036	-1.1234	1	10	38	1	GGPAGDLYIFLSVKPHEFYQRDGADLYCSVPISMTTAALGGK + Oxidation (M)
☒	3209	585.2813	1168.5480	1168.6928	-0.1448	1	10	3e+02	1	TLTRVPGIGQK
☒	3982	722.5908	1443.1670	1443.8449	-0.6779	1	10	1.9e+02	1	VFNVEVEILVRK
☒	7662	955.2327	3816.9017	3815.9967	0.9050	1	10	65	1	IMLSPHPYALRSHLCVGEETASLGVYLHSKPVVR
☒	8019	699.3364	4189.9747	4191.0989	-1.1241	1	10	52	1	AIRLIQACVDVLSSNGWLSPALAAMELAQMVTQAMWSK + 2 Oxidation (M)
☒	2642	445.1187	888.2228	888.4930	-0.2701	1	10	3e+02	1	LGFGRSPR
☒	4361	777.9373	1553.8600	1553.8413	0.0188	1	10	2.6e+02	1	DLAPEQVSAEVLRK
☒	6817	584.4355	2917.1411	2917.4756	-0.3345	1	10	89	1	MWNLAERFVAGESIESAIQAVQALER
☒	7049	764.8320	3055.2989	3055.4054	-0.1065	1	10	93	1	RVGDADTGPNTGGMGAYSPAPVVTQDVHDR + Oxidation (M)
☒	7451	719.5323	3592.6251	3591.6895	0.9356	1	10	77	1	METQTVQQELESLPTTKMAQTNPMPGSVGPWK + 3 Oxidation (M)
☒	7757	991.2135	3960.8249	3959.8202	1.0047	1	10	57	1	CPETLFQPAFIGMESAGIHETTYNSIMKCDIDIR + Oxidation (M)
☒	7923	1028.2227	4108.8617	4108.0246	0.8371	1	10	49	1	MDLATRFLQVLSSTQLDESPSVAPNGTMVIYGTTHQGK + Oxidation (M)
☒	4203	755.8373	1509.6600	1510.6324	-0.9724	0	10	2.5e+02	1	GEGTYLWDADDNR
☒	7804	801.3381	4001.6541	4000.6472	1.0069	1	10	44	1	AAGANSADAAAAKGNGDESAIDDSGDSSDGIDTTNMDDVADK + Oxidation (M)
☒	7990	833.5625	4162.7761	4162.0899	0.6862	1	10	42	1	DRPGSVLMNVVSGGIAEASAQQAGDLMQDLKTGHLLGASPR + Oxidation (M)
☒	8754	957.6215	5739.6853	5739.6926	-0.0073	1	10	18	1	IIKDGLWENNGVLCMLLGMCPTMAMTGTATNGLGMGLATAAVMAASNLMVAMFR + 4 Oxidation (M)
☒	4866	864.8283	1727.6420	1727.9709	-0.3289	1	10	1.8e+02	1	SVEIPFIKTEVPEIK
☒	6888	992.4503	2974.3291	2973.4212	0.9079	1	10	1.1e+02	1	TQAANIKSMDPASGYSNNIFTIDQMLK + Oxidation (M)
☒	2512	781.7193	780.7120	781.5174	-0.8054	0	10	78	1	IKPVGIR
☒	6549	691.7921	2763.1393	2763.4113	-0.2720	1	10	1.1e+02	1	FLASDKPQEMANASLTLETPLTSKR + Oxidation (M)
☒	7077	780.8557	3119.3937	3120.4968	-1.1031	1	10	97	1	MTVPNDDTWGPATSVGTTATMAAAARAIATR + Oxidation (M)
☒	7405	885.1570	3536.5989	3535.7154	0.8835	1	10	77	1	WATGWGRALHTSGSAPAGSSAESALLPEMGQLDPK
☒	3064	553.5494	1105.0842	1104.5849	0.4994	0	10	2.4e+02	1	LMAVSEQVTK
☒	3215	587.6915	1173.3684	1172.5786	0.7899	0	10	2.6e+02	1	INSSNVPGTER
☒	3365	635.5446	1269.0746	1269.7404	-0.6658	1	10	2e+02	1	AAALAAEITGVRK
☒	3721	704.1608	1406.3070	1406.7187	-0.4117	1	10	1.9e+02	1	SDIILSMRATER + Oxidation (M)
☒	6754	719.3301	2873.2913	2872.3987	0.8926	0	10	1.2e+02	1	EVLANMGLSLGMSVSGWPPENIEDLAK + Oxidation (M)
☒	7012	1007.8110	3020.4112	3020.4302	-0.0190	1	10	1.1e+02	1	ILLMKHATLICSNMMMNAFFSINEMK + 4 Oxidation (M)
☒	3409	644.3538	1286.6930	1286.7632	-0.0701	0	10	3.2e+02	1	LLLLLTIADMR + Oxidation (M)
☒	5817	802.7520	2405.2342	2404.1766	1.0575	1	10	1.7e+02	1	YYMITEQTCLQNITLGKANK + Oxidation (M)
☒	6035	838.7640	2513.2702	2513.1566	0.1135	1	10	1.6e+02	1	NYSIASLDGMFERTITMNGFSK + 2 Oxidation (M)
☒	6503	542.6310	2708.1186	2709.3036	-1.1850	1	10	1.1e+02	1	SAERAHGLMLAHMEAEPLLSLDMR + 2 Oxidation (M)
☒	6943	600.1090	2995.5086	2996.4451	-0.9364	1	10	1.2e+02	1	QAGLVTGMADGVVASNHAGYEQFEFRVK + Oxidation (M)
☒	8056	708.1623	4242.9301	4243.1371	-0.2070	1	10	47	1	MNVSDLLNILNELLHPEYFSDYGPNGLQVGNAQTAIRK
☒	2552	403.4360	804.8574	805.4294	-0.5719	1	10	1.9e+02	1	RTISTQT
☒	3067	553.8281	1105.6416	1106.6196	-0.9780	1	10	3.5e+02	1	ELLQSKHPR
☒	5322	992.1230	1982.2314	1982.0446	0.1868	1	10	1.6e+02	1	QESRTDHPLGIVPHQIR
☒	5772	784.1721	2349.4945	2348.2991	1.1953	0	10	1.2e+02	1	IFGQVSQDGLALVVLALYLSDK
☒	6027	838.3999	2512.1779	2511.3348	0.8430	0	10	1.6e+02	1	SSVIIGLFCGGGFALIAFVLWER
☒	6411	889.7776	2666.3110	2665.2150	1.0959	0	10	1.4e+02	1	NHGNNEADAALQGLAQQGVDMEDLR
☒	7927	822.7800	4108.8636	4109.7399	-0.8763	0	10	50	1	YDQFGHAGVGSSAASGGGQYGAGASDFSDIFSAFNDMFGGGK + Oxidation (M)
☒	6173	642.7962	2567.1557	2566.2888	0.8669	1	10	1.4e+02	1	GVSSAASAANAAIDHVRDWVLGTNGK
☒	7723	785.3499	3921.7131	3920.7256	0.9876	0	10	55	1	VSGSGMMSTPLLGATASGGMSMPQMAPPTSGDMFSPLMR + 3 Oxidation (M)
☒	7882	1014.6481	4054.5633	4053.8949	0.6684	0	10	40	1	WGLDPSLDFFEVTGNCNLGRPNSEGSNQSLMGSVTIR
☒	8411	753.5222	4515.0895	4514.2349	0.8546	0	10	40	1	AYGLENAMSGELVEFSNGVLGMAQNLDATDVGIIVLGDFLSIR + Oxidation (M)
☒	8562	1156.2390	4620.9269	4620.1207	0.8062	1	10	28	1	SSHSGSTAIIYPNDMNKATVILQDTYCTVTPGQACVVYDGDR + Oxidation (M)
☒	3737	706.3523	1410.6900	1409.7627	0.9274	0	10	2.8e+02	1	VSVSGPAAAAVPTAGR
☒	7508	910.4733	3637.8641	3636.8221	1.0420	1	10	75	1	LGLLEALLKVGDWQHAQNIMDQMPPYYAASHK
☒	7634	954.9819	3815.8985	3816.0641	-0.1656	1	10	68	1	IVEASTHVPIVLSSNTCIGINLMNEIVSKIAPQLR
☒	7790	999.7109	3994.8145	3993.6519	1.1626	1	10	55	1	LDAEDGEEEEEPEGKPSDAESSESSDDEKGWVEEVR
☒	8335	1118.7467	4470.9577	4470.1505	0.8072	1	10	37	1	VMGDIPVELINEVMSTITDPAGMLGPETNFLSAHSARDEAAR + Oxidation (M)
☒	6671	952.7627	2855.2663	2854.4573	0.8089	0	10	1.2e+02	1	VVAALGDSLTTAVGARPNNSSDLPTSWR
☒	6893	994.7402	2981.1988	2981.3958	-0.1970	1	10	88	1	TSEEVAQVGTIAGNGDESVGKMIAEAMQK + 2 Oxidation (M)
☒	6997	1004.0951	3009.2635	3010.4351	-1.1716	1	10	93	1	ISMNSADKIHFSLGCTGSFPMAVTPAQK + Oxidation (M)
☒	7014	605.0901	3020.4141	3020.3291	0.0850	0	10	1.1e+02	1	SCDMWSLGVAMYILLCGYPPFYSMK + 2 Oxidation (M)
☒	7243	844.5974	3374.3605	3373.6735	0.6870	0	10	66	1	LGMAPMHFWLPEVMAGLDLTTGMILATWQK + Oxidation (M)
☒	7267	681.6788	3403.3576	3402.7852	0.5724	0	10	65	1	MEVAAMEISTSLLLTTVALSVIVCYALVFSR + Oxidation (M)
☒	7590	745.7780	3723.8536	3722.8800	0.9736	1	10	74	1	DHFIQLLKPYMPNITVDMLEYGGSGVRSQAISK + Oxidation (M)
☒	8086	1062.5060	4245.9949	4245.1371	0.8578	1	10	50	1	AIVIVALDNLTKGSSGQALQNANLMLGEDETAGLMMAPLFP + 2 Oxidation (M)
☒	8438	1130.0283	4516.0841	4515.1369	0.9472	1	10	39	1	DWALGTPEGDWVSMAVPSQGEYGIPEGIVYSFPVTAKDGAYR
☒	6991	751.3571	3001.3993	3001.3732	0.0261	1	10	1.1e+02	1	CCSESLVNRRPCFSGLEVDETYVPK
☒	8064	708.5056	4244.9899	4245.2528	-0.2628	1	10	51	1	ELLDVLSVLSLMERNSPIVISPLLMNLTVENDFSTTVK + Oxidation (M)
☒	5135	935.4312	1868.8478	1869.7518	-0.9040	1	10	2.2e+02	1	KDAAPAGMPGGMGGMGGMDF + Oxidation (M)
☒	6082	633.5305	2530.0929	2529.2534	0.8395	1	10	1.4e+02	1	GGYVLAIDQGTTSTRAIVFDGNMK + Oxidation (M)
☒	6456	889.7817	2666.3233	2665.2150	1.1082	0	10	1.4e+02	1	NHGNNEADAALQGLAQQGVDMEDLR
☒	7647	764.3862	3816.8946	3816.7434	0.1512	1	10	67	1	LLCGGEALKGDGFDNGAWVAPTVFTDCSDEMTIVR + Oxidation (M)
☒	3416	645.6626	1289.3106	1288.6775	0.6331	1	9	2.2e+02	1	QLLEYDPQKR
☒	4522	801.1354	1600.2562	1599.8766	0.3796	1	9	1.8e+02	1	ILAQMGLDAQGLARK + Oxidation (M)
☒	5975	625.2831	2497.1033	2496.1156	0.9877	1	9	1.5e+02	1	RTDCGTIQGISVNTQNGMMPER + 2 Oxidation (M)
☒	7829	806.3752	4026.8396	4027.7919	-0.9523	0	9	55	1	QIEDGEWSATDNPLVNAPHTQVDLMESEWTHGYSR + Oxidation (M)
☒	8078	850.2055	4245.9911	4245.1371	0.8540	1	9	51	1	AIVIVALDNLTKGSSGQALQNANLMLGEDETAGLMMAPLFP + 2 Oxidation (M)
☒	2437	366.1995	730.3844	729.4021	0.9823	0	9	1.6e+02	1	GPLNTTK
☒	4246	509.5096	1525.5070	1524.7606	0.7464	1	9	1.9e+02	1	KQIGAAAYIECSSK
☒	6535	684.0510	2732.1749	2732.1377	0.0372	1	9	1.2e+02	1	DDLQEMIDNDHGAEAVCRFCGHK + Oxidation (M)
☒	3401	643.2695	1284.5244	1285.6878	-1.1633	1	9	3.1e+02	1	KDGEVLDGNVLK
☒	5061	615.4407	1843.3003	1843.8696	-0.5693	1	9	1.6e+02	1	TISANIGMFGTMKEITD + Oxidation (M)
☒	5415	1014.4629	2026.9112	2027.1051	-0.1939	1	9	2e+02	1	ASAQFDEVVVAVLVNPNKK
☒	5594	543.7441	2170.9473	2171.0998	-0.1525	0	9	1.8e+02	1	GVTVETVFSVDEFSASVLTGK
☒	6130	511.8315	2554.1211	2554.2988	-0.1777	1	9	1.4e+02	1	EGGMSVTPAAYSSLNYLSKLPIEK
☒	8198	873.4146	4362.0366	4363.1504	-1.1138	1	9	46	1	ESLILAGDIFMKAVEEFNAELIPVDSEHSAIFQCLQSGR
☒	8468	647.8603	4527.9712	4528.3646	-0.3934	1	9	37	1	KVETALGMGMATTFVMTLASICAWLINDFILLPLNIPYLR + 2 Oxidation (M)
☒	2410	357.2341	712.4536	712.3755	0.0781	0	9	52	1	LPTPGAAS
☒	8393	753.1850	4513.0663	4513.1166	-0.0503	1	9	41	1	FLADVILDDGTAVTAHCANSGSMASVKEPGSPVWLSESPNPDR + Oxidation (M)
☒	2644	445.2857	888.5568	888.4553	0.1016	0	9	4.4e+02	1	DGGVIDSVK
☒	5045	917.4196	1832.8246	1833.9836	-1.1589	1	9	2.3e+02	1	ILTERELESLLEYAR
☒	5314	493.9992	1971.9677	1972.0557	-0.0880	0	9	2.1e+02	1	LTPYYLSSGAALDLFLTK
☒	6621	939.7629	2816.2669	2817.3432	-1.0763	0	9	1.3e+02	1	HLAEYTHVEAECPFLTFEELLNR
☒	7641	764.3857	3816.8921	3816.7434	0.1487	1	9	68	1	LLCGGEALKGDGFDNGAWVAPTVFTDCSDEMTIVR + Oxidation (M)
☒	6486	674.2950	2693.1509	2693.3054	-0.1545	1	9	1.2e+02	1	GLSDLQPWPEQMAIACGSRAHLEK
☒	7567	619.7824	3712.6507	3713.1286	-0.4778	1	9	66	1	NTGAAWSLLEGKMIFFYVITVIVSVVIIYLLIK
☒	8434	1130.0276	4516.0813	4515.1369	0.9444	1	9	40	1	DWALGTPEGDWVSMAVPSQGEYGIPEGIVYSFPVTAKDGAYR
☒	6500	903.3823	2707.1251	2707.1879	-0.0628	1	9	1.2e+02	1	ASSGLSDEEVEQMVRDAEANADADAK
☒	8074	850.2050	4245.9886	4247.0919	-1.1033	1	9	51	1	VHEMLPCTILGAKPEPKVVTLGNDYDYVWDISAAAGSSK + Oxidation (M)
☒	2555	806.6035	805.5962	806.4208	-0.8246	0	9	2.5e+02	1	DILMTAK + Oxidation (M)
☒	5495	1031.9723	2061.9300	2063.1123	-1.1822	1	9	2e+02	1	SGRTALHLAAEEANLELIR
☒	6483	673.0897	2688.3297	2687.3667	0.9630	1	9	1.4e+02	1	VRDWNIQGLPSDAFSTENGIIVTR
☒	8602	934.4197	4667.0621	4667.2615	-0.1994	0	9	32	1	NNEEEEAVDVVHLTHLTTLHSIYHMRPGFAEFASRPYVSR + Oxidation (M)
☒	4445	525.2456	1572.7150	1571.7620	0.9529	0	9	2.7e+02	1	FSGFLVAPDSDVYR

☒	6159	639.7897	2555.1297	2554.3616	0.7681	1	9	1.4e+02	1	AAIRASSNAIIVLHNHPSGDVTPSK
☒	6853	588.7041	2938.4841	2939.5037	-1.0196	0	9	1.3e+02	1	VVDAMMPVDQYIGGIEHAILHLLYSR
☒	6643	708.3251	2829.2713	2829.2664	0.0049	0	9	1.3e+02	1	SSINIMSAFESPFGPNSNGSQGSNSWK
☒	6666	952.1249	2853.3529	2852.3585	0.9943	0	9	1.3e+02	1	SMASTGFQAANLGDAIQIVNQMLDWR + Oxidation (M)
☒	6673	952.7637	2855.2693	2854.4573	0.8119	0	9	1.2e+02	1	VVAALGDSLTTAVGARPNNSSDLPTSWR
☒	7794	799.9725	3994.8261	3993.7957	1.0305	1	9	57	1	ASTVSPGGYMVPKGTTASATSAASAASSSSSSFQPSPENCR + Oxidation (M)
☒	8141	1075.7307	4298.8937	4299.9210	-1.0273	0	9	42	1	TALQAAGSIAGLMITTEAMVADLPEDKPAAAPDMGGMGGMGGMM + 4 Oxidation (M)
☒	3255	606.0758	1210.1370	1210.5764	-0.4394	0	9	2e+02	1	NVGAMSAYLNR + Oxidation (M)
☒	7312	1152.5525	3454.6357	3453.6697	0.9660	1	9	87	1	HGRVDDSPYGGGAGMLLMPQPIFDAIADLPEAK + Oxidation (M)
☒	7725	785.5510	3922.7186	3922.9326	-0.2140	1	9	57	1	LMASLVASVVLTACSGGGSSGSSSKPNSELTPKVDMSAPK + Oxidation (M)
☒	4323	771.8265	1541.6384	1540.7668	0.8717	0	9	2.6e+02	1	ATAHVDDIVAMVQR + Oxidation (M)
☒	5586	721.1797	2160.5173	2160.0746	0.4427	1	9	1.3e+02	1	MNYQRPKGTADILPGQSER
☒	5831	805.3557	2413.0453	2413.0373	0.0080	1	9	1.6e+02	1	CIESSDDNDSIDSMSIQQKNK
☒	6153	639.7892	2555.1277	2555.3067	-0.1790	1	9	1.4e+02	1	SPVTSFAVVHLNGSVDWMVAQRR
☒	6587	933.7556	2798.2450	2797.4684	0.7766	1	9	1.3e+02	1	LCGISAGASTPDWIVQNVIDKIEALK
☒	7543	919.3936	3673.5453	3672.8280	0.7173	0	9	62	1	LVVGAMAGANALSGISCGLLASVDALTYGQFAEYGAR
☒	8585	927.8278	4634.1026	4633.3309	0.7718	0	9	36	1	QHQHYLESMQLINVPQVVNEKPVLEAMPTSEAPQPAVPAPGK + 2 Oxidation (M)
☒	8610	935.0157	4670.0421	4671.2070	-1.1648	1	9	33	1	LIVDLMYEGGIANMNYSISNNAEYGEYVTGPEVITAASKEAMK + Oxidation (M)
☒	8887	742.4623	5931.6402	5931.9333	-0.2931	1	9	13	1	EGSITIHDTLQGEIVIENSHIDDMILIRTDGTATYMLAVIVDDHDMGITHIIR + 2 Oxidation (M)
☒	2593	850.4088	849.4015	849.3902	0.0113	0	9	3.7e+02	1	VMEGEAAK + Oxidation (M)
☒	4424	784.4880	1566.9614	1566.7460	0.2154	1	9	2.4e+02	1	DIVDYERNQLMR + Oxidation (M)
☒	5614	731.3099	2190.9079	2192.0817	-1.1738	1	9	1.7e+02	1	TLTAVDSALMMVDGAKGVEER
☒	6329	878.4244	2632.2514	2632.2803	-0.0289	1	9	1.5e+02	1	AEPVGDLVVDLSLDAARFDEQMAR + Oxidation (M)
☒	6668	952.4265	2854.2577	2854.4573	-0.1997	0	9	1.2e+02	1	VVAALGDSLTTAVGARPNNSSDLPTSWR
☒	7045	1018.8226	3053.4460	3053.4045	0.0415	1	9	1.2e+02	1	QEAAEGMLIILAGGDRSVFEECHSCFK
☒	7397	590.2748	3535.6051	3535.7612	-0.1561	1	9	82	1	EGTFDALQTLSLCLAAMTGMVRDLEPVPEVLK + 2 Oxidation (M)
☒	7775	1327.6902	3980.0488	3981.0517	-1.0030	1	9	64	1	MATASQGKVTQVIGAVVDVQFDGGLPPILNALETENNGK
☒	7892	813.9202	4064.5646	4063.9731	0.5915	1	9	43	1	ENSHFQATSLETLRNMVAANAGITFMPELAVLNEGTR + 2 Oxidation (M)
☒	8641	789.5637	4731.3385	4730.1674	1.1712	1	9	37	1	MMFTVFLLIVLATTVVSFSTDDESDGSNEEPSADQTARSSMNR + 2 Oxidation (M)
☒	5796	787.3836	2359.1290	2359.1961	-0.0671	1	9	1.8e+02	1	ADAEVKLILYHWTHSFSSQK
☒	6832	975.9437	2924.8093	2924.5508	0.2584	1	9	92	1	NPVRFRPAVVSSPSVPEGTYTVLNNPK
☒	7201	832.1249	3324.4705	3323.5226	0.9479	0	9	88	1	LSSGFGGGGGGSGSGGGSGGGGLGSSSHPLSSLLSMWR + Oxidation (M)
☒	5369	673.6740	2018.0002	2017.9197	0.0805	0	9	2.2e+02	1	EAMSQLHTAMGEVADLQR + 2 Oxidation (M)
☒	5878	820.0609	2457.1609	2457.0617	0.0992	1	9	1.6e+02	1	MGLSYDWGRELATCDVDYYK + Oxidation (M)
☒	7937	1028.2301	4108.8913	4109.9976	-1.1063	1	9	54	1	YASAAAAGSSGSTAEESTALIESTTTATATTTSTTTTGAVGKVK
☒	3441	434.8470	1301.5192	1300.6095	0.9097	1	9	3.1e+02	1	KCSNPQFQHR
☒	5715	761.2565	2280.7477	2280.0721	0.6756	1	9	1.2e+02	1	YNHWFDGMALLHQFRMAK + Oxidation (M)
☒	6798	969.4630	2905.3672	2905.4235	-0.0564	1	9	1.3e+02	1	LIVVDEKGTVLDGDQIMALCADDMLR + Oxidation (M)
☒	2429	727.9807	726.9734	727.3501	-0.3766	0	9	52	1	ETHVATA
☒	5194	631.3320	1890.9742	1890.9510	0.0232	1	9	2.4e+02	1	MAVTIVLGSQWGDEGKGK + Oxidation (M)
☒	5363	671.7579	2012.2519	2013.0315	-0.7796	0	9	1.6e+02	1	VAFMNPLQTMLSFLIEK + 2 Oxidation (M)
☒	8163	1081.5107	4322.0137	4322.8889	-0.8752	1	9	51	1	SSQEEPEAGSGSVEIPVMTSPGTDGVSGGGGSGGGSGGGGGSMRGIDR + Oxidation (M)
☒	8477	647.8610	4527.9761	4527.1006	0.8755	1	9	38	1	HVIVDARPRTNALAQMALGGGTENMDNYNFFLADNNMGVDK + 3 Oxidation (M)
☒	5557	711.2985	2130.8737	2131.1637	-0.2901	1	9	1.8e+02	1	SRSISSLGFGAVAGVGIDITPK
☒	7472	602.4442	3608.6215	3608.7583	-0.1367	1	9	79	1	FAEMNGVWEGHATQAAPPAATATVNRWIIGETGR
☒	2682	915.7579	914.7506	915.4297	-0.6791	1	9	3.3e+02	1	DKGENEPK
☒	4027	730.2886	1458.5626	1457.7726	0.7901	0	9	2.7e+02	1	TENSLLLLDEVGR
☒	5825	803.6830	2408.0272	2409.0940	-1.0668	1	9	1.6e+02	1	DTVEARLAMAEIHESMGNYEK + Oxidation (M)
☒	7171	820.8600	3279.4109	3279.6367	-0.2258	0	9	89	1	APLQELEMAPVTATISDTLPITVEAADACPR
☒	7524	912.1416	3644.5373	3644.5995	-0.0622	1	9	64	1	MASPSKGNDLFSSDEEGPAMVAGPGPGPGGAEGAAEER + Oxidation (M)
☒	7711	969.9849	3875.9105	3875.0720	0.8385	0	9	71	1	VSLGLAASLDTVADLELLQASGLSFLLPQQYLNYPR
☒	7339	1160.5320	3478.5742	3478.6782	-0.1041	1	9	83	1	LTLPFVDMSKAAAMDGTSTTLNYATALMGGNLR + 3 Oxidation (M)
☒	3449	652.3860	1302.7574	1303.6740	-0.9166	1	9	3.3e+02	1	LVNQMMLDGKR
☒	4555	810.2844	1618.5542	1617.7239	0.8304	0	9	2e+02	1	AEDMVAANAMASHLR + 2 Oxidation (M)
☒	6966	750.1359	2996.5145	2996.6488	-0.1343	1	9	1.2e+02	1	LLTPTHFEFVFKNATPAVSPNLTLLAR
☒	7006	604.4561	3017.2441	3016.4859	0.7582	1	9	93	1	RLGVIAMNTPVEVDIYGHANSTNVMGSR + Oxidation (M)
☒	7406	885.1599	3536.6105	3535.7327	0.8778	0	9	83	1	VSGVQDLIAVYGELAAQCDYALHLGLTEAGMGSK
☒	7967	689.4996	4130.9539	4130.9954	-0.0414	1	9	57	1	QAPSAPETGGAGASEAPAPTGGEDGAGAETTPVAEPTKLVLPMK + Oxidation (M)
☒	8451	1130.2672	4517.0397	4518.0984	-1.0587	1	9	39	1	LIMAGNYEEALSVAKVQVEMGAQVLDINMDDGMLDGPSAMTK + 4 Oxidation (M)
☒	8542	655.2750	4579.8741	4580.2488	-0.3748	1	9	31	1	NGDVIVFNLETLQPTMVIEAHKGEIAAMAISFDGTLMATASDK + 2 Oxidation (M)
☒	7146	813.8654	3251.4325	3251.5923	-0.1598	1	9	93	1	ATADYMGMLATVMNALALQDAMRHIGLVSR + 2 Oxidation (M)
☒	2652	450.2727	898.5308	898.3967	0.1341	0	9	3.6e+02	1	ASPDPCPR
☒	5266	972.4059	1942.7972	1941.9656	0.8316	1	9	2e+02	1	NESAQGALSHNFANIKNK
☒	5512	692.0110	2073.0112	2072.1993	0.8118	1	9	2.1e+02	1	IDKVHELSVAPLIGEAIIR
☒	5601	1091.0431	2180.0716	2181.0637	-0.9920	1	9	2e+02	1	FVIAQANAALGDDMRFAEAR + Oxidation (M)
☒	7207	555.2710	3325.5823	3326.5581	-0.9757	1	9	1e+02	1	DMMQSELELLPPNTQVMEGQHVEKQLDR + 2 Oxidation (M)
☒	7884	676.9345	4055.5633	4054.9322	0.6312	1	9	41	1	MVMSVMSVFVTLGEVPFIVATDDDVTTQMARMLETK + 4 Oxidation (M)
☒	7991	1041.7015	4162.7769	4161.7997	0.9772	1	9	45	1	QDMPIQQQHATGNMSRFVLYQLQNSNDCDCSTTSR + 2 Oxidation (M)
☒	2781	497.9191	993.8236	993.3974	0.4262	0	9	2.3e+02	1	EQWCSER
☒	6024	837.0898	2508.2476	2507.4475	0.8000	1	9	1.7e+02	1	GIVIDAWVRVSLVGIELLSIEAR
☒	6884	990.1194	2967.3364	2968.4173	-1.0809	0	9	1.2e+02	1	MLYIHMLGYDVDFGHMEAVSLISAPK + 2 Oxidation (M)
☒	7044	1018.7733	3053.2981	3052.5406	0.7574	1	9	1e+02	1	YGGPLIAGIQDHISGGYLLTREGAYFER
☒	7098	633.6771	3163.3491	3163.6403	-0.2912	0	9	91	1	VQFQFVLLTDTLYSPLPPELLPTEMEK + Oxidation (M)
☒	7536	733.1233	3660.5801	3661.7610	-1.1809	0	9	66	1	GLIQSDQELFSTPGADTIPLVNQYSSDMSVFFR
☒	7626	756.3430	3776.6786	3775.9157	0.7629	0	9	67	1	EEVVDVVSVTGGHLGSGLGVVELTVAIHSVFDTPDDK
☒	3089	1122.7725	1121.7652	1121.5209	0.2443	0	9	2.9e+02	1	LDTLCDMVR
☒	3577	679.2512	1356.4878	1357.5795	-1.0916	1	9	2.6e+02	1	CDKEGAWMAYK
☒	6819	973.4299	2917.2679	2917.4432	-0.1754	1	9	1.2e+02	1	VLEYWETKLEMYGTFQAVNTSRPR
☒	6933	600.1085	2995.5061	2996.4691	-0.9629	1	9	1.3e+02	1	VSAAKLESMGLITCGDIQQCDLAMLLK + 2 Oxidation (M)
☒	6996	1003.7617	3008.2633	3007.4855	0.7778	1	9	99	1	AANQEIRELHVGLLNMMPDAALEATER + Oxidation (M)
☒	8162	1077.7566	4306.9973	4306.2334	0.7639	0	9	51	1	AEESIDVDSIAVFPLALPLYTGPGAITAGIVLYSQAGDVVMK + Oxidation (M)
☒	2489	384.2344	766.4542	766.4450	0.0093	0	9	89	1	AHGILTR
☒	7342	580.7708	3478.5811	3479.6848	-1.1037	1	9	85	1	NAKLFMNLFAMMVSTGIPELQSMEDLNYLK + 2 Oxidation (M)
☒	7485	605.6008	3627.5611	3627.8031	-0.2420	0	9	69	1	EQDLNNFFGAMYAAVLFLGATNAATVQPAVAIER + Oxidation (M)
☒	8363	1122.7438	4486.9461	4486.1086	0.8375	1	9	37	1	LIMAGNYEEALSVAKVQVEMGAQVLDINMDDGMLDGPSAMTK + 2 Oxidation (M)
☒	4575	542.9106	1625.7100	1626.8511	-1.1412	1	9	2.7e+02	1	TNAISIKNHMNLQK + Oxidation (M)
☒	5349	668.3224	2001.9454	2001.8805	0.0648	1	9	2.2e+02	1	LLRISSEDEMAGMDMTR + 3 Oxidation (M)
☒	6418	667.5851	2666.3113	2665.4448	0.8665	1	9	1.5e+02	1	GLFMTTNPVPVKAALGMLGLNVGPPR + Oxidation (M)
☒	6918	500.2574	2995.5007	2995.3985	0.1022	1	9	1.3e+02	1	SIAEAMLMDLEKQPGFVMSLPHTCMK + 2 Oxidation (M)
☒	7100	792.3486	3165.3653	3164.4657	0.8996	1	9	96	1	FSALADFMASFPQAVEEEGFEARIMMPK + Oxidation (M)
☒	7635	954.9828	3815.9021	3815.9967	-0.0946	1	9	73	1	IMLSPHPYALRSHLCVGEETASLGVYLHSKPVVR
☒	8664	806.3315	4831.9453	4831.6051	0.3402	1	9	27	1	AGTAWILGTSGLSPADEKAVEEIAALVPVVYAPNFAPGVNLVLALAEK
☒	4498	797.7349	1593.4552	1592.8059	0.6493	1	9	1.9e+02	1	LDWAEGPTAGPAPRR
☒	5069	925.9489	1849.8832	1848.9437	0.9395	1	9	2.5e+02	1	SLLQMIGQDMGLDIKR + 2 Oxidation (M)
☒	7940	1028.4723	4109.8601	4109.7399	0.1202	0	9	52	1	YDQFGHAGVGSSAASGGGQYGAGASDFSDIFSAFNDMFGGGK + Oxidation (M)
☒	4692	829.4116	1656.8086	1657.6067	-0.7981	0	9	2.8e+02	1	DMPPMPGGGMGGMGGMY + Oxidation (M)
☒	7061	1034.4718	3100.3936	3101.4459	-1.0524	0	9	1.1e+02	1	FGSDLDGNVTNSLSMVNGDSADLTLTSISR + Oxidation (M)
☒	7355	701.4955	3502.4411	3502.9545	-0.5134	1	9	71	1	EILTNINGIMKPGLNAILGPTGGGKSSLLDVLAAR
☒	7765	794.7852	3968.8896	3967.8676	1.0221	1	9	65	1	SALQNATSVASTFLTTEAAVASIKEPAPAMPAGGPGGMGMM + 3 Oxidation (M)
☒	8177	727.1586	4356.9079	4357.0665	-0.1586	1	9	43	1	TGNVDLVTVAMNMYTQGVSPNLDFSDLTSISEIVHRCNK + 2 Oxidation (M)
	4022	729.3200	1456.6254	1455.7259	0.8995	0	9	3e+02	1	GAGPNGWLLDGFPR

4747	562.2792	1683.8158	1683.8614	-0.0456	0	9	2.7e+02	1	MANQSEAVLPGIELGR
5304	655.3197	1962.9373	1964.0513	-1.1140	0	9	2.3e+02	1	ASVMVHLPALNLTNNINK + Oxidation (M)
6351	884.0959	2649.2659	2649.3618	-0.0960	1	9	1.5e+02	1	AGLGMMEGVLENVPSARISVVGIYR + 2 Oxidation (M)
6659	950.7838	2849.3296	2849.4294	-0.0999	1	9	1.4e+02	1	VTATIESNSGKTEELVLLDNGAGADAFK
5643	737.3776	2209.1110	2210.0130	-0.9020	1	9	2e+02	1	SGMSSASLCMENTLDPKQVR
5925	621.5460	2482.1549	2482.3253	-0.1704	1	9	1.7e+02	1	LPLTELSTEFHGLLLEAMKNAR
6322	526.6374	2628.1506	2627.2439	0.9068	0	9	1.4e+02	1	VSWAGLPIAEVATGDEFDCHAQVR
7226	835.6871	3338.7193	3338.6859	0.0334	1	9	99	1	MMLAETEVGVSWNVRMAALAMIAVAGLLTSR + 3 Oxidation (M)
3446	651.7673	1301.5200	1302.7183	-1.1983	0	9	3.2e+02	1	TQEILSQLPFK
4815	851.0510	1700.0874	1700.7464	-0.6590	0	9	2.2e+02	1	QEVTYMSFEGNQPR + Oxidation (M)
6836	976.0771	2925.2095	2925.2319	-0.0224	0	9	1.1e+02	1	HAEEESDDVSELTSHQSPQAPMGSGER + Oxidation (M)
7110	1063.4659	3187.3759	3188.4025	-1.0266	0	9	97	1	SMAESKPPTGTGGSGSAGSGAGASAGNGANPGADAER
7328	580.6030	3477.5743	3476.7360	0.8384	1	9	86	1	MLGYGTLFGDLACAINPIGELFKTEVYELAR + Oxidation (M)
8310	893.0182	4460.0546	4461.1695	-1.1149	1	9	43	1	MASDFLRGEGATSYGGGAAQIFFLTLMGVAEGVLLVLMSYDR + 3 Oxidation (M)
5639	1103.0127	2204.0108	2204.1153	-0.1045	1	9	2e+02	1	LYYYNNITFKDVAGLEGPK
6641	566.6811	2828.3691	2828.4394	-0.0702	0	9	1.5e+02	1	LLWAVEPLDGCGPLHPSWISLMPPK + Oxidation (M)
6929	500.2581	2995.5049	2994.4646	1.0403	1	9	1.3e+02	1	MKSVITISASLAISLMLCCTAQANDHK + 2 Oxidation (M)
7329	696.5223	3477.5751	3477.5633	0.0119	1	9	87	1	DSFIGENSRTCMIATISPGMASCENTLNTLR + 2 Oxidation (M)
7563	1238.2217	3711.6433	3710.7823	0.8610	0	9	70	1	MFFNLDNIDTALTLYMNLPMLFGGGDPNLLYR + 2 Oxidation (M)
7905	1021.9670	4083.8389	4083.9037	-0.0648	1	9	56	1	VNSMMETMTGKTPPASCGLTSEDLEAGELPPVDVLTFK + 2 Oxidation (M)
8367	641.9998	4486.9477	4486.2150	0.7327	1	9	38	1	KSVIFEMDPSPEILAVQAEYMHLAETLWAGTEPLDAMPLK + Oxidation (M)
2985	1045.3914	1044.3841	1043.5876	0.7965	0	9	3.9e+02	1	APPGGAPRPPK
6691	717.3193	2865.2481	2865.3969	-0.1488	1	9	1.3e+02	1	LGEMEVWAMEAYGAAYTLKEFLTVK + Oxidation (M)
8070	708.6712	4245.9835	4245.1371	0.8465	1	9	54	1	AIVIVALDNLTKGSSGQALQNANLMLGEDETAGLMMAPLFP + 2 Oxidation (M)
8401	904.0241	4515.0841	4515.2058	-0.1217	1	9	44	1	LNQIGAGDQGLMFGYATNETEELMPLPIMLSHHLMQRIAK + 3 Oxidation (M)
8613	669.4313	4678.9682	4678.8737	0.0945	1	9	30	1	KDQNNNNNNNNSNSNCQNGGGSICDDSSNSSTPSLSSYSNGNNK
3674	694.3136	1386.6126	1385.8547	0.7580	1	9	3.2e+02	1	LYKPILWRGLK
4240	760.8253	1519.6360	1519.7705	-0.1344	0	9	2.9e+02	1	DQSLMIFDIAVPR + Oxidation (M)
4678	550.8546	1649.5420	1648.8243	0.7177	1	9	1.9e+02	1	TWTVEPRAMQLSSK + Oxidation (M)
4880	870.4023	1738.7900	1739.8400	-1.0500	0	9	2.6e+02	1	LAMVTTTLEDPTGYGR + Oxidation (M)
6913	749.0864	2992.3165	2992.4861	-0.1696	1	9	1.2e+02	1	LSNSLSMLFNKMYLNGGCSVVPTGFLK + Oxidation (M)
8007	838.8015	4188.9711	4189.0775	-0.1064	0	9	59	1	VKPQINEGDSVLLQIEQEVSSVAEGNGSSNSSLGVTFNTR
3617	683.1941	1364.3736	1363.6983	0.6753	0	9	2.1e+02	1	LLIGQFSETNDK
6648	947.9485	2840.8237	2841.2473	-0.4236	0	9	97	1	FLNTSDPLELAAHCMEDIDADFSSK + Oxidation (M)
6702	956.8097	2867.4073	2868.4116	-1.0044	1	9	1.4e+02	1	VDPMAIVVFHQADIGEYVRHEETLT
7695	774.7697	3868.8121	3869.9305	-1.1184	1	9	72	1	LDEMGVERASVTLHVGAGTFKPIQGPVADHVMHAER + Oxidation (M)
8375	749.1653	4488.9481	4490.0992	-1.1511	1	9	39	1	DAIAGSTVSNEEMTPFESIDHISEPVITKSIEAQNMDDLPK + 2 Oxidation (M)
3269	610.1824	1218.3502	1217.6438	0.7065	1	9	2.5e+02	1	NLVNKMGGEIK + Oxidation (M)
6862	982.1187	2943.3343	2943.5211	-0.1868	1	9	1.3e+02	1	AHMFLATAAALGVAMFPTQINEAARGLR + Oxidation (M)
7283	570.9313	3419.5441	3420.5798	-1.0356	0	9	90	1	GGNAADAMVATMFCIGVVGMYHSGIGGGGFMLIK + 2 Oxidation (M)
7533	731.9319	3654.6231	3653.7362	0.8870	1	9	74	1	NFPYERRPTSAASIPFGQGDFYYGSLMVGGTPR + Oxidation (M)
7812	668.2845	4003.6633	4004.0870	-0.4236	1	9	50	1	SALFHLITHAYSKALLFLGSGSVIHSMETLVGYSPNK + Oxidation (M)
3689	698.2053	1394.3960	1394.7261	-0.3301	1	9	2.1e+02	1	LSTMIKNMVGASK + Oxidation (M)
6131	852.3824	2554.1254	2554.2432	-0.1178	1	9	1.5e+02	1	NIMTTSSNLKESDSGSILENVATK + Oxidation (M)
6593	562.4516	2807.2216	2806.3140	0.9077	1	9	1.3e+02	1	SYSLYLDAVQDWWHKNVDPEWR
6946	600.1098	2995.5126	2996.6488	-1.1361	1	9	1.3e+02	1	LLTPTHFEFVFKNATPAVSPNLTLLAR
7814	670.2807	4015.6405	4015.8631	-0.2226	1	9	45	1	TASDRQPGGRPQPPTLDPTSTSYESQLGQNSSSEESPK
6420	889.7778	2666.3116	2665.2150	1.0965	0	9	1.6e+02	1	NHGNNEADAALQGLAQQGVDMEDLR
6937	600.1087	2995.5071	2995.6688	-0.1617	1	9	1.3e+02	1	RPPTPTTTRPVMRPRGPVALPPVMTVR + Oxidation (M)
7755	793.1715	3960.8211	3960.8225	-0.0013	1	9	64	1	MQEVLVVVGGRALEEDEDGGEEPSHHTGNFAFYNTK
8001	838.8002	4188.9646	4190.0230	-1.0584	1	9	59	1	IWSIDSILRCMELESLTPEIPLPQDLQMPLCSMSR + 2 Oxidation (M)
8104	850.6042	4247.9846	4247.0919	0.8927	1	9	54	1	VHEMLPCTILGAKPEPKVVTLGNDYDYVWDISAAAGSSK + Oxidation (M)
6431	534.2697	2666.3121	2667.2448	-0.9326	1	9	1.6e+02	1	MYEKLAGMTGTAATEADELFDIYK
7161	816.6082	3262.4037	3262.6002	-0.1965	1	9	94	1	RSLLEMYADFMAHPDLLLAITAGATPEER + 2 Oxidation (M)
7802	667.9489	4001.6497	4000.9346	0.7151	1	9	49	1	VQMLQAMVHGVTTEECQAALQSHSWSVQRAAQYLK + Oxidation (M)
3163	581.2816	1160.5486	1161.4720	-0.9234	0	9	3.8e+02	1	SSSMNNSTYR + Oxidation (M)
3420	647.7877	1293.5608	1293.6122	-0.0513	1	9	3.2e+02	1	KGEESLLEMDK + Oxidation (M)
4238	760.3712	1518.7278	1519.6573	-0.9294	0	9	3.1e+02	1	EGGINLDDCADVSR
5832	1207.5323	2413.0500	2412.3165	0.7335	1	9	1.7e+02	1	DLALGGQGAPLAPFLDYALIGRGK
7788	665.2809	3985.6417	3986.1378	-0.4960	1	9	51	1	ALGLPVSVAFASFDTVPLASASIAQVHAATLPPDANGVRR
8137	1074.5117	4294.0177	4292.9711	1.0466	0	9	51	1	NNDGFTAFMNIFGNNYFDYIVPVIQIMLDYGADINDK + 2 Oxidation (M)
8577	661.5718	4623.9517	4623.1781	0.7736	1	9	32	1	SPPLGQAAALNPFTMYSQTHQGQHVLMNGGQAHQRFGMQMPK + 2 Oxidation (M)
2439	732.4292	731.4219	732.3766	-0.9547	0	9	1.7e+02	1	GSLSDVR
4280	768.9539	1535.8932	1535.8096	0.0836	0	9	2.9e+02	1	DWITELKPEHLR
5046	917.4205	1832.8264	1833.8059	-0.9795	1	9	2.5e+02	1	METRTAENTAIFMCK + 2 Oxidation (M)
6042	840.3985	2518.1737	2517.1920	0.9817	0	9	1.7e+02	1	ALIGFCGSPWTLATYMIESEGSK
6821	974.0983	2919.2731	2918.6456	0.6275	1	9	1.2e+02	1	GVDVKPFYIKKPVDNLFALMLILNR + Oxidation (M)
7316	866.1189	3460.4465	3460.5662	-0.1197	1	9	75	1	GDHASLENEKPGTGDVCSAPAGRNQSPEPSSGPR
8318	1118.4957	4469.9537	4470.1441	-0.1904	1	9	41	1	QTGSASSMGNGVVRLLTKPWDVVPMVTQMAMTDTTPFGQQR + 3 Oxidation (M)
8595	775.5056	4646.9899	4647.0236	-0.0337	1	9	34	1	AEAVDGDDSPLAAVTGKEVDMLHTGSSVATAGGAESGYSSSMEDVSSS + Oxidation (M)
5340	664.6310	1990.8712	1990.9557	-0.0846	0	9	2.2e+02	1	EAEMEFEGTVVEVLPNAK
6654	948.7620	2843.2642	2843.6524	-0.3882	1	9	1.3e+02	1	LLKLGDWELTVPLVVPTAIAAEINIR
7558	615.7743	3688.6021	3689.7231	-1.1210	1	9	71	1	GEIAPEAAQKMMTAESMGVAPMTSDMLPPQNALTR + Oxidation (M)
7748	660.9435	3959.6173	3958.9775	0.6398	1	9	51	1	WGLAPMTGSLAPPPPYGSEQGSILLEAGTKDGFTSTLGR
4200	754.7947	1507.5748	1506.7314	0.8435	1	9	2.6e+02	1	ENEAAATEEFIKR
5290	652.8829	1955.6269	1955.9840	-0.3571	1	9	1.6e+02	1	AFLLEQFPSESSSTTGKK
5497	688.5938	2062.7596	2061.9612	0.7984	0	9	1.6e+02	1	IQTVSFAGGPMPQNSPMER + Oxidation (M)
5694	569.2308	2272.8941	2273.1692	-0.2751	1	9	1.5e+02	1	VLPPEKQSSGGSAELQYGWLK
6447	667.5859	2666.3145	2666.2729	0.0416	0	9	1.6e+02	1	QFLNYTLTGVGGFMAASMLMPMVR + 2 Oxidation (M)
6928	749.8835	2995.5049	2994.4467	1.0582	0	9	1.3e+02	1	TSTEGMISHFESVLPMGPTIIYNVPSR + 2 Oxidation (M)
7403	884.9092	3535.6077	3535.5456	0.0621	0	9	88	1	ATMAHANTSHPLYVAGMAGNTNHMSPWGEDAHK + 2 Oxidation (M)
8154	861.3998	4301.9626	4301.1638	0.7989	1	9	50	1	MEAEQRPAAGASEGATPGLEAVPPVAPPPATAASGPIPKSGPEPK + Oxidation (M)
8460	906.4016	4526.9716	4528.1334	-1.1618	1	9	41	1	MNISFEKFLECLSIFMHSFFVLLMVVMCGAYMSFVER + Oxidation (M)
3585	681.0013	1359.9880	1360.7939	-0.8059	1	9	2.4e+02	1	GAVVLPHGTGKVAR
3629	686.4684	1370.9222	1370.7340	0.1883	1	9	2.7e+02	1	HITMLSIKSGER
6419	667.5851	2666.3113	2667.3465	-1.0352	1	9	1.6e+02	1	MSSFVQELDKAFEGQLQVLLEEK
6638	941.7813	2822.3221	2821.5034	0.8186	1	9	1.5e+02	1	LSAVVAEITKADLLIMLSDIDGLYDK + Oxidation (M)
7908	682.9548	4091.6851	4092.8764	-1.1913	1	9	48	1	KMLTVFDPAMCCSTGVCGSDVDQVLVNFSADVQWLK + Oxidation (M)
4826	855.8090	1709.6034	1709.7059	-0.1025	0	9	2e+02	1	VGCVLAMDGCDPDTGK + Oxidation (M)
5659	741.0485	2220.1237	2220.0083	0.1153	0	9	2.1e+02	1	HFGSSDDSLSVAFNEIQPDR
6833	976.0736	2925.1990	2925.4906	-0.2916	1	9	1.1e+02	1	SITVSALPFLRENDLSIMHSPSASEPK
8491	755.8384	4528.9867	4529.9666	-0.9799	1	9	41	1	VASTMTDGANTMIEHDDTLPSQLGTMVINAEDEEEEGTMKR + 4 Oxidation (M)
3529	666.4118	1330.8090	1331.6867	-0.8777	1	9	3.4e+02	1	AADKGMEIVNIR + Oxidation (M)
6926	600.1082	2995.5046	2996.6153	-1.1106	1	9	1.3e+02	1	MSKLLVLFVVSAMALLQFSMVEATPQK + Oxidation (M)
7126	800.1187	3196.4457	3196.4481	-0.0024	1	9	1.1e+02	1	DGKYHLEGEGLSLTAQEWTDMMAAWVDK + Oxidation (M)
7417	1189.1975	3564.5707	3565.7215	-1.1508	1	9	86	1	AGITAVIQPGGSMRDSEVIAAADEADIAMVFTGMR + Oxidation (M)
8000	838.7992	4188.9596	4187.9462	1.0134	1	9	60	1	QCYIGNNCSLSIVSLNEIYNQKEYNHLINNSNMNK
8083	850.2059	4245.9931	4247.0919	-1.0988	1	9	56	1	VHEMLPCTILGAKPEPKVVTLGNDYDYVWDISAAAGSSK + Oxidation (M)
8427	753.6865	4516.0753	4517.1552	-1.0799	1	9	44	1	TLVEGANQYIGAGNMYNGDVEDLNKLHLYIMSQMEKPTTK + 2 Oxidation (M)
6999	603.2684	3011.3056	3011.6404	-0.3347	1	9	1.1e+02	1	TVVVIAHRLSTIENADEILVIDHGEIR

☒	7281	684.9152	3419.5396	3419.7065	-0.1669	1	9	92	1	MLIPTGSKCIDSLLGGGVETGTVTQIYGHGGTGK + Oxidation (M)
☒	7345	580.7711	3478.5829	3477.6135	0.9694	1	9	89	1	ADRSMFVQSLMLSMLCTEALDLNASDESLAK + 2 Oxidation (M)
☒	7989	1041.4509	4161.7745	4161.7997	-0.0252	1	9	47	1	QDMPIQQQHATGNMSRFVLYQLQNSNDCDCSTTSR + 2 Oxidation (M)
☒	6569	693.5679	2770.2425	2769.4443	0.7982	1	9	1.4e+02	1	VRLGLLLSEIGQQNGVEVSAQEMQR + Oxidation (M)
☒	6965	600.3096	2996.5116	2995.4307	1.0809	0	9	1.3e+02	1	HATGEAVFCDDMSVLPGELFLAVVTSSK + Oxidation (M)
☒	7386	590.1076	3534.6019	3534.6324	-0.0304	0	9	90	1	MIGILSDVDTSQGGAGAASLAMMGMGGGGAPRPPSGGR + 3 Oxidation (M)
☒	7843	808.5604	4037.7656	4037.8363	-0.0706	0	9	56	1	DASQSEVSGENEGNLPSPEEDGFTISLSHVAVPGSPDQR
☒	7874	1013.9686	4051.8453	4051.0720	0.7733	1	9	62	1	DYTPSTNSLVSMASKFSLGQTELILLLMCFILALSR + 2 Oxidation (M)
☒	2331	661.3737	660.3664	660.3806	-0.0142	0	9	25	1	TAALTGK
☒	2780	494.2999	986.5852	987.4257	-0.8405	0	9	4.5e+02	1	EHSSAAGDSK
☒	4543	806.7386	1611.4626	1611.7055	-0.2429	0	9	2e+02	1	MGCVSMTLGIDEAGR + Oxidation (M)
☒	5285	650.2767	1947.8083	1948.0339	-0.2257	1	9	2.2e+02	1	TFSDMLIPSGKTNSLPIK
☒	5337	995.9582	1989.9018	1988.9969	0.9049	1	9	2.4e+02	1	DQVFINHGWQSFRTVR
☒	6364	531.0680	2650.3036	2650.2638	0.0398	1	9	1.6e+02	1	NFYEAVQAQWFTQMFSRLEQK
☒	7382	884.6575	3534.6009	3533.7972	0.8037	1	9	90	1	RTAAIMYAGGADTTVSTIQSFVLAMMVYPEVLK
☒	7865	811.3754	4051.8406	4052.9807	-1.1401	1	9	61	1	GSTLWVMCVGRFLMGLVLGLVCVASPMYVNENAHPK + 3 Oxidation (M)
☒	3684	696.4361	1390.8576	1390.8660	-0.0083	1	9	3.1e+02	1	ITIGHVIAGAIAKK
☒	4051	368.9047	1471.5897	1471.6436	-0.0539	0	9	2.9e+02	1	FCGVEAAVSEMTR + Oxidation (M)
☒	5183	943.9808	1885.9470	1887.0213	-1.0743	1	9	2.5e+02	1	AETAPAAAPAAAPAPAAKAAAK
☒	6934	600.1086	2995.5066	2995.4599	0.0467	1	9	1.3e+02	1	SLNALGATPMDLMSILQSMQSAGCLRAK + 2 Oxidation (M)
☒	7870	811.3759	4051.8431	4051.0720	0.7711	1	9	62	1	DYTPSTNSLVSMASKFSLGQTELILLLMCFILALSR + 2 Oxidation (M)
☒	6452	667.5872	2666.3197	2667.3465	-1.0268	1	9	1.6e+02	1	MSSFVQELDKAFEGQLQVLLEEK
☒	6812	729.7930	2915.1429	2915.4919	-0.3490	1	9	1e+02	1	FMTNTMCNAISLLGLTISKSTKPVTR + 2 Oxidation (M)
☒	7896	814.3737	4066.8321	4066.8079	0.0243	1	9	59	1	MINMEMFASYTYTSMAFYFSRDDVALPGFAHFFK + 2 Oxidation (M)
☒	8017	839.0013	4189.9701	4190.0230	-0.0529	1	9	60	1	IWSIDSILRCMELESLTPEIPLPQDLQMPLCSMSR + 2 Oxidation (M)
☒	8496	907.0021	4529.9741	4530.3589	-0.3848	1	9	40	1	KEVMSILAPLGPVYQAETLSGHPLGMAAGFACLTELARPGLHK
☒	4048	736.5977	1471.1808	1471.7565	-0.5757	1	9	2.2e+02	1	QGRIDCILVGADR
☒	6590	561.0886	2800.4066	2801.3523	-0.9457	1	9	1.6e+02	1	FAVVFAAMGITFEEADYFISDFRR
☒	7089	1047.4656	3139.3750	3138.4492	0.9258	1	9	1.1e+02	1	NNEFLEMSSNLLDFSKDDQFLNDFIK + Oxidation (M)
☒	8718	795.4114	5560.8289	5559.7054	1.1235	1	9	22	1	IMDQLVQAWNVLATMTPPEYSAMRPHLGQSSGFQSYQYREIEFILGNK + Oxidation (M)
☒	4037	733.7158	1465.4170	1465.7201	-0.3031	1	9	2.2e+02	1	GEGIYVWDSQGKK
☒	5634	734.6620	2200.9642	2202.1566	-1.1924	1	9	2e+02	1	ALESGKPANIVEKMVTGSVEK + Oxidation (M)
☒	2740	476.2272	950.4398	950.4709	-0.0311	0	9	3.9e+02	1	EFQIEGTK
☒	4671	824.1149	1646.2152	1646.7206	-0.5053	1	9	2e+02	1	MDPDIENERIEDR + Oxidation (M)
☒	6800	727.8484	2907.3645	2908.4059	-1.0414	1	9	1.4e+02	1	NPISFMNGQKGQEVLMGSENVTEVIR + 2 Oxidation (M)
☒	6864	982.1298	2943.3676	2942.7547	0.6128	1	9	1.4e+02	1	MNTFLWILLVIIALLAGLVGGTFIARK
☒	7353	875.6580	3498.6029	3498.5807	0.0222	1	9	91	1	THQPEQYDIPTDMSPLMTIAASESADKFTDK + 2 Oxidation (M)
☒	7491	1210.6174	3628.8304	3628.7688	0.0616	1	9	92	1	RLLDAGVTVALATDCNPGSSYTSSMPFCIALAVR + Oxidation (M)
☒	8449	1130.0309	4516.0945	4515.1369	0.9576	1	9	45	1	DWALGTPEGDWVSMAVPSQGEYGIPEGIVYSFPVTAKDGAYR
☒	5750	579.7595	2315.0089	2316.0151	-1.0062	0	9	1.9e+02	1	QQCDDNGAVLIFDEVMSGFR + Oxidation (M)
☒	7240	1121.7985	3362.3737	3363.3931	-1.0194	0	9	77	1	ENTQETTGQPGQPGHQEETESGQAGHPMDDR + Oxidation (M)
☒	7358	878.9202	3511.6517	3511.8861	-0.2344	1	9	93	1	VVVGLSGGVDSSVAAALLHRQGYAVEGLTLWLMK + Oxidation (M)
☒	8187	872.8057	4358.9921	4360.1142	-1.1221	1	9	50	1	TAIPSWSANSSVAGSTSSSLCEHWVSRLPGSKPASVTSAASSR
☒	2396	355.0685	708.1224	707.3602	0.7622	0	9	20	1	ATGGGAFK
☒	4456	788.9083	1575.8020	1574.7788	1.0233	0	9	3.2e+02	1	SSSQSTLIPSNEPTK
☒	6154	639.7892	2555.1277	2555.0574	0.0703	1	9	1.6e+02	1	KMEEMDAEAGQDMDAEAGQSQLR + Oxidation (M)
☒	6752	719.3297	2873.2897	2872.3987	0.8910	0	9	1.4e+02	1	EVLANMGLSLGMSVSGWPPENIEDLAK + Oxidation (M)
☒	7093	630.5076	3147.5016	3148.5977	-1.0961	1	9	1.2e+02	1	LTIDQVVCSPVFNFLFFSGMGILEGKSK + Oxidation (M)
☒	8142	860.7875	4298.9011	4299.9583	-1.0572	1	9	47	1	DGEELPATCQSSCGHMHALVLPGVTREDAGEVTFSLGNSR + Oxidation (M)
☒	2986	523.2358	1044.4570	1045.5556	-1.0986	0	9	4.4e+02	1	LAFTPNIDR
☒	5546	1059.0149	2116.0152	2117.1593	-1.1440	1	9	2.3e+02	1	TAPPPATAAAAATAAAKPSTPRK
☒	6207	863.7150	2588.1232	2588.3632	-0.2401	1	9	1.6e+02	1	EKVLHLTQQEVPHSVAVVVDSMK + Oxidation (M)
☒	6279	654.0443	2612.1481	2613.2823	-1.1342	1	9	1.5e+02	1	AIEALPPGNFKVSFPNGAEGSGPDSR
☒	7627	945.1810	3776.6949	3776.9117	-0.2168	1	9	74	1	MLQAESYGADIAKVAVMPASPQDLLVLFQATQEAR + Oxidation (M)
☒	8366	748.8318	4486.9471	4488.1069	-1.1598	1	9	40	1	MFTVLLDSNASEHAARTMAMQIATDNANDLLQDLTVMYNK + 2 Oxidation (M)
☒	5204	950.8555	1899.6964	1899.1413	0.5551	1	9	1.9e+02	1	KLLLIVGLLLACSTVMR
☒	7149	1084.8201	3251.4385	3252.5746	-1.1361	1	9	1e+02	1	VTAPASATVTSAAGTSAAISREESGSEDQFLNK
☒	7934	822.7811	4108.8691	4109.7399	-0.8708	0	9	58	1	YDQFGHAGVGSSAASGGGQYGAGASDFSDIFSAFNDMFGGGK + Oxidation (M)
☒	8232	882.6164	4408.0456	4408.7203	-0.6747	1	9	50	1	NDIENIDVVKNATETHGDNTTTTNNNDDGDDDDDDDDDK
☒	8236	631.2798	4411.9077	4411.3800	0.5277	1	9	43	1	LKVVEAFIESGNRPEWMIMEVIPVIPPELRPLVPLDGGR + Oxidation (M)
☒	8680	998.8705	4989.3161	4989.3612	-0.0451	1	9	33	1	LLIEACLELMNTDAIVAIQDMGAAGLTSSCFEMASKGGMGVDLALDR + 2 Oxidation (M)
☒	8740	957.2885	5737.6873	5737.8933	-0.2060	1	9	21	1	ALLMSSTATMVKAFDEAINDGVDVLSISLASAAPFRPIDSITGDLELGSFHAVMK + Oxidation (M)
☒	4372	780.1890	1558.3634	1558.7086	-0.3452	0	9	2.1e+02	1	EAFAGTLMVGGGYDR + Oxidation (M)
☒	4468	793.1318	1584.2490	1583.7614	0.4877	0	9	2e+02	1	QDISPHITNVGEMK + Oxidation (M)
☒	6321	658.0439	2628.1465	2628.3581	-0.2116	1	9	1.5e+02	1	FIPVVDRITPDIEAMAGAAASELSR
☒	7478	904.2196	3612.8493	3613.8563	-1.0070	1	9	90	1	SDINHVLVIGSGPIVIGQAAEFDYSGTQACRVLK
☒	7801	801.3364	4001.6456	4000.8383	0.8073	0	9	51	1	INSGAAPAATPDNSSGGGASNAAPDAAAGAAAQQPAGSSQPGDPAK
☒	3417	646.0242	1290.0338	1289.7091	0.3247	1	9	2.5e+02	1	LLAGAYRIEER
☒	6105	636.2932	2541.1437	2542.3279	-1.1842	1	9	1.7e+02	1	QTGEAVGKQLGIGEVYAELLPQDK
☒	6922	600.1080	2995.5036	2994.4977	1.0060	1	9	1.4e+02	1	MKSVFTLSASLAISLLLCCTAQANDHK + Oxidation (M)
☒	7378	707.9259	3534.5931	3535.7565	-1.1634	0	9	91	1	FSFPNTHLYAAVLTAYSSSLPLHASSAFSFFR
☒	7679	1273.6373	3817.8901	3818.7042	-0.8141	1	9	79	1	NGKGMDAATADYIGMLGTVMNAMALQDAMEAAGQPTR + 3 Oxidation (M)
☒	7909	1024.2166	4092.8373	4093.9725	-1.1352	1	9	58	1	GMISTQPGSTPLASFKILALESADGLGGCSAGNDIGPYGER
☒	8404	753.5215	4515.0853	4514.1644	0.9210	1	9	47	1	SGQMMLAQGLLLHLMPTDWRWSDCHALTDVDFEVLKPR + 3 Oxidation (M)
☒	8512	650.2887	4544.9700	4544.2113	0.7587	1	9	40	1	FYQALDDRLYCVYQSMVLFFFENYTGVQILLYGDLPK + Oxidation (M)
☒	8557	920.5831	4597.8791	4597.1341	0.7450	1	9	32	1	GFPPPGMAGPRGFPMDGPPGLGPMPGFAGPNQAPPFGMGPPTQHSR + 2 Oxidation (M)
☒	4347	775.1074	1548.2002	1548.7566	-0.5563	1	9	2.1e+02	1	NLDTGGNKSVLMER + Oxidation (M)
☒	4366	778.8320	1555.6494	1555.8722	-0.2228	0	9	2.9e+02	1	LGSVFPGVEAGLAALR
☒	4996	604.3385	1809.9937	1809.8645	0.1291	0	9	2.6e+02	1	GFAAEGQSQNNYLNGLK
☒	5291	979.1970	1956.3794	1955.9914	0.3880	1	9	1.6e+02	1	MGVPKLDAYVIYLDTDK + Oxidation (M)
☒	6254	653.5781	2610.2833	2610.2813	0.0020	1	9	1.7e+02	1	ITSSNRSLDNTWIDEVYIDEIK
☒	6393	889.6961	2666.0665	2666.2534	-0.1869	0	9	1.3e+02	1	LTTDNPHGEYYLTDMAAILGGASEK
☒	6951	600.1107	2995.5171	2995.3991	0.1181	0	9	1.4e+02	1	GHTPMNDVTMLACIVSFISSSFLHSGR + 2 Oxidation (M)
☒	7605	625.2762	3745.6135	3745.7056	-0.0920	0	9	68	1	CPDGGDNADSSNTALNMPVIPMNTIAEAVIEMINR + Oxidation (M)
☒	7311	1152.5521	3454.6345	3453.6697	0.9648	1	9	99	1	HGRVDDSPYGGGAGMLLMPQPIFDAIADLPEAK + Oxidation (M)
☒	7338	696.7209	3478.5681	3477.6973	0.8708	1	9	91	1	NSSSYPQKPALIDSDTNETLSFSQFKSMVIK + Oxidation (M)
☒	7633	954.9815	3815.8969	3815.9967	-0.0998	1	9	79	1	IMLSPHPYALRSHLCVGEETASLGVYLHSKPVVR
☒	7789	798.3325	3986.6261	3985.8674	0.7587	0	9	52	1	VSDADVEDVIATVEDDPADMQPRPPVITVMGHVDHGK + 2 Oxidation (M)
☒	3558	674.3307	1346.6468	1346.6686	-0.0218	0	9	3.6e+02	1	CPITGLAIGGMNK + Oxidation (M)
☒	3641	689.3009	1376.5872	1377.7100	-1.1227	0	9	3.4e+02	1	GATTSVTSSAAGQLK
☒	3659	692.2930	1382.5714	1382.6904	-0.1189	0	9	3.2e+02	1	SHLLESYYMLK
☒	3660	692.2930	1382.5714	1382.6904	-0.1189	0	9	3.2e+02	1	SHLLESYYMLK
☒	5039	914.1940	1826.3734	1826.8469	-0.4734	0	9	1.8e+02	1	NESMLTFETSQLQGAR + Oxidation (M)
☒	5874	815.0628	2442.1666	2442.2902	-0.1237	1	9	1.9e+02	1	IYVGSIFAAPGKVVDMITSMSIK + Oxidation (M)
☒	5475	514.4706	2053.8533	2052.8517	1.0016	1	9	2.1e+02	1	STMKSSDMPWPGDEGEQR + Oxidation (M)
☒	7434	898.6663	3590.6361	3589.6231	1.0130	1	9	88	1	MGPLDQVMDMIPGLGGGIKDQLPDDAMDMTQER + Oxidation (M)
☒	7729	984.9908	3935.9341	3934.7612	1.1729	1	9	74	1	VTYEGGKAMEGEPSEGVPAVEEEAGGAVDDDVPMAAVEK + Oxidation (M)
☒	3715	703.0217	1404.0288	1403.8244	0.2045	1	9	2.4e+02	1	LSLALALLGKMMK + Oxidation (M)
☒	4079	739.0365	1476.0584	1476.7824	-0.7240	0	9	2.3e+02	1	DTINLTVAAAFVDK
☒	6152	639.7892	2555.1277	2554.3616	0.7661	1	9	1.6e+02	1	AAIRASSNAIIVLHNHPSGDVTPSK

☒	6613	939.4277	2815.2613	2816.3217	-1.0604	1	9	1.4e+02	1	MAMPDLTHDLTGKLLIAMPGMEDPR + 4 Oxidation (M)
☒	7076	1040.8020	3119.3842	3120.4025	-1.0183	1	9	1.1e+02	1	GAFMLEPEGMSPMEPAGVSPMPGTQKDTGR + Oxidation (M)
☒	7145	1084.8164	3251.4274	3251.4662	-0.0388	1	9	1e+02	1	DSKPSSTVSSSSLSFNSSSKDNSPSTNSSTSR
☒	7151	814.1152	3252.4317	3251.5923	0.8394	1	9	1e+02	1	ATADYMGMLATVMNALALQDAMRHIGLVSR + 2 Oxidation (M)
☒	7724	785.3516	3921.7216	3922.8791	-1.1575	0	9	66	1	MDAVASLLGNASGIPPPCELGLDNETLFCLDQPPPSK
☒	8237	883.3903	4411.9151	4412.1706	-0.2555	1	9	44	1	NMQLKFVANPNVIEDPAAEPVDAPNVADLPAGTSAPAAEQNAR
☒	8732	1123.7554	5613.7406	5613.5620	0.1787	1	9	26	1	VTNLMTGQTANATSLLGTMTDAFPMLHFDLCDLIGDDWDETGLECRTPGGR
☒	4479	795.3668	1588.7190	1587.8216	0.8975	1	9	3.1e+02	1	STSQKNEALAAIAER
☒	7109	638.1392	3185.6596	3185.5009	0.1587	1	9	1.2e+02	1	EALEFFSDMIAAGHKSDEVSMTSIIAASGR + Oxidation (M)
☒	7663	764.3878	3816.9026	3817.8875	-0.9849	1	9	79	1	VWCAVDVGMVAQPDIVKAQMEGGIIQGLSVALMER + 3 Oxidation (M)
☒	7742	987.2416	3944.9373	3945.8116	-0.8743	1	9	74	1	DVMNAALAVGGTSFDSLYVNVNGESGYFDRSLDAYGR + Oxidation (M)
☒	7743	658.6110	3945.6223	3945.7271	-0.1047	1	9	55	1	NEYDLEQSVDDLCGVAEWDPILEELKEMGFCDK
☒	4865	864.7770	1727.5394	1726.9213	0.6181	1	9	1.9e+02	1	LELEGDEAIGASIVRR
☒	5307	984.4323	1966.8500	1967.8870	-1.0369	1	9	2.3e+02	1	GWDTCPMIGFDAEAVKR + Oxidation (M)
☒	5567	714.3666	2140.0780	2140.1780	-0.1000	1	9	2.2e+02	1	STWVTPKTLPVEEATALLGK
☒	7115	1065.4746	3193.4020	3194.5152	-1.1132	1	9	1.1e+02	1	TKDSSDDALVAVIMAIYFCNDQEVALYK + Oxidation (M)
☒	8365	748.8317	4486.9465	4485.9404	1.0061	1	9	41	1	VASTMSDGANTMIEHDDTLPSQLGTMVINADDEEEEGTMKR + 3 Oxidation (M)
☒	2304	644.2818	643.2745	642.3449	0.9296	0	9	22	1	GVGPASR
☒	5636	551.2489	2200.9665	2202.1275	-1.1610	1	9	2.1e+02	1	VLNMMPEEKLVEALAAATEK + Oxidation (M)
☒	5839	806.7495	2417.2267	2418.1936	-0.9670	0	9	1.9e+02	1	VERPIHIMNPSAFMSYVQQR + Oxidation (M)
☒	6763	719.3323	2873.3001	2872.3425	0.9576	1	9	1.4e+02	1	MEYAFWMDGAESLALNQAYRHVVR + Oxidation (M)
☒	6971	1000.7833	2999.3281	2999.3940	-0.0659	1	9	1.2e+02	1	CCTESLVNRRPCFSALTPDETYVPK
☒	7534	732.3386	3656.6566	3657.7742	-1.1176	1	9	81	1	QWSGTPVIAANMDSVGSFAMAKALSEHGVMTAIHK + Oxidation (M)
☒	3518	664.1079	1326.2012	1326.6490	-0.4477	0	9	2.3e+02	1	MGGEFSLTVSVGK + Oxidation (M)
☒	6669	952.4302	2854.2688	2853.4185	0.8503	1	9	1.4e+02	1	KSDEVNFLFNLINDAFIDLLNDER
☒	6765	719.3325	2873.3009	2874.4038	-1.1029	1	9	1.4e+02	1	GKLVCGEDVCIDVNVIIEGCVSLGNR
☒	7075	1040.7941	3119.3605	3119.5370	-0.1766	1	9	1.1e+02	1	ENTPIHHNESLNQLSKDLGSISLSEETK
☒	8065	1062.2552	4244.9917	4245.1371	-0.1454	1	9	60	1	AIVIVALDNLTKGSSGQALQNANLMLGEDETAGLMMAPLFP + 2 Oxidation (M)
☒	8445	904.2252	4516.0896	4517.2326	-1.1429	1	9	47	1	LQRALTQFMLDSHTDAGYEEVYVPFLVNQDSLHGTGQLPK
☒	4794	847.1564	1692.2982	1691.8586	0.4397	1	9	2e+02	1	EAELKMGSSLGLCLGK
☒	5504	689.7949	2066.3629	2067.1762	-0.8133	0	9	1.6e+02	1	QVLIMLPEIALSTQIINR + Oxidation (M)
☒	7827	1005.9235	4019.6649	4020.0199	-0.3550	1	9	54	1	MLLNFCSIFGLSSESLAFKGLTHLSELLIACQNYK + Oxidation (M)
☒	4464	790.8547	1579.6948	1579.8432	-0.1484	0	9	3e+02	1	LAPIAAFGAMAFTVGK + Oxidation (M)
☒	5732	575.7664	2299.0365	2299.1453	-0.1088	0	9	2e+02	1	QFHIHAQSAVLSDSAVMAMLK + Oxidation (M)
☒	6818	584.4357	2917.1421	2916.4060	0.7361	1	9	1.1e+02	1	VWEGGMAFHGGFLGVVVAMMLYARMR + 2 Oxidation (M)
☒	6829	731.8643	2923.4281	2924.4829	-1.0548	0	9	1.5e+02	1	TNELLGPILLMIHNLHHYMAFFER + Oxidation (M)
☒	7412	1183.8101	3548.4085	3547.5990	0.8095	1	9	75	1	YAPESNPENNPDMDAAADFAVRYFNDFVKPK + Oxidation (M)
☒	8112	853.3802	4261.8646	4261.1187	0.7459	1	9	51	1	NWGRVPALGTEPMFISDLADAVVESLPYVGAMAVSNLEAR + Oxidation (M)
☒	2034	574.2537	573.2464	573.2758	-0.0294	0	9	1.7	1	AAAAAEA
☒	7661	764.3875	3816.9011	3816.7434	0.1577	1	9	80	1	LLCGGEALKGDGFDNGAWVAPTVFTDCSDEMTIVR + Oxidation (M)
☒	7869	811.3758	4051.8426	4052.8821	-1.0395	0	9	65	1	CPEQEVSVNHLFTVASCSYFVFAMLGGIMLDFAGPK + 2 Oxidation (M)
☒	8398	753.5206	4515.0799	4514.1644	0.9156	1	9	48	1	SGQMMLAQGLLLHLMPTDWRWSDCHALTDVDFEVLKPR + 3 Oxidation (M)
☒	2620	439.4918	876.9690	876.4851	0.4839	1	9	3.3e+02	1	SVMKSLGR
☒	6136	639.5391	2554.1273	2554.1910	-0.0637	1	9	1.6e+02	1	DAAHFDMEIVHNADLWELEKR + Oxidation (M)
☒	6211	649.5424	2594.1405	2594.1039	0.0366	0	9	1.6e+02	1	DMGGPSSTSDPATQATQSEDTSATPR
☒	6843	976.7917	2927.3533	2928.2866	-0.9333	1	9	1.4e+02	1	DDIQQRLTDSFDPTNPISMMSDSGAR + 2 Oxidation (M)
☒	7387	884.6579	3534.6025	3535.7891	-1.1866	1	9	95	1	ACFAEDMVFLRQVGLHPVVVHGGGPQISQMLK + Oxidation (M)
☒	5317	991.1609	1980.3072	1980.0779	0.2294	0	9	1.7e+02	1	VADNDLIILDQLTLEAPK
☒	5588	722.3650	2164.0732	2165.0173	-0.9441	1	9	2.2e+02	1	DKVIFMVDMESFFASVER + Oxidation (M)
☒	6160	852.7173	2555.1301	2554.2432	0.8869	1	9	1.7e+02	1	NIMTTSSNLKESDSGSILENVATK + Oxidation (M)
☒	7326	696.5184	3477.5556	3476.5889	0.9667	1	9	93	1	EIETTLRQNAGLIAEQFLDDGGPEEDAAGAAEM + Oxidation (M)
☒	7421	892.6551	3566.5913	3565.6783	0.9130	0	9	89	1	AQLGDQSEYAFQQEQLGTAHAVMQAAPYLQEK + Oxidation (M)
☒	7482	724.3257	3616.5921	3615.5854	1.0067	1	9	84	1	QMPEEAAMQMIHAMEMMKNGSMWIVSMGQLK + 3 Oxidation (M)
☒	7740	789.9370	3944.6486	3943.7952	0.8534	1	9	57	1	TYMNGKPLFESSICGDSAADVSQSEENGQKPENKAR
☒	8923	1225.7880	6123.9036	6124.1993	-0.2957	1	9	18	1	VHGSALGFGMAVLSITVGSFVGVGFSIVTQNLICLLTFQLKLHYDGISSQLVSLDCR
☒	3554	673.0491	1344.0836	1343.6616	0.4221	1	9	2.5e+02	1	RGVQAMLPDSDR
☒	5424	1016.9595	2031.9044	2033.0225	-1.1180	1	9	2.4e+02	1	HAQMRGPQAAVAGTVNGQPK + Oxidation (M)
☒	6594	562.4519	2807.2231	2807.4786	-0.2555	1	9	1.4e+02	1	LTSVEVAHITMPSLTAAHIGTMTVRR + Oxidation (M)
☒	7693	771.9327	3854.6271	3853.9532	0.6739	1	9	64	1	KSSSSSSTTTTTTPTTPPTTTTTTSSSINALSAIPIPK
☒	8199	873.4213	4362.0701	4363.0130	-0.9428	1	9	55	1	ENLNDVMAELGMTEGFDVGLEMSGAPPAFRALLNSMNHGGR + Oxidation (M)
☒	3068	553.9505	1105.8864	1106.6335	-0.7471	1	9	2.8e+02	1	KGYTVIGLEK
☒	3399	643.2660	1284.5174	1284.6748	-0.1573	0	9	3.6e+02	1	MAQVVADPIVDK
☒	6905	1493.1884	2984.3622	2984.4987	-0.1365	0	9	1.3e+02	1	QVLVAILNDTPDIHVMGAASDPIFAMSK + 2 Oxidation (M)
☒	7958	1032.4738	4125.8661	4126.0032	-0.1371	1	9	58	1	GLVGSSGASHGDKVYILGGSNLSIFNGFFQDNVAAGEDQAK
☒	8255	736.6620	4413.9283	4414.2026	-0.2743	1	9	47	1	MGMSWWLACAAAFSALCVLKASSLDTFLAAVYEHAVILPK + Oxidation (M)
☒	2445	368.1874	734.3602	734.3381	0.0221	0	9	90	1	AEGGVMR + Oxidation (M)
☒	2456	745.8085	744.8012	745.3719	-0.5706	0	9	1.6e+02	1	QDIASGR
☒	4461	790.1525	1578.2904	1578.7348	-0.4443	1	9	2.1e+02	1	GERIMADGEFTEPK
☒	6915	998.7798	2993.3176	2992.4600	0.8575	1	9	1.3e+02	1	VDLLTRQVSAPGVDWSYGEPTEMLSSR
☒	7583	621.6486	3723.8479	3723.9472	-0.0993	1	9	90	1	RNPTVITLYELAQALGVSHVELVTPTDETPFGTR
☒	7660	955.2325	3816.9009	3817.7941	-0.8932	1	9	81	1	TVSGNHNLINICIDGEFKQIMYDPNHNELFNR + Oxidation (M)
☒	8719	1113.1735	5560.8311	5559.7452	1.0859	1	9	23	1	MTSAVGTSGTAITSRVHSLNRPNMVSVGTIVWLSSELMFFAGLFAMYFTAR + 3 Oxidation (M)
☒	2399	355.0686	708.1226	707.3602	0.7624	0	9	21	1	ATGGGAFK
☒	2960	511.5952	1021.1758	1021.5226	-0.3468	0	9	2.9e+02	1	SQVVSSLMR + Oxidation (M)
☒	6025	838.0975	2511.2707	2510.2278	1.0429	1	9	1.9e+02	1	NFIHRGYVGDIQTGPFCGFGLR
☒	6380	889.3605	2665.0597	2665.5108	-0.4511	0	9	1.3e+02	1	SYGLALLLFLATLLAWQPAHAQLR
☒	6677	952.7651	2855.2735	2854.3767	0.8968	1	9	1.4e+02	1	GTDIQLGGNAEMRISEELADLPAGPER + Oxidation (M)
☒	7669	955.2334	3816.9045	3815.9967	0.9078	1	9	81	1	IMLSPHPYALRSHLCVGEETASLGVYLHSKPVVR
☒	3162	580.8080	1159.6014	1158.7125	0.8890	1	9	4.2e+02	1	GLGIKVFIGQK
☒	4028	730.4714	1458.9282	1458.8235	0.1047	0	9	3.1e+02	1	FVGLVVYHILGDK
☒	4058	738.0256	1474.0366	1473.7212	0.3155	0	9	2.4e+02	1	DPTDLAFAGAAAQAR
☒	5849	811.3643	2431.0711	2432.2581	-1.1870	0	9	1.8e+02	1	TSLVSALIDTTSNIMVSVSHTTR
☒	6259	653.5809	2610.2945	2611.1432	-0.8487	0	9	1.8e+02	1	GADMGASFGAGASATLFGSSGSGNFMTR
☒	7629	757.3619	3781.7731	3780.8754	0.8977	1	9	84	1	ALANMASHQGHERLVDLGNWVAPTPDLEGAQQALR + Oxidation (M)
☒	7655	764.3869	3816.8981	3816.7434	0.1547	1	9	81	1	LLCGGEALKGDGFDNGAWVAPTVFTDCSDEMTIVR + Oxidation (M)
☒	8105	852.9778	4259.8526	4258.9078	0.9449	1	9	50	1	TDETAQGAAASSSVAGAVGMTTSGESESDDSEMGRLQALLEAR + Oxidation (M)
☒	3679	695.0086	1388.0026	1388.7486	-0.7459	1	9	2.5e+02	1	VSDYKVLMGHLK
☒	4495	797.3675	1592.7204	1592.8271	-0.1066	0	9	3.1e+02	1	AGHAIETADLGGGLGVR
☒	5683	1128.2560	2254.4974	2254.0463	0.4511	0	9	1.5e+02	1	MYNSIPSEAVSEEVVDWATK
☒	6287	654.0445	2612.1489	2611.3255	0.8234	1	9	1.6e+02	1	RPQLIHFLETHESHERAGPTEK
☒	7129	1071.4641	3211.3705	3211.4590	-0.0885	1	9	1e+02	1	AYNLFMKNETMYADNHSTEDIYAIGLR + 2 Oxidation (M)
☒	8173	871.9841	4354.8841	4355.0052	-0.1211	1	9	45	1	EDDQVVILSDIQGMEDALGDMDLKVAGTEEGITAMQMDIK + 2 Oxidation (M)
☒	8482	906.7996	4528.9616	4527.9874	0.9743	1	9	43	1	VASTMTDGANTMIEHDDTLPSQLGTMVINTEDEEEEGTMKR + 2 Oxidation (M)
☒	4047	736.5142	1471.0138	1470.7143	0.2995	0	9	2.5e+02	1	TVADTFIAEYWR
☒	5268	648.9338	1943.7796	1943.9371	-0.1575	0	9	2.2e+02	1	GGEVSAVVLNSGGANACTGPK
☒	6850	735.6277	2938.4817	2938.4092	0.0725	0	9	1.5e+02	1	LADDMFETMYDAPGIGLAAIQIAEPVR + 2 Oxidation (M)
☒	2668	906.4045	905.3972	906.4811	-1.0839	0	9	5.1e+02	1	AATPSVSFK
☒	7241	841.8502	3363.3717	3362.4926	0.8791	1	9	83	1	QRMDDQHLGSMNEVLTQTPGITMSQDGGER + 2 Oxidation (M)
☒	7726	654.7938	3922.7191	3922.0678	0.6513	0	9	68	1	RPAMVASLGLVVIVVCYIGVNLLGIGLHSYGWFFDA + Oxidation (M)
☒	7910	819.9288	4094.6076	4094.0315	0.5761	1	9	49	1	MLDETAQERNQPIFPALIYSSAMIWTVGMAKPDTAAK
☒	8004	838.8008	4188.9676	4190.0230	-1.0554	1	9	65	1	IWSIDSILRCMELESLTPEIPLPQDLQMPLCSMSR + 2 Oxidation (M)

☒	8448	904.2260	4516.0936	4515.9988	0.0948	1	9	48	1	ACAQGTFKPLSGEGSCQPCPANSHSNTIGSAVCQCRVGYFR
☒	7041	763.3634	3049.4245	3050.4522	-1.0277	1	9	1.4e+02	1	FGTENLNSSAAGFDPDKLEWLNGHYLR
☒	7150	814.1137	3252.4257	3253.5363	-1.1106	1	9	1.1e+02	1	QAPGQGLEWMGWISAYNGNTNYAQKLQGR + Oxidation (M)
☒	8316	639.5716	4469.9503	4470.2430	-0.2927	1	9	45	1	LADSVILYLNAQIANGAQSVMIFDSWGGALSHSAYREFSLR
☒	5264	969.4655	1936.9164	1937.9604	-1.0440	1	9	2.6e+02	1	LLTGCVGGCVWYLERR
☒	7727	786.1528	3925.7276	3926.7395	-1.0119	0	9	64	1	QSSVEQHSSVQPENVQGANTSGSDLSSLQMQDCQHK
☒	8574	771.6639	4623.9397	4624.2326	-0.2929	1	9	35	1	MQLNVNDAQAIEVSELTFGGEFNETLVHQAVVAYMAGGRQGGSK + Oxidation (M)
☒	4514	798.9280	1595.8414	1594.7297	1.1117	1	9	3.2e+02	1	GERIMADGEFTEPK + Oxidation (M)
☒	4573	542.2031	1623.5875	1623.8944	-0.3069	0	9	2.4e+02	1	SHLATVNLSSLLDVR
☒	4952	595.2722	1782.7948	1783.8887	-1.0939	1	9	2.8e+02	1	EITIAAKMGGGDPTGNPR
☒	5510	1037.4661	2072.9176	2073.0742	-0.1566	0	9	2.3e+02	1	NISLSFDEGILAVVGPNGSGK
☒	5805	797.4193	2389.2361	2390.1835	-0.9474	1	9	2.1e+02	1	GDRPFVMCNLKEQAGLDAVVR + Oxidation (M)
☒	6528	684.0494	2732.1685	2732.0297	0.1388	0	9	1.4e+02	1	SCPEAPTSDTASDSEASDSDAMDQSR + Oxidation (M)
☒	7667	955.2333	3816.9041	3815.9967	0.9074	1	9	82	1	IMLSPHPYALRSHLCVGEETASLGVYLHSKPVVR
☒	8523	761.6589	4563.9097	4564.1207	-0.2110	0	9	37	1	NEEEEMLNSDASEAMLTVSSVPSPTLTENESVNESVVPNILK + 2 Oxidation (M)
☒	3993	724.1548	1446.2950	1445.6391	0.6559	1	9	2.4e+02	1	RSSYSCIQMNGK + Oxidation (M)
☒	5431	1019.1558	2036.2970	2037.0751	-0.7780	1	9	1.8e+02	1	TMELNLKGSFLMAQIIGR + Oxidation (M)
☒	5815	802.7512	2405.2318	2405.2260	0.0057	1	9	2.1e+02	1	NLLRMTFEETLPLQNDTINK + Oxidation (M)
☒	6851	588.7038	2938.4826	2938.4092	0.0734	0	9	1.5e+02	1	LADDMFETMYDAPGIGLAAIQIAEPVR + 2 Oxidation (M)
☒	7190	1104.1544	3309.4414	3309.5719	-0.1306	1	9	1e+02	1	VMIHQPASSFYEGQAGECMIEAEEVLKLK + Oxidation (M)
☒	7320	1154.8223	3461.4451	3461.6985	-0.2534	1	9	84	1	TADSGTMGLGDTKDGSNGADLVLPVPVGTVVFEAR + Oxidation (M)
☒	7506	910.4729	3637.8625	3637.7168	0.1457	1	9	93	1	YFGDGFQYIPGNHGGGAYHHEFTDLQWLKVGK
☒	7978	830.3710	4146.8186	4147.8920	-1.0734	0	9	57	1	AQGIYTSISQEEMGLMMVMTMAFSDGEFNGSTLSILAR + 2 Oxidation (M)
☒	8290	892.6198	4458.0626	4458.1051	-0.0425	1	9	52	1	ELPLPMMNILNGGAHADNNVDIQEFMIMPAGARCFAEALR + 2 Oxidation (M)
☒	3203	389.5605	1165.6597	1165.5406	0.1191	1	9	3.8e+02	1	GPRGGMIMMGK + 2 Oxidation (M)
☒	3838	715.9330	1429.8514	1429.7599	0.0916	1	9	3.5e+02	1	VMPRGEGSDILIK + Oxidation (M)
☒	4572	812.5656	1623.1166	1622.8165	0.3001	1	9	2.2e+02	1	RSAQFGGAAASVTWSK
☒	7348	696.7242	3478.5846	3477.5684	1.0162	1	9	99	1	SLVFFRGYSASHFSGGQWNSGGACDSETEPIK
☒	8339	746.3312	4471.9435	4472.1856	-0.2421	1	9	44	1	LFNLVHTYLLWLVTEHEEELFSMMELKDMFMPLPTK + 3 Oxidation (M)
☒	4888	871.7045	1741.3944	1740.7811	0.6133	0	9	2e+02	1	MLNVFGVSASMPDDSR + Oxidation (M)
☒	6588	933.7571	2798.2495	2798.4095	-0.1600	1	9	1.6e+02	1	FLTGLGLGGAMPNTITMTSEYLPARR + 2 Oxidation (M)
☒	7487	908.1462	3628.5557	3628.5521	0.0036	1	9	83	1	GDAEETDSYQEGDNESGAGSRSPSPTASSGIEGGSIK
☒	7548	922.6569	3686.5985	3686.7902	-0.1917	1	9	77	1	TLNYGMGFVWSMNFLRGSNFIGLGYDDGTVVLK + Oxidation (M)
☒	7787	797.5580	3982.7536	3983.9059	-1.1523	1	9	61	1	DQNNIDPAISGATMLTGPPQTPTQPDVTGQETPKTYGK + Oxidation (M)
☒	8010	699.1702	4188.9775	4189.1479	-0.1704	1	9	67	1	ILGLKPTVIFADQIMYTGFAYAARSGASVGIDDMVIPEK + 2 Oxidation (M)
☒	8313	894.7955	4468.9411	4469.0140	-0.0729	1	9	45	1	DDLLCSACKELLVRPVVLNCGHVYCEGCVVDMAEESEK + Oxidation (M)
☒	6134	511.8326	2554.1266	2555.2802	-1.1536	1	9	1.7e+02	1	WMLDLNSAIQAAKSGGDTAPALPGR + Oxidation (M)
☒	8140	717.4894	4298.8927	4297.9215	0.9713	1	9	50	1	GEYFLASDISPLTRNCEEVYYVNSGEWGYFSQNEFK
☒	6037	839.0853	2514.2341	2514.2226	0.0114	1	9	1.9e+02	1	GHRFMHPEAFPVSNLQEYLAR + Oxidation (M)
☒	8085	1062.5059	4245.9945	4245.1371	0.8574	1	9	63	1	AIVIVALDNLTKGSSGQALQNANLMLGEDETAGLMMAPLFP + 2 Oxidation (M)
☒	8586	1159.7866	4635.1173	4634.9393	0.1780	0	9	42	1	GANPEKPYTMALDYGGSMEQLEAMIDSSEHCEQEVAYHCR + 2 Oxidation (M)
☒	5852	609.5635	2434.2249	2434.2461	-0.0212	1	9	2e+02	1	AMLEPMTVRTLPGVGPATGDHLR + Oxidation (M)
☒	6298	654.0449	2612.1505	2613.3050	-1.1545	1	9	1.7e+02	1	DGFLVEQVFSPHPYPASLKAHMK + Oxidation (M)
☒	6487	539.8425	2694.1761	2693.2464	0.9298	0	9	1.6e+02	1	ADGENVGLPEGQMGNSEVGHLNIGAGR + Oxidation (M)
☒	7452	599.7782	3592.6255	3591.6579	0.9677	1	9	98	1	SMHDQHPTMSFVDLNRSGVALMEIVSKPDMR + 4 Oxidation (M)
☒	8170	726.6552	4353.8875	4353.1879	0.6997	1	9	47	1	TASNLLLKILALDSQPNFSSGEVDGGYFFMPADDILIEPR
☒	5733	575.7786	2299.0853	2298.1889	0.8964	1	9	2.2e+02	1	LKDQGMVTVISITHDLNEAAK + Oxidation (M)
☒	5941	830.3675	2488.0807	2487.2281	0.8525	0	9	1.8e+02	1	NLDYVLLGLGDSNYSSYQTIPR
☒	6496	677.3257	2705.2737	2705.3218	-0.0481	1	9	1.7e+02	1	RAVEAGEMDFVVGAIAGLDVEDDALK + Oxidation (M)
☒	7220	834.0880	3332.3229	3331.7412	0.5817	1	9	87	1	FGNTVTHLEAVSPLSTLARGYSVTSAADGAVLK
☒	7438	898.9130	3591.6229	3591.8402	-0.2173	1	9	96	1	AVSAGKAVHIMGLLSDGGVHSHQDHLVAMAELAVR + Oxidation (M)
☒	8327	746.1654	4470.9487	4471.1671	-0.2183	1	9	46	1	MVIQVAPTHYELFLSPDVNQLRDHYQWFFFQVSNMR + Oxidation (M)
☒	8455	904.5950	4517.9386	4518.4261	-0.4875	1	9	41	1	GDALLVGLFQDGPEPALALAALDAAVAAVVLEVISEEEFKPKSR
☒	8587	928.0308	4635.1176	4635.2486	-0.1310	1	9	42	1	SNPGPGANGNNGPLRSVVAMSANTPQVMVVTSDGNFYVFNIDLSK
☒	6670	952.4445	2854.3117	2854.3708	-0.0592	0	9	1.5e+02	1	DQWIQTILWGGGDEIHLAVDSSVCR
☒	7009	604.6545	3018.2361	3018.4458	-0.2097	1	9	1.1e+02	1	TYGGNGATLEEDAFTVNLEKAEPAPEAPK
☒	8874	742.2135	5929.6498	5930.8425	-1.1928	0	9	18	1	ELIFTVAAETAEPLGDALLDLGALSVTVEDDAAGGYDENPLYEEPGLSPEVQAWDR
☒	2500	773.4678	772.4605	771.4854	0.9751	0	9	3.7e+02	1	LKPLSSK
☒	3992	724.0026	1445.9906	1446.7731	-0.7825	1	9	2.7e+02	1	WANGRLYDLIAR
☒	5411	674.6810	2021.0212	2021.0694	-0.0483	1	9	2.6e+02	1	VNFGSRKPAPQTTYQVTK
☒	8226	881.0133	4400.0301	4400.0679	-0.0378	0	9	54	1	VVYDELMSLLENMTNMCTLLMLEANSQPEPMIGPILDK + 3 Oxidation (M)
☒	2458	746.3437	745.3364	745.3718	-0.0354	1	9	2.5e+02	1	DAEKAGR
☒	4980	903.4010	1804.7874	1805.8717	-1.0842	0	9	2.9e+02	1	MSSSIPESQIALTDSPK + Oxidation (M)
☒	6543	688.3123	2749.2201	2750.2752	-1.0551	1	9	1.5e+02	1	GATLFDDRISNVTMTNPQPNDTMGR
☒	7139	1075.8025	3224.3857	3225.3714	-0.9857	1	9	1.1e+02	1	MPQLNGGGGDELGANDEMISFKDEGEQEDK + Oxidation (M)
☒	7807	801.5386	4002.6566	4002.7536	-0.0970	0	9	57	1	MAGNEWINGYLEAILDSGGAAGGGGGGGGGGGGGGGGGGGGGGGGVDPR + Oxidation (M)
☒	3720	704.1041	1406.1936	1406.7630	-0.5694	1	9	2.5e+02	1	RTETNPIVHVNK
☒	4931	590.1077	1767.3013	1766.9236	0.3776	1	9	2e+02	1	RLLTAFGSMGESELLK + Oxidation (M)
☒	7701	968.4639	3869.8265	3869.9305	-0.1040	1	9	84	1	LDEMGVERASVTLHVGAGTFKPIQGPVADHVMHAER + Oxidation (M)
☒	7276	852.3661	3405.4353	3405.6111	-0.1758	1	9	89	1	SLMKMGQMVTINQPLDQDTAMIVVEEMGHK + 2 Oxidation (M)
☒	8306	1115.7745	4459.0689	4460.2686	-1.1997	1	9	53	1	NSATLNLIYDGLVDASLDRGSFILALGGGVIGDMAGFAAASYLR + Oxidation (M)
☒	4362	778.0718	1554.1290	1554.7889	-0.6599	0	8	2.2e+02	1	AETLPEVEAELAQR
☒	5652	739.3256	2214.9550	2215.9700	-1.0151	0	8	2.2e+02	1	MQNLLDPTFNGMPGSEMYR + Oxidation (M)
☒	8308	892.8212	4459.0696	4459.2699	-0.2003	1	8	53	1	LDGSIPPALADLNSLGYLNISYNNLSGEIPFKGHLVTFDER
☒	8389	751.8291	4504.9309	4505.1465	-0.2156	0	8	43	1	QVETLMPDTQIEVECRPEEDVINIPSEEGGAADDVLVPSPK
☒	3621	684.5145	1367.0144	1366.6697	0.3448	1	8	2.5e+02	1	SSSPVLRMSTMR + Oxidation (M)
☒	4042	734.6871	1467.3596	1467.6599	-0.3002	0	8	2.3e+02	1	AIQYGHMTQTMR + 2 Oxidation (M)
☒	8107	853.1786	4260.8566	4260.0361	0.8205	1	8	54	1	TKSSYPSASFFMTARPYVTGQGTFDNSTVAGILEYEPPK + Oxidation (M)
☒	8116	712.4993	4268.9521	4268.9982	-0.0460	1	8	60	1	EGTDFAVLSDILGDEDHLGDMDFKVAGTVDGVTALQMDIK + 2 Oxidation (M)
☒	8470	906.6016	4527.9716	4528.1859	-0.2143	1	8	46	1	QTGSASSMVNGVVRLLTKPWDVVPMVTQMAMTDTTPFGQQR + 4 Oxidation (M)
☒	3714	702.8385	1403.6624	1403.6238	0.0386	0	8	3.8e+02	1	MLGEEEEAVPER + Oxidation (M)
☒	4018	728.8251	1455.6356	1456.7457	-1.1100	0	8	3.5e+02	1	VHTMSALVVTGNGR + Oxidation (M)
☒	6216	650.0252	2596.0717	2595.3890	0.6827	0	8	1.5e+02	1	SGVRPPIMNGPMHPRPLVALLDGR + Oxidation (M)
☒	6676	952.7646	2855.2720	2854.4573	0.8146	0	8	1.5e+02	1	VVAALGDSLTTAVGARPNNSSDLPTSWR
☒	6873	740.3547	2957.3897	2956.3801	1.0096	0	8	1.5e+02	1	LIDGTEYGEMSLDNQFIGDSHVFIEK
☒	7809	1001.6725	4002.6609	4001.9653	0.6955	1	8	58	1	TCFETLLQFSLLEDHANTNNNRLNANVLTAGAAGAGGK
☒	5477	1027.9724	2053.9302	2053.0813	0.8490	1	8	2.5e+02	1	RSMILPDMVGVTIAVHNGK + Oxidation (M)
☒	8208	876.7917	4378.9221	4379.1705	-0.2484	1	8	49	1	LLEEAEKAGATPVSGVGMLVYQGALAFELWTGEEAPVEVMR + 2 Oxidation (M)
☒	8505	909.8002	4543.9646	4544.1233	-0.1587	1	8	44	1	MPVGTLITDMDTGEVIADLTEHGQRVCLAEGGMGGWGNLHFK + 2 Oxidation (M)
☒	4872	867.4313	1732.8480	1731.8284	1.0197	0	8	3.1e+02	1	VTMTDIHAMALASAER + Oxidation (M)
☒	6960	750.1345	2996.5089	2995.3991	1.1098	0	8	1.5e+02	1	GHTPMNDVTMLACIVSFISSSFLHSGR + 2 Oxidation (M)
☒	7033	1015.7902	3044.3488	3044.5535	-0.2048	1	8	1.3e+02	1	ATEARPPPSPPLVNEKAAIGALCVVCGDR
☒	7637	764.1899	3815.9131	3816.7434	-0.8303	1	8	87	1	LLCGGEALKGDGFDNGAWVAPTVFTDCSDEMTIVR + Oxidation (M)
☒	4249	766.4115	1530.8084	1529.9141	0.8944	1	8	3.6e+02	1	TKILLNTASTVLTR
☒	5519	696.4512	2086.3318	2087.0656	-0.7338	0	8	1.8e+02	1	HICLIAGGTGITPMYQLAR + Oxidation (M)
☒	6755	719.3307	2873.2937	2872.4065	0.8872	0	8	1.5e+02	1	SQSVAELLYGFIHNMVEEVTGHEGVK
☒	7063	1035.4661	3103.3765	3102.4419	0.9345	0	8	1.2e+02	1	MATMTVEAAAAAAAHAVAEAEAIMAEAEAAAR + 2 Oxidation (M)
☒	8212	877.0195	4380.0611	4379.2359	0.8252	1	8	58	1	VSVFVEVEGAGDFLPPYAGNLDIMTAAATKVGEVIAGQITSAR + Oxidation (M)
☒	8483	755.8349	4528.9657	4529.2213	-0.2556	1	8	45	1	QPGPGGDMSALLAQAQQMQQKLMEAQQQLANAEVHGQAGGGLVK + Oxidation (M)
☒	2517	392.7476	783.4806	782.5014	0.9792	1	8	2.1e+02	1	GPKAIGLK
☒	5541	1056.8103	2111.6060	2111.1223	0.4838	0	8	1.7e+02	1	GDLHVTLTSAAGTSTVLLAER

☒	6384	667.2726	2665.0613	2666.2437	-1.1824	1	8	1.4e+02	1	CIGIPMQVMAIEPGYAVCAGRGER + 2 Oxidation (M)
☒	6493	540.8687	2699.3071	2699.3701	-0.0629	1	8	1.8e+02	1	RPSIPRSSSIISISSNVAEFMYSR + Oxidation (M)
☒	6831	732.1107	2924.4137	2925.4555	-1.0418	1	8	1.6e+02	1	KTLSPAGAHQWTPTNAKPENMVPDAHK
☒	7288	856.3986	3421.5653	3420.6044	0.9608	1	8	1.1e+02	1	SRSGPTAHLYFSQSYGAFTGESLAAEIEGTMR
☒	3489	657.1121	1312.2096	1312.6122	-0.4025	0	8	2.6e+02	1	QIFQMDSGFPK + Oxidation (M)
☒	5412	675.2589	2022.7549	2022.0795	0.6754	0	8	2e+02	1	VSVLCIWMSALFLGVGVR + Oxidation (M)
☒	6260	653.5818	2610.2981	2611.3767	-1.0786	1	8	1.9e+02	1	YIIFQLSNQMFLIVHLRMTGR + 2 Oxidation (M)
☒	4328	773.0001	1543.9856	1542.8882	1.0974	1	8	3.1e+02	1	IFSKISTLTLHQR
☒	6842	976.7887	2927.3443	2927.5314	-0.1871	1	8	1.5e+02	1	DKGWTGIVDPMTGKPVNLTTEELGLLK + Oxidation (M)
☒	7511	910.4737	3637.8657	3637.6838	0.1819	1	8	97	1	VSMDMISIDLGPESTDKVGDEALMWGAELPVER + 3 Oxidation (M)
☒	8472	906.6017	4527.9721	4528.1859	-0.2138	1	8	47	1	QTGSASSMVNGVVRLLTKPWDVVPMVTQMAMTDTTPFGQQR + 4 Oxidation (M)
☒	3358	632.0270	1262.0394	1262.6111	-0.5717	0	8	2.8e+02	1	AAMEAAGMVGIAR + Oxidation (M)
☒	6562	554.0361	2765.1441	2765.2546	-0.1105	1	8	1.4e+02	1	MQGRNVLQPMGWDAFGMPAENAAMK + Oxidation (M)
☒	6610	563.2854	2811.3906	2810.3399	1.0507	1	8	1.7e+02	1	ATSVGDEGGFAPDFADNEEPLKYLIR
☒	6808	971.4624	2911.3654	2912.3540	-0.9886	1	8	1.6e+02	1	AALAEITEMMAQLHGNMIKMENFQK + 4 Oxidation (M)
☒	8003	838.8007	4188.9671	4190.0230	-1.0559	1	8	69	1	IWSIDSILRCMELESLTPEIPLPQDLQMPLCSMSR + 2 Oxidation (M)
☒	8216	880.4035	4396.9811	4398.0527	-1.0716	0	8	52	1	DTFSSGSATGFLIGVGNVGEFLTPMDDPSTATFLSTDGGVTWK + Oxidation (M)
☒	2602	429.8595	857.7044	858.4923	-0.7878	1	8	4.5e+02	1	LGLSEGRK
☒	4509	798.8122	1595.6098	1596.7679	-1.1580	1	8	2.8e+02	1	DHGEGGTMVGAPLKGR + Oxidation (M)
☒	8592	928.4295	4637.1111	4638.2478	-1.1367	1	8	45	1	MTTMTTMTTEQLGMNNSVNDKLDVLLIGAGFTGLYQLYHLR + 3 Oxidation (M)
☒	4919	586.6052	1756.7938	1755.9367	0.8571	0	8	3.1e+02	1	GDILADGAATVGGQLTLGK
☒	5297	654.7941	1961.3605	1961.6756	-0.3152	0	8	1.9e+02	1	AEGGPAMPDMGGMGGMGGMGM + 4 Oxidation (M)
☒	6040	840.1108	2517.3106	2516.2511	1.0595	1	8	2e+02	1	NCIGQTFAMMEAKIILAMLVSK + 3 Oxidation (M)
☒	7128	803.5978	3210.3621	3211.5382	-1.1761	1	8	1.1e+02	1	EHASGYVSRDTSSSPTTGNTVQSVDLIFTR
☒	7753	793.1337	3960.6321	3961.1135	-0.4814	1	8	60	1	KDVLSIIDDLAEGPQILVGSSLGGWLMFHAAIARPQK + Oxidation (M)
☒	2548	802.3939	801.3866	802.4232	-1.0365	1	8	4.7e+02	1	ACGRALR
☒	3741	707.9283	1413.8420	1414.6762	-0.8342	0	8	3.8e+02	1	ENEPVLSMRPLD + Oxidation (M)
☒	7143	811.1110	3240.4149	3239.5663	0.8486	0	8	1.1e+02	1	GNYSYPLDPSWSTEEITTVLHFLSQVEK
☒	8037	845.1997	4220.9621	4220.9929	-0.0308	1	8	66	1	VMEAMSEGLGIAQADALSALVTAEGSDQVFRVNHYPPCR + 2 Oxidation (M)
☒	5866	814.3889	2440.1449	2440.0709	0.0740	0	8	2.1e+02	1	DGMVATEGWAETSSLPATEMAMR
☒	8071	1416.3356	4245.9850	4245.2528	0.7322	1	8	65	1	ELLDVLSVLSLMERNSPIVISPLLMNLTVENDFSTTVK + Oxidation (M)
☒	8416	753.5226	4515.0919	4514.0247	1.0673	1	8	53	1	MTMEGASGSSFGIDTILSSASSGSPGMMNGDFRPLGEARTADFR + Oxidation (M)
☒	3340	626.3591	1250.7036	1250.5853	0.1184	0	8	4e+02	1	ALEEFMPDGVK + Oxidation (M)
☒	7445	898.9203	3591.6521	3590.5799	1.0722	1	8	1e+02	1	LGSGKSPMQMQYGGSGHLGGGLPPYCSMSSNGYGR + Oxidation (M)
☒	8539	1143.2576	4569.0013	4568.3156	0.6857	1	8	44	1	ASGRTLEGVQGLLELGLQHSQNLAFVSMLNDIATPSPAAMPFR + 2 Oxidation (M)
☒	2643	445.1187	888.2228	888.4930	-0.2701	1	8	4.1e+02	1	LGFGRSPR
☒	3011	528.2490	1054.4834	1053.6183	0.8652	0	8	4.1e+02	1	VIPGGQSIGVK
☒	3576	679.0758	1356.1370	1356.6310	-0.4939	0	8	2.6e+02	1	DVEVNVWDPER
☒	5052	919.1978	1836.3810	1836.8564	-0.4753	0	8	2e+02	1	DQLLGPLSNAMGEAYDK + Oxidation (M)
☒	7719	780.3253	3896.5901	3896.0207	0.5694	1	8	64	1	LETLETWVQDGLQPDLTLLFDVPLETASERLAGAR
☒	8348	639.9998	4472.9477	4473.0584	-0.1107	1	8	47	1	QSSKMGLFTSTTSASSVFPSMTTQLDSSTMVNMPAPSETIPK + 4 Oxidation (M)
☒	8603	934.4221	4667.0741	4668.2566	-1.1825	1	8	42	1	GMTMQGMQRVPGVPPGLMPLQRPFMGPAGVITMGGGVGPSPNQQK + 5 Oxidation (M)
☒	8841	975.7812	5848.6435	5848.8856	-0.2421	1	8	20	1	AGSSLVGIDAGELAGVGKLNVIIMDSLDYATDDFDVLIDFTAPDASIVHLDWCVR
☒	3016	1058.5206	1057.5133	1058.5971	-1.0838	1	8	4.9e+02	1	EISEIVKNK
☒	3373	638.3401	1274.6656	1274.6037	0.0619	0	8	4.3e+02	1	MDSGSVAAERPR
☒	6684	715.1232	2856.4637	2855.3322	1.1315	0	8	1.7e+02	1	APESVAAESNIANDAAFEGQQAPQSPTR
☒	7365	879.1707	3512.6537	3512.8405	-0.1868	1	8	1.1e+02	1	VCLKLPGGSCMAALTVTLTVLSSPLALAGDTQPR + Oxidation (M)
☒	8515	1137.2521	4544.9793	4544.3665	0.6128	1	8	46	1	DESVKPLLLVAGGTGMSYTLSILKNSLAQGFNQPIYVYWGAK + Oxidation (M)
☒	8530	914.4082	4567.0046	4566.3767	0.6279	1	8	46	1	IVREHLDAMEHMGSPADLVEHFALPVPSLVIAELLGVPPPDR
☒	8650	793.0408	4752.2011	4753.2900	-1.0889	1	8	46	1	QDMPYLQDGTPVDMVFNPLGVPSRMNVGQIFECSLGLAGDLLK + 2 Oxidation (M)
☒	2394	355.0683	708.1220	708.3225	-0.2004	0	8	23	1	SSAGMTR
☒	3093	562.9876	1123.9606	1124.6230	-0.6623	1	8	2.6e+02	1	VPKIGTGYYK
☒	5900	824.6966	2471.0680	2470.2097	0.8583	1	8	1.9e+02	1	HALMDMSSQLEHQDFVVIARK + Oxidation (M)
☒	7468	601.9534	3605.6767	3605.8044	-0.1277	1	8	1e+02	1	MTQGSRLYFAVTVLMCAFVSINGVGLNDLLER + 2 Oxidation (M)
☒	5270	487.2198	1944.8501	1945.8993	-1.0492	0	8	2.7e+02	1	GPMGFPGPQGPPGSPGPPGDK + Oxidation (M)
☒	7374	884.4078	3533.6021	3533.8374	-0.2353	1	8	1e+02	1	MSNTILQNLPLGQKVGIAFSGGLDTSAALLWMR + 2 Oxidation (M)
☒	2444	368.1872	734.3598	735.3109	-0.9511	0	8	99	1	AVIGMDD + Oxidation (M)
☒	8266	738.9917	4427.9065	4428.9718	-1.0653	1	8	46	1	LIMEDIDRWNLPIYNFPFDEDEISDEDYETNMYLR + Oxidation (M)
☒	2597	426.6944	851.3742	850.3855	0.9888	0	8	3.7e+02	1	TLDAEMR + Oxidation (M)
☒	4730	840.5223	1679.0300	1677.8620	1.1680	1	8	2.8e+02	1	AHPGAALEQLEAGMRK
☒	5226	478.7409	1910.9345	1909.9940	0.9405	1	8	2.8e+02	1	AKYLAQIMVMGMQVVGR + Oxidation (M)
☒	7020	757.3281	3025.2833	3026.4156	-1.1323	1	8	1.3e+02	1	MGGAIEKQYMDVNSVPMIAHAMMVLAR + 4 Oxidation (M)
☒	8356	748.6628	4485.9331	4486.1205	-0.1873	1	8	45	1	MPIKEFHLQNVLSEAGLSQDEFIDLCILLGCDYCDSIK + Oxidation (M)
☒	8405	904.0244	4515.0856	4515.2058	-0.1202	1	8	53	1	LNQIGAGDQGLMFGYATNETEELMPLPIMLSHHLMQRIAK + 3 Oxidation (M)
☒	8667	691.7178	4834.9737	4835.1527	-0.1790	0	8	32	1	FGPTMMVGDGMNDAAALAAADVGVSMGISGSAAAMETSHATLMSSDVLR + 4 Oxidation (M)
☒	5651	739.1594	2214.4564	2214.2116	0.2448	0	8	1.7e+02	1	SIMSILMLGAPQGGEILVTIR + Oxidation (M)
☒	8334	746.1663	4470.9541	4470.1113	0.8429	1	8	49	1	ENNQNGGGFGGNSHTSHVVSTFAAVSALYVIGTEESYKTIDR
☒	4864	863.8917	1725.7688	1724.8944	0.8744	0	8	3.1e+02	1	EALLAISNLVDDEAPR
☒	8544	917.1840	4580.8836	4580.3510	0.5327	1	8	40	1	EDATLVTIVPPSGYAEEQKAAAAAAAGGAAPAAAAPAAGAAAPAAAAPAAAAK
☒	4054	736.8423	1471.6700	1470.7790	0.8910	1	8	3.8e+02	1	ENERVITDIGAVR
☒	7279	1140.5227	3418.5463	3419.5254	-0.9791	1	8	1.1e+02	1	AMRESDSCKPAIFAMSNPTLNAECTAADAFK + Oxidation (M)
☒	8128	1074.0126	4292.0213	4291.1518	0.8695	0	8	63	1	AEQGSQTPTLASHHFNLAAGPLPPSTSPAMAPMTPLPGGLGLR + Oxidation (M)
☒	8178	1090.2577	4357.0017	4356.0097	0.9920	1	8	60	1	GSGQVGTYGLNTFTNMEVQLIRQMEAEGVITQDDMHNPK + 3 Oxidation (M)
☒	8369	748.9972	4487.9395	4488.1790	-0.2395	1	8	46	1	GVSLIGEFNHWGGNDAPMRVLGSSGVWELFWPGFPPDGLYK
☒	2459	746.3439	745.3366	745.3970	-0.0604	1	8	2.6e+02	1	GEVKGEK
☒	4920	586.6053	1756.7941	1755.8146	0.9794	1	8	3.2e+02	1	FMLFYGMSSTSAMKR
☒	5305	655.3212	1962.9418	1964.0513	-1.1095	0	8	2.8e+02	1	ASVMVHLPALNLTNNINK + Oxidation (M)
☒	5308	985.2314	1968.4482	1967.8385	0.6098	1	8	1.9e+02	1	QESEEGEGDFWVDEKGK
☒	6578	697.0737	2784.2657	2783.4469	0.8188	0	8	1.7e+02	1	FSHPIMPYLTEQIWQTIAPLLNR + Oxidation (M)
☒	8377	898.7975	4488.9511	4489.3890	-0.4379	1	8	48	1	RVLLSSMVGHAATEVTIAGVLHEYSSIDGVQEDVVNILLNLK
☒	6645	947.0865	2838.2377	2839.3958	-1.1582	0	8	1.6e+02	1	YFIPCLLLPTMMLASGGGGETDIVER
☒	7639	955.2299	3816.8905	3815.9967	0.8938	1	8	90	1	IMLSPHPYALRSHLCVGEETASLGVYLHSKPVVR
☒	8100	850.4079	4247.0031	4247.0919	-0.0888	1	8	67	1	VHEMLPCTILGAKPEPKVVTLGNDYDYVWDISAAAGSSK + Oxidation (M)
☒	8325	746.1647	4470.9445	4471.3939	-0.4493	1	8	49	1	MLPYEMVIAVLVYLSPAQILNLNLPFAYQKSVLFASNSAK + Oxidation (M)
☒	6379	534.0192	2665.0596	2665.2914	-0.2318	1	8	1.4e+02	1	EVIAFPKTQSAACVMTQAPGMVDAK + Oxidation (M)
☒	7172	820.8611	3279.4153	3279.6003	-0.1850	0	8	1.1e+02	1	APLQEPEMAPVTATISDTLPITVEAADACPR + Oxidation (M)
☒	3051	1084.4955	1083.4882	1084.4648	-0.9766	0	8	4.3e+02	1	GSVDYWACK
☒	8271	1109.7725	4435.0609	4434.2047	0.8562	1	8	58	1	LQESLAMLLREDPSLHVTVDEDSGQTLLSGMGELHLEIAR + 2 Oxidation (M)
☒	8457	906.3978	4526.9526	4526.2067	0.7460	1	8	48	1	QTGSASSMVNGVVRLLTKPWDVIPMVTQMAMTDTTPFGQQR + 3 Oxidation (M)
☒	8570	661.4276	4622.9423	4623.3582	-0.4159	1	8	39	1	MFTMFSSFSMASLALPGMSGFVAELIVFFGIITGQKYVLIPK + 3 Oxidation (M)
☒	8666	806.8336	4834.9579	4835.3653	-0.4073	0	8	32	1	TAMLIALMTVMFMSIGYLLGGGGGMMIALVIAVAMNLFGYWNSDK + 4 Oxidation (M)
☒	4039	734.2871	1466.5596	1467.6698	-1.1101	1	8	3.2e+02	1	MDTKGVNSGIDGMK + Oxidation (M)
☒	6338	881.3697	2641.0873	2640.1068	0.9804	0	8	1.6e+02	1	SMVGDLAAGGGGGSGGGGVEEEEDMFPR + Oxidation (M)
☒	7688	764.7876	3818.9016	3817.9164	0.9852	1	8	91	1	SQIALCCMSHSPLLNLPGPAQELLDEIDKAIAAAR + Oxidation (M)
☒	7849	1010.4512	4037.7757	4037.8810	-0.1053	1	8	68	1	GGSRDAVVCVEGSIEGTAVQVAVFDFSFMGGSMGSVVGEK + Oxidation (M)
☒	2751	963.4581	962.4508	961.4902	0.9606	0	8	5.3e+02	1	LLLADMDR + Oxidation (M)
☒	6016	834.0646	2499.1720	2498.1405	1.0314	1	8	2.2e+02	1	NWMFSNAKSPPHCELMAGHLR + Oxidation (M)
☒	8319	746.0018	4469.9671	4470.1644	-0.1973	1	8	51	1	NPKQFDVLLTGNMFGDILSDEAAMLTGSIGMLPSASLNVEDK + 2 Oxidation (M)
☒	8598	775.8398	4648.9951	4649.0325	-0.0373	1	8	42	1	MRECISVHVGQAGVQMGNTCWELYCLEHGIQPDGQMPSDK + 2 Oxidation (M)
☒	3632	687.4706	1372.9266	1372.7020	0.2246	1	8	3.3e+02	1	EKADMPLEVSVR
☒	4237	760.3547	1518.6948	1519.7379	-1.0431	1	8	3.8e+02	1	ERTGGSTFINPEGR

☒	7024	757.8334	3027.3045	3027.5448	-0.2403	1	8	1.3e+02	1	TQPRILGVPQVIQGTNDDDMIVLYGGGR + Oxidation (M)
☒	7310	691.9341	3454.6341	3453.6697	0.9644	1	8	1.1e+02	1	HGRVDDSPYGGGAGMLLMPQPIFDAIADLPEAK + Oxidation (M)
☒	7642	764.3858	3816.8926	3816.7434	0.1492	1	8	91	1	LLCGGEALKGDGFDNGAWVAPTVFTDCSDEMTIVR + Oxidation (M)
☒	7817	804.5366	4017.6466	4018.7805	-1.1339	1	8	59	1	IHDNDSSSSSSFSQSMMTVNIPDEFRCPISLDLMR + Oxidation (M)
☒	2514	392.4493	782.8840	783.4239	-0.5398	0	8	1.3e+02	1	ADIPVNR
☒	3226	592.6711	1183.3276	1183.6924	-0.3648	1	8	3e+02	1	KDERPILVSK
☒	4493	797.3544	1592.6942	1591.8062	0.8881	1	8	3.4e+02	1	MADMSVKQLADLVR + Oxidation (M)
☒	5185	630.2642	1887.7708	1888.0339	-0.2632	1	8	2.7e+02	1	LSGGVAVIKIGAATEVEMK + Oxidation (M)
☒	7011	605.0878	3020.4026	3021.4212	-1.0186	0	8	1.5e+02	1	MLLAASEEAVSYGANTFMIYTGAPQNTR + Oxidation (M)
☒	7278	684.7157	3418.5421	3417.7829	0.7592	1	8	1.1e+02	1	YANVPAGLRGLGITFITTGLMALGFMSFSGVQL + Oxidation (M)
☒	3363	634.9951	1267.9756	1267.5788	0.3968	0	8	2.8e+02	1	MVMADTIATGDK + Oxidation (M)
☒	5275	649.3568	1945.0486	1943.9087	1.1398	0	8	2.9e+02	1	FNQVMAGIDAAFEAGFEK
☒	6144	639.7889	2555.1265	2555.3570	-0.2305	1	8	1.9e+02	1	REEFFLGSVINAIVSQAMFLLR + Oxidation (M)
☒	7155	1085.1547	3252.4423	3251.5923	0.8500	1	8	1.2e+02	1	ATADYMGMLATVMNALALQDAMRHIGLVSR + 2 Oxidation (M)
☒	8898	992.4594	5948.7127	5947.8873	0.8255	0	8	19	1	SHVDLDALGSMQWTPLHLAAFQGEEGVMLALLQCGANPNAAEQSGWTPLHLAVHK + 2 Oxidation (M)
☒	7824	1005.6680	4018.6429	4017.8785	0.7644	1	8	61	1	YPMSYISFHFWALQGQYQNDLRGLTFDSQGSAFK + Oxidation (M)
☒	8221	880.6122	4398.0246	4397.1568	0.8678	1	8	57	1	IRTPSFPHLQMISAVAPGMMVADLVAYLGSIDYVMSDVDR + 2 Oxidation (M)
☒	4318	771.3835	1540.7524	1541.8487	-1.0962	0	8	3.7e+02	1	NMAILGIEGISALPK + Oxidation (M)
☒	4601	817.3694	1632.7242	1632.7453	-0.0211	0	8	3.4e+02	1	GIAFSMSYNGISNEK + Oxidation (M)
☒	5716	1141.4833	2280.9520	2282.0500	-1.0980	0	8	2.1e+02	1	SGVLPYMICNQPPEFDFPR + Oxidation (M)
☒	5731	767.0406	2298.1000	2297.1499	0.9501	0	8	2.4e+02	1	VIPQQIDTAAQVTALSEDNER
☒	5756	1168.0331	2334.0516	2333.1031	0.9485	0	8	2.3e+02	1	SHSYILINMLEPVEADAEMR + Oxidation (M)
☒	3620	456.5491	1366.6255	1365.7867	0.8387	0	8	3.9e+02	1	IIQGDELAPGILK
☒	3994	724.2031	1446.3916	1446.7579	-0.3663	1	8	2.7e+02	1	ISLRASDNPFATR
☒	4698	832.6320	1663.2494	1663.7988	-0.5494	0	8	2.3e+02	1	GSPGAPGLPGDPGLMGER
☒	5970	833.3734	2497.0984	2496.2867	0.8117	1	8	2e+02	1	QTTSNSGLMNKANIQALIHGLNR + Oxidation (M)
☒	8441	904.2247	4516.0871	4515.2058	0.8813	1	8	54	1	LNQIGAGDQGLMFGYATNETEELMPLPIMLSHHLMQRIAK + 3 Oxidation (M)
☒	6949	600.1102	2995.5146	2995.5232	-0.0086	1	8	1.6e+02	1	QFPWAVQTWYDSIGAINKIQDFLQK
☒	7672	955.2342	3816.9077	3816.8671	0.0406	1	8	92	1	IIAETGAGQHGVATALACAMLDLPCVIYMGAKDVER + Oxidation (M)
☒	7898	1017.9668	4067.8381	4068.9489	-1.1108	0	8	74	1	NILFLTLAAIYAYQVEAEAVITGVCETDFSGYPDCR
☒	8053	849.4071	4241.9991	4240.9423	1.0568	1	8	67	1	DAEALKPVYEEVPGWADEVDGICNYDDLPTEAKDYVR
☒	2372	690.0430	689.0357	688.3755	0.6602	0	8	55	1	SVGIEGK
☒	5410	506.2597	2021.0097	2020.0000	1.0096	1	8	2.8e+02	1	DAYALLKAEDGVISVEVDN
☒	2651	450.2726	898.5306	898.4443	0.0863	0	8	4.7e+02	1	HTNAGMLR
☒	3236	597.8525	1193.6904	1192.6564	1.0341	1	8	4.2e+02	1	HVAEIAQKLNA
☒	8211	1096.0190	4380.0469	4379.1075	0.9394	1	8	62	1	GDSMIDAGINDGDIVVIEEGNTADNGDIVVALVEDHEATLKR
☒	4357	777.3694	1552.7242	1553.6740	-0.9498	0	8	3.7e+02	1	VTDGAGGQSASSSMQR + Oxidation (M)
☒	4436	786.0081	1570.0016	1569.8111	0.1906	1	8	3.1e+02	1	RINPPSSGGSSSSPIK
☒	7458	599.7795	3592.6333	3591.6895	0.9438	1	8	1.1e+02	1	METQTVQQELESLPTTKMAQTNPMPGSVGPWK + 3 Oxidation (M)
☒	8359	641.8560	4485.9411	4486.4457	-0.5046	1	8	48	1	MLNKLNLPIHIIGGGLAGSEASWQIAQLGIPVILHEMRPQR + Oxidation (M)
☒	8552	766.1714	4590.9847	4590.0479	0.9369	0	8	45	1	NEGMDATHNPEFTSIEVYQAYADYQDIMDLTEGIIQHTAK + 2 Oxidation (M)
☒	8729	927.9796	5561.8339	5561.6164	0.2175	1	8	27	1	MTDNDALSLPPDVFAAEDLAAEIDEETSCVVVQTPDAFGNLRDLSPLAAACK
☒	3060	551.0492	1100.0838	1100.6077	-0.5239	0	8	3.3e+02	1	TIGLEPAVSSK
☒	7670	955.2336	3816.9053	3817.9455	-1.0402	1	8	93	1	RMGELGTTLIEGSVGVLTHCNTGSLATGGFGTALGVIR + Oxidation (M)
☒	8182	872.7819	4358.8731	4358.1149	0.7582	1	8	52	1	LLWAVEPLDGCGPLHPSWWSLMPPKQVPETVVDAACMR + Oxidation (M)
☒	5646	738.3361	2211.9865	2212.0881	-0.1016	1	8	2.5e+02	1	QLAFLNRSFVNMNMDRPK + 2 Oxidation (M)
☒	7476	903.9670	3611.8389	3612.7692	-0.9303	1	8	1.1e+02	1	ILGFMTGTSLDAVDMAVLETDGVEISAFGPAGERK + Oxidation (M)
☒	7741	987.1729	3944.6625	3944.0466	0.6159	1	8	67	1	YGAVPVVARVGGLSDTVVDANEMAIAAGVATGVQFSPVTR
☒	7856	1010.9097	4039.6097	4039.8615	-0.2518	1	8	58	1	DLAGFDCEGMSAAIEAAGCLFQYAQETQRSQLPHIR
☒	8424	904.0272	4515.0996	4515.2058	-0.1062	1	8	57	1	LNQIGAGDQGLMFGYATNETEELMPLPIMLSHHLMQRIAK + 3 Oxidation (M)
☒	2989	523.7527	1045.4908	1044.4038	1.0870	0	8	5.3e+02	1	MMSNGMAQK + 3 Oxidation (M)
☒	5595	724.9896	2171.9470	2172.0375	-0.0905	1	8	2.5e+02	1	ASVEYDTKVQEANWELYK
☒	5770	784.0527	2349.1363	2348.1682	0.9681	1	8	2.4e+02	1	TAELVTFGETMIRLSPPEGER + Oxidation (M)
☒	8753	1148.7484	5738.7056	5737.8933	0.8123	1	8	26	1	ALLMSSTATMVKAFDEAINDGVDVLSISLASAAPFRPIDSITGDLELGSFHAVMK + Oxidation (M)
☒	8907	1222.3850	6106.8886	6105.8778	1.0108	1	8	21	1	VIPAADLSQQISTAGTEASGTGNMKFMLNGALTIGTMDGANVEMAEEAGEENLFIFGMR
☒	3486	656.2335	1310.4524	1310.6540	-0.2016	0	8	3.2e+02	1	EFLSGALAGAMTK + Oxidation (M)
☒	4551	539.9874	1616.9404	1617.8185	-0.8781	1	8	3.5e+02	1	AIPNMTLFAPRDEK + Oxidation (M)
☒	4722	839.3042	1676.5938	1676.8304	-0.2366	1	8	2.7e+02	1	MYADRDLPGAGGLAVR + Oxidation (M)
☒	5205	951.2476	1900.4806	1900.8295	-0.3488	1	8	2e+02	1	LAEQSERYDDMAQAMK + Oxidation (M)
☒	6492	675.5829	2698.3025	2698.2214	0.0811	0	8	1.9e+02	1	VVMNSSSASQEIGDHQYTIICSEK + Oxidation (M)
☒	6764	719.3325	2873.3009	2872.3987	0.9022	0	8	1.7e+02	1	EVLANMGLSLGMSVSGWPPENIEDLAK + Oxidation (M)
☒	7193	828.3705	3309.4529	3310.5558	-1.1029	1	8	1.2e+02	1	ATNLEAISIDSNISPRCNFGIMTDQDGTVR + Oxidation (M)
☒	8546	1146.2286	4580.8853	4580.2331	0.6522	1	8	42	1	QIIHMIETSNCWYSVKFLMDNGISSLFSLGITPHHTYPK + Oxidation (M)
☒	3751	709.5770	1417.1394	1417.7347	-0.5953	1	8	2.8e+02	1	MDVSGGAAGALIGKR + Oxidation (M)
☒	6423	889.7779	2666.3119	2666.2729	0.0390	0	8	2e+02	1	QFLNYTLTGVGGFMAASMLMPMVR + 2 Oxidation (M)
☒	7902	1021.7123	4082.8201	4081.9197	0.9004	1	8	69	1	WHDQFIVDPIQGGAGTSMNMNANEVIGNRALEIMGHK + 2 Oxidation (M)
☒	8135	1432.3431	4294.0075	4295.1565	-1.1490	1	8	65	1	MRTISISDTEGGSGPGGNGGGGGGGGAIVPLGLGAGLNLNSLQVTTR + Oxidation (M)
☒	8379	1123.5311	4490.0953	4489.1563	0.9390	1	8	58	1	AMTIIQSQSTSNPCSISQMAAIEALNGTQDYIKSNALNFQK + Oxidation (M)
☒	8549	1146.4802	4581.8917	4580.9690	0.9227	1	8	41	1	IQSHGFPPEVEEQDDGTLPMSCAQESGMDHHSAACASARINV + Oxidation (M)
☒	2782	498.0177	994.0208	993.4913	0.5295	1	8	3e+02	1	MGVSGSKGQK + Oxidation (M)
☒	2685	917.4205	916.4132	917.4317	-1.0184	1	8	5.9e+02	1	KDYMFAK + Oxidation (M)
☒	4517	799.5385	1597.0624	1596.8035	0.2589	1	8	2.8e+02	1	GDKDIFTVADIFEK
☒	4923	881.4020	1760.7894	1759.8603	0.9291	0	8	3.3e+02	1	MIEISNLFFNYQNK
☒	7203	832.3683	3325.4441	3324.5757	0.8684	1	8	1.1e+02	1	LVTSYMYKGLMTFGGYQEDFMVVVSTQSK + Oxidation (M)
☒	3664	692.9847	1383.9548	1383.6387	0.3161	1	8	3.2e+02	1	RMGGVGSPMSFSR + Oxidation (M)
☒	4011	727.0016	1451.9886	1451.7442	0.2444	1	8	2.9e+02	1	SAVQEIMRDIYK
☒	4226	758.8463	1515.6780	1515.7967	-0.1186	1	8	3.9e+02	1	TLSPLKTCQLELN
☒	6656	950.0981	2847.2725	2847.3248	-0.0523	1	8	1.7e+02	1	LVKGELTQYYYSAPESFVMSCGFR + Oxidation (M)
☒	7408	708.5256	3537.5916	3536.8045	0.7871	1	8	1.1e+02	1	APVTEMPPAGSFLNQQQQLEVLTQQQKVNAAR + Oxidation (M)
☒	8119	855.1965	4270.9461	4270.1952	0.7509	1	8	63	1	AGGAMPAVLNAANEQAVALFIAEAIAFLEIPRLIEMVCDR + Oxidation (M)
☒	8392	645.2844	4509.9399	4509.3651	0.5747	0	8	50	1	ATATAIASMLSYLGGACAFLVGPLVVPAPNSTSGLLLYSGSVGAIR + Oxidation (M)
☒	8492	756.0020	4529.9683	4531.0942	-1.1258	1	8	49	1	EFAVVTDMKNSSSVSNTLTNGCVINGHLDFPSTTPLSGMESR + 2 Oxidation (M)
☒	3533	667.9662	1333.9178	1334.7558	-0.8379	1	8	3.3e+02	1	VLGLIFDKSSTR
☒	4754	844.4906	1686.9666	1687.8741	-0.9074	0	8	3.5e+02	1	SVDASQGPISTTASVIR
☒	2515	392.7473	783.4800	783.4351	0.0449	1	8	2.3e+02	1	GPGAAQRK
☒	7154	814.1163	3252.4361	3251.5923	0.8438	1	8	1.3e+02	1	ATADYMGMLATVMNALALQDAMRHIGLVSR + 2 Oxidation (M)
☒	7209	666.3235	3326.5811	3325.6938	0.8873	1	8	1.3e+02	1	IAEFDILFGKGISTLGCLLDMAEETNIVTR
☒	8270	1478.9187	4433.7343	4434.0910	-0.3567	1	8	45	1	FYPGISYRHLLVASNGLGANADCTPPHDVIDGEINDHMPR + Oxidation (M)
☒	2601	429.2431	856.4716	856.4515	0.0202	1	8	5.3e+02	1	TAGAREPR
☒	7057	769.8563	3075.3961	3074.5304	0.8657	1	8	1.5e+02	1	IIDRTNTTLYIVMEYCEGGDLASVITK
☒	8600	667.3092	4664.1135	4663.1521	0.9613	1	8	47	1	HATQPWQTQPDMAGSPPSLLSGSSAGSAGGGGYSLKSSPFSSVGEER + Oxidation (M)
☒	4720	838.6879	1675.3612	1675.7844	-0.4231	0	8	2.5e+02	1	LMGQLMQESHISMR + Oxidation (M)
☒	6511	904.0479	2709.1219	2708.2317	0.8902	0	8	1.6e+02	1	GAGETQACGSGACGAVAVGIMQGLLDSR + Oxidation (M)
☒	7095	1051.8013	3152.3821	3152.5158	-0.1338	0	8	1.3e+02	1	LSIGDLHFSQGDGEISFCGAIEMAGSITIK
☒	5509	691.9636	2072.8690	2073.0564	-0.1874	1	8	2.5e+02	1	LAANWVMGELAAKLNAEEK + Oxidation (M)
☒	6550	922.0549	2763.1429	2763.4086	-0.2657	1	8	1.6e+02	1	QHEREATIVANAGLPGAVTIATNMAGR + Oxidation (M)
☒	7303	862.4053	3445.5921	3444.6024	0.9896	1	8	1.2e+02	1	INELMSSSDRYSDLTDDSSLLVQAMELSGPR + Oxidation (M)
☒	7351	873.4137	3489.6257	3488.7327	0.8930	1	8	1.2e+02	1	GGGSSSLMLPPPPATSCGKPPLSSAAAAMSAGVGHKR
☒	2440	368.1871	734.3596	734.3381	0.0215	0	8	1.1e+02	1	AEGGVMR + Oxidation (M)
☒	5775	785.6699	2353.9879	2353.1848	0.8030	1	8	2.1e+02	1	AQISKTSVSQIFQSMIHNYR + Oxidation (M)

☒	6513	906.4002	2716.1788	2716.3490	-0.1703	1	8	1.8e+02	1	VSGQATLDGEDIYGPAGMDPVLLRAR + Oxidation (M)
☒	7125	1066.4736	3196.3990	3196.5099	-0.1109	0	8	1.3e+02	1	HDLAYIALDGMIGCMVNGAGLAMATMDIIK + 2 Oxidation (M)
☒	7214	555.6046	3327.5839	3326.6257	0.9583	1	8	1.3e+02	1	EFRENIEAGMLGVNIGVPAPMAFFPFSGWK + Oxidation (M)
☒	7846	808.5609	4037.7681	4036.8760	0.8921	1	8	72	1	DGLLQNVVADVNNGEEFLSESESEASLEIDSDIKDEK
☒	8529	914.4064	4566.9956	4566.3064	0.6892	1	8	49	1	HKVAATLSGAGPALIALTTNPDLPPEAVEYGAANGFTITAMTAGDR + Oxidation (M)
☒	8878	989.4479	5930.6437	5930.8492	-0.2055	0	8	19	1	QFLPFMLILSAICMLGFSASMGAGSTSLFLMIAFYALSGFFQSTGGSCSYSTITK + Oxidation (M)
☒	4325	772.4940	1542.9734	1543.8206	-0.8471	0	8	3.5e+02	1	SILTTAEEIINGQR
☒	7965	1033.5320	4130.0989	4129.8989	0.2000	1	8	77	1	MATMTVEAAAAAAAHAVAEAEAIMAEAEAAAREAEAAEAEAR + 2 Oxidation (M)
☒	6299	654.0450	2612.1509	2611.3255	0.8254	1	8	1.9e+02	1	RPQLIHFLETHESHERAGPTEK
☒	7621	941.2204	3760.8525	3760.7417	0.1108	1	8	1.1e+02	1	TPMNGVLGMLQMLMDTDLDTTQQDYVRTAQASGK + 2 Oxidation (M)
☒	8233	1103.0249	4408.0705	4407.8649	0.2056	1	8	64	1	GAHTMCASSLSSPRASQSLVDYDSSDDSDVESTEQCLANSK + Oxidation (M)
☒	2359	684.1998	683.1925	684.3918	-1.1993	0	8	19	1	ALAASPR
☒	2673	457.0001	911.9856	911.4825	0.5032	0	8	3.1e+02	1	TTHILDGR
☒	4585	814.5654	1627.1162	1626.7858	0.3305	1	8	2.6e+02	1	TISRFMECVNLNK + Oxidation (M)
☒	4982	604.0063	1808.9971	1808.8226	0.1745	0	8	3.3e+02	1	APNPGGPFGTMWMSLSK + 2 Oxidation (M)
☒	7831	673.8012	4036.7635	4036.1000	0.6636	1	8	73	1	TLVTDEISLGDYVLTGGELAAMTMIDATVRLIPEVIGK + 2 Oxidation (M)
☒	8382	899.4228	4492.0776	4492.3827	-0.3051	1	8	57	1	VQQSHVSVNQHIVGQINQGLTLLVGISPTDTDAELQWLARK
☒	5136	935.5166	1869.0186	1867.9978	1.0208	0	8	3.2e+02	1	FVDIGCGNGLLVHLLNK
☒	5324	992.4610	1982.9074	1982.9122	-0.0048	0	8	3e+02	1	GNYSISNVTHYFNPENK
☒	5729	575.2828	2297.1021	2298.1096	-1.0075	1	8	2.5e+02	1	HIITKCEEAGNDQIILCER
☒	7205	666.1231	3325.5791	3326.5581	-0.9790	1	8	1.4e+02	1	DMMQSELELLPPNTQVMEGQHVEKQLDR + 2 Oxidation (M)
☒	7357	878.9195	3511.6489	3510.7449	0.9040	0	8	1.2e+02	1	AYLTVLGVPEKPQISGFSSPVMEGDLMQLTCK + Oxidation (M)
☒	7423	596.4647	3572.7445	3573.8211	-1.0766	1	8	1.2e+02	1	SGSGDEAWIFADANHPMIVSLVTVSGLMTKILAK + Oxidation (M)
☒	8275	892.0168	4455.0476	4455.3723	-0.3246	0	8	61	1	KPMPEPLAAGLIALQEHLQNLQIQSIYAAAEADDVIATLATK
☒	8387	1126.7440	4502.9469	4502.3010	0.6459	0	8	50	1	DADGSVLDQGIALWFPGPNSFTGEDVLELQGHGGPVILDLLLK
☒	8639	789.5578	4731.3031	4730.1674	1.1358	1	8	52	1	MMFTVFLLIVLATTVVSFSTDDESDGSNEEPSADQTARSSMNR + 2 Oxidation (M)
☒	2680	457.7537	913.4928	913.4869	0.0060	0	8	5.7e+02	1	ISGPADVQK
☒	4508	798.7042	1595.3938	1595.7388	-0.3450	0	8	2.5e+02	1	EEVEALMNLFEEK + Oxidation (M)
☒	6257	871.1050	2610.2932	2611.2377	-0.9445	0	8	2.1e+02	1	HSTDILVYVGEGSAWDSIMVNYR
☒	6756	719.3307	2873.2937	2873.5031	-0.2094	1	8	1.7e+02	1	TQLTALAKAAEGLPVYLEVMAPMVADR + Oxidation (M)
☒	6801	970.4531	2908.3375	2907.4888	0.8487	1	8	1.7e+02	1	VVWLGNPAGMEATLVPRHGIPMEYVR + Oxidation (M)
☒	8450	753.6914	4516.1047	4517.2036	-1.0988	1	8	58	1	GGPAGDLYIFLSVKPHEFYQRDGADLYCSVPISMTTAALGGK + Oxidation (M)
☒	3618	683.2501	1364.4856	1363.6521	0.8336	0	8	3.4e+02	1	TEDFNGWLLNR
☒	4013	727.4910	1452.9674	1453.7096	-0.7421	1	8	3.1e+02	1	AYSGGTISQQMRR
☒	5563	712.3057	2133.8953	2133.1946	0.7007	1	8	2.5e+02	1	GKTLSIFGAGGAALAAIEAFIR
☒	7456	899.1647	3592.6297	3592.7508	-0.1211	0	8	1.1e+02	1	WTTAVLDVEADGGLAVVQSDQGFLLDTNGAMFPR
☒	8426	753.6857	4516.0705	4515.3653	0.7052	0	8	56	1	LIWGATLMIPLVSISSYLGLLSGLTVGMIEMPAGHALAGEMVR + 3 Oxidation (M)
☒	4968	898.1658	1794.3170	1794.9410	-0.6240	1	8	2.3e+02	1	KPMIADGGIRTHGDIAK + Oxidation (M)
☒	5798	791.1851	2370.5335	2371.1914	-0.6579	1	8	1.7e+02	1	AAVRSDGVGGSATNAELPMANLVR + Oxidation (M)
☒	6939	749.8843	2995.5081	2994.4431	1.0650	0	8	1.7e+02	1	GVEIGDGFLEASRPGSQAHDEIVVNADGR
☒	2035	574.2538	573.2465	573.2581	-0.0115	0	8	2.1	1	AAAGMPG
☒	4709	834.9803	1667.9460	1668.8848	-0.9388	0	8	3.6e+02	1	WHLGLKPEQTPHAR
☒	7893	1017.4620	4065.8189	4065.9495	-0.1306	1	8	75	1	VLFQNWGIEPIDNVMGMIGILPDMTPSTEMSMRGVR + 2 Oxidation (M)
☒	7918	822.7783	4108.8551	4109.7399	-0.8848	0	8	73	1	YDQFGHAGVGSSAASGGGQYGAGASDFSDIFSAFNDMFGGGK + Oxidation (M)
☒	8079	708.6725	4245.9913	4245.1371	0.8543	1	8	73	1	AIVIVALDNLTKGSSGQALQNANLMLGEDETAGLMMAPLFP + 2 Oxidation (M)
☒	8218	880.6047	4397.9871	4398.1374	-0.1503	1	8	58	1	IHFMLSSYAPVISAEKAFHEQLSVAEITNSAFEPASMMAK + Oxidation (M)
☒	2345	673.4307	672.4234	673.3759	-0.9525	0	8	57	1	LSGVNGK
☒	5442	682.3168	2043.9286	2043.8336	0.0950	1	8	2.9e+02	1	SMADEMNEKQMYDAHTK + Oxidation (M)
☒	6468	671.3309	2681.2945	2682.4455	-1.1510	1	8	2e+02	1	LAKGFGILAIPPFGLSTGSMYASLQK + Oxidation (M)
☒	6878	592.6849	2958.3881	2957.5876	0.8005	0	8	1.7e+02	1	TVYGLPHHPSVHAAGVVISENPLPLPTR
☒	7847	808.5609	4037.7681	4036.9437	0.8245	1	8	73	1	DFAEVAGQSPVDIQMVDHALDQLQVDQQGLDQIDRK + Oxidation (M)
☒	2672	456.3693	910.7240	911.4349	-0.7108	1	8	3e+02	1	KVDDDLHA
☒	8002	838.8006	4188.9666	4190.0230	-1.0564	1	8	78	1	IWSIDSILRCMELESLTPEIPLPQDLQMPLCSMSR + 2 Oxidation (M)
☒	8289	892.6194	4458.0606	4458.4782	-0.4175	1	8	61	1	DQLKVPMTSFLILSVFMILMPILLMNLLIGLAVGDIESVR + Oxidation (M)
☒	3839	716.0732	1430.1318	1429.6950	0.4369	1	8	2.9e+02	1	QEPHDSIYSAKR
☒	7286	684.9178	3419.5526	3419.6382	-0.0856	1	8	1.2e+02	1	LTPSPRGGPFNPSSGGPLPASSPSSFDGPSPPDTR
☒	8213	626.7305	4380.0626	4380.2115	-0.1490	0	8	66	1	NPFMGYNNHFYSQIILEGFVVVVEILFSLQFCISSLR
☒	8223	880.8121	4399.0241	4400.0679	-1.0438	0	8	62	1	VVYDELMSLLENMTNMCTLLMLEANSQPEPMIGPILDK + 3 Oxidation (M)
☒	5063	923.4274	1844.8402	1844.9778	-0.1376	0	8	3.3e+02	1	MSAQTLAISNLKPNTTR
☒	7544	735.7167	3673.5471	3672.8141	0.7330	1	8	88	1	EIQNASHRVVMITGDNPLTACHVAQELHFIEK + Oxidation (M)
☒	7897	814.5719	4067.8231	4066.8079	1.0153	1	8	78	1	MINMEMFASYTYTSMAFYFSRDDVALPGFAHFFK + 2 Oxidation (M)
☒	2388	707.4431	706.4358	706.4014	0.0345	0	8	45	1	AYVGLGK
☒	4822	853.4066	1704.7986	1705.6898	-0.8912	0	8	3.6e+02	1	NMPNEMNYIMDYR + Oxidation (M)
☒	5094	932.4569	1862.8992	1862.8621	0.0371	0	8	3.4e+02	1	HMAGLPVEAGWADGLPQD
☒	5478	1028.2300	2054.4454	2055.0969	-0.6514	1	8	2e+02	1	GDEILMILVMRSGTVLHR + Oxidation (M)
☒	7426	894.6637	3574.6257	3575.7501	-1.1244	0	8	1.1e+02	1	HLTVDHIPYGGGAGMIMRPDVVGDAVDSVLSTHK + 2 Oxidation (M)
☒	7858	809.1281	4040.6041	4040.1326	0.4715	1	8	65	1	VLVISDNGPETYLSVLACAKLGAIAVMADGNLPIAAIER + Oxidation (M)
☒	8248	1104.4786	4413.8853	4414.0436	-0.1583	0	8	55	1	LESLLNYQTMVSDITGLDMANASLLDEATAAAEAMQLCHR + 3 Oxidation (M)
☒	2568	821.1042	820.0969	820.3637	-0.2667	0	8	3.2e+02	1	EMDPSVK + Oxidation (M)
☒	3655	691.8100	1381.6054	1381.6547	-0.0493	1	8	4.2e+02	1	MVEEARELFDK + Oxidation (M)
☒	6227	870.7693	2609.2861	2610.3397	-1.0536	1	8	2.2e+02	1	MLKVLISAGEASGEMYGAALLDALR + 2 Oxidation (M)
☒	7754	793.1345	3960.6361	3960.9080	-0.2719	0	8	68	1	TGITIMQMELGLDTGPMLLMESLPIAADDTTASLHDK + 2 Oxidation (M)
☒	7873	811.3763	4051.8451	4051.0720	0.7731	1	8	81	1	DYTPSTNSLVSMASKFSLGQTELILLLMCFILALSR + 2 Oxidation (M)
☒	7944	1028.4775	4109.8809	4109.7399	0.1410	0	8	74	1	YDQFGHAGVGSSAASGGGQYGAGASDFSDIFSAFNDMFGGGK + Oxidation (M)
☒	8151	1076.4957	4301.9537	4302.9752	-1.0215	0	8	66	1	LENSEVTIEMDDPQQAAAGNNNMLGQMGIDLGDSLGALMPK + Oxidation (M)
☒	8219	1100.5050	4397.9909	4397.0825	0.9084	1	8	60	1	QSDPITGGGILNAMQAGMIAGEVVADAVSSGDTGVEGLMAYEKR + 2 Oxidation (M)
☒	8770	824.2557	5762.7390	5762.9530	-0.2140	1	8	28	1	MAFLGAAAGLPGFFAIMGATEMTNVWVFLLGTLVVGFGGGLFSHGTLTATMRLAPK + 3 Oxidation (M)
☒	3675	694.4058	1386.7970	1385.7092	1.0878	0	8	4.4e+02	1	HLVSGFDWAGLGK
☒	4950	595.2716	1782.7930	1782.9305	-0.1375	0	8	3.4e+02	1	WQIPDAAIFVEELPR
☒	7750	792.9705	3959.8161	3960.9184	-1.1022	1	8	86	1	EEATCPVAPTDLLPTSGASEEISSIGSLVMERSTELGK
☒	8121	714.7013	4282.1641	4281.9847	0.1794	1	8	73	1	LGGYGMMRITMILEPTTTSMSYPFMMLSLWGMVMTTR + 3 Oxidation (M)
☒	8675	608.4333	4859.4082	4858.2716	1.1366	0	8	48	1	DNSVDAIMASGAVSVFFPHGLGHHVGLEVHDVAEQSVMAATDDSSPR
☒	3640	688.4594	1374.9042	1375.8261	-0.9219	1	8	3.8e+02	1	RILELVMVFIK + Oxidation (M)
☒	3690	698.5234	1395.0322	1394.7518	0.2805	1	8	2.9e+02	1	AIVTRSATFNTSK
☒	6678	952.7679	2855.2819	2854.4793	0.8025	1	8	1.8e+02	1	ALVAMVEGMVAGVHVTTQDISAQLARR + 2 Oxidation (M)
☒	7699	968.4593	3869.8081	3870.9949	-1.1868	1	8	98	1	DMALGATPVFWIKDTNYLWVIMITCALTMLLVR + Oxidation (M)
☒	8090	850.4036	4246.9816	4247.1539	-0.1723	0	8	73	1	ILDENGATLPPGQEGDIALQVLPERPFGLFTHYVDNPSK
☒	7352	874.6647	3494.6297	3494.7385	-0.1088	0	8	1.2e+02	1	GVLQAVNHIEENIAPALCGLSSLDQATVDSVMK + Oxidation (M)
☒	7449	719.5320	3592.6236	3591.6386	0.9851	1	8	1.2e+02	1	IAWTQNCILTTTVDDFFDGDGSKEEMENLVK + Oxidation (M)
☒	8022	1048.7527	4190.9817	4190.0230	0.9587	1	8	78	1	IWSIDSILRCMELESLTPEIPLPQDLQMPLCSMSR + 2 Oxidation (M)
☒	8041	846.9794	4229.8606	4230.0863	-0.2257	1	8	66	1	TGGQALKLNFMNGGSVSGVVGTAANSVGDITTAGATSFASSVNAK + Oxidation (M)
☒	8110	853.3771	4261.8491	4261.1187	0.7304	1	8	62	1	NWGRVPALGTEPMFISDLADAVVESLPYVGAMAVSNLEAR + Oxidation (M)
☒	7813	1003.7155	4010.8329	4009.9199	0.9130	1	8	81	1	FGVFMVNLTKGSMIGPHWNPSACEISIVLEGEGMVR + 3 Oxidation (M)
☒	7886	1015.2803	4057.0921	4056.8036	0.2885	1	8	83	1	SMDMAFAPAIESGIPWVAILGNHDQESDMTRETMMK + 3 Oxidation (M)
☒	5287	650.2792	1947.8158	1947.9109	-0.0951	1	8	2.9e+02	1	AADKDNCFATEGPNLVAR
☒	5310	985.4885	1968.9624	1967.8385	1.1240	1	8	3.1e+02	1	QESEEGEGDFWVDEKGK
☒	6313	875.0527	2622.1363	2621.3714	0.7649	1	8	1.9e+02	1	AQEARAAYQAHIEQPWTLAQVLK
☒	7319	866.3682	3461.4437	3461.6985	-0.2548	1	8	1e+02	1	TADSGTMGLGDTKDGSNGADLVLPVPVGTVVFEAR + Oxidation (M)
☒	8522	913.5901	4562.9141	4562.1893	0.7248	1	8	46	1	MFLVNSFLKGGGGGGGGGGLGGGLGNVLGGLISGAGGGGGGGGGGGGGGGGTAMR + Oxidation (M)
☒	2647	896.6708	895.6635	895.4143	0.2492	0	8	3.5e+02	1	ILCTDMK + Oxidation (M)
☒	3622	684.8442	1367.6738	1367.8388	-0.1650	0	8	4.4e+02	1	VQLVLVGLADTLK

☒	4829	858.1000	1714.1854	1713.7888	0.3966	1	8	2.6e+02	1	VVMAMYNPKMIDDR + 2 Oxidation (M)
☒	5793	786.3818	2356.1236	2356.1807	-0.0571	1	8	2.7e+02	1	AGNIPLVPGVPLPDFMEKMGDK + 2 Oxidation (M)
☒	8606	934.4248	4667.0876	4667.3406	-0.2530	1	8	49	1	LLPEKMTLIPMNMMQVMSEGGPQPQSIVHFSISNQTVAVVNR + Oxidation (M)
☒	5572	537.7689	2147.0465	2148.1399	-1.0934	1	8	2.9e+02	1	NPIASGIRGQELTPQNALNR
☒	8576	771.6652	4623.9475	4624.2360	-0.2885	1	8	43	1	TAIVTLLSLATLIGFGGSAIAQNAMGTHMNGDGTDMSHSQGKYSGK + Oxidation (M)
☒	6039	630.3013	2517.1761	2518.2816	-1.1055	1	8	2.3e+02	1	FSDLLETARELGADALATGHYIR
☒	7822	1005.4205	4017.6529	4016.9459	0.7070	0	8	66	1	RPAEPSLGGLPQTMSGDGSGGMSAVSDGSSSSLAAPASLFPLK
☒	8608	934.6225	4668.0761	4667.4395	0.6366	1	8	47	1	NVFPVPSAGIRPGIVPQVLGDYGRNVILNAGTGIMDHPSGPASGVR + Oxidation (M)
☒	8673	810.7420	4858.4083	4859.2862	-0.8779	1	8	50	1	HILANFNNYQFFIGENMNPDGMVALLDYREDGVTPFMIFFK + 2 Oxidation (M)
☒	6309	654.5455	2614.1529	2613.1622	0.9907	1	8	2.1e+02	1	NMVELGSGNSFQIPDDMRSVCNK + Oxidation (M)
☒	7198	1104.4889	3310.4449	3309.5719	0.8729	1	8	1.2e+02	1	VMIHQPASSFYEGQAGECMIEAEEVLKLK + Oxidation (M)
☒	7363	703.3387	3511.6571	3510.7634	0.8937	1	8	1.2e+02	1	LLAEICLDIQEPMMYVLPYNSKCDLLTLR
☒	5611	1096.0237	2190.0328	2190.9521	-0.9193	1	8	2.9e+02	1	ALMCDEEHEIGSEKETSAR
☒	7561	924.6699	3694.6505	3694.8166	-0.1661	1	8	1e+02	1	MKWMSLVFLCGLLAMAVASPLNPGNVIINGDCR + 3 Oxidation (M)
☒	8447	753.6891	4516.0909	4517.2036	-1.1126	1	8	60	1	GGPAGDLYIFLSVKPHEFYQRDGADLYCSVPISMTTAALGGK + Oxidation (M)
☒	8891	992.2923	5947.7101	5947.8873	-0.1771	0	8	20	1	SHVDLDALGSMQWTPLHLAAFQGEEGVMLALLQCGANPNAAEQSGWTPLHLAVHK + 2 Oxidation (M)
☒	2424	724.4838	723.4765	722.3599	1.1166	0	8	56	1	VGSGEFK
☒	4320	771.5069	1540.9992	1540.7668	0.2325	0	8	3.3e+02	1	ATAHVDDIVAMVQR + Oxidation (M)
☒	7321	866.3739	3461.4665	3461.6985	-0.2320	1	8	1.1e+02	1	TADSGTMGLGDTKDGSNGADLVLPVPVGTVVFEAR + Oxidation (M)
☒	7842	808.5603	4037.7651	4038.8496	-1.0845	1	8	76	1	ADEALAAGAEAVGMDDLAEKMQAGDLNYDVVIATPDAMR + 2 Oxidation (M)
☒	8573	925.7944	4623.9356	4624.2360	-0.3004	1	8	43	1	TAIVTLLSLATLIGFGGSAIAQNAMGTHMNGDGTDMSHSQGKYSGK + Oxidation (M)
☒	4019	729.0591	1456.1036	1456.7748	-0.6712	0	8	2.9e+02	1	SPIICLYGPPGVGK
☒	5552	708.1294	2121.3664	2121.1028	0.2636	0	8	2e+02	1	KPYPVIATTSELMTTGVDAK
☒	5773	588.5116	2350.0173	2349.2951	0.7222	1	8	2.4e+02	1	VLVVGGSQGARILNQAMPEIAAR
☒	8179	727.3234	4357.8967	4357.1722	0.7245	1	8	59	1	DEQLDLARVLAVHEIAPDSQIILDANMGFSAGSMLAFLDR + Oxidation (M)
☒	8561	923.4200	4612.0636	4611.4337	0.6299	1	8	54	1	IILQGQPGIPGIPGVPGINGSEGLKGDPGPQGLPGETGFDGIPGVAGPK
☒	8677	811.0767	4860.4165	4861.5310	-1.1145	1	8	49	1	TLVELFGLARELNIDAEGIDMGPSPTDAALAADLALPIEELELTVR
☒	1929	546.8377	545.8304	546.2510	-0.4206	0	8	0.44	1	GGGGGSR
☒	4374	781.2591	1560.5036	1560.7314	-0.2278	1	8	2.9e+02	1	MTLEDAQRNSPQR + Oxidation (M)
☒	5327	993.4622	1984.9098	1986.0422	-1.1324	1	8	3.1e+02	1	VYSGVLQSGSYVLNTSKGK
☒	6455	889.7816	2666.3230	2667.3292	-1.0063	0	8	2.2e+02	1	HWETTILPSGYGLEARPVAEANEK
☒	7706	646.4753	3872.8081	3872.9560	-0.1479	1	8	96	1	RIIMYGVPENPVFWGEVVGFLGLDPAGTAEAAEGGVR
☒	7796	999.9619	3995.8185	3996.7485	-0.9300	1	8	87	1	ELEGICNPIIAKMYEGEGAGMGAAAGMDEDAPSGGSGAGPK + 2 Oxidation (M)
☒	6110	848.0575	2541.1507	2541.3114	-0.1608	1	8	2.2e+02	1	KDLAADPIEQFNLWLEQAIEAK
☒	7414	711.3256	3551.5916	3551.6627	-0.0711	1	8	1.1e+02	1	WQMEALGSDAVEIPNGVDVDSFASAARLDGYPR + Oxidation (M)
☒	8456	905.1981	4520.9541	4520.1808	0.7734	1	8	52	1	NEGTIVSVSDGIVRINGLAECMQGEMIELPNGGFAMALNLER + Oxidation (M)
☒	2655	900.6152	899.6079	899.4209	0.1870	1	8	5.8e+02	1	GDRGAPGDR
☒	4973	899.4188	1796.8230	1797.9182	-1.0952	1	8	3.6e+02	1	YAKLDVNIIATSDMTK + Oxidation (M)
☒	6841	976.4542	2926.3408	2926.4825	-0.1418	1	8	1.9e+02	1	FSVVTDSGSGIPSTIWGSVTYTGRVPTR
☒	6898	995.4594	2983.3564	2984.4987	-1.1424	0	8	1.7e+02	1	QVLVAILNDTPDIHVMGAASDPIFAMSK + 2 Oxidation (M)
☒	7051	1020.4668	3058.3786	3059.5651	-1.1865	1	8	1.6e+02	1	MTEPTLNFHIQTHQNQFKIPHELIK + Oxidation (M)
☒	7202	1109.1655	3324.4747	3324.7242	-0.2495	1	8	1.3e+02	1	SPDPETDLVSFQVPKSVELPQATFLSQPLR
☒	7359	878.9204	3511.6525	3511.6237	0.0288	1	8	1.2e+02	1	GFKMIVEGEMDNIPEQAFYMVGTIEEVFEK + 2 Oxidation (M)
☒	7440	898.9137	3591.6257	3592.6297	-1.0040	0	8	1.2e+02	1	YIVSISTLETGSETIDSENCTMSNAIGASWNSR
☒	7907	1023.7230	4090.8629	4091.1006	-0.2377	0	8	80	1	MRPLLVFVLMVSVSLAFSLEGMPNNGGDSVASITANQAR
☒	8220	880.6088	4398.0076	4398.1705	-0.1628	1	8	63	1	DEGESSNGNPMVYFAVATVLELVFGILASIITMWFSRYR + Oxidation (M)
☒	8399	904.0233	4515.0801	4515.2058	-0.1257	1	8	61	1	LNQIGAGDQGLMFGYATNETEELMPLPIMLSHHLMQRIAK + 3 Oxidation (M)
☒	8623	937.4004	4681.9656	4681.2405	0.7251	0	8	41	1	DGYMHDPVPITISDPIMMHDFAITENYAVFMDLPLIFRPK + 2 Oxidation (M)
☒	8727	927.9783	5561.8261	5562.8353	-1.0092	0	8	30	1	MNSIAILEAVNTSYVPFNGQHVLTAMVAGVAYVAMKPVVDNIGLSWSSQVQK + Oxidation (M)
☒	2460	746.3443	745.3370	745.3970	-0.0600	1	8	3e+02	1	GEVKGEK
☒	5065	923.6223	1845.2300	1844.9493	0.2808	1	8	2.5e+02	1	NKPFESGVDSVGNARLR
☒	6018	834.0657	2499.1753	2498.3017	0.8736	1	8	2.5e+02	1	NAIVTLTYISTSQRDDFLNTVK
☒	6529	684.0496	2732.1693	2731.3456	0.8237	1	8	1.8e+02	1	GVKAMGLETCVTLGMLETHQAQGLR + 2 Oxidation (M)
☒	7963	1033.2775	4129.0809	4129.1122	-0.0313	1	8	81	1	FVDEGNFVDVMIIFKEVINNVIELGTDPFGNYLIQK
☒	4236	760.0549	1518.0952	1518.7824	-0.6872	1	8	2.9e+02	1	SPMTSSGNLVKSVGR
☒	4243	507.9385	1520.7937	1521.8151	-1.0214	1	8	4.2e+02	1	AYAKLVTSADTGAVR
☒	7287	685.1199	3420.5631	3420.7713	-0.2082	0	8	1.3e+02	1	TTPWDISLDIKPATAWALYAAATMVMTPLIK + 2 Oxidation (M)
☒	4183	751.3433	1500.6720	1499.7766	0.8955	1	8	4.3e+02	1	MGLEGPTAKEVVNR
☒	6092	633.8062	2531.1957	2531.2768	-0.0811	1	8	2.4e+02	1	EVAPADVHIVATGKDEHVFAAAER
☒	8072	850.2047	4245.9871	4246.0753	-0.0882	1	8	77	1	VTTITYNGHAATDVSSTFTSYPSHIVVRNLSIMNTYNR + Oxidation (M)
☒	8281	892.4187	4457.0571	4457.1241	-0.0669	1	8	67	1	DFAITQLFYTTNTFHDVLYSLGFTEEAGNFQVNNGNKGGK
☒	8471	906.6017	4527.9721	4529.0879	-1.1157	1	8	56	1	NESQQDALFDIYRVMRPGEPPTMDTAAAMFHSLFFDPER
☒	6626	564.6684	2818.3056	2819.4892	-1.1835	0	8	2e+02	1	SPALDAVVIGAGVTGIYQAFLINQAGMK + Oxidation (M)
☒	7366	879.1711	3512.6553	3511.6237	1.0316	1	8	1.2e+02	1	GFKMIVEGEMDNIPEQAFYMVGTIEEVFEK + 2 Oxidation (M)
☒	7475	722.9325	3609.6261	3609.8398	-0.2136	0	8	1.2e+02	1	WGMADGQVFSLVIMALTSAEVSLALAMVVYLHR + 2 Oxidation (M)
☒	5051	918.9700	1835.9254	1834.9281	0.9974	1	8	3.5e+02	1	NILENAMKVIASMETR + Oxidation (M)
☒	7018	757.1169	3024.4385	3024.2462	0.1923	0	8	1.8e+02	1	NPDGPFSSSTSMLNLQGNGMEGDEHESR + 2 Oxidation (M)
☒	7215	833.1519	3328.5785	3327.7496	0.8288	1	8	1.4e+02	1	DPNGLVAHECILDLRPLKSQLGIEVEDVAK
☒	7230	837.8596	3347.4093	3346.6941	0.7152	1	8	1.1e+02	1	LSSGSNGPKIMQAPCTVIIGYDLDFPQTLPK
☒	8315	894.9963	4469.9451	4469.9455	-0.0004	1	8	56	1	VASTMSDGANTMIEHDDTLPSQLGTMVINADDEEEEGTMKR + 2 Oxidation (M)
☒	8490	906.8044	4528.9856	4529.2723	-0.2867	0	8	56	1	HLFDLTPDQIEVIVHPQSIIHSMVGYSDGSVLAQLGVPDMR + Oxidation (M)
☒	6301	872.0551	2613.1435	2612.3632	0.7803	0	8	2.1e+02	1	VILNNIDGWVKPGEVTALMGASGAGK + Oxidation (M)
☒	6990	1501.7058	3001.3970	3001.3732	0.0238	1	8	1.8e+02	1	CCSESLVNRRPCFSGLEVDETYVPK
☒	7036	1016.4636	3046.3690	3046.5290	-0.1600	1	8	1.6e+02	1	AELGAMGALLDYVELTQVGRMPALMPPR + 3 Oxidation (M)
☒	7443	898.9164	3591.6365	3591.6498	-0.0133	1	8	1.2e+02	1	VDLYEDATTVAADDLHVAVAADEHDVPMYKMR + 2 Oxidation (M)
☒	8201	1092.2607	4365.0137	4365.0466	-0.0329	1	8	70	1	FNIPCDAAQVYAGDDMSIDYVASVKPSFNMIEHLMKLK + 3 Oxidation (M)
☒	8258	631.7113	4414.9282	4414.1151	0.8131	1	8	59	1	AGHEVTVIDRQPGPALETSFANAGEVSFGYCSPWAAPGIPMK
☒	2303	644.0438	643.0365	642.3085	0.7280	0	8	14	1	AGGADPR
☒	6307	872.0580	2613.1522	2614.3077	-1.1555	0	8	2.1e+02	1	TGVMCWTVFQTINFSVIPPFVR + Oxidation (M)
☒	6327	877.4207	2629.2403	2629.4626	-0.2223	1	8	2.3e+02	1	VLGIDEAGRGPVIGPLVVAGVMIPER + Oxidation (M)
☒	6954	750.1319	2996.4985	2995.3991	1.0994	0	8	1.8e+02	1	GHTPMNDVTMLACIVSFISSSFLHSGR + 2 Oxidation (M)
☒	7032	760.8212	3039.2557	3039.5223	-0.2666	1	8	1.4e+02	1	EEIVSQADMLIIRELTGGIYFGTPSER + Oxidation (M)
☒	7808	1335.2267	4002.6583	4001.9797	0.6786	0	8	71	1	SDRPTVTLLGEMFPADPMVIGAMLAPMGLAVGPTVPMR + 4 Oxidation (M)
☒	8059	708.3297	4243.9345	4243.1371	0.7974	1	8	73	1	MNVSDLLNILNELLHPEYFSDYGPNGLQVGNAQTAIRK
☒	8385	751.4963	4502.9341	4502.2137	0.7204	1	8	53	1	EISYIHAEGYAAGELKHGPLALVDNEMPVVILAPHDDMLDK + 2 Oxidation (M)
☒	8578	925.7992	4623.9596	4624.2400	-0.2804	1	8	45	1	TGDTKPNFFQDCLMEVFDSLEQHIQNPVVLRSILSLMDR + 2 Oxidation (M)
☒	8916	1222.7870	6108.8986	6108.0331	0.8655	1	8	23	1	ILGLGDQGIGGMGIPIGKLALYTACGGISPAYCLPILLDVGTNNQQLLDDPMYMGWR + 2 Oxidation (M)
☒	2363	684.2012	683.1939	684.3919	-1.1980	0	8	20	1	AVGVPSR
☒	4808	847.9280	1693.8414	1693.8900	-0.0485	0	8	3.9e+02	1	WSLSSLNALNHPISR
☒	7432	896.2213	3580.8561	3579.9011	0.9550	1	8	1.3e+02	1	LMVSLLEHNQTPEGKVIVPEVLRPYTGFDIIG + Oxidation (M)
☒	7507	910.4731	3637.8633	3637.8895	-0.0262	1	8	1.2e+02	1	SMVVPEMIGLTIAVHNGRQHVPILVTENMVGHK + 2 Oxidation (M)
☒	7939	1028.4717	4109.8577	4110.8901	-1.0324	1	8	76	1	GDAKLPELSPLDLSGSGMMFGVHDGFSELGMSYSSSVFK + 2 Oxidation (M)
☒	6598	703.0643	2808.2281	2808.3202	-0.0921	1	8	1.9e+02	1	EPTSAQEDSQFIRTYTGGVLHTEDK
☒	7891	1016.9727	4063.8617	4063.9354	-0.0737	0	8	85	1	GGPSDLFEEASSASVSIPMMSQTESLNVSVSLGIALHER + 2 Oxidation (M)
☒	8062	850.0027	4244.9771	4245.1371	-0.1600	1	8	79	1	AIVIVALDNLTKGSSGQALQNANLMLGEDETAGLMMAPLFP + 2 Oxidation (M)
☒	8428	753.6872	4516.0795	4517.2326	-1.1530	1	8	61	1	LQRALTQFMLDSHTDAGYEEVYVPFLVNQDSLHGTGQLPK
☒	3270	611.2741	1220.5336	1219.6673	0.8664	1	8	4.9e+02	1	KVGNYSINLGR
☒	3724	704.5211	1407.0276	1407.6300	-0.6023	0	8	3.1e+02	1	ADADAALSTAMESR
☒	4677	824.8627	1647.7108	1647.7634	-0.0526	1	8	3.9e+02	1	AQGAEISEMNSPSRR + Oxidation (M)
☒	4979	903.3962	1804.7778	1803.9090	0.8688	1	8	3.6e+02	1	HLFFCTNPKSVDIAR
☒	3279	619.2525	1236.4904	1236.6714	-0.1809	1	8	4.4e+02	1	AKVGISYDAVSK

☒	6537	912.0632	2733.1678	2734.2876	-1.1199	1	8	1.8e+02	1	EEMNRAGAIEMLMPTLQQADLWR + 2 Oxidation (M)
☒	7760	992.0192	3964.0477	3963.8771	0.1706	1	8	95	1	DIILHLINQYGVDFGTGYALEFGGSTMRNLSMDER + 2 Oxidation (M)
☒	3379	639.2761	1276.5376	1275.6129	0.9248	1	8	5e+02	1	VLPSSMDKDER
☒	3649	690.9424	1379.8702	1380.7224	-0.8521	1	8	4.1e+02	1	RLMGLPASFFDK
☒	6506	903.7151	2708.1235	2708.3778	-0.2543	0	8	1.8e+02	1	ALFAAGMRPINNVVDVTNYVMLER + Oxidation (M)
☒	7768	993.4850	3969.9109	3970.9636	-1.0527	1	8	97	1	FSGGTRGPMIGHIAPEAFVGGPIGALEDGDHITVDIPDR
☒	7993	838.0043	4184.9851	4183.9388	1.0463	1	8	82	1	DCLVNLLDTPGHEDFSEDTYRTLTAVDSCLMVIDAAK
☒	4449	788.1198	1574.2250	1574.8814	-0.6563	1	8	2.9e+02	1	TTLTAAITKVMAAAGR
☒	5554	532.9529	2127.7825	2128.9261	-1.1436	1	8	2.2e+02	1	REMMAKPSLEEMIDDMR + 3 Oxidation (M)
☒	6901	995.4611	2983.3615	2984.5107	-1.1492	1	8	1.8e+02	1	TFRVVYPTCSYVFTTDPVNVEHILK
☒	7147	1084.8182	3251.4328	3251.5923	-0.1595	1	8	1.4e+02	1	ATADYMGMLATVMNALALQDAMRHIGLVSR + 2 Oxidation (M)
☒	7428	716.5292	3577.6096	3577.8928	-0.2832	1	8	1.2e+02	1	TVEFVTFFSCQMILYLIAIISIVEGSNRAIK + Oxidation (M)
☒	7712	777.9246	3884.5866	3885.0776	-0.4909	0	8	76	1	GGEEILALVEEGIDFELVPGVTSAIGVPTTIGLPVTHR
☒	7879	1014.2183	4052.8441	4053.9562	-1.1121	1	8	87	1	VCEVIVGILCGGMMMMILPSTSDGTALLTALKNMHAR + 4 Oxidation (M)
☒	8452	904.4238	4517.0826	4516.2439	0.8387	1	8	62	1	DNTHVLVVGDGVNDAPAMAAAHTSIAMGRAGADLTVQTADVVTIR + Oxidation (M)
☒	2728	468.0117	934.0088	933.4702	0.5386	0	8	4.2e+02	1	SGASGMIVGR
☒	8626	783.6651	4695.9469	4696.4444	-0.4974	1	8	42	1	NELMIVQGNHALLYHQILSASKPHWISTPPTLPLMCSAKIR + 2 Oxidation (M)
☒	3527	665.8240	1329.6334	1328.7227	0.9107	0	8	5e+02	1	VTLELYISTYK
☒	6757	719.3312	2873.2957	2872.3987	0.8970	0	8	1.9e+02	1	EVLANMGLSLGMSVSGWPPENIEDLAK + Oxidation (M)
☒	8619	781.0047	4679.9845	4680.3752	-0.3907	1	8	44	1	RPSVCNAAETLLIDAAIADHTMPRLVAALQGAGVTVHGGLSGDPDK + Oxidation (M)
☒	8643	947.5828	4732.8776	4733.2721	-0.3945	1	8	42	1	WGLAPMTGSLAPPPPYGSEQGSILLETGRENSQTPQWWGSAHSR
☒	3573	677.8710	1353.7274	1353.6387	0.0887	0	8	4.6e+02	1	TFHFTLAENMK + Oxidation (M)
☒	4176	749.7156	1497.4166	1496.7875	0.6292	1	8	2.9e+02	1	LLSGKEALGYGDFK
☒	5050	612.9416	1835.8030	1836.8312	-1.0282	0	8	3.5e+02	1	NVGNAIADFNMIENGDK + Oxidation (M)
☒	7189	828.3669	3309.4385	3309.4691	-0.0306	1	8	1.3e+02	1	ALDEQTQLTISSYRSSSSGSMASEDSTNWR + Oxidation (M)
☒	7682	764.5860	3817.8936	3816.7434	1.1502	1	8	1.1e+02	1	LLCGGEALKGDGFDNGAWVAPTVFTDCSDEMTIVR + Oxidation (M)
☒	5480	686.6393	2056.8961	2057.1190	-0.2229	1	7	2.9e+02	1	ATDNIAQMIEIIEKLIAR + Oxidation (M)
☒	7531	730.5147	3647.5371	3647.5753	-0.0381	1	7	98	1	QMPEEAAMQMIHAMEMMKNGSMWIVSMGQLK + 5 Oxidation (M)
☒	8143	860.7889	4298.9081	4299.9860	-1.0779	1	7	66	1	QLPQGPGEVPMASSVTTPTPTEEGECQSLVQSGRQEETSR + Oxidation (M)
☒	4084	740.3530	1478.6914	1478.7187	-0.0273	1	7	4.3e+02	1	LPNSTKMEAGEFR
☒	6962	600.3091	2996.5091	2996.4691	0.0401	1	7	1.9e+02	1	VSAAKLESMGLITCGDIQQCDLAMLLK + 2 Oxidation (M)
☒	8406	904.0247	4515.0871	4514.1644	0.9228	1	7	65	1	SGQMMLAQGLLLHLMPTDWRWSDCHALTDVDFEVLKPR + 3 Oxidation (M)
☒	2497	772.3293	771.3220	771.4239	-0.1019	0	7	4e+02	1	ATPQISR
☒	5055	920.9068	1839.7990	1839.9479	-0.1488	0	7	3.4e+02	1	INFLPDDSQNAQLLPR
☒	5277	973.9204	1945.8262	1946.1201	-0.2938	1	7	3.3e+02	1	LTTILPTDLKTPDHLIR
☒	6896	746.8455	2983.3529	2983.3717	-0.0188	0	7	1.8e+02	1	MLQSENIDVGSGAFAANSGTISLVDDDNK + Oxidation (M)
☒	7608	751.9435	3754.6811	3755.8352	-1.1541	0	7	1.1e+02	1	TLVIQNEDGQIEEPYSISAGLDYPGIGPMHANLAK + Oxidation (M)
☒	7696	774.9654	3869.7906	3870.0792	-0.2886	1	7	1e+02	1	TPIVTIDGPSGVGKGTISQLLAQHLGWHLLDSGAIYR
☒	5663	1115.7693	2229.5240	2230.2620	-0.7379	1	7	2e+02	1	VVRAMQVHKPILLEGSPGVGK + Oxidation (M)
☒	7460	899.1658	3592.6341	3591.9467	0.6874	1	7	1.3e+02	1	MKLVLAIVLILMLLSLSTGAETSDNSASSSATALR + Oxidation (M)
☒	8386	901.5954	4502.9406	4503.2268	-0.2862	1	7	55	1	LTKSGALISVAGGGDTVAALNQAGAAADFTYISTAGGAFLEWMEGK + Oxidation (M)
☒	3638	688.2601	1374.5056	1375.6653	-1.1597	0	7	4.1e+02	1	GTDPVAIDLGSMGK + Oxidation (M)
☒	7782	797.1567	3980.7471	3980.9268	-0.1797	1	7	84	1	TPGRTQHINFFELQNGNFMVDLPGYGYAQVPEAVR + Oxidation (M)
☒	8210	730.8507	4379.0605	4378.2036	0.8570	1	7	72	1	VLPNELTYKEAVAASSIANVLTASLLTNNLEVAGQMMEADR + 2 Oxidation (M)
☒	8532	914.4100	4567.0136	4567.2464	-0.2328	0	7	57	1	YWGAPIPMMTLEDGTVVPTPEDQLPVVLPEDVVMDGISSPLK + 2 Oxidation (M)
☒	8908	1222.5835	6107.8811	6108.0331	-0.1520	1	7	24	1	ILGLGDQGIGGMGIPIGKLALYTACGGISPAYCLPILLDVGTNNQQLLDDPMYMGWR + 2 Oxidation (M)
☒	2551	805.6876	804.6803	804.3362	0.3441	0	7	3.2e+02	1	GDAGENSR
☒	6417	889.7777	2666.3113	2667.3803	-1.0690	1	7	2.3e+02	1	DQPLIVRGPGAGAEVTAGGVFCDILR
☒	6865	736.8566	2943.3973	2942.5111	0.8862	1	7	2e+02	1	ALLQQAQALQNAYAPTEAVRGGGGGAQFR
☒	8008	838.8024	4188.9756	4190.0822	-1.1066	1	7	87	1	EYFPNYSLSELNATHFILNERPQEYVKLICDFIK
☒	8049	1060.0071	4235.9993	4236.0732	-0.0739	1	7	81	1	RIPVPAMNIINGGEHADNSVDIQEFMAVPVGAPSFGEGLR + 2 Oxidation (M)
☒	7977	1037.7103	4146.8121	4147.0978	-0.2857	1	7	75	1	VGLESNPQNFLLMHAMGPNVAGVIGSAIAAGVMLKYVLAM + 4 Oxidation (M)
☒	8312	745.6617	4467.9265	4467.3360	0.5906	1	7	58	1	LLAEGIDGAAPLPPLILDEPTVFLDGGHVSRLVDLVEDMQSR
☒	4169	748.3671	1494.7196	1493.8202	0.8995	1	7	4.4e+02	1	EQGKQQPALLPSAK
☒	8593	928.4303	4637.1151	4636.3193	0.7958	1	7	57	1	LILDEFASTMQWRSDGEDALLDVGSGSGNVLMDFVKPLLPIR + 2 Oxidation (M)
☒	4608	819.3760	1636.7374	1637.7276	-0.9902	0	7	4e+02	1	MVEAAAEASEELMNK + Oxidation (M)
☒	6885	743.3501	2969.3713	2969.3291	0.0422	0	7	1.9e+02	1	IDGECTHAGYFDVLGGSLETPWQFDR
☒	7513	728.5808	3637.8676	3637.9941	-0.1265	1	7	1.2e+02	1	FTLNMLVTMAPIVLILLGLLLFKMYPIDEER + 2 Oxidation (M)
☒	3549	672.5497	1343.0848	1342.5454	0.5394	1	7	3.4e+02	1	MKEIAMEEMEA + 2 Oxidation (M)
☒	4330	773.3845	1544.7544	1545.8225	-1.0680	1	7	4.6e+02	1	TLAMHFETLDKLK
☒	5762	781.0236	2340.0490	2341.2322	-1.1832	1	7	2.7e+02	1	MLKYIIPTIMLMPLTWMSK + 2 Oxidation (M)
☒	5920	1238.1272	2474.2398	2473.1750	1.0649	1	7	2.6e+02	1	ASGYTFTSYWMHWVKQRPGR + Oxidation (M)
☒	5988	625.5328	2498.1021	2499.1960	-1.0939	0	7	2.4e+02	1	LMNCGGFGMEATLLLVVGYSHSK + Oxidation (M)
☒	6924	500.2580	2995.5043	2996.5356	-1.0312	1	7	1.9e+02	1	AIHDLDISSVGQLEPWHPTELDQKIR
☒	7372	884.3991	3533.5673	3533.7972	-0.2299	1	7	1.2e+02	1	RTAAIMYAGGADTTVSTIQSFVLAMMVVPEVLK
☒	7025	1012.1218	3033.3436	3032.4406	0.9030	1	7	1.7e+02	1	MAMASAMDLSSPPTFFLSGTSTSSPSLRR
☒	7244	675.8832	3374.3796	3374.6565	-0.2769	1	7	1.1e+02	1	EDCSVLAFVLDHLLPHTQNAEDKDTPALAR
☒	3631	687.3671	1372.7196	1371.7510	0.9686	0	7	5e+02	1	TAFLAAGLPVAEGR
☒	4576	814.2584	1626.5022	1626.7858	-0.2835	1	7	2.9e+02	1	TISRFMECVNLNK + Oxidation (M)
☒	6288	654.0445	2612.1489	2611.3017	0.8472	1	7	2.2e+02	1	LYDEGDPSEKLELVTGTNVYITR
☒	7457	899.1656	3592.6333	3592.7098	-0.0765	1	7	1.3e+02	1	KDPTSTMSALQLAAVTQPGTGGSVTGGGGAAGGGGSAAGMK + 2 Oxidation (M)
☒	4003	726.0302	1450.0458	1450.7429	-0.6971	1	7	3.2e+02	1	RFGLEGHLTEHR
☒	8533	914.6054	4567.9906	4567.2464	0.7442	0	7	55	1	YWGAPIPMMTLEDGTVVPTPEDQLPVVLPEDVVMDGISSPLK + 2 Oxidation (M)
☒	4004	726.0541	1450.0936	1449.7286	0.3651	0	7	3.2e+02	1	YLKMAGETHGTVK + Oxidation (M)
☒	6790	965.7834	2894.3284	2894.4848	-0.1564	1	7	2e+02	1	KAMGDAAVAAAASIGYIGVGTVEFLLDER
☒	7525	608.4307	3644.5405	3643.8306	0.7100	0	7	1e+02	1	LMEMEVAEAEVQPSFLNPFWGGSALLKPAEPIK + Oxidation (M)
☒	7966	1033.7162	4130.8357	4131.0519	-0.2162	1	7	81	1	LGDWAFDEQVAAVFPNMITRSIPGYTNIITMIGMLSK + 2 Oxidation (M)
☒	8134	1074.5071	4293.9993	4293.0285	0.9708	1	7	76	1	GTSVGVFTGVMYHDYATRLTDVPEGIEGYLGTGNSGSVASGR + Oxidation (M)
☒	8750	957.4559	5738.6917	5739.5384	-0.8466	1	7	30	1	DAHKSQDLAAIDTAMAEINTVFQTASAEMYAQGGAQGGAQAGPDMDAGQNAGQDNSK + Oxidation (M)
☒	5008	604.6725	1810.9957	1811.9166	-0.9209	0	7	3.7e+02	1	EEAHGDLASNVAFLLAR
☒	6445	889.7786	2666.3140	2665.2150	1.0989	0	7	2.4e+02	1	NHGNNEADAALQGLAQQGVDMEDLR
☒	6882	740.8538	2959.3861	2958.4368	0.9493	1	7	2e+02	1	DSNVPEHFKLKPSPEMQAMSAFNNIK
☒	7219	667.4711	3332.3191	3332.3658	-0.0467	1	7	1.1e+02	1	DNNNQNNNNNQNDNNKNDNNQNDFDQIK
☒	7948	687.9517	4121.6665	4122.1473	-0.4808	1	7	65	1	DMRLVQFQAPHLVGPHLGLETGNGGGVINLNAFDPTLPK
☒	8215	880.0071	4394.9991	4395.3314	-0.3323	1	7	71	1	YWMSLGVRPEVVVGHSLGEFAALHIAGVLSAGDTLFLVGRR + Oxidation (M)
☒	8296	892.6205	4458.0661	4458.9861	-0.9199	1	7	70	1	ADTPSDMHDPMYMLVNLAIGGMAGPPTDGLMGGAEMKVDYVK + 4 Oxidation (M)
☒	8919	1021.3251	6121.9069	6120.9148	0.9921	1	7	24	1	STAMAMAAAASSSAAAATATTTAISNSTNTTASPATTNTINTTTKTITTSEVPLEPASNMAPK + 3 Oxidation (M)
☒	2358	684.1995	683.1922	682.3762	0.8160	0	7	22	1	GGPLSPR
☒	6815	584.2354	2916.1406	2916.4060	-0.2654	1	7	1.5e+02	1	VWEGGMAFHGGFLGVVVAMMLYARMR + 2 Oxidation (M)
☒	7314	864.9146	3455.6293	3454.7993	0.8300	1	7	1.4e+02	1	DKGADVNIFVQNVFMYLDSIVCNAVILLLR + Oxidation (M)
☒	7777	664.4648	3980.7451	3980.8740	-0.1288	1	7	86	1	DLDESSFEISLFAESLYEMLQYQMGSRIFEFLK + Oxidation (M)
☒	8284	892.4201	4457.0641	4458.2516	-1.1875	1	7	72	1	AMDVVGISTEEQDAIFSVVAAILHIGNIEFAKGEEIDSSIPK + Oxidation (M)
☒	8453	1130.2798	4517.0901	4516.2617	0.8283	1	7	65	1	ASLKNYLGQLNGQEVNDLYELVLAEVEQPLLDMIMQHTR + 2 Oxidation (M)
☒	8884	989.4498	5930.6551	5930.8492	-0.1941	0	7	22	1	QFLPFMLILSAICMLGFSASMGAGSTSLFLMIAFYALSGFFQSTGGSCSYSTITK + Oxidation (M)
☒	2625	882.0157	881.0084	881.5586	-0.5502	0	7	3.3e+02	1	ILDALLPK
☒	3422	648.0682	1294.1218	1293.6169	0.5049	1	7	3.2e+02	1	VEKMSMQAAQR + Oxidation (M)
☒	8638	676.7678	4730.3237	4729.9532	0.3704	1	7	59	1	WGRMFPGMGGMGGMGGMGGMMMGSRPMASCDVTKPNSCGNPALMK + 3 Oxidation (M)
☒	2264	617.9858	616.9785	616.3293	0.6493	0	7	7.2	1	GISAGGR
☒	7072	780.6044	3118.3885	3119.5274	-1.1389	1	7	1.6e+02	1	NEHPKFPDGGLMTQYLDSLPVGAYIDVK + Oxidation (M)
☒	7751	661.1117	3960.6265	3959.8292	0.7973	1	7	76	1	ATADLNLGDERASSAFGTSPETLDEFMMTDVLPSEVK + Oxidation (M)

☒	7913	683.7766	4096.6159	4097.0820	-0.4660	0	7	66	1	QPLMALVDALGDFTPQFLPPVEQQATVSLEYLNHATK + Oxidation (M)
☒	7955	1032.2224	4124.8605	4125.0186	-0.1582	1	7	79	1	KLDIIANEVLIEANEWGGHLAAMASEEMDDIYVVPNR
☒	8113	1066.4745	4261.8689	4261.1187	0.7502	1	7	71	1	NWGRVPALGTEPMFISDLADAVVESLPYVGAMAVSNLEAR + Oxidation (M)
☒	8469	755.6692	4527.9715	4527.0835	0.8880	1	7	60	1	LGSASARMLDVPGHTSGHVAFWFEDDAALFCGDTLFALGCGR + Oxidation (M)
☒	8632	787.3636	4718.1379	4718.2165	-0.0786	1	7	55	1	NVFQTTLINHFHLDMYYSQTTFVTLMQSMGESKSGMLLNK + 4 Oxidation (M)
☒	4500	798.1080	1594.2014	1594.7892	-0.5878	1	7	2.8e+02	1	FNVFQRHVEEYK
☒	4812	850.2001	1698.3856	1697.7533	0.6324	0	7	2.8e+02	1	HSEASSVVEYAYNDK
☒	6163	853.0494	2556.1264	2556.1261	0.0003	1	7	2.3e+02	1	DMNIADYDADLWQAMEQEKVR + Oxidation (M)
☒	6596	702.8134	2807.2245	2808.3202	-1.0957	1	7	2e+02	1	EPTSAQEDSQFIRTYTGGVLHTEDK
☒	6840	976.4537	2926.3393	2926.4825	-0.1433	1	7	2e+02	1	FSVVTDSGSGIPSTIWGSVTYTGRVPTR
☒	7516	1213.6313	3637.8721	3636.8320	1.0401	1	7	1.2e+02	1	AVVLLSGGLDSATAMAIAKDMGFQVYALSFSYGQR
☒	8431	904.2234	4516.0806	4517.1995	-1.1189	1	7	65	1	LERALGQYMLDLHSTEHGFTEMQVPALVNDATAYGTGQLPK + Oxidation (M)
☒	2592	850.4009	849.3936	849.5436	-0.1500	0	7	6e+02	1	IPAALLPR
☒	5940	830.3667	2488.0783	2487.2024	0.8759	1	7	2.4e+02	1	SPMTGRAPGADDNGSGSVTILEALR + Oxidation (M)
☒	6867	983.1302	2946.3688	2946.3553	0.0135	1	7	2e+02	1	FSSFPNLESLEAMKNDGITSISDTDGR + Oxidation (M)
☒	7192	1104.1577	3309.4513	3308.4577	0.9936	0	7	1.4e+02	1	CGPDYIDPGYLTLAADNPCHNLDSWMLGK + Oxidation (M)
☒	7246	676.1142	3375.5346	3374.7373	0.7973	1	7	1.4e+02	1	TDPVALDLHSINSHFFSLAIKWIMLFSEK + Oxidation (M)
☒	7565	743.5361	3712.6441	3713.8321	-1.1880	1	7	1.1e+02	1	TGFYMSLIGQPTESKVADSWLAAMVDVTNVIDQK
☒	7935	822.7819	4108.8731	4108.1701	0.7031	1	7	85	1	ALSELLLRPVTFAPDCLNAADVVSKMSPGDVVLLENVR
☒	7705	1291.2778	3870.8116	3869.7136	1.0980	1	7	1.1e+02	1	SFSEIRGLGSFESDSAPDIDQCVESMVETILSFSD + Oxidation (M)
☒	7710	776.1857	3875.8921	3875.9098	-0.0177	1	7	1.1e+02	1	SEIATQELTGEKFLEMANPNVAGLEAGSIEDIVTQR + Oxidation (M)
☒	2533	398.9362	795.8578	796.4191	-0.5613	0	7	1.5e+02	1	APAPGTAGR
☒	4927	882.7529	1763.4912	1763.9855	-0.4942	1	7	2.8e+02	1	IINIMLYINKSIDAK + Oxidation (M)
☒	7148	813.8659	3251.4345	3250.5306	0.9039	1	7	1.5e+02	1	EYAVGTESYELGFSKGLIDPGESVYEAANR
☒	7769	796.3334	3976.6306	3975.9131	0.7175	0	7	79	1	IAMSALAMGLASDKPVTVDDTAFIATSFPDFVPMMQR + 2 Oxidation (M)
☒	8466	906.6011	4527.9691	4528.1859	-0.2168	1	7	61	1	QTGSASSMVNGVVRLLTKPWDVVPMVTQMAMTDTTPFGQQR + 4 Oxidation (M)
☒	8691	1019.4930	5092.4286	5093.6019	-1.1733	1	7	46	1	AIVEELHQQYPELRLTIVDGNSHQAMEAADVVLLASGTATLEAALYK + Oxidation (M)
☒	5928	1242.6195	2483.2244	2483.2188	0.0056	1	7	2.7e+02	1	GPGEAAKAFAMGADAVMLGYALAGTK + Oxidation (M)
☒	7816	804.5360	4017.6436	4017.8070	-0.1634	1	7	73	1	AICYARFMVPEPSIFEIDAEAEEEAADAEGMADIAAGR + 2 Oxidation (M)
☒	7975	1036.7820	4143.0989	4144.1808	-1.0820	1	7	90	1	VSYFLMGIGAISLLVAGIGIGNVMLMSVVERTTEIGVMR + 3 Oxidation (M)
☒	8240	1103.9904	4411.9325	4411.9921	-0.0596	1	7	64	1	LGEKMYADMQAQGAAGAAGAAGGAGAAAGAEAAGASQQADDVVDAEFK + Oxidation (M)
☒	8340	639.8565	4471.9446	4473.0533	-1.1088	0	7	60	1	SPALMNFMMTGSWMTIGATFAAMIGAGMLVQSISYEQSPGPK + 4 Oxidation (M)
☒	7541	917.4202	3665.6517	3665.0030	0.6487	1	7	1.2e+02	1	WVYHLTSAWMILVVIASVFTNGLVLVATMRFK
☒	8058	708.3287	4243.9285	4243.1371	0.7914	1	7	79	1	MNVSDLLNILNELLHPEYFSDYGPNGLQVGNAQTAIRK
☒	8527	761.8257	4564.9105	4564.1944	0.7161	1	7	51	1	WMGPDSAFVGMTGFGASAPAGDLYRHFGITADHVVAEALELLR + 2 Oxidation (M)
☒	7880	1014.2215	4052.8569	4053.9562	-1.0993	1	7	94	1	VCEVIVGILCGGMMMMILPSTSDGTALLTALKNMHAR + 4 Oxidation (M)
☒	8849	836.8353	5850.7962	5851.8612	-1.0651	1	7	30	1	GGEPVVCLTAYTTPMARLVDAHCDIALVGDSLGMVLHGLPSTIGVSMEMMILHGK + 2 Oxidation (M)
☒	5722	1143.0817	2284.1488	2282.9566	1.1923	0	7	3e+02	1	VVCEEAGESMMVAAGAGNNDTR + Oxidation (M)
☒	7330	870.4015	3477.5769	3476.7982	0.7787	1	7	1.4e+02	1	ELARQIVGVIQAMGTFVEGLFVATAVPMEMNR
☒	7413	888.6571	3550.5993	3551.6946	-1.0953	1	7	1.3e+02	1	MLVTAVGMRTEWGNLMETLSQGGEDETPLQVK + 2 Oxidation (M)
☒	7652	764.3867	3816.8971	3817.9164	-1.0193	1	7	1.1e+02	1	SQIALCCMSHSPLLNLPGPAQELLDEIDKAIAAAR + Oxidation (M)
☒	8061	708.5011	4244.9629	4245.0720	-0.1091	1	7	85	1	RGDEMDVAVEIGQLPADDNGVASVPAGQTAPQNNALNLQVR + Oxidation (M)
☒	8203	874.2078	4366.0026	4367.1429	-1.1402	1	7	74	1	LTCVMIIAVLFLTAWTFATADDSGNGLENLFSKAHHEMK + Oxidation (M)
☒	8517	1137.5020	4545.9789	4545.9633	0.0156	1	7	60	1	TALQNAASVAALMLTTEAMVAESPKDDAPAGGMPGGMGGMGGMGMDM + 3 Oxidation (M)
☒	4518	799.7935	1597.5724	1596.7507	0.8217	1	7	3.4e+02	1	RSHVEMEFPFFR + Oxidation (M)
☒	6262	871.3881	2611.1425	2612.2850	-1.1425	1	7	2.3e+02	1	MVWMKFLGVLLMSMGCFVIFR + 3 Oxidation (M)
☒	7502	910.4724	3637.8605	3638.7201	-0.8596	1	7	1.3e+02	1	NVEIPQLNRLMGELLELSCCQSCPFSSTAAAK + Oxidation (M)
☒	7547	615.4378	3686.5831	3686.6653	-0.0821	1	7	1e+02	1	FMILYIEDSMFGSFDPNVPVSMQSREMDEIK + 2 Oxidation (M)
☒	8055	849.4083	4242.0051	4241.0227	0.9824	1	7	83	1	NMGMSLEDVVGECKLFYLAGQESTSTMLVWTMILLSR + 2 Oxidation (M)
☒	8653	802.3747	4808.2045	4809.2269	-1.0223	1	7	53	1	SIWAGDAETGVTIMQMDIGLDTGDMLKIATLPIDASDTSATMYDK + 3 Oxidation (M)
☒	2797	1001.1302	1000.1229	999.5713	0.5516	1	7	4.3e+02	1	LVTERGTPK
☒	8054	849.4074	4242.0006	4242.1307	-0.1301	1	7	83	1	NLFLVALEQLGDSLEEIWGFASGGNMLLGGDSAYGVKSIR + Oxidation (M)
☒	3432	650.1437	1298.2728	1298.6904	-0.4176	0	7	3.4e+02	1	GADIAMVPVALDK
☒	4321	771.5355	1541.0564	1541.8413	-0.7848	1	7	3.3e+02	1	EEARSILLDIVER
☒	6625	705.3292	2817.2877	2817.2780	0.0097	1	7	2.1e+02	1	MTLPCMYDQCKHMLFISTELQR + Oxidation (M)
☒	7895	814.3698	4066.8126	4065.6799	1.1327	1	7	87	1	LYKNGEGPDMEHSPNNSDSMACNSPSSPPIWDNSGSR + 2 Oxidation (M)
☒	4571	812.4261	1622.8376	1622.8562	-0.0186	1	7	4.4e+02	1	GAYITVAGGAAIMRTR + Oxidation (M)
☒	5693	1136.9963	2271.9780	2271.0736	0.9045	1	7	2.7e+02	1	SMNSLYIMADSGARGSQAQIR + Oxidation (M)
☒	7453	599.7784	3592.6267	3592.9287	-0.3020	1	7	1.3e+02	1	NQSVGDPTVDLIRTINIPLLTVVEMAQQPFLR + Oxidation (M)
☒	8521	1140.8009	4559.1745	4559.2636	-0.0891	1	7	67	1	IIGIDLGTTNSCVAIMEGGEPVVIQNAEGGRTTPSIIGFTSDGGR
☒	5801	793.7079	2378.1019	2378.1590	-0.0571	0	7	2.9e+02	1	AMELHTHISAQGWAFDRPSPK
☒	6563	692.2940	2765.1469	2765.4018	-0.2549	1	7	1.9e+02	1	MPVSQKLTLVAPPGGPNGDSSGGTQGVAK + Oxidation (M)
☒	6872	986.8034	2957.3884	2957.5876	-0.1992	0	7	2e+02	1	TVYGLPHHPSVHAAGVVISENPLPLPTR
☒	7747	988.5176	3950.0413	3949.7429	0.2983	0	7	1e+02	1	SFNSITVDGDMSTNDTIVAMANGAAGGELIDNTSSSAER + 2 Oxidation (M)
☒	8204	1095.2655	4377.0329	4377.0246	0.0083	1	7	74	1	DQYEAFIDALLAAEKTEFHAGETAGYFDGCLPIEVMAER
☒	4360	777.8489	1553.6832	1554.8365	-1.1533	1	7	4.4e+02	1	RTGLSPDIAEVIER
☒	6759	719.3314	2873.2965	2872.3194	0.9771	0	7	2.1e+02	1	GYQATHGIDPMILIDALASCMGHEEK + Oxidation (M)
☒	7781	797.1567	3980.7471	3979.9673	0.7799	1	7	90	1	ENFGYLEGINLTYVGDGRNNIAHSLMVAGAMLGVNVR + Oxidation (M)
☒	8160	861.6028	4302.9776	4302.8612	0.1164	1	7	79	1	MMQMLQGQGGGGGGGLPGLGGMDLQSMMSQMSGLMGGGGGGGGGRGR + 3 Oxidation (M)
☒	8583	773.3528	4634.0731	4635.2075	-1.1343	0	7	58	1	ALHHLFSEIIDNAMDEAVAGYADLIDITLDSNNYLTVTDNGR + Oxidation (M)
☒	2976	519.1361	1036.2576	1035.4906	0.7670	0	7	4.1e+02	1	TNLDAVMEK + Oxidation (M)
☒	3341	626.4417	1250.8688	1251.6684	-0.7995	0	7	3.9e+02	1	QSVIHVQASGAR
☒	4166	748.0153	1494.0160	1494.8042	-0.7881	0	7	3.4e+02	1	TPATPEEAAVIIQR
☒	7274	1135.8206	3404.4400	3404.6850	-0.2450	0	7	1.2e+02	1	NEDYDLLIFLLSGGASALMEYSNVPYEILR
☒	7435	1197.8937	3590.6593	3590.6206	0.0387	1	7	1.3e+02	1	IAVAEANVDNAEFDEKSLPPMSNLQGDEEADEK + Oxidation (M)
☒	7646	764.3862	3816.8946	3816.7434	0.1512	1	7	1.2e+02	1	LLCGGEALKGDGFDNGAWVAPTVFTDCSDEMTIVR + Oxidation (M)
☒	7780	664.4651	3980.7469	3980.8628	-0.1159	1	7	90	1	TMQLMNVPKGATALDVCCGTGDWTIALADAAGETGEIK + Oxidation (M)
☒	7941	1028.4730	4109.8629	4109.8292	0.0337	1	7	84	1	KPYVNWYQVMTIKPKESPATSSEDISSCSDCDSER + Oxidation (M)
☒	4354	776.0241	1550.0336	1549.8616	0.1720	0	7	3.4e+02	1	HPNIIAEFIAGQLK
☒	7477	1205.2833	3612.8281	3613.9375	-1.1095	1	7	1.4e+02	1	EKPVNKPHTNEVRNKPVMPSRPGAPKPPTAANR
☒	7659	955.2324	3816.9005	3815.9967	0.9038	1	7	1.2e+02	1	IMLSPHPYALRSHLCVGEETASLGVYLHSKPVVR
☒	3624	685.0966	1368.1786	1367.7885	0.3902	1	7	3.4e+02	1	IVVERLGTAAPSR
☒	5964	624.3205	2493.2529	2493.1338	0.1191	0	7	2.8e+02	1	SAETMQSALAAMQFYGINMTGK + Oxidation (M)
☒	6041	840.2802	2517.8188	2518.2519	-0.4332	1	7	1.8e+02	1	MSSVEAVTQQQLLAAENPVRMGK + 2 Oxidation (M)
☒	5843	810.5727	2428.6963	2428.3036	0.3927	0	7	1.9e+02	1	ELAETGVLVCAVVGFPLGAGLSAAK
☒	7997	1047.9994	4187.9685	4187.0297	0.9388	0	7	89	1	IRPDISISSDFIIGFPGENEQDFLDTMTLIDEIGFDK
☒	8597	930.6065	4647.9961	4647.3241	0.6721	0	7	54	1	MLPARPEPLSVAVEETAVVVVDMQNAYASPGGYLDIAGFDISGAK
☒	6485	539.6361	2693.1441	2694.1729	-1.0287	0	7	2.1e+02	1	DGNFYQTNVDANGVNHGGSEMVQNK
☒	7448	599.7778	3592.6231	3592.9170	-0.2938	1	7	1.4e+02	1	MNLFIMLTMSSITVSIVVALNLLTAKTSPDPEK + Oxidation (M)
☒	4208	756.3530	1510.6914	1511.8857	-1.1943	1	7	4.6e+02	1	LIPAGTGLIMNKIR + Oxidation (M)
☒	7384	707.9276	3534.6016	3535.5541	-0.9525	1	7	1.4e+02	1	SMNGDRDSEIALVIEDMQYEDGVMNEKPYR + 2 Oxidation (M)
☒	7872	811.3763	4051.8451	4052.9587	-1.1135	1	7	95	1	ESYSAMHFHADALHAMADITASGRIPLLVGGTMLYYK + Oxidation (M)
☒	7960	1033.0262	4128.0757	4129.1122	-1.0365	1	7	91	1	FVDEGNFVDVMIIFKEVINNVIELGTDPFGNYLIQK
☒	8352	1120.7600	4479.0109	4479.2441	-0.2332	0	7	69	1	ENAKPAFPVDYLIVSLTHGFPVAADTPPTFQAHTGFPWANR
☒	8511	758.5006	4544.9599	4545.2078	-0.2479	1	7	60	1	LWWPHSEAMIAFLMGYSETGDPALLRIFYQVAEYTFHR
☒	8594	928.4328	4637.1276	4636.2045	0.9231	0	7	62	1	YFGGSGPMSMPVPMMNVINGGAHANNTLDLQELMILPVGAASFR + 2 Oxidation (M)
☒	2993	525.7531	1049.4916	1050.5458	-1.0541	0	7	5.9e+02	1	HLSAEPELR
☒	5316	989.2819	1976.5492	1976.9857	-0.4364	1	7	2.5e+02	1	WDWRNGEVANVVITYR
☒	6619	939.4323	2815.2751	2816.3217	-1.0466	1	7	2.2e+02	1	MAMPDLTHDLTGKLLIAMPGMEDPR + 4 Oxidation (M)
☒	8655	687.8951	4808.2148	4809.0970	-0.8822	0	7	55	1	MEVDANEASNWTANFDIPMETAHVANLDSDGSDVWSTEEPLSSK

☒ 3816	711.3900	1420.7654	1419.6518	1.1137	0	7	5e+02	1	DAGDVIPDAFDASK
☒ 7839	673.9673	4037.7601	4037.1248	0.6353	1	7	87	1	GYNVKLLVIGSLETENSIDESDYLFLTQNPNVVLIK
☒ 4898	874.2126	1746.4106	1745.8546	0.5561	1	7	2.9e+02	1	DEGEKFDLIIMDPPK
☒ 7317	866.1190	3460.4469	3460.5049	-0.0580	0	7	1.2e+02	1	HDFDNHDVAIAGGGDSAIDMATMLNEFTHSTR + Oxidation (M)
☒ 7535	915.9028	3659.5821	3659.8988	-0.3167	1	7	1.1e+02	1	CRPLSQRPNGHLTGGKVQLNLMDLASAIPASQSK + Oxidation (M)
☒ 4229	759.1070	1516.1994	1515.6922	0.5072	1	7	3.3e+02	1	GCRLFMSTTNGTR + Oxidation (M)
☒ 6622	705.0764	2816.2765	2815.4765	0.8000	0	7	2.2e+02	1	MATPNCLILWVLLIADTVWTQSVR + Oxidation (M)
☒ 3428	649.1982	1296.3818	1296.7150	-0.3331	0	7	3.5e+02	1	LVAQNVAGQLER
☒ 3740	707.9279	1413.8412	1413.6677	0.1735	0	7	5.1e+02	1	NGSPYQAVEFFR
☒ 5170	941.6245	1881.2344	1880.9158	0.3186	1	7	2.7e+02	1	MAQALVVMADMLSADRK + 2 Oxidation (M)
☒ 7158	1085.4852	3253.4338	3254.4908	-1.0571	0	7	1.6e+02	1	WAAMQIGMSFISAYAMCAGEAAVADLSFAAK + Oxidation (M)
☒ 7772	995.9445	3979.7489	3980.8740	-1.1251	1	7	97	1	DLDESSFEISLFAESLYEMLQYQMGSRIFEFLK + Oxidation (M)
☒ 8879	989.4485	5930.6473	5930.8492	-0.2019	0	7	24	1	QFLPFMLILSAICMLGFSASMGAGSTSLFLMIAFYALSGFFQSTGGSCSYSTITK + Oxidation (M)
☒ 2282	634.2744	633.2671	632.2766	0.9906	0	7	12	1	GDAAGDK
☒ 6925	749.8834	2995.5045	2994.4474	1.0571	1	7	2.1e+02	1	AAHAALSSCPVTCPLSHMERTVSEVLR + Oxidation (M)
☒ 8559	922.6194	4608.0606	4607.2604	0.8002	1	7	59	1	EPLGTAGPLALARELLTDNQEPFFVLNSDVICDFPFDDMLK
☒ 8509	758.3405	4543.9993	4543.5102	0.4891	1	7	63	1	ENLLTLGQWELVLPWIVPMPLALEINQRLLIMGLFSLDR
☒ 7346	696.7239	3478.5831	3479.5569	-0.9738	0	7	1.4e+02	1	NGSLPGNTTYPTPANQSPNTPVDCVVSSMESSR + Oxidation (M)
☒ 8341	560.0005	4471.9458	4472.4613	-0.5155	1	7	63	1	AIALGAAGLPGIPSIKPDSLAMYCVGMLISASIAFTLTVILGKR + Oxidation (M)
☒ 7333	696.5231	3477.5791	3476.6691	0.9101	1	7	1.4e+02	1	TAAENVCYTLINVPMDSEPPSEISLKNDLEK
☒ 8249	631.5669	4413.9174	4414.1151	-0.1977	1	7	69	1	AGHEVTVIDRQPGPALETSFANAGEVSFGYCSPWAAPGIPMK
☒ 8628	783.8337	4696.9585	4697.9936	-1.0351	0	7	47	1	MAGYGHSTAGGFGSGSGSGPPGPQYMLPQYDEGDDPDADATPAGQGVR + Oxidation (M)
☒ 3686	696.5793	1391.1440	1391.7119	-0.5679	0	7	3.4e+02	1	DMGTVVFPSAQIK
☒ 4349	775.6340	1549.2534	1548.8875	0.3659	0	7	3.1e+02	1	ILVLPGDGIGPEITR
☒ 7055	1022.1307	3063.3703	3063.5050	-0.1347	1	7	1.9e+02	1	AYGGHIAIGLDARDGFVSTEGWLETSSVR
☒ 5523	1048.2501	2094.4856	2095.1026	-0.6169	1	7	2.4e+02	1	ITILGAGKVGMATAVMLMMR + 2 Oxidation (M)
☒ 6038	1258.2977	2514.5808	2515.2927	-0.7118	1	7	2e+02	1	MAADFPMISLVNSVNPVRIEGQK
☒ 6239	653.3297	2609.2897	2610.2354	-0.9457	1	7	2.6e+02	1	IAYVNKMDIMGANFFNVIEMMK + 2 Oxidation (M)
☒ 7569	929.4204	3713.6525	3713.8512	-0.1987	1	7	1.2e+02	1	GIIWPEGVTPFHAGIVNLKQGDSSTDLACEALYR
☒ 8196	1091.0106	4360.0133	4360.9563	-0.9430	1	7	77	1	HSPGEITGPGGHFMTSDSSLTKSPESLSSPAMEDLAMEWGGK + Oxidation (M)
☒ 8388	901.5967	4502.9471	4502.1035	0.8436	1	7	62	1	LIMAGNYEEALSVAKVQVEMGAQVLDINMDDGMLDGPSAMTK + 3 Oxidation (M)
☒ 8567	925.3967	4621.9471	4623.0639	-1.1168	1	7	53	1	SIMLAAQSLMCGHQQVMVAGGMESMSNVPYCMSRGATPYGGVK + 2 Oxidation (M)
☒ 4547	808.0690	1614.1234	1613.8988	0.2246	0	7	3.2e+02	1	EGLLVGISSGASAAAALK
☒ 8749	957.4558	5738.6911	5739.6736	-0.9825	0	7	33	1	MADLSETTVVLPDHPDTGTTGAFLDLMGFFGASTWYPAAGVTVMGADEVAHTPPK + 3 Oxidation (M)
☒ 6809	971.7875	2912.3407	2913.3783	-1.0376	1	7	2.1e+02	1	KVIAVIGDGAMSAGMAYEAMNNAGALNAR + 3 Oxidation (M)
☒ 7624	944.6796	3774.6893	3774.8922	-0.2029	0	7	1.1e+02	1	MDDFISISLLSLAMLVGCYVAGIIPLAVNFSEER + 2 Oxidation (M)
☒ 8005	1048.2495	4188.9689	4188.1372	0.8317	1	7	97	1	LEASVLSVCLSTQSSLYQIPESLGAAASTRVANELGAGNPK
☒ 8080	607.5775	4245.9916	4245.0560	0.9356	1	7	90	1	EMLFDIFFMMFNLGLTAYLIGNMTNLVVHWTSRTR + 4 Oxidation (M)
☒ 8261	883.9965	4414.9461	4416.0751	-1.1290	1	7	70	1	SETQVNNNQTAVVASGSQADNDFPPPPPVAVMKAEHCPPSTK
☒ 8371	898.5974	4487.9506	4488.4425	-0.4918	1	7	64	1	RTMVLGAIVLALGYFMTGMSLLKPEMIFIALGTIAVGNGLFK + 2 Oxidation (M)
☒ 8601	933.9973	4664.9501	4665.9626	-1.0125	1	7	49	1	TALQNAASVASLMLTTECMVAEAPKDEAAGGGMPGGMGGMGGMGGMDM + 3 Oxidation (M)
☒ 5236	960.9794	1919.9442	1919.9629	-0.0186	0	7	3.9e+02	1	YSEITFPILSPDPATNR
☒ 6889	992.7700	2975.2882	2975.3865	-0.0983	1	7	1.9e+02	1	QIFNTQMSQSRDAAYSTAGAAMLDNLR + Oxidation (M)
☒ 7495	727.1202	3630.5646	3629.6874	0.8772	1	7	1.1e+02	1	MGSLFRSETMCLAQLFLQSGTAYECLSALGEK + 2 Oxidation (M)
☒ 7774	995.9984	3979.9645	3979.9949	-0.0304	1	7	1.1e+02	1	ESGLLGLSGLTNDMRELEAAAAGGNTNAQLALEIFVYR + Oxidation (M)
☒ 7959	1033.0210	4128.0549	4127.0424	1.0125	1	7	95	1	RQMDDILSIATVKPAVLQVECHPYLAQNELIACCR + Oxidation (M)
☒ 7972	827.5541	4132.7341	4132.8381	-0.1040	1	7	75	1	AHPTVGTCSGMFTANTMGSMSEALGMALPGSASPTATSSRR + 3 Oxidation (M)
☒ 7980	831.5353	4152.6401	4152.2003	0.4398	1	7	67	1	AGFPIVQMCNLIPVASTVGANKIVPTISIPYPLGDPSTSK
☒ 8009	1397.3331	4188.9775	4188.0593	0.9182	0	7	97	1	LLTQLSLWWFDQLSDIIDNHVISDTPPPGAPDDWAGR
☒ 8444	904.2252	4516.0896	4515.9988	0.0908	1	7	71	1	ACAQGTFKPLSGEGSCQPCPANSHSNTIGSAVCQCRVGYFR
☒ 6761	719.3319	2873.2985	2872.3987	0.8998	0	7	2.2e+02	1	EVLANMGLSLGMSVSGWPPENIEDLAK + Oxidation (M)
☒ 6942	500.2587	2995.5085	2994.4824	1.0262	1	7	2.1e+02	1	SSMGMAISAVKTGEADAALSAGNTGALMAIAK
☒ 3421	648.0247	1294.0348	1294.5864	-0.5515	0	7	3.5e+02	1	DMGTTWTEAVGK
☒ 4216	757.3143	1512.6140	1511.8671	0.7469	0	7	4.5e+02	1	VGAVTLIQESLGAVR
☒ 6223	870.3799	2608.1179	2607.1945	0.9234	1	7	2.3e+02	1	LVNELTEFAKTCVADESHAGCEK
☒ 7668	955.2333	3816.9041	3815.8141	1.0899	1	7	1.2e+02	1	GNASGMAVGLPEGQMGNSEVGHLNMGAGRIVYQELTR + Oxidation (M)
☒ 8679	995.4625	4972.2761	4973.1695	-0.8934	0	7	51	1	MAAAGAPDGMEEPGMDTEAETVATEAPARPVNCLEAEAAAGAAAEDSGAAR + Oxidation (M)
☒ 2420	362.2203	722.4260	722.3017	0.1243	0	7	57	1	EGGGTMR + Oxidation (M)
☒ 5860	1219.1234	2436.2322	2435.2162	1.0161	1	7	2.9e+02	1	CGTSPMHAHLNELTNLSLRIR + Oxidation (M)
☒ 5902	1236.5461	2471.0776	2470.1911	0.8866	1	7	2.6e+02	1	RYNISGITPGDDYAAMNQVLTR + Oxidation (M)
☒ 7422	892.9080	3567.6029	3567.8153	-0.2124	1	7	1.4e+02	1	QALGIGLFQCLALWPGFSRSGATISGGMLMGISR + Oxidation (M)
☒ 8462	647.8593	4527.9642	4527.9874	-0.0232	1	7	65	1	VASTMTDGANTMIEHDDTLPSQLGTMVINTEDEEEEGTMKR + 2 Oxidation (M)
☒ 8467	755.6688	4527.9691	4528.3393	-0.3701	1	7	66	1	FIGILTEEYAGFFPTWLAPQQVVVMNITDGQADYVQKLVK
☒ 8861	979.7809	5872.6417	5873.1080	-0.4662	1	7	27	1	DKPSESFICIVLSMVLVSAFFTDIIGIHPIFGGFLVGTIIPHENDLTVKITEK + Oxidation (M)
☒ 4287	769.9291	1537.8436	1537.7961	0.0476	0	7	4.7e+02	1	GLEPSGASRPNLGQR
☒ 6768	960.7977	2879.3713	2879.4315	-0.0602	1	7	2.3e+02	1	GWPVDLVPNGSAGSAATSWDSPNARVLR
☒ 7841	673.9674	4037.7607	4037.8874	-0.1266	0	7	90	1	SLGLSPSEAEVADLMNEIDVDGNHAIEFSEFLALMSR + 2 Oxidation (M)
☒ 5521	697.6815	2090.0227	2089.0408	0.9818	1	7	3.7e+02	1	MREIVLTQAGQCGNQIGAK + Oxidation (M)
☒ 7470	722.3421	3606.6741	3606.6128	0.0613	0	7	1.4e+02	1	MYGDAQAAQGAEAAAAQAAAGGSAGAAAQDDNVVDAEVK + Oxidation (M)
☒ 7654	955.2316	3816.8973	3815.8016	1.0957	1	7	1.2e+02	1	QETIAAEDVLQDMGIFSMTSSDSQAMGRVGEVLIR + 2 Oxidation (M)
☒ 7836	673.9652	4037.7475	4037.8369	-0.0894	0	7	89	1	AAGPAGMNSNIWLNSSNVGNNSGNQSGISAPTTTSTNTSSR + Oxidation (M)
☒ 4377	782.0085	1562.0024	1561.8212	0.1812	1	7	4.1e+02	1	RDIIASAYEAAVQR
☒ 8152	1076.4966	4301.9573	4302.9752	-1.0179	0	7	82	1	LENSEVTIEMDDPQQAAAGNNNMLGQMGIDLGDSLGALMPK + Oxidation (M)
☒ 8394	1129.5242	4514.0677	4513.2410	0.8267	1	7	71	1	NEAEEQFGFLIDALAMGAPPHGGIAFGLDRIVMLLANEDSIR + Oxidation (M)
☒ 7250	564.7729	3382.5937	3383.7573	-1.1635	1	7	1.6e+02	1	NVHLTDTGINVVEKALGGVDLFSAEDTTLLSR
☒ 7486	726.7173	3628.5501	3628.7694	-0.2193	0	7	1.2e+02	1	ANDTIPVAMKPLGDYQFDPCASYLLIGGFGGIGR + Oxidation (M)
☒ 8737	951.2913	5701.7041	5700.8542	0.8500	1	7	34	1	RMFAYSTISHMGFMLLGVLAGSQNGYGAAMFYVLVYTLMTLGGFGMILLLSR + 2 Oxidation (M)
☒ 6789	965.7664	2894.2774	2894.5654	-0.2880	1	7	2.1e+02	1	RYLPAGALADVGSGAGFPGLVLALIEPDR
☒ 7079	781.1077	3120.4017	3121.5312	-1.1295	1	7	1.8e+02	1	YSVITVTSVHTMDTNISEITSRYLTMK + 2 Oxidation (M)
☒ 7234	838.8680	3351.4429	3350.8099	0.6330	1	7	1.4e+02	1	NLSLTSSAPPLPSPGRSGPLPPPPSERPPPPVR
☒ 7863	811.3749	4051.8381	4052.9587	-1.1205	1	7	1e+02	1	ESYSAMHFHADALHAMADITASGRIPLLVGGTMLYYK + Oxidation (M)
☒ 8627	671.8592	4695.9635	4696.3450	-0.3815	1	7	49	1	VPNIDQAIISVHCHDDLGMSVANSIAAVQAGARQIEGTINGIGER
☒ 3716	703.3364	1404.6582	1403.6901	0.9682	0	7	5.6e+02	1	NPIASMMAVEGLR + Oxidation (M)
☒ 4609	819.8310	1637.6474	1638.8148	-1.1673	1	7	4.1e+02	1	RAGSPEVQGAMGSPAPK
☒ 6560	554.0359	2765.1431	2766.3184	-1.1753	0	7	2e+02	1	LAVADKPDSHDICFISHGDTGGWLR
☒ 6667	714.3503	2853.3721	2853.5641	-0.1920	0	7	2.3e+02	1	INNYTVSGIFIVTVVNVLISAFTVDR
☒ 8698	1274.6160	5094.4349	5094.4880	-0.0531	1	7	50	1	EVQMNFLNQLTSVFNPRTVPSPPISPQALVEGENDEQSSPDQVSAAK
☒ 3413	645.1360	1288.2574	1287.5257	0.7317	0	7	4e+02	1	VAMMMGGTSSER + 2 Oxidation (M)
☒ 8181	1090.5067	4357.9977	4357.1029	0.8948	0	7	80	1	SVAVMVGDVGVGGDHPIVVQSMTNTDTADIDATVMQVSALWR + Oxidation (M)
☒ 4820	852.6143	1703.2140	1703.8916	-0.6776	0	7	3.2e+02	1	ISHGFAISVGMVLETK + Oxidation (M)
☒ 7137	806.8548	3223.3901	3223.2660	0.1241	1	7	1.6e+02	1	AGDQTGGAFCMCDVSGGGGECVSGSGGGGESGRK + Oxidation (M)
☒ 7512	910.4741	3637.8673	3636.8221	1.0452	1	7	1.4e+02	1	LGLLEALLKVGDWQHAQNIMDQMPPYYAASHK
☒ 8534	914.6063	4567.9951	4568.1675	-0.1724	1	7	63	1	DVCGGVLTGLSGVLTSPEYPNNYPNSMECHWVIRAAGPAHVK + Oxidation (M)
☒ 2979	519.1368	1036.2590	1035.4848	0.7743	0	7	4.4e+02	1	IYMFGFSR + Oxidation (M)
☒ 3054	1087.1357	1086.1284	1085.5063	0.6221	1	7	4.5e+02	1	MSKETSYPK + Oxidation (M)
☒ 7714	778.3269	3886.5981	3887.7492	-1.1511	1	7	92	1	MSAEEYIEVSSSPDIFTDDDDMITIEPELNKNPK + Oxidation (M)
☒ 7924	822.7798	4108.8626	4107.7772	1.0854	1	7	94	1	STLQHTYMESGDYVFMDMSSYEETRLTAQQIGDSR + 2 Oxidation (M)
☒ 8263	1105.6915	4418.7369	4418.1873	0.5496	0	7	60	1	VMASELVEFEDGTVGIALNLEEDNVGVVLMGAGLGIEEGSTVR
☒ 2518	392.7478	783.4810	782.4762	1.0048	1	7	3.1e+02	1	AARAAPVK
☒ 6844	978.1014	2931.2824	2930.3492	0.9332	0	7	2e+02	1	QDEIGTPYCVTIDYNTIEDETVTVR
☒ 8440	904.2245	4516.0861	4517.1995	-1.1134	1	7	73	1	LERALGQYMLDLHSTEHGFTEMQVPALVNDATAYGTGQLPK + Oxidation (M)

☒	8615	781.0015	4679.9653	4680.3186	-0.3532	1	7	50	1	MAILGDTAWDFGSLGGVFTSIGKALHQVFGAIYGAAFSGVSWTMK + Oxidation (M)
☒	5151	627.6425	1879.9057	1878.9813	0.9244	1	7	4.2e+02	1	ATVLGHIQRGGQPGAFDR
☒	7301	689.7266	3443.5966	3442.5942	1.0024	0	7	1.7e+02	1	ISADTTDNSGTVNQIMMMANSPEDWLSLLLK + 3 Oxidation (M)
☒	7529	912.3994	3645.5685	3644.8202	0.7483	1	7	1.2e+02	1	SMFPVMVLSAFLNNTPCVTFMIPILMSWARR + Oxidation (M)
☒	8267	633.5659	4427.9104	4427.3969	0.5135	1	7	65	1	LLIFLGLYVLLGILMTNIIMNLPLSTDNPMQMRRPPER + 2 Oxidation (M)
☒	8713	914.6013	5481.5641	5480.6025	0.9616	1	7	39	1	VIEINNGLTMFVMALQGMKPHHAAKSWNFMNFQSANHSAVQMEFTTPK + 3 Oxidation (M)
☒	8108	1066.2218	4260.8581	4261.1187	-0.2606	1	7	79	1	NWGRVPALGTEPMFISDLADAVVESLPYVGAMAVSNLEAR + Oxidation (M)
☒	8516	910.2012	4545.9696	4546.3281	-0.3585	1	7	65	1	TTTGDGIIAGLQVLSAMVGNHMNLHDLCSGMRLLPQILVNVR + 2 Oxidation (M)
☒	5019	605.0066	1811.9980	1811.7865	0.2114	0	7	4.3e+02	1	HMMDLACLQEQQHR + Oxidation (M)
☒	5029	911.0032	1819.9918	1819.9655	0.0264	1	7	4.3e+02	1	GTYKALGHPVMINYLK + Oxidation (M)
☒	6591	702.5609	2806.2145	2807.3523	-1.1378	0	7	2.2e+02	1	NIGDHIGMMSTLINGLFIYNHFER + Oxidation (M)
☒	7735	658.2792	3943.6315	3944.0032	-0.3716	1	7	87	1	LASQMPLENTTVQQMVFMLLSNLALSHDCKGVIQK
☒	8634	945.2200	4721.0636	4721.3384	-0.2748	1	7	56	1	LRHMHLVSVLGHCIGTYQDHHPYAGSTIFIVQEYISNGSLR + Oxidation (M)
☒	6036	629.5638	2514.2261	2514.3734	-0.1473	0	7	2.9e+02	1	LEDGLVFPIPTNIQVIPEPQPAK
☒	6912	749.0863	2992.3161	2991.6718	0.6443	1	7	2e+02	1	SLYPMDALSLIGFLEILSKGLSILNIR + Oxidation (M)
☒	7594	745.7798	3723.8626	3724.7458	-0.8832	0	7	1.4e+02	1	TEVDGSLYTGALFFILMMLMFNGMSELSMTIAK + 4 Oxidation (M)
☒	7979	1037.9623	4147.8201	4147.1618	0.6583	1	7	85	1	IASGASLDEVLLLLVNYAEEMKPGLLCTVMLLDREQK + Oxidation (M)
☒	8343	639.8570	4471.9481	4473.0584	-1.1103	1	7	67	1	QSSKMGLFTSTTSASSVFPSMTTQLDSSTMVNMPAPSETIPK + 4 Oxidation (M)
☒	8378	1123.2465	4488.9569	4488.2853	0.6716	0	7	68	1	DADGATLDQGIALWFPGPNSFTGEDVLELQGHGGPVILDLLLK
☒	3557	673.8086	1345.6026	1346.5846	-0.9820	1	7	5.8e+02	1	GDEKTICMSYK + Oxidation (M)
☒	7866	1013.9678	4051.8421	4050.8823	0.9598	1	7	1e+02	1	SGRSAAAASTASSASSAASSAAGAAAAAGGPRPTDGGGSGAAASSSGSAK
☒	8423	904.0264	4515.0956	4514.2378	0.8578	1	7	77	1	LFHVPYDQNGWIAKQGVVVDEILTYCMNHPFLNMKPPK + Oxidation (M)
☒	8425	1130.0234	4516.0645	4516.4173	-0.3528	1	7	73	1	GVDPAIVEIALDIAATLLVEIAGGTVDAGRTLVGDVPAMQPITMK + Oxidation (M)
☒	5416	676.6758	2027.0056	2026.9167	0.0889	0	7	3.9e+02	1	YTTSHPNDMADDLIAAHR
☒	7628	945.4312	3777.6957	3776.9117	0.7840	1	7	1.2e+02	1	MLQAESYGADIAKVAVMPASPQDLLVLFQATQEAR + Oxidation (M)
☒	8214	731.3333	4381.9561	4383.1101	-1.1539	1	7	77	1	LAADQLYLVWSNHHIMMDGWSMGVLMKSLFQNYEALR + Oxidation (M)
☒	8458	1132.7479	4526.9625	4526.1171	0.8454	1	7	69	1	DEQIGDLVGEAMSKVGHDGVVSVEESSTLGTELEFTEGIGFDK + Oxidation (M)
☒	8117	1068.4830	4269.9029	4270.9078	-1.0049	1	7	82	1	TDEAPQGAAASSSVAGAVGMTTSGESESDDSEMGRLQALLEAR + 2 Oxidation (M)
☒	8175	872.1708	4355.8176	4356.1848	-0.3672	1	7	67	1	ICSIFEAWGGADWEARLGELASVDLAAVTAADVSPQAIALGR
☒	5851	811.6939	2432.0599	2432.3050	-0.2451	1	7	2.9e+02	1	EKFLIQVLDDSSDESIQLLIK
☒	5897	824.0282	2469.0628	2469.2937	-0.2310	0	7	2.8e+02	1	AHSKPLPSDIAVIMYTSGSTGIPK
☒	7881	811.9199	4054.5631	4053.8949	0.6682	0	7	77	1	WGLDPSLDFFEVTGNCNLGRPNSEGSNQSLMGSVTIR
☒	7903	1021.7326	4082.9013	4083.0493	-0.1480	1	7	1e+02	1	AGTVRTACLVVAMLLSLDFPGQAQPPPPPPDATCHQVR + Oxidation (M)
☒	8102	1062.7582	4247.0037	4248.1911	-1.1874	1	7	95	1	LGLVADEASFWNVHIDSLFFSVFTGMLFLWVFRSVAK
☒	8279	892.4167	4457.0471	4458.1051	-1.0580	1	7	82	1	ELPLPMMNILNGGAHADNNVDIQEFMIMPAGARCFAEALR + 2 Oxidation (M)
☒	5088	930.6072	1859.1998	1858.8956	0.3043	1	7	3.2e+02	1	DTSTSHGRANIIEVMGR + Oxidation (M)
☒	6176	1286.3691	2570.7236	2571.2097	-0.4861	0	7	2e+02	1	NATHLVWATGGGMVPEAEMSADLAK + Oxidation (M)
☒	7915	822.5794	4107.8606	4108.1701	-0.3094	1	7	95	1	ALSELLLRPVTFAPDCLNAADVVSKMSPGDVVLLENVR
☒	6589	560.8950	2799.4386	2799.3280	0.1106	1	7	2.6e+02	1	GSTPRSPARPTVSTGPPMDDLAASMER + Oxidation (M)
☒	7113	799.0652	3192.2317	3192.3304	-0.0987	1	7	1.4e+02	1	GQPDQCMGHSGASTHASCCYAPGLECKVK
☒	7678	955.4790	3817.8869	3816.9295	0.9574	1	7	1.3e+02	1	ILMCHCLAAIAMQLPVLGDGMLGDLMELYKVIGR + Oxidation (M)
☒	7602	625.1096	3744.6139	3745.1329	-0.5190	1	7	1.1e+02	1	ELNKIVAAILFASLIAMMVGFIANILYKPVLEPK + Oxidation (M)
☒	7943	1028.4753	4109.8721	4109.8344	0.0377	1	7	95	1	AKMVYEMALQLIESESTESPESFESPESSESSEAEVK + Oxidation (M)
☒	8133	1074.2654	4293.0325	4293.7930	-0.7605	0	7	90	1	VGMGPGNQMGGMMGGPNQMQGPGAGMVGGMPGQMGVGMGPGGMSGGK + Oxidation (M)
☒	3224	592.3768	1182.7390	1182.5591	0.1800	0	7	5.7e+02	1	LENLFSEMGK + Oxidation (M)
☒	6887	991.4600	2971.3582	2971.3799	-0.0217	1	7	2.2e+02	1	GLTPEMMATLFGGPSDADLMKASMEALR + 2 Oxidation (M)
☒	5259	967.9183	1933.8220	1933.9601	-0.1381	1	7	3.9e+02	1	RSLGSLSVAEPAMIAECK + Oxidation (M)
☒	7785	664.6310	3981.7423	3980.8628	0.8795	1	7	97	1	TMQLMNVPKGATALDVCCGTGDWTIALADAAGETGEIK + Oxidation (M)
☒	3639	688.3158	1374.6170	1373.7224	0.8947	0	7	5.8e+02	1	TLNAMEAALALEK
☒	5939	830.3661	2488.0765	2487.2778	0.7987	0	7	2.7e+02	1	TAALLEAAAVLGVIMGGGTEEEIEK + Oxidation (M)
☒	7622	941.2244	3760.8685	3759.9864	0.8821	1	7	1.4e+02	1	MLGIPISNILMVGNYASDLELDPMKDILILSALR + 2 Oxidation (M)
☒	7920	822.7792	4108.8596	4109.9719	-1.1122	1	7	97	1	SYLRGAFLAGGSVNNPETSSYHLEIFSQNESHAEGLTK
☒	5303	655.3190	1962.9352	1963.9924	-1.0573	1	7	4.1e+02	1	QPLISKAFEITMAAGDEK + Oxidation (M)
☒	7400	708.1285	3535.6061	3534.7137	0.8924	1	7	1.5e+02	1	AQGQHPDVASGRFGADMQVHLVNDGPVTFMLQI
☒	8579	925.9964	4624.9456	4625.4187	-0.4731	0	7	56	1	YYLIPVVTACGLIGFYYGGYWVWLGAATFPALMVLDVILPK + Oxidation (M)
☒	4212	756.6979	1511.3812	1510.8289	0.5523	1	7	3.4e+02	1	TALNIAIDMIKHR + Oxidation (M)
☒	6866	982.4615	2944.3627	2945.5069	-1.1443	1	7	2.3e+02	1	EHLSNRAFCVTDGLSTLQFSPLTKPK
☒	8099	1062.7576	4247.0013	4247.0919	-0.0906	1	7	97	1	VHEMLPCTILGAKPEPKVVTLGNDYDYVWDISAAAGSSK + Oxidation (M)
☒	8126	613.5889	4288.0714	4287.0465	1.0248	1	7	93	1	SLKMAPCIPYLGIYLFDMTYIDSAYPASDSIIETEQR + Oxidation (M)
☒	8538	914.8058	4568.9926	4569.2367	-0.2441	1	7	65	1	RHLTLDELNATSDNTMVAHLGIVYTLLGDDVLEAEMPVDTR + 2 Oxidation (M)
☒	8864	840.0973	5873.6302	5874.7686	-1.1384	1	7	27	1	IIMEYYTHQMPGMDGIPARPVESQGNTTANNMFNPAMFDLLKMLPPGYNLSK + 2 Oxidation (M)
☒	8286	892.4211	4457.0691	4458.1051	-1.0360	1	7	85	1	ELPLPMMNILNGGAHADNNVDIQEFMIMPAGARCFAEALR + 2 Oxidation (M)
☒	8681	1258.5470	5030.1589	5029.2361	0.9228	1	7	43	1	MSLLQKGLGDSNDLMENQLSNDDYSIGFMAAENQNMSLVEEQFR + 3 Oxidation (M)
☒	8917	1021.1549	6120.8857	6120.9148	-0.0291	1	7	27	1	STAMAMAAAASSSAAAATATTAISNSTNTTASPATTNTINTTTKTITTSEVPLEPASNMAPK + 3 Oxidation (M)
☒	4045	735.4342	1468.8538	1469.7361	-0.8823	1	7	5.2e+02	1	ENLAEAAAPAKEEK
☒	4967	898.0421	1794.0696	1793.7389	0.3308	0	7	4.1e+02	1	ATDYGMGMWVYNNEK + Oxidation (M)
☒	5341	996.4460	1990.8774	1990.0483	0.8291	0	7	3.9e+02	1	HGHELLINTLITSGADTAK
☒	5684	752.8325	2255.4757	2256.0515	-0.5758	1	7	2.3e+02	1	GGDTDAQAKAVSNFIMTEVMR + Oxidation (M)
☒	6302	872.0555	2613.1447	2613.3611	-0.2165	0	7	2.6e+02	1	MTYDVLSAIASPIIVALDYTNISK + Oxidation (M)
☒	7275	1136.1449	3405.4129	3405.7668	-0.3539	1	7	1.3e+02	1	EDTTDLFIDVTGTDPVVYRALNIVVTALAER
☒	8910	1018.9889	6107.8897	6107.8930	-0.0032	1	7	29	1	AAAAAFGLPLYRYLGGVYAHVLPVPMMNIMNGGQHATNSTDFQEFMIMPVGADSFR + 4 Oxidation (M)
☒	2057	592.3711	591.3638	591.2500	0.1138	0	7	2	1	EGASAAS
☒	6950	749.8860	2995.5149	2996.3572	-0.8423	1	7	2.3e+02	1	DTLAGFKGLLAGDYDDVPEQAFMYCGK + Oxidation (M)
☒	7118	1065.8077	3194.4013	3194.5813	-0.1800	1	7	1.8e+02	1	TTAAGAGAGANTVTAPMPGTILNVGCHAGDKVSK
☒	7962	1033.2765	4129.0769	4128.0014	1.0754	1	7	1e+02	1	MFEATIANTLMNRYADALIMHGMDIWELMPVLTLR + 3 Oxidation (M)
☒	8136	1074.5109	4294.0145	4294.0187	-0.0042	1	7	90	1	TTDVTGSCELPEGVEMVMPGDNVKMTVSLIAPIAMEDGLR + 2 Oxidation (M)
☒	6692	956.0900	2865.2482	2865.3062	-0.0580	0	7	2.4e+02	1	SPIGSCTQAASSASPFVSAFNTFSMASR
☒	7362	1171.5594	3511.6564	3511.8861	-0.2298	1	7	1.6e+02	1	VVVGLSGGVDSSVAAALLHRQGYAVEGLTLWLMK + Oxidation (M)
☒	7752	793.1331	3960.6291	3959.9775	0.6517	1	7	90	1	VGAAFVPLDFSHPEGHLRAVMQPLNADIILCADNTR + Oxidation (M)
☒	8321	746.1627	4470.9325	4471.2906	-0.3580	0	7	71	1	AMDVALILYAEHEINASTLAVMTVGSTLSDYYSAILAGIGALK + Oxidation (M)
☒	7200	832.1229	3324.4625	3324.7242	-0.2617	1	7	1.6e+02	1	SPDPETDLVSFQVPKSVELPQATFLSQPLR
☒	7259	846.9080	3383.6029	3382.8372	0.7657	1	7	1.7e+02	1	GFLLLSIMGALCAIFWVFKATVGVMGRPLLS + Oxidation (M)
☒	7347	696.7240	3478.5836	3478.5949	-0.0113	1	7	1.6e+02	1	EEMDTTHCMKQNEIISQAVCALLNSGGGVVR + 2 Oxidation (M)
☒	8011	838.9589	4189.7581	4190.9085	-1.1503	1	7	82	1	RAPTSGAMSTQPVTATASETMSMPQLTVPASGSMSMLQMR + 4 Oxidation (M)
☒	7459	899.1657	3592.6337	3592.8234	-0.1897	0	7	1.5e+02	1	HAGDLGNVTAGEDGVANIHVVDSQIPLTGPNSIIGR
☒	7636	764.1884	3815.9056	3816.7434	-0.8378	1	7	1.3e+02	1	LLCGGEALKGDGFDNGAWVAPTVFTDCSDEMTIVR + Oxidation (M)
☒	7773	796.9572	3979.7496	3978.8655	0.8841	1	7	1.1e+02	1	EISDFFESMIVNGADAKLSANWLMGDVSAYLNAEQK + Oxidation (M)
☒	7885	812.1201	4055.5641	4055.8419	-0.2778	1	7	77	1	TDGTPTYNFCVVVDDWDMGITYYVRGEDHINNTPR
☒	7952	688.2897	4123.6945	4123.8885	-0.1940	1	7	77	1	CLSPTEPQAPAASSGPLPDSDQLTHVDTEGRMAMVDVGR + 2 Oxidation (M)
☒	4673	824.6134	1647.2122	1646.7583	0.4539	1	7	3.4e+02	1	QEVICWDRGNTNR
☒	4830	858.8719	1715.7292	1714.8890	0.8403	0	7	4.7e+02	1	AYDLGEPQLSSVAPLR
☒	7528	730.1146	3645.5366	3644.7239	0.8127	1	7	1.2e+02	1	HAEIDTYNDHRVAMCFSLVALSDTPVTINDPK + Oxidation (M)
☒	8095	1062.7555	4246.9929	4248.1911	-1.1982	1	7	98	1	LGLVADEASFWNVHIDSLFFSVFTGMLFLWVFRSVAK
☒	6859	980.8359	2939.4859	2938.5110	0.9749	1	7	2.4e+02	1	QEILKEAEPQAWLQEVSQGMVPALTK + Oxidation (M)
☒	7631	954.9770	3815.8789	3815.9967	-0.1178	1	7	1.3e+02	1	IMLSPHPYALRSHLCVGEETASLGVYLHSKPVVR
☒	7650	955.2314	3816.8965	3815.7238	1.1727	1	7	1.3e+02	1	KPFWETKSLNQMTVPEWESLCDGCGLCCLVR + Oxidation (M)
☒	7919	1028.2212	4108.8557	4109.8344	-0.9787	1	7	99	1	AKMVYEMALQLIESESTESPESFESPESSESSEAEVK + Oxidation (M)
☒	7922	822.7796	4108.8616	4109.7399	-0.8783	0	7	99	1	YDQFGHAGVGSSAASGGGQYGAGASDFSDIFSAFNDMFGGGK + Oxidation (M)
☒	7416	595.1020	3564.5683	3564.7130	-0.1446	1	7	1.5e+02	1	AFIVHKMPIPGPLFATEEECAGVDQVHGSQER + Oxidation (M)
☒	7614	940.9730	3759.8629	3759.9864	-0.1235	1	7	1.5e+02	1	MLGIPISNILMVGNYASDLELDPMKDILILSALR + 2 Oxidation (M)

☒	7157	814.1187	3252.4457	3252.5577	-0.1120	1	7	1.8e+02	1	IASTVGRAAPGMVGVGAMEAGGAYQQTADEIMK + Oxidation (M)
☒	7336	870.4025	3477.5809	3478.6391	-1.0582	1	7	1.6e+02	1	EAAQKGAQIVALPECFNSPYGTTYFPEYAEK
☒	7709	969.7351	3874.9113	3873.7514	1.1599	0	7	1.3e+02	1	SEGVIPMSEFIDSSEGLSVGAEVEVYLDQTEDEEGK
☒	7834	1346.5995	4036.7767	4036.9676	-0.1909	1	7	1e+02	1	TSPAAAAEAFWRAAMPGAPMPDAIVELLHHEHGVASAGGK + Oxidation (M)
☒	8096	1062.7561	4246.9953	4248.1911	-1.1958	1	7	1e+02	1	LGLVADEASFWNVHIDSLFFSVFTGMLFLWVFRSVAK
☒	8125	613.4462	4287.0725	4288.1402	-1.0678	0	7	96	1	TNEIGMTLTAGMAVNLLSGESRPLSQLHDVVAMAGIGHPPR + 3 Oxidation (M)
☒	8273	889.9891	4444.9091	4444.1996	0.7096	1	7	71	1	IESASLQGSDRVLIASSNLLEPSGITIDYLTDTLYWCDTK
☒	8611	1168.8004	4671.1725	4672.3407	-1.1682	1	7	72	1	IEPGAIIRDMVEIGEGAVIMMGAVINVGAVIGEGTMIDMNAVVGGR + 3 Oxidation (M)
☒	1946	558.8586	557.8513	558.2874	-0.4361	0	7	0.5	1	GGGAAAR
☒	3426	648.3207	1294.6268	1295.5881	-0.9613	0	7	5.8e+02	1	LYSGTAPDTDEK
☒	4179	750.1333	1498.2520	1497.7173	0.5347	1	7	3.5e+02	1	DMAKLEFAAPDFK + Oxidation (M)
☒	7615	940.9758	3759.8741	3759.9512	-0.0771	0	7	1.5e+02	1	YSVDIFPHSPFLSNASSAITAGAISTVATNPIWVVK
☒	8590	928.0336	4635.1316	4634.0044	1.1272	1	7	68	1	GLLENEEVTIEVEQAPKANPMGDMMGQMGMDMSSMLGDMLPK + 5 Oxidation (M)
☒	6693	574.0579	2865.2531	2864.2970	0.9561	0	7	2.4e+02	1	NFSGGILGGISCNMDYPLNLNHENGR + Oxidation (M)
☒	7425	1192.5487	3574.6243	3575.5423	-0.9180	0	7	1.5e+02	1	TSMPWSNDYTEIPTNASGGNSYFQSSEFSLSR + Oxidation (M)
☒	8164	865.6028	4322.9776	4322.1306	0.8470	1	7	93	1	MNQILLCSPILLLLFTVASCDSEQQLDSAMHLKSDSTK + Oxidation (M)
☒	8524	913.7899	4563.9131	4564.1207	-0.2076	0	7	61	1	NEEEEMLNSDASEAMLTVSSVPSPTLTENESVNESVVPNILK + 2 Oxidation (M)
☒	8921	1225.3898	6121.9126	6121.8727	0.0399	1	7	29	1	VIPAADLSQQISTAGTEASGTGNMKFMLNGALTIGTMDGANVEMAEEAGEENLFIFGMR + Oxidation (M)
☒	6023	836.7144	2507.1214	2506.3505	0.7709	1	7	2.9e+02	1	WLLSQEVIYDTILNMVKGQIK + Oxidation (M)
☒	7946	1372.2295	4113.6667	4113.0609	0.6058	1	7	81	1	AMRVLAMVVAVYTICFLPSIIFGMASMVAFWLSACR + 2 Oxidation (M)
☒	8420	904.0259	4515.0931	4515.0006	0.0925	1	7	81	1	SETETMNVNGPQDFYSDSPFCLEASFSSSDLLQNETKNVK
☒	8572	661.4282	4622.9465	4622.0281	0.9184	0	7	58	1	MMITGDNVENSEEALNLATNYECFTSTVGVHPCQAQCFLR + Oxidation (M)
☒	7665	955.2332	3816.9037	3815.9967	0.9070	1	6	1.3e+02	1	IMLSPHPYALRSHLCVGEETASLGVYLHSKPVVR
☒	8489	1133.2512	4528.9757	4527.9874	0.9883	1	6	72	1	VASTMTDGANTMIEHDDTLPSQLGTMVINTEDEEEEGTMKR + 2 Oxidation (M)
☒	8714	927.6463	5559.8341	5559.9090	-0.0749	1	6	41	1	MNPLIFIMLILTIILGTSIILTSTHWFMIWLGFEMNMMAMIPVLMKK + 4 Oxidation (M)
☒	7008	1006.8008	3017.3806	3017.5314	-0.1508	0	6	2.2e+02	1	ELAGNTQNLAFISGMIASVPGVAALMSAPR + 2 Oxidation (M)
☒	7023	606.4666	3027.2966	3026.4080	0.8887	0	6	1.9e+02	1	LMEAAAANAGFSLNSDSHQAINFFADEIK + Oxidation (M)
☒	8899	992.6249	5949.7057	5949.8865	-0.1808	1	6	28	1	VPVVVDAGIGTPSEAAQAMEMGADAVLINSAIAMAANPVAMAQAMGMATQAGRLAYLSGR + 4 Oxidation (M)
☒	6825	974.4491	2920.3255	2921.3535	-1.0280	1	6	2.3e+02	1	EMGCRLIESVSVISYAGEEGTQHEPK + Oxidation (M)
☒	7815	804.3375	4016.6511	4017.8070	-1.1559	1	6	85	1	AICYARFMVPEPSIFEIDAEAEEAADAEGMADIAAGR + 2 Oxidation (M)
☒	7875	1351.6228	4051.8466	4051.7437	0.1029	1	6	1.1e+02	1	GTSTNQFTFQTYNDNSCSQATSDQALSFTCSDNVKK
☒	8304	892.8204	4459.0656	4458.9861	0.0796	1	6	84	1	ADTPSDMHDPMYMLVNLAIGGMAGPPTDGLMGGAEMKVDYVK + 4 Oxidation (M)
☒	8421	904.0260	4515.0936	4515.2058	-0.1122	1	6	82	1	LNQIGAGDQGLMFGYATNETEELMPLPIMLSHHLMQRIAK + 3 Oxidation (M)
☒	8747	957.4548	5738.6851	5739.6926	-1.0075	1	6	37	1	IIKDGLWENNGVLCMLLGMCPTMAMTGTATNGLGMGLATAAVMAASNLMVAMFR + 4 Oxidation (M)
☒	4604	818.0444	1634.0742	1634.9355	-0.8613	1	6	3.9e+02	1	ISYVASTLSAVRAAVK
☒	4373	780.3192	1558.6238	1558.7012	-0.0774	0	6	5e+02	1	FAPGGVGDSGPGEEQR
☒	5989	833.7084	2498.1034	2499.2210	-1.1176	1	6	3e+02	1	SSDGSRSMTAGLGGFMVGALAGSVLR + Oxidation (M)
☒	6564	692.2940	2765.1469	2764.2140	0.9329	1	6	2.2e+02	1	MAMEAAAERSAGAGAAATAAPESGGGGAGER + 2 Oxidation (M)
☒	7861	1351.6168	4051.8286	4052.8821	-1.0536	0	6	1.1e+02	1	CPEQEVSVNHLFTVASCSYFVFAMLGGIMLDFAGPK + 2 Oxidation (M)
☒	5030	911.4164	1820.8182	1819.9289	0.8894	1	6	4.6e+02	1	DANPARPNVNGASNAPKK
☒	6972	1000.7943	2999.3611	2998.5330	0.8280	1	6	2.2e+02	1	GINGYKVPMGVSMVLGDCLFQLGAITVK + 2 Oxidation (M)
☒	2333	662.8758	661.8685	662.3170	-0.4485	0	6	28	1	GVGAGMR + Oxidation (M)
☒	4951	595.2718	1782.7936	1783.8047	-1.0111	0	6	4.6e+02	1	ISNNYGGAGVMITGDDGK + Oxidation (M)
☒	7268	1135.4801	3403.4185	3403.6516	-0.2331	1	6	1.4e+02	1	FGIEQFAAVMGGSLGGMQALAWSLMYPDRLR + 2 Oxidation (M)
☒	7450	719.5320	3592.6236	3592.7508	-0.1272	0	6	1.6e+02	1	WTTAVLDVEADGGLAVVQSDQGFLLDTNGAMFPR
☒	7867	811.3757	4051.8421	4052.9587	-1.1165	1	6	1.1e+02	1	ESYSAMHFHADALHAMADITASGRIPLLVGGTMLYYK + Oxidation (M)
☒	8873	848.0992	5929.6435	5929.6973	-0.0539	1	6	28	1	ISESAAKASEQLVTGATQESATFSNDGLSGTMALSVQDTVGQEVLQMSDMMGSLSDR + 4 Oxidation (M)
☒	7111	798.5646	3190.2293	3190.5005	-0.2712	1	6	1.5e+02	1	IAPYVAHNFSKLFSMENWGGATFDVAMR + 2 Oxidation (M)
☒	8288	892.6189	4458.0581	4457.0654	0.9927	1	6	85	1	FFQERGFVYTNTPIITGSDCEGAGEMFQLTTMDLNNIPK + Oxidation (M)
☒	8481	906.6083	4528.0051	4527.3407	0.6645	1	6	76	1	FRAIADEVGAYLMVDMAHIAGLVAAGLHPNPVGIADVVTSTTHK
☒	2688	925.9485	924.9412	925.4691	-0.5279	0	6	4.1e+02	1	SIHPDMVK
☒	4172	748.6680	1495.3214	1495.8035	-0.4820	1	6	3.7e+02	1	VLTSYAAPKVYER
☒	5808	1197.8958	2393.7770	2394.0315	-0.2544	1	6	2.4e+02	1	QDGAAAMEMQPLKSAEGGEADEK + 2 Oxidation (M)
☒	8265	1106.5150	4422.0309	4422.1658	-0.1349	1	6	85	1	LFSGASTEFGKPQNSNVSLVDAAMPGMLPVINEECVKQAVR + 2 Oxidation (M)
☒	2605	860.5813	859.5740	860.4028	-0.8288	0	6	8.9e+02	1	YEIDGHK
☒	7770	796.7534	3978.7306	3978.8728	-0.1422	1	6	1.1e+02	1	EVNADIQRIVDDMFDTMYAEEGIGLAATQVDIHQR + Oxidation (M)
☒	2311	647.3940	646.3867	647.2809	-0.8942	0	6	26	1	GAGACGR
☒	3503	659.6721	1317.3296	1316.6208	0.7088	0	6	4.3e+02	1	GDGPDSDIVLSSR
☒	8153	1076.4973	4301.9601	4302.8612	-0.9011	1	6	92	1	MMQMLQGQGGGGGGGLPGLGGMDLQSMMSQMSGLMGGGGGGGGRGR + 3 Oxidation (M)
☒	3610	682.2025	1362.3904	1361.8179	0.5726	0	6	4.2e+02	1	IIIVIFLMCLK
☒	4957	894.4117	1786.8088	1786.9797	-0.1709	1	6	4.9e+02	1	VLDMGCGTGILAILARK
☒	7489	726.7203	3628.5651	3628.7119	-0.1468	1	6	1.4e+02	1	EFSRTVFVNCNLESLISPDGWMPWNGDFALK
☒	7492	605.9351	3629.5669	3629.7052	-0.1382	1	6	1.4e+02	1	MRALNGTTMYTNPPGAEEATLDTVMEFGVLGSPK + 2 Oxidation (M)
☒	7698	968.4592	3869.8077	3869.9047	-0.0970	0	6	1.4e+02	1	AEALPGVVHVITHLNCPDIYYTPGGQSAPEPSPLDR
☒	8048	1060.0071	4235.9993	4236.6485	-0.6492	1	6	1e+02	1	DGDSLDNDGDSSSADCMHESYRNSMQIGVEEGGSNMENK
☒	8459	755.5021	4526.9689	4526.2136	0.7553	1	6	75	1	VAADTEALRAANGGDATVPMNLVTASGSGLDPHITPEAAYFQVGR + Oxidation (M)
☒	8840	975.7788	5848.6291	5847.6866	0.9425	1	6	32	1	MMHQEYLQGLATNAGHPMSLTHHQWLPNPASDWGSGSHLGAQAEHGKSGVQSSR + 2 Oxidation (M)
☒	5196	631.6667	1891.9783	1892.9010	-0.9227	1	6	4.6e+02	1	AIQSNGMVNSQETQSKR + Oxidation (M)
☒	5203	950.3916	1898.7686	1897.8265	0.9422	1	6	3.8e+02	1	RSYDEHSTSWMDQLK + Oxidation (M)
☒	7094	789.1027	3152.3817	3153.4015	-1.0198	1	6	1.9e+02	1	VVMVIDGNSEDDVYMMDIFTEIMGRDK + 2 Oxidation (M)
☒	8264	1106.2665	4421.0369	4420.9588	0.0781	1	6	87	1	MDENNHGVSSSSLPPFLTKTYEMVDDSSSDSIVSWSQSNK + Oxidation (M)
☒	8556	767.0385	4596.1873	4596.2583	-0.0710	1	6	82	1	LDTPNATMNPDAVLVQSLMAKMLHNGTESLVMEASPQELALGK + 2 Oxidation (M)
☒	5441	1021.3363	2040.6580	2040.0528	0.6053	0	6	2.9e+02	1	EQTAEKPVPALSSTAWTPK
☒	6566	692.2942	2765.1477	2766.2595	-1.1118	1	6	2.3e+02	1	EFDNFQFVNFTEIMTRNSPESAK + Oxidation (M)
☒	8131	1074.2618	4293.0181	4293.1223	-0.1042	0	6	97	1	QLLIVSQLTIADQAVEAPAEATQFDGVAVSVAHAEGDVCDR
☒	8422	753.5232	4515.0955	4514.1644	0.9312	1	6	84	1	SGQMMLAQGLLLHLMPTDWRWSDCHALTDVDFEVLKPR + 3 Oxidation (M)
☒	8645	1185.0153	4736.0321	4737.2136	-1.1815	1	6	61	1	DIKSMLQQDMTGYSQGTLDAGEQESVGVITDYVHSGLTEFIMK + Oxidation (M)
☒	7490	908.1496	3628.5693	3628.7839	-0.2146	1	6	1.4e+02	1	GVIQVYDLGHDGQGMSRVASVSQNAIVCAAGNIAR + Oxidation (M)
☒	7853	1010.7007	4038.7737	4038.8722	-0.0985	1	6	1e+02	1	EGVVGSIQDMGAAGITSSAFEMSASGGTGMDLHLDRVPTR + 2 Oxidation (M)
☒	6902	995.7903	2984.3491	2985.4093	-1.0602	1	6	2.3e+02	1	ELEEISMNAMASSSREMINVSLEVLR + 3 Oxidation (M)
☒	6553	922.3881	2764.1425	2764.3088	-0.1663	1	6	2.3e+02	1	CVTEAWAEIPEELEMDRVFTVPK + Oxidation (M)
☒	6555	692.0433	2764.1441	2763.4113	0.7328	1	6	2.3e+02	1	FLASDKPQEMANASLTLETPLTSKR + Oxidation (M)
☒	7871	1013.9683	4051.8441	4052.8821	-1.0380	0	6	1.1e+02	1	CPEQEVSVNHLFTVASCSYFVFAMLGGIMLDFAGPK + 2 Oxidation (M)
☒	8127	1431.6777	4292.0113	4293.0122	-1.0010	1	6	97	1	MGRFLFVSFGLLVVFLSLSGTAADCPSDWSSHEGHCYK + Oxidation (M)
☒	8246	1104.2380	4412.9229	4414.0682	-1.1453	1	6	79	1	NETDGRMFSPAIGIVEDPVTGNANGPMGAWLVHHGLMAHDGK + Oxidation (M)
☒	6890	993.1237	2976.3493	2975.5004	0.8489	0	6	2.3e+02	1	MPIPPPPPPPPGPPPPPTFHQANTEQPK + Oxidation (M)
☒	7503	910.4728	3637.8621	3637.7168	0.1453	1	6	1.6e+02	1	YFGDGFQYIPGNHGGGAYHHEFTDLQWLKVGK
☒	7552	738.5269	3687.5981	3688.6080	-1.0099	1	6	1.3e+02	1	AATSYMTPPSGDLDMALGGGGYHTSSPRSAADAGEMK + 2 Oxidation (M)
☒	8439	1130.0287	4516.0857	4516.2717	-0.1860	0	6	82	1	DVCAATSAPVVASGGVTTLADIEALMELVPVGVEGAIAGTALYEGR + Oxidation (M)
☒	8678	1230.6984	4918.7645	4919.5741	-0.8096	1	6	49	1	QEPQLGTGHAVQQATPALAGDGTVVVLSGDVPLTQAATLRALVEAGAGER
☒	4248	766.0167	1530.0188	1529.8929	0.1259	1	6	4.5e+02	1	AAGLLVLEGYIRQK
☒	7984	693.3687	4154.1685	4153.1690	0.9995	1	6	1.1e+02	1	AMVTDLLNGLNAELEGVEGVDVVVAPPAMYLDLAERLIK + Oxidation (M)
☒	8252	631.5682	4413.9265	4413.1264	0.8001	1	6	81	1	SMAIGFINGTGLKQGAVATSVGHDAHNMSVIGTNDADMVVAANR + Oxidation (M)
☒	8297	892.6205	4458.0661	4458.4782	-0.4120	1	6	88	1	DQLKVPMTSFLILSVFMILMPILLMNLLIGLAVGDIESVR + Oxidation (M)
☒	8863	840.0972	5873.6295	5874.3566	-0.7271	1	6	29	1	ASNFDSDDEMDENEIWSALVKSRPDVEDDSDDSELDFAEDDFSDSTSDDEPK
☒	6994	1002.7889	3005.3449	3006.5095	-1.1646	1	6	2.3e+02	1	NAQVTPMMAQYLEIKAAHPDALLFYR + Oxidation (M)
☒	7142	1080.4908	3238.4506	3238.3724	0.0781	1	6	2e+02	1	YGTLEEDLEEESEDEYESPEEDSIFKK
☒	7877	1014.2179	4052.8425	4051.8233	1.0192	1	6	1.1e+02	1	CSDMVGKLVEFLGIDGVLITEEGYGNPDTDLMMNCK + 2 Oxidation (M)
☒	2441	368.1871	734.3596	734.3381	0.0216	0	6	1.6e+02	1	EAAAAMR + Oxidation (M)
☒	7568	743.5375	3712.6511	3711.8375	0.8136	1	6	1.4e+02	1	LTIAEAPDAFVLTTEVEIDPSSNKALMGLYMSGGR + Oxidation (M)

8028	841.7754	4203.8406	4204.0470	-0.2063	1	6	94	1	FARINQIINEDLHSAFSMPDDQFNVLLANMIAGAQER + Oxidation (M)
8501	907.6253	4533.0901	4532.3130	0.7771	1	6	79	1	EAIWTRDAYVPTLNEYMENAYVSFALGPIVKPAIYFVGPK
8591	928.2313	4636.1201	4635.2486	0.8715	1	6	72	1	SNPGPGANGNNGPLRSVVAMSANTPQVMVVTSDGNFYVFNIDLSK
8701	1289.3346	5153.3093	5154.2725	-0.9632	1	6	48	1	MPPRPNYTMPNMQTAGMGNLMGMEKQYQMNYKPMPNMAQGQMLR + 3 Oxidation (M)
4755	844.5181	1687.0216	1686.8723	0.1494	0	6	4.9e+02	1	RPLVEASTAVAAAMER + Oxidation (M)
5514	693.5329	2077.5769	2078.1021	-0.5252	1	6	2.8e+02	1	NHQWTEGLLSAAKAVGVAAR
5976	625.2833	2497.1041	2496.1156	0.9885	1	6	3.1e+02	1	RTDCGTIQGISVNTQNGMMPER + 2 Oxidation (M)
6497	677.5367	2706.1177	2705.1722	0.9455	0	6	2.4e+02	1	ELSESNQEELGSQVMAEGPGESQDR
8502	909.7979	4543.9531	4544.2986	-0.3455	1	6	72	1	HLPRVSYITAMDLFVSVCFIFVFAALMEYATLNYLVGNK + 2 Oxidation (M)
3484	654.1403	1306.2660	1305.7405	0.5256	1	6	4.2e+02	1	SYTSRKPLVQK
7242	844.3523	3373.3801	3372.4836	0.8965	1	6	1.4e+02	1	GMETELDVVEGMQFDRGYLSQYMVTDSEK + Oxidation (M)
8354	748.3269	4483.9177	4483.0489	0.8688	0	6	73	1	MLPVPMMNIINGGMHADNPIDFQEFMIMPVGADSFSEALR + 3 Oxidation (M)
8475	906.6020	4527.9736	4527.9874	-0.0137	1	6	77	1	VASTMTDGANTMIEHDDTLPSQLGTMVINTEDEEEEGTMKR + 2 Oxidation (M)
4687	827.1954	1652.3762	1651.8240	0.5523	0	6	3.6e+02	1	GTVNTYPMTVDVALR + Oxidation (M)
7364	1171.8916	3512.6530	3512.5678	0.0852	1	6	1.7e+02	1	AQAAFVESWADAVEDTIPKEEDLADGMDGYNR
6156	852.7166	2555.1280	2555.1395	-0.0116	1	6	3e+02	1	WNMGWMNDLLSFMEKESIHR + 2 Oxidation (M)
7474	903.4117	3609.6177	3610.8051	-1.1874	1	6	1.6e+02	1	MGLNLMVDEKLNIEVDPSTIQNFFAPYELVR + Oxidation (M)
7736	789.7368	3943.6476	3943.8576	-0.2100	1	6	1e+02	1	MLKDNDIDISGLQLASTAHVTMPYHCVIDAAMEER
8066	850.0063	4244.9951	4243.9737	1.0214	1	6	1.1e+02	1	SVTVPEAENPRDSLVLPPTQSSSDSSPPEVSGPSSSQMSMK + Oxidation (M)
8630	1175.5402	4698.1317	4698.2179	-0.0862	1	6	69	1	FIDEINSVTAEKLIYNCAVEMVQSAALDEMFQQTEDIVYR + Oxidation (M)
3677	694.5840	1387.1534	1386.6422	0.5112	1	6	4.2e+02	1	RTNGGNTTMGHNK
7462	899.1666	3592.6373	3593.7269	-1.0896	1	6	1.7e+02	1	VQDSEKPGPAPKLMLPSSVFASEFEESEGMLNK + Oxidation (M)
7942	822.9815	4109.8711	4109.9813	-0.1102	1	6	1.1e+02	1	FGVLSPNEIRQMSVTAIITSEVYDEDGTPIEGGVMDPK + Oxidation (M)
8566	925.3932	4621.9296	4621.2185	0.7111	1	6	63	1	WEASISEYNIALGCLPGDVVVAAAFMSYAGPFPSEYRDELVK
8918	1225.1863	6120.8951	6121.9857	-1.0906	1	6	30	1	SENPDLSYEISMQPYNAFQPDGVILFSDILTPLPGMGIDFDIIESKGPQINDPIR + Oxidation (M)
2554	806.2725	805.2652	805.4004	-0.1352	0	6	5.7e+02	1	IDLMGNK + Oxidation (M)
5432	679.9938	2036.9596	2036.9724	-0.0129	0	6	4.3e+02	1	MNSLVDPDIIDALYEASR + Oxidation (M)
6209	865.3874	2593.1404	2592.4747	0.6657	0	6	2.9e+02	1	MAATMQALLVIALLHLATATPVVTK + Oxidation (M)
7186	1103.8248	3308.4526	3309.4691	-1.0166	1	6	1.8e+02	1	ALDEQTQLTISSYRSSSSGSMASEDSTNWR + Oxidation (M)
8504	909.7997	4543.9621	4543.2397	0.7225	1	6	74	1	GSELEITLTKGSCVPGPACAYVNLNICANGTEQGGVLLLENPK
8868	1175.8121	5874.0241	5874.0816	-0.0575	0	6	34	1	ILLITASIFMMGISLTISGLLPQSGFFIFVVCSAIMGLSVPFYSGVQTALFQEK + 2 Oxidation (M)
3207	584.6393	1167.2640	1167.7227	-0.4586	0	6	4.3e+02	1	EALLIAIQVAK
6454	889.7810	2666.3212	2665.4448	0.8764	1	6	3.1e+02	1	GLFMTTNPVPVKAALGMLGLNVGPPR + Oxidation (M)
8565	661.2822	4621.9245	4621.1815	0.7429	0	6	63	1	DKPSGINMEGEFLTTASMVSILPQDSSLPCIHFFTGTPDPER
8646	948.4207	4737.0671	4737.3791	-0.3120	0	6	65	1	TLHDDDSCQVIPVLPHVMVMLIPGQTLPLQLFHPQEVSMVR + 3 Oxidation (M)
7840	673.9674	4037.7607	4037.8598	-0.0991	1	6	1.1e+02	1	DVGFDGIYGNSMDAVSDRDFVLEALFGGSTIMMHLSR + Oxidation (M)
8551	766.1697	4590.9745	4590.0479	0.9267	0	6	70	1	NEGMDATHNPEFTSIEVYQAYADYQDIMDLTEGIIQHTAK + 2 Oxidation (M)
8660	966.2581	4826.2541	4826.1933	0.0608	0	6	69	1	AYAEGLAWVLEYYFQGCPSWEWFYPYHYAPFAADFVDLGK
6629	940.4447	2818.3123	2817.1900	1.1223	1	6	2.8e+02	1	FGADNIMNMMDRLGMEEDMPIESR + Oxidation (M)
7119	799.6098	3194.4101	3193.5608	0.8493	0	6	2e+02	1	GQFFEIQAESIEVVGWVENPDTYPIQAK
7469	902.6620	3606.6189	3607.6995	-1.0806	1	6	1.6e+02	1	TEQMLSLPAEVSSNNLEPAERGASAAQVDMGPHR + Oxidation (M)
7914	822.3070	4106.4986	4106.0037	0.4949	0	6	79	1	TADVTGEIVDLPAGVEMVMPGDNVEMTIELIYPVAIEK + 3 Oxidation (M)
7481	724.3121	3616.5241	3617.6762	-1.1521	0	6	1.4e+02	1	LIDVQMENLTMPQCPLYEEVLDTQMFGLSR + 3 Oxidation (M)
8510	758.4996	4544.9539	4543.9840	0.9699	1	6	74	1	TALQNAASVASLMLTTDCAVAELPKDDAAPAMPGGMGGMGGMDGMM + 2 Oxidation (M)
8671	969.2575	4841.2511	4840.2284	1.0227	0	6	68	1	MSAVVLGMGSIPNGLALQNTGPFDLCNGDTVCLMPPIFPNVCCR + 3 Oxidation (M)
2503	777.5229	776.5156	775.4300	1.0856	1	6	4.5e+02	1	RSASSLR
3687	696.6296	1391.2446	1391.7157	-0.4711	1	6	4.2e+02	1	QAATGQEVKGTFR
6985	1001.4667	3001.3783	3001.3914	-0.0132	1	6	2.4e+02	1	TEAAGGAPAGSPGAPFQARSHTPDEGPSPER
8442	904.2249	4516.0881	4517.2326	-1.1444	1	6	86	1	LQRALTQFMLDSHTDAGYEEVYVPFLVNQDSLHGTGQLPK
4731	841.2392	1680.4638	1680.9410	-0.4771	1	6	3.6e+02	1	RSGEVEVLPIAEAIAK
5668	1118.5004	2234.9862	2235.0147	-0.0285	1	6	3.8e+02	1	EKVPDNASDQEMLEVVMNR + 2 Oxidation (M)
6343	882.4210	2644.2412	2644.4776	-0.2365	0	6	3.2e+02	1	GLVLLMMIFFGILEGIFFLASAVK + Oxidation (M)
6665	951.7932	2852.3578	2853.4192	-1.0614	1	6	2.8e+02	1	TTALTNACADHSDQRVVYLEHVVVR
8508	758.3358	4543.9711	4543.2298	0.7414	0	6	75	1	ENLIMRPDQMYGNVVVTSIGQLVLYNARPEDAGLYTCTAR + Oxidation (M)
8689	1274.1134	5092.4245	5093.4593	-1.0348	1	6	60	1	TTDVTGVITLPEGTEMVMPGDTTEMSVELIQPIAMEEGLGFAIREGGR + Oxidation (M)
8735	1136.3887	5676.9071	5677.9921	-1.0849	1	6	41	1	SVLQGILLSSEGERLNSMNLMLYMSPIAVIALLPVTIFMEPDVMSVTLTLGR + Oxidation (M)
2631	887.4165	886.4092	885.5144	0.8948	1	6	9.5e+02	1	SRQIQVR
5965	1247.6355	2493.2564	2493.3955	-0.1390	1	6	3.5e+02	1	GIVIENNAQIILVDTPGIFSPKR
7182	824.6148	3294.4301	3293.4481	0.9820	1	6	1.8e+02	1	MEAHGLEYEQPPHMNAHRLMFDYYNGK + Oxidation (M)
7530	912.6420	3646.5389	3647.7348	-1.1960	1	6	1.3e+02	1	FSGCYHGHSDLMLIAAGSGPATLGSPDSAGVTKATAK + Oxidation (M)
7674	955.4749	3817.8705	3816.7514	1.1191	1	6	1.5e+02	1	SGWQLGTPMADPMCGSGTLLIEAAMMASDRAPGLHR + 2 Oxidation (M)
7521	910.7231	3638.8633	3639.6579	-0.7946	1	6	1.7e+02	1	VIGLVENMSGFVCPHCSECTNVFSKGGGEELAR
8093	1062.7546	4246.9893	4246.2648	0.7245	1	6	1.1e+02	1	TTCFLMFLSAYFLSMWISNTSATLILLPIALGLLHKK + 2 Oxidation (M)
6049	841.7215	2522.1427	2523.1581	-1.0154	0	6	3.2e+02	1	SNMPAANDMTATAGGTDTASGKPTIK + Oxidation (M)
7016	756.5840	3022.3069	3022.3933	-0.0864	0	6	2.2e+02	1	AIEAGDAETGVTIMQMDAGLDTGDMLLER
7850	808.5628	4037.7776	4038.8496	-1.0720	1	6	1.1e+02	1	ADEALAAGAEAVGMDDLAEKMQAGDLNYDVVIATPDAMR + 2 Oxidation (M)
8324	746.1643	4470.9421	4470.2430	0.6991	1	6	80	1	LADSVILYLNAQVANGAQSLMIFDSWGGALSHSAYREFSLR
8581	661.7157	4624.9590	4625.7526	-0.7936	0	6	64	1	VEEEPEEEPEDTTEDTEQDEEEEMDAGTDEQEQEQEPEK + Oxidation (M)
5749	1156.7485	2311.4824	2311.1195	0.3630	0	6	2.7e+02	1	NELITSSMDFSIIHWDFLK + Oxidation (M)
5992	625.5335	2498.1049	2499.2953	-1.1904	1	6	3.3e+02	1	NIFPGMQGGPLMHIIAAKAVCFK
7857	808.9612	4039.7696	4039.8734	-0.1037	1	6	1.1e+02	1	MTHSTDIATLARWMAADFSNQAQAFENPPFYAHIR + 2 Oxidation (M)
2421	723.8781	722.8708	722.3460	0.5248	0	6	48	1	AHAGDPR
7859	1011.7097	4042.8097	4042.0725	0.7372	1	6	1.1e+02	1	IIMNSMPELMMISLASLVNIKSWSSISDIIFCTVAK
6936	749.8840	2995.5069	2996.5542	-1.0473	1	6	2.6e+02	1	ILMVLNGLASSAYNISHDTVHYGKHLK + Oxidation (M)
2520	786.8828	785.8755	785.4395	0.4360	0	6	4.6e+02	1	ASVGPLSR
5680	750.6502	2248.9288	2248.1322	0.7966	1	6	3.4e+02	1	VTEEEKSSVETAISDLELAAK
7001	753.8954	3011.5525	3011.4580	0.0945	1	6	2.6e+02	1	SEPNDPNAMTLATVDPDGLPDARMVLLK + 2 Oxidation (M)
7318	1154.4924	3460.4554	3459.7344	0.7210	1	6	1.5e+02	1	GDILNSMLPIERNGEILGYIWANELTEDIR + Oxidation (M)
8345	639.8582	4471.9565	4472.0685	-0.1120	1	6	80	1	TFMIAAISGDGVDDLRSTLAEMVPPGPFLYPEDQMSDAPMR + 2 Oxidation (M)
2381	699.9092	698.9019	699.3300	-0.4281	0	6	74	1	THSGGNK
4832	860.0485	1718.0824	1717.9111	0.1713	1	6	4.5e+02	1	AATIDQALNGKFGSAVR
7537	916.9185	3663.6449	3662.8457	0.7992	0	6	1.6e+02	1	VTRPGGLAVLSYTVWLGPFGGHEMGLSHYLGGYR + Oxidation (M)
7000	753.8952	3011.5517	3010.3978	1.1539	1	6	2.6e+02	1	MADTPAFAPVEPPTTHNRDEENESVLK + Oxidation (M)
7407	708.3326	3536.6266	3536.6360	-0.0094	1	6	1.8e+02	1	DNSFIASMLSLNCYSMNDSLSLKETDPEVIK + Oxidation (M)
8029	1051.9675	4203.8409	4204.9720	-1.1311	0	6	1e+02	1	SGVVGYNSGSTNLGDQAGNYTNSFGGTSSACPGAAGVAALILSR
8172	622.9949	4353.9134	4353.9318	-0.0184	1	6	87	1	GWGEDEPDEESHTPLPPPMKIFDNDPTQDEMSSSLLAAR + Oxidation (M)
8353	1121.0116	4480.0173	4479.9845	0.0328	1	6	87	1	VRRPIPGSDQTTTMSGGEGEPGEGEGDGEDVSSDSAPDSAPGPAPK
8410	904.0251	4515.0891	4515.2058	-0.1167	1	6	90	1	LNQIGAGDQGLMFGYATNETEELMPLPIMLSHHLMQRIAK + 3 Oxidation (M)
8430	753.6873	4516.0801	4517.1552	-1.0751	1	6	88	1	TLVEGANQYIGAGNMYNGDVEDLNKLHLYIMSQMEKPTTK + 2 Oxidation (M)
8558	767.8279	4600.9237	4602.0957	-1.1720	1	6	70	1	VATHCTEADVSAQHIETAREIGMDVSGFLMMSHMAPAEELAK + 2 Oxidation (M)
8758	957.6226	5739.6919	5739.1039	0.5880	1	6	41	1	MRIALFLLTNLAVMVVFGLVLSLTGIQSSSMTGLLIMALLFGFGGSIVSLMMSK + 3 Oxidation (M)
5719	1142.7349	2283.4552	2283.1430	0.3122	0	6	2.8e+02	1	HSVQVEVGGRPMVFEAGEIAR + Oxidation (M)
7005	754.8495	3015.3689	3014.3222	1.0467	1	6	2.4e+02	1	DLVDHNCINMRMQSGGGLYAWDFQR + 2 Oxidation (M)
7159	1086.1384	3255.3934	3254.4397	0.9537	1	6	1.9e+02	1	DPRSYDGTEVSYGCRPVPPSWMDDEALR
7620	1254.3026	3759.8860	3759.9864	-0.1004	1	6	1.6e+02	1	MLGIPISNILMVGNYASDLELDPMKDILILSALR + 2 Oxidation (M)
8186	872.8048	4358.9876	4359.1418	-0.1542	1	6	98	1	SDVWSFGILLYEIITYGKMPYSGMTGAQVIQMLSQNYR
4030	730.7156	1459.4166	1459.7823	-0.3657	1	6	4.5e+02	1	KHWFVLTDSSLK
8013	699.3018	4189.7671	4189.0426	0.7245	1	6	95	1	TVIVDNNTWNNTHIATVGTAMSSPEKAAWEIFNSLDVK + Oxidation (M)
8892	992.2928	5947.7131	5947.8873	-0.1741	0	6	30	1	SHVDLDALGSMQWTPLHLAAFQGEEGVMLALLQCGANPNAAEQSGWTPLHLAVHK + 2 Oxidation (M)
4049	736.6601	1471.3056	1471.7527	-0.4470	0	6	4.3e+02	1	MVSGLAANAGLPMPK + Oxidation (M)
7087	1046.4603	3136.3591	3136.4883	-0.1293	1	6	2.1e+02	1	GSSLNKPLSDRVEDINNQSSMIYDPWR + Oxidation (M)

☒	7473	903.1671	3608.6393	3608.6979	-0.0586	1	6	1.7e+02	1	TDVGNHSSKSSTVVQGSSEATPQSYLASSPNPFSR
☒	7218	1111.7799	3332.3179	3332.5694	-0.2515	1	6	1.6e+02	1	YAGGTNDKGIGMGMTVPISFAVFPNEDGSLQK + 2 Oxidation (M)
☒	8269	1109.4386	4433.7253	4434.3515	-0.6263	1	6	72	1	ILPPGLYKWTSPVGASADFTIIGTSTDTWIFQIAGTLGLAAGK
☒	2633	888.9058	887.8985	887.5076	0.3909	1	6	6.6e+02	1	ADGSIGKLK
☒	7851	1347.2600	4038.7582	4038.7733	-0.0152	0	6	1.1e+02	1	SSDSRPPQPDTSTQSNSESESPAISSPTPQDHAPMTNR + Oxidation (M)
☒	6264	871.3885	2611.1437	2610.2596	0.8841	1	6	3e+02	1	TDDDAFVRVDEVLLSLSMTNNTR
☒	8920	1021.3253	6121.9081	6122.8066	-0.8985	1	6	33	1	YSPQMGWEGANVIFDDWVHPLMMGLEEHLIGMFKHDFEFVDGHQSHLGHLGGK + 3 Oxidation (M)
☒	2531	795.7352	794.7279	795.3619	-0.6340	0	6	2.4e+02	1	MASMNVK + Oxidation (M)
☒	7002	753.8962	3011.5557	3010.4421	1.1136	1	6	2.6e+02	1	DQKGIYPSSANFSTDLHSLGQYIQEGR
☒	8030	1051.9680	4203.8429	4203.1773	0.6656	1	6	1e+02	1	GSQSAINQMIDYISPEGSIALLGVSEFPVEVNTRLVLEK
☒	5048	918.1594	1834.3042	1833.8090	0.4953	0	6	3.5e+02	1	VLSLGDDGGDGGSGSGGGGGTR
☒	8605	934.4243	4667.0851	4667.2995	-0.2144	1	6	74	1	LTLPGVAIGLAWTPMGGEIMFVEASRMEGEGQLTLTGQLGDVMK + 4 Oxidation (M)
☒	4319	771.4728	1540.9310	1541.8599	-0.9289	1	6	5.7e+02	1	MRLIPLTSAEQVGK
☒	5830	1207.5293	2413.0440	2413.2675	-0.2235	0	6	3.5e+02	1	MVSANQTASVTEFILLGLSAHPK
☒	6559	922.7210	2765.1412	2765.4018	-0.2607	1	6	2.5e+02	1	MPVSQKLTLVAPPGGPNGDSSGGTQGVAK + Oxidation (M)
☒	7083	1041.8105	3122.4097	3123.4529	-1.0432	1	6	2.4e+02	1	SGVEMERMASVYLEEGNLENAFVLYNK + 2 Oxidation (M)
☒	7848	808.5609	4037.7681	4036.8760	0.8921	1	6	1.1e+02	1	DGLLQNVVADVNNGEEFLSESESEASLEIDSDIKDEK
☒	8696	1019.6932	5093.4296	5093.5478	-0.1182	1	6	61	1	GSSDFIGVIHYLTALVTNIDINPSLSGIPDFNSDMGESINILSMRVR + Oxidation (M)
☒	5924	827.7313	2480.1721	2480.3420	-0.1700	1	6	3.6e+02	1	VLTEQALNEGKPANIVEKMIAGR
☒	6876	987.1170	2958.3292	2958.5154	-0.1863	1	6	2.6e+02	1	VLDSERTTIQGADGMLMVPQLAIAGVNK + 2 Oxidation (M)
☒	7617	940.9767	3759.8777	3761.0376	-1.1599	1	6	1.7e+02	1	AQRPLPRPLAADDAALLLEEAAAVPEAPWIGLRDR
☒	8180	1090.5056	4357.9933	4358.1212	-0.1279	1	6	99	1	ENPSTLAHLAMNDFAWTTNGCSVPTSAHTLANQLILRYK + Oxidation (M)
☒	2467	377.6082	753.2018	752.3640	0.8379	0	6	1.2e+02	1	MFGGSVR
☒	4164	747.6768	1493.3390	1492.6762	0.6628	1	6	4.2e+02	1	GVMINASDPRDMR + 2 Oxidation (M)
☒	5262	968.4163	1934.8180	1933.9601	0.8579	1	6	4.6e+02	1	RSLGSLSVAEPAMIAECK + Oxidation (M)
☒	7007	1006.7822	3017.3248	3016.5514	0.7734	1	6	2.3e+02	1	ATQMFDHVILAIAASPGKKPMFTLEER + Oxidation (M)
☒	7048	1019.4393	3055.2961	3055.5019	-0.2059	0	6	2.2e+02	1	EMVQGLIAGGLEAVTNAVEGAEDNEELAVK
☒	4378	782.2585	1562.5024	1561.8134	0.6891	1	6	4.3e+02	1	NVVVDELSGSLMKR + Oxidation (M)
☒	4701	833.7326	1665.4506	1664.8773	0.5733	1	6	3.8e+02	1	ELYAILENNKFSPK
☒	6292	654.0447	2612.1497	2611.2808	0.8689	1	6	3.1e+02	1	ESIATRLNFEMACTPAMTQLALK + Oxidation (M)
☒	7277	852.3832	3405.5037	3405.6811	-0.1774	0	6	1.8e+02	1	HAETIDIVIMTLGYLSMHLSFVSLFFSMR + 3 Oxidation (M)
☒	7415	1189.1444	3564.4114	3564.6896	-0.2782	1	6	1.4e+02	1	TLYETEVFSTDFSNVSAAQHEINSYVEKQTK
☒	8795	1160.1504	5795.7156	5794.5599	1.1557	1	6	41	1	AELTSDKDMYLDNNSIEEASGVYPIDDDDYASASGSGAGEDGESPELTTSRPIPK + Oxidation (M)
☒	4718	838.1076	1674.2006	1674.8002	-0.5995	1	6	4e+02	1	DGKSHLAVEENWYK
☒	7792	799.9724	3994.8256	3994.0948	0.7308	1	6	1.3e+02	1	SCQKPGTIMIFKNQLFVSTQDFDIELLLIQLPNK + Oxidation (M)
☒	2346	674.4718	673.4645	672.3079	1.1567	0	6	1.1e+02	1	GGPGEEK
☒	5790	590.0353	2356.1121	2356.1255	-0.0134	1	6	4.1e+02	1	GDGSQGEDVTQNVRTIADIPER
☒	6346	882.7568	2645.2486	2644.3516	0.8969	1	6	3.3e+02	1	AGMGAEAIKELLAEIDLDELSAELK + Oxidation (M)
☒	7086	1046.4574	3136.3504	3135.5686	0.7818	0	6	2.1e+02	1	LADTSVETGSPIVLEATYSGTPPISVSWMK
☒	8588	773.5269	4635.1177	4634.3996	0.7182	1	6	78	1	WPQPWALGQVVTKTMVNTVVANASFTYLTALTDSNIVAIWGR + Oxidation (M)
☒	6251	653.3306	2609.2933	2610.2901	-0.9968	1	6	3.4e+02	1	TVGWFTTMSPLVSAPWDSLSRTR + Oxidation (M)
☒	6505	678.0377	2708.1217	2708.3003	-0.1786	1	6	2.7e+02	1	LYYARENGLPEPDIPALSDDMVSK + Oxidation (M)
☒	6636	705.8366	2819.3173	2820.3860	-1.0687	1	6	3e+02	1	LKAEIDLPLNFEMAATDEMVAIACR
☒	7632	1272.9719	3815.8939	3816.8386	-0.9447	1	6	1.6e+02	1	QQMLALEGRLPDSLVACIGGGSNAMGLFHPFLDDR + 2 Oxidation (M)
☒	8209	730.8306	4378.9399	4379.2359	-0.2960	1	6	92	1	VSVFVEVEGAGDFLPPYAGNLDIMTAAATKVGEVIAGQITSAR + Oxidation (M)
☒	8351	896.8077	4479.0021	4478.3968	0.6053	1	6	92	1	VEPRPMITRPLPPPLPEPVVRPRVTPNDSAPAANGSQGLAER
☒	6986	1001.4676	3001.3810	3001.6045	-0.2236	1	6	2.6e+02	1	ASLTNVAELGAELGLLPEEMLLFGSTKAK
☒	7442	1198.2184	3591.6334	3590.8403	0.7931	1	6	1.8e+02	1	TAQSVFDLMLSLNERLATSFIIVTHDEQLAGR + Oxidation (M)
☒	4007	726.7030	1451.3914	1451.7303	-0.3389	1	6	4.4e+02	1	SGVPDPNMNPRLR
☒	6448	1334.1646	2666.3146	2665.2150	1.0996	0	6	3.4e+02	1	NHGNNEADAALQGLAQQGVDMEDLR
☒	6617	939.4317	2815.2733	2816.4201	-1.1468	1	6	2.9e+02	1	LELVSGNPGLHMSFTGSPGTGKTTVAMK
☒	7612	1253.9663	3758.8771	3759.8423	-0.9652	1	6	1.7e+02	1	IPQILDMVSELEFNLDTMPRINCAAAGNPFPVR + 2 Oxidation (M)
☒	8805	1160.1514	5795.7206	5794.5599	1.1607	1	6	42	1	AELTSDKDMYLDNNSIEEASGVYPIDDDDYASASGSGAGEDGESPELTTSRPIPK + Oxidation (M)
☒	7381	884.6575	3534.6009	3533.7534	0.8475	0	6	1.9e+02	1	AAVAQAGELPMLHVTDFISDMAAAYSAADLIISR + Oxidation (M)
☒	8694	849.9111	5093.4229	5092.5170	0.9059	1	6	63	1	TSIFPSDIEILENQQADVLICHEAPKPHPMGFQVINDLAMKMGVK + 2 Oxidation (M)
☒	8036	1052.4644	4205.8285	4206.1267	-0.2982	0	6	1e+02	1	SLAGGTLMVTPLIGADGEVYAVAQGQAQIGGYAVQGQSASVQK + Oxidation (M)
☒	8541	1143.5094	4570.0085	4570.3682	-0.3597	1	6	81	1	VLRPLEAVDLGLETIYEKFHPAVQSFSNILGHYMTGERPK + Oxidation (M)
☒	8723	927.8149	5560.8457	5559.9090	0.9367	1	6	45	1	MNPLIFIMLILTIILGTSIILTSTHWFMIWLGFEMNMAMIPVLMKK + 4 Oxidation (M)
☒	2451	738.1082	737.1009	736.3789	0.7220	1	6	84	1	KTAATVM + Oxidation (M)
☒	8358	641.8555	4485.9376	4486.1072	-0.1696	1	6	81	1	IILVDVDEEMAATVLCAGYHRGMYGDNYVWILPGYHSDK + Oxidation (M)
☒	8582	1157.4927	4625.9417	4625.3536	0.5881	0	6	69	1	ADGLLVSDLLQAIIPLFELDSYAPPLVMMAAVEGDTLDPEVER
☒	3634	687.5948	1373.1750	1372.6405	0.5345	0	6	4.9e+02	1	VTGPGGSPCLGSER
☒	4359	777.8389	1553.6632	1554.7711	-1.1079	1	6	5.9e+02	1	ESMALQLEATYRK + Oxidation (M)
☒	7296	859.3593	3433.4081	3434.5093	-1.1012	0	6	1.5e+02	1	MQNAAAAAGMPGQFMSPMFYGQQPGFFPPNGR + 2 Oxidation (M)
☒	7526	729.9154	3644.5406	3644.9418	-0.4011	1	6	1.5e+02	1	IDVWEMKMTALAILVTPVLVLLGAALAMMTDAGR + 2 Oxidation (M)
☒	7401	1179.5427	3535.6063	3535.5456	0.0607	0	6	1.9e+02	1	ATMAHANTSHPLYVAGMAGNTNHMSPWGEDAHK + 2 Oxidation (M)
☒	7566	929.1693	3712.6481	3712.8786	-0.2305	1	6	1.5e+02	1	ANGFKLETGPPMGVAPPPPPPPDYMLPPGLHAPEPK
☒	7616	940.9763	3759.8761	3759.9864	-0.1103	1	6	1.7e+02	1	MLGIPISNILMVGNYASDLELDPMKDILILSALR + 2 Oxidation (M)
☒	8893	992.2940	5947.7203	5947.8873	-0.1669	0	6	32	1	SHVDLDALGSMQWTPLHLAAFQGEEGVMLALLQCGANPNAAEQSGWTPLHLAVHK + 2 Oxidation (M)
☒	8239	883.3920	4411.9236	4412.9871	-1.0634	1	6	90	1	LRPGDCYMCPNIESNNWRNFIFNGQHITLCNACGLR + Oxidation (M)
☒	6441	889.7783	2666.3131	2665.3343	0.9788	0	6	3.4e+02	1	AIIQVQETELLAEVCNTVFDVMK + Oxidation (M)
☒	7062	1034.7964	3101.3674	3102.5213	-1.1539	0	6	2.3e+02	1	IIGIDLGTTNSCVAVMEGNEPVVIANSEGK + Oxidation (M)
☒	7928	822.7800	4108.8636	4109.7399	-0.8763	0	6	1.2e+02	1	YDQFGHAGVGSSAASGGGQYGAGASDFSDIFSAFNDMFGGGK + Oxidation (M)
☒	4120	741.0067	1479.9988	1480.7231	-0.7243	1	6	4.8e+02	1	VIAEEDNYIKCK
☒	6499	677.7884	2707.1245	2707.2382	-0.1137	0	6	2.7e+02	1	DNQDESILETEIMPSDDSLTIAQK + Oxidation (M)
☒	7744	987.4141	3945.6273	3944.8310	0.7963	1	6	1.1e+02	1	TDGSPTYNFCVVVDDIDMAITHIIRGDDHVNNTPK + Oxidation (M)
☒	8194	873.0053	4359.9901	4360.2287	-0.2386	1	6	1e+02	1	LNEEYEVPQEVLDTIYLMPKEADAIGLLEVGTAALASIDK
☒	8743	957.2893	5737.6921	5736.9640	0.7281	1	6	44	1	KLLVAYYMPEYPVAGATLPVLEALQENGADIIELGIPFSDPVGDGPVIQEAAQR
☒	7779	797.1566	3980.7466	3980.9268	-0.1802	1	6	1.3e+02	1	TPGRTQHINFFELQNGNFMVDLPGYGYAQVPEAVR + Oxidation (M)
☒	8114	853.4041	4261.9841	4261.4170	0.5671	0	6	1.2e+02	1	EALVLGAVLALLAFGLVLTTNAATIAWSFAALAVTLAYPFAK
☒	8295	892.6204	4458.0656	4459.0932	-1.0276	1	6	1e+02	1	ALFYMVMQCLGAICGAGVVKGFQEGLYMGAGGGANAVNPGYTK + 2 Oxidation (M)
☒	8372	748.9991	4487.9509	4489.0698	-1.1189	0	6	85	1	SDGQVTEEEVATIGAGDQGMMFGFACNETPELMPLPIALAHR
☒	8374	749.1641	4488.9409	4489.9493	-1.0083	1	6	85	1	MYSMMMETDLHSPGGAQAPTNLSGPAGAGGGGGGGGGGGGGGGTKANQDR
☒	8432	753.6874	4516.0807	4517.2326	-1.1518	1	6	94	1	LQRALTQFMLDSHTDAGYEEVYVPFLVNQDSLHGTGQLPK
☒	5844	810.6981	2429.0725	2429.2909	-0.2185	1	6	3.7e+02	1	ALVILAKGAEEMETVIPVDIMR + 2 Oxidation (M)
☒	6019	834.0662	2499.1768	2500.0931	-0.9163	1	6	3.9e+02	1	YSEGYPGARYYGGNEFIDASER
☒	7878	1014.2181	4052.8433	4051.7437	1.0996	1	6	1.3e+02	1	GTSTNQFTFQTYNDNSCSQATSDQALSFTCSDNVKK
☒	8476	567.0041	4527.9746	4528.2288	-0.2542	1	6	87	1	DPALSLDSLSICMSISALSFMVSVGFNAAVSVRTSNELGAGNPK + Oxidation (M)
☒	8687	849.4037	5090.3785	5089.5066	0.8720	1	6	69	1	VAVMIMQALGLQVVMPELQPDAFPFWDSCMMVLSIVAMILMTRK + 5 Oxidation (M)
☒	2443	368.1872	734.3598	734.3381	0.0217	0	6	1.8e+02	1	MEGGLGR + Oxidation (M)
☒	2526	789.5712	788.5639	789.4232	-0.8593	0	6	7.8e+02	1	VIETNSK
☒	6983	1001.4639	3001.3699	3000.4838	0.8861	1	6	2.7e+02	1	MFEKPVVKSFQYGNHTVTLETGVMAR + 2 Oxidation (M)
☒	7410	708.9288	3539.6076	3540.6640	-1.0564	1	6	1.8e+02	1	LLKLLNALDDMDDVQEVFGNYEIDDEVMER
☒	7936	822.7838	4108.8826	4108.9398	-0.0572	1	6	1.2e+02	1	DIITTFTESFPQDFFCVCSVEEANNLVSTVQDLKR
☒	8120	855.3917	4271.9221	4272.1552	-0.2331	0	6	1.1e+02	1	QQEMAAAAAATGGSVLNVAALLASGTQVTPQIAMAAQMAALQAK + 3 Oxidation (M)
☒	8347	746.4982	4472.9455	4473.0749	-0.1294	1	6	86	1	HIAEGEWQGKQFLLMDTGGYNADGDTISMAMLEQTLMAIR + 2 Oxidation (M)
☒	7371	1178.1476	3531.4210	3531.8283	-0.4074	1	6	1.5e+02	1	TYPQGGLPMVALEEVSLEIPTGSVFGIVGRSGAGK + Oxidation (M)
☒	7455	1198.5504	3592.6294	3592.7098	-0.0804	1	6	1.9e+02	1	KDPTSTMSALQLAAVTQPGTGGSVTGGGGAAGGGGSAAGMK + 2 Oxidation (M)
☒	7996	699.0012	4187.9635	4186.9608	1.0028	1	6	1.2e+02	1	KASASASGAGAAGAAGGATGGAASGAPSAQEGGGATTPCPETLNMVLR + Oxidation (M)
☒	3636	687.8007	1373.5868	1373.7052	-0.1183	1	6	7.3e+02	1	DPGRVVFDSGVAR
☒	3818	711.8633	1421.7120	1422.6371	-0.9250	0	6	6.9e+02	1	GDIEMDAIIMDGK + Oxidation (M)

☒	4008	726.7197	1451.4248	1450.6246	0.8003	0	6	4.7e+02	1	AADSPSMASNIDEK + Oxidation (M)
☒	6953	999.8401	2996.4985	2995.4498	1.0487	0	6	2.9e+02	1	MDIAHDLQVIAAQEHTLVFPQFDADR + Oxidation (M)
☒	7518	1213.6320	3637.8742	3637.1004	0.7737	1	6	1.8e+02	1	NLLIGIGAGAAVLMVALLVLASVLSRIFGDVSGGLNK + Oxidation (M)
☒	2442	368.1871	734.3596	734.3381	0.0215	0	6	1.8e+02	1	MEGGLGR + Oxidation (M)
☒	8783	828.9648	5795.7027	5796.8290	-1.1264	0	6	43	1	MNYIPCMITIGNFICGLLAIHSLLYHNIHSAVLFIFTGMFLDFFDGMAAR + 2 Oxidation (M)
☒	3371	638.3101	1274.6056	1275.6492	-1.0436	1	6	8e+02	1	AAVKAMDNLSEK
☒	5147	937.6340	1873.2534	1872.9152	0.3382	0	6	3.8e+02	1	GPSAMPDPAQGPPSPALQR
☒	6820	730.5425	2918.1409	2917.2641	0.8768	1	6	2.2e+02	1	MSASPDDLSTGGRLQNMTVDECFQSR + Oxidation (M)
☒	8827	967.2948	5797.7251	5798.5560	-0.8308	0	6	43	1	FPHPSDCSQVQQNTNAASGLYTIYLNGDASRPMQVYCDMDTDGGGWIVFQR + 2 Oxidation (M)
☒	5682	751.6750	2252.0032	2252.1068	-0.1037	0	6	4.2e+02	1	QMMQTLEAETGDLLLFVADK
☒	6988	1001.4681	3001.3825	3001.4824	-0.0999	1	6	2.7e+02	1	VSHQTSPVLITLEPMWMDSSRMATLR + Oxidation (M)
☒	8314	559.7500	4469.9418	4469.0041	0.9376	1	6	89	1	DQPIDFSGGSEDNELSVEDFEASEDLNDIDESIPVVKNSSK
☒	7266	851.1154	3400.4325	3401.5863	-1.1538	1	6	1.7e+02	1	MDEGLDTGPIAMTEKIAIGPDMTAGELHDVMK + Oxidation (M)
☒	8063	850.0032	4244.9796	4245.1739	-0.1943	1	6	1.2e+02	1	LSSGEIEEVILATNATVEGQATAHYLLEATKHLPVHMTR + Oxidation (M)
☒	4605	818.2332	1634.4518	1633.7981	0.6537	1	6	4.3e+02	1	VPSSIGDTAEVEMRK + Oxidation (M)
☒	6968	1000.1308	2997.3706	2998.4553	-1.0848	1	6	2.7e+02	1	IVEGQQSSEVISPPEEVNRMNDLDLK + Oxidation (M)
☒	7122	1066.1416	3195.4030	3196.4124	-1.0095	1	6	2.2e+02	1	FLSNACRFAEDVNEHTTHLVMMNSEGR + 2 Oxidation (M)
☒	7564	743.3374	3711.6506	3712.8283	-1.1777	1	6	1.6e+02	1	AMLQDIAILTGGQVISEDLGMKLESVTMDMLGSAK + 3 Oxidation (M)
☒	7235	838.8686	3351.4453	3350.6243	0.8210	1	6	1.9e+02	1	GIASLCKSLCHPDCILEHLVLANCSLNEK
☒	8571	771.4979	4622.9437	4622.2368	0.7070	1	6	72	1	ATDISPIALLSENMIGGMQQDGNGTVAVEALQELFTSSDPQSKK + 2 Oxidation (M)
☒	8637	947.0718	4730.3226	4731.2435	-0.9208	1	6	88	1	AGDVFVESGADYFVVDPVMVCKGEDEVLNPGNTEAMIQYLLPK + Oxidation (M)
☒	7271	852.1140	3404.4269	3404.6170	-0.1901	1	6	1.8e+02	1	IETMFTAMQNVVPSHLADFNTFDFKSINR + 2 Oxidation (M)
☒	5283	974.4132	1946.8118	1946.9659	-0.1540	1	6	4.9e+02	1	LEPKAADLSMWSDLVEK + Oxidation (M)
☒	7969	827.3964	4131.9456	4131.8742	0.0714	1	6	1.3e+02	1	FYAGDGTAGMFMSGFFPIMMFGLPGAALAMYFAAPKER + 4 Oxidation (M)
☒	8115	853.6044	4262.9856	4263.8804	-0.8948	0	6	1.2e+02	1	GMVAAVSVGIVNGEAICDLEYVEDSAAETDMNVVMTEDGR + 3 Oxidation (M)
☒	2491	384.8701	767.7256	767.4038	0.3218	0	6	1.4e+02	1	SHAVAGAR
☒	5474	1026.6215	2051.2284	2051.9366	-0.7082	0	6	4.1e+02	1	FGVCSDVMVGSSLISMYGK + Oxidation (M)
☒	7643	764.3858	3816.8926	3816.7434	0.1492	1	6	1.7e+02	1	LLCGGEALKGDGFDNGAWVAPTVFTDCSDEMTIVR + Oxidation (M)
☒	5665	1116.0210	2230.0274	2230.2110	-0.1835	0	6	4.6e+02	1	QPVLVAANFENLYLVGALGSR
☒	8519	910.3999	4546.9631	4546.3332	0.6299	1	6	85	1	DFHRIPCLNASQAWIAALGEIVAQNLQGWPVQALPVPHTTGA
☒	8688	1274.1123	5092.4201	5092.5202	-0.1001	1	6	68	1	FKAWEVVPSMFALVPNQPDMPAGQVASAESDAVSALISLGYKPQEASK + 2 Oxidation (M)
☒	5765	782.8469	2345.5189	2345.1936	0.3252	1	6	3e+02	1	TLAAEMTKFVHSEEALEQALK
☒	7411	709.1274	3540.6006	3540.7565	-0.1559	1	6	1.9e+02	1	AQQAAEAARPYAELMQRMLAALADNVAGSPSAPR + Oxidation (M)
☒	7619	940.9780	3759.8829	3759.9864	-0.1035	1	6	1.8e+02	1	MLGIPISNILMVGNYASDLELDPMKDILILSALR + 2 Oxidation (M)
☒	8176	1089.9811	4355.8953	4355.1955	0.6998	1	6	96	1	TNKTVVVGSHQDSINMYLPTILAAPGADDDGSGTVTILEALR + Oxidation (M)
☒	8193	1091.0034	4359.9845	4360.9126	-0.9281	1	6	1.1e+02	1	QMVGEPLAKMTAAAASATGAAPPQQMQEEGNENPMQMHSNK + 5 Oxidation (M)
☒	2915	1012.9723	1011.9650	1012.4648	-0.4998	0	6	5.3e+02	1	SGMDPQGPPK
☒	6561	692.2931	2765.1433	2765.4348	-0.2915	1	6	2.7e+02	1	AVAPLKQAADAYLLDNSHLDIESGVR
☒	6907	995.7954	2984.3644	2983.4882	0.8762	1	6	2.8e+02	1	GTETTLALPMASDLLLYDVTENSMRVK + Oxidation (M)
☒	7957	826.1802	4125.8646	4125.8293	0.0353	1	6	1.2e+02	1	AKMVYEMALQLIESESTESPESFESPESSESSEAEVK + 2 Oxidation (M)
☒	8563	771.1624	4620.9307	4622.0718	-1.1410	1	6	72	1	GIMAMNSYNHMGQGIHHATSSLLTSMMAETFEELAVDYMKK + 3 Oxidation (M)
☒	8742	957.2890	5737.6903	5737.8933	-0.2030	1	6	46	1	ALLMSSTATMVKAFDEAINDGVDVLSISLASAAPFRPIDSITGDLELGSFHAVMK + Oxidation (M)
☒	4457	789.3514	1576.6882	1575.8256	0.8626	1	6	6.6e+02	1	ANKEGITVFELAER
☒	8866	735.2119	5873.6370	5873.8969	-0.2599	1	6	36	1	MKTLLLDLALWSLLFQPGWLSFSSQVSQNCHNGSYEISVLMMGNSAFAEPLK + Oxidation (M)
☒	8911	1018.9898	6107.8951	6108.9370	-1.0419	1	6	37	1	VCGAVHGSNPLRPESGSFTDYMTSESEFTLKIPAGLSFEQAVGMGVTGIGTLGMALFR + 2 Oxidation (M)
☒	4426	784.8101	1567.6056	1566.6806	0.9250	1	6	5.7e+02	1	NEYKVVHDECMK + Oxidation (M)
☒	7758	991.4598	3961.8101	3962.7446	-0.9345	1	6	1.4e+02	1	GWVPGNMMDGFLCPGCSKSYMPWELEAQSSGVIPK + 3 Oxidation (M)
☒	7985	1039.6698	4154.6501	4155.0632	-0.4131	1	6	96	1	EGLGMGLAICLTIAEVHNGKIWAESPPAGGACFFVSIPVS
☒	8157	1076.5007	4301.9737	4300.9529	1.0208	1	6	1.2e+02	1	KEVFEEDLEQMMNDFGGDAVAAATGLPDNGVALNGGTPAYR + 2 Oxidation (M)
☒	8525	761.6595	4563.9133	4564.1636	-0.2503	1	6	77	1	MAMEAVMALLEATHDTPACVVTLSGNQSVRPLPLMECVQMTK + 2 Oxidation (M)
☒	7519	1213.6328	3637.8766	3636.9298	0.9468	1	6	1.9e+02	1	TTTSVTFMSQAGIANNIPEQLGLQKTVLPVGHIR + Oxidation (M)
☒	8276	892.2193	4456.0601	4457.0654	-1.0053	1	6	1.1e+02	1	FFQERGFVYTNTPIITGSDCEGAGEMFQLTTMDLNNIPK + Oxidation (M)
☒	6938	999.5097	2995.5073	2994.4467	1.0606	0	5	3e+02	1	TSTEGMISHFESVLPMGPTIIYNVPSR + 2 Oxidation (M)
☒	8149	1076.2313	4300.8961	4300.1016	0.7945	1	5	1.1e+02	1	ISVDADALLTLQETVNNMAERTTAILYGSTPDIGSSSSSTR + Oxidation (M)
☒	3425	648.3185	1294.6224	1293.6308	0.9916	0	5	7.4e+02	1	QMLTAEMIPTK + 2 Oxidation (M)
☒	3635	687.7151	1373.4156	1373.7952	-0.3795	1	5	5.4e+02	1	MILLANKTDLVK + Oxidation (M)
☒	6570	924.4232	2770.2478	2769.4443	0.8035	1	5	3.3e+02	1	VRLGLLLSEIGQQNGVEVSAQEMQR + Oxidation (M)
☒	7092	787.8820	3147.4989	3146.4748	1.0241	0	5	2.7e+02	1	AAGIVAPTMLVCGDDLVVISESQGAEEDER + Oxidation (M)
☒	2310	647.2991	646.2918	646.3399	-0.0480	0	5	33	1	TGGGTVR
☒	4077	738.4252	1474.8358	1475.7871	-0.9513	1	5	7.2e+02	1	SIIDIVPEYKDGK
☒	7828	1007.1642	4024.6277	4024.5916	0.0361	1	5	1.1e+02	1	NLEEINESDPLNSSDNSMTSSSEESCSEESDKESDK + Oxidation (M)
☒	7860	1013.7173	4050.8401	4049.8795	0.9606	1	5	1.4e+02	1	FIAVNGATAHPHYDPDGTAYNMGNSFGPYGFSYKVIR + Oxidation (M)
☒	7610	1253.9629	3758.8669	3758.8264	0.0405	1	5	1.9e+02	1	TSFRPRGPEDFGDGGAFPEIHVAQFPLGLGLGDMR + Oxidation (M)
☒	7124	799.8605	3195.4129	3196.4554	-1.0425	1	5	2.4e+02	1	DHRTNTEMGNVQAVMDGDIDTFIDAYLR
☒	8676	695.2085	4859.4086	4858.2872	1.1214	1	5	83	1	VIISSSGGSVSGGSGGASGGSGGASGSGGSVGVSGGGGASVGGSILGSSASMDTKGNLR + Oxidation (M)
☒	7199	828.6221	3310.4593	3309.3607	1.0986	1	5	2.1e+02	1	NGGTGTNFFNEKEDSESDSAHFDYTPNSK
☒	6899	995.4598	2983.3576	2984.5107	-1.1531	1	5	2.9e+02	1	TFRVVYPTCSYVFTTDPVNVEHILK
☒	7784	996.1957	3980.7537	3981.7503	-0.9966	0	5	1.4e+02	1	MGTNGVHVSFGAGHEMGGVMGNGSGQPPATEYTLQGVMR + 4 Oxidation (M)
☒	8067	1062.2567	4244.9977	4245.1371	-0.1394	1	5	1.3e+02	1	AIVIVALDNLTKGSSGQALQNANLMLGEDETAGLMMAPLFP + 2 Oxidation (M)
☒	8642	947.4729	4732.3281	4731.2435	1.0847	1	5	92	1	AGDVFVESGADYFVVDPVMVCKGEDEVLNPGNTEAMIQYLLPK + Oxidation (M)
☒	5231	960.1650	1918.3154	1917.9254	0.3900	1	5	3.8e+02	1	STADLMYERYGITVQR + Oxidation (M)
☒	6345	882.7504	2645.2294	2646.3918	-1.1624	1	5	3.7e+02	1	RILEVYLNVVEWGNGIFGAEAAAR
☒	7756	991.2128	3960.8221	3959.7895	1.0326	0	5	1.5e+02	1	MTSTQAVSFDTFQLAAATADLSDESAASDYADIVEFR + Oxidation (M)
☒	5802	1190.5466	2379.0786	2378.3508	0.7279	1	5	4.1e+02	1	LLKFVCLLASVAAAKPTPGASHK
☒	8478	755.6700	4527.9763	4527.0835	0.8928	1	5	94	1	LGSASARMLDVPGHTSGHVAFWFEDDAALFCGDTLFALGCGR + Oxidation (M)
☒	3843	717.8633	1433.7120	1433.7514	-0.0394	1	5	7.6e+02	1	SFVEAAVEQSRLV
☒	6900	1492.6869	2983.3592	2982.5572	0.8021	1	5	2.9e+02	1	KQMQSIHAFLGSLCLPSHVLDFSIVR
☒	6904	995.7943	2984.3611	2983.4709	0.8902	1	5	2.9e+02	1	LEDMVQRGHNFAIVDEVDSILIDEAR
☒	8649	950.6547	4748.2371	4748.2361	0.0011	1	5	87	1	KENMGLVTHLQYPVPLEEEDAIDEAEEDTVESVMSPPELPPR + Oxidation (M)
☒	8906	1219.5853	6092.8901	6092.0382	0.8519	1	5	39	1	ILGLGDQGIGGMGIPIGKLALYTACGGISPAYCLPILLDVGTNNQQLLDDPMYMGWR + Oxidation (M)
☒	2635	445.1177	888.2208	887.5076	0.7132	1	5	8e+02	1	VSASKAPTK
☒	6655	948.7650	2843.2732	2842.1805	1.0926	0	5	3.2e+02	1	DWECMTELLVEEPMQGEEVMSER + Oxidation (M)
☒	7080	1041.1443	3120.4111	3121.4734	-1.0624	1	5	2.6e+02	1	EVAVSHKPDAFLRESDMGSNVTSNSSSVR + Oxidation (M)
☒	7194	828.3707	3309.4537	3310.4791	-1.0254	1	5	2.2e+02	1	AQIAMYESELERAHGQMLEEMQSLEEDK + Oxidation (M)
☒	7737	658.4448	3944.6251	3945.0346	-0.4095	1	5	1.2e+02	1	SEGIVLGSDILKVNSFLNHQIDPFLMSQIGQAFAEK
☒	7855	1010.7023	4038.7801	4039.8793	-1.0992	0	5	1.3e+02	1	NITSGVAHVSASFNNTMVTITDAQGNAISWSSAGHMGFK + 2 Oxidation (M)
☒	7931	1370.6293	4108.8661	4107.9084	0.9577	1	5	1.3e+02	1	MGLYGQACPSVTSLRMTSELESSLTSMDWLPQLTMR + 2 Oxidation (M)
☒	6138	852.7151	2555.1235	2556.1485	-1.0251	1	5	3.6e+02	1	STNLDSWSPEQLRTMMFGGNNR + Oxidation (M)
☒	7811	1001.9224	4003.6605	4003.0275	0.6330	1	5	1.2e+02	1	SYLHHLHRAGEILGSMLNTVHNLHYYQTLTAELR + Oxidation (M)
☒	7868	1013.9679	4051.8425	4051.0720	0.7705	1	5	1.4e+02	1	DYTPSTNSLVSMASKFSLGQTELILLLMCFILALSR + 2 Oxidation (M)
☒	8486	648.0032	4528.9715	4530.1188	-1.1473	1	5	93	1	DQLLQIYNAAIEEMYRFYSYGDAMWIAPEAVLFDARPE + 2 Oxidation (M)
☒	8144	1433.9775	4298.9107	4298.9332	-0.0225	1	5	1.1e+02	1	NMSEFLGDTSIPGQESPAPSSCSLPVGSSVGSSGSSESWKNR
☒	8564	925.1940	4620.9336	4621.3064	-0.3728	0	5	75	1	AVWKPAPDLATSAAAWLTAGAAHHTVMSTQVGVEVFEDFAEIAR
☒	2307	647.2855	646.2782	646.3398	-0.0616	0	5	34	1	VAGGTSR
☒	8018	1048.5006	4189.9733	4189.0775	0.8958	0	5	1.4e+02	1	VKPQINEGDSVLLQIEQEVSSVAEGNGSSNSSLGVTFNTR
☒	8241	883.5782	4412.8546	4413.9714	-1.1167	1	5	92	1	MYADMQAAQGAEAAAAGAAGAGGAGASAGGASQQQDDVVDAEFKEVK + Oxidation (M)
☒	8260	1104.7412	4414.9357	4414.1302	0.8055	1	5	1e+02	1	LFNLLLPLDPEDPKDGACSHGHSHGGHSHGVSLQLAPSDFR
☒	8852	976.3048	5851.7851	5851.8612	-0.0761	1	5	47	1	GGEPVVCLTAYTTPMARLVDAHCDIALVGDSLGMVLHGLPSTIGVSMEMMILHGK + 2 Oxidation (M)
☒	2446	368.1878	734.3610	734.3381	0.0230	0	5	2e+02	1	EAAAAMR + Oxidation (M)
☒	4131	742.1810	1482.3474	1482.7678	-0.4204	1	5	4.6e+02	1	DPDDARNVILEVK
	7520	1213.9591	3638.8555	3639.6821	-0.8266	0	5	2e+02	1	LIAANAATEPDAGSDIYNMQATAQPCEGGYILNGK + Oxidation (M)

7987	832.5634	4157.7806	4157.1535	0.6272	1	5	1.1e+02	1	VTALSVGMDPVLSVAANSSGGVTGKMISPQSIAVACAAVGLAGK + Oxidation (M)
8736	947.9523	5681.6701	5681.9243	-0.2542	0	5	50	1	FDSTDTGWVIMSIGMAIGAGIVFLPVQVGLMGLWVFLLSSVIGYPAMYLFQR + Oxidation (M)
4425	784.5722	1567.1298	1567.7525	-0.6227	1	5	4.8e+02	1	RLTIMDQDGHNPR + Oxidation (M)
7176	1094.8092	3281.4058	3280.6142	0.7916	1	5	2.2e+02	1	MDEIVPNASGNVVAAPMVDVVDHSAVEMKIK + Oxidation (M)
7264	850.6135	3398.4249	3398.5799	-0.1550	0	5	1.9e+02	1	AMIISWVMPLNEDGSNVVTYWIASSDGSDNK
7549	1229.8752	3686.6038	3685.7460	0.8578	1	5	1.6e+02	1	TGVEMEALTGASVAALTVYDMCKALSHSMSIGPTR + 2 Oxidation (M)
8282	892.4193	4457.0601	4457.0654	-0.0053	1	5	1.1e+02	1	FFQERGFVYTNTPIITGSDCEGAGEMFQLTTMDLNNIPK + Oxidation (M)
8640	789.5595	4731.3133	4732.2203	-0.9069	1	5	96	1	IASLSTFLVGAGAIGCEMLKNWAMMGVATGESGHISVTDMDSIEK + 3 Oxidation (M)
8665	967.4017	4831.9721	4832.6450	-0.6729	1	5	66	1	TLMLAVEKGAIALIIAGFASSLHEGLMAAGLNMLIGIIIWYVAFSK + Oxidation (M)
8896	1190.7462	5948.6946	5949.8865	-1.1919	1	5	36	1	VPVVVDAGIGTPSEAAQAMEMGADAVLINSAIAMAANPVAMAQAMGMATQAGRLAYLSGR + 4 Oxidation (M)
6266	871.3888	2611.1446	2612.1880	-1.0434	1	5	3.5e+02	1	MMMASKDVVPTAASSENANNNSSIK + Oxidation (M)
6615	939.4309	2815.2709	2815.3673	-0.0965	0	5	3.3e+02	1	QTGWIEILGAGMVHPNVLEMAGFDSK + Oxidation (M)
8568	771.4960	4622.9323	4622.4896	0.4427	1	5	76	1	LFVDSAFQRDYPVIMAMTVISAVLVVVGNLIADILYAIVDPR + Oxidation (M)
8636	1181.7334	4722.9045	4723.2031	-0.2986	1	5	70	1	QEHGNYTQTSIPGIFAAGDVIDHVYRQAITSSASGCMAALDSER
8734	947.1561	5676.8929	5677.8693	-0.9763	1	5	50	1	FSDLKNLGVLDVSHNQLTGNLNVLTDLQNLVSLNISYNDFSGDLPNTPFFR
6897	995.4593	2983.3561	2983.5358	-0.1798	1	5	3e+02	1	MATSLSTAGIETTVIADAAIFAVMSRVNK + Oxidation (M)
5018	907.0055	1811.9964	1812.9078	-0.9114	1	5	6e+02	1	RSIATEHLTQTEANSR
5288	975.6132	1949.2118	1948.8697	0.3421	1	5	4.5e+02	1	VGPEDAGQGAVYRCGEER
8184	872.8044	4358.9856	4358.0795	0.9062	1	5	1.2e+02	1	ASASSGPSSSVGFSSFDPAVPSCTLSSAASGIKRPMASEVLEAR + Oxidation (M)
7494	908.3998	3629.5701	3628.7611	0.8090	0	5	1.8e+02	1	MSDLFIGELITLSIMAFALGMDAFSVGLGMGMVK + 4 Oxidation (M)
8189	872.8067	4358.9971	4358.0795	0.9177	1	5	1.2e+02	1	ASASSGPSSSVGFSSFDPAVPSCTLSSAASGIKRPMASEVLEAR + Oxidation (M)
8633	1181.2714	4721.0565	4721.2676	-0.2111	1	5	79	1	MTVAEAARNLSCVGAEPIAVTDNLNFGSPEKPIGYWQLAEACR + Oxidation (M)
2276	631.6090	630.6017	630.3701	0.2317	0	5	19	1	IGSVAGK
2650	897.9116	896.9043	897.5032	-0.5989	1	5	5e+02	1	LGKAPGEAR
7550	922.9065	3687.5969	3686.7378	0.8591	1	5	1.7e+02	1	KPEETLRMLEDNVMQPGYYQLLDSALACSQR + 2 Oxidation (M)
5695	1138.7433	2275.4720	2275.2035	0.2686	0	5	3.3e+02	1	TSFVTLFAAGMLVSALAQAHPK + Oxidation (M)
7852	1010.6998	4038.7701	4038.8722	-0.1021	1	5	1.3e+02	1	EGVVGSIQDMGAAGITSSAFEMSASGGTGMDLHLDRVPTR + 2 Oxidation (M)
7932	1028.2240	4108.8669	4110.0580	-1.1911	1	5	1.4e+02	1	QVDVSRGDMIVAIDSQPQSGLSFDAQIVALQPEGIEPGK + Oxidation (M)
8251	631.5676	4413.9223	4413.0242	0.8981	1	5	1e+02	1	DLGCAVSHDGLLDLACAVEMVHAASLILDDMPCMDDAKLR + Oxidation (M)
7559	738.7296	3688.6116	3689.7088	-1.0972	0	5	1.7e+02	1	GQQQVGQWDQTQVQAQAQAQPQAQTSSSSSSMLR + Oxidation (M)
8518	910.3997	4546.9621	4547.2789	-0.3167	1	5	92	1	GMVVENTNAPISVPVGHGTLGRIMNVLGEPIDEAGPIQYTETR + Oxidation (M)
2594	851.1081	850.1008	849.5548	0.5460	0	5	6.4e+02	1	VRPLPLR
2729	936.7991	935.7918	936.4916	-0.6998	1	5	6.3e+02	1	TYKSVPDK
8612	1170.7445	4678.9489	4678.8737	0.0752	1	5	74	1	KDQNNNNNNNNSNSNCQNGGGSICDDSSNSSTPSLSSYSNGNNK
7925	822.7798	4108.8626	4109.7399	-0.8773	0	5	1.4e+02	1	YDQFGHAGVGSSAASGGGQYGAGASDFSDIFSAFNDMFGGGK + Oxidation (M)
8895	992.4560	5948.6923	5947.8873	0.8051	0	5	36	1	SHVDLDALGSMQWTPLHLAAFQGEEGVMLALLQCGANPNAAEQSGWTPLHLAVHK + 2 Oxidation (M)
6274	871.7231	2612.1475	2613.2282	-1.0807	1	5	3.6e+02	1	AQEFTTFKDGQTAMAFHVVQGER + Oxidation (M)
8332	746.1660	4470.9523	4471.1026	-0.1503	1	5	1e+02	1	NLFNPENIFISKEGGGAGNNWASGYTQGEAVQETLLDMIDR + Oxidation (M)
8575	1156.9923	4623.9401	4623.1025	0.8376	1	5	77	1	SANYCYASEACRSGVLLLCEVALGEMNELLNADYDANNLPK + Oxidation (M)
8648	949.4540	4742.2336	4742.5052	-0.2716	1	5	94	1	NLIIEDEKMVVVALQPPADLEVGSAEVIVESLTQGGLASQLPSQR
8712	1097.3193	5481.5601	5481.5277	0.0324	1	5	56	1	VLFSVANMMHPGTLEHGDPAEFSTKGISFFMTLPCYGEAEQNFNLMLR + 3 Oxidation (M)
7427	894.6647	3574.6297	3574.7411	-0.1114	1	5	2.1e+02	1	LERALIDFMIDVHVTEHGYVEMMPPFIANR + 2 Oxidation (M)
8886	989.4514	5930.6647	5931.5884	-0.9237	0	5	37	1	IAHQLAMGELEDHYVTIEVEEQQPSMFDMLQGSGMEQMGMNMQDALGNLMPK + 5 Oxidation (M)
5901	1236.5427	2471.0708	2470.2057	0.8652	1	5	3.9e+02	1	VAVMGCVVNGPGEAREADLGIAGGR + Oxidation (M)
6565	922.7231	2765.1475	2764.3598	0.7877	0	5	3e+02	1	APMGTVSIQIWSGACATVVALVGDAMK + 2 Oxidation (M)
7034	1016.1202	3045.3388	3044.4543	0.8845	1	5	2.7e+02	1	SMTNSDETVASGSLGVRTGLNLAVPMYSR + 2 Oxidation (M)
7130	645.2823	3221.3751	3222.5615	-1.1864	1	5	2.3e+02	1	ENMDSGAFQAVQEAAIVALRDGDAFLAEIR + Oxidation (M)
4966	897.9151	1793.8156	1794.9120	-1.0964	1	5	6.4e+02	1	MAGRSPSFLEMLGLVR + 2 Oxidation (M)
7081	1041.1451	3120.4135	3121.4734	-1.0600	1	5	2.8e+02	1	EVAVSHKPDAFLRESDMGSNVTSNSSSVR + Oxidation (M)
7255	1128.5430	3382.6072	3381.6367	0.9705	1	5	2.4e+02	1	GMSVSDLIAMGNSARTGEPGEGMLYNTLQAIAK
7191	828.3693	3309.4481	3309.2773	0.1708	1	5	2.3e+02	1	AGCGFEWCWVCQTEWTRDCMGAHWFG + Oxidation (M)
7447	719.5319	3592.6231	3591.6307	0.9924	1	5	2.2e+02	1	DLQEDKEGMFDTLETVHTSLDIFAGMIETMK + 3 Oxidation (M)
7949	825.3462	4121.6946	4120.9432	0.7515	1	5	1.1e+02	1	QVEPPGYNELFMVEDVCNVDLEQGLDLCKPEKVNK + Oxidation (M)
7798	801.1362	4000.6446	3999.8727	0.7720	1	5	1.2e+02	1	YLEDKVSLFGVTSLVSCGSSCLLSCVMQGNVYDANK
8274	891.3969	4451.9481	4452.3338	-0.3856	1	5	1.1e+02	1	AKPEWMILSLIPVIPPDLRPMVQLDGGRFATSDLNDLYR + Oxidation (M)
3823	713.0324	1424.0502	1424.7082	-0.6580	1	5	5.4e+02	1	LRAGMTFVTNDGK + Oxidation (M)
7337	696.5245	3477.5861	3478.5949	-1.0088	1	5	2.3e+02	1	EEMDTTHCMKQNEIISQAVCALLNSGGGVVR + 2 Oxidation (M)
7876	1351.6257	4051.8553	4051.7437	0.1116	1	5	1.5e+02	1	GTSTNQFTFQTYNDNSCSQATSDQALSFTCSDNVKK
3227	592.7441	1183.4736	1182.6145	0.8591	0	5	8.2e+02	1	HIEAGAFVSPR
8865	840.0974	5873.6309	5872.8032	0.8277	1	5	40	1	MDFEAVIGLEVHAELSTNTKIYCGCTTEFGGQPNTHVCPICLGLPGSLPQLNK
3271	611.2789	1220.5432	1220.6223	-0.0791	1	5	8.7e+02	1	VMNLDRFDLV
8764	1152.7567	5758.7471	5757.9899	0.7572	1	5	51	1	ILFISLCILCLLQFIVYSYFHREGFSVILMGLAAWPWLLHADYAFSHK
6957	999.8426	2996.5060	2997.5416	-1.0357	1	5	3.3e+02	1	AGDMPFDVVIAAPDAMRVVGTLGQILGPR + 2 Oxidation (M)
6210	1297.5840	2593.1534	2593.4045	-0.2511	1	5	3.9e+02	1	DGKNYLMLALVMVGSAMILALGLGK + Oxidation (M)
4423	784.4857	1566.9568	1567.7916	-0.8348	0	5	6.7e+02	1	TTTGDGMIAALQVFK + Oxidation (M)
7611	940.7259	3758.8745	3759.9864	-1.1119	1	5	2.1e+02	1	MLGIPISNILMVGNYASDLELDPMKDILILSALR + 2 Oxidation (M)
8043	1058.4786	4229.8853	4229.0854	0.7999	1	5	1.3e+02	1	DRTLDQMCTVTRPGLSMMASSIAVELLISTIHHPYGGR + Oxidation (M)
2539	799.8267	798.8194	799.4664	-0.6470	1	5	5.2e+02	1	DGLLARR
5864	611.0261	2440.0753	2441.2373	-1.1620	1	5	4.2e+02	1	STVLEPVWMVGVDPASDGLARAR + Oxidation (M)
7291	858.3771	3429.4793	3430.4010	-0.9217	1	5	2.1e+02	1	SEAGETTASEMMFDDGIDEDINMAEVEKYR + 3 Oxidation (M)
7431	896.2195	3580.8489	3581.9387	-1.0898	1	5	2.3e+02	1	RLMLSLPVLVLVLAMVLEGPAPAQGTMDLDFTR + Oxidation (M)
7613	940.7274	3758.8805	3759.9864	-1.1059	1	5	2.1e+02	1	MLGIPISNILMVGNYASDLELDPMKDILILSALR + 2 Oxidation (M)
8415	904.0255	4515.0911	4515.2058	-0.1147	1	5	1.2e+02	1	LNQIGAGDQGLMFGYATNETEELMPLPIMLSHHLMQRIAK + 3 Oxidation (M)
1961	561.2857	560.2784	560.2667	0.0117	0	5	1.4	1	GGAGGSR
7657	955.2320	3816.8989	3815.7238	1.1751	1	5	1.9e+02	1	KPFWETKSLNQMTVPEWESLCDGCGLCCLVR + Oxidation (M)
7791	799.9718	3994.8226	3995.8968	-1.0742	0	5	1.6e+02	1	AIQLHPLVCAAFNADFDGDQMAVHVPLSIEAQMEAR + 2 Oxidation (M)
8060	849.8017	4243.9721	4245.1371	-1.1650	1	5	1.4e+02	1	AIVIVALDNLTKGSSGQALQNANLMLGEDETAGLMMAPLFP + 2 Oxidation (M)
7509	910.4734	3637.8645	3636.9915	0.8730	0	5	2.2e+02	1	LTDLPIGLLSLINLLMLLMTAFIATDTFISWR + 2 Oxidation (M)
8848	836.8337	5850.7850	5851.8612	-1.0763	1	5	51	1	GGEPVVCLTAYTTPMARLVDAHCDIALVGDSLGMVLHGLPSTIGVSMEMMILHGK + 2 Oxidation (M)
2603	430.1923	858.3700	858.4559	-0.0859	0	5	1.3e+03	1	APSSASALR
2622	440.3577	878.7008	879.3909	-0.6900	0	5	7.4e+02	1	WLSDMGR + Oxidation (M)
6243	870.7709	2609.2909	2610.3021	-1.0113	0	5	4.2e+02	1	QFDLLIDWGWFYFITKPMFK + Oxidation (M)
7539	917.1682	3664.6437	3664.9772	-0.3335	0	5	2e+02	1	ILIHIGHHLNVVNLLGACTKPGGPLMVIVEFCK + Oxidation (M)
7640	1273.3046	3816.8920	3815.8581	1.0338	0	5	1.9e+02	1	NIMASGVIPQISVIMGPCAGGAVYSPAMTDFIFMVR + Oxidation (M)

8651	1189.2732	4753.0637	4753.3576	-0.2939	1	5	85	1	PLGAPALLALALGLGLWLGALAGDPGRGCGPCPLPCFCGPAPDAACR
7461	899.1665	3592.6369	3592.7009	-0.0640	1	5	2.3e+02	1	AWEAHYLMSMTVFSVMGSSIMTLGIRMVQER + 2 Oxidation (M)
7618	940.9776	3759.8813	3761.0376	-1.1563	1	5	2.1e+02	1	AQRPLPRPLAADDAALLLEEAAAVPEAPWIGLRDR
8046	1059.7574	4235.0005	4234.7734	0.2271	0	5	1.4e+02	1	LQSSPMGYQMPQFQGNMRPNMQQSQMFDPSAYGMSR + 5 Oxidation (M)
8625	1171.5006	4681.9733	4681.1617	0.8116	1	5	78	1	ASELDDKEVEIELAPSGSQGMPMFEIPGMPGASMGAINISDMLGK + 2 Oxidation (M)
4917	879.4038	1756.7930	1756.8917	-0.0986	0	5	6.8e+02	1	AGAIEGEDMLPEVALVK + Oxidation (M)
7293	687.4888	3432.4076	3431.6829	0.7248	1	5	1.9e+02	1	YEFGQALFTGWAAASLCLLGGVLLSCSCPRK
8473	755.6694	4527.9727	4527.1760	0.7967	1	5	1e+02	1	LMTGLLEVLASATEDFYTDGRNLSDVNAALCLLNGYYCLR + Oxidation (M)
6300	871.7247	2612.1523	2613.2823	-1.1300	1	5	3.9e+02	1	AIEALPPGNFKVSFPNGAEGSGPDSR
7379	884.6569	3534.5985	3534.7700	-0.1715	0	5	2.3e+02	1	LVGDVDFEDALPMVSAITPVPGGVGPMTIAYLMK + 2 Oxidation (M)
5621	1100.2537	2198.4928	2198.0967	0.3961	1	5	3.7e+02	1	VLQQIDEALDTRDEAAFHK
7738	1315.8831	3944.6275	3943.9753	0.6521	1	5	1.4e+02	1	IHFAPGEDGVAFATQEKETLTMWIPPLQMVFRPR + 2 Oxidation (M)
8654	962.6494	4808.2106	4807.2754	0.9352	1	5	90	1	VMGICGGFQMLGEWVHDPLGIEGEAGSSEGLGLFAMQTELTAEKR
8741	957.2889	5737.6897	5738.7664	-1.0766	1	5	53	1	MTVPPTATSSAPVAVPSTAPVTYPMPQTQMGCSQPPKMEAPAIRPPSHATAAPHK + 4 Oxidation (M)
7195	1104.1603	3309.4591	3310.4493	-0.9902	0	5	2.4e+02	1	ATDGSLQSEDWTLNMEICDIINETEEGPK + Oxidation (M)

☒	7945	1028.7223	4110.8601	4111.1645	-0.3044	0	5	1.5e+02	1	SLSTVLSLSTQFAWLIPIYGFAGMVVSLPWATGWIQR + Oxidation (M)
☒	8842	1170.9320	5849.6236	5848.8620	0.7617	1	5	46	1	YSVIVIEYGVPDVGPLIQMPAALSFPMNMETYDWGFSSEPEPHIGGRSLVTPR
☒	8858	979.7773	5872.6201	5871.7442	0.8760	1	5	43	1	FENPGNSVTVTVDAPATGMYDLSIIYCANMGQKINSLTVNDQSVGDITFTENTK + 2 Oxidation (M)
☒	4921	880.4067	1758.7988	1759.8417	-1.0429	1	5	6.9e+02	1	IGFSVPAYKDYDETR
☒	8443	1130.0294	4516.0885	4516.5113	-0.4228	0	5	1.1e+02	1	LLHPLFVQMNVPGSLSHVISFIFAYAIITFLHVVVGELAPK
☒	7389	590.1078	3534.6031	3533.4450	1.1581	1	5	2.3e+02	1	FQMLRQHNSSSNSNSSSNNNNSNNNSSMMR + 2 Oxidation (M)
☒	8161	1076.9977	4303.9617	4304.1093	-0.1476	1	5	1.4e+02	1	HVAMHVAASSPEYLTPSDVPADVVAKEQQVQIEIAMNEGK + Oxidation (M)
☒	8859	735.0860	5872.6298	5872.7798	-0.1500	0	5	43	1	FLEDAVEIDVDALFDGTDMYLGGIMEHIEEAGIHSGDSACVLPPITLGNNVIER + Oxidation (M)
☒	7437	898.9128	3591.6221	3590.8167	0.8054	1	5	2.3e+02	1	YAHMVDVGQVGVNVPIPVPLPMFSFTGSRSSFR
☒	8684	1262.0455	5044.1529	5044.4149	-0.2620	1	5	65	1	NAAVATCAVAPDSQAAWDDLISVDGLGPTVALSLSDAFANPEERAAFDR
☒	7648	1273.3057	3816.8953	3816.8386	0.0567	1	5	2e+02	1	EQMLALEGRLPNSLVACIGGGSNAMGLFHPFLDDR + 2 Oxidation (M)
☒	7749	792.9335	3959.6311	3960.0601	-0.4290	1	5	1.4e+02	1	IQSVVVEFILNHVDVLFSGRISMAMQEGAASLSRPK + 2 Oxidation (M)
☒	8050	1060.2589	4237.0065	4238.1459	-1.1394	1	5	1.5e+02	1	LEAMTAVCSVGLDMIAVPGKTPAHTLSGIIADEAAIGMINGK + Oxidation (M)
☒	4918	879.4041	1756.7936	1756.8917	-0.0980	0	5	6.9e+02	1	AGAIEGEDMLPEVALVK + Oxidation (M)
☒	8883	1187.1378	5930.6526	5931.2008	-0.5481	0	5	39	1	EGGTAAGFVEGAGIQHFVAAFLVALFILLLLMNGEMLPSGIGLSAAIAGAVLMSLLTIK + 3 Oxidation (M)
☒	6291	871.7238	2612.1496	2612.3122	-0.1627	1	5	3.9e+02	1	FSLPSDNTQSWLLTTYLDKDLR
☒	8026	841.7752	4203.8396	4204.1589	-0.3193	0	5	1.3e+02	1	MGNVLAASSPAPPPAGSPPVPGLVSVPPGFTMPPVAGLTPTPDK + 2 Oxidation (M)
☒	8822	829.1101	5796.7198	5797.7186	-0.9988	1	5	51	1	GSPPVRSAEGTGGTYLMQDSSGLNYVSVFKPMDEEPMAVNNPQQLPVSSDGQGLK + 2 Oxidation (M)
☒	7454	719.5331	3592.6291	3591.6895	0.9396	1	5	2.3e+02	1	METQTVQQELESLPTTKMAQTNPMPGSVGPWK + 3 Oxidation (M)
☒	6306	872.0574	2613.1504	2613.2282	-0.0778	1	5	4.1e+02	1	AQEFTTFKDGQTAMAFHVVQGER + Oxidation (M)
☒	7786	996.4434	3981.7445	3980.8731	0.8714	1	5	1.5e+02	1	ALSEAATAGVEVTDEAMAMERQGQRPLLVEGSEDNFK + 2 Oxidation (M)
☒	2623	880.4064	879.3991	879.4371	-0.0380	0	5	9.8e+02	1	GLSMESIK + Oxidation (M)
☒	8109	1066.2222	4260.8597	4260.0844	0.7753	1	5	1.3e+02	1	FLVNSFLKGGGGGGGGGLGGGLGNVLGGLISGAGGGGGGGGGGGGGGGTAMR + Oxidation (M)
☒	8766	961.1349	5760.7657	5759.7764	0.9893	1	5	56	1	VAAQIKFGFSVNTWAVGDLVQVVNSISDGDVNALVDEYESCYTMTPATQIHGK + Oxidation (M)
☒	8191	873.0027	4359.9771	4361.0707	-1.0935	1	5	1.3e+02	1	GVSSGPGPMGLMGPRGPPGAVGAPGPQGFQGPAGEPGEPGQTGPAGPR + 2 Oxidation (M)
☒	6280	654.0443	2612.1481	2613.2745	-1.1264	1	5	4e+02	1	RNDAYIGVMIDDLVTLGTSEPYR + Oxidation (M)
☒	6911	998.4456	2992.3150	2992.5076	-0.1927	1	5	3.2e+02	1	QTSYADMLDLAVEHIKGLQHQVEVRP + Oxidation (M)
☒	7553	615.6071	3687.5989	3688.6952	-1.0962	1	5	1.9e+02	1	WDSNMSAVITDDDGDEVFYTVGILRSAAAAGDVGR + Oxidation (M)
☒	2482	383.9490	765.8834	766.3717	-0.4883	0	5	1.8e+02	1	SAVMMTK
☒	5859	812.8607	2435.5603	2435.1899	0.3704	1	5	3.3e+02	1	KDLGLMAMTYGNVYVASIAMGAK + 2 Oxidation (M)
☒	8513	758.5023	4544.9701	4544.2785	0.6916	0	5	1e+02	1	EIIPVSAGTGDGVEQLVQLVHGLLPEGPCYFPDDILTDVPER
☒	8909	1222.5850	6107.8886	6108.0331	-0.1445	1	5	44	1	ILGLGDQGIGGMGIPIGKLALYTACGGISPAYCLPILLDVGTNNQQLLDDPMYMGWR + 2 Oxidation (M)
☒	3718	703.5392	1405.0638	1404.7408	0.3230	0	5	6.2e+02	1	MSAAAPKPRPAHR + Oxidation (M)
☒	7666	955.2332	3816.9037	3817.0079	-0.1042	1	5	2e+02	1	DSMDCLATKGHLLEVVVLLSSYLLSMAQPIQILK + 2 Oxidation (M)
☒	4043	734.7165	1467.4184	1466.7089	0.7096	0	5	5.6e+02	1	QSFSRPFSMPQR
☒	2961	511.5962	1021.1778	1021.5226	-0.3448	0	5	7.2e+02	1	SQVVSSLMR + Oxidation (M)
☒	2742	952.4506	951.4433	951.5614	-0.1181	1	5	9.8e+02	1	QPLRVPSR
☒	8033	841.9753	4204.8401	4205.8879	-1.0478	0	5	1.4e+02	1	MNPLQPIQHSITNSQMSGGQQLEAEGSQAHNSYSHPDR + 2 Oxidation (M)
☒	8536	1143.0098	4568.0101	4567.2464	0.7637	0	5	1e+02	1	YWGAPIPMMTLEDGTVVPTPEDQLPVVLPEDVVMDGISSPLK + 2 Oxidation (M)
☒	7522	910.9696	3639.8493	3638.7609	1.0884	0	5	2.3e+02	1	GLVAPGRPGLVAGMASLLDGGGGGGGGGAGGAGGAGSAGGGADFR + Oxidation (M)
☒	7658	955.2324	3816.9005	3815.9967	0.9038	1	5	2e+02	1	IMLSPHPYALRSHLCVGEETASLGVYLHSKPVVR
☒	8554	1149.3068	4593.1981	4593.0958	0.1023	1	5	1.1e+02	1	QEEGGESMGVDVGWKITEAFASSFEPIFDATYLQPGWTEER
☒	8023	841.2061	4200.9941	4201.1048	-0.1107	1	5	1.5e+02	1	LPAWMKNNPALMASGGNSSTTTTTTSELGVPRPPTPPLHR + Oxidation (M)
☒	8506	909.8006	4543.9666	4545.0598	-1.0932	0	5	1e+02	1	GYIEAIADFIGPDIDILAPDVYTNEMMMGWMMDQYSIIR + 3 Oxidation (M)
☒	7304	862.4069	3445.5985	3446.6844	-1.0859	1	5	2.6e+02	1	MGKVIGIDLGTTNSCVAVMEGGEPVVIANAEGSR + Oxidation (M)
☒	7532	1218.9631	3653.8675	3654.8498	-0.9823	1	5	2.3e+02	1	LPADALVGAEDAAIAQLFAGLNPERIMGAASAVGMGR + 2 Oxidation (M)
☒	8808	1160.1519	5795.7231	5794.5599	1.1632	1	5	54	1	AELTSDKDMYLDNNSIEEASGVYPIDDDDYASASGSGAGEDGESPELTTSRPIPK + Oxidation (M)
☒	4171	748.5709	1495.1272	1494.7500	0.3772	1	5	5.5e+02	1	YRVEALIGEGGMGK + Oxidation (M)
☒	8779	1159.9504	5794.7156	5794.5599	0.1557	1	5	56	1	AELTSDKDMYLDNNSIEEASGVYPIDDDDYASASGSGAGEDGESPELTTSRPIPK + Oxidation (M)
☒	8493	907.0012	4529.9696	4529.2723	0.6973	0	5	1.1e+02	1	HLFDLTPDQIEVIVHPQSIIHSMVGYSDGSVLAQLGVPDMR + Oxidation (M)
☒	7862	1013.9647	4051.8297	4052.8594	-1.0297	1	5	1.7e+02	1	DANISMYLAIGDNMTHFTDSIMAAKLDYWSSQLGNK + 2 Oxidation (M)
☒	8746	1435.6761	5738.6753	5737.8933	0.7820	1	5	56	1	ALLMSSTATMVKAFDEAINDGVDVLSISLASAAPFRPIDSITGDLELGSFHAVMK + Oxidation (M)
☒	8935	1089.1604	6528.9187	6528.0910	0.8277	1	5	29	1	YMNHGQFIEMVWTTFPAVMLLIMAFPSFMLLYICDEVMAPAMTIKAMGLQWYWK + 4 Oxidation (M)
☒	8894	992.2971	5947.7389	5947.8931	-0.1542	1	5	42	1	EAGVELVLVSSGAVAAGMSQLGWTTRPSAMNELQAAASLGQMRLVQAWESSFGEHGK + 3 Oxidation (M)
☒	7003	1004.8621	3011.5645	3010.4210	1.1435	0	5	3.5e+02	1	AFYDDSGADSQPFGRPQKPYEGGIVGPR
☒	5146	937.4056	1872.7966	1872.9469	-0.1503	0	5	6.6e+02	1	IAPADISLDVNDFIQDK
☒	8731	1113.3759	5561.8431	5561.6164	0.2267	1	5	61	1	MTDNDALSLPPDVFAAEDLAAEIDEETSCVVVQTPDAFGNLRDLSPLAAACK
☒	8915	1222.7860	6108.8936	6108.0331	0.8605	1	5	46	1	ILGLGDQGIGGMGIPIGKLALYTACGGISPAYCLPILLDVGTNNQQLLDDPMYMGWR + 2 Oxidation (M)
☒	8870	847.9561	5928.6418	5928.7117	-0.0699	1	5	47	1	LISDGDVAGAVAAAQDSNSVDIMMGTGGTPEGIITACAMKCMGGEIQGILAPMNDFER + Oxidation (M)
☒	2395	355.0683	708.1220	707.3602	0.7618	0	5	54	1	ATGGGAFK
☒	6297	871.7241	2612.1505	2612.2850	-0.1345	1	5	4.2e+02	1	MVWMKFLGVLLMSMGCFVIFR + 3 Oxidation (M)
☒	6992	1001.8000	3002.3782	3001.5720	0.8062	1	5	3.5e+02	1	ELENDETLLVQSGKPIGILKTHEDAPR
☒	8118	1068.4969	4269.9585	4268.9857	0.9728	1	5	1.5e+02	1	SKQPLMSPAWSPDGQTLAYVSFQNGQAEIYMMNIYSGK + 2 Oxidation (M)
☒	8402	904.0242	4515.0846	4514.2349	0.8497	0	5	1.3e+02	1	AYGLENAMSGELVEFSNGVLGMAQNLDATDVGIIVLGDFLSIR + Oxidation (M)
☒	3528	665.8787	1329.7428	1330.7609	-1.0180	0	5	9.8e+02	1	AILGAEAVFAITR
☒	7021	1009.4381	3025.2925	3025.5141	-0.2216	0	5	3e+02	1	IVPIEDMPYMADGTPVDVVLNPLGVPSR + 2 Oxidation (M)
☒	7197	1104.4883	3310.4431	3311.5407	-1.0976	1	5	2.5e+02	1	GGFTTVCMMPNTNPAMDSKNNLAIAQDIIR + 2 Oxidation (M)
☒	8799	1160.1509	5795.7181	5794.5599	1.1582	1	5	56	1	AELTSDKDMYLDNNSIEEASGVYPIDDDDYASASGSGAGEDGESPELTTSRPIPK + Oxidation (M)
☒	8877	742.3376	5930.6426	5930.9916	-0.3490	1	5	42	1	MNPSIFIILLTTLILGTMMVITSSHWLLAWIGFEMNMMAFIPIMMKNPSPR + 5 Oxidation (M)
☒	2980	519.1374	1036.2602	1035.5349	0.7254	1	5	7.4e+02	1	KDTLDAAFR
☒	7716	972.9062	3887.5957	3888.1306	-0.5349	1	5	1.5e+02	1	SVFIMRTVQIAVMCVLALTVVFGIFFLGCNLLIK + Oxidation (M)
☒	7027	1013.4445	3037.3117	3037.4783	-0.1666	1	5	3.2e+02	1	GMDRAQGDYAGMLATVINALMLQNALER + Oxidation (M)
☒	6508	678.0382	2708.1237	2708.3261	-0.2024	1	5	3.7e+02	1	GGTLSMPKDEAANGLMASYLAQAGLAR + Oxidation (M)
☒	8166	868.9999	4339.9631	4339.1440	0.8192	0	5	1.3e+02	1	QVHFVNGMEELYTALQTHVAPQTLLLCMGAGDITHLATK + 2 Oxidation (M)
☒	5037	913.9874	1825.9602	1826.0335	-0.0733	0	5	7.3e+02	1	QMLPLLQTELLVSGLR + Oxidation (M)
☒	7065	1035.8014	3104.3824	3104.4823	-0.0999	1	5	3.2e+02	1	MVQIWQMEPYPCGDPRLPHHLFPPK + 2 Oxidation (M)
☒	1933	556.4404	555.4331	556.2718	-0.8387	0	4	0.35	1	GPGGGGR
☒	3055	1089.3258	1088.3185	1088.6230	-0.3045	0	4	8.4e+02	1	SGLFGILSPAK
☒	7675	1273.6349	3817.8829	3818.7042	-0.8213	1	4	2.2e+02	1	NGKGMDAATADYIGMLGTVMNAMALQDAMEAAGQPTR + 3 Oxidation (M)
☒	4141	745.9984	1489.9822	1489.7348	0.2475	0	4	7e+02	1	VQPGDLMVFNNTR
☒	5042	916.4033	1830.7920	1829.8474	0.9447	1	4	7.1e+02	1	QEKLMLYMGVGDSMGR + Oxidation (M)
☒	5622	1100.5002	2198.9858	2198.0967	0.8891	1	4	5.7e+02	1	VLQQIDEALDTRDEAAFHK
☒	8618	1171.0015	4679.9769	4680.3085	-0.3316	1	4	87	1	TLIASLPEALAETDFVCASVNVGSTRSGINMDAVAQMGEVVVAGSK + Oxidation (M)
☒	4494	797.3573	1592.7000	1592.7947	-0.0946	1	4	8.2e+02	1	KVYNHYQNLSAEK
☒	7680	955.4799	3817.8905	3819.0831	-1.1926	1	4	2.2e+02	1	VVEIVALFALSFWLMSKASPTSLVLGVVMNGIAQGR + Oxidation (M)
☒	2475	758.0223	757.0150	757.3606	-0.3456	0	4	4.3e+02	1	QAPGEEK
☒	7761	1322.6909	3965.0509	3964.9088	0.1421	1	4	1.9e+02	1	MSVFEHVDSPLYLGLMSGTSADGIDAALVRFADATHR + Oxidation (M)
☒	8881	848.2431	5930.6508	5929.6973	0.9534	1	4	43	1	ISESAAKASEQLVTGATQESATFSNDGLSGTMALSVQDTVGQEVLQMSDMMGSLSDR + 4 Oxidation (M)
☒	6784	964.4346	2890.2820	2891.4310	-1.1490	1	4	3.6e+02	1	MEEQVCVIWAGTNGYLDALPLNKVR + Oxidation (M)
☒	7555	738.5276	3687.6016	3688.6390	-1.0374	1	4	2e+02	1	GSDRGSGWSAAQNMSDWGNHFSFDVISDIVFGAR + Oxidation (M)
☒	7606	937.4112	3745.6157	3744.8848	0.7309	1	4	1.9e+02	1	LASEAGIESVAMPAMGAGVGGLSVAEVAREAAMAASILR + 2 Oxidation (M)
☒	3523	664.7320	1327.4494	1327.7347	-0.2852	1	4	7.5e+02	1	KLTAAAAESLEPK
☒	7778	797.1564	3980.7456	3981.2253	-0.4797	1	4	1.7e+02	1	RAAGWVLLGSVLGCVVLGGSPGLWMLLLSLGVFLWLR + Oxidation (M)
☒	7938	1028.4701	4109.8513	4110.8384	-0.9871	1	4	1.6e+02	1	EMTVREALNSALDEEMSADPSVFLMGEEVGEYQGAYK + Oxidation (M)
☒	4702	833.7330	1665.4514	1665.7668	-0.3154	0	4	5.4e+02	1	FSVHTMIDESASQAK + Oxidation (M)
☒	6480	671.8346	2683.3093	2682.2678	1.0415	0	4	4.6e+02	1	QFLNYTLTGVGGFMAASMLMPMVR + 3 Oxidation (M)
☒	6810	971.7932	2912.3578	2912.4154	-0.0576	1	4	3.9e+02	1	QDVPIAVVMSEYVDIAKAFFEGDEPK + Oxidation (M)
☒	7906	819.1464	4090.6956	4090.9616	-0.2660	1	4	1.4e+02	1	DLDKDVASLLEPFQGSFDPSAEQATACTLLVNHLSMR + Oxidation (M)
☒	8912	1222.5872	6107.8996	6107.8930	0.0067	1	4	48	1	AAAAAFGLPLYRYLGGVYAHVLPVPMMNIMNGGQHATNSTDFQEFMIMPVGADSFR + 4 Oxidation (M)
☒	8183	872.8018	4358.9726	4358.3217	0.6509	1	4	1.4e+02	1	ALYNNLSIDKFPVGWGSSILGFISLGMIAIPVFFYLNGPK
☒	8781	966.7949	5794.7257	5794.0433	0.6825	1	4	60	1	AGLVPLIMGLVTNPTGALMDEAMAILSILSSHPEGKAAIGAAEPVPVLVEMIGSGTPR + 2 Oxidation (M)

☒	5356	1003.5811	2005.1476	2005.0197	0.1280	1	4	6.3e+02	1	NLATAARNLGLGMMLGSQR + 2 Oxidation (M)
☒	5714	761.2433	2280.7081	2280.2147	0.4934	0	4	3.9e+02	1	MIAGATAPSSQHEILIASNLIK + Oxidation (M)
☒	7837	808.5591	4037.7591	4036.8760	0.8831	1	4	1.6e+02	1	DGLLQNVVADVNNGEEFLSESESEASLEIDSDIKDEK
☒	7797	800.1742	3995.8346	3995.9150	-0.0803	0	4	1.9e+02	1	DAMMGAAEMPQIPPEILMNTAALTGFPGLPMPMPGVQR + Oxidation (M)
☒	8391	902.7993	4508.9601	4509.2878	-0.3277	1	4	1.1e+02	1	MEGIEEPLARIAGNTYVMDAAATLITSGIMMGEKPAVLSAIVK + 3 Oxidation (M)
☒	8744	1148.5477	5737.7021	5737.8933	-0.1912	1	4	61	1	ALLMSSTATMVKAFDEAINDGVDVLSISLASAAPFRPIDSITGDLELGSFHAVMK + Oxidation (M)
☒	5186	944.9265	1887.8384	1887.9149	-0.0764	0	4	7.3e+02	1	SYPHIEEVAILSTCNR
☒	6362	884.3990	2650.1752	2649.1579	1.0173	0	4	4.3e+02	1	NHENSVDEDNGNIPESFYTNGGLK
☒	8596	1162.7557	4646.9937	4646.2924	0.7013	1	4	1e+02	1	AGDLAALLTNIEEGDILFIDEIHRMSPAIEEILYPAMEDFR + Oxidation (M)
☒	7651	1273.3062	3816.8968	3816.8386	0.0582	1	4	2.2e+02	1	QQMLALEGRLPDSLVACIGGGSNAMGLFHPFLDDR + 2 Oxidation (M)
☒	2277	631.7285	630.7212	631.2813	-0.5601	0	4	23	1	SSPAAEA
☒	2559	809.7503	808.7430	809.3966	-0.6536	0	4	4.2e+02	1	GIACPHR
☒	7933	822.7808	4108.8676	4110.0580	-1.1903	1	4	1.7e+02	1	QVDVSRGDMIVAIDSQPQSGLSFDAQIVALQPEGIEPGK + Oxidation (M)
☒	8703	1035.2747	5171.3371	5171.6178	-0.2807	0	4	77	1	MNPLVFTVIMSTVMLGTAIVATSSHWLMAWIGFEMNMLAVIPILMK + 4 Oxidation (M)
☒	2523	789.0532	788.0459	788.3916	-0.3457	0	4	7.4e+02	1	DPETLSK
☒	2532	795.7353	794.7280	795.4464	-0.7183	1	4	3.5e+02	1	AAVRHSR
☒	3204	1167.5334	1166.5261	1165.6819	0.8442	1	4	9.9e+02	1	IKVLDLHSNK
☒	7483	905.4059	3617.5945	3616.9287	0.6658	1	4	2.3e+02	1	SHVKQGLHVFDIPAVASSICVATTDVLAEIQALK
☒	8034	841.9764	4204.8456	4204.0094	0.8362	1	4	1.5e+02	1	NIEAIKLYLAVMSIYEPDLLDEYMIDDDFHPMISR + 2 Oxidation (M)
☒	3842	717.5696	1433.1246	1433.7514	-0.6268	1	4	6.6e+02	1	SFVEAAVEQSRLV
☒	5650	1107.9642	2213.9138	2215.0116	-1.0978	0	4	5.4e+02	1	EFGSVNGDNKPIWMHAEER
☒	8097	1416.6724	4246.9954	4245.9230	1.0724	1	4	1.7e+02	1	TLMGMNWVATYCPHIPYVMKTDSDMFVNTEYLINK + 4 Oxidation (M)
☒	5370	1010.1966	2018.3786	2017.9527	0.4259	0	4	4.8e+02	1	TGSLVMVHAENGDAADYLR
☒	8846	975.9460	5849.6323	5850.1238	-0.4915	1	4	53	1	TGLIFGAIETLTPLIGWSLGMLASQFILEWNHWIAFTLLVFLGGRMVIEGFR + Oxidation (M)
☒	2387	706.9433	705.9360	706.3320	-0.3959	0	4	85	1	AGGMVEK + Oxidation (M)
☒	8262	1473.5844	4417.7314	4418.3624	-0.6311	1	4	1.1e+02	1	RSYVLLAFAIVLIISIISAQVEADASSDAFADAVADAVADPIK
☒	2519	784.4888	783.4815	782.4286	1.0529	0	4	5.6e+02	1	VLTEHGK
☒	8624	937.4011	4681.9691	4682.2133	-0.2442	1	4	91	1	GPPFGSPMGHPGPMPPHGMRGPPPLMPPHGYTGPPRPPPYGYQR + 2 Oxidation (M)
☒	8913	1222.5883	6107.9051	6108.0331	-0.1280	1	4	50	1	ILGLGDQGIGGMGIPIGKLALYTACGGISPAYCLPILLDVGTNNQQLLDDPMYMGWR + 2 Oxidation (M)
☒	8925	1228.3965	6136.9461	6137.8447	-0.8985	0	4	48	1	EGPEGCNLLIYHLPQEFGDAELMQMFLPFGFVSFDNPASAQTAIQAMNGFQIGMK + 2 Oxidation (M)
☒	8946	951.1533	6651.0222	6651.1807	-0.1585	1	4	32	1	SSQGRPSNMYFQTHDQISMISAGPSHVAAMNIPIPFNLVMPPMPPPGYFGQANGPAAGRGTPK + 2 Oxidation (M)
☒	7196	828.6180	3310.4429	3309.5719	0.8710	1	4	2.7e+02	1	VMIHQPASSFYEGQAGECMIEAEEVLKLK + Oxidation (M)
☒	8106	853.1768	4260.8476	4261.0031	-0.1555	0	4	1.4e+02	1	SGIAHSMEEAYGVLEQVGFPCIIRPSFTMGGTGGGIAYNR + 2 Oxidation (M)
☒	8760	1148.9469	5739.6981	5740.0017	-0.3036	1	4	62	1	KGPIDAVVLDPPTIPSHHGPDVNTVLPHIDIVTMAMIVPTTQMHMHAITIELK
☒	7720	975.4120	3897.6189	3897.7720	-0.1532	1	4	1.7e+02	1	VPSDSSGPVGVCTYDDHRVAMSFSLLAGMVNSQNER + Oxidation (M)
☒	2479	761.6084	760.6011	760.3715	0.2296	0	4	6.2e+02	1	GVTEAER
☒	4541	806.3569	1610.6992	1611.7927	-1.0934	0	4	8.5e+02	1	GSMGDTGLPGLPGTPGAK
☒	5582	1076.4996	2150.9846	2151.2627	-0.2780	0	4	6.3e+02	1	LIIALGVAGLLITQGTDLSAGR
☒	7341	1160.5341	3478.5805	3477.6053	0.9752	0	4	2.7e+02	1	MNITTLTNSISTSSFSPNNTNGSSTETVNSDIK + Oxidation (M)
☒	8231	1101.6929	4402.7425	4403.3439	-0.6014	1	4	1.1e+02	1	HGFGPMFLLLCFPFTPSAAVNVVAGLSRIGTRPFILAAASGK + Oxidation (M)
☒	2398	355.0686	708.1226	708.3629	-0.2402	0	4	59	1	AAAAFMK
☒	8225	734.3381	4399.9849	4400.9467	-0.9617	1	4	1.4e+02	1	SSPITVQTSKDLAMADLSSFEETSADDFAMEMGLACVVCR + 2 Oxidation (M)
☒	8685	848.2469	5083.4377	5084.5223	-1.0846	1	4	97	1	GMEINYSVADAPNTVVLQQVTAGVHVRMAILFQLLIGGHSASETETDA + 2 Oxidation (M)
☒	8872	1186.9335	5929.6311	5930.8111	-1.1800	1	4	47	1	LMQANMTASEEDRLPENELLGQMSTFIFAGMDTTSGALAHTLQLLAEHPDVQDK + Oxidation (M)
☒	8403	904.0242	4515.0846	4516.2018	-1.1171	0	4	1.4e+02	1	ELPQQFFDVGIAEQHAVTMAAGMAANGMKPVVAIYSTFLQR + Oxidation (M)
☒	2401	355.0691	708.1236	707.2908	0.8328	0	4	59	1	NGDGAMK + Oxidation (M)
☒	8436	904.2239	4516.0831	4517.2326	-1.1494	1	4	1.4e+02	1	LQRALTQFMLDSHTDAGYEEVYVPFLVNQDSLHGTGQLPK
☒	8657	962.6519	4808.2231	4809.3742	-1.1511	1	4	1.1e+02	1	LATGLWVWGSLLMAAGTVQPSASQSVCAGTENKLSSLSDLEQQYR
☒	8042	846.9816	4229.8716	4230.9912	-1.1196	0	4	1.5e+02	1	VYGLDNVQAGEMVEFPGGIMGMALNLESDNVGVVIFGSDR + 2 Oxidation (M)
☒	8708	911.5956	5463.5299	5464.6076	-1.0776	1	4	72	1	VIEINNGLTMFVMALQGMKPHHAAKSWNFMNFQSANHSAVQMEFTTPK + 2 Oxidation (M)
☒	8829	972.9435	5831.6173	5830.9802	0.6371	0	4	52	1	DVVAALGMYPGQAPPLGAEGAGVVLETGPEVTDLAVGDAVMGFLGGAGPLAVVDQQLVTR + Oxidation (M)
☒	6631	705.8328	2819.3021	2818.2538	1.0483	1	4	4.4e+02	1	MMENSRPEDVSFQGRGVFELSDEK + 2 Oxidation (M)
☒	2624	881.8147	880.8074	881.5447	-0.7372	1	4	6.8e+02	1	LIQAKGPR
☒	5207	951.4548	1900.8950	1901.9054	-1.0103	1	4	7.5e+02	1	FQSNSTPMNQRAPSPPK + Oxidation (M)
☒	8052	1060.5120	4238.0189	4239.1377	-1.1188	0	4	1.7e+02	1	ALAGMHMLAVLDLSNNSLTGNIPADLGASPTLEMLNVSFNK
☒	8762	1148.9489	5739.7081	5738.7195	0.9886	1	4	64	1	SFLQSMVKLFSEEFSHDKPQLMEYIFIADNLAMFPYQMIDEPLYVMR + 3 Oxidation (M)
☒	2400	355.0687	708.1228	707.3450	0.7779	1	4	60	1	KSSDGSK
☒	1945	558.4190	557.4117	558.2874	-0.8757	0	4	1.3	1	AAGGAGR
☒	2349	676.9460	675.9387	676.3140	-0.3753	0	4	67	1	GSSDGVR
☒	8710	1096.4888	5477.4076	5477.7417	-0.3341	1	4	63	1	KEISLYINSPGGVVTSGMAIYDTMQFIRPPISTLCMGQAASMGSLLLTAGAK + 2 Oxidation (M)
☒	2521	787.3604	786.3531	787.4552	-1.1021	1	4	1.1e+03	1	SGEIKVR
☒	7073	1040.4705	3118.3897	3118.4450	-0.0553	1	4	3.5e+02	1	KQVTLTCMVTDFMPEDIYVEWTNNGK
☒	8051	1060.2610	4237.0149	4237.0038	0.0111	0	4	1.7e+02	1	TTDVTGVVTLPEGTEMVMPGDNTDMTVALIQPVAMEEGLK + 3 Oxidation (M)
☒	8520	759.1639	4548.9397	4549.1914	-0.2517	1	4	1.1e+02	1	GSQLDLVVAGTEAAVLMVESEADQLSEEVMLGAVVFGHTEMQK + 2 Oxidation (M)
☒	4934	885.3692	1768.7238	1769.8393	-1.1155	0	4	7.5e+02	1	VTSIIESMATAYEDPK + Oxidation (M)
☒	8635	1181.5156	4722.0333	4721.4746	0.5587	1	4	1e+02	1	YLELVISAIAAYFIFNEFPDKSTAHGAVIIIPATLFIMYSEK + Oxidation (M)
☒	8889	1190.1466	5945.6966	5946.0681	-0.3715	1	4	48	1	WRPRPAFRPWQVSMQPTPTMVTPMLQAPDLQAQQMQQLISAVSALTTKQNVK + Oxidation (M)
☒	2492	769.0312	768.0239	768.3258	-0.3019	0	4	2e+02	1	ASTMGMR + Oxidation (M)
☒	8027	1051.9673	4203.8401	4205.0263	-1.1862	1	4	1.6e+02	1	QTAAFVEAMDDDFNVANALAAIFELVRLANSYVDSGDVK + Oxidation (M)
☒	2412	713.9984	712.9911	713.4072	-0.4161	0	4	1.5e+02	1	SPKPGTK
☒	2283	634.8124	633.8051	634.3286	-0.5235	0	4	11	1	VSAGSSK
☒	8658	1203.3107	4809.2137	4809.5846	-0.3709	1	4	1.1e+02	1	YRSIMPKPIMVIPTLASLASPTTLQSQMLGGLGQDVLLNNSLTPK + Oxidation (M)
☒	8738	1148.5431	5737.6791	5737.8933	-0.2142	1	4	65	1	ALLMSSTATMVKAFDEAINDGVDVLSISLASAAPFRPIDSITGDLELGSFHAVMK + Oxidation (M)
☒	7916	822.5820	4107.8736	4107.9084	-0.0348	1	4	1.8e+02	1	MGLYGQACPSVTSLRMTSELESSLTSMDWLPQLTMR + 2 Oxidation (M)
☒	8733	947.1521	5676.8689	5675.7193	1.1496	1	4	68	1	TNSSNPTPPAPNNPVLYVDENDIHVGNHIYRATFPSTDEPPTDVYLNITGGR
☒	7917	685.8163	4108.8541	4108.9445	-0.0904	0	4	1.8e+02	1	MYAQGVTQYGQMTAGSYCYIGPQGIVHGTTITLLNAGR + 2 Oxidation (M)
☒	8020	1048.7468	4190.9581	4190.1206	0.8375	1	4	1.8e+02	1	VVVAMSGGVDSSAVAAQLADEGYDVVGVTLQLYDHGAALAKK + Oxidation (M)
☒	6394	889.7717	2666.2933	2667.1799	-0.8866	1	4	5.1e+02	1	EYSSMDVFWQEGKASGAVYSGEPK + Oxidation (M)
☒	8268	886.7927	4428.9271	4427.9841	0.9431	1	4	1.3e+02	1	ESLRDVMADLHMTEGFDVGLEMSGVPSAFTSMLEAMNHGGK + 2 Oxidation (M)
☒	2351	680.4244	679.4171	680.2799	-0.8628	0	4	49	1	QMSAASA + Oxidation (M)
☒	7686	1273.6476	3817.9210	3816.8386	1.0824	1	4	2.4e+02	1	EQMLALEGRLPNSLVACIGGGSNAMGLFHPFLDDR + 2 Oxidation (M)
☒	2678	457.5038	912.9930	913.4869	-0.4938	0	4	9e+02	1	ISGPADVQK
☒	5526	1048.4470	2094.8794	2094.9508	-0.0713	0	4	6.3e+02	1	YPFGGGAQSDEAHHQPLER
☒	8821	967.1271	5796.7189	5797.7099	-0.9909	1	4	62	1	LRAGPQAVLASGSGFCDNMLADLGLIGTIGEDDEVPVEPESDSGDEEEEGPIVLGR + Oxidation (M)
☒	5527	1048.4481	2094.8816	2095.0374	-0.1558	1	4	6.3e+02	1	GLEVIEARWLFDVDYDR
☒	7270	1135.4875	3403.4407	3403.6841	-0.2434	1	4	2.6e+02	1	CLNIVFTSMFSMECVLKIIAFGVLNYFR + 2 Oxidation (M)
☒	7844	808.5607	4037.7671	4036.8933	0.8739	1	4	1.8e+02	1	QSNLFENVNSNNNNNNNNNGITVLLFENKINEMK + Oxidation (M)
☒	8146	860.8111	4299.0191	4300.1094	-1.0903	1	4	1.7e+02	1	GFKAMIFFMALYLVALGSGCVKPNMIAHGADQFSQSHPK + 2 Oxidation (M)
☒	8845	975.9454	5849.6287	5850.6291	-1.0003	1	4	57	1	SEAATPDLLVDPGCGQCFVFDESASVPGDAYACGLLMDARTGVVGASLDMLVCDR + Oxidation (M)
☒	2284	635.0822	634.0749	633.3446	0.7303	1	4	11	1	GTSKGGK
☒	8707	1093.7128	5463.5276	5463.6432	-0.1156	1	4	75	1	DNRQHIAVIGDGALTGGMAFEAMNHAGVTNSNLLIVLNDNCMAIDPNVGALK + 2 Oxidation (M)
☒	2499	773.3844	772.3771	771.4351	0.9420	1	4	1.1e+03	1	VVRAGDR
☒	7734	789.7330	3943.6286	3943.0634	0.5652	1	4	1.7e+02	1	SWIVMPHYTSLNLVYKLTPSFNELNLTLFIFDK
☒	5764	782.3074	2343.9004	2344.1403	-0.2399	0	4	4.7e+02	1	SLGEACGLQVAAASVALMDPGEAK
☒	8690	1019.4923	5092.4251	5093.5478	-1.1227	1	4	1e+02	1	GSSDFIGVIHYLTALVTNIDINPSLSGIPDFNSDMGESINILSMRVR + Oxidation (M)
☒	8437	904.2240	4516.0836	4516.3249	-0.2412	0	4	1.4e+02	1	VDLWFVMSLIAILALGIVMVASASISVSESIHNTPYFFMGR + 2 Oxidation (M)
☒	8871	847.9580	5928.6551	5927.9311	0.7239	0	4	56	1	GWYSTITLAVYGILTNSVTQPIASPTLPCEPAGPEMCNLSTEAHNVLLQEEVLK + Oxidation (M)
☒	7607	937.4133	3745.6241	3744.7016	0.9225	1	4	2.2e+02	1	AIEAGDTETGITIMQMDEGLDTGDMLLSERESIR + 3 Oxidation (M)
☒	7854	1010.7011	4038.7753	4037.9740	0.8013	1	4	1.8e+02	1	DYDVIYKLLEDIQMAMEGLLEPELVEEALGEAEVR + Oxidation (M)
☒	8790	1160.1500	5795.7136	5794.8862	0.8274	0	4	65	1	GESSIENLIDVIQEYDQSELGVEALAAFALTGMKPPPLGAHEMSIVPPSPAPSHR
☒	2306	645.2856	644.2783	643.3653	0.9130	1	4	64	1	AGGKSPK

☒	2402	355.0692	708.1238	708.3442	-0.2204	0	4	64	1	SASGYPK
☒	8616	669.5740	4679.9671	4679.3768	0.5903	1	4	1e+02	1	LGADAAMLVTPYYNKTSQAGLVAHFTAVADAVDLPIMLYNVPSR + Oxidation (M)
☒	5238	961.3004	1920.5862	1920.0581	0.5282	1	4	5.4e+02	1	VESLRPQAAYIKQYVR
☒	8765	823.9723	5760.7552	5759.8413	0.9139	0	4	70	1	FSNYSGWLADPTHVKPSAQVVWPIFGQEILNGDMGAGFNGIQITSGLFHVWR + Oxidation (M)
☒	8830	834.1163	5831.7632	5832.7903	-1.0271	0	4	63	1	VITEALGGAGLFGVEFFIQGDTVYFSEVSPRPHDTGMVTMATQNMSEFELHVR + Oxidation (M)
☒	2390	708.1276	707.1203	706.3650	0.7553	0	4	77	1	ATAPYGK
☒	4211	756.5542	1511.0938	1511.8395	-0.7456	1	4	6.7e+02	1	SRPPLWKMVVQR + Oxidation (M)
☒	6296	1307.0824	2612.1502	2611.3170	0.8333	0	4	5e+02	1	YYLPVGANISVAEDSFVNAGDVIAK
☒	7272	852.1143	3404.4281	3403.5813	0.8468	0	4	2.6e+02	1	GEGMLIGSQASGMFLVNSESDDSPYVAARPFR + Oxidation (M)
☒	4821	852.8458	1703.6770	1703.8110	-0.1340	0	4	8.2e+02	1	LVDDMVNEIGDMLPK + Oxidation (M)
☒	8138	1074.5118	4294.0181	4292.9711	1.0470	0	4	1.7e+02	1	NNDGFTAFMNIFGNNYFDYIVPVIQIMLDYGADINDK + 2 Oxidation (M)
☒	8569	925.5952	4622.9396	4622.3578	0.5818	1	4	1.1e+02	1	QGDNVSIGLLDYPVLMAADILLYDADLVPVGEDQKQHLELAR + Oxidation (M)
☒	8647	1185.5247	4738.0697	4738.3476	-0.2779	1	4	1.2e+02	1	QISPFDLHLIPVNMKSEEQVAFAETLYSSLQDAGFSVLIDDR + Oxidation (M)
☒	8192	1091.0028	4359.9821	4359.1928	0.7893	1	4	1.6e+02	1	AAYTFSVGLIASQVYTPMAAAMAAGMVPPIGMTVATWIARNK + 2 Oxidation (M)
☒	8514	758.5027	4544.9725	4545.1918	-0.2192	1	4	1.3e+02	1	FLVNSFLKGGGGGGGGGGLGGGLGNVLGGLISGAGGGGGGGGGGGGGGGGGGGTAMR + Oxidation (M)
☒	5067	924.9114	1847.8082	1846.8681	0.9402	0	4	8.4e+02	1	AVMGAMKPGVWWPDMR + Oxidation (M)
☒	7163	1089.3291	3264.9655	3264.4380	0.5275	1	4	2.7e+02	1	SMTNMNVSMMGRQGTSIQQAYGGSDANLQR + 2 Oxidation (M)
☒	8803	966.9606	5795.7199	5794.5599	1.1600	1	4	67	1	AELTSDKDMYLDNNSIEEASGVYPIDDDDYASASGSGAGEDGESPELTTSRPIPK + Oxidation (M)
☒	8855	979.6121	5871.6289	5871.9354	-0.3065	1	4	56	1	GAMAVWVEQMVALREAGVELVLVSSGAVAAGMSQLGWTARPSAMNELQAAASIGQMR + Oxidation (M)
☒	8044	1058.4794	4229.8885	4230.1480	-0.2595	0	4	1.7e+02	1	DEIVSYLCDLAPEAPPPTLPPDMAQVTVGPGLLGVSTLGPK + Oxidation (M)
☒	8139	1074.5139	4294.0265	4293.7930	0.2335	0	4	1.8e+02	1	VGMGPGNQMGGMMGGPNQMQGPGAGMVGGMPGQMGVGMGPGGMSGGK + Oxidation (M)
☒	7436	898.9109	3591.6145	3590.6408	0.9737	1	4	2.9e+02	1	YYDVRTMTMSEVGLDGGFWGDLYIVMGDSLGK + Oxidation (M)
☒	2397	355.0685	708.1224	707.2908	0.8316	0	4	66	1	NGDGAMK + Oxidation (M)
☒	2504	778.0576	777.0503	776.4432	0.6071	0	4	5.1e+02	1	AVSLYPK
☒	8699	858.4099	5144.4157	5143.5500	0.8657	0	4	99	1	AGALPADVGVGGAAALGIGAVAAQGGGSYVADAGAGHPGAGSGSGYASWAPSQAPLGLR
☒	8844	1170.9329	5849.6281	5848.8620	0.7662	1	4	61	1	YSVIVIEYGVPDVGPLIQMPAALSFPMNMETYDWGFSSEPEPHIGGRSLVTPR
☒	2367	686.4673	685.4600	686.3963	-0.9362	1	4	1.9e+02	1	DIGAKAL
☒	4002	725.5431	1449.0716	1448.7545	0.3172	1	4	7.4e+02	1	DGVTSIQMDIKVK + Oxidation (M)
☒	4562	811.0737	1620.1328	1620.6483	-0.5155	0	4	6.7e+02	1	GFHMECCDPPLSR + Oxidation (M)
☒	8839	836.5255	5848.6276	5847.9788	0.6488	0	4	60	1	NHSLALLVCTAIATSLDAMAIGVGLAFLQVNILHTAMVIGCATMIMVTLGMMIGR + 4 Oxidation (M)
☒	7542	1225.1874	3672.5404	3672.9048	-0.3644	0	4	2.3e+02	1	AMGIDDHWFSLGDSIILTVTGQIAFMPVLVLAAR + Oxidation (M)
☒	8272	1110.0239	4436.0665	4437.1620	-1.0956	1	4	1.7e+02	1	MLSPANGEQIHLVNYVEDYLDSIESLPFDLQRNVSLMR + 2 Oxidation (M)
☒	7344	870.6530	3478.5829	3478.6782	-0.0953	1	4	3.1e+02	1	LTLPFVDMSKAAAMDGTSTTLNYATALMGGNLR + 3 Oxidation (M)
☒	2403	709.8320	708.8247	708.3555	0.4693	0	4	61	1	AAGATYR
☒	2332	662.3331	661.3258	660.3555	0.9703	0	4	96	1	AAGTSVR
☒	8798	828.9670	5795.7181	5796.7795	-1.0614	1	4	69	1	IVANRLTSPSSALVNASVAFECWINFGTDVAYLWDFGDGTVSLGSSSSSHVYSR
☒	8927	1260.1874	6295.9006	6295.3121	0.5886	1	4	45	1	RSFYVYAGILATLNLLQGLGSALLCANIIVGLCCVDATTFLYFSFFAPLIYVAFLR
☒	2279	634.2743	633.2670	632.3242	0.9428	0	4	26	1	SGGSGLR
☒	7810	801.7354	4003.6406	4003.1050	0.5357	1	4	1.7e+02	1	MPEILINKPVEDELVESYLLYSMSVIVGRAIPDVR + Oxidation (M)
☒	8809	828.9682	5795.7265	5795.9686	-0.2421	1	4	70	1	FMIPSIFAYGLLQCFNRFLQAQNNVFPVVFCSGVTTSLHVLLCWVLVFK + Oxidation (M)
☒	7441	898.9146	3591.6293	3591.9467	-0.3174	1	4	3e+02	1	MKLVLAIVLILMLLSLSTGAETSDNSASSSATALR + Oxidation (M)
☒	7247	845.0994	3376.3685	3375.7173	0.6512	1	4	2.7e+02	1	TLLAKAVAGEAGVPFFSTSGSSFVEMFVGVGAAR + Oxidation (M)
☒	2393	355.0681	708.1216	707.3602	0.7614	0	4	68	1	ATGGGAFK
☒	2434	729.6898	728.6825	729.3116	-0.6290	0	4	4.2e+02	1	ADAMHAV + Oxidation (M)
☒	8777	966.7916	5794.7059	5794.7652	-0.0593	0	4	73	1	GYEGAAVGAALGAGITGYNSSSAGATLGVGLAAGLVGMAADAMVEDVNYTMITDVQIAER + 3 Oxidation (M)
☒	7964	1377.5587	4129.6543	4129.0558	0.5985	1	4	1.5e+02	1	AMRVLAMVVAVYTICFLPSIIFGMASMVAFWLSACR + 3 Oxidation (M)
☒	8012	838.9606	4189.7666	4190.9561	-1.1894	0	3	1.7e+02	1	TGMEEAIITGEATIEGYPVVIAVMDFHFMGGSMGSVVGEK + 2 Oxidation (M)
☒	2422	724.3054	723.2981	724.4847	-1.1866	0	3	1.5e+02	1	IVVGLPK
☒	2322	655.6849	654.6776	655.2483	-0.5707	0	3	17	1	MTGSSAS + Oxidation (M)
☒	4882	870.7704	1739.5262	1738.9253	0.6009	1	3	6.7e+02	1	ENIYSLNQLKNYLK
☒	2501	775.5978	774.5905	775.4010	-0.8105	0	3	1e+03	1	ALSGCIR
☒	8756	957.6223	5739.6901	5738.6701	1.0200	1	3	74	1	DAGFGAVHRVGTPPVPAPGTTEGSFEISNSSTATTGGSALSATGGMGSASASSSMPSTPVR + 2 Oxidation (M)
☒	7806	1001.6703	4002.6521	4003.0275	-0.3754	1	3	1.9e+02	1	SYLHHLHRAGEILGSMLNTVHNLHYYQTLTAELR + Oxidation (M)
☒	5091	932.3698	1862.7250	1862.8257	-0.1007	1	3	7.7e+02	1	DFADWECGKLTAADHK
☒	8692	1019.4962	5092.4446	5093.5478	-1.1032	1	3	1.1e+02	1	GSSDFIGVIHYLTALVTNIDINPSLSGIPDFNSDMGESINILSMRVR + Oxidation (M)
☒	8414	904.0255	4515.0911	4516.2439	-1.1528	1	3	1.7e+02	1	DNTHVLVVGDGVNDAPAMAAAHTSIAMGRAGADLTVQTADVVTIR + Oxidation (M)
☒	8474	755.6694	4527.9727	4527.2613	0.7114	0	3	1.5e+02	1	WWLLPLIAALASGSFWFFSTPEVETTAVQEFQNQMNQLK
☒	6632	940.7766	2819.3080	2818.3926	0.9154	0	3	5.3e+02	1	YSGDEGGVDLYIGGVEHAVLHLLYSR
☒	7135	1075.1388	3222.3946	3221.3327	1.0619	1	3	3.5e+02	1	NSSSINISFDNGRSSDEDGMDEEDEAFVR
☒	8015	699.3026	4189.7719	4190.1214	-0.3495	0	3	1.8e+02	1	SAVNTVGLQTQTNGYLPHAIMGWISTSLVPVSVAISMGMK + 2 Oxidation (M)
☒	8854	839.6667	5870.6160	5871.1534	-0.5374	1	3	61	1	EIHHLVFHVANVCFAVGLLIPTTLHLHMILLRVMLSLGCTLYVVWATLYR + 2 Oxidation (M)
☒	8869	735.3376	5874.6426	5875.8284	-1.1859	1	3	61	1	SLAANLNDVMCSGGDYSFALPAGQWPVMKGGAATLHTAGVTLSTQFTDYVSLNSLR + Oxidation (M)
☒	2671	909.3965	908.3892	907.3957	0.9935	0	3	1.3e+03	1	MEQLEDK + Oxidation (M)
☒	8897	1190.7498	5948.7126	5949.8865	-1.1739	1	3	57	1	VPVVVDAGIGTPSEAAQAMEMGADAVLINSAIAMAANPVAMAQAMGMATQAGRLAYLSGR + 4 Oxidation (M)
☒	8039	1057.9749	4227.8705	4227.0198	0.8507	0	3	1.9e+02	1	AVLEILQPNAGEDTAEAIAAAEAGDEMDELMALAEGTTRPK + Oxidation (M)
☒	8888	848.3862	5931.6525	5931.8421	-0.1896	1	3	52	1	MQPGGQPDMSALLAQAQQMQQQLMAAQQEMAEAEVTGQAGGGLVTATVKGTGEVVGLK + 4 Oxidation (M)
☒	8686	1271.8674	5083.4405	5084.5223	-1.0818	1	3	1.2e+02	1	GMEINYSVADAPNTVVLQQVTAGVHVRMAILFQLLIGGHSASETETDA + 2 Oxidation (M)
☒	5357	1003.5814	2005.1482	2005.0952	0.0531	1	3	8.2e+02	1	MGTIVLKMTAEQISVLQK + Oxidation (M)
☒	6633	705.8343	2819.3081	2819.2690	0.0391	1	3	5.4e+02	1	ALQEYAAKFDPCQCAPCPNNGRPR
☒	2373	690.1250	689.1177	688.3755	0.7422	0	3	1.7e+02	1	SVGIEGK
☒	4878	869.4515	1736.8884	1736.8693	0.0191	0	3	1e+03	1	DQLEAHLDETLAQVR
☒	8843	1170.9325	5849.6261	5848.8620	0.7642	1	3	67	1	YSVIVIEYGVPDVGPLIQMPAALSFPMNMETYDWGFSSEPEPHIGGRSLVTPR
☒	8412	904.0252	4515.0896	4514.2205	0.8691	1	3	1.7e+02	1	IGVMVEFEGDDTAAKDVAMHIAAMKPVALSMADVPAESIAIER + Oxidation (M)
☒	8751	820.8209	5738.6954	5738.0260	0.6693	1	3	77	1	DWHELLILTNSALVPPASSYVSIVVGTHISAAPVLGPVQVWANYAIVSNEERR
☒	2628	884.0595	883.0522	882.5035	0.5487	1	3	8.3e+02	1	AGRVIDPR
☒	7973	827.5975	4132.9511	4131.9848	0.9664	1	3	2.3e+02	1	ADGLPLAGWVGNLIDPGLSFDEVADEACYRDNLATLER
☒	7551	922.9067	3687.5977	3686.7433	0.8544	0	3	2.6e+02	1	AIQVGDGDIFIIGGVEHMGHVPMSHGVDFHPGMAK + 2 Oxidation (M)
☒	7439	898.9131	3591.6233	3591.9467	-0.3234	1	3	3.3e+02	1	MKLVLAIVLILMLLSLSTGAETSDNSASSSATALR + Oxidation (M)
☒	8497	1133.5045	4529.9889	4530.0919	-0.1030	1	3	1.5e+02	1	LIMAGNYEEALSVAKMQVEMGAQVLDINMDDGMLDGPSAMTR + Oxidation (M)
☒	8796	1449.9362	5795.7157	5794.8044	0.9113	1	3	76	1	DMSYGDYLHLDELLGAQRPLSPEHNEMLFIVQHQTSELWMKLMLHELR + 2 Oxidation (M)
☒	8837	975.6110	5847.6223	5848.5471	-0.9247	1	3	66	1	SQDLDPMATDASPMAINMTPTVEQGEGEEAMKDMDSDQQYEKPPPLHTGADWK + Oxidation (M)
☒	8882	848.2433	5930.6522	5929.6973	0.9548	1	3	57	1	ISESAAKASEQLVTGATQESATFSNDGLSGTMALSVQDTVGQEVLQMSDMMGSLSDR + 4 Oxidation (M)
☒	5564	1068.2094	2134.4042	2135.0721	-0.6679	1	3	5.7e+02	1	EMFGIEFAPPVVAADGSVRK + Oxidation (M)
☒	7290	858.3757	3429.4737	3428.5175	0.9562	0	3	3.2e+02	1	SYAPAPHPMAPPSPSTNSSSNNSSNNSSGEQLSK
☒	2435	729.9129	728.9056	728.3817	0.5239	0	3	4.5e+02	1	TAQQGPK
☒	2569	822.0975	821.0902	821.4283	-0.3381	1	3	8.3e+02	1	DKGEFVK
☒	2591	849.6689	848.6616	849.4015	-0.7398	0	3	1.3e+03	1	DNVVMTR + Oxidation (M)
☒	5237	961.1378	1920.2610	1919.9993	0.2618	0	3	6.5e+02	1	QVVSGIAQFYAPDELVGK
☒	6269	1306.5801	2611.1456	2612.1638	-1.0181	1	3	5.8e+02	1	AGMDRVTGDHMGMLATVMNALAMR + 4 Oxidation (M)
☒	8828	727.8299	5814.5810	5814.9041	-0.3231	0	3	66	1	WGATLMNSFLFNVGLILLCSISVIQFCATAFGYYAQATAAQEIFGHTLQSLR + Oxidation (M)
☒	1939	556.4411	555.4338	556.2718	-0.8379	0	3	0.48	1	GGGGGPR
☒	7332	870.4017	3477.5777	3476.6017	0.9760	0	3	3.5e+02	1	AAIGVGVSGFFMETHPNPNEAFSDGPNMVPIDK + 2 Oxidation (M)
☒	8904	869.8134	6081.6429	6081.1420	0.5009	1	3	52	1	IPLLRPCTPKGIMTLLESTGADLYGMDAVVVGASNIVGRPMAMELLLAGCTVTVTHR + Oxidation (M)
☒	8944	1307.5889	6532.9081	6532.0132	0.8949	1	3	43	1	MLVLFFLESELHEHAAYLVDSLWESSQELLKDWECMTELLVEEPMQGEEVMSER + 3 Oxidation (M)
☒	5144	936.9128	1871.8110	1873.0091	-1.1981	1	3	9.5e+02	1	RSLLLDMDVAIQQLSR + Oxidation (M)
☒	7295	1145.4764	3433.4074	3433.7129	-0.3055	1	3	2.8e+02	1	FPLEESAPPGPRPLPWPPGNDEAAKMQAPPPK + Oxidation (M)
☒	8794	1160.1503	5795.7151	5794.8862	0.8289	0	3	78	1	GESSIENLIDVIQEYDQSELGVEALAAFALTGMKPPPLGAHEMSIVPPSPAPSHR
☒	2648	897.2142	896.2069	896.4828	-0.2759	1	3	9.1e+02	1	SRHIEQK
☒	7759	991.4601	3961.8113	3961.0118	0.7995	1	3	2.5e+02	1	DTLRFEACLPLYGHELSATISPIEAGMGFAVKPQVK + Oxidation (M)
☒	8494	1510.9972	4529.9698	4530.2734	-0.3036	1	3	1.5e+02	1	NMQEALALLLREDPSLHVTIDEDSGQTLLSGMGELHLEIAR + Oxidation (M)
☒	8867	979.9473	5873.6401	5872.8340	0.8062	1	3	63	1	NSLENQKDSILFNGVMGMLGNGIGAAASAATGSAVGVASSATGMVSSAGNAVLQIQGMQAK + 4 Oxidation (M)

☒	8622	937.2025	4680.9761	4680.3085	0.6676	1	3	1.2e+02	1	TLIASLPEALAETDFVCASVNVGSTRSGINMDAVAQMGEVVVAGSK + Oxidation (M)
☒	8739	1148.5447	5737.6871	5738.3325	-0.6454	1	3	81	1	LQLSMDPMLDMMGMQMLMEKYGDQAMAGMDHSQMMGHMGHGNMNHMNHGGK + Oxidation (M)
☒	8145	860.8104	4299.0156	4299.1941	-0.1785	0	3	2.1e+02	1	LTFGPQWAAEGVFMLFAVLFTFIGAQFIGMGLLGEYIGR + 2 Oxidation (M)
☒	2423	724.3054	723.2981	722.4075	0.8906	0	3	1.6e+02	1	HSAAPLK
☒	8031	1052.2152	4204.8317	4205.1970	-0.3653	1	3	2e+02	1	NVHSTKFVTYFCEQVLPILSTLTSPAESIDVQLEVLK
☒	8555	920.0468	4595.1976	4596.3472	-1.1496	1	3	1.7e+02	1	IFTMFSSFSMASLALPGMSGFVAEFVIFLGIITSPKYLVMSK + 2 Oxidation (M)
☒	2611	869.4027	868.3954	869.4494	-1.0540	0	3	1.3e+03	1	EPLGETPK
☒	8702	1293.5859	5170.3145	5171.5108	-1.1963	0	3	1e+02	1	MSQPVFPTASGIFLPDLHHDEVSAPQLVMLANVVVSAEASASEYSTEGK + Oxidation (M)
☒	2265	618.4558	617.4485	616.3544	1.0941	0	3	30	1	VSAAAAK
☒	8769	961.3002	5761.7575	5760.9715	0.7860	1	3	86	1	LPVEAAVVTAMGGVQGAFIGGLMGTLSPEMPQAGIDPQAMASLKQTQALVGGPLVQAR + 2 Oxidation (M)
☒	2428	727.3461	726.3388	726.4640	-0.1252	0	3	2.8e+02	1	VLGTPIK
☒	8788	828.9662	5795.7125	5795.8410	-0.1286	1	3	80	1	TVYPEIPFSGDFDLNALQHTIKDSEDLLLAQEVLSETTPSGLSNIEYWSWK
☒	8711	783.9435	5480.5536	5481.5232	-0.9696	1	3	95	1	FTGALPMMLADVTYGACCIDDYTAKEMGAEMIVHYGHSCLIPVSQTTLK + Oxidation (M)
☒	8880	989.4489	5930.6497	5931.7704	-1.1207	0	3	60	1	EENINNAINNADTPVMDALWQAYPHTLIEASGADVGLPDNQMGNSEVGHLTIGAGR + 2 Oxidation (M)
☒	2432	365.1640	728.3134	729.3909	-1.0774	0	3	6e+02	1	LEVVAAE
☒	2068	602.3151	601.3078	601.3184	-0.0106	0	3	31	1	GNGVGAK
☒	2338	664.2848	663.2775	662.3421	0.9354	0	3	65	1	VMAAGAK + Oxidation (M)
☒	8156	1076.5005	4301.9729	4302.1745	-0.2016	1	3	2.1e+02	1	IIELCSMGTYQFITDFEWYINILVKLSQIQDSIHGK
☒	8905	1014.7828	6082.6531	6081.8626	0.7905	1	3	55	1	STCSVLEQTDIEAVEALVCMSSWGQRSQMRPLTPVSDSGDVTTAVLMDTAAPDLPK + 2 Oxidation (M)
☒	2618	874.7155	873.7082	874.4508	-0.7426	1	3	1.5e+03	1	TKQQNEK
☒	8776	725.3449	5794.7010	5793.7745	0.9265	1	3	84	1	MSDKGGQQQPGVGMPINAAAGTAGGGPGTPVTVSNASGVPSGSSGAQASQMGALAVGSALSPSR + Oxidation (M)
☒	8825	829.1110	5796.7261	5797.8814	-1.1553	1	3	79	1	IPYPLQWGRPSFHGSDAFAMMAATYVAIVETTGSFIAASRFGSATHIPPSVLSR + 2 Oxidation (M)
☒	2534	797.0869	796.0796	796.4807	-0.4011	1	3	4.2e+02	1	GVTKAPPK
☒	7300	861.9061	3443.5953	3442.5942	1.0011	0	3	4.1e+02	1	ISADTTDNSGTVNQIMMMANSPEDWLSLLLK + 3 Oxidation (M)
☒	8771	962.6235	5769.6973	5768.7833	0.9141	1	3	83	1	ERGENIMFEGAQGTLLDIDHGTYPYVTSSNTTAGGTATGTGFGPLYLDYVLGITK
☒	8835	1170.5302	5847.6146	5847.9683	-0.3536	1	3	70	1	YHHLILYVLVISQESQEQLQARIQVNPNEVNAFMWLGPDVAAAVVATEDGTR + Oxidation (M)
☒	4889	872.5885	1743.1624	1743.7331	-0.5707	0	3	8.1e+02	1	QMIDSPDIDYMQEK + 2 Oxidation (M)
☒	8748	1148.7450	5738.6886	5739.6736	-0.9850	0	3	85	1	MADLSETTVVLPDHPDTGTTGAFLDLMGFFGASTWYPAAGVTVMGADEVAHTPPK + 3 Oxidation (M)
☒	8938	817.2443	6529.8962	6528.9434	0.9528	0	3	44	1	SEASAFGCIQADGMGTITEFVEKPENPPATPDDPNMTYASMGNYVFTTQALIDALLEDEK + 2 Oxidation (M)
☒	8945	824.2371	6585.8386	6587.0149	-1.1763	1	3	40	1	IVQMASMMGGVENLMENPLALQMIMSSSGASAGASAAGSSAAGSSAAGAGGGAGAAGAAGASASAGAGAGASARAGV + 3 Oxidation (M)
☒	6567	922.7244	2765.1514	2764.4065	0.7449	1	3	5.2e+02	1	LDRIMNGELDEIIDALIVHDQAQK + Oxidation (M)
☒	2355	682.8201	681.8128	681.3307	0.4822	0	3	29	1	AGHGNAR
☒	8759	957.6226	5739.6919	5740.5300	-0.8381	1	3	85	1	MITTMSLLLPGVAVNYYGDEIGMSDTYISWEDTQDPQGCGAGKENYQTMSR + 3 Oxidation (M)
☒	8463	1510.3290	4527.9652	4527.2407	0.7245	1	3	1.7e+02	1	DGATSFSEDTALVLEYYPSASMKSSEPWTLNQPLGVSVLPLK
☒	8801	828.9672	5795.7195	5794.5875	1.1320	1	3	83	1	SMFSLNEGEIIDSFQEFLSDFDTLTYCSYLCELIDISMAEGESNRDLFK + Oxidation (M)
☒	8775	1159.9467	5794.6971	5794.6463	0.0508	1	3	85	1	YFQEETQLELSPTADVLQMDEAFVCYIAMTRASTHVTFSYSLMGLNNDDK + Oxidation (M)
☒	8948	1026.1903	7176.2812	7176.6983	-0.4171	1	3	32	1	NQLHIADFEDFIQTDAAINPGNSGGPLLNIDGQVIGVNTAIVSGSGGYIGIGFAIPSLMANRIIDQLIR + Oxidation (M)
☒	8862	735.2092	5873.6154	5872.7625	0.8529	1	3	65	1	MMEQFPQGQDLLEQLESTYQGSASGILGQLNLYPREDEIFSQDTQHGPYLR + Oxidation (M)
☒	7237	1118.1562	3351.4468	3351.8046	-0.3579	1	3	3.7e+02	1	DGAEIGIAQAMQAFAARVLPSALMNPVLLKPK + 2 Oxidation (M)
☒	8926	1023.9963	6137.9341	6137.0216	0.9126	0	3	67	1	MPQQPSTAAEVTPAAWTALGIDAVVVGQVQPSADGSYVVSYQLVDTSGSAGSILAQNQYK + Oxidation (M)
☒	2391	708.8398	707.8325	708.4031	-0.5706	0	3	79	1	HGGVALR
☒	8772	1155.1447	5770.6871	5771.7719	-1.0847	1	3	83	1	MSTISGSLDELMPHDLINSKMITNTILEFFATGQLSQFMDQTNPLSEITHK + 3 Oxidation (M)
☒	8876	848.1011	5929.6568	5928.8229	0.8339	1	3	67	1	CWGAGGGGGATWLLLVVAGCVVCGSADVNVVMLQESQVDMNSSQQFCYKNVLIPK
☒	2285	636.3959	635.3886	636.2901	-0.9015	0	3	15	1	AAMGSGK + Oxidation (M)
☒	8695	1274.3646	5093.4293	5093.5478	-0.1185	1	3	1.3e+02	1	GSSDFIGVIHYLTALVTNIDINPSLSGIPDFNSDMGESINILSMRVR + Oxidation (M)
☒	2327	656.7620	655.7547	656.3969	-0.6422	0	3	43	1	GVIAAAR
☒	6895	995.4557	2983.3453	2982.5583	0.7869	0	3	5.4e+02	1	LLNQTIAEPLVDAETGEILVEAGTVMTR
☒	8793	966.9597	5795.7145	5795.8857	-0.1712	1	3	87	1	KPIHMYINSPGGSVTAGLAIYDTIQMISAPVSTWVIGQASSMGSLLLCAGEKGMR + 2 Oxidation (M)
☒	8705	1093.0879	5460.4031	5459.4885	0.9146	1	3	96	1	ALDLDVAGFLMMAHMNSPEGLAKQAQMMEAYGAHCVYVTDSAGALTMDGVR + Oxidation (M)
☒	2318	651.1581	650.1508	650.3136	-0.1628	0	3	12	1	AGGAYGR
☒	8900	994.6533	5961.8761	5961.7746	0.1015	0	3	75	1	VDLAHLQAIGDELGINGNPGDFIMMDALPNMNQWIDNNSELNAIYGCVEPYLR + 2 Oxidation (M)
☒	8853	836.9771	5851.7888	5852.7687	-0.9799	1	3	88	1	TSEQTILNMISQSSSDTTIAGLMSMSTSTTSAVPSLPVSEESSSNLTSVEMLPGKR + 3 Oxidation (M)
☒	8683	1008.2903	5036.4151	5035.4971	0.9180	1	3	1.4e+02	1	SIALFAGLALAASSAQEATILGGYTQKNATSDDIELLTQATSSANMYNK + Oxidation (M)
☒	8774	1449.6802	5794.6917	5794.8342	-0.1425	0	3	90	1	DNPMTLPISSGGPEGSMMASDYVTPGDPVLTLPTGPLSTSLGPSLGLPSAQSPSLCLK
☒	8780	828.8243	5794.7192	5794.5599	0.1593	1	3	91	1	AELTSDKDMYLDNNSIEEASGVYPIDDDDYASASGSGAGEDGESPELTTSRPIPK + Oxidation (M)
☒	3725	704.8057	1407.5968	1407.6670	-0.0702	1	3	1.4e+03	1	DPETKGLYWDGK
☒	8188	1090.7556	4358.9933	4359.1418	-0.1485	1	3	2.2e+02	1	SDVWSFGILLYEIITYGKMPYSGMTGAQVIQMLAQNYR + Oxidation (M)
☒	6906	995.7948	2984.3626	2983.3505	1.0120	1	3	5.6e+02	1	GASSMSSRSPVNLEPESDSVDSEWVFGK
☒	6721	1434.7136	2867.4126	2867.2259	0.1867	0	2	6.7e+02	1	MMSTESANSFTLIGEASDGGTMENLSR + 2 Oxidation (M)
☒	8885	1187.1399	5930.6631	5931.1572	-0.4941	1	2	69	1	DPTKGLSLVFLSVATSLDALAVGLSFSIMDYPIAFPCVMIGITALVLTSFGLWLGK + Oxidation (M)
☒	7015	1008.1060	3021.2962	3021.5038	-0.2077	1	2	5.1e+02	1	GVVTEEMAYVAKIENLPESLVMEEVAR + Oxidation (M)
☒	2614	872.0973	871.0900	870.4811	0.6089	1	2	1.3e+03	1	VAKDVDPK
☒	8936	817.1225	6528.9218	6529.9904	-1.0686	1	2	49	1	EQLYVTSGHLEWYADGMFPAMHIDAEYDADGAVRKPGQNYYLKPMNCPMHHLIYR + 2 Oxidation (M)
☒	8797	828.9668	5795.7167	5794.5599	1.1568	1	2	92	1	AELTSDKDMYLDNNSIEEASGVYPIDDDDYASASGSGAGEDGESPELTTSRPIPK + Oxidation (M)
☒	8784	828.9650	5795.7041	5796.7795	-1.0754	1	2	91	1	IVANRLTSPSSALVNASVAFECWINFGTDVAYLWDFGDGTVSLGSSSSSHVYSR
☒	8930	1287.8243	6434.0851	6435.1111	-1.0260	0	2	66	1	TIHTYHVEGAGGGHAPDIISLVQNPNILPSSTNPTRPFTTNTLDEELDMLMVCHHLSR + 2 Oxidation (M)
☒	7604	1249.2157	3744.6253	3745.8219	-1.1967	1	2	3.1e+02	1	TGFYMSLIGQPTESKVADSWLAAMVDVTNVIDQK + 2 Oxidation (M)
☒	2383	702.1052	701.0979	700.3392	0.7588	0	2	3.2e+02	1	DPDAVGK
☒	8902	1011.3183	6061.8661	6062.7849	-0.9187	1	2	77	1	KGEPITMLTAHDFPSAHVADAAGMDMILVGDSLAMVALGMQDTSEVTLDDMLVHCR + 5 Oxidation (M)
☒	8709	912.0966	5466.5359	5466.5591	-0.0231	1	2	1.1e+02	1	TWRYETQVIATVLSMDQENDPDMLSMVGASAALCISDLPFFTPIACVR + 3 Oxidation (M)
☒	2344	671.8342	670.8269	670.4377	0.3892	0	2	1e+02	1	VLAIAGK
☒	8819	1160.3501	5796.7141	5795.9841	0.7300	1	2	89	1	EQGVRFEVAHLSNSSATMARPDLTFDLVRPGIAVYGLSPVPALGDMGLVPAMTVK + Oxidation (M)
☒	7739	987.1656	3944.6333	3944.7940	-0.1607	1	2	2.5e+02	1	YGDGECIMKLCNTLFSENSAEGDPGLNLMQLDPLK + Oxidation (M)
☒	5539	1055.9977	2109.9808	2111.1222	-1.1413	0	2	1e+03	1	LINIAINQISASQEQVNEK
☒	8928	1282.4207	6407.0671	6406.8344	0.2327	1	2	62	1	MDPKPINEGMLLEDHDHGMPEEEEITEDDMNGIHNIEEDTRTISEIAMELHHPNK + 2 Oxidation (M)
☒	8847	732.3552	5850.7834	5850.8107	-0.0273	1	2	95	1	QFSENIYEAFLVGKPSDCTLPSAQCNEYSEEEELVKGVLMAGLYPNLIQVR + Oxidation (M)
☒	8693	849.9106	5093.4199	5093.7564	-0.3364	1	2	1.4e+02	1	IILFLFLMSVAGIFNYYLIFFFGSDFENYIKTISTTISPLLLIP
☒	7983	831.7385	4153.6561	4153.2797	0.3764	1	2	2.1e+02	1	LGKYVTMMPYTVISGFMSGIGIILVILQLAPFLGQASPK
☒	8768	961.2988	5761.7491	5761.6835	0.0657	1	2	1e+02	1	LGARTHLASIHSEEEHQAIMSMLASSQPYSESEEEVADEEVWIGLHRPMGR + 2 Oxidation (M)
☒	7385	1179.2079	3534.6019	3535.4614	-0.8595	1	2	4.4e+02	1	AAIDKSYDKPMVDDDDEGMETLEEDTEESSR + Oxidation (M)
☒	3483	1307.0829	1306.0756	1305.7292	0.3464	1	2	1.1e+03	1	FGQSVKGLLTEK
☒	7538	1222.2273	3663.6601	3664.6807	-1.0206	1	2	4e+02	1	SLEDLETWVTMDDFEVIGYESHEAIKYPFSV + Oxidation (M)
☒	8860	1175.5336	5872.6316	5872.1538	0.4778	0	2	82	1	GMEGLLQNSTNFVLTGLITHPAFPGLLFAVVFSIFVVAITANLVMILLIHMDSR + 2 Oxidation (M)
☒	8937	933.7117	6528.9310	6528.0910	0.8399	1	2	54	1	YMNHGQFIEMVWTTFPAVMLLIMAFPSFMLLYICDEVMAPAMTIKAMGLQWYWK + 4 Oxidation (M)
☒	8856	979.6131	5871.6349	5870.8279	0.8071	1	2	84	1	DIEGAMAPSNLTVNILTTSTHPEMSSWLLCEVSGFFPENIHLMWLGVHSKMK + Oxidation (M)
☒	8832	973.1376	5832.7819	5831.6294	1.1525	0	2	92	1	RPRPAGATGGGGGGVHANGGSVFGHPTGGTSTPAHPPPYHSQGGSESMGGSDSSGFAEGSFR
☒	8857	735.0839	5872.6130	5872.7999	-0.1870	1	2	82	1	LVSIPTPHPNFNNINNINNNNNNNNNNNSYNGNIETNTPISLPPPICNKYK
☒	2295	636.3976	635.3903	634.3035	1.0869	1	2	17	1	GSRTGAS
☒	8807	966.9608	5795.7211	5795.8857	-0.1646	1	2	1e+02	1	KPIHMYINSPGGSVTAGLAIYDTIQMISAPVSTWVIGQASSMGSLLLCAGEKGMR + 2 Oxidation (M)
☒	8190	1090.7572	4358.9997	4358.1888	0.8109	1	2	2.5e+02	1	GHYGSWEMITGDEIQILSNLVMEELLPTLQTDLLPKMK + Oxidation (M)
☒	7560	923.4069	3689.5985	3689.8804	-0.2819	1	2	3.6e+02	1	SEPIPSFIDGIGNGLGYGFVLMTVGFFRELLGSGK + Oxidation (M)
☒	8757	957.6224	5739.6907	5739.7820	-0.0913	1	2	1e+02	1	MSTISGSLDELMPHDLINSKMITNTILEFFATGQLSQFMDQTNPLSEITHK + Oxidation (M)
☒	8890	1190.5446	5947.6866	5946.9100	0.7766	0	2	74	1	YYAGGPTVVSQCVVYIFGVEINTASFAMYTFSVSVFIQAILIISMSGAADHGSHR + Oxidation (M)
☒	8836	975.6107	5847.6205	5848.6987	-1.0781	1	2	89	1	INLNSNLDLNPNPLYAEEGEQEEEEEEEEDREVDVDLHIGLPGFGKPSNDAK
☒	2356	683.2106	682.2033	683.2875	-1.0841	0	2	48	1	EPGHASS
☒	8934	925.9934	6474.9029	6475.9183	-1.0155	0	2	58	1	MNQENPPPYPGPGPTAPYPPYPPQPMGPGPMGGPYPPPQGYPYQGYPQYGWQGGPQEPPK + 2 Oxidation (M)
☒	2375	694.2949	693.2876	692.3341	0.9536	0	2	1e+02	1	SEASATK
☒	5471	1023.7240	2045.4334	2045.0252	0.4083	1	2	8e+02	1	DSVSVAPCEPFGKEGVVIR
☒	2297	636.3980	635.3907	634.3035	1.0873	1	2	18	1	GSRTGAS
☒	2341	671.1740	670.1667	670.3398	-0.1731	0	2	86	1	TAAGSHK
	8811	1450.1836	5796.7053	5797.8095	-1.1042	0	2	1e+02	1	ISMLTAYDASMSALLDQAGIDILLVGDSLGMTVLGYDSTVPVTMADMVHHMAAVR + 2 Oxidation (M)

☒

☒	3353	1260.8409	1259.8336	1258.6670	1.1666	0	2	1.7e+03	1	SPQFLGLQNQK
☒	8806	966.9608	5795.7211	5795.0769	0.6442	1	2	1.1e+02	1	PNWGQVMFQDAASSVMLQLVSFHDHALLVLTLVLTVVGYALLALMLNKQVNR + 2 Oxidation (M)
☒	8785	1160.1487	5795.7071	5795.4445	0.2626	0	2	1.1e+02	1	YDLGCSVIGDVTDGNYVCTFDGETVVDCDAEYLADGAPMNDLDHVEPTQPDR + Oxidation (M)
☒	2427	726.4413	725.4340	726.3660	-0.9320	0	2	3e+02	1	HSLGGEK
☒	2340	671.0449	670.0376	670.4014	-0.3637	0	2	77	1	ISGGPLK
☒	5267	972.4498	1942.8850	1943.0914	-0.2063	0	2	1.3e+03	1	NLSLVLAAFCLGIASAVPK
☒	8778	828.8235	5794.7136	5795.8857	-1.1722	1	2	1.1e+02	1	KPIHMYINSPGGSVTAGLAIYDTIQMISAPVSTWVIGQASSMGSLLLCAGEKGMR + 2 Oxidation (M)
☒	2300	642.2028	641.1955	641.3860	-0.1905	1	2	42	1	AAPGKAK
☒	2436	731.3642	730.3569	730.3973	-0.0404	0	2	1e+03	1	NAAAGSLK
☒	7446	1198.5479	3592.6219	3593.4967	-0.8749	0	2	4.8e+02	1	GADHSSAPPADGDDEEMMATEVTPSAMAELTDLGK + 3 Oxidation (M)
☒	8032	1052.2159	4204.8345	4205.0351	-0.2006	0	2	2.7e+02	1	TTGVLAGFFGAGEAGYSHADLAGVPLTGMNPLCPYLNVDPR
☒	2319	651.7683	650.7610	650.2871	0.4739	0	2	11	1	GSGESSK
☒	7930	1370.6290	4108.8652	4108.1028	0.7624	1	2	3.1e+02	1	MCASVFFIIIYMFYIHYSHKSVLLNEAIQSLNIK + Oxidation (M)
☒	8700	1031.4698	5152.3126	5151.5798	0.7328	1	2	1.5e+02	1	VFGVGELGMANTTPAAAMVSVFTDSDPELAVGIGANFPSEQLHHKVAVVR + Oxidation (M)
☒	8931	1290.6233	6448.0801	6447.4396	0.6406	0	2	76	1	VIFTLAPMIAFTSMLIAFAIVPITPTWGVADLNIGILFFLMMAGLAVYAVLFAGWASNNK + Oxidation (M)
☒	7517	1213.6315	3637.8727	3638.8628	-0.9901	1	2	4.7e+02	1	VAVLSLHTSPWAQPGTGDAGGMNVYIRNTSTVLAR
☒	2477	759.8525	758.8452	758.3997	0.4456	0	2	1e+03	1	GLMPTPK + Oxidation (M)
☒	2380	699.3007	698.2934	698.3711	-0.0777	0	2	2.5e+02	1	GAIADPR
☒	2538	799.5934	798.5861	799.4803	-0.8942	0	2	1.4e+03	1	EAGIIIGK
☒	2481	765.3816	764.3743	763.3898	0.9845	1	2	6.7e+02	1	GDAKVMK + Oxidation (M)
☒	8831	973.1189	5832.6697	5832.9138	-0.2441	1	2	95	1	LGILGGLSILGTSGIVRPFSCAAYIASIHQGIDVATTNGYRHIAACTGNASEDTMR
☒	2498	772.3821	771.3748	771.4490	-0.0742	1	2	1.6e+03	1	AEKSLPK
☒	8792	966.9597	5795.7145	5796.7795	-1.0650	1	2	1.1e+02	1	IVANRLTSPSSALVNASVAFECWINFGTDVAYLWDFGDGTVSLGSSSSSHVYSR
☒	8704	1038.2690	5186.3086	5186.7118	-0.4032	1	1	1.4e+02	1	LMNPTASIIMTLAMAMKMGLAPLHFWVPEVTQGISLSSGLILLTWQK + 3 Oxidation (M)
☒	8834	836.2382	5846.6165	5845.9171	0.6994	1	1	96	1	LPAQPVTHFDDALAEIVQNMYETMYHFHGIGLAAPQVNIQQRLIVMDVPQR + 2 Oxidation (M)
☒	2329	660.7296	659.7223	660.3555	-0.6332	0	1	1.1e+02	1	GTGASLR
☒	2469	756.0067	754.9994	754.4589	0.5405	0	1	4.5e+02	1	IVIGEPK
☒	2753	967.9484	966.9411	966.5862	0.3549	1	1	1.3e+03	1	GLSLKQPPK
☒	8761	957.6240	5739.7003	5740.5300	-0.8297	1	1	1.2e+02	1	MITTMSLLLPGVAVNYYGDEIGMSDTYISWEDTQDPQGCGAGKENYQTMSR + 3 Oxidation (M)
☒	4180	750.1353	1498.2560	1497.7173	0.5387	1	1	1.2e+03	1	DMAKLEFAAPDFK + Oxidation (M)
☒	7638	1273.3013	3816.8821	3818.0691	-1.1870	1	1	4.4e+02	1	GSLGGFGGAGVGGITAVTVNQSLLNPLKLEVDPNIQAVR
☒	8815	967.1259	5796.7117	5797.7954	-1.0836	1	1	1.1e+02	1	IESAICESVLADASETMLANVTSVRQEYAVPVGPVFPPGMNWTDLITNYSPSR + Oxidation (M)
☒	8824	967.1279	5796.7237	5795.8374	0.8863	0	1	1.1e+02	1	NIIGSWPFVFVCLSLNYFGQGVWILNNPTYRPADGGVLNPFYEMIPSDFR + Oxidation (M)
☒	2385	703.5911	702.5838	701.4436	1.1403	1	1	4.7e+02	1	SLIGKGK
☒	8802	966.9606	5795.7199	5796.7795	-1.0596	1	1	1.2e+02	1	IVANRLTSPSSALVNASVAFECWINFGTDVAYLWDFGDGTVSLGSSSSSHVYSR
☒	8942	1089.4944	6530.9227	6529.9396	0.9831	1	1	67	1	EYDDLFMNKLSTPSSSSSSSSSSSSSTTTTTTSTGSTIPLSPPPSSSSSSNNNNSLSPIMTFK + Oxidation (M)
☒	8838	1170.7275	5848.6011	5849.3109	-0.7098	1	1	1e+02	1	EAVIAGIFVLGLALNALIGVALAYTKSMEFGMSLLSLLPLTLVLTANVFATSLVIR + 2 Oxidation (M)
☒	8813	1160.3494	5796.7106	5797.7099	-0.9992	1	1	1.2e+02	1	LRAGPQAVLASGSGFCDNMLADLGLIGTIGEDDEVPVEPESDSGDEEEEGPIVLGR + Oxidation (M)
☒	2405	711.0466	710.0393	710.3269	-0.2876	0	1	94	1	TAMTSGK + Oxidation (M)
☒	8804	828.9673	5795.7202	5794.7652	0.9550	0	1	1.2e+02	1	GYEGAAVGAALGAGITGYNSSSAGATLGVGLAAGLVGMAADAMVEDVNYTMITDVQIAER + 3 Oxidation (M)
☒	8755	1148.9448	5739.6876	5739.8309	-0.1432	0	1	1.3e+02	1	MNPLILSMILTTLAMGTMATMLSSNWLLAWMGLEMNMLAMIPILTMNPNPR + 3 Oxidation (M)
☒	8812	967.1257	5796.7105	5796.7795	-0.0690	1	1	1.2e+02	1	IVANRLTSPSSALVNASVAFECWINFGTDVAYLWDFGDGTVSLGSSSSSHVYSR
☒	8814	1160.3496	5796.7116	5795.9841	0.7275	1	1	1.2e+02	1	EQGVRFEVAHLSNSSATMARPDLTFDLVRPGIAVYGLSPVPALGDMGLVPAMTVK + Oxidation (M)
☒	2364	684.2136	683.2063	684.3555	-1.1492	0	1	91	1	GPTAPSR
☒	8682	1008.2855	5036.3911	5035.4439	0.9472	1	1	2e+02	1	FSDGEVQINLEESVRGCDVYVVQSTCAPVNQHIMELLIMIDALK + Oxidation (M)
☒	8800	966.9603	5795.7181	5794.5875	1.1307	1	1	1.3e+02	1	SMFSLNEGEIIDSFQEFLSDFDTLTYCSYLCELIDISMAEGESNRDLFK + Oxidation (M)
☒	8789	966.9595	5795.7133	5795.8410	-0.1277	1	1	1.3e+02	1	TVYPEIPFSGDFDLNALQHTIKDSEDLLLAQEVLSETTPSGLSNIEYWSWK
☒	2278	632.8872	631.8799	632.2878	-0.4079	0	1	39	1	DAAGSGR
☒	8697	1274.3680	5093.4429	5094.5504	-1.1075	1	1	2e+02	1	ELEVQHPAAKMIVMASHMQEQEVGDGTNFVLVFAGALLELAEELLR + 2 Oxidation (M)
☒	8933	925.8482	6473.8865	6474.0137	-0.1273	1	1	73	1	MKFGVIVFPGSNCDQDVHYALGSVLGQNVDYLWHGDTSVSGYDCLILPGGFSYGDYLR + Oxidation (M)
☒	8787	828.9660	5795.7111	5794.5875	1.1236	1	1	1.3e+02	1	SMFSLNEGEIIDSFQEFLSDFDTLTYCSYLCELIDISMAEGESNRDLFK + Oxidation (M)
☒	2686	923.4105	922.4032	923.4456	-1.0424	0	1	2.4e+03	1	QEMIMIK + 2 Oxidation (M)
☒	2406	711.2962	710.2889	709.4123	0.8767	1	1	1.7e+02	1	GPPKSPK
☒	8791	966.9596	5795.7139	5796.7795	-1.0656	1	1	1.3e+02	1	IVANRLTSPSSALVNASVAFECWINFGTDVAYLWDFGDGTVSLGSSSSSHVYSR
☒	8929	1285.2229	6421.0781	6422.0854	-1.0073	1	1	86	1	EVLELPNLIEIQSNSYQWFLDEGLREMFDDIMPIDDFAGNLSLEFVDYQLLEPK + 2 Oxidation (M)
☒	8943	933.9966	6530.9253	6531.1469	-0.2217	0	1	76	1	VLMMSTNNILHPANGHPIIVPSQDMVLGLYYLSIMNQNEPGEGMAFSDIGELHHALENK + 2 Oxidation (M)
☒	8823	967.1274	5796.7207	5795.8857	0.8350	1	1	1.3e+02	1	KPIHMYINSPGGSVTAGLAIYDTIQMISAPVSTWVIGQASSMGSLLLCAGEKGMR + 2 Oxidation (M)
☒	8939	933.8518	6529.9117	6529.4271	0.4845	1	1	75	1	NITELMELFSNPETIKTLETGDLMTGIGVTVVLGMGITVVALIFLMYIIGGMAAIMAEKPK + 2 Oxidation (M)
☒	8932	1079.8202	6472.8775	6472.2115	0.6661	1	1	83	1	KEEVGGAIYVFMNQAGTSFPAHPSLLLHGPSGSAFGLSVASIGDINQDGFQDIAVGAPFEGLGK
☒	8903	1213.5790	6062.8586	6061.9970	0.8616	1	1	1.1e+02	1	MTSSTDSVPVITFADSPFRLHQPFPPAGDQPAAIELLTEGIGDGLMYQTLLGVTGSGK + 2 Oxidation (M)
☒	8816	1160.3497	5796.7121	5797.7099	-0.9977	1	0	1.4e+02	1	LRAGPQAVLASGSGFCDNMLADLGLIGTIGEDDEVPVEPESDSGDEEEEGPIVLGR + Oxidation (M)
☒	8940	933.8531	6529.9208	6528.9117	1.0091	1	0	79	1	STPQSKEPTLTFPGLPKPSFPMGMPGMMQGFPFFNPMMTAYGCFPGAFPMSQGFMGAQR + 5 Oxidation (M)
☒	8195	1091.0050	4359.9909	4359.0835	0.9074	1	0	3.5e+02	1	DWALGSKGEWVTMGVPSNGEYGIPAGIVFGFPVTTENGEYK
☒	8767	824.1140	5761.7471	5762.8307	-1.0836	1	0	1.6e+02	1	NVSPGAPQRPPSTGSASGMLSTTDLTVPNGAGNSPVGNGFVNSRASPNLIGNTGANSLGK + Oxidation (M)
☒	8818	1160.3500	5796.7136	5796.7795	-0.0659	1	0	1.4e+02	1	IVANRLTSPSSALVNASVAFECWINFGTDVAYLWDFGDGTVSLGSSSSSHVYSR
☒	8706	1366.3610	5461.4149	5460.4764	0.9385	0	0	1.6e+02	1	AAMADPTCAGPATFTAAGAFSQPDLMAFSLPEEEPIWGFDTIAPSMASWQGK + Oxidation (M)
☒	8833	1167.7638	5833.7826	5834.6694	-0.8868	0	0	1.4e+02	1	LDMPITHEDYVAEEAEIQVPEESVPSGDGGFPEDPFIHSTPAPASLTEEQQQR + Oxidation (M)
☒	2287	636.3972	635.3899	636.2537	-0.8638	0	0	26	1	GGDGMGK + Oxidation (M)
☒	8167	869.2001	4340.9641	4340.9859	-0.0218	1	0	3.6e+02	1	NELPQVHQMPGMPPYIPVSYLDRMDLAYAAADMMLCR + 3 Oxidation (M)
☒	8941	817.3723	6530.9202	6530.0592	0.8610	1	0	85	1	NWPADLPQGVIHADLFPDNVFFLGDRLSGFIDFYFACTDTLAYDVAVCLNAWCFEK
☒	2362	684.2010	683.1937	683.4218	-0.2281	0	0	1.1e+02	1	IIVPVGS
☒	2296	636.3977	635.3904	636.3231	-0.9327	0	0	27	1	APAPANP
☒	2558	808.3616	807.3543	806.3666	0.9877	0	0	2e+03	1	VMMGPEK + Oxidation (M)
☒	4727	839.5611	1677.1076	1677.8209	-0.7133	0	0	1.6e+03	1	LESQSPNSAEYNVIK
☒	2271	619.8668	618.8595	618.3085	0.5510	0	0	26	1	ATGGASR
☒	1	*350.2956*	*349.2883*							
☒	2	*350.2959*	*349.2886*							
☒	3	*350.2961*	*349.2888*							
☒	4	*350.2962*	*349.2889*							
☒	5	*350.2963*	*349.2890*							
☒	6	*350.2963*	*349.2890*							
☒	7	*350.2964*	*349.2891*							
☒	8	*350.2964*	*349.2891*							
☒	9	*350.2964*	*349.2891*							
☒	10	*350.2964*	*349.2891*							
☒	11	*350.2964*	*349.2891*							
☒	12	*350.2965*	*349.2892*							
☒	13	*350.2965*	*349.2892*							
☒	14	*350.2965*	*349.2892*							
☒	15	*350.2965*	*349.2892*							
☒	16	*350.2966*	*349.2893*							
☒	17	*350.2966*	*349.2893*							
☒	18	*350.2966*	*349.2893*							
☒	19	*350.2967*	*349.2894*							
☒	20	*350.2967*	*349.2894*							
☒	21	*350.2967*	*349.2894*							
☒	22	*350.2968*	*349.2895*							
☒	23	*350.2969*	*349.2896*							
☒	24	*350.3050*	*349.2977*							
☒	25	*352.3202*	*351.3129*							

26	353.1558	352.1485
27	353.1558	352.1485
28	353.1560	352.1487
29	353.1560	352.1487
30	355.0677	354.0604
31	355.0681	354.0608
32	355.0681	354.0608
33	355.0681	354.0608
34	355.0682	354.0609
35	355.0682	354.0609
36	355.0682	354.0609
37	355.0683	354.0610
38	355.0683	354.0610
39	355.0683	354.0610
40	355.0683	354.0610
41	355.0683	354.0610
42	355.0683	354.0610
43	355.0683	354.0610
44	355.0683	354.0610
45	355.0683	354.0610
46	355.0683	354.0610
47	355.0684	354.0611
48	355.0684	354.0611
49	355.0684	354.0611
50	355.0684	354.0611
51	355.0684	354.0611
52	355.0685	354.0612
53	355.0685	354.0612
54	355.0685	354.0612
55	355.0685	354.0612
56	355.0685	354.0612
57	355.0685	354.0612
58	355.0685	354.0612
59	355.0685	354.0612
60	355.0685	354.0612
61	355.0685	354.0612
62	355.0685	354.0612
63	355.0685	354.0612
64	355.0685	354.0612
65	355.0685	354.0612
66	355.0685	354.0612
67	355.0685	354.0612
68	355.0686	354.0613
69	355.0686	354.0613
70	355.0686	354.0613
71	355.0686	354.0613
72	355.0686	354.0613
73	355.0686	354.0613
74	355.0686	354.0613
75	355.0686	354.0613
76	355.0686	354.0613
77	355.0686	354.0613
78	355.0686	354.0613
79	355.0686	354.0613
80	355.0686	354.0613
81	355.0686	354.0613
82	355.0686	354.0613
83	355.0686	354.0613
84	355.0686	354.0613
85	355.0686	354.0613
86	355.0686	354.0613
87	355.0686	354.0613
88	355.0687	354.0614
89	355.0687	354.0614
90	355.0687	354.0614
91	355.0687	354.0614
92	355.0687	354.0614
93	355.0687	354.0614
94	355.0687	354.0614
95	355.0687	354.0614
96	355.0687	354.0614
97	355.0687	354.0614
98	355.0687	354.0614
99	355.0687	354.0614
100	355.0687	354.0614
101	355.0687	354.0614
102	355.0687	354.0614
103	355.0687	354.0614
104	355.0687	354.0614
105	355.0687	354.0614
106	355.0687	354.0614
107	355.0687	354.0614
108	355.0687	354.0614
109	355.0687	354.0614
110	355.0687	354.0614
111	355.0687	354.0614
112	355.0687	354.0614
113	355.0687	354.0614
114	355.0687	354.0614
115	355.0687	354.0614
116	355.0687	354.0614
117	355.0687	354.0614
118	355.0687	354.0614
119	355.0687	354.0614
120	355.0688	354.0615
121	355.0688	354.0615
122	355.0688	354.0615
123	355.0688	354.0615
124	355.0688	354.0615
125	355.0688	354.0615
126	355.0688	354.0615

127	355.0688	354.0615
128	355.0688	354.0615
129	355.0688	354.0615
130	355.0688	354.0615
131	355.0688	354.0615
132	355.0688	354.0615
133	355.0688	354.0615
134	355.0688	354.0615
135	355.0688	354.0615
136	355.0688	354.0615
137	355.0688	354.0615
138	355.0688	354.0615
139	355.0688	354.0615
140	355.0688	354.0615
141	355.0688	354.0615
142	355.0688	354.0615
143	355.0688	354.0615
144	355.0688	354.0615
145	355.0688	354.0615
146	355.0688	354.0615
147	355.0688	354.0615
148	355.0688	354.0615
149	355.0688	354.0615
150	355.0688	354.0615
151	355.0688	354.0615
152	355.0688	354.0615
153	355.0688	354.0615
154	355.0688	354.0615
155	355.0688	354.0615
156	355.0688	354.0615
157	355.0688	354.0615
158	355.0688	354.0615
159	355.0688	354.0615
160	355.0688	354.0615
161	355.0688	354.0615
162	355.0688	354.0615
163	355.0688	354.0615
164	355.0688	354.0615
165	355.0688	354.0615
166	355.0688	354.0615
167	355.0688	354.0615
168	355.0688	354.0615
169	355.0688	354.0615
170	355.0688	354.0615
171	355.0688	354.0615
172	355.0688	354.0615
173	355.0688	354.0615
174	355.0688	354.0615
175	355.0688	354.0615
176	355.0688	354.0615
177	355.0688	354.0615
178	355.0688	354.0615
179	355.0688	354.0615
180	355.0688	354.0615
181	355.0689	354.0616
182	355.0689	354.0616
183	355.0689	354.0616
184	355.0689	354.0616
185	355.0689	354.0616
186	355.0689	354.0616
187	355.0689	354.0616
188	355.0689	354.0616
189	355.0689	354.0616
190	355.0689	354.0616
191	355.0689	354.0616
192	355.0689	354.0616
193	355.0689	354.0616
194	355.0689	354.0616
195	355.0689	354.0616
196	355.0689	354.0616
197	355.0689	354.0616
198	355.0689	354.0616
199	355.0689	354.0616
200	355.0689	354.0616
201	355.0689	354.0616
202	355.0689	354.0616
203	355.0689	354.0616
204	355.0689	354.0616
205	355.0689	354.0616
206	355.0689	354.0616
207	355.0689	354.0616
208	355.0689	354.0616
209	355.0689	354.0616
210	355.0689	354.0616
211	355.0689	354.0616
212	355.0689	354.0616
213	355.0689	354.0616
214	355.0689	354.0616
215	355.0689	354.0616
216	355.0689	354.0616
217	355.0690	354.0617
218	355.0690	354.0617
219	355.0690	354.0617
220	355.0690	354.0617
221	355.0690	354.0617
222	355.0690	354.0617
223	355.0690	354.0617
224	355.0690	354.0617
225	355.0690	354.0617
226	355.0690	354.0617
227	355.0690	354.0617

228	355.0690	354.0617
229	355.0690	354.0617
230	355.0690	354.0617
231	355.0690	354.0617
232	355.0690	354.0617
233	355.0690	354.0617
234	355.0690	354.0617
235	355.0690	354.0617
236	355.0690	354.0617
237	355.0690	354.0617
238	355.0690	354.0617
239	355.0690	354.0617
240	355.0690	354.0617
241	355.0690	354.0617
242	355.0690	354.0617
243	355.0690	354.0617
244	355.0690	354.0617
245	355.0690	354.0617
246	355.0690	354.0617
247	355.0690	354.0617
248	355.0690	354.0617
249	355.0690	354.0617
250	355.0690	354.0617
251	355.0691	354.0618
252	355.0691	354.0618
253	355.0691	354.0618
254	355.0691	354.0618
255	355.0691	354.0618
256	355.0691	354.0618
257	355.0691	354.0618
258	355.0691	354.0618
259	355.0691	354.0618
260	355.0691	354.0618
261	355.0691	354.0618
262	355.0691	354.0618
263	355.0691	354.0618
264	355.0691	354.0618
265	355.0691	354.0618
266	355.0692	354.0619
267	355.0692	354.0619
268	355.0692	354.0619
269	355.0692	354.0619
270	355.0692	354.0619
271	355.0692	354.0619
272	355.0692	354.0619
273	355.0693	354.0620
274	355.0693	354.0620
275	355.0694	354.0621
276	361.2205	360.2132
277	361.2209	360.2136
278	361.2209	360.2136
279	361.2209	360.2136
280	361.2209	360.2136
281	361.2209	360.2136
282	361.2210	360.2137
283	361.2210	360.2137
284	361.2210	360.2137
285	361.2210	360.2137
286	361.2210	360.2137
287	361.2210	360.2137
288	361.2210	360.2137
289	361.2211	360.2138
290	361.2211	360.2138
291	361.2211	360.2138
292	361.2211	360.2138
293	361.2211	360.2138
294	361.2211	360.2138
295	361.2211	360.2138
296	361.2211	360.2138
297	361.2211	360.2138
298	361.2212	360.2139
299	361.2212	360.2139
300	361.2212	360.2139
301	361.2212	360.2139
302	361.2212	360.2139
303	361.2212	360.2139
304	361.2213	360.2140
305	361.2213	360.2140
306	361.2213	360.2140
307	361.2213	360.2140
308	361.2213	360.2140
309	361.2213	360.2140
310	361.2213	360.2140
311	361.2214	360.2141
312	361.2214	360.2141
313	361.2214	360.2141
314	361.2214	360.2141
315	361.2216	360.2143
316	361.2216	360.2143
317	361.2217	360.2144
318	361.2218	360.2145
319	361.2219	360.2146
320	362.2204	361.2131
321	363.1281	362.1208
322	363.1282	362.1209
323	363.1283	362.1210
324	363.1284	362.1211
325	363.1284	362.1211
326	363.1286	362.1213

327	363.3094	362.3021
328	364.1954	363.1881
329	364.1964	363.1891
330	364.2832	363.2759
331	364.2838	363.2765
332	364.2840	363.2767
333	366.1392	365.1319
334	366.1394	365.1321
335	366.1395	365.1322
336	366.1395	365.1322
337	366.1397	365.1324
338	366.1398	365.1325
339	366.3199	365.3126
340	366.3200	365.3127
341	366.3202	365.3129
342	366.3202	365.3129
343	366.3202	365.3129
344	366.3203	365.3130
345	366.3203	365.3130
346	366.3203	365.3130
347	366.3203	365.3130
348	366.3203	365.3130
349	366.3203	365.3130
350	366.3203	365.3130
351	366.3203	365.3130
352	366.3203	365.3130
353	366.3203	365.3130
354	366.3203	365.3130
355	366.3203	365.3130
356	366.3203	365.3130
357	366.3203	365.3130
358	366.3204	365.3131
359	366.3204	365.3131
360	366.3204	365.3131
361	366.3204	365.3131
362	366.3204	365.3131
363	366.3205	365.3132
364	366.3205	365.3132
365	366.3205	365.3132
366	366.3205	365.3132
367	366.3206	365.3133
368	366.3206	365.3133
369	366.3206	365.3133
370	366.3207	365.3134
371	366.3207	365.3134
372	366.3207	365.3134
373	366.3208	365.3135
374	366.3208	365.3135
375	366.3209	365.3136
376	367.2681	366.2608
377	367.2682	366.2609
378	367.2682	366.2609
379	367.2683	366.2610
380	368.2263	367.2190
381	368.2274	367.2201
382	368.3149	367.3076
383	371.0992	370.0919
384	371.0994	370.0921
385	371.0994	370.0921
386	371.0994	370.0921
387	371.0994	370.0921
388	371.0994	370.0921
389	371.0995	370.0922
390	371.0995	370.0922
391	371.0995	370.0922
392	371.0995	370.0922
393	371.0995	370.0922
394	371.0995	370.0922
395	371.0995	370.0922
396	371.0996	370.0923
397	371.0996	370.0923
398	371.0996	370.0923
399	371.0996	370.0923
400	371.0996	370.0923
401	371.0996	370.0923
402	371.0996	370.0923
403	371.0996	370.0923
404	371.0996	370.0923
405	371.0996	370.0923
406	371.0996	370.0923
407	371.0996	370.0923
408	371.0996	370.0923
409	371.0996	370.0923
410	371.0997	370.0924
411	371.0997	370.0924
412	371.0997	370.0924
413	371.0997	370.0924
414	371.0997	370.0924
415	371.0997	370.0924
416	371.0997	370.0924
417	371.0997	370.0924
418	371.0997	370.0924
419	371.0997	370.0924
420	371.0997	370.0924
421	371.0997	370.0924
422	371.0997	370.0924
423	371.0997	370.0924
424	371.0997	370.0924
425	371.0997	370.0924
426	371.0997	370.0924
427	371.0997	370.0924

428	371.0997	370.0924
429	371.0997	370.0924
430	371.0998	370.0925
431	371.0998	370.0925
432	371.0998	370.0925
433	371.0998	370.0925
434	371.0998	370.0925
435	371.0998	370.0925
436	371.0998	370.0925
437	371.0998	370.0925
438	371.0998	370.0925
439	371.0998	370.0925
440	371.0998	370.0925
441	371.0998	370.0925
442	371.0998	370.0925
443	371.0998	370.0925
444	371.0998	370.0925
445	371.0998	370.0925
446	371.0998	370.0925
447	371.0998	370.0925
448	371.0998	370.0925
449	371.0998	370.0925
450	371.0998	370.0925
451	371.0998	370.0925
452	371.0998	370.0925
453	371.0998	370.0925
454	371.0998	370.0925
455	371.0998	370.0925
456	371.0999	370.0926
457	371.0999	370.0926
458	371.0999	370.0926
459	371.0999	370.0926
460	371.0999	370.0926
461	371.0999	370.0926
462	371.0999	370.0926
463	371.0999	370.0926
464	371.0999	370.0926
465	371.0999	370.0926
466	371.0999	370.0926
467	371.0999	370.0926
468	371.0999	370.0926
469	371.0999	370.0926
470	371.0999	370.0926
471	371.0999	370.0926
472	371.0999	370.0926
473	371.0999	370.0926
474	371.0999	370.0926
475	371.0999	370.0926
476	371.0999	370.0926
477	371.0999	370.0926
478	371.0999	370.0926
479	371.0999	370.0926
480	371.0999	370.0926
481	371.0999	370.0926
482	371.0999	370.0926
483	371.0999	370.0926
484	371.0999	370.0926
485	371.0999	370.0926
486	371.0999	370.0926
487	371.0999	370.0926
488	371.0999	370.0926
489	371.0999	370.0926
490	371.0999	370.0926
491	371.0999	370.0926
492	371.0999	370.0926
493	371.0999	370.0926
494	371.0999	370.0926
495	371.0999	370.0926
496	371.0999	370.0926
497	371.0999	370.0926
498	371.0999	370.0926
499	371.1000	370.0927
500	371.1000	370.0927
501	371.1000	370.0927
502	371.1000	370.0927
503	371.1000	370.0927
504	371.1000	370.0927
505	371.1000	370.0927
506	371.1000	370.0927
507	371.1000	370.0927
508	371.1000	370.0927
509	371.1000	370.0927
510	371.1000	370.0927
511	371.1000	370.0927
512	371.1000	370.0927
513	371.1000	370.0927
514	371.1000	370.0927
515	371.1000	370.0927
516	371.1000	370.0927
517	371.1000	370.0927
518	371.1000	370.0927
519	371.1000	370.0927
520	371.1000	370.0927
521	371.1000	370.0927
522	371.1000	370.0927
523	371.1000	370.0927
524	371.1000	370.0927
525	371.1000	370.0927
526	371.1000	370.0927
527	371.1000	370.0927
528	371.1000	370.0927

529	*371.1000*	*370.0927*
530	*371.1000*	*370.0927*
531	*371.1000*	*370.0927*
532	*371.1000*	*370.0927*
533	*371.1000*	*370.0927*
534	*371.1000*	*370.0927*
535	*371.1000*	*370.0927*
536	*371.1000*	*370.0927*
537	*371.1000*	*370.0927*
538	*371.1000*	*370.0927*
539	*371.1000*	*370.0927*
540	*371.1000*	*370.0927*
541	*371.1000*	*370.0927*
542	*371.1000*	*370.0927*
543	*371.1000*	*370.0927*
544	*371.1000*	*370.0927*
545	*371.1001*	*370.0928*
546	*371.1001*	*370.0928*
547	*371.1001*	*370.0928*
548	*371.1001*	*370.0928*
549	*371.1001*	*370.0928*
550	*371.1001*	*370.0928*
551	*371.1001*	*370.0928*
552	*371.1001*	*370.0928*
553	*371.1001*	*370.0928*
554	*371.1001*	*370.0928*
555	*371.1001*	*370.0928*
556	*371.1001*	*370.0928*
557	*371.1001*	*370.0928*
558	*371.1001*	*370.0928*
559	*371.1001*	*370.0928*
560	*371.1001*	*370.0928*
561	*371.1001*	*370.0928*
562	*371.1001*	*370.0928*
563	*371.1001*	*370.0928*
564	*371.1001*	*370.0928*
565	*371.1001*	*370.0928*
566	*371.1001*	*370.0928*
567	*371.1001*	*370.0928*
568	*371.1001*	*370.0928*
569	*371.1001*	*370.0928*
570	*371.1001*	*370.0928*
571	*371.1001*	*370.0928*
572	*371.1001*	*370.0928*
573	*371.1001*	*370.0928*
574	*371.1001*	*370.0928*
575	*371.1001*	*370.0928*
576	*371.1001*	*370.0928*
577	*371.1001*	*370.0928*
578	*371.1001*	*370.0928*
579	*371.1001*	*370.0928*
580	*371.1002*	*370.0929*
581	*371.1002*	*370.0929*
582	*371.1002*	*370.0929*
583	*371.1002*	*370.0929*
584	*371.1002*	*370.0929*
585	*371.1002*	*370.0929*
586	*371.1002*	*370.0929*
587	*371.1002*	*370.0929*
588	*371.1002*	*370.0929*
589	*371.1002*	*370.0929*
590	*371.1002*	*370.0929*
591	*371.1002*	*370.0929*
592	*371.1002*	*370.0929*
593	*371.1002*	*370.0929*
594	*371.1002*	*370.0929*
595	*371.1002*	*370.0929*
596	*371.1002*	*370.0929*
597	*371.1002*	*370.0929*
598	*371.1002*	*370.0929*
599	*371.1002*	*370.0929*
600	*371.1003*	*370.0930*
601	*371.1003*	*370.0930*
602	*371.1003*	*370.0930*
603	*371.1003*	*370.0930*
604	*371.1003*	*370.0930*
605	*371.1003*	*370.0930*
606	*371.1003*	*370.0930*
607	*371.1003*	*370.0930*
608	*371.1003*	*370.0930*
609	*371.1003*	*370.0930*
610	*371.1003*	*370.0930*
611	*371.1003*	*370.0930*
612	*371.1003*	*370.0930*
613	*371.1003*	*370.0930*
614	*371.1003*	*370.0930*
615	*371.1003*	*370.0930*
616	*371.1003*	*370.0930*
617	*371.1003*	*370.0930*
618	*371.1003*	*370.0930*
619	*371.1003*	*370.0930*
620	*371.1003*	*370.0930*
621	*371.1003*	*370.0930*
622	*371.1004*	*370.0931*
623	*371.1004*	*370.0931*
624	*371.1004*	*370.0931*
625	*371.1004*	*370.0931*
626	*371.1004*	*370.0931*
627	*371.1005*	*370.0932*
628	*371.1005*	*370.0932*
629	*371.1005*	*370.0932*

630	371.1005	370.0932
631	371.1005	370.0932
632	371.1005	370.0932
633	371.1005	370.0932
634	371.1006	370.0933
635	371.1006	370.0933
636	371.1006	370.0933
637	371.1006	370.0933
638	371.1009	370.0936
639	372.0999	371.0926
640	372.1001	371.0928
641	372.1002	371.0929
642	372.1002	371.0929
643	373.9344	372.9271
644	374.0962	373.0889
645	374.0966	373.0893
646	374.0966	373.0893
647	374.0967	373.0894
648	374.0967	373.0894
649	374.0968	373.0895
650	374.0968	373.0895
651	374.0968	373.0895
652	374.0968	373.0895
653	374.0969	373.0896
654	374.0969	373.0896
655	374.0969	373.0896
656	374.0969	373.0896
657	374.0969	373.0896
658	374.0969	373.0896
659	374.0970	373.0897
660	374.0970	373.0897
661	374.0970	373.0897
662	374.0970	373.0897
663	374.0970	373.0897
664	374.0970	373.0897
665	374.0970	373.0897
666	374.0970	373.0897
667	374.0970	373.0897
668	374.0970	373.0897
669	374.0970	373.0897
670	374.0970	373.0897
671	374.0970	373.0897
672	374.0970	373.0897
673	374.0971	373.0898
674	374.0971	373.0898
675	374.0971	373.0898
676	374.0971	373.0898
677	374.0971	373.0898
678	374.0971	373.0898
679	374.0971	373.0898
680	374.0971	373.0898
681	374.0971	373.0898
682	374.0971	373.0898
683	374.0971	373.0898
684	374.0972	373.0899
685	374.0972	373.0899
686	374.0972	373.0899
687	374.0972	373.0899
688	374.0972	373.0899
689	374.0973	373.0900
690	374.0973	373.0900
691	374.0973	373.0900
692	374.0973	373.0900
693	374.0973	373.0900
694	374.0973	373.0900
695	374.0974	373.0901
696	374.0975	373.0902
697	374.0975	373.0902
698	374.3260	373.3187
699	375.1998	374.1925
700	375.2000	374.1927
701	375.2000	374.1927
702	375.2000	374.1927
703	375.2220	374.2147
704	378.2479	377.2406
705	380.3354	379.3281
706	380.3359	379.3286
707	380.3359	379.3286
708	380.3361	379.3288
709	380.3361	379.3288
710	380.3362	379.3289
711	380.3362	379.3289
712	380.3362	379.3289
713	380.3362	379.3289
714	380.3363	379.3290
715	380.3363	379.3290
716	380.3363	379.3290
717	380.3363	379.3290
718	380.3364	379.3291
719	381.1385	380.1312
720	381.1387	380.1314
721	381.1387	380.1314
722	381.1388	380.1315
723	381.1388	380.1315
724	381.1389	380.1316
725	381.1389	380.1316
726	381.1389	380.1316
727	381.1390	380.1317
728	381.1390	380.1317
729	381.1391	380.1318
730	381.1391	380.1318

731	*381.1392*	*380.1319*
732	*381.1394*	*380.1321*
733	*382.1425*	*381.1352*
734	*382.1431*	*381.1358*
735	*384.1492*	*383.1419*
736	*384.1503*	*383.1430*
737	*384.2948*	*383.2875*
738	*384.2949*	*383.2876*
739	*384.3100*	*383.3027*
740	*384.3103*	*383.3030*
741	*385.2629*	*384.2556*
742	*385.2630*	*384.2557*
743	*385.2631*	*384.2558*
744	*385.2631*	*384.2558*
745	*386.3254*	*385.3181*
746	*386.3256*	*385.3183*
747	*386.3258*	*385.3185*
748	*387.1691*	*386.1618*
749	*387.1692*	*386.1619*
750	*387.1693*	*386.1620*
751	*387.1694*	*386.1621*
752	*387.1790*	*386.1717*
753	*387.1791*	*386.1718*
754	*387.2368*	*386.2295*
755	*388.3413*	*387.3340*
756	*388.3413*	*387.3340*
757	*388.3414*	*387.3341*
758	*391.2827*	*390.2754*
759	*391.2831*	*390.2758*
760	*391.2832*	*390.2759*
761	*391.2833*	*390.2760*
762	*392.2260*	*391.2187*
763	*392.2261*	*391.2188*
764	*392.2262*	*391.2189*
765	*392.2264*	*391.2191*
766	*392.2265*	*391.2192*
767	*392.2265*	*391.2192*
768	*392.2266*	*391.2193*
769	*392.2267*	*391.2194*
770	*392.2267*	*391.2194*
771	*392.2268*	*391.2195*
772	*392.2268*	*391.2195*
773	*392.2269*	*391.2196*
774	*392.2271*	*391.2198*
775	*394.1595*	*393.1522*
776	*394.1604*	*393.1531*
777	*394.1605*	*393.1532*
778	*394.1606*	*393.1533*
779	*397.1815*	*396.1742*
780	*397.1816*	*396.1743*
781	*397.1817*	*396.1744*
782	*397.1817*	*396.1744*
783	*397.2067*	*396.1994*
784	*397.2068*	*396.1995*
785	*398.1650*	*397.1577*
786	*398.1652*	*397.1579*
787	*398.1652*	*397.1579*
788	*398.1652*	*397.1579*
789	*398.1653*	*397.1580*
790	*398.1654*	*397.1581*
791	*398.1655*	*397.1582*
792	*398.1656*	*397.1583*
793	*399.1489*	*398.1416*
794	*399.1492*	*398.1419*
795	*399.1493*	*398.1420*
796	*399.1496*	*398.1423*
797	*399.1498*	*398.1425*
798	*399.1498*	*398.1425*
799	*401.1483*	*400.1410*
800	*401.2158*	*400.2085*
801	*401.2160*	*400.2087*
802	*402.1143*	*401.1070*
803	*402.1149*	*401.1076*
804	*402.1150*	*401.1077*
805	*402.1150*	*401.1077*
806	*402.1150*	*401.1077*
807	*402.1151*	*401.1078*
808	*402.1152*	*401.1079*
809	*403.1204*	*402.1131*
810	*403.1207*	*402.1134*
811	*403.1207*	*402.1134*
812	*403.1207*	*402.1134*
813	*403.1208*	*402.1135*
814	*403.1208*	*402.1135*
815	*403.1208*	*402.1135*
816	*403.1210*	*402.1137*
817	*404.2052*	*403.1979*
818	*406.2014*	*405.1941*
819	*408.2213*	*407.2140*
820	*410.3458*	*409.3385*
821	*410.3461*	*409.3388*
822	*410.3463*	*409.3390*
823	*410.3464*	*409.3391*
824	*410.3464*	*409.3391*
825	*410.3464*	*409.3391*
826	*410.3464*	*409.3391*
827	*410.3464*	*409.3391*
828	*410.3465*	*409.3392*
829	*410.3466*	*409.3393*
830	*410.3466*	*409.3393*
831	*410.3466*	*409.3393*

832	410.3467	409.3394
833	410.3468	409.3395
834	410.3468	409.3395
835	410.3470	409.3397
836	410.3473	409.3400
837	415.2002	414.1929
838	415.2004	414.1931
839	415.2311	414.2238
840	415.2313	414.2240
841	415.2313	414.2240
842	415.2314	414.2241
843	415.2314	414.2241
844	416.1305	415.1232
845	416.1306	415.1233
846	416.1309	415.1236
847	419.2260	418.2187
848	419.2264	418.2191
849	419.2264	418.2191
850	419.2265	418.2192
851	419.3139	418.3066
852	419.3145	418.3072
853	419.3149	418.3076
854	419.3149	418.3076
855	420.0078	419.0005
856	420.0084	419.0011
857	421.2172	420.2099
858	421.2176	420.2103
859	422.2520	421.2447
860	422.2521	421.2448
861	423.2405	422.2332
862	424.3621	423.3548
863	424.3621	423.3548
864	424.3621	423.3548
865	424.3622	423.3549
866	424.3622	423.3549
867	424.3622	423.3549
868	424.3622	423.3549
869	424.3622	423.3549
870	424.3623	423.3550
871	424.3623	423.3550
872	424.3623	423.3550
873	424.3624	423.3551
874	424.3624	423.3551
875	424.3624	423.3551
876	424.3624	423.3551
877	424.3624	423.3551
878	424.3624	423.3551
879	424.3624	423.3551
880	424.3626	423.3553
881	424.3627	423.3554
882	424.3628	423.3555
883	424.3632	423.3559
884	425.1303	424.1230
885	425.1306	424.1233
886	425.1308	424.1235
887	425.1309	424.1236
888	432.2791	431.2718
889	433.2417	432.2344
890	434.0498	433.0425
891	434.0498	433.0425
892	434.0499	433.0426
893	434.0499	433.0426
894	434.2735	433.2662
895	434.2737	433.2664
896	436.2521	435.2448
897	436.2521	435.2448
898	436.2523	435.2450
899	436.2525	435.2452
900	436.2525	435.2452
901	436.2526	435.2453
902	436.2527	435.2454
903	436.2527	435.2454
904	436.2531	435.2458
905	436.2533	435.2460
906	439.1458	438.1385
907	439.1458	438.1385
908	439.1463	438.1390
909	439.1463	438.1390
910	439.1464	438.1391
911	439.1464	438.1391
912	439.1464	438.1391
913	439.1464	438.1391
914	439.1465	438.1392
915	439.1466	438.1393
916	439.1469	438.1396
917	439.1469	438.1396
918	441.2081	440.2008
919	441.2083	440.2010
920	442.3361	441.3288
921	442.3362	441.3289
922	442.3363	441.3290
923	442.3363	441.3290
924	443.2318	442.2245
925	443.2319	442.2246
926	445.1176	444.1103
927	445.1177	444.1104
928	445.1177	444.1104
929	445.1178	444.1105
930	445.1178	444.1105
931	445.1178	444.1105
932	445.1178	444.1105

933	*445.1178*	*444.1105*
934	*445.1178*	*444.1105*
935	*445.1179*	*444.1106*
936	*445.1179*	*444.1106*
937	*445.1179*	*444.1106*
938	*445.1179*	*444.1106*
939	*445.1180*	*444.1107*
940	*445.1180*	*444.1107*
941	*445.1180*	*444.1107*
942	*445.1180*	*444.1107*
943	*445.1180*	*444.1107*
944	*445.1180*	*444.1107*
945	*445.1180*	*444.1107*
946	*445.1180*	*444.1107*
947	*445.1181*	*444.1108*
948	*445.1181*	*444.1108*
949	*445.1181*	*444.1108*
950	*445.1181*	*444.1108*
951	*445.1181*	*444.1108*
952	*445.1181*	*444.1108*
953	*445.1181*	*444.1108*
954	*445.1181*	*444.1108*
955	*445.1181*	*444.1108*
956	*445.1182*	*444.1109*
957	*445.1182*	*444.1109*
958	*445.1182*	*444.1109*
959	*445.1182*	*444.1109*
960	*445.1182*	*444.1109*
961	*445.1182*	*444.1109*
962	*445.1182*	*444.1109*
963	*445.1182*	*444.1109*
964	*445.1182*	*444.1109*
965	*445.1183*	*444.1110*
966	*445.1183*	*444.1110*
967	*445.1183*	*444.1110*
968	*445.1183*	*444.1110*
969	*445.1183*	*444.1110*
970	*445.1183*	*444.1110*
971	*445.1183*	*444.1110*
972	*445.1183*	*444.1110*
973	*445.1183*	*444.1110*
974	*445.1183*	*444.1110*
975	*445.1183*	*444.1110*
976	*445.1183*	*444.1110*
977	*445.1183*	*444.1110*
978	*445.1183*	*444.1110*
979	*445.1183*	*444.1110*
980	*445.1183*	*444.1110*
981	*445.1183*	*444.1110*
982	*445.1183*	*444.1110*
983	*445.1183*	*444.1110*
984	*445.1183*	*444.1110*
985	*445.1183*	*444.1110*
986	*445.1183*	*444.1110*
987	*445.1184*	*444.1111*
988	*445.1184*	*444.1111*
989	*445.1184*	*444.1111*
990	*445.1184*	*444.1111*
991	*445.1184*	*444.1111*
992	*445.1184*	*444.1111*
993	*445.1184*	*444.1111*
994	*445.1184*	*444.1111*
995	*445.1184*	*444.1111*
996	*445.1184*	*444.1111*
997	*445.1184*	*444.1111*
998	*445.1184*	*444.1111*
999	*445.1184*	*444.1111*
1000	*445.1184*	*444.1111*
1001	*445.1184*	*444.1111*
1002	*445.1184*	*444.1111*
1003	*445.1184*	*444.1111*
1004	*445.1184*	*444.1111*
1005	*445.1184*	*444.1111*
1006	*445.1185*	*444.1112*
1007	*445.1185*	*444.1112*
1008	*445.1185*	*444.1112*
1009	*445.1185*	*444.1112*
1010	*445.1185*	*444.1112*
1011	*445.1185*	*444.1112*
1012	*445.1185*	*444.1112*
1013	*445.1185*	*444.1112*
1014	*445.1185*	*444.1112*
1015	*445.1185*	*444.1112*
1016	*445.1185*	*444.1112*
1017	*445.1185*	*444.1112*
1018	*445.1185*	*444.1112*
1019	*445.1185*	*444.1112*
1020	*445.1185*	*444.1112*
1021	*445.1185*	*444.1112*
1022	*445.1185*	*444.1112*
1023	*445.1185*	*444.1112*
1024	*445.1185*	*444.1112*
1025	*445.1185*	*444.1112*
1026	*445.1185*	*444.1112*
1027	*445.1185*	*444.1112*
1028	*445.1185*	*444.1112*
1029	*445.1185*	*444.1112*
1030	*445.1185*	*444.1112*
1031	*445.1185*	*444.1112*
1032	*445.1185*	*444.1112*

1033	*445.1185*	*444.1112*
1034	*445.1185*	*444.1112*
1035	*445.1185*	*444.1112*
1036	*445.1185*	*444.1112*
1037	*445.1185*	*444.1112*
1038	*445.1186*	*444.1113*
1039	*445.1186*	*444.1113*
1040	*445.1186*	*444.1113*
1041	*445.1186*	*444.1113*
1042	*445.1186*	*444.1113*
1043	*445.1186*	*444.1113*
1044	*445.1186*	*444.1113*
1045	*445.1186*	*444.1113*
1046	*445.1186*	*444.1113*
1047	*445.1186*	*444.1113*
1048	*445.1186*	*444.1113*
1049	*445.1186*	*444.1113*
1050	*445.1186*	*444.1113*
1051	*445.1186*	*444.1113*
1052	*445.1186*	*444.1113*
1053	*445.1186*	*444.1113*
1054	*445.1186*	*444.1113*
1055	*445.1186*	*444.1113*
1056	*445.1186*	*444.1113*
1057	*445.1186*	*444.1113*
1058	*445.1186*	*444.1113*
1059	*445.1186*	*444.1113*
1060	*445.1186*	*444.1113*
1061	*445.1186*	*444.1113*
1062	*445.1186*	*444.1113*
1063	*445.1186*	*444.1113*
1064	*445.1186*	*444.1113*
1065	*445.1186*	*444.1113*
1066	*445.1186*	*444.1113*
1067	*445.1186*	*444.1113*
1068	*445.1186*	*444.1113*
1069	*445.1186*	*444.1113*
1070	*445.1187*	*444.1114*
1071	*445.1187*	*444.1114*
1072	*445.1187*	*444.1114*
1073	*445.1187*	*444.1114*
1074	*445.1187*	*444.1114*
1075	*445.1187*	*444.1114*
1076	*445.1187*	*444.1114*
1077	*445.1187*	*444.1114*
1078	*445.1187*	*444.1114*
1079	*445.1187*	*444.1114*
1080	*445.1187*	*444.1114*
1081	*445.1187*	*444.1114*
1082	*445.1187*	*444.1114*
1083	*445.1187*	*444.1114*
1084	*445.1187*	*444.1114*
1085	*445.1187*	*444.1114*
1086	*445.1187*	*444.1114*
1087	*445.1187*	*444.1114*
1088	*445.1187*	*444.1114*
1089	*445.1187*	*444.1114*
1090	*445.1187*	*444.1114*
1091	*445.1187*	*444.1114*
1092	*445.1187*	*444.1114*
1093	*445.1187*	*444.1114*
1094	*445.1187*	*444.1114*
1095	*445.1187*	*444.1114*
1096	*445.1187*	*444.1114*
1097	*445.1187*	*444.1114*
1098	*445.1187*	*444.1114*
1099	*445.1187*	*444.1114*
1100	*445.1187*	*444.1114*
1101	*445.1187*	*444.1114*
1102	*445.1187*	*444.1114*
1103	*445.1187*	*444.1114*
1104	*445.1187*	*444.1114*
1105	*445.1187*	*444.1114*
1106	*445.1187*	*444.1114*
1107	*445.1187*	*444.1114*
1108	*445.1187*	*444.1114*
1109	*445.1187*	*444.1114*
1110	*445.1187*	*444.1114*
1111	*445.1187*	*444.1114*
1112	*445.1188*	*444.1115*
1113	*445.1188*	*444.1115*
1114	*445.1188*	*444.1115*
1115	*445.1188*	*444.1115*
1116	*445.1188*	*444.1115*
1117	*445.1188*	*444.1115*
1118	*445.1188*	*444.1115*
1119	*445.1188*	*444.1115*
1120	*445.1188*	*444.1115*
1121	*445.1188*	*444.1115*
1122	*445.1188*	*444.1115*
1123	*445.1188*	*444.1115*
1124	*445.1188*	*444.1115*
1125	*445.1188*	*444.1115*
1126	*445.1188*	*444.1115*
1127	*445.1188*	*444.1115*
1128	*445.1188*	*444.1115*
1129	*445.1188*	*444.1115*
1130	*445.1188*	*444.1115*
1131	*445.1188*	*444.1115*
1132	*445.1188*	*444.1115*

1133	*445.1189*	*444.1116*
1134	*445.1189*	*444.1116*
1135	*445.1189*	*444.1116*
1136	*445.1189*	*444.1116*
1137	*445.1189*	*444.1116*
1138	*445.1189*	*444.1116*
1139	*445.1189*	*444.1116*
1140	*445.1189*	*444.1116*
1141	*445.1189*	*444.1116*
1142	*445.1189*	*444.1116*
1143	*445.1190*	*444.1117*
1144	*445.1190*	*444.1117*
1145	*445.1190*	*444.1117*
1146	*445.1190*	*444.1117*
1147	*445.1190*	*444.1117*
1148	*445.1190*	*444.1117*
1149	*445.1191*	*444.1118*
1150	*445.1194*	*444.1121*
1151	*445.1194*	*444.1121*
1152	*445.1194*	*444.1121*
1153	*445.1195*	*444.1122*
1154	*446.1189*	*445.1116*
1155	*449.2181*	*448.2108*
1156	*450.2681*	*449.2608*
1157	*450.2682*	*449.2609*
1158	*450.2683*	*449.2610*
1159	*450.2683*	*449.2610*
1160	*450.2684*	*449.2611*
1161	*450.2684*	*449.2611*
1162	*452.0604*	*451.0531*
1163	*452.0609*	*451.0536*
1164	*452.1753*	*451.1680*
1165	*452.1754*	*451.1681*
1166	*453.3416*	*452.3343*
1167	*455.3823*	*454.3750*
1168	*455.3827*	*454.3754*
1169	*455.3827*	*454.3754*
1170	*455.3827*	*454.3754*
1171	*455.3828*	*454.3755*
1172	*455.3829*	*454.3756*
1173	*455.3829*	*454.3756*
1174	*455.3830*	*454.3757*
1175	*455.3830*	*454.3757*
1176	*457.2107*	*456.2034*
1177	*457.2113*	*456.2040*
1178	*462.1440*	*461.1367*
1179	*462.1440*	*461.1367*
1180	*462.1441*	*461.1368*
1181	*462.1442*	*461.1369*
1182	*462.1442*	*461.1369*
1183	*462.1443*	*461.1370*
1184	*462.1443*	*461.1370*
1185	*462.1443*	*461.1370*
1186	*462.1443*	*461.1370*
1187	*462.1443*	*461.1370*
1188	*462.1443*	*461.1370*
1189	*462.1443*	*461.1370*
1190	*462.1443*	*461.1370*
1191	*462.1443*	*461.1370*
1192	*462.1444*	*461.1371*
1193	*462.1444*	*461.1371*
1194	*462.1445*	*461.1372*
1195	*462.1445*	*461.1372*
1196	*462.1446*	*461.1373*
1197	*462.1446*	*461.1373*
1198	*462.1446*	*461.1373*
1199	*462.1446*	*461.1373*
1200	*462.1446*	*461.1373*
1201	*462.1446*	*461.1373*
1202	*462.1446*	*461.1373*
1203	*462.1446*	*461.1373*
1204	*462.1446*	*461.1373*
1205	*462.1447*	*461.1374*
1206	*462.1447*	*461.1374*
1207	*462.1447*	*461.1374*
1208	*462.1447*	*461.1374*
1209	*462.1447*	*461.1374*
1210	*462.1447*	*461.1374*
1211	*462.1447*	*461.1374*
1212	*462.1447*	*461.1374*
1213	*462.1447*	*461.1374*
1214	*462.1447*	*461.1374*
1215	*462.1447*	*461.1374*
1216	*462.1447*	*461.1374*
1217	*462.1448*	*461.1375*
1218	*462.1448*	*461.1375*
1219	*462.1448*	*461.1375*
1220	*462.1448*	*461.1375*
1221	*462.1448*	*461.1375*
1222	*462.1448*	*461.1375*
1223	*462.1448*	*461.1375*
1224	*462.1448*	*461.1375*
1225	*462.1448*	*461.1375*
1226	*462.1448*	*461.1375*
1227	*462.1448*	*461.1375*
1228	*462.1448*	*461.1375*
1229	*462.1448*	*461.1375*
1230	*462.1448*	*461.1375*
1231	*462.1449*	*461.1376*
1232	*462.1449*	*461.1376*
1233	*462.1449*	*461.1376*

1234	*462.1449*	*461.1376*
1235	*462.1449*	*461.1376*
1236	*462.1449*	*461.1376*
1237	*462.1449*	*461.1376*
1238	*462.1449*	*461.1376*
1239	*462.1449*	*461.1376*
1240	*462.1449*	*461.1376*
1241	*462.1449*	*461.1376*
1242	*462.1449*	*461.1376*
1243	*462.1449*	*461.1376*
1244	*462.1449*	*461.1376*
1245	*462.1449*	*461.1376*
1246	*462.1449*	*461.1376*
1247	*462.1449*	*461.1376*
1248	*462.1449*	*461.1376*
1249	*462.1449*	*461.1376*
1250	*462.1449*	*461.1376*
1251	*462.1449*	*461.1376*
1252	*462.1449*	*461.1376*
1253	*462.1449*	*461.1376*
1254	*462.1450*	*461.1377*
1255	*462.1450*	*461.1377*
1256	*462.1450*	*461.1377*
1257	*462.1450*	*461.1377*
1258	*462.1450*	*461.1377*
1259	*462.1450*	*461.1377*
1260	*462.1450*	*461.1377*
1261	*462.1450*	*461.1377*
1262	*462.1450*	*461.1377*
1263	*462.1450*	*461.1377*
1264	*462.1450*	*461.1377*
1265	*462.1450*	*461.1377*
1266	*462.1450*	*461.1377*
1267	*462.1450*	*461.1377*
1268	*462.1450*	*461.1377*
1269	*462.1450*	*461.1377*
1270	*462.1450*	*461.1377*
1271	*462.1450*	*461.1377*
1272	*462.1450*	*461.1377*
1273	*462.1450*	*461.1377*
1274	*462.1450*	*461.1377*
1275	*462.1450*	*461.1377*
1276	*462.1450*	*461.1377*
1277	*462.1450*	*461.1377*
1278	*462.1450*	*461.1377*
1279	*462.1450*	*461.1377*
1280	*462.1450*	*461.1377*
1281	*462.1450*	*461.1377*
1282	*462.1450*	*461.1377*
1283	*462.1450*	*461.1377*
1284	*462.1450*	*461.1377*
1285	*462.1450*	*461.1377*
1286	*462.1450*	*461.1377*
1287	*462.1450*	*461.1377*
1288	*462.1451*	*461.1378*
1289	*462.1451*	*461.1378*
1290	*462.1451*	*461.1378*
1291	*462.1451*	*461.1378*
1292	*462.1451*	*461.1378*
1293	*462.1451*	*461.1378*
1294	*462.1451*	*461.1378*
1295	*462.1451*	*461.1378*
1296	*462.1451*	*461.1378*
1297	*462.1451*	*461.1378*
1298	*462.1451*	*461.1378*
1299	*462.1451*	*461.1378*
1300	*462.1451*	*461.1378*
1301	*462.1451*	*461.1378*
1302	*462.1451*	*461.1378*
1303	*462.1451*	*461.1378*
1304	*462.1451*	*461.1378*
1305	*462.1451*	*461.1378*
1306	*462.1451*	*461.1378*
1307	*462.1451*	*461.1378*
1308	*462.1451*	*461.1378*
1309	*462.1451*	*461.1378*
1310	*462.1451*	*461.1378*
1311	*462.1451*	*461.1378*
1312	*462.1451*	*461.1378*
1313	*462.1451*	*461.1378*
1314	*462.1451*	*461.1378*
1315	*462.1451*	*461.1378*
1316	*462.1451*	*461.1378*
1317	*462.1451*	*461.1378*
1318	*462.1451*	*461.1378*
1319	*462.1451*	*461.1378*
1320	*462.1451*	*461.1378*
1321	*462.1451*	*461.1378*
1322	*462.1451*	*461.1378*
1323	*462.1451*	*461.1378*
1324	*462.1451*	*461.1378*
1325	*462.1451*	*461.1378*
1326	*462.1451*	*461.1378*
1327	*462.1451*	*461.1378*
1328	*462.1451*	*461.1378*
1329	*462.1451*	*461.1378*
1330	*462.1452*	*461.1379*
1331	*462.1452*	*461.1379*
1332	*462.1452*	*461.1379*
1333	*462.1452*	*461.1379*
1334	*462.1452*	*461.1379*

1335	462.1452	461.1379
1336	462.1452	461.1379
1337	462.1452	461.1379
1338	462.1452	461.1379
1339	462.1452	461.1379
1340	462.1452	461.1379
1341	462.1452	461.1379
1342	462.1452	461.1379
1343	462.1452	461.1379
1344	462.1452	461.1379
1345	462.1452	461.1379
1346	462.1452	461.1379
1347	462.1452	461.1379
1348	462.1452	461.1379
1349	462.1452	461.1379
1350	462.1452	461.1379
1351	462.1452	461.1379
1352	462.1452	461.1379
1353	462.1452	461.1379
1354	462.1452	461.1379
1355	462.1452	461.1379
1356	462.1452	461.1379
1357	462.1452	461.1379
1358	462.1452	461.1379
1359	462.1452	461.1379
1360	462.1452	461.1379
1361	462.1452	461.1379
1362	462.1452	461.1379
1363	462.1452	461.1379
1364	462.1453	461.1380
1365	462.1453	461.1380
1366	462.1453	461.1380
1367	462.1453	461.1380
1368	462.1453	461.1380
1369	462.1453	461.1380
1370	462.1453	461.1380
1371	462.1453	461.1380
1372	462.1453	461.1380
1373	462.1453	461.1380
1374	462.1453	461.1380
1375	462.1453	461.1380
1376	462.1453	461.1380
1377	462.1453	461.1380
1378	462.1453	461.1380
1379	462.1453	461.1380
1380	462.1453	461.1380
1381	462.1453	461.1380
1382	462.1453	461.1380
1383	462.1453	461.1380
1384	462.1453	461.1380
1385	462.1453	461.1380
1386	462.1453	461.1380
1387	462.1453	461.1380
1388	462.1453	461.1380
1389	462.1454	461.1381
1390	462.1454	461.1381
1391	462.1454	461.1381
1392	462.1454	461.1381
1393	462.1454	461.1381
1394	462.1454	461.1381
1395	462.1454	461.1381
1396	462.1454	461.1381
1397	462.1454	461.1381
1398	462.1454	461.1381
1399	462.1454	461.1381
1400	462.1454	461.1381
1401	462.1454	461.1381
1402	462.1454	461.1381
1403	462.1454	461.1381
1404	462.1454	461.1381
1405	462.1454	461.1381
1406	462.1454	461.1381
1407	462.1455	461.1382
1408	462.1455	461.1382
1409	462.1455	461.1382
1410	462.1455	461.1382
1411	462.1455	461.1382
1412	462.1455	461.1382
1413	462.1456	461.1383
1414	462.1456	461.1383
1415	462.1456	461.1383
1416	462.1457	461.1384
1417	462.1457	461.1384
1418	462.1457	461.1384
1419	462.1458	461.1385
1420	462.1458	461.1385
1421	463.1453	462.1380
1422	466.2780	465.2707
1423	466.2781	465.2708
1424	468.3878	467.3805
1425	468.3879	467.3806
1426	468.3879	467.3806
1427	468.3879	467.3806
1428	468.3879	467.3806
1429	468.3880	467.3807
1430	468.3880	467.3807
1431	468.3880	467.3807
1432	468.3880	467.3807
1433	468.3881	467.3808
1434	468.3881	467.3808
1435	468.3881	467.3808

1436	468.3881	467.3808
1437	468.3881	467.3808
1438	468.3881	467.3808
1439	468.3882	467.3809
1440	468.3882	467.3809
1441	468.3882	467.3809
1442	468.3882	467.3809
1443	468.3883	467.3810
1444	468.3883	467.3810
1445	468.3884	467.3811
1446	468.3884	467.3811
1447	468.3884	467.3811
1448	468.3884	467.3811
1449	468.3885	467.3812
1450	468.3885	467.3812
1451	468.3886	467.3813
1452	469.2757	468.2684
1453	469.2761	468.2688
1454	471.2376	470.2303
1455	473.0712	472.0639
1456	476.3040	475.2967
1457	476.3044	475.2971
1458	476.3051	475.2978
1459	476.3051	475.2978
1460	477.3644	476.3571
1461	477.3644	476.3571
1462	477.3645	476.3572
1463	477.3648	476.3575
1464	484.2275	483.2202
1465	484.2278	483.2205
1466	484.2279	483.2206
1467	484.2279	483.2206
1468	484.2280	483.2207
1469	484.2280	483.2207
1470	484.2280	483.2207
1471	486.1530	485.1457
1472	487.3725	486.3652
1473	487.3727	486.3654
1474	487.3727	486.3654
1475	487.3728	486.3655
1476	491.2831	490.2758
1477	491.2833	490.2760
1478	494.4039	493.3966
1479	494.4040	493.3967
1480	496.2746	495.2673
1481	496.2751	495.2678
1482	498.1806	497.1733
1483	498.1808	497.1735
1484	498.1810	497.1737
1485	498.1810	497.1737
1486	498.1813	497.1740
1487	498.1814	497.1741
1488	502.3203	501.3130
1489	502.3209	501.3136
1490	502.3215	501.3142
1491	502.3217	501.3144
1492	504.3182	503.3109
1493	504.3183	503.3110
1494	504.3183	503.3110
1495	504.3185	503.3112
1496	504.3185	503.3112
1497	504.3185	503.3112
1498	504.3188	503.3115
1499	504.3188	503.3115
1500	504.3188	503.3115
1501	504.3188	503.3115
1502	504.3188	503.3115
1503	504.3189	503.3116
1504	504.3189	503.3116
1505	504.3192	503.3119
1506	504.3192	503.3119
1507	504.3192	503.3119
1508	504.3195	503.3122
1509	505.0429	504.0356
1510	505.0432	504.0359
1511	508.3097	507.3024
1512	508.3101	507.3028
1513	508.3107	507.3034
1514	508.3109	507.3036
1515	509.3542	508.3469
1516	509.3546	508.3473
1517	509.3547	508.3474
1518	509.3550	508.3477
1519	512.4136	511.4063
1520	512.4137	511.4064
1521	512.4138	511.4065
1522	512.4138	511.4065
1523	512.4138	511.4065
1524	512.4139	511.4066
1525	512.4141	511.4068
1526	512.4141	511.4068
1527	512.4142	511.4069
1528	512.4143	511.4070
1529	512.4143	511.4070
1530	512.4143	511.4070
1531	512.4143	511.4070
1532	512.4144	511.4071
1533	512.4146	511.4073
1534	512.4146	511.4073
1535	512.4146	511.4073
1536	512.4147	511.4074

1537	*512.4149*	*511.4076*
1538	*512.4149*	*511.4076*
1539	*512.4150*	*511.4077*
1540	*518.2070*	*517.1997*
1541	*518.2072*	*517.1999*
1542	*518.3151*	*517.3078*
1543	*519.1356*	*518.1283*
1544	*519.1360*	*518.1287*
1545	*519.1360*	*518.1287*
1546	*519.1361*	*518.1288*
1547	*519.1362*	*518.1289*
1548	*519.1364*	*518.1291*
1549	*519.1365*	*518.1292*
1550	*519.1365*	*518.1292*
1551	*519.1365*	*518.1292*
1552	*519.1366*	*518.1293*
1553	*519.1366*	*518.1293*
1554	*519.1366*	*518.1293*
1555	*519.1366*	*518.1293*
1556	*519.1367*	*518.1294*
1557	*519.1367*	*518.1294*
1558	*519.1367*	*518.1294*
1559	*519.1367*	*518.1294*
1560	*519.1367*	*518.1294*
1561	*519.1367*	*518.1294*
1562	*519.1367*	*518.1294*
1563	*519.1367*	*518.1294*
1564	*519.1367*	*518.1294*
1565	*519.1367*	*518.1294*
1566	*519.1367*	*518.1294*
1567	*519.1367*	*518.1294*
1568	*519.1367*	*518.1294*
1569	*519.1367*	*518.1294*
1570	*519.1367*	*518.1294*
1571	*519.1367*	*518.1294*
1572	*519.1368*	*518.1295*
1573	*519.1368*	*518.1295*
1574	*519.1368*	*518.1295*
1575	*519.1368*	*518.1295*
1576	*519.1368*	*518.1295*
1577	*519.1368*	*518.1295*
1578	*519.1368*	*518.1295*
1579	*519.1368*	*518.1295*
1580	*519.1368*	*518.1295*
1581	*519.1368*	*518.1295*
1582	*519.1368*	*518.1295*
1583	*519.1368*	*518.1295*
1584	*519.1368*	*518.1295*
1585	*519.1368*	*518.1295*
1586	*519.1368*	*518.1295*
1587	*519.1368*	*518.1295*
1588	*519.1368*	*518.1295*
1589	*519.1368*	*518.1295*
1590	*519.1368*	*518.1295*
1591	*519.1368*	*518.1295*
1592	*519.1368*	*518.1295*
1593	*519.1368*	*518.1295*
1594	*519.1369*	*518.1296*
1595	*519.1369*	*518.1296*
1596	*519.1369*	*518.1296*
1597	*519.1369*	*518.1296*
1598	*519.1369*	*518.1296*
1599	*519.1369*	*518.1296*
1600	*519.1369*	*518.1296*
1601	*519.1369*	*518.1296*
1602	*519.1369*	*518.1296*
1603	*519.1369*	*518.1296*
1604	*519.1369*	*518.1296*
1605	*519.1369*	*518.1296*
1606	*519.1369*	*518.1296*
1607	*519.1369*	*518.1296*
1608	*519.1369*	*518.1296*
1609	*519.1370*	*518.1297*
1610	*519.1370*	*518.1297*
1611	*519.1370*	*518.1297*
1612	*519.1370*	*518.1297*
1613	*519.1370*	*518.1297*
1614	*519.1370*	*518.1297*
1615	*519.1370*	*518.1297*
1616	*519.1370*	*518.1297*
1617	*519.1370*	*518.1297*
1618	*519.1370*	*518.1297*
1619	*519.1370*	*518.1297*
1620	*519.1370*	*518.1297*
1621	*519.1370*	*518.1297*
1622	*519.1370*	*518.1297*
1623	*519.1370*	*518.1297*
1624	*519.1370*	*518.1297*
1625	*519.1370*	*518.1297*
1626	*519.1370*	*518.1297*
1627	*519.1370*	*518.1297*
1628	*519.1370*	*518.1297*
1629	*519.1370*	*518.1297*
1630	*519.1370*	*518.1297*
1631	*519.1370*	*518.1297*
1632	*519.1370*	*518.1297*
1633	*519.1370*	*518.1297*
1634	*519.1370*	*518.1297*
1635	*519.1370*	*518.1297*
1636	*519.1370*	*518.1297*
1637	*519.1371*	*518.1298*

1638	519.1371	518.1298
1639	519.1371	518.1298
1640	519.1371	518.1298
1641	519.1371	518.1298
1642	519.1371	518.1298
1643	519.1371	518.1298
1644	519.1371	518.1298
1645	519.1371	518.1298
1646	519.1371	518.1298
1647	519.1371	518.1298
1648	519.1371	518.1298
1649	519.1371	518.1298
1650	519.1371	518.1298
1651	519.1371	518.1298
1652	519.1371	518.1298
1653	519.1371	518.1298
1654	519.1372	518.1299
1655	519.1372	518.1299
1656	519.1372	518.1299
1657	519.1372	518.1299
1658	519.1372	518.1299
1659	519.1372	518.1299
1660	519.1372	518.1299
1661	519.1372	518.1299
1662	519.1373	518.1300
1663	519.1373	518.1300
1664	519.1373	518.1300
1665	519.1373	518.1300
1666	519.1373	518.1300
1667	519.1373	518.1300
1668	519.1374	518.1301
1669	519.1374	518.1301
1670	519.1374	518.1301
1671	519.1375	518.1302
1672	519.1375	518.1302
1673	519.1375	518.1302
1674	519.1375	518.1302
1675	519.1375	518.1302
1676	519.1376	518.1303
1677	519.1376	518.1303
1678	519.1379	518.1306
1679	520.3300	519.3227
1680	520.3303	519.3230
1681	520.3306	519.3233
1682	520.3306	519.3233
1683	520.3311	519.3238
1684	520.3314	519.3241
1685	529.3833	528.3760
1686	529.3833	528.3760
1687	529.3833	528.3760
1688	529.3835	528.3762
1689	536.1622	535.1549
1690	536.1624	535.1551
1691	536.1624	535.1551
1692	536.1625	535.1552
1693	536.1625	535.1552
1694	536.1626	535.1553
1695	536.1628	535.1555
1696	536.1628	535.1555
1697	536.1628	535.1555
1698	536.1628	535.1555
1699	536.1628	535.1555
1700	536.1628	535.1555
1701	536.1628	535.1555
1702	536.1629	535.1556
1703	536.1629	535.1556
1704	536.1629	535.1556
1705	536.1629	535.1556
1706	536.1630	535.1557
1707	536.1630	535.1557
1708	536.1630	535.1557
1709	536.1630	535.1557
1710	536.1630	535.1557
1711	536.1630	535.1557
1712	536.1630	535.1557
1713	536.1630	535.1557
1714	536.1631	535.1558
1715	536.1631	535.1558
1716	536.1631	535.1558
1717	536.1631	535.1558
1718	536.1631	535.1558
1719	536.1631	535.1558
1720	536.1631	535.1558
1721	536.1631	535.1558
1722	536.1631	535.1558
1723	536.1631	535.1558
1724	536.1632	535.1559
1725	536.1632	535.1559
1726	536.1632	535.1559
1727	536.1632	535.1559
1728	536.1632	535.1559
1729	536.1632	535.1559
1730	536.1632	535.1559
1731	536.1632	535.1559
1732	536.1632	535.1559
1733	536.1632	535.1559
1734	536.1633	535.1560
1735	536.1633	535.1560
1736	536.1633	535.1560
1737	536.1633	535.1560
1738	536.1633	535.1560

1739	*536.1633*	*535.1560*
1740	*536.1633*	*535.1560*
1741	*536.1633*	*535.1560*
1742	*536.1633*	*535.1560*
1743	*536.1633*	*535.1560*
1744	*536.1633*	*535.1560*
1745	*536.1633*	*535.1560*
1746	*536.1633*	*535.1560*
1747	*536.1633*	*535.1560*
1748	*536.1633*	*535.1560*
1749	*536.1633*	*535.1560*
1750	*536.1633*	*535.1560*
1751	*536.1634*	*535.1561*
1752	*536.1634*	*535.1561*
1753	*536.1634*	*535.1561*
1754	*536.1634*	*535.1561*
1755	*536.1634*	*535.1561*
1756	*536.1634*	*535.1561*
1757	*536.1634*	*535.1561*
1758	*536.1634*	*535.1561*
1759	*536.1634*	*535.1561*
1760	*536.1634*	*535.1561*
1761	*536.1634*	*535.1561*
1762	*536.1634*	*535.1561*
1763	*536.1634*	*535.1561*
1764	*536.1634*	*535.1561*
1765	*536.1635*	*535.1562*
1766	*536.1635*	*535.1562*
1767	*536.1635*	*535.1562*
1768	*536.1635*	*535.1562*
1769	*536.1635*	*535.1562*
1770	*536.1635*	*535.1562*
1771	*536.1635*	*535.1562*
1772	*536.1635*	*535.1562*
1773	*536.1635*	*535.1562*
1774	*536.1635*	*535.1562*
1775	*536.1635*	*535.1562*
1776	*536.1635*	*535.1562*
1777	*536.1635*	*535.1562*
1778	*536.1635*	*535.1562*
1779	*536.1635*	*535.1562*
1780	*536.1635*	*535.1562*
1781	*536.1635*	*535.1562*
1782	*536.1635*	*535.1562*
1783	*536.1635*	*535.1562*
1784	*536.1635*	*535.1562*
1785	*536.1635*	*535.1562*
1786	*536.1635*	*535.1562*
1787	*536.1635*	*535.1562*
1788	*536.1635*	*535.1562*
1789	*536.1635*	*535.1562*
1790	*536.1635*	*535.1562*
1791	*536.1635*	*535.1562*
1792	*536.1635*	*535.1562*
1793	*536.1635*	*535.1562*
1794	*536.1635*	*535.1562*
1795	*536.1635*	*535.1562*
1796	*536.1635*	*535.1562*
1797	*536.1636*	*535.1563*
1798	*536.1636*	*535.1563*
1799	*536.1636*	*535.1563*
1800	*536.1636*	*535.1563*
1801	*536.1636*	*535.1563*
1802	*536.1636*	*535.1563*
1803	*536.1636*	*535.1563*
1804	*536.1636*	*535.1563*
1805	*536.1636*	*535.1563*
1806	*536.1636*	*535.1563*
1807	*536.1636*	*535.1563*
1808	*536.1636*	*535.1563*
1809	*536.1636*	*535.1563*
1810	*536.1636*	*535.1563*
1811	*536.1636*	*535.1563*
1812	*536.1636*	*535.1563*
1813	*536.1636*	*535.1563*
1814	*536.1636*	*535.1563*
1815	*536.1636*	*535.1563*
1816	*536.1636*	*535.1563*
1817	*536.1636*	*535.1563*
1818	*536.1636*	*535.1563*
1819	*536.1636*	*535.1563*
1820	*536.1636*	*535.1563*
1821	*536.1636*	*535.1563*
1822	*536.1636*	*535.1563*
1823	*536.1636*	*535.1563*
1824	*536.1636*	*535.1563*
1825	*536.1636*	*535.1563*
1826	*536.1636*	*535.1563*
1827	*536.1637*	*535.1564*
1828	*536.1637*	*535.1564*
1829	*536.1637*	*535.1564*
1830	*536.1637*	*535.1564*
1831	*536.1637*	*535.1564*
1832	*536.1637*	*535.1564*
1833	*536.1637*	*535.1564*
1834	*536.1637*	*535.1564*
1835	*536.1637*	*535.1564*
1836	*536.1637*	*535.1564*
1837	*536.1637*	*535.1564*

1838	536.1637	535.1564
1839	536.1637	535.1564
1840	536.1637	535.1564
1841	536.1637	535.1564
1842	536.1637	535.1564
1843	536.1637	535.1564
1844	536.1637	535.1564
1845	536.1637	535.1564
1846	536.1638	535.1565
1847	536.1638	535.1565
1848	536.1638	535.1565
1849	536.1638	535.1565
1850	536.1638	535.1565
1851	536.1638	535.1565
1852	536.1638	535.1565
1853	536.1638	535.1565
1854	536.1638	535.1565
1855	536.1638	535.1565
1856	536.1638	535.1565
1857	536.1638	535.1565
1858	536.1638	535.1565
1859	536.1638	535.1565
1860	536.1638	535.1565
1861	536.1638	535.1565
1862	536.1638	535.1565
1863	536.1638	535.1565
1864	536.1638	535.1565
1865	536.1638	535.1565
1866	536.1638	535.1565
1867	536.1638	535.1565
1868	536.1638	535.1565
1869	536.1638	535.1565
1870	536.1638	535.1565
1871	536.1638	535.1565
1872	536.1638	535.1565
1873	536.1638	535.1565
1874	536.1638	535.1565
1875	536.1638	535.1565
1876	536.1638	535.1565
1877	536.1638	535.1565
1878	536.1638	535.1565
1879	536.1638	535.1565
1880	536.1638	535.1565
1881	536.1639	535.1566
1882	536.1639	535.1566
1883	536.1639	535.1566
1884	536.1639	535.1566
1885	536.1639	535.1566
1886	536.1639	535.1566
1887	536.1639	535.1566
1888	536.1639	535.1566
1889	536.1639	535.1566
1890	536.1639	535.1566
1891	536.1639	535.1566
1892	536.1639	535.1566
1893	536.1639	535.1566
1894	536.1639	535.1566
1895	536.1639	535.1566
1896	536.1639	535.1566
1897	536.1639	535.1566
1898	536.1639	535.1566
1899	536.1639	535.1566
1900	536.1639	535.1566
1901	536.1640	535.1567
1902	536.1640	535.1567
1903	536.1641	535.1568
1904	536.1641	535.1568
1905	536.1641	535.1568
1906	536.1642	535.1569
1907	536.1642	535.1569
1908	536.1642	535.1569
1909	536.1642	535.1569
1910	536.1642	535.1569
1911	536.1644	535.1571
1912	536.1645	535.1572
1913	536.1646	535.1573
1914	540.2842	539.2769
1915	542.2060	541.1987
1916	542.2065	541.1992
1917	542.2066	541.1993
1918	542.2070	541.1997
1919	542.2070	541.1997
1920	542.2070	541.1997
1921	542.2070	541.1997
1922	542.2072	541.1999
1923	542.2073	541.2000
1924	542.2075	541.2002
1925	542.2077	541.2004
1926	542.2078	541.2005
1927	544.2426	543.2353
1928	544.2426	543.2353
1930	548.3451	547.3378
1931	548.3452	547.3379
1932	556.4397	555.4324
1934	556.4404	555.4331
1935	556.4406	555.4333
1936	556.4407	555.4334
1937	556.4410	555.4337
1938	556.4410	555.4337
1940	558.2010	557.1937
1941	558.2014	557.1941

1942	558.2016	557.1943
1943	558.2021	557.1948
1944	558.4188	557.4115
1947	559.2329	558.2256
1948	559.2332	558.2259
1949	559.2332	558.2259
1950	559.2335	558.2262
1951	559.2335	558.2262
1952	559.2336	558.2263
1953	559.2336	558.2263
1954	559.2338	558.2265
1955	559.2344	558.2271
1956	560.2172	559.2099
1957	560.2173	559.2100
1958	560.2174	559.2101
1959	560.2818	559.2745
1960	560.6945	559.6872
1962	563.5486	562.5413
1963	563.5487	562.5414
1964	563.5488	562.5415
1965	563.5489	562.5416
1966	563.5490	562.5417
1967	563.5490	562.5417
1968	563.5490	562.5417
1969	563.5491	562.5418
1970	563.5491	562.5418
1971	563.5491	562.5418
1972	563.5491	562.5418
1973	563.5491	562.5418
1974	563.5491	562.5418
1975	563.5492	562.5419
1976	563.5492	562.5419
1977	563.5492	562.5419
1978	563.5492	562.5419
1979	563.5492	562.5419
1980	563.5492	562.5419
1981	563.5493	562.5420
1982	563.5493	562.5420
1983	563.5493	562.5420
1984	563.5493	562.5420
1985	563.5493	562.5420
1986	563.5493	562.5420
1987	563.5493	562.5420
1988	563.5493	562.5420
1989	563.5493	562.5420
1990	563.5494	562.5421
1991	563.5494	562.5421
1992	563.5494	562.5421
1993	563.5494	562.5421
1994	563.5494	562.5421
1995	563.5494	562.5421
1996	563.5494	562.5421
1997	563.5494	562.5421
1998	563.5494	562.5421
1999	563.5494	562.5421
2000	563.5494	562.5421
2001	563.5494	562.5421
2002	563.5495	562.5422
2003	563.5495	562.5422
2004	563.5496	562.5423
2005	563.5496	562.5423
2006	563.5496	562.5423
2007	563.5496	562.5423
2008	563.5497	562.5424
2009	563.5497	562.5424
2010	563.5499	562.5426
2011	563.5499	562.5426
2012	563.5499	562.5426
2013	563.5500	562.5427
2014	563.5500	562.5427
2015	563.5505	562.5432
2016	564.1890	563.1817
2017	564.1891	563.1818
2018	564.2993	563.2920
2019	564.2995	563.2922
2020	564.3561	563.3488
2021	564.3563	563.3490
2022	564.3566	563.3493
2023	564.3569	563.3496
2024	566.4246	565.4173
2025	566.4254	565.4181
2026	566.4256	565.4183
2027	566.4257	565.4184
2028	566.4257	565.4184
2029	566.4258	565.4185
2030	566.4265	565.4192
2031	570.0429	569.0356
2032	574.2536	573.2463
2033	574.2537	573.2464
2036	575.7743	574.7670
2037	586.2328	585.2255
2038	586.2328	585.2255
2039	586.2333	585.2260
2040	586.2334	585.2261
2041	586.5380	585.5307
2042	586.5386	585.5313
2043	586.5388	585.5315
2044	586.5390	585.5317
2045	586.5396	585.5323
2046	586.5400	585.5327
2047	591.1967	590.1894

2048	*591.1974*	*590.1901*
2049	*592.1724*	*591.1651*
2050	*592.1738*	*591.1665*
2051	*592.3701*	*591.3628*
2052	*592.3703*	*591.3630*
2053	*592.3705*	*591.3632*
2054	*592.3709*	*591.3636*
2055	*592.3710*	*591.3637*
2056	*592.3711*	*591.3638*
2058	*592.3713*	*591.3640*
2059	*592.3715*	*591.3642*
2060	*592.3716*	*591.3643*
2061	*592.3716*	*591.3643*
2062	*592.3718*	*591.3645*
2063	*601.3887*	*600.3814*
2064	*601.3894*	*600.3821*
2065	*601.3895*	*600.3822*
2067	*602.2297*	*601.2224*
2069	*603.2595*	*602.2522*
2070	*603.2595*	*602.2522*
2071	*603.2597*	*602.2524*
2072	*603.2597*	*602.2524*
2073	*603.2599*	*602.2526*
2074	*603.2601*	*602.2528*
2075	*603.2602*	*602.2529*
2076	*604.2433*	*603.2360*
2077	*604.2436*	*603.2363*
2078	*604.2638*	*603.2565*
2079	*604.2639*	*603.2566*
2080	*604.2640*	*603.2567*
2081	*604.2642*	*603.2569*
2082	*608.3825*	*607.3752*
2083	*608.3827*	*607.3754*
2084	*610.1805*	*609.1732*
2085	*610.1808*	*609.1735*
2086	*610.1808*	*609.1735*
2087	*610.1810*	*609.1737*
2088	*610.1810*	*609.1737*
2089	*610.1810*	*609.1737*
2090	*610.1811*	*609.1738*
2091	*610.1811*	*609.1738*
2092	*610.1812*	*609.1739*
2093	*610.1812*	*609.1739*
2094	*610.1812*	*609.1739*
2095	*610.1812*	*609.1739*
2096	*610.1812*	*609.1739*
2097	*610.1812*	*609.1739*
2098	*610.1813*	*609.1740*
2099	*610.1813*	*609.1740*
2100	*610.1813*	*609.1740*
2101	*610.1813*	*609.1740*
2102	*610.1813*	*609.1740*
2103	*610.1813*	*609.1740*
2104	*610.1813*	*609.1740*
2105	*610.1813*	*609.1740*
2106	*610.1813*	*609.1740*
2107	*610.1813*	*609.1740*
2108	*610.1814*	*609.1741*
2109	*610.1814*	*609.1741*
2110	*610.1814*	*609.1741*
2111	*610.1814*	*609.1741*
2112	*610.1814*	*609.1741*
2113	*610.1815*	*609.1742*
2114	*610.1815*	*609.1742*
2115	*610.1815*	*609.1742*
2116	*610.1815*	*609.1742*
2117	*610.1815*	*609.1742*
2118	*610.1816*	*609.1743*
2119	*610.1816*	*609.1743*
2120	*610.1816*	*609.1743*
2121	*610.1816*	*609.1743*
2122	*610.1816*	*609.1743*
2123	*610.1816*	*609.1743*
2124	*610.1816*	*609.1743*
2125	*610.1816*	*609.1743*
2126	*610.1816*	*609.1743*
2127	*610.1816*	*609.1743*
2128	*610.1816*	*609.1743*
2129	*610.1816*	*609.1743*
2130	*610.1816*	*609.1743*
2131	*610.1816*	*609.1743*
2132	*610.1816*	*609.1743*
2133	*610.1816*	*609.1743*
2134	*610.1816*	*609.1743*
2135	*610.1817*	*609.1744*
2136	*610.1817*	*609.1744*
2137	*610.1817*	*609.1744*
2138	*610.1818*	*609.1745*
2139	*610.1818*	*609.1745*
2140	*610.1818*	*609.1745*
2141	*610.1818*	*609.1745*
2142	*610.1818*	*609.1745*
2143	*610.1818*	*609.1745*
2144	*610.1818*	*609.1745*
2145	*610.1818*	*609.1745*
2146	*610.1818*	*609.1745*
2147	*610.1818*	*609.1745*
2148	*610.1818*	*609.1745*
2149	*610.1818*	*609.1745*
2150	*610.1818*	*609.1745*

2151	610.1818	609.1745
2152	610.1818	609.1745
2153	610.1818	609.1745
2154	610.1818	609.1745
2155	610.1818	609.1745
2156	610.1818	609.1745
2157	610.1818	609.1745
2158	610.1818	609.1745
2159	610.1818	609.1745
2160	610.1818	609.1745
2161	610.1818	609.1745
2162	610.1818	609.1745
2163	610.1818	609.1745
2164	610.1818	609.1745
2165	610.1818	609.1745
2166	610.1818	609.1745
2167	610.1818	609.1745
2168	610.1818	609.1745
2169	610.1818	609.1745
2170	610.1819	609.1746
2171	610.1819	609.1746
2172	610.1819	609.1746
2173	610.1819	609.1746
2174	610.1819	609.1746
2175	610.1819	609.1746
2176	610.1819	609.1746
2177	610.1819	609.1746
2178	610.1819	609.1746
2179	610.1819	609.1746
2180	610.1819	609.1746
2181	610.1819	609.1746
2182	610.1819	609.1746
2183	610.1819	609.1746
2184	610.1819	609.1746
2185	610.1820	609.1747
2186	610.1820	609.1747
2187	610.1820	609.1747
2188	610.1820	609.1747
2189	610.1820	609.1747
2190	610.1820	609.1747
2191	610.1820	609.1747
2192	610.1820	609.1747
2193	610.1820	609.1747
2194	610.1821	609.1748
2195	610.1821	609.1748
2196	610.1821	609.1748
2197	610.1821	609.1748
2198	610.1821	609.1748
2199	610.1821	609.1748
2200	610.1821	609.1748
2201	610.1821	609.1748
2202	610.1821	609.1748
2203	610.1821	609.1748
2204	610.1821	609.1748
2205	610.1821	609.1748
2206	610.1821	609.1748
2207	610.1821	609.1748
2208	610.1821	609.1748
2209	610.1821	609.1748
2210	610.1821	609.1748
2211	610.1821	609.1748
2212	610.1821	609.1748
2213	610.1822	609.1749
2214	610.1822	609.1749
2215	610.1822	609.1749
2216	610.1822	609.1749
2217	610.1822	609.1749
2218	610.1822	609.1749
2219	610.1823	609.1750
2220	610.1823	609.1750
2221	610.1823	609.1750
2222	610.1823	609.1750
2223	610.1823	609.1750
2224	610.1823	609.1750
2225	610.1823	609.1750
2226	610.1823	609.1750
2227	610.1823	609.1750
2228	610.1823	609.1750
2229	610.1823	609.1750
2230	610.1824	609.1751
2231	610.1824	609.1751
2232	610.1824	609.1751
2233	610.1824	609.1751
2234	610.1824	609.1751
2235	610.1824	609.1751
2236	610.1824	609.1751
2237	610.1824	609.1751
2238	610.1824	609.1751
2239	610.1824	609.1751
2240	610.1824	609.1751
2241	610.1824	609.1751
2242	610.1824	609.1751
2243	610.1825	609.1752
2244	610.1825	609.1752
2245	610.1825	609.1752
2246	610.1825	609.1752
2247	610.1825	609.1752
2248	610.1826	609.1753
2249	610.1826	609.1753
2250	610.1826	609.1753
2251	610.1826	609.1753

☒	2252	610.1826	609.1753
☒	2253	610.1827	609.1754
☒	2254	610.1827	609.1754
☒	2255	610.1827	609.1754
☒	2256	610.1828	609.1755
☒	2257	610.1829	609.1756
☒	2258	610.1829	609.1756
☒	2259	610.1832	609.1759
☒	2260	610.1833	609.1760
☒	2261	610.1835	609.1762
☒	2262	611.7903	610.7830
☒	2263	617.0736	616.0663
☒	2266	619.2535	618.2462
☒	2267	619.2540	618.2467
☒	2268	619.2543	618.2470
☒	2269	619.2546	618.2473
☒	2270	619.2546	618.2473
☒	2273	626.9494	625.9421
☒	2274	627.3001	626.2928
☒	2275	627.5167	626.5094
☒	2280	634.2743	633.2670
☒	2281	634.2743	633.2670
☒	2286	636.3964	635.3891
☒	2288	636.3972	635.3899
☒	2290	636.3973	635.3900
☒	2291	636.3973	635.3900
☒	2292	636.3973	635.3900
☒	2293	636.3973	635.3900
☒	2294	636.3974	635.3901
☒	2298	636.9732	635.9659
☒	2299	640.9093	639.9020
☒	2305	644.4498	643.4425
☒	2308	647.2861	646.2788
☒	2312	649.1923	648.1850
☒	2313	649.1927	648.1854
☒	2314	649.1928	648.1855
☒	2315	649.1931	648.1858
☒	2316	650.1772	649.1699
☒	2317	650.1772	649.1699
☒	2320	652.3658	651.3585
☒	2321	652.4521	651.4448
☒	2325	656.6054	655.5981
☒	2326	656.7610	655.7537
☒	2328	656.9602	655.9529
☒	2334	663.5466	662.5393
☒	2335	664.2845	663.2772
☒	2336	664.2846	663.2773
☒	2337	664.2846	663.2773
☒	2339	669.2750	668.2677
☒	2342	671.7693	670.7620
☒	2348	676.8366	675.8293
☒	2350	680.4225	679.4152
☒	2352	681.3304	680.3231
☒	2353	681.5291	680.5218
☒	2354	682.3452	681.3379
☒	2357	683.9089	682.9016
☒	2360	684.2001	683.1928
☒	2361	684.2008	683.1935
☒	2377	694.9218	693.9145
☒	2378	696.3260	695.3187
☒	2379	696.4340	695.4267
☒	2384	702.8548	701.8475
☒	2389	707.6905	706.6832
☒	2404	710.6031	709.5958
☒	2411	713.6995	712.6922
☒	2425	725.3129	724.3056
☒	2431	728.6318	727.6245
☒	2448	736.4614	735.4541
☒	2478	760.8334	759.8261
☒	2483	767.0557	766.0484
☒	2484	767.0599	766.0526
☒	2485	767.0612	766.0539
☒	2486	767.0645	766.0572
☒	2506	780.2208	779.2135
☒	7031	1014.0850	3039.2332
☒	8168	1086.2505	4340.9729
☒	8763	1148.9490	5739.7086
☒	8782	1449.9296	5795.6893
☒	8786	1449.9346	5795.7093
☒	8810	1450.1835	5796.7049
☒	8817	1450.1855	5796.7129
☒	8820	1450.1859	5796.7145
☒	8826	1160.3527	5796.7271
☒	8901	858.8069	6004.5974

Search Parameters

```
Type of search         : MS/MS Ion Search
Enzyme                 : Trypsin
Fixed modifications    : Carbamidomethyl (C)
Variable modifications : Oxidation (M)
Mass values            : Monoisotopic
Protein Mass           : Unrestricted
Peptide Mass Tolerance : ± 1.2 Da
Fragment Mass Tolerance: ± 0.6 Da
Max Missed Cleavages   : 1
Instrument type        : ESI-FTICR
Number of queries      : 8948
```
